# Supplementary material for: Mechanistic Investigation of the Pyrolysis Temperature of Reed Wood Vinegar for Maximising the Antibacterial Activity of Escherichia coli and Its Inhibitory Activity
Source: Biology (Basel). 2024 Nov 8;13(11):912. doi: 10.3390/biology13110912 (PMC11592125; doi:10.3390/biology13110912)
Supplement: Supplementary file 1 [file biology-13-00912-s001.zip › S1. List of abbreviations.pdf]

List of abbreviations

Abbreviations	Full name
ATP	Adenosine triphosphate
ABC	ATP-binding cassette
BCA	Bicinchoninic Acid Assay
BHI	Brain-Heart Infusion Broth
CAMP	cationic antimicrobial peptide
COG	Cluster of Orthologous Groups
DEGs	Differential Expressed Genes
E. coli	*Escherichia coli*
GO	Gene Ontology
KEGG	Kyoto Encyclopedia of Genes and Genomes
L. acidophilus	*Lactobacillus acidophilus*
LB	Luria-Bertani
MDA	Malondialdehyde
MBP	Maltose Binding Protein
MIC	Minimum Inhibitory Concentration
MLST	Multilocus Sequence Typing
NCBI	National Council for Biotechnology
NO	Nitric Oxide
PBS	Phosphate Buffered Saline
PCA	Principal Component Qnalysis
P. aeruginosa	*Pseudomonas aeruginosa*
qPCR	Quantitative Polymerase Chain Reaction
ROS	Reactive Oxygen Species
RNA-Seq	RNA Sequencing
S. enterica	*Salmonella enterica*
SEM	Scanning Electron Microscopy
ST	Sequence Type
S. aureus	*Staphylococcus aureus*
TEM	Transmission Electron Microscopy
