# Supplementary material for: Mechanistic Investigation of the Pyrolysis Temperature of Reed Wood Vinegar for Maximising the Antibacterial Activity of Escherichia coli and Its Inhibitory Activity
Source: Biology (Basel). 2024 Nov 8;13(11):912. doi: 10.3390/biology13110912 (PMC11592125; doi:10.3390/biology13110912)
Supplement: Supplementary file 1 [file biology-13-00912-s001.zip › S2. The Wood vinegar preparation method.pdf]

# The Wood vinegar preparation method

## 1. Pre-treatment of reeds

In this study, naturally dried reeds were used, which were crushed and then made into cylindrical pellets with a diameter of about 0.5 cm and a length of about 3 cm by a compression granulator.

## 2.Pyrolysis parameters of reed wood vinegar

The reed pellets were evenly spread in a 304s stainless steel basin and then placed into a high temperature pyrolysis carbonisation furnace (this is a customised furnace) for pyrolysis. The heating rate of the furnace was set at 10 °C/min, and nitrogen was continuously fed at a flow rate of 3 mL/min, and the pyrolysis reaction was maintained for 4 h after reaching the target temperatures (set temperatures of 300°C, 500°C, and 700°C, respectively).

## 3. Crude reed wood vinegar collection and purification of impurities refining process

The flue gas generated in the pyrolysis process will produce condensed liquid when passing through the discharge pipe, the flue gas condensed liquid in the pyrolysis process was collected to obtain the crude reed wood vinegar at the corresponding temperature, which was collected and sealed in brown bottles for storage. After that, the thicker impurities in the crude wood vinegar were filtered out by qualitative filter paper, and a small amount of activated carbon was added to the liquid obtained (the added amount was about one tenth of the volume of the wood vinegar), and then sealed and put into a constant temperature oscillation incubator to oscillate for 12 h. The supernatant was aspirated and filtered through 0.45 μm filter membrane to get the refined wood vinegar, which was sealed in a brown bottle and stored at low temperature.

## 4. Wood vinegar detection method

Take 10 mL each of the treated wood vinegar samples , add 10 mL ethyl acetate three times, shake fully and evenly, and let it stand for extraction for 2 h. After stratification, the upper organic phase was separated and combined (organic phase combination after three times extraction), 1 mL was transferred to Agilent brown sample bottle, and the sample was analyzed by gas chromatogram-mass spectrometer (GC-MS).
