# Supplementary material for: Mechanistic Investigation of the Pyrolysis Temperature of Reed Wood Vinegar for Maximising the Antibacterial Activity of Escherichia coli and Its Inhibitory Activity
Source: Biology (Basel). 2024 Nov 8;13(11):912. doi: 10.3390/biology13110912 (PMC11592125; doi:10.3390/biology13110912)
Supplement: Supplementary file 1 [file biology-13-00912-s001.zip › Supplementary Figure.pdf]

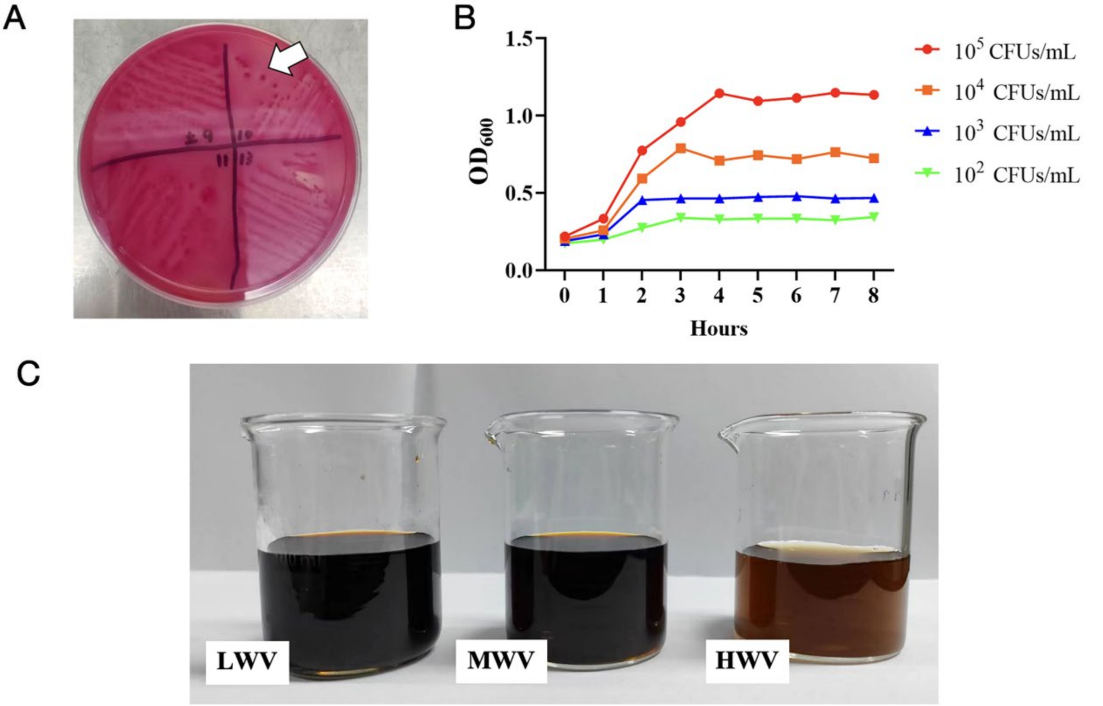

Figure S1. Isolation and culture of *E. coli* and MIC determination of wood vinegar. (A) Isolation of *E. coli* from soil by MacConkey's medium, with red *E. coli* colonies indicated by arrows. (B) 1×10^2, 1×10^3, 1×10^4 and 1×10^5 CFUs/mL *E. coli* were inoculated in LB medium for 8h and the OD_{600} per hour versus time curves were counted. (C) Wood vinegar at different distillation temperatures was obtained by distillation. Where, LWV represents wood vinegar with dark brown color at 300℃ distillation temperature, MWV shows dark brown color at 500℃ distillation temperature, and light brown color at 700°C distillation temperature is indicated by HWV.

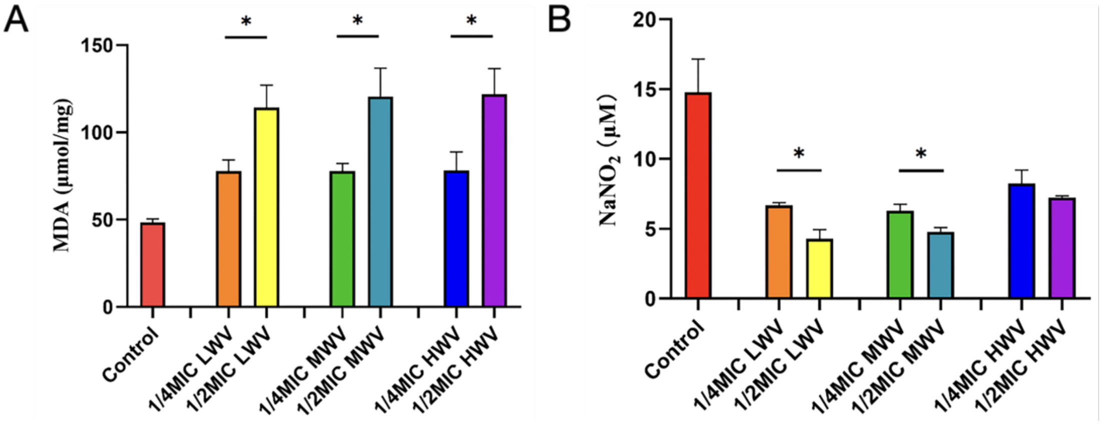

Figure S2. Wood vinegar stimulates oxidative stress in *E. coli*. (A) MDA level, (B) NO level. The differences between the experimental values of the two groups were assessed using the Student's t-test with the following statistical significance: *$P < 0.05$.

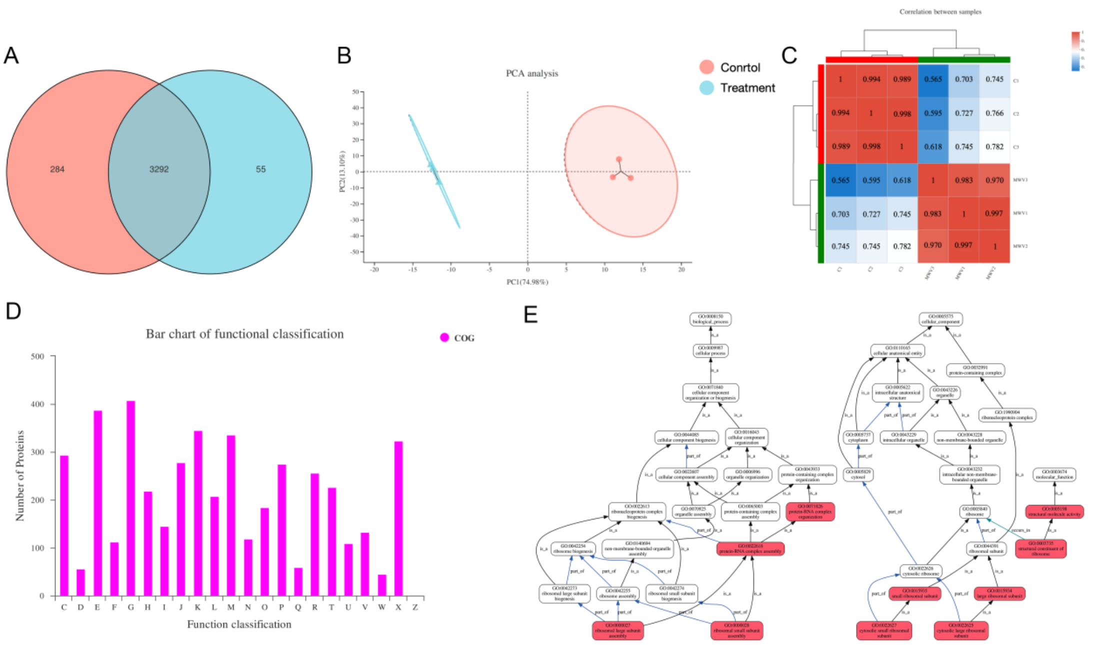

Figure S3. Effect of wood vinegar on the transcriptome of *E. coli*. Transcriptome results for control and MWV groups including (A)Venn diagram, (B)PCA, (C) Relevance analysis, (D) COG classification, (E) GO enrichment analysis.

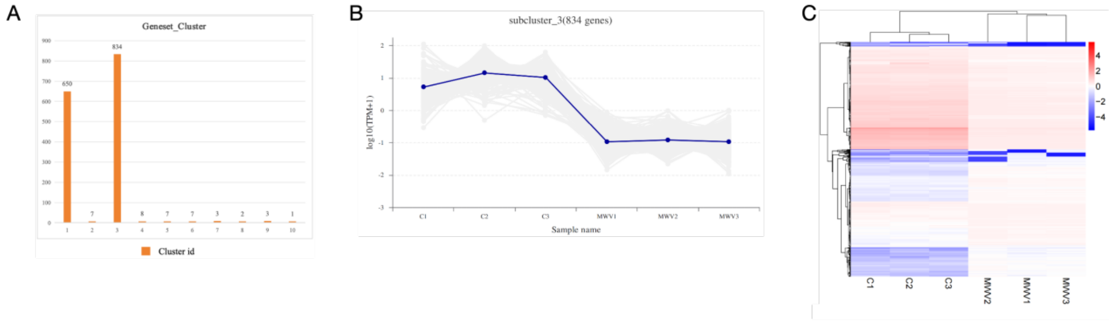

Figure S4. Changes in *E. coli* genes after wood vinegar treatment. (A) Cluster analysis histogram of DEGs in control and MWV transcriptome. (B) Gene trends of 834 DEGs in the third group, the blue line indicates the trend of the mean expression of all genes. (C) Heatmap of clustering of 834 DEGs.
