# Supplementary material for: Mechanistic Investigation of the Pyrolysis Temperature of Reed Wood Vinegar for Maximising the Antibacterial Activity of Escherichia coli and Its Inhibitory Activity
Source: Biology (Basel). 2024 Nov 8;13(11):912. doi: 10.3390/biology13110912 (PMC11592125; doi:10.3390/biology13110912)
Supplement: Supplementary file 1 [file biology-13-00912-s001.zip › Supplementary Table.pdf]

Table S1. ST typing of *E. coli*.

Gene							ST
adk	*fumC*	*icd*	*purA*	*gyrB*	*recA*	*mdh*	
43	41	18	7	15	6	11	101

Table S2. Comprehensive Antibiotic Resistance Database (CARD) annotation.

gene_id	gene_name	description	aro_name	aro_accession	aro_description	drug_class	resistance_mechanism	amr_gene_family	identity	evalue	score
ECs_4501	waaD	lipopolysaccharide 1%2C2-N-acetylglucosamine transferase WaaD	LpsB	ARO:3005051	LpsB is involved in lipopolysaccharide synthesis. It confers intrinsic resistance to colistin and other peptide antibiotics.	peptide antibiotic	reduced permeability to antibiotic	Intrinsic peptide antibiotic resistant Lps	24.1	7.16E-09	53.9
ECs_4698	hdfR	flhDC operon transcriptional repressor	NmcR	ARO:3003665	NmcR is a homolog of the LysR regulator found in Enterobacter cloacae that contribute to the regulation of NmcA beta-lactamase.	carbapenem;cephalosporin;cephamycin;penam	antibiotic inactivation	NmcA beta-lactamase	42.6	7.36E-07	46.6
ECs_3403	hcaR	hca operon transcription	NmcR	ARO:3003665	NmcR is a homolog of the LysR regulator found in Enterobacter	carbapenem;cephalosporin	antibiotic inactivation	NmcA beta-lactama	36.9	9.88E-10	55.5

		al regulator			cloacae that contribute to the regulation of NmcA beta-lactamase.	;cephamycin;penam		se			
ECs_4116	aaeR	transcriptional regulator	NmcR	ARO:3003665	NmcR is a homolog of the LysR regulator found in Enterobacter cloacae that contribute to the regulation of NmcA beta-lactamase.	carbapenem;cephalosporin;cephamycin;penam	antibiotic inactivation	NmcA beta-lactamase	25.9	9.05E-07	46.6
ECs_0565	allS	allD operon transcriptional activator	NmcR	ARO:3003665	NmcR is a homolog of the LysR regulator found in Enterobacter cloacae that contribute to the regulation of NmcA beta-lactamase.	carbapenem;cephalosporin;cephamycin;penam	antibiotic inactivation	NmcA beta-lactamase	32.1	3.76E-07	47.8
ECs_4401	yhjC	LysR family transcriptional regulator	NmcR	ARO:3003665	NmcR is a homolog of the LysR regulator found in Enterobacter cloacae that contribute to the regulation of NmcA beta-lactamase.	carbapenem;cephalosporin;cephamycin;penam	antibiotic inactivation	NmcA beta-lactamase	28	1.44E-11	61.2
ECs_1910	pgrR	murein peptide degradation regulator	NmcR	ARO:3003665	NmcR is a homolog of the LysR regulator found in Enterobacter cloacae that contribute to the regulation of NmcA beta-lactamase.	carbapenem;cephalosporin;cephamycin;penam	antibiotic inactivation	NmcA beta-lactamase	25.8	2.01E-16	75.1
ECs_0080	leuO	DNA-binding transcriptional activator	NmcR	ARO:3003665	NmcR is a homolog of the LysR regulator found in Enterobacter cloacae that contribute to the	carbapenem;cephalosporin;cephamycin;	antibiotic inactivation	NmcA beta-lactamase	37.7	9.14E-08	49.7

		LeuO			regulation of NmcA beta-lactamase.	penam					
ECs_0391	cynR	transcriptional activator of cyn operon	NmcR	ARO:3003665	NmcR is a homolog of the LysR regulator found in Enterobacter cloacae that contribute to the regulation of NmcA beta-lactamase.	carbapenem;cephalosporin;cephamycin;penam	antibiotic inactivation	NmcA beta-lactamase	27.7	1.27E-13	67
ECs_3668	gcvA	glycine cleavage system transcriptional activator	NmcR	ARO:3003665	NmcR is a homolog of the LysR regulator found in Enterobacter cloacae that contribute to the regulation of NmcA beta-lactamase.	carbapenem;cephalosporin;cephamycin;penam	antibiotic inactivation	NmcA beta-lactamase	33.7	1.80E-46	155
ECs_3173	lrhA	transcriptional repressor	NmcR	ARO:3003665	NmcR is a homolog of the LysR regulator found in Enterobacter cloacae that contribute to the regulation of NmcA beta-lactamase.	carbapenem;cephalosporin;cephamycin;penam	antibiotic inactivation	NmcA beta-lactamase	33.6	5.23E-06	44.3
ECs_2508	dmlR	transcriptional activator of dmlA	NmcR	ARO:3003665	NmcR is a homolog of the LysR regulator found in Enterobacter cloacae that contribute to the regulation of NmcA beta-lactamase.	carbapenem;cephalosporin;cephamycin;penam	antibiotic inactivation	NmcA beta-lactamase	27.4	1.39E-13	67
ECs_0333	ycaN	transcriptional regulator	NmcR	ARO:3003665	NmcR is a homolog of the LysR regulator found in Enterobacter cloacae that contribute to the regulation of NmcA	carbapenem;cephalosporin;cephamycin;penam	antibiotic inactivation	NmcA beta-lactamase	26.9	1.72E-11	60.8

					beta-lactamase.						
ECs_2301	ynfL	LysR family transcriptional regulator	NmcR	ARO:3003665	NmcR is a homolog of the LysR regulator found in Enterobacter cloacae that contribute to the regulation of NmcA beta-lactamase.	carbapenem;cephalosporin;cephamycin;penam	antibiotic inactivation	NmcA beta-lactamase	34	3.24E-09	53.9
ECs_0796	ybhD	transcriptional regulator	NmcR	ARO:3003665	NmcR is a homolog of the LysR regulator found in Enterobacter cloacae that contribute to the regulation of NmcA beta-lactamase.	carbapenem;cephalosporin;cephamycin;penam	antibiotic inactivation	NmcA beta-lactamase	29.7	3.89E-08	50.8
ECs_0305	yagP	LysR family transcriptional regulator	NmcR	ARO:3003665	NmcR is a homolog of the LysR regulator found in Enterobacter cloacae that contribute to the regulation of NmcA beta-lactamase.	carbapenem;cephalosporin;cephamycin;penam	antibiotic inactivation	NmcA beta-lactamase	31.7	6.93E-14	67.8
ECs_3786	argP	transcriptional regulator	NmcR	ARO:3003665	NmcR is a homolog of the LysR regulator found in Enterobacter cloacae that contribute to the regulation of NmcA beta-lactamase.	carbapenem;cephalosporin;cephamycin;penam	antibiotic inactivation	NmcA beta-lactamase	30.1	1.46E-07	48.9
ECs_0204	yafC	LysR family transcriptional regulator	NmcR	ARO:3003665	NmcR is a homolog of the LysR regulator found in Enterobacter cloacae that contribute to the regulation of NmcA beta-lactamase.	carbapenem;cephalosporin;cephamycin;penam	antibiotic inactivation	NmcA beta-lactamase	27.3	5.01E-12	62.4

ECs_4691	rbsA	D-ribose ABC transporter ATPase	oleC	ARO:3003748	oleC is an ABC transporter isolated from Streptomyces antibioticus and is involved in oleandomycin secretion.	macrolide antibiotic	antibiotic efflux	ATP-binding cassette (ABC) antibiotic efflux pump	26.4	1.72E-18	83.6
ECs_2045	ydcT	ABC transporter ATPase	oleC	ARO:3003748	oleC is an ABC transporter isolated from Streptomyces antibioticus and is involved in oleandomycin secretion.	macrolide antibiotic	antibiotic efflux	ATP-binding cassette (ABC) antibiotic efflux pump	27.9	9.57E-22	91.3
ECs_0691	gltL	ATP-binding protein of glutamate/aspartate transport system	oleC	ARO:3003748	oleC is an ABC transporter isolated from Streptomyces antibioticus and is involved in oleandomycin secretion.	macrolide antibiotic	antibiotic efflux	ATP-binding cassette (ABC) antibiotic efflux pump	30.8	3.19E-27	103
ECs_2120	lsrA	autoinducer 2 import ATP-binding protein	oleC	ARO:3003748	oleC is an ABC transporter isolated from Streptomyces antibioticus and is involved in oleandomycin secretion.	macrolide antibiotic	antibiotic efflux	ATP-binding cassette (ABC) antibiotic efflux pump	28.7	9.34E-15	72.4
ECs_1571	potA	spermidine/putrescine ABC transporter ATPase	oleC	ARO:3003748	oleC is an ABC transporter isolated from Streptomyces antibioticus and is involved in oleandomycin secretion.	macrolide antibiotic	antibiotic efflux	ATP-binding cassette (ABC) antibiotic efflux pump	30	1.20E-21	91.7

ECs_3041	mglA	methyl-galactoside ABC transporter ATPase	oleC	ARO:3003748	oleC is an ABC transporter isolated from Streptomyces antibioticus and is involved in oleandomycin secretion.	macrolide antibiotic	antibiotic efflux	ATP-binding cassette (ABC) antibiotic efflux pump	25.5	2.01E-16	77.4
ECs_4302	livG	branched-chain amino acid ABC transporter ATPase	oleC	ARO:3003748	oleC is an ABC transporter isolated from Streptomyces antibioticus and is involved in oleandomycin secretion.	macrolide antibiotic	antibiotic efflux	ATP-binding cassette (ABC) antibiotic efflux pump	29.8	8.11E-23	92.4
ECs_5080	phnK	carbon-phosphorus lyase complex subunit	oleC	ARO:3003748	oleC is an ABC transporter isolated from Streptomyces antibioticus and is involved in oleandomycin secretion.	macrolide antibiotic	antibiotic efflux	ATP-binding cassette (ABC) antibiotic efflux pump	28.2	1.68E-19	83.2
ECs_4080	lptB	lipopolysaccharide export ABC transporter ATPase	oleC	ARO:3003748	oleC is an ABC transporter isolated from Streptomyces antibioticus and is involved in oleandomycin secretion.	macrolide antibiotic	antibiotic efflux	ATP-binding cassette (ABC) antibiotic efflux pump	30.8	1.55E-30	112
ECs_2655	yecC	ABC transporter ATPase	oleC	ARO:3003748	oleC is an ABC transporter isolated from Streptomyces antibioticus and is involved in oleandomycin secretion.	macrolide antibiotic	antibiotic efflux	ATP-binding cassette (ABC) antibiotic efflux pump	31.4	1.04E-29	110
ECs_0887	glnQ	ATP-binding	oleC	ARO:3003748	oleC is an ABC transporter	macrolide	antibiotic	ATP-binding	32.1	7.21E-3	119

		component of glutamine high-affinity transport system			isolated from Streptomyces antibioticus and is involved in oleandomycin secretion.	antibiotic	efflux	cassette (ABC) antibiotic efflux pump		3	
ECs_0413	afuC	ferric transporter subunit	oleC	ARO:3003748	oleC is an ABC transporter isolated from Streptomyces antibioticus and is involved in oleandomycin secretion.	macrolide antibiotic	antibiotic efflux	ATP-binding cassette (ABC) antibiotic efflux pump	29	4.53E-27	106
ECs_3016	yehX	transporter subunit: ATP-binding component of ABC superfamily protein	oleC	ARO:3003748	oleC is an ABC transporter isolated from Streptomyces antibioticus and is involved in oleandomycin secretion.	macrolide antibiotic	antibiotic efflux	ATP-binding cassette (ABC) antibiotic efflux pump	31.6	2.63E-24	97.8
ECs_0872	ybhF	ABC transporter ATPase	oleC	ARO:3003748	oleC is an ABC transporter isolated from Streptomyces antibioticus and is involved in oleandomycin secretion.	macrolide antibiotic	antibiotic efflux	ATP-binding cassette (ABC) antibiotic efflux pump	37.1	1.86E-41	149
ECs_4387	ECs_4387	ATP-binding component of hemin transport	oleC	ARO:3003748	oleC is an ABC transporter isolated from Streptomyces antibioticus and is involved in oleandomycin secretion.	macrolide antibiotic	antibiotic efflux	ATP-binding cassette (ABC) antibiotic	24.4	8.44E-12	61.2

		system						efflux pump			
ECs_5079	phnL	ribophosphonate triphosphate synthase subunit	oleC	ARO:3003748	oleC is an ABC transporter isolated from Streptomyces antibioticus and is involved in oleandomycin secretion.	macrolide antibiotic	antibiotic efflux	ATP-binding cassette (ABC) antibiotic efflux pump	31.3	1.33E-11	60.1
ECs_0131	yadG	ABC transporter ATPase	oleC	ARO:3003748	oleC is an ABC transporter isolated from Streptomyces antibioticus and is involved in oleandomycin secretion.	macrolide antibiotic	antibiotic efflux	ATP-binding cassette (ABC) antibiotic efflux pump	31.1	5.10E-43	147
ECs_0627	fepC	ferrienterobactin ABC transporter ATPase	oleC	ARO:3003748	oleC is an ABC transporter isolated from Streptomyces antibioticus and is involved in oleandomycin secretion.	macrolide antibiotic	antibiotic efflux	ATP-binding cassette (ABC) antibiotic efflux pump	28.2	4.99E-20	85.1
ECs_1697	ECs_1697	ferric enterobactin transport ATP-binding protein	oleC	ARO:3003748	oleC is an ABC transporter isolated from Streptomyces antibioticus and is involved in oleandomycin secretion.	macrolide antibiotic	antibiotic efflux	ATP-binding cassette (ABC) antibiotic efflux pump	27.1	6.01E-16	73.2
ECs_2568	znuC	zinc ABC transporter ATPase	oleC	ARO:3003748	oleC is an ABC transporter isolated from Streptomyces antibioticus and is involved in oleandomycin secretion.	macrolide antibiotic	antibiotic efflux	ATP-binding cassette (ABC) antibiotic efflux pump	35.6	3.56E-21	87.8

ECs_0935	potG	putrescine ABC transporter ATPase	oleC	ARO:3003748	oleC is an ABC transporter isolated from Streptomyces antibioticus and is involved in oleandomycin secretion.	macrolide antibiotic	antibiotic efflux	ATP-binding cassette (ABC) antibiotic efflux pump	32	5.30E-27	106
ECs_2087	ddpF	D%2CD-dipeptide ABC transporter ATPase	oleC	ARO:3003748	oleC is an ABC transporter isolated from Streptomyces antibioticus and is involved in oleandomycin secretion.	macrolide antibiotic	antibiotic efflux	ATP-binding cassette (ABC) antibiotic efflux pump	26.2	1.01E-17	79.3
ECs_2088	ddpD	D%2CD-dipeptide ABC transporter ATPase	oleC	ARO:3003748	oleC is an ABC transporter isolated from Streptomyces antibioticus and is involved in oleandomycin secretion.	macrolide antibiotic	antibiotic efflux	ATP-binding cassette (ABC) antibiotic efflux pump	30	9.66E-18	79.7
ECs_5206	ytfR	sugar ABC transporter ATPase	oleC	ARO:3003748	oleC is an ABC transporter isolated from Streptomyces antibioticus and is involved in oleandomycin secretion.	macrolide antibiotic	antibiotic efflux	ATP-binding cassette (ABC) antibiotic efflux pump	29.5	9.50E-22	93.2
ECs_4301	livF	branched-chain amino acid ABC transporter ATPase	oleC	ARO:3003748	oleC is an ABC transporter isolated from Streptomyces antibioticus and is involved in oleandomycin secretion.	macrolide antibiotic	antibiotic efflux	ATP-binding cassette (ABC) antibiotic efflux pump	29.3	6.84E-21	86.7
ECs_0558	ybbA	ABC	oleC	ARO:3003748	oleC is an ABC transporter	macrolide	antibiotic	ATP-binding	30	1.29E-1	80.1

		transporter ATPase			isolated from Streptomyces antibioticus and is involved in oleandomycin secretion.	antibiotic	efflux	cassette (ABC) antibiotic efflux pump		8	
ECs_0947	artP	arginine ABC transporter ATPase	oleC	ARO:3003748	oleC is an ABC transporter isolated from Streptomyces antibioticus and is involved in oleandomycin secretion.	macrolide antibiotic	antibiotic efflux	ATP-binding cassette (ABC) antibiotic efflux pump	32.9	4.34E-30	111
ECs_3413	yphE	sugar ABC transporter ATPase	oleC	ARO:3003748	oleC is an ABC transporter isolated from Streptomyces antibioticus and is involved in oleandomycin secretion.	macrolide antibiotic	antibiotic efflux	ATP-binding cassette (ABC) antibiotic efflux pump	31	1.16E-15	75.1
ECs_0201	metN	DL-methionine transporter subunit	oleC	ARO:3003748	oleC is an ABC transporter isolated from Streptomyces antibioticus and is involved in oleandomycin secretion.	macrolide antibiotic	antibiotic efflux	ATP-binding cassette (ABC) antibiotic efflux pump	29	5.56E-22	92
ECs_4359	rbbA	ribosome-associated ATPase: ATP-binding protein/ATP-binding membrane	oleC	ARO:3003748	oleC is an ABC transporter isolated from Streptomyces antibioticus and is involved in oleandomycin secretion.	macrolide antibiotic	antibiotic efflux	ATP-binding cassette (ABC) antibiotic efflux pump	35.2	8.66E-35	132

		protein									
ECs_0502	mdlA	multidrug ABC transporter ATPase	tetA(60)	ARO:3004035	tetA(60) is a subunit of tetAB(60), an ABC transporter that confers resistance to tetracycline and tigercycline identified by screening a human saliva metagenomic library in Escherichia coli, which is required for resistance.	tetracycline antibiotic	antibiotic efflux	ATP-binding cassette (ABC) antibiotic efflux pump	35	5.62E-105	325
ECs_3100	yojI	microcin J25 efflux ABC transporter permease/ATPase	tetA(60)	ARO:3004035	tetA(60) is a subunit of tetAB(60), an ABC transporter that confers resistance to tetracycline and tigercycline identified by screening a human saliva metagenomic library in Escherichia coli, which is required for resistance.	tetracycline antibiotic	antibiotic efflux	ATP-binding cassette (ABC) antibiotic efflux pump	26.3	7.01E-14	71.2
ECs_0543	lapB	ABC transporter ATP-binding protein	tetA(60)	ARO:3004035	tetA(60) is a subunit of tetAB(60), an ABC transporter that confers resistance to tetracycline and tigercycline identified by screening a human saliva metagenomic library in Escherichia coli, which is required for resistance.	tetracycline antibiotic	antibiotic efflux	ATP-binding cassette (ABC) antibiotic efflux pump	28.1	9.99E-33	130
ECs_0997	msbA	lipid ABC	msbA	ARO:3003950	MsbA is a multidrug resistance	nitroimidazo	antibiotic	ATP-binding	100	0	1092

		transporter permease/ATPase			transporter homolog from E. coli and belongs to a superfamily of transporters that contain an adenosine triphosphate (ATP) binding cassette (ABC) which is also called a nucleotide-binding domain (NBD). MsbA is a member of the MDR-ABC transporter group by sequence homology. MsbA transports lipid A, a major component of the bacterial outer cell membrane, and is the only bacterial ABC transporter that is essential for cell viability.	le antibiotic	efflux	cassette (ABC) antibiotic efflux pump			
ECs_4074	mlaF	organic solvent ABC transporter ATP-binding protein	msbA	ARO:3003950	MsbA is a multidrug resistance transporter homolog from E. coli and belongs to a superfamily of transporters that contain an adenosine triphosphate (ATP) binding cassette (ABC) which is also called a nucleotide-binding domain (NBD). MsbA is a member of the MDR-ABC transporter group by sequence homology. MsbA transports lipid A, a major component of the bacterial outer	nitroimidazole antibiotic	antibiotic efflux	ATP-binding cassette (ABC) antibiotic efflux pump	29	3.53E-16	75.1

					cell membrane, and is the only bacterial ABC transporter that is essential for cell viability.						
ECs_3190	hisP	histidine ABC transporter ATP-binding protein	msbA	ARO:3003950	MsbA is a multidrug resistance transporter homolog from E. coli and belongs to a superfamily of transporters that contain an adenosine triphosphate (ATP) binding cassette (ABC) which is also called a nucleotide-binding domain (NBD). MsbA is a member of the MDR-ABC transporter group by sequence homology. MsbA transports lipid A, a major component of the bacterial outer cell membrane, and is the only bacterial ABC transporter that is essential for cell viability.	nitroimidazole antibiotic	antibiotic efflux	ATP-binding cassette (ABC) antibiotic efflux pump	29	9.27E-19	82.4
ECs_0503	mdlB	multidrug ABC transporter ATPase	msbA	ARO:3003950	MsbA is a multidrug resistance transporter homolog from E. coli and belongs to a superfamily of transporters that contain an adenosine triphosphate (ATP) binding cassette (ABC) which is also called a nucleotide-binding domain (NBD). MsbA is a member of	nitroimidazole antibiotic	antibiotic efflux	ATP-binding cassette (ABC) antibiotic efflux pump	30	4.67E-72	239

					the MDR-ABC transporter group by sequence homology. MsbA transports lipid A, a major component of the bacterial outer cell membrane, and is the only bacterial ABC transporter that is essential for cell viability.					
ECs_4296	ugpC	sn-glycerol-3-phosphate ABC transporter ATPase	msbA	ARO:3003950	MsbA is a multidrug resistance transporter homolog from E. coli and belongs to a superfamily of transporters that contain an adenosine triphosphate (ATP) binding cassette (ABC) which is also called a nucleotide-binding domain (NBD). MsbA is a member of the MDR-ABC transporter group by sequence homology. MsbA transports lipid A, a major component of the bacterial outer cell membrane, and is the only bacterial ABC transporter that is essential for cell viability.	nitroimidazole antibiotic	antibiotic efflux	ATP-binding cassette (ABC) antibiotic efflux pump	27.7	2.76E-17	80.1
ECs_4144	yhdZ	amino acid ABC transporter ATPase	msbA	ARO:3003950	MsbA is a multidrug resistance transporter homolog from E. coli and belongs to a superfamily of transporters that contain an	nitroimidazole antibiotic	antibiotic efflux	ATP-binding cassette (ABC) antibiotic	31.1	1.25E-22	93.6

					adenosine triphosphate (ATP) binding cassette (ABC) which is also called a nucleotide-binding domain (NBD). MsbA is a member of the MDR-ABC transporter group by sequence homology. MsbA transports lipid A, a major component of the bacterial outer cell membrane, and is the only bacterial ABC transporter that is essential for cell viability.			efflux pump			
ECs_0070	thiQ	thiamine/thiamine pyrophosphate ABC transporter ATPase	msbA	ARO:3003950	MsbA is a multidrug resistance transporter homolog from E. coli and belongs to a superfamily of transporters that contain an adenosine triphosphate (ATP) binding cassette (ABC) which is also called a nucleotide-binding domain (NBD). MsbA is a member of the MDR-ABC transporter group by sequence homology. MsbA transports lipid A, a major component of the bacterial outer cell membrane, and is the only bacterial ABC transporter that is essential for cell viability.	nitroimidazole antibiotic	antibiotic efflux	ATP-binding cassette (ABC) antibiotic efflux pump	34.6	4.15E-23	94.4

ECs_1897	ycjV	sugar transporter subunit	msbA	ARO:3003950	MsbA is a multidrug resistance transporter homolog from E. coli and belongs to a superfamily of transporters that contain an adenosine triphosphate (ATP) binding cassette (ABC) which is also called a nucleotide-binding domain (NBD). MsbA is a member of the MDR-ABC transporter group by sequence homology. MsbA transports lipid A, a major component of the bacterial outer cell membrane, and is the only bacterial ABC transporter that is essential for cell viability.	nitroimidazole antibiotic	antibiotic efflux	ATP-binding cassette (ABC) antibiotic efflux pump	31	3.21E-21	92
ECs_4347	nikE	nickel ABC transporter ATPase	msbA	ARO:3003950	MsbA is a multidrug resistance transporter homolog from E. coli and belongs to a superfamily of transporters that contain an adenosine triphosphate (ATP) binding cassette (ABC) which is also called a nucleotide-binding domain (NBD). MsbA is a member of the MDR-ABC transporter group by sequence homology. MsbA transports lipid A, a major	nitroimidazole antibiotic	antibiotic efflux	ATP-binding cassette (ABC) antibiotic efflux pump	31.2	3.48E-19	84

					component of the bacterial outer cell membrane, and is the only bacterial ABC transporter that is essential for cell viability.						
ECs_4346	nikD	nickel ABC transporter ATPase	msbA	ARO:3003950	MsbA is a multidrug resistance transporter homolog from E. coli and belongs to a superfamily of transporters that contain an adenosine triphosphate (ATP) binding cassette (ABC) which is also called a nucleotide-binding domain (NBD). MsbA is a member of the MDR-ABC transporter group by sequence homology. MsbA transports lipid A, a major component of the bacterial outer cell membrane, and is the only bacterial ABC transporter that is essential for cell viability.	nitroimidazole antibiotic	antibiotic efflux	ATP-binding cassette (ABC) antibiotic efflux pump	27.4	1.31E-14	70.1
gene-hlyB	hlyB	Hemolysin B	msbA	ARO:3003950	MsbA is a multidrug resistance transporter homolog from E. coli and belongs to a superfamily of transporters that contain an adenosine triphosphate (ATP) binding cassette (ABC) which is also called a nucleotide-binding	nitroimidazole antibiotic	antibiotic efflux	ATP-binding cassette (ABC) antibiotic efflux pump	34.7	2.41E-79	261

					domain (NBD). MsbA is a member of the MDR-ABC transporter group by sequence homology. MsbA transports lipid A, a major component of the bacterial outer cell membrane, and is the only bacterial ABC transporter that is essential for cell viability.						
ECs_4450	xylG	D-xylose ABC transporter dual domain ATPase	msbA	ARO:3003950	MsbA is a multidrug resistance transporter homolog from E. coli and belongs to a superfamily of transporters that contain an adenosine triphosphate (ATP) binding cassette (ABC) which is also called a nucleotide-binding domain (NBD). MsbA is a member of the MDR-ABC transporter group by sequence homology. MsbA transports lipid A, a major component of the bacterial outer cell membrane, and is the only bacterial ABC transporter that is essential for cell viability.	nitroimidazole antibiotic	antibiotic efflux	ATP-binding cassette (ABC) antibiotic efflux pump	28.4	7.18E-17	80.5
ECs_1746	oppD	oligopeptide ABC transporter	msbA	ARO:3003950	MsbA is a multidrug resistance transporter homolog from E. coli and belongs to a superfamily of	nitroimidazole antibiotic	antibiotic efflux	ATP-binding cassette (ABC)	29.3	5.78E-14	69.7

		ATPase			transporters that contain an adenosine triphosphate (ATP) binding cassette (ABC) which is also called a nucleotide-binding domain (NBD). MsbA is a member of the MDR-ABC transporter group by sequence homology. MsbA transports lipid A, a major component of the bacterial outer cell membrane, and is the only bacterial ABC transporter that is essential for cell viability.			antibiotic efflux pump			
ECs_1747	oppF	oligopeptide ABC transporter ATPase	msbA	ARO:3003950	MsbA is a multidrug resistance transporter homolog from E. coli and belongs to a superfamily of transporters that contain an adenosine triphosphate (ATP) binding cassette (ABC) which is also called a nucleotide-binding domain (NBD). MsbA is a member of the MDR-ABC transporter group by sequence homology. MsbA transports lipid A, a major component of the bacterial outer cell membrane, and is the only bacterial ABC transporter that is	nitroimidazole antibiotic	antibiotic efflux	ATP-binding cassette (ABC) antibiotic efflux pump	28.9	3.03E-15	73.6

					essential for cell viability.						
ECs_0972	cydD	amino acid ABC transporter permease	msbA	ARO:3003950	MsbA is a multidrug resistance transporter homolog from E. coli and belongs to a superfamily of transporters that contain an adenosine triphosphate (ATP) binding cassette (ABC) which is also called a nucleotide-binding domain (NBD). MsbA is a member of the MDR-ABC transporter group by sequence homology. MsbA transports lipid A, a major component of the bacterial outer cell membrane, and is the only bacterial ABC transporter that is essential for cell viability.	nitroimidazole antibiotic	antibiotic efflux	ATP-binding cassette (ABC) antibiotic efflux pump	26.9	2.21E-41	155
ECs_0971	cydC	glutathione ABC transporter ATP-binding protein	msbA	ARO:3003950	MsbA is a multidrug resistance transporter homolog from E. coli and belongs to a superfamily of transporters that contain an adenosine triphosphate (ATP) binding cassette (ABC) which is also called a nucleotide-binding domain (NBD). MsbA is a member of the MDR-ABC transporter group by sequence homology. MsbA	nitroimidazole antibiotic	antibiotic efflux	ATP-binding cassette (ABC) antibiotic efflux pump	27.7	3.15E-50	179

					transports lipid A, a major component of the bacterial outer cell membrane, and is the only bacterial ABC transporter that is essential for cell viability.						
ECs_3293	cysA	sulfate/thiosulfate transporter subunit	tetA(58)	ARO:3003980	TetA(58) is a Tetracycline efflux pump described in Paenibacillus sp. LC231, a strain of Paenibacillus isolated from Lechuguilla Cave, NM, USA. Described by Pawlowski et al. 2016.	tetracycline antibiotic	antibiotic efflux	major facilitator superfamily (MFS) antibiotic efflux pump	28.6	6.87E-22	92.4
ECs_0965	macB	macrolide ABC transporter permease/ATPase	tetA(58)	ARO:3003980	TetA(58) is a Tetracycline efflux pump described in Paenibacillus sp. LC231, a strain of Paenibacillus isolated from Lechuguilla Cave, NM, USA. Described by Pawlowski et al. 2016.	tetracycline antibiotic	antibiotic efflux	major facilitator superfamily (MFS) antibiotic efflux pump	29.3	1.03E-18	85.1
ECs_5088	phnC	phosphonate ABC transporter ATPase	tetA(58)	ARO:3003980	TetA(58) is a Tetracycline efflux pump described in Paenibacillus sp. LC231, a strain of Paenibacillus isolated from Lechuguilla Cave, NM, USA. Described by Pawlowski et al. 2016.	tetracycline antibiotic	antibiotic efflux	major facilitator superfamily (MFS) antibiotic efflux pump	30	9.33E-25	98.2

ECs_5018	malK	maltose ABC transporter ATPase	tetA(58)	ARO:3003980	TetA(58) is a Tetracycline efflux pump described in Paenibacillus sp. LC231, a strain of Paenibacillus isolated from Lechuguilla Cave, NM, USA. Described by Pawlowski et al. 2016.	tetracycline antibiotic	antibiotic efflux	major facilitator superfamily (MFS) antibiotic efflux pump	30.5	6.83E-24	98.2
ECs_3916	fepC_2	ABC-type iron-siderophore transport system ATP-binding protein	tetA(58)	ARO:3003980	TetA(58) is a Tetracycline efflux pump described in Paenibacillus sp. LC231, a strain of Paenibacillus isolated from Lechuguilla Cave, NM, USA. Described by Pawlowski et al. 2016.	tetracycline antibiotic	antibiotic efflux	major facilitator superfamily (MFS) antibiotic efflux pump	27.8	2.50E-18	80.5
ECs_3540	proV	glycine betaine/proline ABC transporter periplasmic binding protein	tetA(58)	ARO:3003980	TetA(58) is a Tetracycline efflux pump described in Paenibacillus sp. LC231, a strain of Paenibacillus isolated from Lechuguilla Cave, NM, USA. Described by Pawlowski et al. 2016.	tetracycline antibiotic	antibiotic efflux	major facilitator superfamily (MFS) antibiotic efflux pump	30.6	2.18E-28	111
ECs_1868	sapD	antimicrobial peptide ABC transporter ATPase	tetA(58)	ARO:3003980	TetA(58) is a Tetracycline efflux pump described in Paenibacillus sp. LC231, a strain of Paenibacillus isolated from Lechuguilla Cave, NM, USA.	tetracycline antibiotic	antibiotic efflux	major facilitator superfamily (MFS) antibiotic	26	1.53E-10	58.5

					Described by Pawlowski et al. 2016.			efflux pump			
ECs_0788	modF	molybdate ABC transporter ATPase	tetA(58)	ARO:3003980	TetA(58) is a Tetracycline efflux pump described in Paenibacillus sp. LC231, a strain of Paenibacillus isolated from Lechuguilla Cave, NM, USA. Described by Pawlowski et al. 2016.	tetracycline antibiotic	antibiotic efflux	major facilitator superfamily (MFS) antibiotic efflux pump	23.7	5.96E-12	63.9
ECs_1016	ssuB	aliphatic sulfonate ABC transporter ATPase	tetA(58)	ARO:3003980	TetA(58) is a Tetracycline efflux pump described in Paenibacillus sp. LC231, a strain of Paenibacillus isolated from Lechuguilla Cave, NM, USA. Described by Pawlowski et al. 2016.	tetracycline antibiotic	antibiotic efflux	major facilitator superfamily (MFS) antibiotic efflux pump	34.6	1.13E-19	84
ECs_3090	ccmA	heme export ABC transporter ATPase	tetA(58)	ARO:3003980	TetA(58) is a Tetracycline efflux pump described in Paenibacillus sp. LC231, a strain of Paenibacillus isolated from Lechuguilla Cave, NM, USA. Described by Pawlowski et al. 2016.	tetracycline antibiotic	antibiotic efflux	major facilitator superfamily (MFS) antibiotic efflux pump	29.4	1.91E-16	73.6
ECs_2462	ynjD	ABC transporter ATPase	bcrA	ARO:3002987	bcrA is an ABC transporter found in Bacillus licheniformis that confers bacitracin resistance.	peptide antibiotic	antibiotic efflux	ATP-binding cassette (ABC)	28	3.28E-15	70.1

								antibiotic efflux pump			
ECs_4421	dppD	dipeptide/heme ABC transporter ATPase	bcrA	ARO:3002987	bcrA is an ABC transporter found in Bacillus licheniformis that confers bacitracin resistance.	peptide antibiotic	antibiotic efflux	ATP-binding cassette (ABC) antibiotic efflux pump	23.8	6.57E-12	62.4
ECs_1292	ECs_1292	ABC transporter ATP-binding protein	bcrA	ARO:3002987	bcrA is an ABC transporter found in Bacillus licheniformis that confers bacitracin resistance.	peptide antibiotic	antibiotic efflux	ATP-binding cassette (ABC) antibiotic efflux pump	27.8	6.14E-18	78.2
ECs_4312	ftsE	cell division ATP-binding protein	bcrA	ARO:3002987	bcrA is an ABC transporter found in Bacillus licheniformis that confers bacitracin resistance.	peptide antibiotic	antibiotic efflux	ATP-binding cassette (ABC) antibiotic efflux pump	26.4	6.78E-19	80.5
ECs_3072	yejF	microcin C ABC transporter ATPase	bcrA	ARO:3002987	bcrA is an ABC transporter found in Bacillus licheniformis that confers bacitracin resistance.	peptide antibiotic	antibiotic efflux	ATP-binding cassette (ABC) antibiotic efflux pump	30.3	4.98E-18	82
ECs_4661	pstB	phosphate ABC transporter ATPase	bcrA	ARO:3002987	bcrA is an ABC transporter found in Bacillus licheniformis that confers bacitracin resistance.	peptide antibiotic	antibiotic efflux	ATP-binding cassette (ABC) antibiotic	26.9	6.09E-21	87

								efflux pump			
ECs_2608	araG	L-arabinose ABC transporter ATPase	bcrA	ARO:3002987	bcrA is an ABC transporter found in Bacillus licheniformis that confers bacitracin resistance.	peptide antibiotic	antibiotic efflux	ATP-binding cassette (ABC) antibiotic efflux pump	23.9	1.02E-14	72
ECs_1867	sapF	antimicrobial peptide ABC transporter ATPase	bcrA	ARO:3002987	bcrA is an ABC transporter found in Bacillus licheniformis that confers bacitracin resistance.	peptide antibiotic	antibiotic efflux	ATP-binding cassette (ABC) antibiotic efflux pump	29	1.72E-18	80.5
ECs_0155	fhuC	iron(3+)-hydroxamate import ABC transporter ATPase	bcrA	ARO:3002987	bcrA is an ABC transporter found in Bacillus licheniformis that confers bacitracin resistance.	peptide antibiotic	antibiotic efflux	ATP-binding cassette (ABC) antibiotic efflux pump	25	3.84E-17	76.6
ECs_0420	tauB	taurine ABC transporter ATPase	bcrA	ARO:3002987	bcrA is an ABC transporter found in Bacillus licheniformis that confers bacitracin resistance.	peptide antibiotic	antibiotic efflux	ATP-binding cassette (ABC) antibiotic efflux pump	25.4	3.27E-23	93.2
ECs_0793	modC	molybdate ABC transporter ATPase	bcrA	ARO:3002987	bcrA is an ABC transporter found in Bacillus licheniformis that confers bacitracin resistance.	peptide antibiotic	antibiotic efflux	ATP-binding cassette (ABC) antibiotic efflux pump	32.2	1.55E-20	87.8

ECs_4696	hsrA	multidrug or homocysteine efflux system	emrY	ARO:3000254	emrY is a multidrug transport that moves substrates across the inner membrane of the Gram-negative E. coli. It is a homolog of emrB.	tetracycline antibiotic	antibiotic efflux	major facilitator superfamily (MFS) antibiotic efflux pump	31.5	8.72E-45	161
ECs_2885	iceT	transporter	emrY	ARO:3000254	emrY is a multidrug transport that moves substrates across the inner membrane of the Gram-negative E. coli. It is a homolog of emrB.	tetracycline antibiotic	antibiotic efflux	major facilitator superfamily (MFS) antibiotic efflux pump	28.9	6.49E-41	150
ECs_3548	emrY	multidrug efflux system	emrY	ARO:3000254	emrY is a multidrug transport that moves substrates across the inner membrane of the Gram-negative E. coli. It is a homolog of emrB.	tetracycline antibiotic	antibiotic efflux	major facilitator superfamily (MFS) antibiotic efflux pump	62.4	1.50E-223	623
ECs_3246	emrY	multidrug efflux system	emrY	ARO:3000254	emrY is a multidrug transport that moves substrates across the inner membrane of the Gram-negative E. coli. It is a homolog of emrB.	tetracycline antibiotic	antibiotic efflux	major facilitator superfamily (MFS) antibiotic efflux pump	99.2	0	983
ECs_2138	marA	multiple antibiotic	marA	ARO:3000263	In the presence of antibiotic stress, E. coli overexpresses the	carbapenem;cephalosporin	antibiotic efflux;reduce	General Bacterial	100	2.43E-92	259

		resistance transcriptional regulator			global activator protein MarA, which besides inducing MDR efflux pump AcrAB, also down- regulates synthesis of the porin OmpF.	;cephamycin;disinfecting agents and antiseptics;fluoroquinolone antibiotic;glycylcycline;monobactam;penam;penem;phenicol antibiotic;rifamycin antibiotic;tetracycline antibiotic	d permeability to antibiotic	Porin with reduced permeability to beta-lactams;resistance-nodulation-cell division (RND) antibiotic efflux pump			
ECs_5044	soxS	transcriptional regulator	marA	ARO:3000263	In the presence of antibiotic stress, E. coli overexpresses the global activator protein MarA, which besides inducing MDR efflux pump AcrAB, also down- regulates synthesis of the porin OmpF.	carbapenem;cephalosporin;cephamycin;disinfecting agents and antiseptics;fluoroquinolone antibiotic;glycylcycline	antibiotic efflux;reduced permeability to antibiotic	General Bacterial Porin with reduced permeability to beta-lactams;resistance-nodulation-cell division	41.8	9.19E-27	93.2

						;monobactam;penam;penem;phenicol antibiotic;rifamycin antibiotic;tetracycline antibiotic		(RND) antibiotic efflux pump			
ECs_4833	rhaR	transcriptional activator RhaR	marA	ARO:3000263	In the presence of antibiotic stress, E. coli overexpresses the global activator protein MarA, which besides inducing MDR efflux pump AcrAB, also down- regulates synthesis of the porin OmpF.	carbapenem;cephalosporin;cephamycin;disinfecting agents and antiseptics;fluoroquinolone antibiotic;glycylcycline;monobactam;penam;penem;phenicol antibiotic;rifamycin antibiotic;tetracycline antibiotic	antibiotic efflux;reduced permeability to antibiotic	General Bacterial Porin with reduced permeability to beta-lactams;resistance-nodulation-cell division (RND) antibiotic efflux pump	25.8	7.26E-06	42

ECs_3262	ypdC	DNA-binding protein	marA	ARO:3000263	In the presence of antibiotic stress, E. coli overexpresses the global activator protein MarA, which besides inducing MDR efflux pump AcrAB, also down- regulates synthesis of the porin OmpF.	carbapenem;cephalosporin;cephamycin;disinfecting agents and antiseptics;fluoroquinolone antibiotic;glycylcycline;monobactam;penam;penem;phenicol antibiotic;rifamycin antibiotic;tetracycline antibiotic	antibiotic efflux;reduced permeability to antibiotic	General Bacterial Porin with reduced permeability to beta-lactams;resistance-nodulation-cell division (RND) antibiotic efflux pump	28.1	1.59E-10	55.1
ECs_5354	rob	transcriptional regulator	marA	ARO:3000263	In the presence of antibiotic stress, E. coli overexpresses the global activator protein MarA, which besides inducing MDR efflux pump AcrAB, also down- regulates synthesis of the porin OmpF.	carbapenem;cephalosporin;cephamycin;disinfecting agents and antiseptics;fluoroquinolone	antibiotic efflux;reduced permeability to antibiotic	General Bacterial Porin with reduced permeability to beta-lactams;resistance-	50.5	5.41E-30	107

						antibiotic;glycylcycline;monobactam;penam;penem;phenicol antibiotic;rifamycin antibiotic;tetracycline antibiotic		nodulation-cell division (RND) antibiotic efflux pump			
ECs_0337	ykgA	transcriptional regulator	marA	ARO:3000263	In the presence of antibiotic stress, E. coli overexpresses the global activator protein MarA, which besides inducing MDR efflux pump AcrAB, also down- regulates synthesis of the porin OmpF.	carbapenem;cephalosporin;cephamycin;disinfecting agents and antiseptics;fluoroquinolone antibiotic;glycylcycline;monobactam;penam;penem;phenicol antibiotic;rifamycin antibiotic;t	antibiotic efflux;reduced permeability to antibiotic	General Bacterial Porin with reduced permeability to beta-lactams;resistance-nodulation-cell division (RND) antibiotic efflux pump	36.7	2.23E-18	76.6

						etracycline antibiotic					
ECs_2135	ydeA	arabinose efflux transporter	cmlv	ARO:3002700	cmlv is a chromosome-encoded chloramphenicol phoshotransferase that is found in Streptomyces venezuelae.	phenicol antibiotic	antibiotic inactivation	chloramphenicol phosphotransferase	27.4	5.77E-29	114
ECs_0446	araJ	L-arabinose-inducible transporter	cmlv	ARO:3002700	cmlv is a chromosome-encoded chloramphenicol phoshotransferase that is found in Streptomyces venezuelae.	phenicol antibiotic	antibiotic inactivation	chloramphenicol phosphotransferase	27	1.28E-37	139
ECs_2366	ydhP	MFS transporter	cmlv	ARO:3002700	cmlv is a chromosome-encoded chloramphenicol phoshotransferase that is found in Streptomyces venezuelae.	phenicol antibiotic	antibiotic inactivation	chloramphenicol phosphotransferase	35.4	8.20E-63	205
ECs_4596	nepI	transporter	cmlv	ARO:3002700	cmlv is a chromosome-encoded chloramphenicol phoshotransferase that is found in Streptomyces venezuelae.	phenicol antibiotic	antibiotic inactivation	chloramphenicol phosphotransferase	23	4.90E-21	91.7
ECs_0311	ECs_0311	transport protein	cmlv	ARO:3002700	cmlv is a chromosome-encoded chloramphenicol phoshotransferase that is found in Streptomyces venezuelae.	phenicol antibiotic	antibiotic inactivation	chloramphenicol phosphotransferase	25.5	7.06E-19	84.7
ECs_4097	nanT	sialic acid transporter	Rhodococcus fascians cmr	ARO:3002701	cmr is a plasmid-encoded chloramphenicol exporter that is found in Rhodococcus fascians and Corynebacterium glutamicum.	phenicol antibiotic	antibiotic efflux	major facilitator superfamily (MFS)	23.8	6.68E-08	51.6

								antibiotic efflux pump			
ECs_4190	tufB	translation elongation factor EF-Tu 1	tet(W/32/O)	ARO:3007124	Tet(W/32/O) is a mosaic tetracycline resistance gene and ribosomal protection protein.	tetracycline antibiotic	antibiotic target protection	tetracycline-resistant ribosomal protection protein	27.3	1.32E-20	90.9
ECs_3435	lepA	back-translocating elongation factor EF4	tet(W/32/O)	ARO:3007124	Tet(W/32/O) is a mosaic tetracycline resistance gene and ribosomal protection protein.	tetracycline antibiotic	antibiotic target protection	tetracycline-resistant ribosomal protection protein	24.6	7.48E-26	109
ECs_4903	tufB	translation elongation factor EF-Tu 1	tet(W/32/O)	ARO:3007124	Tet(W/32/O) is a mosaic tetracycline resistance gene and ribosomal protection protein.	tetracycline antibiotic	antibiotic target protection	tetracycline-resistant ribosomal protection protein	27.3	1.32E-20	90.9
ECs_4191	fusA	protein chain elongation factor EF-G	tet(O/W)	ARO:3007121	Tet(O/W) is a mosaic tetracycline resistance gene and ribosomal protection protein.	tetracycline antibiotic	antibiotic target protection	tetracycline-resistant ribosomal protection protein	27.4	2.57E-64	222
ECs_4467	selB	selenocysteinyl-tRNA-specific translation	tet(O/W)	ARO:3007121	Tet(O/W) is a mosaic tetracycline resistance gene and ribosomal protection protein.	tetracycline antibiotic	antibiotic target protection	tetracycline-resistant ribosomal protection	32.1	3.52E-09	56.6

		factor						protein		
ECs_4049	infB	translation initiation factor IF-2	tet(O/W)	ARO:3007121	Tet(O/W) is a mosaic tetracycline resistance gene and ribosomal protection protein.	tetracycline antibiotic	antibiotic target protection	tetracycline-resistant ribosomal protection protein	30.2	3.45E-09	57.4
ECs_3605	cysN	sulfate adenylyltransferase subunit 1	tet(O/W)	ARO:3007121	Tet(O/W) is a mosaic tetracycline resistance gene and ribosomal protection protein.	tetracycline antibiotic	antibiotic target protection	tetracycline-resistant ribosomal protection protein	33.8	3.03E-09	56.2
ECs_4926	zraS	two-component system sensor histidine kinase ZraS	evgS	ARO:3000833	EvgS is a sensor protein that phosphorylates the regulatory protein EvgA. evgS corresponds to 1 locus in Pseudomonas aeruginosa PAO1 and 1 locus in Pseudomonas aeruginosa LESB58.	fluoroquinolone antibiotic;macrolide antibiotic;penam;tetracycline antibiotic	antibiotic efflux	major facilitator superfamily (MFS) antibiotic efflux pump;resistance-nodulation-cell division (RND) antibiotic efflux pump	26.6	4.46E-17	81.3
ECs_3646	barA	hybrid sensory	evgS	ARO:3000833	EvgS is a sensor protein that phosphorylates the regulatory	fluoroquinolone	antibiotic efflux	major facilitator	23.2	7.62E-38	150

		histidine kinase BarA			protein EvgA. evgS corresponds to 1 locus in Pseudomonas aeruginosa PAO1 and 1 locus in Pseudomonas aeruginosa LESB58.	antibiotic;macrolide antibiotic;penam;tetracycline antibiotic		superfamily (MFS) antibiotic efflux pump;resistance-nodulation-cell division (RND) antibiotic efflux pump			
ECs_2592	cheY	chemotaxis regulator transmitting signal to flagellar motor component	evgS	ARO:3000833	EvgS is a sensor protein that phosphorylates the regulatory protein EvgA. evgS corresponds to 1 locus in Pseudomonas aeruginosa PAO1 and 1 locus in Pseudomonas aeruginosa LESB58.	fluoroquinolone antibiotic;macrolide antibiotic;penam;tetracycline antibiotic	antibiotic efflux	major facilitator superfamily (MFS) antibiotic efflux pump;resistance-nodulation-cell division (RND) antibiotic efflux pump	35.2	1.74E-16	71.6
ECs_4089	arcB	aerobic respiration	evgS	ARO:3000833	EvgS is a sensor protein that phosphorylates the regulatory	fluoroquinolone	antibiotic efflux	major facilitator	26.1	8.66E-50	186

		control sensor histidine protein kinase			protein EvgA. evgS corresponds to 1 locus in Pseudomonas aeruginosa PAO1 and 1 locus in Pseudomonas aeruginosa LESB58.	antibiotic;macrolide antibiotic;penam;tetracycline antibiotic		superfamily (MFS) antibiotic efflux pump;resistance-nodulation-cell division (RND) antibiotic efflux pump			
ECs_5074	ECs_5074	histidine protein kinase	evgS	ARO:3000833	EvgS is a sensor protein that phosphorylates the regulatory protein EvgA. evgS corresponds to 1 locus in Pseudomonas aeruginosa PAO1 and 1 locus in Pseudomonas aeruginosa LESB58.	fluoroquinolone antibiotic;macrolide antibiotic;penam;tetracycline antibiotic	antibiotic efflux	major facilitator superfamily (MFS) antibiotic efflux pump;resistance-nodulation-cell division (RND) antibiotic efflux pump	26.3	1.80E-15	77.8
ECs_3107	rcsC	hybrid sensory	evgS	ARO:3000833	EvgS is a sensor protein that phosphorylates the regulatory	fluoroquinolone	antibiotic efflux	major facilitator	30.8	1.93E-57	212

		kinase in two-component regulatory system with RcsB and YojN			protein EvgA. evgS corresponds to 1 locus in Pseudomonas aeruginosa PAO1 and 1 locus in Pseudomonas aeruginosa LESB58.	antibiotic;macrolide antibiotic;penam;tetracycline antibiotic		superfamily (MFS) antibiotic efflux pump;resistance-nodulation-cell division (RND) antibiotic efflux pump			
ECs_3249	evgS	hybrid sensory histidine kinase in two-component regulatory system with EvgA	evgS	ARO:3000833	EvgS is a sensor protein that phosphorylates the regulatory protein EvgA. evgS corresponds to 1 locus in Pseudomonas aeruginosa PAO1 and 1 locus in Pseudomonas aeruginosa LESB58.	fluoroquinolone antibiotic;macrolide antibiotic;penam;tetracycline antibiotic	antibiotic efflux	major facilitator superfamily (MFS) antibiotic efflux pump;resistance-nodulation-cell division (RND) antibiotic efflux pump	97.8	0	2323
ECs_1148	torS	hybrid sensory	evgS	ARO:3000833	EvgS is a sensor protein that phosphorylates the regulatory	fluoroquinolone	antibiotic efflux	major facilitator	28.1	6.51E-37	147

		histidine kinase in two-component regulatory system with TorR			protein EvgA. evgS corresponds to 1 locus in Pseudomonas aeruginosa PAO1 and 1 locus in Pseudomonas aeruginosa LESB58.	antibiotic;macrolide antibiotic;penam;tetracycline antibiotic		superfamily (MFS) antibiotic efflux pump;resistance-nodulation-cell division (RND) antibiotic efflux pump			
ECs_5107	dcuS	two-component system sensor histidine kinase DcuS	evgS	ARO:3000833	EvgS is a sensor protein that phosphorylates the regulatory protein EvgA. evgS corresponds to 1 locus in Pseudomonas aeruginosa PAO1 and 1 locus in Pseudomonas aeruginosa LESB58.	fluoroquinolone antibiotic;macrolide antibiotic;penam;tetracycline antibiotic	antibiotic efflux	major facilitator superfamily (MFS) antibiotic efflux pump;resistance-nodulation-cell division (RND) antibiotic efflux pump	28.8	4.23E-08	53.1
ECs_3422	glrK	two-component system sensor	evgS	ARO:3000833	EvgS is a sensor protein that phosphorylates the regulatory	fluoroquinolone	antibiotic efflux	major facilitator	30.3	4.70E-18	84.3

		histidine kinase GlrK			protein EvgA. evgS corresponds to 1 locus in Pseudomonas aeruginosa PAO1 and 1 locus in Pseudomonas aeruginosa LESB58.	antibiotic;macrolide antibiotic;penam;tetracycline antibiotic		superfamily (MFS) antibiotic efflux pump;resistance-nodulation-cell division (RND) antibiotic efflux pump			
ECs_1601	phoQ	two-component system sensor histidine kinase PhoQ	basS	ARO:3003583	Histidine protein kinase sensor Lipid A modification gene; part of a two-component system involved in polymyxin resistance that senses high extracellular Fe(2+).	peptide antibiotic	antibiotic efflux;antibiotic target alteration	pmr phosphoethanolamine transferase	28.1	8.55E-18	82.8
ECs_2706	yedV	two-component system sensor histidine kinase YedV	basS	ARO:3003583	Histidine protein kinase sensor Lipid A modification gene; part of a two-component system involved in polymyxin resistance that senses high extracellular Fe(2+).	peptide antibiotic	antibiotic efflux;antibiotic target alteration	pmr phosphoethanolamine transferase	26	9.13E-25	103
ECs_5094	basS	two-component system sensor histidine	basS	ARO:3003583	Histidine protein kinase sensor Lipid A modification gene; part of a two-component system	peptide antibiotic	antibiotic efflux;antibiotic target	pmr phosphoethanolamine	33.8	1.32E-31	121

		kinase BasS			involved in polymyxin resistance that senses high extracellular Fe(2+).		alteration	transferase			
ECs_0608	cusS	copper-sensing histidine kinase in two-component regulatory system with CusR	basS	ARO:3003583	Histidine protein kinase sensor Lipid A modification gene; part of a two-component system involved in polymyxin resistance that senses high extracellular Fe(2+).	peptide antibiotic	antibiotic efflux;antibiotic target alteration	pmr phosphoethanolamine transferase	25.1	6.03E-36	136
ECs_4791	glnL	two-component system sensor histidine kinase GlnL	basS	ARO:3003583	Histidine protein kinase sensor Lipid A modification gene; part of a two-component system involved in polymyxin resistance that senses high extracellular Fe(2+).	peptide antibiotic	antibiotic efflux;antibiotic target alteration	pmr phosphoethanolamine transferase	27.9	7.66E-13	66.2
ECs_5357	creC	two-component system sensor histidine kinase CreC	basS	ARO:3003583	Histidine protein kinase sensor Lipid A modification gene; part of a two-component system involved in polymyxin resistance that senses high extracellular Fe(2+).	peptide antibiotic	antibiotic efflux;antibiotic target alteration	pmr phosphoethanolamine transferase	28.4	4.15E-22	95.9
ECs_4246	envZ	two-component system sensor histidine kinase EnvZ	basS	ARO:3003583	Histidine protein kinase sensor Lipid A modification gene; part of a two-component system involved in polymyxin resistance	peptide antibiotic	antibiotic efflux;antibiotic target alteration	pmr phosphoethanolamine transferase	27	2.00E-25	105

					that senses high extracellular Fe(2+).						
ECs_3908	qseC	quorum sensing sensory histidine kinase in two-component regulatory system with QseB	basS	ARO:3003583	Histidine protein kinase sensor Lipid A modification gene; part of a two-component system involved in polymyxin resistance that senses high extracellular Fe(2+).	peptide antibiotic	antibiotic efflux;antibiotic target alteration	pmr phosphoethanolamine transferase	31.1	4.11E-47	166
ECs_4837	cpxA	two-component system sensor histidine kinase CpxA	cpxA	ARO:3000830	CpxA is a membrane-localized sensor kinase that is activated by envelope stress. It starts a kinase cascade that activates CpxR, which promotes efflux complex expression.	aminocoumarin antibiotic;aminoglycoside antibiotic	antibiotic efflux	resistance-nodulation-cell division (RND) antibiotic efflux pump	100	0	887
ECs_0450	phoR	two-component system sensor histidine kinase PhoR	cpxA	ARO:3000830	CpxA is a membrane-localized sensor kinase that is activated by envelope stress. It starts a kinase cascade that activates CpxR, which promotes efflux complex expression.	aminocoumarin antibiotic;aminoglycoside antibiotic	antibiotic efflux	resistance-nodulation-cell division (RND) antibiotic efflux pump	29.9	1.56E-24	102
ECs_2886	baeS	two-component system sensor histidine	cpxA	ARO:3000830	CpxA is a membrane-localized sensor kinase that is activated by envelope stress. It starts a	aminocoumarin antibiotic;a	antibiotic efflux	resistance-nodulation-cell division	28	4.76E-28	113

		kinase BaeS			kinase cascade that activates CpxR, which promotes efflux complex expression.	minoglycoside antibiotic		(RND) antibiotic efflux pump			
ECs_2315	rstB	two-component system sensor histidine kinase RstB	cpxA	ARO:3000830	CpxA is a membrane-localized sensor kinase that is activated by envelope stress. It starts a kinase cascade that activates CpxR, which promotes efflux complex expression.	aminocoumarin antibiotic;aminoglycoside antibiotic	antibiotic efflux	resistance-nodulation-cell division (RND) antibiotic efflux pump	32.8	1.20E-38	142
ECs_0723	kdpD	sensory histidine kinase in two-component regulatory system	ParS	ARO:3005067	ParS is the sensor component of the two-component ParRS system. Alongside its counterpart ParR, it confers resistance to polycationic antibiotics though regulation of efflux pumps and porins.	aminoglycoside antibiotic;carbapenem;cephalosporin;cephamycin;disinfecting agents and antiseptics;fluoroquinolone antibiotic;macrolide antibiotic;monobactam;penam;penem;phenicol	antibiotic efflux;reduced permeability to antibiotic	Outer Membrane Porin (Opr);resistance-nodulation-cell division (RND) antibiotic efflux pump	32	6.45E-22	96.7

						antibiotic;tetracycline antibiotic					
ECs_0658	citA	sensory histidine kinase in two-component regulatory system with CitB	ParS	ARO:3005067	ParS is the sensor component of the two-component ParRS system. Alongside its counterpart ParR, it confers resistance to polycationic antibiotics though regulation of efflux pumps and porins.	aminoglycoside antibiotic;carbapenem;cephalosporin;cephamycin;disinfecting agents and antiseptics;fluoroquinolone antibiotic;macrolide antibiotic;monobactam;penam;penem;phenicol antibiotic;tetracycline antibiotic	antibiotic efflux;reduced permeability to antibiotic	Outer Membrane Porin (Opr);resistance-nodulation-cell division (RND) antibiotic efflux pump	24.6	5.62E-06	45.8
ECs_2389	sufC	SufBCD Fe-S cluster assembly	novA	ARO:3002522	A type III ABC transporter, identified on the novobiocin biosynthetic gene cluster,	aminocoumarin antibiotic	antibiotic efflux	ATP-binding cassette (ABC)	29.9	1.33E-16	75.9

		scaffold protein			involved in the transport and resistance of novobiocin.			antibiotic efflux pump			
ECs_5073	ECs_5073	ATP-binding component of sugar ABC transporter	novA	ARO:3002522	A type III ABC transporter, identified on the novobiocin biosynthetic gene cluster, involved in the transport and resistance of novobiocin.	aminocoumarin antibiotic	antibiotic efflux	ATP-binding cassette (ABC) antibiotic efflux pump	29.8	7.33E-17	80.5
ECs_0553	fetA	iron export ABC transporter ATPase	patB	ARO:3000025	PatB is an ABC transporter of Streptococcus pneumoniae that interacts with PatA to confer fluoroquinolone resistance..	fluoroquinolone antibiotic	antibiotic efflux	ATP-binding cassette (ABC) antibiotic efflux pump	27.4	6.15E-19	82
ECs_4420	dppF	dipeptide/heme ABC transporter ATPase	tetB(60)	ARO:3004036	tetB(60) is a subunit of tetAB(60), an ABC transporter that confers resistance to tetracycline and tigercycline identified by screening a human saliva metagenomic library in Escherichia coli, which is required for resistance.	tetracycline antibiotic	antibiotic efflux	ATP-binding cassette (ABC) antibiotic efflux pump	29.3	8.09E-19	84.3
ECs_2101	yddA	multidrug ABC transporter permease/ATPase	tetB(60)	ARO:3004036	tetB(60) is a subunit of tetAB(60), an ABC transporter that confers resistance to tetracycline and tigercycline identified by screening a human saliva metagenomic library in	tetracycline antibiotic	antibiotic efflux	ATP-binding cassette (ABC) antibiotic efflux pump	26.8	4.36E-11	62.4

					Escherichia coli, which is required for resistance.						
ECs_3802	ECs_3802	ABC transporter ATP-binding protein	tetB(60)	ARO:3004036	tetB(60) is a subunit of tetAB(60), an ABC transporter that confers resistance to tetracycline and tigercycline identified by screening a human saliva metagenomic library in Escherichia coli, which is required for resistance.	tetracycline antibiotic	antibiotic efflux	ATP-binding cassette (ABC) antibiotic efflux pump	27.8	2.49E-07	47.8
ECs_0908	gsiA	glutathione ABC transporter ATPase	tetB(60)	ARO:3004036	tetB(60) is a subunit of tetAB(60), an ABC transporter that confers resistance to tetracycline and tigercycline identified by screening a human saliva metagenomic library in Escherichia coli, which is required for resistance.	tetracycline antibiotic	antibiotic efflux	ATP-binding cassette (ABC) antibiotic efflux pump	27.4	3.17E-15	75.9
ECs_0375	ECs_0375	ATP-binding component of sugar ABC transporter	tetB(60)	ARO:3004036	tetB(60) is a subunit of tetAB(60), an ABC transporter that confers resistance to tetracycline and tigercycline identified by screening a human saliva metagenomic library in Escherichia coli, which is required for resistance.	tetracycline antibiotic	antibiotic efflux	ATP-binding cassette (ABC) antibiotic efflux pump	25.4	8.79E-11	60.5

ECs_1495	lolD	lipoprotein-releasing system ATP-binding protein	tetB(46)	ARO:3004033	tetB(46) is a subunit of tetAB(46), a heterodimeric ABC transporter, that is required for conferring tetracycline resistance in Streptococcus australis isolated from the oral cavity.	tetracycline antibiotic	antibiotic efflux	ATP-binding cassette (ABC) antibiotic efflux pump	27.8	2.81E-17	77.4
ECs_3082	narP	two-component regulatory system response regulator NarP	evgA	ARO:3000832	EvgA, when phosphorylated, is a positive regulator for efflux protein complexes emrKY and mdtEF. While usually phosphorylated in a EvgS dependent manner, it can be phosphorylated in the absence of EvgS when overexpressed.	fluoroquinolone antibiotic;macrolide antibiotic;penam;tetracycline antibiotic	antibiotic efflux	major facilitator superfamily (MFS) antibiotic efflux pump;resistance-nodulation-cell division (RND) antibiotic efflux pump	27.3	2.12E-22	87.8
ECs_2652	uvrY	two-component regulatory system response regulator UvrY	evgA	ARO:3000832	EvgA, when phosphorylated, is a positive regulator for efflux protein complexes emrKY and mdtEF. While usually phosphorylated in a EvgS dependent manner, it can be	fluoroquinolone antibiotic;macrolide antibiotic;penam;tetracy	antibiotic efflux	major facilitator superfamily (MFS) antibiotic efflux	27.2	4.67E-21	84.3

					phosphorylated in the absence of EvgS when overexpressed.	cline antibiotic		pump;resistance-nodulation-cell division (RND) antibiotic efflux pump			
ECs_3712	ECs_3712	transcriptional regulator	evgA	ARO:3000832	EvgA, when phosphorylated, is a positive regulator for efflux protein complexes emrKY and mdtEF. While usually phosphorylated in a EvgS dependent manner, it can be phosphorylated in the absence of EvgS when overexpressed.	fluoroquinolone antibiotic;macrolide antibiotic;penam;tetracycline antibiotic	antibiotic efflux	major facilitator superfamily (MFS) antibiotic efflux pump;resistance-nodulation-cell division (RND) antibiotic efflux pump	25.2	3.05E-14	65.9
ECs_0597	sfmZ	response regulator	evgA	ARO:3000832	EvgA, when phosphorylated, is a positive regulator for efflux protein complexes emrKY and mdtEF. While usually phosphorylated in a EvgS dependent manner, it can be	fluoroquinolone antibiotic;macrolide antibiotic;penam;tetracy	antibiotic efflux	major facilitator superfamily (MFS) antibiotic efflux	32.7	5.58E-37	125

					phosphorylated in the absence of EvgS when overexpressed.	cline antibiotic		pump;resistance-nodulation-cell division (RND) antibiotic efflux pump			
ECs_1726	narL	two-component regulatory system response regulator NarL	evgA	ARO:3000832	EvgA, when phosphorylated, is a positive regulator for efflux protein complexes emrKY and mdtEF. While usually phosphorylated in a EvgS dependent manner, it can be phosphorylated in the absence of EvgS when overexpressed.	fluoroquinolone antibiotic;macrolide antibiotic;penam;tetracycline antibiotic	antibiotic efflux	major facilitator superfamily (MFS) antibiotic efflux pump;resistance-nodulation-cell division (RND) antibiotic efflux pump	23.8	3.81E-24	92.4
ECs_0418	ECs_0418	transcription regulator	evgA	ARO:3000832	EvgA, when phosphorylated, is a positive regulator for efflux protein complexes emrKY and mdtEF. While usually phosphorylated in a EvgS dependent manner, it can be	fluoroquinolone antibiotic;macrolide antibiotic;penam;tetracy	antibiotic efflux	major facilitator superfamily (MFS) antibiotic efflux	25.5	2.68E-21	84.7

					phosphorylated in the absence of EvgS when overexpressed.	cline antibiotic		pump;resistance-nodulation-cell division (RND) antibiotic efflux pump			
ECs_1417	csgD	transcriptional regulator	evgA	ARO:3000832	EvgA, when phosphorylated, is a positive regulator for efflux protein complexes emrKY and mdtEF. While usually phosphorylated in a EvgS dependent manner, it can be phosphorylated in the absence of EvgS when overexpressed.	fluoroquinolone antibiotic;macrolide antibiotic;penam;tetracycline antibiotic	antibiotic efflux	major facilitator superfamily (MFS) antibiotic efflux pump;resistance-nodulation-cell division (RND) antibiotic efflux pump	22.7	1.00E-07	47.8
ECs_3106	rcsB	two-component regulatory system response regulator RcsB	evgA	ARO:3000832	EvgA, when phosphorylated, is a positive regulator for efflux protein complexes emrKY and mdtEF. While usually phosphorylated in a EvgS dependent manner, it can be	fluoroquinolone antibiotic;macrolide antibiotic;penam;tetracy	antibiotic efflux	major facilitator superfamily (MFS) antibiotic efflux	25.4	6.82E-16	70.5

					phosphorylated in the absence of EvgS when overexpressed.	cline antibiotic		pump;resistance-nodulation-cell division (RND) antibiotic efflux pump			
ECs_3248	evgA	two-component regulatory system response regulator EvgA	evgA	ARO:3000832	EvgA, when phosphorylated, is a positive regulator for efflux protein complexes emrKY and mdtEF. While usually phosphorylated in a EvgS dependent manner, it can be phosphorylated in the absence of EvgS when overexpressed.	fluoroquinolone antibiotic;macrolide antibiotic;penam;tetracycline antibiotic	antibiotic efflux	major facilitator superfamily (MFS) antibiotic efflux pump;resistance-nodulation-cell division (RND) antibiotic efflux pump	100	6.00E-144	396
ECs_4606	uhpA	two-component regulatory system response regulator UhpA	evgA	ARO:3000832	EvgA, when phosphorylated, is a positive regulator for efflux protein complexes emrKY and mdtEF. While usually phosphorylated in a EvgS dependent manner, it can be	fluoroquinolone antibiotic;macrolide antibiotic;penam;tetracy	antibiotic efflux	major facilitator superfamily (MFS) antibiotic efflux	28.3	8.06E-19	77.8

					phosphorylated in the absence of EvgS when overexpressed.	cline antibiotic		pump;resistance-nodulation-cell division (RND) antibiotic efflux pump			
ECs_1602	phoP	two-component regulatory system response regulator PhoP	arlR	ARO:3000838	ArlR is a response regulator that binds to the norA promoter to activate expression. ArlR must first be phosphorylated by ArlS.	disinfecting agents and antiseptics; fluoroquinolone antibiotic	antibiotic efflux	major facilitator superfamily (MFS) antibiotic efflux pump	32.1	1.07E-34	120
ECs_0722	kdpE	two-component regulatory system response regulator KdpE	arlR	ARO:3000838	ArlR is a response regulator that binds to the norA promoter to activate expression. ArlR must first be phosphorylated by ArlS.	disinfecting agents and antiseptics; fluoroquinolone antibiotic	antibiotic efflux	major facilitator superfamily (MFS) antibiotic efflux pump	39.1	6.93E-46	149
ECs_2707	yedW	response regulator	arlR	ARO:3000838	ArlR is a response regulator that binds to the norA promoter to activate expression. ArlR must first be phosphorylated by ArlS.	disinfecting agents and antiseptics; fluoroquinolone antibiotic	antibiotic efflux	major facilitator superfamily (MFS) antibiotic efflux pump	40.9	8.25E-54	171
ECs_1737	rssB	response	arlR	ARO:3000838	ArlR is a response regulator that	disinfecting	antibiotic	major	32.8	4.67E-1	58.9

		regulator			binds to the norA promoter to activate expression. ArlR must first be phosphorylated by ArlS.	agents and antiseptics; fluoroquinolone antibiotic	efflux	facilitator superfamily (MFS) antibiotic efflux pump		1	
ECs_0609	cusR	two-component regulatory system response regulator CusR	arlR	ARO:3000838	ArlR is a response regulator that binds to the norA promoter to activate expression. ArlR must first be phosphorylated by ArlS.	disinfecting agents and antiseptics; fluoroquinolone antibiotic	antibiotic efflux	major facilitator superfamily (MFS) antibiotic efflux pump	40.2	3.23E-50	160
ECs_4790	glnG	nitrogen regulation protein NR(I)	arlR	ARO:3000838	ArlR is a response regulator that binds to the norA promoter to activate expression. ArlR must first be phosphorylated by ArlS.	disinfecting agents and antiseptics; fluoroquinolone antibiotic	antibiotic efflux	major facilitator superfamily (MFS) antibiotic efflux pump	32.8	3.13E-14	68.9
ECs_0449	phoB	two-component regulatory system response regulator PhoB	arlR	ARO:3000838	ArlR is a response regulator that binds to the norA promoter to activate expression. ArlR must first be phosphorylated by ArlS.	disinfecting agents and antiseptics; fluoroquinolone antibiotic	antibiotic efflux	major facilitator superfamily (MFS) antibiotic efflux pump	36.8	9.76E-50	159
ECs_1150	torR	two-component regulatory system	arlR	ARO:3000838	ArlR is a response regulator that binds to the norA promoter to activate expression. ArlR must	disinfecting agents and antiseptics;	antibiotic efflux	major facilitator superfamily	32.4	7.96E-33	115

		response regulator TorR			first be phosphorylated by ArlS.	fluoroquinolone antibiotic		(MFS) antibiotic efflux pump			
ECs_5095	basR	two-component regulatory system response regulator BasR	arlR	ARO:3000838	ArlR is a response regulator that binds to the norA promoter to activate expression. ArlR must first be phosphorylated by ArlS.	disinfecting agents and antiseptics; fluoroquinolone antibiotic	antibiotic efflux	major facilitator superfamily (MFS) antibiotic efflux pump	39.5	1.41E-41	138
ECs_5359	arcA	two-component regulatory system response regulator	vanR gene in vanA cluster	ARO:3002919	Also known as vanRA, is a vanR variant found in the vanA gene cluster.	glycopeptide antibiotic	antibiotic target alteration	glycopeptide resistance gene cluster;vanR	35.2	2.53E-40	135
ECs_2887	baeR	two-component regulatory system response regulator BaeR	vanR gene in vanA cluster	ARO:3002919	Also known as vanRA, is a vanR variant found in the vanA gene cluster.	glycopeptide antibiotic	antibiotic target alteration	glycopeptide resistance gene cluster;vanR	38.8	1.58E-48	157
ECs_4247	ompR	two-component regulatory system response regulator OmpR	vanR gene in vanA cluster	ARO:3002919	Also known as vanRA, is a vanR variant found in the vanA gene cluster.	glycopeptide antibiotic	antibiotic target alteration	glycopeptide resistance gene cluster;vanR	39.4	6.04E-51	163

ECs_5067	ECs_5067	regulatory protein	ParR	ARO:3005068	ParR is a component of the two-component sensor ParRS. Alongside its counterpart ParS, it confers resistance to polycationic antibiotics through the regulation of efflux components and porins.	aminoglycoside antibiotic;carbapenem;cephalosporin;cephamycin;disinfecting agents and antiseptics;fluoroquinolone antibiotic;macrolide antibiotic;monobactam;penam;penem;phenicol antibiotic;tetracycline antibiotic	antibiotic efflux;reduced permeability to antibiotic	Outer Membrane Porin (Opr);resistance-nodulation-cell division (RND) antibiotic efflux pump	37.9	1.05E-42	142
ECs_5115	cadC	cadBA operon transcriptional activator	ParR	ARO:3005068	ParR is a component of the two-component sensor ParRS. Alongside its counterpart ParS, it confers resistance to polycationic antibiotics through the regulation of efflux	aminoglycoside antibiotic;carbapenem;cephalosporin;cephamycin;d	antibiotic efflux;reduced permeability to antibiotic	Outer Membrane Porin (Opr);resistance-nodulation-cell	39.5	6.13E-06	44.7

					components and porins.	isinfecting agents and antiseptics;fluoroquinolone antibiotic;macrolide antibiotic;monobactam;penam;penem;phenicol antibiotic;tetracycline antibiotic		division (RND) antibiotic efflux pump			
ECs_2314	rstA	two-component regulatory system response regulator rstA	ParR	ARO:3005068	ParR is a component of the two-component sensor ParRS. Alongside its counterpart ParS, it confers resistance to polycationic antibiotics through the regulation of efflux components and porins.	aminoglycoside antibiotic;carbapenem;cephalosporin;cephamycin;disinfecting agents and antiseptics;fluoroquinolone antibiotic;m	antibiotic efflux;reduced permeability to antibiotic	Outer Membrane Porin (Opr);resistance-nodulation-cell division (RND) antibiotic efflux pump	45.2	3.08E-68	207

						acrolide antibiotic;monobactam;penam;penem;phenicol antibiotic;tetracycline antibiotic					
ECs_0897	ybiT	ABC-F family regulatory ATPase	carA	ARO:3002817	carA is an ABC-F subfamily protein involved in macrolide resistance. It is found in Streptomyces thermotolerans.	macrolide antibiotic	antibiotic target protection	Miscellaneous ABC-F subfamily ATP-binding cassette ribosomal protection proteins	30.2	1.00E-59	204
ECs_5349	ettA	energy-dependent translational throttle A	carA	ARO:3002817	carA is an ABC-F subfamily protein involved in macrolide resistance. It is found in Streptomyces thermotolerans.	macrolide antibiotic	antibiotic target protection	Miscellaneous ABC-F subfamily ATP-binding cassette ribosomal protection proteins	32.7	1.66E-64	217
ECs_3530	stpA	DNA binding protein	H-NS	ARO:3000676	H-NS is a histone-like protein involved in global gene	cephalosporin;cephamycin	antibiotic efflux	major facilitator	60	4.65E-41	130

					regulation in Gram-negative bacteria. It is a repressor of the membrane fusion protein genes acrE, mdtE, and emrK as well as nearby genes of many RND-type multidrug exporters.	;fluoroquinolone antibiotic;macrolide antibiotic;penam;tetracycline antibiotic		superfamily (MFS) antibiotic efflux pump;resistance-nodulation-cell division (RND) antibiotic efflux pump			
ECs_1739	hns	global DNA-binding transcriptional dual regulator H-NS	H-NS	ARO:3000676	H-NS is a histone-like protein involved in global gene regulation in Gram-negative bacteria. It is a repressor of the membrane fusion protein genes acrE, mdtE, and emrK as well as nearby genes of many RND-type multidrug exporters.	cephalosporin;cephamycin;fluoroquinolone antibiotic;macrolide antibiotic;penam;tetracycline antibiotic	antibiotic efflux	major facilitator superfamily (MFS) antibiotic efflux pump;resistance-nodulation-cell division (RND) antibiotic efflux pump	100	3.20E-86	245
ECs_4793	typA	GTP-binding protein	tet(T)	ARO:3000193	Tet(T) is a ribosomal protection protein of streptococci. It is	tetracycline antibiotic	antibiotic target	tetracycline-resistant	39.2	1.49E-27	114

					similar to Tet(Q).		protection	ribosomal protection protein			
ECs_5333	prfC	peptide chain release factor RF-3	tet(T)	ARO:3000193	Tet(T) is a ribosomal protection protein of streptococci. It is similar to Tet(Q).	tetracycline antibiotic	antibiotic target protection	tetracycline-resistant ribosomal protection protein	28.6	1.60E-38	146
ECs_4203	yheS	ABC-F family regulatory ATPase	vmlR	ARO:3004476	vmlR is an ABC-F ATPase ribosomal protection protein identified in Bacillus subtilus. Shown to confer resistance to lincomycin and streptogramin A virginiamycin. Described by Crowe-McAuliffe et al. 2018.	lincosamide antibiotic;streptogramin B antibiotic;streptogramin antibiotic	antibiotic target protection	Miscellaneous ABC-F subfamily ATP-binding cassette ribosomal protection proteins	29.1	1.46E-57	200
ECs_3923	tolC	transport channel	TolC	ARO:3000237	TolC is a protein subunit of many multidrug efflux complexes in Gram negative bacteria. It is an outer membrane efflux protein and is constitutively open. Regulation of efflux activity is often at its periplasmic entrance by other components of the efflux complex.	aminocoumarin antibiotic;aminoglycoside antibiotic;carbapenem;cephalosporin;cephamycin;disinfecting	antibiotic efflux	ATP-binding cassette (ABC) antibiotic efflux pump;major facilitator superfamily (MFS) antibiotic	99.8	0	917

						agents and antiseptics; fluoroquinolone antibiotic;glycylcycline;macrolide antibiotic;penam;penem;peptide antibiotic;phenicol antibiotic;rifamycin antibiotic;tetracycline antibiotic		efflux pump;resistance-nodulation-cell division (RND) antibiotic efflux pump			
ECs_0540	ECs_0540	outer membrane transport protein	TolC	ARO:3000237	TolC is a protein subunit of many multidrug efflux complexes in Gram negative bacteria. It is an outer membrane efflux protein and is constitutively open. Regulation of efflux activity is often at its periplasmic entrance by other components of the efflux complex.	aminocoumarin antibiotic;aminoglycoside antibiotic;carbapenem;cephalosporin;cephamycin;d	antibiotic efflux	ATP-binding cassette (ABC) antibiotic efflux pump;major facilitator superfamily (MFS)	24.9	7.07E-15	73.6

						isinfecting agents and antiseptics; fluoroquinolone antibiotic;glycylcycline;macrolide antibiotic;penam;penem;peptide antibiotic;phenicol antibiotic;rifamycin antibiotic;tetracycline antibiotic		antibiotic efflux pump;resistance-nodulation-cell division (RND) antibiotic efflux pump			
ECs_3443	yfiE	transcriptional regulator	adeL	ARO:3000620	AdeL is a regulator of AdeFGH in Acinetobacter baumannii. AdeL mutations are associated with AdeFGH overexpression and multidrug resistance.	fluoroquinolone antibiotic;tetracycline antibiotic	antibiotic efflux	resistance-nodulation-cell division (RND) antibiotic efflux pump	24.9	1.23E-06	46.2
ECs_4461	yiaU	transcriptional regulator	adeL	ARO:3000620	AdeL is a regulator of AdeFGH in Acinetobacter baumannii. AdeL	fluoroquinolone	antibiotic efflux	resistance-nodulation-ce	23.8	5.93E-11	59.7

					mutations are associated with AdeFGH overexpression and multidrug resistance.	antibiotic;tetracycline antibiotic		ll division (RND) antibiotic efflux pump		
ECs_3943	ttdR	transcriptional activator	adeL	ARO:3000620	AdeL is a regulator of AdeFGH in Acinetobacter baumannii. AdeL mutations are associated with AdeFGH overexpression and multidrug resistance.	fluoroquinolone antibiotic;tetracycline antibiotic	antibiotic efflux	resistance-nodulation-cell division (RND) antibiotic efflux pump	31.1	3.47E-37	132
ECs_0309	ECs_0309	transcriptional regulator	adeL	ARO:3000620	AdeL is a regulator of AdeFGH in Acinetobacter baumannii. AdeL mutations are associated with AdeFGH overexpression and multidrug resistance.	fluoroquinolone antibiotic;tetracycline antibiotic	antibiotic efflux	resistance-nodulation-cell division (RND) antibiotic efflux pump	29.3	4.29E-29	110
ECs_3907	qseB	quorum sensing DNA-binding response regulator in two-component regulatory system with QseC	baeR	ARO:3000828	BaeR is a response regulator that promotes the expression of MdtABC and AcrD efflux complexes.	aminocoumarin antibiotic;aminoglycoside antibiotic	antibiotic efflux	resistance-nodulation-cell division (RND) antibiotic efflux pump	34.5	4.77E-37	127
ECs_5356	creB	two-component	baeR	ARO:3000828	BaeR is a response regulator that	aminocoumari	antibiotic	resistance-n	42.7	5.51E-5	171

		regulatory system response regulator CreR			promotes the expression of MdtABC and AcrD efflux complexes.	n antibiotic;aminoglycoside antibiotic	efflux	odulation-cell division (RND) antibiotic efflux pump		4	
ECs_3420	glrR	two-component regulatory system response regulator glrR	Pseudomonas aeruginosa CpxR	ARO:3004054	CpxR is directly involved in activation of expression of RND efflux pump MexAB-OprM in P. aeruginosa. CpxR is required to enhance mexAB-oprM expression and drug resistance, in the absence of repressor MexR.	aminocoumarin antibiotic;aminoglycoside antibiotic;carbapenem;cephalosporin;cephamycin;diaminopyrimidine antibiotic;fluoroquinolone antibiotic;macrolide antibiotic;monobactam;penam;penem;peptide antibiotic;p	antibiotic efflux	resistance-nodulation-cell division (RND) antibiotic efflux pump	32.8	1.79E-16	75.5

ECs_0659	citB	response regulator in two-component regulatory system with CitA	Pseudomonas aeruginosa CpxR	ARO:3004054	CpxR is directly involved in activation of expression of RND efflux pump MexAB-OprM in P. aeruginosa. CpxR is required to enhance mexAB-oprM expression and drug resistance, in the absence of repressor MexR.	henicol antibiotic;sulfonamide antibiotic;tetracycline antibiotic aminocoumarin antibiotic;aminoglycoside antibiotic;carbapenem;cephalosporin;cephamycin;diaminopyrimidine antibiotic;fluoroquinolone antibiotic;macrolide antibiotic;monobactam;penam;penem;peptide	antibiotic efflux	resistance-nodulation-cell division (RND) antibiotic efflux pump	25.6	7.20E-08	48.5

ECs_4838	cpxR	two-component regulatory system response regulator CpxR	Pseudomonas aeruginosa CpxR	ARO:3004054	CpxR is directly involved in activation of expression of RND efflux pump MexAB-OprM in P. aeruginosa. CpxR is required to enhance mexAB-oprM expression and drug resistance, in the absence of repressor MexR.	antibiotic;phenicol antibiotic;sulfonamide antibiotic;tetracycline antibiotic aminocoumarin antibiotic;aminoglycoside antibiotic;carbapenem;cephalosporin;cephamycin;diaminopyrimidine antibiotic;fluoroquinolone antibiotic;macrolide antibiotic;monobactam;penam;penem;pe	antibiotic efflux	resistance-nodulation-cell division (RND) antibiotic efflux pump	47.8	7.50E-65	198

ECs_4927	zraR	transcriptional regulatory protein ZraR	Pseudomonas aeruginosa CpxR	ARO:3004054	CpxR is directly involved in activation of expression of RND efflux pump MexAB-OprM in P. aeruginosa. CpxR is required to enhance mexAB-oprM expression and drug resistance, in the absence of repressor MexR.	ptide antibiotic;phenicol antibiotic;sulfonamide antibiotic;tetracycline antibiotic aminocoumarin antibiotic;aminoglycoside antibiotic;carbapenem;cephalosporin;cephamycin;diaminopyrimidine antibiotic;fluoroquinolone antibiotic;macrolide antibiotic;monobactam;pe	antibiotic efflux	resistance-nodulation-cell division (RND) antibiotic efflux pump	33.6	2.76E-15	72

						nam;penem;peptide antibiotic;phenicol antibiotic;sulfonamide antibiotic;tetracycline antibiotic					
ECs_3261	ypdB	response regulator	vanR gene in vanC cluster	ARO:3002922	Also known as vanRC, is a vanR variant found in the vanC gene cluster.	glycopeptide antibiotic	antibiotic target alteration	glycopeptide resistance gene cluster;vanR	29.2	7.43E-07	45.8
ECs_2593	cheB	chemotaxis-specific methylesterase	vanR gene in vanC cluster	ARO:3002922	Also known as vanRC, is a vanR variant found in the vanC gene cluster.	glycopeptide antibiotic	antibiotic target alteration	glycopeptide resistance gene cluster;vanR	31.8	4.64E-06	44.3
ECs_5064	mdtN	membrane fusion protein of efflux pump	mdtN	ARO:3003548	Multidrug resistance efflux pump. Could be involved in resistance to puromycin, acriflavine and tetraphenylarsonium chloride.	disinfecting agents and antiseptics; nucleoside antibiotic	antibiotic efflux	major facilitator superfamily (MFS) antibiotic efflux pump	99.4	2.80E-232	631
ECs_0873	ybhG	membrane fusion protein	mdtN	ARO:3003548	Multidrug resistance efflux pump. Could be involved in resistance to puromycin,	disinfecting agents and antiseptics;	antibiotic efflux	major facilitator superfamily	22	8.82E-10	56.2

		component of efflux pump			acriflavine and tetraphenylarsonium chloride.	nucleoside antibiotic		(MFS) antibiotic efflux pump			
ECs_3247	emrK	multidrug resistance efflux pump membrane fusion protein	mdtN	ARO:3003548	Multidrug resistance efflux pump. Could be involved in resistance to puromycin, acriflavine and tetraphenylarsonium chloride.	disinfecting agents and antiseptics; nucleoside antibiotic	antibiotic efflux	major facilitator superfamily (MFS) antibiotic efflux pump	27.4	9.40E-29	112
ECs_2353	ydhJ	membrane fusion protein of YdhJK efflux pump	mdtN	ARO:3003548	Multidrug resistance efflux pump. Could be involved in resistance to puromycin, acriflavine and tetraphenylarsonium chloride.	disinfecting agents and antiseptics; nucleoside antibiotic	antibiotic efflux	major facilitator superfamily (MFS) antibiotic efflux pump	31.2	2.73E-38	135
ECs_4462	yibH	membrane fusion protein component of efflux pump	mdtN	ARO:3003548	Multidrug resistance efflux pump. Could be involved in resistance to puromycin, acriflavine and tetraphenylarsonium chloride.	disinfecting agents and antiseptics; nucleoside antibiotic	antibiotic efflux	major facilitator superfamily (MFS) antibiotic efflux pump	28.3	6.03E-20	87
ECs_4114	aaeA	p-hydroxybenzoic acid efflux system component	mdtN	ARO:3003548	Multidrug resistance efflux pump. Could be involved in resistance to puromycin, acriflavine and tetraphenylarsonium chloride.	disinfecting agents and antiseptics; nucleoside antibiotic	antibiotic efflux	major facilitator superfamily (MFS) antibiotic	28.9	2.63E-25	100

								efflux pump			
ECs_3547	emrA	multidrug efflux system protein	mdtN	ARO:3003548	Multidrug resistance efflux pump. Could be involved in resistance to puromycin, acriflavine and tetraphenylarsonium chloride.	disinfecting agents and antiseptics; nucleoside antibiotic	antibiotic efflux	major facilitator superfamily (MFS) antibiotic efflux pump	28.2	6.71E-28	109
ECs_4473	yibH	membrane fusion protein component of efflux pump	mdtN	ARO:3003548	Multidrug resistance efflux pump. Could be involved in resistance to puromycin, acriflavine and tetraphenylarsonium chloride.	disinfecting agents and antiseptics; nucleoside antibiotic	antibiotic efflux	major facilitator superfamily (MFS) antibiotic efflux pump	26.5	1.27E-18	83.2
ECs_5063	mdtO	multidrug resistance protein MdtO	mdtO	ARO:3003549	Multidrug resistance efflux pump. Could be involved in resistance to puromycin, acriflavine and tetraphenylarsonium chloride.	disinfecting agents and antiseptics; nucleoside antibiotic	antibiotic efflux	major facilitator superfamily (MFS) antibiotic efflux pump	99	0	1322
ECs_0964	macA	macrolide transporter membrane fusion protein component	macA	ARO:3000533	MacA is a membrane fusion protein that forms an antibiotic efflux complex with MacB and TolC. macA corresponds to 1 locus in Pseudomonas aeruginosa PAO1 and 1 locus in Pseudomonas aeruginosa LESB58.	macrolide antibiotic	antibiotic efflux	ATP-binding cassette (ABC) antibiotic efflux pump	36.2	1.86E-57	189

ECs_0612	cusB	membrane fusion protein of copper/silver efflux system	macA	ARO:3000533	MacA is a membrane fusion protein that forms an antibiotic efflux complex with MacB and TolC. macA corresponds to 1 locus in Pseudomonas aeruginosa PAO1 and 1 locus in Pseudomonas aeruginosa LESB58.	macrolide antibiotic	antibiotic efflux	ATP-binding cassette (ABC) antibiotic efflux pump	22.4	8.47E-08	50.8
ECs_2882	mdtA	multidrug efflux system subunit A	adeG	ARO:3000778	AdeG is the inner membrane transporter of the AdeFGH multidrug efflux complex.	fluoroquinolone antibiotic;tetracycline antibiotic	antibiotic efflux	resistance-nodulation-cell division (RND) antibiotic efflux pump	32.7	4.22E-51	174
ECs_5040	uvrA	excinuclease ABC system protein UvrA	macB	ARO:3000535	MacB is an ATP-binding cassette (ABC) transporter that exports macrolides with 14- or 15-membered lactones. It forms an antibiotic efflux complex with MacA and TolC. macB corresponds to 1 locus in Pseudomonas aeruginosa PAO1 and 1 locus in Pseudomonas aeruginosa LESB58.	macrolide antibiotic	antibiotic efflux	ATP-binding cassette (ABC) antibiotic efflux pump	29.6	2.53E-08	54.7
ECs_3803	ECs_3803	ABC transporter ATP-binding protein	macB	ARO:3000535	MacB is an ATP-binding cassette (ABC) transporter that exports macrolides with 14- or 15-membered lactones. It forms an	macrolide antibiotic	antibiotic efflux	ATP-binding cassette (ABC) antibiotic	25.5	2.12E-13	65.9

					antibiotic efflux complex with MacA and TolC. macB corresponds to 1 locus in Pseudomonas aeruginosa PAO1 and 1 locus in Pseudomonas aeruginosa LESB58.			efflux pump			
ECs_0189	yaeR	lyase	FosA6	ARO:3004111	FosA6 is a plasmid-encoded enzyme that confers resistance to fosfomycin in Escherichia coli by breaking the epoxide ring of the molecule.	phosphonic acid antibiotic	antibiotic inactivation	fosfomycin thiol transferase	31.5	2.70E-07	43.9
ECs_2052	mnaT	methionine N-acyltransferase	sta	ARO:3004699	Streptothricin acetyltransferase gene (STAT gene) that confers streptothricin resistance on Escherichia coli and Bacillus subtilis.	nucleoside antibiotic	antibiotic inactivation	streptothricin acetyltransferase (SAT)	32.5	1.07E-06	43.9
ECs_4136	acrS	transcriptional regulator	AcrS	ARO:3000656	AcrS is a repressor of the AcrAB efflux complex and is associated with the expression of AcrEF. AcrS is believed to regulate a switch between AcrAB and AcrEF efflux.	cephalosporin;cephamycin;disinfecting agents and antiseptics;fluoroquinolone antibiotic;glycylcycline;penam;pheni	antibiotic efflux	resistance-nodulation-cell division (RND) antibiotic efflux pump	100	2.84E-159	436

						col antibiotic;rifamycin antibiotic;tetracycline antibiotic					
ECs_0517	acrR	transcriptional repressor	AcrS	ARO:3000656	AcrS is a repressor of the AcrAB efflux complex and is associated with the expression of AcrEF. AcrS is believed to regulate a switch between AcrAB and AcrEF efflux.	cephalosporin;cephamycin;disinfecting agents and antiseptics;fluoroquinolone antibiotic;glycylcycline;penam;phenicol antibiotic;rifamycin antibiotic;tetracycline antibiotic	antibiotic efflux	resistance-nodulation-cell division (RND) antibiotic efflux pump	37.7	1.27E-37	127
ECs_0068	araC	ara regulon transcriptional activator	mtrA	ARO:3000816	MtrA is a transcriptional activator of the MtrCDE multidrug efflux pump of Neisseria gonorrhoeae.	macrolide antibiotic;penam	antibiotic efflux	resistance-nodulation-cell division (RND)	26.5	1.97E-06	45.4

								antibiotic efflux pump			
ECs_4452	xylR	xylose divergent operon transcriptional activator	mtrA	ARO:3000816	MtrA is a transcriptional activator of the MtrCDE multidrug efflux pump of Neisseria gonorrhoeae.	macrolide antibiotic;penam	antibiotic efflux	resistance-nodulation-cell division (RND) antibiotic efflux pump	34.6	7.68E-10	56.6
ECs_4621	yidL	AraC family transcriptional regulator	mtrA	ARO:3000816	MtrA is a transcriptional activator of the MtrCDE multidrug efflux pump of Neisseria gonorrhoeae.	macrolide antibiotic;penam	antibiotic efflux	resistance-nodulation-cell division (RND) antibiotic efflux pump	30.8	4.47E-09	53.5
ECs_3894	yqhC	transcriptional activator of yqhD	mtrA	ARO:3000816	MtrA is a transcriptional activator of the MtrCDE multidrug efflux pump of Neisseria gonorrhoeae.	macrolide antibiotic;penam	antibiotic efflux	resistance-nodulation-cell division (RND) antibiotic efflux pump	26	2.91E-09	54.3
ECs_0343	rclR	reactive chlorine species-specific activator of the rcl genes	mtrA	ARO:3000816	MtrA is a transcriptional activator of the MtrCDE multidrug efflux pump of Neisseria gonorrhoeae.	macrolide antibiotic;penam	antibiotic efflux	resistance-nodulation-cell division (RND) antibiotic efflux pump	27.8	3.06E-29	110

ECs_2416	btuD	vitamin B12 ABC transporter ATPase	optrA	ARO:3003746	OptrA is a member of the ABC-F protein subfamily that confers resistance to oxazolidinones. The gene encoding the protein was originally isolated from a plasmid in Enterococcus faecalis and Enterococcus faecium.	oxazolidinone antibiotic;phenicol antibiotic	antibiotic target protection	Miscellaneous ABC-F subfamily ATP-binding cassette ribosomal protection proteins	29	1.41E-08	52
ECs_1033	uup	replication regulatory ABC-F family DNA-binding ATPase	optrA	ARO:3003746	OptrA is a member of the ABC-F protein subfamily that confers resistance to oxazolidinones. The gene encoding the protein was originally isolated from a plasmid in Enterococcus faecalis and Enterococcus faecium.	oxazolidinone antibiotic;phenicol antibiotic	antibiotic target protection	Miscellaneous ABC-F subfamily ATP-binding cassette ribosomal protection proteins	30.6	1.91E-66	227
ECs_5331	rimI	ribosomal-protein-S18-alanine N-acetyltransferase	AAC(3)-Ia	ARO:3002528	AAC(3)-Ia is an aminoglycoside acetyltransferase encoded by plasmids, transposons, integrons in S. marcescens, E. coli, Acinetobacter baumannii, Klebsiella pneumoniae, Klebsiella oxytoca, P. aeruginosa, Salmonella typhimurium and Proteus mirabilis.	aminoglycoside antibiotic	antibiotic inactivation	AAC(3)	26.4	3.40E-06	41.6

ECs_0515	acrB_1	multidrug efflux system protein	acrB	ARO:3000216	Protein subunit of AcrA-AcrB-TolC multidrug efflux complex. AcrB functions as a herterotrimer which forms the inner membrane component and is primarily responsible for substrate recognition and energy transduction by acting as a drug/proton antiporter.	cephalosporin;disinfecting agents and antiseptics;fluoroquinolone antibiotic;glycylcycline;penam;phenicol antibiotic;rifamycin antibiotic;tetracycline antibiotic	antibiotic efflux	resistance-nodulation-cell division (RND) antibiotic efflux pump	99.8	0	1979
ECs_3332	acrB	multidrug efflux system protein	acrB	ARO:3000216	Protein subunit of AcrA-AcrB-TolC multidrug efflux complex. AcrB functions as a herterotrimer which forms the inner membrane component and is primarily responsible for substrate recognition and energy transduction by acting as a drug/proton antiporter.	cephalosporin;disinfecting agents and antiseptics;fluoroquinolone antibiotic;glycylcycline;penam;phenicol antibiotic;r	antibiotic efflux	resistance-nodulation-cell division (RND) antibiotic efflux pump	66	0	1296

						ifamycin antibiotic;tetracycline antibiotic					
ECs_4394	acrB_5	multidrug efflux system protein	acrB	ARO:3000216	Protein subunit of AcrA-AcrB-TolC multidrug efflux complex. AcrB functions as a herterotrimer which forms the inner membrane component and is primarily responsible for substrate recognition and energy transduction by acting as a drug/proton antiporter.	cephalosporin;disinfecting agents and antiseptics;fluoroquinolone antibiotic;glycylcycline;penam;phenicol antibiotic;rifamycin antibiotic;tetracycline antibiotic	antibiotic efflux	resistance-nodulation-cell division (RND) antibiotic efflux pump	71.2	0	1416
ECs_1864	acrB_2	multidrug efflux system protein	acrB	ARO:3000216	Protein subunit of AcrA-AcrB-TolC multidrug efflux complex. AcrB functions as a herterotrimer which forms the inner membrane component and is primarily responsible for substrate recognition and energy	cephalosporin;disinfecting agents and antiseptics;fluoroquinolone antibiotic;g	antibiotic efflux	resistance-nodulation-cell division (RND) antibiotic efflux pump	56.3	0	1031

					transduction by acting as a drug/proton antiporter.	lycylcycline;penam;phenicol antibiotic;rifamycin antibiotic;tetracycline antibiotic					
ECs_0516	acrA	multidrug efflux system	Escherichia coli acrA	ARO:3004043	AcrA is a subunit of the AcrAB-TolC multidrug efflux system that in E. coli.	cephalosporin;disinfecting agents and antiseptics;fluoroquinolone antibiotic;glycylcycline;penam;phenicol antibiotic;rifamycin antibiotic;tetracycline antibiotic	antibiotic efflux	resistance-nodulation-cell division (RND) antibiotic efflux pump	100	7.15E-276	746
ECs_4137	acrE	multidrug transporter	Escherichia coli acrA	ARO:3004043	AcrA is a subunit of the AcrAB-TolC multidrug efflux system that in E. coli.	cephalosporin;disinfecting agents and	antibiotic efflux	resistance-nodulation-cell division	68	7.19E-180	503

						antiseptics; fluoroquinolone antibiotic;glycylcycline;penam;phenicol antibiotic;rifamycin antibiotic;tetracycline antibiotic		(RND) antibiotic efflux pump			
ECs_4393	mdtE	anaerobic multidrug efflux transporter	Escherichia coli acrA	ARO:3004043	AcrA is a subunit of the AcrAB-TolC multidrug efflux system that in E. coli.	cephalosporin;disinfecting agents and antiseptics;fluoroquinolone antibiotic;glycylcycline;penam;phenicol antibiotic;rifamycin antibiotic;tetracycline	antibiotic efflux	resistance-nodulation-cell division (RND) antibiotic efflux pump	54.5	1.69E-134	387

ECs_1863	eefA	multidrug-efflux transport protein precursor	Escherichia coli acrA	ARO:3004043	AcrA is a subunit of the AcrAB-TolC multidrug efflux system that in E. coli.	antibiotic cephalosporin;disinfecting agents and antiseptics; fluoroquinolone antibiotic;glycylcycline;penam;phenicol antibiotic;rifamycin antibiotic;tetracycline antibiotic	antibiotic efflux	resistance-nodulation-cell division (RND) antibiotic efflux pump	51.7	3.60E-107	317
ECs_1259	rutR	transcriptional regulator	MexL	ARO:3003710	MexL is a specific repressor of mexJK transcription and autoregulates its own expression.	disinfecting agents and antiseptics; macrolide antibiotic;tetracycline antibiotic	antibiotic efflux	resistance-nodulation-cell division (RND) antibiotic efflux pump	32.2	5.51E-08	48.5
ECs_2326	uidR	transcriptional repressor	MexL	ARO:3003710	MexL is a specific repressor of mexJK transcription and autoregulates its own	disinfecting agents and antiseptics;	antibiotic efflux	resistance-nodulation-cell division	25.5	1.31E-06	44.3

					expression.	macrolide antibiotic;tetracycline antibiotic		(RND) antibiotic efflux pump			
ECs_1489	ycfQ	TetR family transcriptional regulator	MexL	ARO:3003710	MexL is a specific repressor of mexJK transcription and autoregulates its own expression.	disinfecting agents and antiseptics;macrolide antibiotic;tetracycline antibiotic	antibiotic efflux	resistance-nodulation-cell division (RND) antibiotic efflux pump	40	6.45E-06	42.7
ECs_0512	maa	maltose O-acetyltransferase	vatB	ARO:3002841	vatB is a plasmid-mediated acetyltransferase found in Staphylococcus aureus.	streptogramin A antibiotic;streptogramin antibiotic	antibiotic inactivation	streptogramin vat acetyltransferase	47.1	4.01E-09	51.2
ECs_0395	lacA	galactoside O-acetyltransferase	vatB	ARO:3002841	vatB is a plasmid-mediated acetyltransferase found in Staphylococcus aureus.	streptogramin A antibiotic;streptogramin antibiotic	antibiotic inactivation	streptogramin vat acetyltransferase	47.1	5.53E-13	62.4
ECs_2859	wcaF	acyl transferase	vatB	ARO:3002841	vatB is a plasmid-mediated acetyltransferase found in Staphylococcus aureus.	streptogramin A antibiotic;streptogramin antibiotic	antibiotic inactivation	streptogramin vat acetyltransferase	43.9	8.69E-11	55.8

ECs_0426	ampH	D-alanyl-D-alanine-carboxypeptidase/endopeptidase	SST-1	ARO:3006999	SST-1 is a SST beta-lactamase.	cephalosporin	antibiotic inactivation	SST beta-lactamase	26	1.46E-20	89.4
ECs_0642	ybdO	transcriptional regulator	leuO	ARO:3003843	leuO, a LysR family transcription factor, exists in a wide variety of bacteria of the family Enterobacteriaceae and is involved in the regulation of as yet unidentified genes affecting the stress response and pathogenesis expression. LeuO is also an activator of the MdtNOP efflux pump.	disinfecting agents and antiseptics; nucleoside antibiotic	antibiotic efflux	major facilitator superfamily (MFS) antibiotic efflux pump	23	5.93E-18	79.7
ECs_0667	ybeF	LysR family transcriptional regulator	leuO	ARO:3003843	leuO, a LysR family transcription factor, exists in a wide variety of bacteria of the family Enterobacteriaceae and is involved in the regulation of as yet unidentified genes affecting the stress response and pathogenesis expression. LeuO is also an activator of the MdtNOP efflux pump.	disinfecting agents and antiseptics; nucleoside antibiotic	antibiotic efflux	major facilitator superfamily (MFS) antibiotic efflux pump	26.1	1.81E-23	95.5
ECs_3998	tdcA	tdc operon transcription	leuO	ARO:3003843	leuO, a LysR family transcription factor, exists in a wide variety	disinfecting agents and	antibiotic efflux	major facilitator	27.7	4.06E-08	50.8

		al activator			of bacteria of the family Enterobacteriaceae and is involved in the regulation of as yet unidentified genes affecting the stress response and pathogenesis expression. LeuO is also an activator of the MdtNOP efflux pump.	antiseptics; nucleoside antibiotic		superfamily (MFS) antibiotic efflux pump			
ECs_2784	nac	nitrogen assimilation regulon transcription al regulator	leuO	ARO:3003843	leuO, a LysR family transcription factor, exists in a wide variety of bacteria of the family Enterobacteriaceae and is involved in the regulation of as yet unidentified genes affecting the stress response and pathogenesis expression. LeuO is also an activator of the MdtNOP efflux pump.	disinfecting agents and antiseptics; nucleoside antibiotic	antibiotic efflux	major facilitator superfamily (MFS) antibiotic efflux pump	23.4	1.65E-07	48.9
ECs_3696	lysR	transcription al activator	leuO	ARO:3003843	leuO, a LysR family transcription factor, exists in a wide variety of bacteria of the family Enterobacteriaceae and is involved in the regulation of as yet unidentified genes affecting the stress response and pathogenesis expression. LeuO is	disinfecting agents and antiseptics; nucleoside antibiotic	antibiotic efflux	major facilitator superfamily (MFS) antibiotic efflux pump	36.6	3.07E-07	48.1

					also an activator of the MdtNOP efflux pump.						
ECs_4648	yidZ	transcriptional regulator	leuO	ARO:3003843	leuO, a LysR family transcription factor, exists in a wide variety of bacteria of the family Enterobacteriaceae and is involved in the regulation of as yet unidentified genes affecting the stress response and pathogenesis expression. LeuO is also an activator of the MdtNOP efflux pump.	disinfecting agents and antiseptics; nucleoside antibiotic	antibiotic efflux	major facilitator superfamily (MFS) antibiotic efflux pump	22.9	1.34E-13	67.4
ECs_4157	zntR	zntA gene transcriptional activator	golS	ARO:3000504	GolS is a regulator activated by the presence of golD, and promotes the expression of the MdsABC efflux pump.	carbapenem;cephalosporin;cephamycin;monobactam;penam;penem;phenicol antibiotic	antibiotic efflux	resistance-nodulation-cell division (RND) antibiotic efflux pump	36.4	9.38E-19	74.7
ECs_0545	cueR	copper-responsive regulon transcriptional regulator	golS	ARO:3000504	GolS is a regulator activated by the presence of golD, and promotes the expression of the MdsABC efflux pump.	carbapenem;cephalosporin;cephamycin;monobactam;penam;penem;phenicol antibiotic	antibiotic efflux	resistance-nodulation-cell division (RND) antibiotic efflux pump	42.9	2.39E-32	109

ECs_1902	tyrR	aromatic amino acid biosynthesis and transport regulon transcriptional regulator	TxR	ARO:3005008	TxR is a putative transcription regulator that plays a role in conferring tetracycline resistance. It is required for proper functioning of Tet35.	tetracycline antibiotic	antibiotic efflux	ATP-binding cassette (ABC) antibiotic efflux pump	32.4	2.33E-48	167
ECs_0384	prpR	propionate catabolism operon regulatory protein	TxR	ARO:3005008	TxR is a putative transcription regulator that plays a role in conferring tetracycline resistance. It is required for proper functioning of Tet35.	tetracycline antibiotic	antibiotic efflux	ATP-binding cassette (ABC) antibiotic efflux pump	41.2	3.86E-60	198
ECs_3742	ygeV	sigma-54-interacting transcriptional activator	TxR	ARO:3005008	TxR is a putative transcription regulator that plays a role in conferring tetracycline resistance. It is required for proper functioning of Tet35.	tetracycline antibiotic	antibiotic efflux	ATP-binding cassette (ABC) antibiotic efflux pump	40.2	8.57E-64	209
ECs_1880	pspF	psp operon transcriptional activator	TxR	ARO:3005008	TxR is a putative transcription regulator that plays a role in conferring tetracycline resistance. It is required for proper functioning of Tet35.	tetracycline antibiotic	antibiotic efflux	ATP-binding cassette (ABC) antibiotic efflux pump	32.6	1.47E-50	167
ECs_3587	fhlA	formate hydrogenlyase transcriptional activator	TxR	ARO:3005008	TxR is a putative transcription regulator that plays a role in conferring tetracycline resistance. It is required for	tetracycline antibiotic	antibiotic efflux	ATP-binding cassette (ABC) antibiotic	39.2	2.48E-67	221

					proper functioning of Tet35.			efflux pump			
ECs_3565	norR	anaerobic nitric oxide reductase DNA-binding transcriptional activator	TxR	ARO:3005008	TxR is a putative transcription regulator that plays a role in conferring tetracycline resistance. It is required for proper functioning of Tet35.	tetracycline antibiotic	antibiotic efflux	ATP-binding cassette (ABC) antibiotic efflux pump	40.8	4.44E-65	211
ECs_3353	hyfR	transcriptional activator HyfR	TxR	ARO:3005008	TxR is a putative transcription regulator that plays a role in conferring tetracycline resistance. It is required for proper functioning of Tet35.	tetracycline antibiotic	antibiotic efflux	ATP-binding cassette (ABC) antibiotic efflux pump	44.7	9.44E-65	214
ECs_4265	rtcR	transcriptional regulatory protein RtcR	TxR	ARO:3005008	TxR is a putative transcription regulator that plays a role in conferring tetracycline resistance. It is required for proper functioning of Tet35.	tetracycline antibiotic	antibiotic efflux	ATP-binding cassette (ABC) antibiotic efflux pump	45.9	3.90E-33	125
ECs_3698	galP	D-galactose transporter	lfrA	ARO:3003967	lfrA is involved in the active efflux of quinolones and is found in Mycobacteroides abscessus.	fluoroquinolone antibiotic	antibiotic efflux	major facilitator superfamily (MFS) antibiotic efflux pump	31.2	8.49E-10	57.8
ECs_3819	galP_2	D-galactose transporter	lfrA	ARO:3003967	lfrA is involved in the active efflux of quinolones and is found in Mycobacteroides abscessus.	fluoroquinolone antibiotic	antibiotic efflux	major facilitator superfamily	41.7	1.53E-10	60.1

								(MFS) antibiotic efflux pump			
ECs_0408	mhpT	3-hydroxyphenylpropionic transporter	QepA2	ARO:3004103	QepA2 is a plasmid-mediated quinolone resistance pump found in an Escherichia coli isolate from France.	fluoroquinolone antibiotic	antibiotic efflux	major facilitator superfamily (MFS) antibiotic efflux pump	28.9	2.57E-09	55.8
ECs_2538	yebQ	transporter	QepA2	ARO:3004103	QepA2 is a plasmid-mediated quinolone resistance pump found in an Escherichia coli isolate from France.	fluoroquinolone antibiotic	antibiotic efflux	major facilitator superfamily (MFS) antibiotic efflux pump	30.4	1.71E-33	129
ECs_1866	eefD	membrane transport protein	cmlA8	ARO:3004665	cmlA8 is a plasmid that confers resistance to chloramphenicol.	phenicol antibiotic	antibiotic efflux	major facilitator superfamily (MFS) antibiotic efflux pump	28.1	6.51E-23	96.7
ECs_4614	emrD	multidrug efflux system protein	cmlA8	ARO:3004665	cmlA8 is a plasmid that confers resistance to chloramphenicol.	phenicol antibiotic	antibiotic efflux	major facilitator superfamily (MFS) antibiotic	29	4.45E-26	105

								efflux pump			
ECs_4647	mdtL	multidrug efflux system protein	QepA4	ARO:3004379	A plasmid-mediated quinolone efflux pump variant described in Escherichia coli. QepA4 confers resistance to quinolone and fluoroquinolone antibiotics by expulsion from the cell. Described by Manageiro et al. 2017.	fluoroquinolone antibiotic	antibiotic efflux	major facilitator superfamily (MFS) antibiotic efflux pump	28	1.14E-13	69.3
ECs_5035	alr	alanine racemase	vanT gene in vanG cluster	ARO:3002972	Also known as vanTG, is a vanT variant found in the vanG gene cluster.	glycopeptide antibiotic	antibiotic target alteration	glycopeptide resistance gene cluster;vanT	31.1	1.33E-43	157
ECs_1685	dadX	alanine racemase	vanT gene in vanG cluster	ARO:3002972	Also known as vanTG, is a vanT variant found in the vanG gene cluster.	glycopeptide antibiotic	antibiotic target alteration	glycopeptide resistance gene cluster;vanT	28.2	1.54E-35	134
ECs_4444	wecH	O-acetyltransferase for enterobacterial common antigen	vanT gene in vanE cluster	ARO:3002971	Also known as vanTE, is a vanT variant found in the vanE gene cluster.	glycopeptide antibiotic	antibiotic target alteration	glycopeptide resistance gene cluster;vanT	23.7	1.41E-09	56.2
ECs_4425	eptB	KDO phosphoethanolamine transferase	eptB	ARO:3005047	eptB is a phosphoethanolamine transferase. It confers resistance to peptide antibiotics.	peptide antibiotic	antibiotic target alteration	pmr phosphoethanolamine transferase	81.4	0	927

ECs_4053	yhbX	EptAB family phosphoethanolamine transferase	eptB	ARO:3005047	eptB is a phosphoethanolamine transferase. It confers resistance to peptide antibiotics.	peptide antibiotic	antibiotic target alteration	pmr phosphoethanolamine transferase	27.8	5.69E-21	93.2
ECs_4884	eptC	LPS heptose I phosphoethanolamine transferase	eptB	ARO:3005047	eptB is a phosphoethanolamine transferase. It confers resistance to peptide antibiotics.	peptide antibiotic	antibiotic target alteration	pmr phosphoethanolamine transferase	22.5	6.69E-12	65.1
ECs_5096	eptA	lipid A phosphoethanolamine transferase	MCR-3.4	ARO:3004691	A plasmid-mediated MCR-3.1 variant.	peptide antibiotic	antibiotic target alteration	MCR phosphoethanolamine transferase	40.9	4.80E-144	423
ECs_3074	bcr	drug efflux system	bcr-1	ARO:3003801	Transmembrane protein which expels bicyclomycin from the cell, leading to bicyclomycin resistance. Identified in Pseudomonas aeruginosa strains responsible for outbreaks in Brazil, often appearing with blaSPM-1, another bicyclomycin resistance gene.	bicyclomycin-like antibiotic	antibiotic efflux	major facilitator superfamily (MFS) antibiotic efflux pump	38.6	1.70E-66	214
ECs_3031	ECs_3031	transport protein	bcr-1	ARO:3003801	Transmembrane protein which expels bicyclomycin from the cell, leading to bicyclomycin resistance. Identified in Pseudomonas aeruginosa strains	bicyclomycin-like antibiotic	antibiotic efflux	major facilitator superfamily (MFS) antibiotic	32.5	1.44E-08	53.5

					responsible for outbreaks in Brazil, often appearing with blaSPM-1, another bicyclomycin resistance gene.			efflux pump			
ECs_2369	ydhC	arabinose efflux transporter	bcr-1	ARO:3003801	Transmembrane protein which expels bicyclomycin from the cell, leading to bicyclomycin resistance. Identified in Pseudomonas aeruginosa strains responsible for outbreaks in Brazil, often appearing with blaSPM-1, another bicyclomycin resistance gene.	bicyclomycin-like antibiotic	antibiotic efflux	major facilitator superfamily (MFS) antibiotic efflux pump	35.8	2.48E-36	134
ECs_0922	mdfA	multidrug efflux system protein	bcr-1	ARO:3003801	Transmembrane protein which expels bicyclomycin from the cell, leading to bicyclomycin resistance. Identified in Pseudomonas aeruginosa strains responsible for outbreaks in Brazil, often appearing with blaSPM-1, another bicyclomycin resistance gene.	bicyclomycin-like antibiotic	antibiotic efflux	major facilitator superfamily (MFS) antibiotic efflux pump	29.6	5.61E-20	88.2
ECs_2302	ynfM	arabinose efflux transporter	bcr-1	ARO:3003801	Transmembrane protein which expels bicyclomycin from the cell, leading to bicyclomycin resistance. Identified in	bicyclomycin-like antibiotic	antibiotic efflux	major facilitator superfamily (MFS)	22.4	1.32E-10	59.7

					Pseudomonas aeruginosa strains responsible for outbreaks in Brazil, often appearing with blaSPM-1, another bicyclomycin resistance gene.			antibiotic efflux pump			
ECs_2104	ydeO	UV-inducible global regulator	gadW	ARO:3003838	GadW is an AraC-family regulator that promotes mdtEF expression to confer multidrug resistance. GadW inhibits GadX-dependent activation. GadW clearly represses gadX and, in situations where GadX is missing, activates gadA and gadBC.	fluoroquinolone antibiotic;macrolide antibiotic;penam	antibiotic efflux	resistance-nodulation-cell division (RND) antibiotic efflux pump	34.5	8.18E-39	132
ECs_4396	gadX	acid resistance regulon transcriptional activator	gadW	ARO:3003838	GadW is an AraC-family regulator that promotes mdtEF expression to confer multidrug resistance. GadW inhibits GadX-dependent activation. GadW clearly represses gadX and, in situations where GadX is missing, activates gadA and gadBC.	fluoroquinolone antibiotic;macrolide antibiotic;penam	antibiotic efflux	resistance-nodulation-cell division (RND) antibiotic efflux pump	31.7	2.29E-21	87.4
ECs_4395	gadW	transcriptional activator	gadW	ARO:3003838	GadW is an AraC-family regulator that promotes mdtEF expression to confer multidrug resistance. GadW inhibits GadX-dependent activation. GadW clearly	fluoroquinolone antibiotic;macrolide antibiotic;p	antibiotic efflux	resistance-nodulation-cell division (RND) antibiotic	94.6	9.02E-167	457

					represses gadX and, in situations where GadX is missing, activates gadA and gadBC.	enam		efflux pump			
ECs_5098	adiY	transcriptional activator	gadW	ARO:3003838	GadW is an AraC-family regulator that promotes mdtEF expression to confer multidrug resistance. GadW inhibits GadX-dependent activation. GadW clearly represses gadX and, in situations where GadX is missing, activates gadA and gadBC.	fluoroquinolone antibiotic;macrolide antibiotic;penam	antibiotic efflux	resistance-nodulation-cell division (RND) antibiotic efflux pump	37.5	2.90E-21	86.7
ECs_0287	ECs_0287	transcription regulator	gadW	ARO:3003838	GadW is an AraC-family regulator that promotes mdtEF expression to confer multidrug resistance. GadW inhibits GadX-dependent activation. GadW clearly represses gadX and, in situations where GadX is missing, activates gadA and gadBC.	fluoroquinolone antibiotic;macrolide antibiotic;penam	antibiotic efflux	resistance-nodulation-cell division (RND) antibiotic efflux pump	33.9	2.23E-40	136
ECs_1087	ECs_1087	AraC-family transcriptional regulator	gadW	ARO:3003838	GadW is an AraC-family regulator that promotes mdtEF expression to confer multidrug resistance. GadW inhibits GadX-dependent activation. GadW clearly represses gadX and, in situations where GadX is missing, activates	fluoroquinolone antibiotic;macrolide antibiotic;penam	antibiotic efflux	resistance-nodulation-cell division (RND) antibiotic efflux pump	36.4	5.97E-26	97.4

					gadA and gadBC.						
ECs_0598	envY	porin thermoregulatory transcriptional activator	gadW	ARO:3003838	GadW is an AraC-family regulator that promotes mdtEF expression to confer multidrug resistance. GadW inhibits GadX-dependent activation. GadW clearly represses gadX and, in situations where GadX is missing, activates gadA and gadBC.	fluoroquinolone antibiotic;macrolide antibiotic;penam	antibiotic efflux	resistance-nodulation-cell division (RND) antibiotic efflux pump	41.1	5.59E-22	88.6
ECs_2191	ECs_2191	phage regulatory protein	gadW	ARO:3003838	GadW is an AraC-family regulator that promotes mdtEF expression to confer multidrug resistance. GadW inhibits GadX-dependent activation. GadW clearly represses gadX and, in situations where GadX is missing, activates gadA and gadBC.	fluoroquinolone antibiotic;macrolide antibiotic;penam	antibiotic efflux	resistance-nodulation-cell division (RND) antibiotic efflux pump	35.5	5.22E-28	102
ECs_3454	kgtP	alpha-ketoglutarate transporter	Acinetobacter baumannii AbaF	ARO:3004573	Expression of abaF in E. coli resulted in increased resistance to fosfomycin.	phosphonic acid antibiotic	antibiotic efflux	major facilitator superfamily (MFS) antibiotic efflux pump	29	2.27E-56	189
ECs_0048	yaaU	MFS sugar transporter	Acinetobacter baumannii	ARO:3004573	Expression of abaF in E. coli resulted in increased resistance to fosfomycin.	phosphonic acid antibiotic	antibiotic efflux	major facilitator superfamily	23.6	1.32E-06	47.4

			AbaF					(MFS) antibiotic efflux pump			
ECs_4893	ECs_4893	MFS transporter	Acinetobacter baumannii AbaF	ARO:3004573	Expression of abaF in E. coli resulted in increased resistance to fosfomycin.	phosphonic acid antibiotic	antibiotic efflux	major facilitator superfamily (MFS) antibiotic efflux pump	28	6.74E-44	156
ECs_5093	proP	proline/glycine betaine transporter	Acinetobacter baumannii AbaF	ARO:3004573	Expression of abaF in E. coli resulted in increased resistance to fosfomycin.	phosphonic acid antibiotic	antibiotic efflux	major facilitator superfamily (MFS) antibiotic efflux pump	34.5	7.85E-79	250
ECs_2778	shiA	shikimate transporter	Acinetobacter baumannii AbaF	ARO:3004573	Expression of abaF in E. coli resulted in increased resistance to fosfomycin.	phosphonic acid antibiotic	antibiotic efflux	major facilitator superfamily (MFS) antibiotic efflux pump	37.1	8.68E-91	279
ECs_4403	yhjE	MFS transporter	Acinetobacter baumannii AbaF	ARO:3004573	Expression of abaF in E. coli resulted in increased resistance to fosfomycin.	phosphonic acid antibiotic	antibiotic efflux	major facilitator superfamily (MFS) antibiotic	38.6	9.00E-88	271

								efflux pump			
ECs_2076	ECs_2076	outer membrane porin protein	Klebsiella pneumoniae OmpK37	ARO:3004122	Klebsiella pneumoniae outer membrane porin protein. Is preferentially detected in porin-deficient strains. Functional characterization of this new porin revealed a narrower pore than those of porins OmpK35 and OmpK36, which did not allow penetration by certain beta-lactams. Also, when a resistant strain expresses porin OmpK37 is less susceptible to cefotaxime and cefoxitin than when it is expressing either OmpK36 or OmpK35.	carbapenem;cephalosporin;cephamycin;monobactam;penam;penem	reduced permeability to antibiotic	General Bacterial Porin with reduced permeability to beta-lactams	58.3	3.97E-152	431
ECs_0268	phoE	outer membrane phosphoporin protein E	Klebsiella pneumoniae OmpK37	ARO:3004122	Klebsiella pneumoniae outer membrane porin protein. Is preferentially detected in porin-deficient strains. Functional characterization of this new porin revealed a narrower pore than those of porins OmpK35 and OmpK36, which did not allow penetration by certain beta-lactams. Also, when	carbapenem;cephalosporin;cephamycin;monobactam;penam;penem	reduced permeability to antibiotic	General Bacterial Porin with reduced permeability to beta-lactams	60	3.12E-157	443

					a resistant strain expresses porin OmpK37 is less susceptible to cefotaxime and cefoxitin than when it is expressing either OmpK36 or OmpK35.						
ECs_3104	ompC	outer membrane porin OmpC	Klebsiella pneumoniae OmpK37	ARO:3004122	Klebsiella pneumoniae outer membrane porin protein. Is preferentially detected in porin-deficient strains. Functional characterization of this new porin revealed a narrower pore than those of porins OmpK35 and OmpK36, which did not allow penetration by certain beta-lactams. Also, when a resistant strain expresses porin OmpK37 is less susceptible to cefotaxime and cefoxitin than when it is expressing either OmpK36 or OmpK35.	carbapenem;cephalosporin;cephamycin;monobactam;penam;penem	reduced permeability to antibiotic	General Bacterial Porin with reduced permeability to beta-lactams	65.1	1.14E-168	473
ECs_1012	ompF	outer membrane porin OmpF	Klebsiella pneumoniae OmpK37	ARO:3004122	Klebsiella pneumoniae outer membrane porin protein. Is preferentially detected in porin-deficient strains. Functional characterization of this new porin revealed a	carbapenem;cephalosporin;cephamycin;monobactam;penam;penem	reduced permeability to antibiotic	General Bacterial Porin with reduced permeability to	58.7	8.11E-146	414

					narrower pore than those of porins OmpK35 and OmpK36, which did not allow penetration by certain beta-lactams. Also, when a resistant strain expresses porin OmpK37 is less susceptible to cefotaxime and cefoxitin than when it is expressing either OmpK36 or OmpK35.			beta-lactams			
ECs_5300	mdtM	multidrug efflux system protein	cmlA6	ARO:3002696	cmlA6 is a plasmid-encoded chloramphenicol exporter that is found in Pseudomonas aeruginosa.	phenicol antibiotic	antibiotic efflux	major facilitator superfamily (MFS) antibiotic efflux pump	26.2	4.14E-17	79.7
ECs_2654	sdiA	quorum-sensing transcriptional activator	sdiA	ARO:3000826	SdiA is a cell division regulator that is also a positive regulator of AcrAB only when it's expressed from a plasmid. When the sdiA gene is on the chromosome, it has no effect on expression of acrAB.	cephalosporin;disinfecting agents and antiseptics; fluoroquinolone antibiotic;glycylcycline ;penam;phenicol antibiotic;r	antibiotic efflux	resistance-nodulation-cell division (RND) antibiotic efflux pump	71.3	1.23E-124	350

						ifamycin antibiotic;tetracycline antibiotic					
ECs_0431	ddlA	D-alanine-D-alanine ligase A	vanG	ARO:3002909	VanG is a D-Ala-D-Ala ligase homolog that can synthesize D-Ala-D-Ser, an alternative substrate for peptidoglycan synthesis that reduces vancomycin binding affinity in Enterococcus faecalis.	glycopeptide antibiotic	antibiotic target alteration	Van ligase;glycopeptide resistance gene cluster	38.2	1.65E-85	260
ECs_0096	ddlB	D-alanine:D-alanine ligase	vanG	ARO:3002909	VanG is a D-Ala-D-Ala ligase homolog that can synthesize D-Ala-D-Ser, an alternative substrate for peptidoglycan synthesis that reduces vancomycin binding affinity in Enterococcus faecalis.	glycopeptide antibiotic	antibiotic target alteration	Van ligase;glycopeptide resistance gene cluster	35.4	4.72E-55	179
ECs_5062	mdtP	outer membrane factor of efflux pump	mdtP	ARO:3003550	Multidrug resistance efflux pump. Could be involved in resistance to puromycin, acriflavine and tetraphenylarsonium chloride.	disinfecting agents and antiseptics; nucleoside antibiotic	antibiotic efflux	major facilitator superfamily (MFS) antibiotic efflux pump	98	0	902
ECs_3025	mdtQ	channel/filament proteins	mdtP	ARO:3003550	Multidrug resistance efflux pump. Could be involved in	disinfecting agents and	antibiotic efflux	major facilitator	35.1	7.47E-81	257

					resistance to puromycin, acriflavine and tetraphenylarsonium chloride.	antiseptics; nucleoside antibiotic		superfamily (MFS) antibiotic efflux pump			
ECs_1865	eefC	outer membrane channel protein	adeK	ARO:3000782	AdeK is the outer membrane factor protein in the adeIJK multidrug efflux complex.	carbapenem;cephalosporin;diaminopyrimidine antibiotic;fluoroquinolone antibiotic;lincosamide antibiotic;macrolide antibiotic;penem;phenicol antibiotic;rifamycin antibiotic;tetracycline antibiotic	antibiotic efflux	resistance-nodulation-cell division (RND) antibiotic efflux pump	36.5	6.14E-81	256
ECs_0610	cusC	outer membrane component of	smeF	ARO:3003057	smeF is an outer membrane multidrug efflux protein of the smeDEF complex in	fluoroquinolone antibiotic;m	antibiotic efflux	resistance-nodulation-cell division	43.2	1.56E-104	316

		copper/silver efflux system			Stenotrophomonas maltophilia.	acrolide antibiotic;phenicol antibiotic;tetracycline antibiotic		(RND) antibiotic efflux pump			
ECs_1915	fnr	fumarate/nitrate reduction transcriptional regulator	CRP	ARO:3000518	CRP is a global regulator that represses MdtEF multidrug efflux pump expression.	fluoroquinolone antibiotic;macrolide antibiotic;penam	antibiotic efflux	resistance-nodulation-cell division (RND) antibiotic efflux pump	22.5	1.31E-08	50.8
ECs_4208	crp	cAMP-activated global transcription factor	CRP	ARO:3000518	CRP is a global regulator that represses MdtEF multidrug efflux pump expression.	fluoroquinolone antibiotic;macrolide antibiotic;penam	antibiotic efflux	resistance-nodulation-cell division (RND) antibiotic efflux pump	99.5	1.59E-151	416
ECs_3121	ECs_3121	antibiotic efflux protein	bmr	ARO:3003007	bmr is an MFS antibiotic efflux pump that confers resistance to multiple drugs including acridine dyes, fluoroquinolone antibiotics, chloramphenicol, and puromycin.	disinfecting agents and antiseptics; fluoroquinolone antibiotic;nucleoside antibiotic;p	antibiotic efflux	major facilitator superfamily (MFS) antibiotic efflux pump	26.8	2.71E-13	67.8

						henicol antibiotic disinfecting agents and antiseptics;					
ECs_5316	lgoT	L-galactonate transporter	bmr	ARO:3003007	bmr is an MFS antibiotic efflux pump that confers resistance to multiple drugs including acridine dyes, fluoroquinolone antibiotics, chloramphenicol, and puromycin.	fluoroquinolone antibiotic;nucleoside antibiotic;phenicol antibiotic disinfecting agents and antiseptics;	antibiotic efflux	major facilitator superfamily (MFS) antibiotic efflux pump	28.6	5.97E-09	54.7
ECs_3975	exuT	hexuronate transporter	bmr	ARO:3003007	bmr is an MFS antibiotic efflux pump that confers resistance to multiple drugs including acridine dyes, fluoroquinolone antibiotics, chloramphenicol, and puromycin.	fluoroquinolone antibiotic;nucleoside antibiotic;phenicol antibiotic disinfecting agents and antiseptics;	antibiotic efflux	major facilitator superfamily (MFS) antibiotic efflux pump	21.5	9.69E-09	53.9
ECs_1443	mdtH	multidrug resistance protein MdtH	blt	ARO:3003006	blt is an MFS efflux pump that confers resistance to multiple drugs such as rhodamine and acridine dyes, and	fluoroquinol	antibiotic efflux	major facilitator superfamily (MFS)	20.3	1.48E-07	50.1

					fluoroquinolone antibiotics.	one antibiotic		antibiotic efflux pump			
ECs_0481	yajR	transporter	emeA	ARO:3003551	A multidrug efflux pump from Enterococcus faecalis. There exist efflux activity of several antimicrobial agents such as DAPI, Hoechst 33342 and acriflavine. Efflux of DAPI via EmeA was strongly inhibited by reserpine.	disinfecting agents and antiseptics	antibiotic efflux	multidrug and toxic compound extrusion (MATE) transporter	23.9	5.02E-14	70.5
ECs_0532	fsr	fosmidomycin resistance protein	rosA	ARO:3003048	rosA is part of an efflux pump/potassium antiporter system (RosAB) in Yersinia that confers resistance to cationic antimicrobial peptides such as polymyxin B.	peptide antibiotic	antibiotic efflux	major facilitator superfamily (MFS) antibiotic efflux pump	73.2	7.43E-186	519
ECs_0531	ybaL	inner membrane NAD(P)-binding transporter	rosB	ARO:3003049	rosB is part of an efflux pump/potassium antiporter system (RosAB) in Yersinia that confers resistance to cationic antimicrobial peptides such as polymyxin B.	peptide antibiotic	antibiotic efflux	major facilitator superfamily (MFS) antibiotic efflux pump	74.6	7.32E-263	727
ECs_0050	kefC	glutathione-regulated potassium-efflux system	rosB	ARO:3003049	rosB is part of an efflux pump/potassium antiporter system (RosAB) in Yersinia that confers resistance to cationic	peptide antibiotic	antibiotic efflux	major facilitator superfamily (MFS)	28.6	7.41E-38	145

		protein			antimicrobial peptides such as polymyxin B.			antibiotic efflux pump			
ECs_4201	kefB	glutathione-regulated potassium-efflux system protein	rosB	ARO:3003049	rosB is part of an efflux pump/potassium antiporter system (RosAB) in Yersinia that confers resistance to cationic antimicrobial peptides such as polymyxin B.	peptide antibiotic	antibiotic efflux	major facilitator superfamily (MFS) antibiotic efflux pump	28.6	6.05E-43	159
ECs_0088	ftsI	cell division protein FtsI	mecD	ARO:3004185	mecD is a PBP2a variant identified on a genomic resistance island in Macrococcus caseolyticus. MecD confers resistance to methicillin and other beta-lactam antibiotics through the production of an alternative low-affinity PBP. First described by Schwendener et al. 2017 and identified from canine and bovine sources.	carbapenem;cephalosporin;cephamycin;monobactam;penam	antibiotic target replacement	methicillin resistant PBP2	24.5	3.99E-23	100
ECs_0673	mrdA	penicillin-binding protein 2	mecD	ARO:3004185	mecD is a PBP2a variant identified on a genomic resistance island in Macrococcus caseolyticus. MecD confers resistance to methicillin and other beta-lactam antibiotics through the production of an	carbapenem;cephalosporin;cephamycin;monobactam;penam	antibiotic target replacement	methicillin resistant PBP2	27.9	5.65E-40	152

					alternative low-affinity PBP. First described by Schwendener et al. 2017 and identified from canine and bovine sources.						
ECs_0402	mhpA	3-(3-hydroxyphenyl)propionate hydroxylase	iri	ARO:3002884	iri is a monooxygenase that confers resistance to rifampin found in Rhodococcus hoagii.	rifamycin antibiotic	antibiotic inactivation	rifampin monooxygenase	32.2	2.51E-32	127
ECs_0700	ubiF	2-octaprenyl-3-methyl-6-methoxy-1%2C4-benzoquinol oxygenase	iri	ARO:3002884	iri is a monooxygenase that confers resistance to rifampin found in Rhodococcus hoagii.	rifamycin antibiotic	antibiotic inactivation	rifampin monooxygenase	25.2	7.44E-10	57.4
ECs_3777	ubiI	2-octaprenylphenol hydroxylase	iri	ARO:3002884	iri is a monooxygenase that confers resistance to rifampin found in Rhodococcus hoagii.	rifamycin antibiotic	antibiotic inactivation	rifampin monooxygenase	24.5	4.84E-06	45.4
ECs_0663	flc	fluoride efflux channel	CrcB	ARO:3005049	CrcB is part of the Camphor Resistance Protein Family. It confers resistance to aminoglycoside antibiotics.	aminoglycoside antibiotic	antibiotic efflux	multidrug and toxic compound extrusion (MATE) transporter	31.2	2.60E-13	59.7
ECs_2002	ldhA	fermentative D-lactate dehydrogenase	vanH gene in vanD cluster	ARO:3002944	Also known as vanHD, is a vanH variant in the vanD gene cluster.	glycopeptide antibiotic	antibiotic target alteration	glycopeptide resistance gene cluster;vanH	32.4	5.57E-57	184

ECs_1410	ghrA	glyoxylate/hydroxypyruvate reductase A	vanH gene in vanD cluster	ARO:3002944	Also known as vanHD, is a vanH variant in the vanD gene cluster.	glycopeptide antibiotic	antibiotic target alteration	glycopeptide resistance gene cluster;vanH	30.3	1.07E-12	64.7
ECs_3204	pdxB	erythronate-4-phosphate dehydrogenase	vanH gene in vanD cluster	ARO:3002944	Also known as vanHD, is a vanH variant in the vanD gene cluster.	glycopeptide antibiotic	antibiotic target alteration	glycopeptide resistance gene cluster;vanH	29.3	3.36E-22	93.2
ECs_3784	serA	D-3-phosphoglycerate dehydrogenase	vanH gene in vanD cluster	ARO:3002944	Also known as vanHD, is a vanH variant in the vanD gene cluster.	glycopeptide antibiotic	antibiotic target alteration	glycopeptide resistance gene cluster;vanH	33	9.35E-25	100
ECs_4438	ghrB	glyoxylate/hydroxypyruvate reductase B	vanH gene in vanD cluster	ARO:3002944	Also known as vanHD, is a vanH variant in the vanD gene cluster.	glycopeptide antibiotic	antibiotic target alteration	glycopeptide resistance gene cluster;vanH	30.9	4.69E-24	97.4
ECs_0866	ybhN	inner membrane protein	Brucella suis mprF	ARO:3003772	MprF is a integral membrane protein that modifies the negatively-charged phosphatidylglycerol on the membrane surface. This confers resistance to cationic peptides that disrupt the cell membrane, including defensins.	peptide antibiotic	antibiotic target alteration	defensin resistant mprF	27.2	2.36E-13	67.8
ECs_2829	ugd	UDP-glucose 6-dehydrogena	ugd	ARO:3003577	PmrE is required for the synthesis and transfer of	peptide antibiotic	antibiotic target	pmr phosphoethan	99.2	1.83E-278	752

		se			4-amino-4-deoxy-L-arabinose (Ara4N) to Lipid A, which allows gram-negative bacteria to resist the antimicrobial activity of cationic antimicrobial peptides and antibiotics such as polymyxin.		alteration	olamine transferase			
ECs_3543	ygaY	transporter	fexB	ARO:3007019	FexB is a plasmid-encoded exporter gene which confers resistance to florfenicol. Originally identified in Enterococcus sp. isolated from swine by Liu et al. 2012.	phenicol antibiotic	antibiotic efflux	major facilitator superfamily (MFS) antibiotic efflux pump	22.5	6.86E-08	51.2
ECs_2532	rlmA	23S rRNA m(1)G745 methyltransferase	chrB	ARO:3001302	ChrB is a methyltransferase found in Streptomyces bikiniensis and confers resistance to chalcomycin, mycinamicin, and tylosin. Specifically, this enzyme adds a methyl group to guanosine 748 (E. coli numbering). chrB is found in the chalcomycin biosynthetic cluster and is responsible for self-resistance in S. bikiniensis.	lincosamide antibiotic;macrolide antibiotic	antibiotic target alteration	non-erm 23S ribosomal RNA methyltransferase (G748)	37	3.67E-41	140
ECs_4153	fmt	L-methionyl-t	arnA	ARO:3002985	arnA modifies lipid A with	peptide	antibiotic	pmr	29.4	2.34E-2	115

		RNA(fMet) N-formyltransferase			4-amino-4-deoxy-L-arabinose (Ara4N) which allows gram-negative bacteria to resist the antimicrobial activity of cationic antimicrobial peptides and antibiotics such as polymyxin. arnA is found in E. coli and P. aeruginosa.	antibiotic	target alteration	phosphoethanolamine transferase		9	
ECs_1734	purU	formyltetrahydrofolate hydrolase	arnA	ARO:3002985	arnA modifies lipid A with 4-amino-4-deoxy-L-arabinose (Ara4N) which allows gram-negative bacteria to resist the antimicrobial activity of cationic antimicrobial peptides and antibiotics such as polymyxin. arnA is found in E. coli and P. aeruginosa.	peptide antibiotic	antibiotic target alteration	pmr phosphoethanolamine transferase	28.9	2.02E-06	45.8
ECs_3362	purN	phosphoribosylglycinamide formyltransferase 1	arnA	ARO:3002985	arnA modifies lipid A with 4-amino-4-deoxy-L-arabinose (Ara4N) which allows gram-negative bacteria to resist the antimicrobial activity of cationic antimicrobial peptides and antibiotics such as polymyxin. arnA is found in E. coli and P. aeruginosa.	peptide antibiotic	antibiotic target alteration	pmr phosphoethanolamine transferase	26.6	1.00E-09	54.7

ECs_3143	arnA	UDP-4-amino-4-deoxy-L-arabinose formyltransferase	arnA	ARO:3002985	arnA modifies lipid A with 4-amino-4-deoxy-L-arabinose (Ara4N) which allows gram-negative bacteria to resist the antimicrobial activity of cationic antimicrobial peptides and antibiotics such as polymyxin. arnA is found in E. coli and P. aeruginosa.	peptide antibiotic	antibiotic target alteration	pmr phosphoethanolamine transferase	69.5	0	931
ECs_5106	dcuR	two-component regulatory system response regulator DcuR	adeR	ARO:3000553	AdeR is a positive regulator of AdeABC efflux system. AdeR inactivation leads to susceptibility to aminoglycoside antibiotics.	glycylcycline;tetracycline antibiotic	antibiotic efflux	resistance-nodulation-cell division (RND) antibiotic efflux pump	21.9	3.16E-07	47
ECs_0416	ECs_0416	regulatory protein	norB	ARO:3000421	NorB is a multidrug efflux pump in Staphylococcus aureus that confers resistance to fluoroquinolones and other structurally unrelated antibiotics like tetracycline. It shares 30% similarity with NorB, and is a structural homolog of Blt of Bacillus subtilis. It is regulated by mgrA, also known as NorR.	fluoroquinolone antibiotic	antibiotic efflux	major facilitator superfamily (MFS) antibiotic efflux pump	23	6.49E-09	54.7

ECs_3206	yfcJ	arabinose efflux transporter	norB	ARO:3000421	NorB is a multidrug efflux pump in Staphylococcus aureus that confers resistance to fluoroquinolones and other structurally unrelated antibiotics like tetracycline. It shares 30% similarity with NorB, and is a structural homolog of Blt of Bacillus subtilis. It is regulated by mgrA, also known as NorR.	fluoroquinolone antibiotic	antibiotic efflux	major facilitator superfamily (MFS) antibiotic efflux pump	28	4.61E-06	45.4
ECs_4005	garP	(D)-galactarate transporter	Acinetobacter baumannii AbaQ	ARO:3004574	AbaQ is an MFS transporter mainly involved in the extrusion of quinolone-type drugs in A. baumannii.	fluoroquinolone antibiotic	antibiotic efflux	major facilitator superfamily (MFS) antibiotic efflux pump	24.9	5.51E-23	97.8
ECs_3649	gudP	D-glucarate transporter	Acinetobacter baumannii AbaQ	ARO:3004574	AbaQ is an MFS transporter mainly involved in the extrusion of quinolone-type drugs in A. baumannii.	fluoroquinolone antibiotic	antibiotic efflux	major facilitator superfamily (MFS) antibiotic efflux pump	24	7.63E-26	106
ECs_1614	ECs_1614	multidrug resistance protein	Escherichia coli emrE	ARO:3004039	Member of the small MDR (multidrug resistance) family of transporters; in Escherichia	macrolide antibiotic	antibiotic efflux	small multidrug resistance	98.2	1.83E-79	226

					coli this protein provides resistance against a number of positively charged compounds including ethidium bromide and erythromycin; proton-dependent secondary transporter which exchanges protons for compound translocation.			(SMR) antibiotic efflux pump			
ECs_2305	mdtI	multidrug efflux system transporter	abeS	ARO:3000768	AbeS in an efflux pump of the SMR family of transporters found in Acinetobacter baumannii.	aminocoumarin antibiotic;macrolide antibiotic	antibiotic efflux	small multidrug resistance (SMR) antibiotic efflux pump	38.1	3.57E-18	70.9
ECs_5129	sugE	multidrug efflux system protein	qacL	ARO:3005098	A subunit of the qac multidrug efflux pump in Vibrio cholerae.	disinfecting agents and antiseptics	antibiotic efflux	small multidrug resistance (SMR) antibiotic efflux pump	38.6	6.54E-15	62.4
ECs_2306	mdtJ	multidrug efflux system transporter	qacE	ARO:3005009	QacE is a resistance gene conferring resistance to antiseptics.	disinfecting agents and antiseptics	antibiotic efflux	major facilitator superfamily (MFS) antibiotic efflux pump	30.6	4.10E-11	53.1

ECs_2883	mdtC_1	multidrug efflux system subunit B	mdtB	ARO:3000793	MdtB is a transporter that forms a heteromultimer complex with MdtC to form a multidrug transporter. MdtBC is part of the MdtABC-TolC efflux complex.	aminocoumarin antibiotic	antibiotic efflux	resistance-nodulation-cell division (RND) antibiotic efflux pump	99.7	0	1903
ECs_0613	cusA	copper/silver efflux system	mdtB	ARO:3000793	MdtB is a transporter that forms a heteromultimer complex with MdtC to form a multidrug transporter. MdtBC is part of the MdtABC-TolC efflux complex.	aminocoumarin antibiotic	antibiotic efflux	resistance-nodulation-cell division (RND) antibiotic efflux pump	22.8	1.27E-57	213
ECs_2884	mdtC_2	multidrug efflux system subunit B	mdtB	ARO:3000793	MdtB is a transporter that forms a heteromultimer complex with MdtC to form a multidrug transporter. MdtBC is part of the MdtABC-TolC efflux complex.	aminocoumarin antibiotic	antibiotic efflux	resistance-nodulation-cell division (RND) antibiotic efflux pump	50.2	7.23806171157e-321	911
ECs_3778	ubiH	2-octaprenyl-6-methoxyphenol hydroxylase	tet(49)	ARO:3004582	A tetracycline inactivating enzyme. A flavoenzyme capable of degrading tetracycline antibiotics.	tetracycline antibiotic	antibiotic inactivation	tetracycline inactivation enzyme	24.5	1.38E-07	50.1
ECs_4910	rpoB	RNA polymerase beta subunit	Bifidobacterium adolescent is rpoB	ARO:3004480	Bifidobacterium are antibiotic resistant probiotics are prescribed to upkeep the population beneficial bacteria	peptide antibiotic;rifamycin antibiotic	antibiotic target alteration;antibiotic	rifamycin-resistant beta-subunit of RNA	44.6	0	1060

			mutants conferring resistance to rifampicin		in the gut microbiome. However, horizontal gene transfer among gut microbes could create harmful antibiotic-resistant pathogenic bacteria, such as Mycobacterium tuberculosis. Lokesh et al. analyzed Bifidobacterium antitubercular drug resistance and mutations in rpoB. They found that B. animalis, B. longum and B. adolescentis showed considerable resistance to pyrazinamide, isoniazid, and streptomycin, while B. adolescentis had mutations both in the rifampicin (RIF) pocket and in regions outside the pockets, and also showed considerable resistance to RIF.		target replacement	polymerase (rpoB)			
ECs_5045	soxR	redox-sensitive transcriptional activator SoxR	Pseudomonas aeruginosa soxR	ARO:3004107	SoxR is a redox-sensitive transcriptional activator that induces expression of a small regulon that includes the RND efflux pump-encoding operon mexGHI-opmD. SoxR was shown to be activated by pyocyanin.	cephalosporin;disinfecting agents and antiseptics; fluoroquinolone antibiotic;g	antibiotic efflux;antibiotic target alteration	ATP-binding cassette (ABC) antibiotic efflux pump;major facilitator	62.4	2.37E-62	186

						lycylcycline;penam;phenicol antibiotic;rifamycin antibiotic;tetracycline antibiotic		superfamily (MFS) antibiotic efflux pump;resistance-nodulation-cell division (RND) antibiotic efflux pump			
ECs_4068	murA	UDP-N-acetylglucosamine 1-carboxyvinyltransferase	Chlamydia trachomatis intrinsic murA conferring resistance to fosfomycin	ARO:3003785	Chlamydia murA confers intrinsic resistance to fosfomycin. The presence of an aspartic acid residue in place of the critical cysteine at position 119 that enables fosfomycin binding is believed to be responsible for this intrinsic resistance.	phosphonic acid antibiotic	antibiotic target alteration	antibiotic-resistant murA transferase	33.6	2.75E-59	197
ECs_5131	ampC	beta-lactamase	EC-15	ARO:3006876	EC-15 is a EC beta-lactamase.	cephalosporin	antibiotic inactivation	EC beta-lactamase	98.1	2.82E-281	758
ECs_0625	entF	enterobactin synthase multienzyme complex	almE	ARO:3007433	almE is a glycyltransferase found in Vibrio cholerae that transfers glycine to the carrier protein almF. almE is part of the almEFG	peptide antibiotic	antibiotic target alteration	alm glycyltransferase;polymyxin	29.8	1.93E-45	170

		component			polymyxin resistance operon.			resistance operon			
ECs_4331	ECs_4331	surfactin synthetase	almE	ARO:3007433	almE is a glycyltransferase found in Vibrio cholerae that transfers glycine to the carrier protein almF. almE is part of the almEFG polymyxin resistance operon.	peptide antibiotic	antibiotic target alteration	alm glycyltransferase;polymyxin resistance operon	27.5	1.70E-07	50.4
ECs_3027	ECs_3027	salicylate hydroxylase	tet(54)	ARO:3004590	A tetracycline inactivating enzyme. A flavoenzyme capable of degrading tetracycline antibiotics.	tetracycline antibiotic	antibiotic inactivation	tetracycline inactivation enzyme	22.7	2.65E-09	55.5
ECs_2372	mdtK	multidrug efflux system transporter	hmrM	ARO:3003953	hmrM is a multidrug efflux pump belonging to the MATE family and functions as a Na+/drug antiporter.	disinfecting agents and antiseptics; fluoroquinolone antibiotic	antibiotic efflux	multidrug and toxic compound extrusion (MATE) transporter	47.1	1.21E-141	411
ECs_5027	dinF	DNA damage inducible protein DinF	abeM	ARO:3000753	AbeM is an multidrug efflux pump found in Acinetobacter baumannii.	disinfecting agents and antiseptics; fluoroquinolone antibiotic	antibiotic efflux	multidrug and toxic compound extrusion (MATE) transporter	24	1.25E-10	60.1
ECs_2409	ppsA	phosphoenolpyruvate	rphB	ARO:3003992	rphB is a rifampin phosphotransferase protein found	rifamycin antibiotic	antibiotic inactivation	rifampin phosphotrans	36.3	1.19E-47	179

		synthase			in Paenibacillus sp. LC231, a strain of Paenibacillus isolated from Lechuguilla Cave, NM, USA. Confers resistance to rifamycin antibiotics, specifically rifampin, through rifampin inactivation. Described by Pawlowski et al. 2016.			ferase			
ECs_1051	rlmI	23S rRNA m(5)C1962 methyltransferase	LlmA 23S ribosomal RNA methyltransferase	ARO:3003982	Lincosamide (Clindamycin) resistant (putative) ribosomal methyltransferase related to the RlmK 23S rRNA methyltransferase COG family. Detected in Paenibacillus sp. LC231, an isolated strain of Paenibacillus found in Lechuguilla Cave, NM, USA. Described by Pawlowski et al. 2016.	lincosamide antibiotic	antibiotic target alteration	Llm 23S ribosomal RNA methyltransferase	42	2.45E-13	67
ECs_1032	rlmL	23S rRNA m(2)G2445 and m(7)G2069 methyltransferases	LlmA 23S ribosomal RNA methyltransferase	ARO:3003982	Lincosamide (Clindamycin) resistant (putative) ribosomal methyltransferase related to the RlmK 23S rRNA methyltransferase COG family. Detected in Paenibacillus sp. LC231, an isolated strain of Paenibacillus found in Lechuguilla Cave, NM,	lincosamide antibiotic	antibiotic target alteration	Llm 23S ribosomal RNA methyltransferase	32.1	3.00E-09	55.8

					USA. Described by Pawlowski et al. 2016.						
ECs_0458	yajC	SecYEG protein translocase auxiliary subunit	YajC	ARO:3005040	YajC interacts with the AcrAB-TolC efflux pump in a way that in uncharacterized but is shown to grant increased fitness in the presence of linezolid, rifampicin, and vancomycin.	cephalosporin;disinfecting agents and antiseptics;fluoroquinolone antibiotic;glycopeptide antibiotic;glycylcycline;oxazolidinone antibiotic;penam;phenicol antibiotic;rifamycin antibiotic;tetracycline antibiotic	antibiotic efflux	resistance-nodulation-cell division (RND) antibiotic efflux pump	46.9	2.07E-28	97.1
ECs_0059	lptD	LPS assembly OM complex LptDE beta-barrel	LptD	ARO:3005059	LptD is involved in LPS transport in a ABC Transporter efflux system. It confers resistance to rifamycin, aminocoumarin, and	aminocoumarin antibiotic;peptide	antibiotic efflux	ATP-binding cassette (ABC) antibiotic	82.1	0	1345

		component			peptide antibiotics.	antibiotic;rifamycin antibiotic		efflux pump			
ECs_0051	folA	dihydrofolate reductase	dfrA3	ARO:3003105	dfrA3 is an integron-encoded dihydrofolate reductase found in Escherichia coli.	diaminopyrimidine antibiotic	antibiotic target replacement	trimethoprim resistant dihydrofolate reductase dfr	51.3	1.74E-52	163
ECs_0056	rsmA	ribosomal RNA small subunit methyltransferase A	Erm(34)	ARO:3000600	ErmD confers MLSb phenotype.	lincosamide antibiotic;macrolide antibiotic;streptogramin A antibiotic;streptogramin B antibiotic;streptogramin antibiotic	antibiotic target alteration	Erm 23S ribosomal RNA methyltransferase	24.2	1.62E-16	74.7
ECs_0630	entS	enterobactin exporter	mefF	ARO:3007044	mefF is an mef efflux pump protein.	macrolide antibiotic	antibiotic efflux	major facilitator superfamily (MFS) antibiotic efflux pump	22.9	8.90E-08	50.8

ECs_2360	gloA	lactoylglutathione lyase	FosM2	ARO:3007098	FosM2 is a thiol transferase discovered in human gut microflora that leads to the resistance of fosfomycin.	phosphonic acid antibiotic	antibiotic inactivation	fosfomycin thiol transferase	26.4	8.27E-07	42.7
ECs_1041	ompA	outer membrane protein OmpA	OmpA	ARO:3005044	OmpA is a porin that confers resistance to beta-lactam antibiotics.	peptide antibiotic	reduced permeability to antibiotic	General Bacterial Porin with reduced permeability to peptide antibiotics	85.4	1.03E-217	596
ECs_0776	pal	peptidoglycan-associated outer membrane lipoprotein	OmpA	ARO:3005044	OmpA is a porin that confers resistance to beta-lactam antibiotics.	peptide antibiotic	reduced permeability to antibiotic	General Bacterial Porin with reduced permeability to peptide antibiotics	34.9	2.52E-06	43.5
ECs_3566	norV	anaerobic nitric oxide reductase flavorubredoxin	tet(37)	ARO:3002871	tet(37) is a chromosome-encoded oxidoreductase isolated from an uncultured bacterium that confers resistance to tetracycline.	tetracycline antibiotic	antibiotic inactivation	tetracycline inactivation enzyme	31.5	1.69E-07	46.6
ECs_0028	ribF	in bifunctional riboflavin kinase/FAD	mreA	ARO:3007050	mreA is a macrolide efflux pump.	macrolide antibiotic	antibiotic efflux	major facilitator superfamily	36.2	8.71E-51	167

		synthetase						(MFS) antibiotic efflux pump			
ECs_0029	ileS	isoleucyl-tRNA synthetase	Staphylococcus aureus mupA conferring resistance to mupirocin	ARO:3000521	An alternative isoleucyl-tRNA synthetase conferring resistance to mupirocin.	mupirocin-like antibiotic	antibiotic target alteration	antibiotic-resistant isoleucyl-tRNA synthetase (ileS)	29.6	3.26E-99	331
ECs_5235	valS	valyl-tRNA synthetase	Staphylococcus aureus mupA conferring resistance to mupirocin	ARO:3000521	An alternative isoleucyl-tRNA synthetase conferring resistance to mupirocin.	mupirocin-like antibiotic	antibiotic target alteration	antibiotic-resistant isoleucyl-tRNA synthetase (ileS)	22.5	5.67E-48	182
ECs_0680	leuS	leucyl-tRNA synthetase	Staphylococcus aureus mupA conferring resistance to mupirocin	ARO:3000521	An alternative isoleucyl-tRNA synthetase conferring resistance to mupirocin.	mupirocin-like antibiotic	antibiotic target alteration	antibiotic-resistant isoleucyl-tRNA synthetase (ileS)	26.5	2.24E-11	64.7
ECs_2920	metG	methionyl-tRNA synthetase	Bifidobacterium	ARO:3003730	Bifidobacteria have an intrinsically resistant form of	mupirocin-like	antibiotic target	antibiotic-resistant	45.3	4.43E-08	53.5

			bifidum ileS conferring resistance to mupirocin		ileS (isoleucyl-tRNA synthetase) that confers resistance to mupirocin.	antibiotic	alteration	isoleucyl-tRNA synthetase (ileS)			
ECs_3379	rlmN	50S rRNA methyltransferase	clbB	ARO:3002815	clbB is a plasmid-encoded cfr gene found in Bacillus brevis.	lincosamide antibiotic;oxazolidinone antibiotic;phenicol antibiotic;pleuromutilin antibiotic;streptogramin A antibiotic;streptogramin antibiotic	antibiotic target alteration	Cfr 23S ribosomal RNA methyltransferase	33.7	8.47E-52	173
ECs_0921	ybjG	undecaprenyl pyrophosphate phosphatase	bcrC	ARO:3003250	The bcrC gene product (BcrC) is an undecaprenyl pyrophosphate phosphatase originally isolated from Bacillus subtilis. When overexpressed it can confer resistance to bacitracin.	peptide antibiotic	antibiotic target alteration	undecaprenyl pyrophosphate related proteins	30.2	5.95E-19	78.2
ECs_3142	arnC	undecaprenyl	PmrF	ARO:3003578	PmrF is required for the	peptide	antibiotic	pmr	99.4	7.47E-2	626

		phosphate-L-Ara4FN transferase			synthesis and transfer of 4-amino-4-deoxy-L-arabinose (Ara4N) to Lipid A, which allows gram-negative bacteria to resist the antimicrobial activity of cationic antimicrobial peptides and antibiotics such as polymyxin. pmrF corresponds to 1 locus in Pseudomonas aeruginosa PAO1 and 1 locus in Pseudomonas aeruginosa LESB58.	antibiotic	target alteration	phosphoethanolamine transferase		31	
ECs_4056	folP	7%2C8-dihydropteroate synthase	sul4	ARO:3004361	sul4 is a dihydropteroate synthase gene and mobile sulfonamide resistance gene shown to confer resistance when expressed in E. coli.	sulfonamide antibiotic	antibiotic target replacement	sulfonamide resistant sul	40.1	1.35E-63	199
ECs_3145	arnT	4-amino-4-deoxy-L-arabinose transferase	ArnT	ARO:3005053	ArnT is involved in Cell Wall Biosynthesis, specifically 4-amino-4-deoxy-L-arabinose (Ara4N). It confers resistance to peptide antibiotics.	peptide antibiotic	antibiotic target alteration	pmr phosphoethanolamine transferase	63.9	1.86E-253	702
ECs_2092	ddpX	D-ala-D-ala dipeptidase	vanX gene in vanM cluster	ARO:3002953	Also known as vanXM, is a vanX variant found in the vanM gene cluster.	glycopeptide antibiotic	antibiotic target alteration	glycopeptide resistance gene cluster;vanX	36.2	4.65E-26	96.7
ECs_3546	mprA	transcription	emrR	ARO:3000516	EmrR is a negative regulator for	fluoroquinol	antibiotic	major	100	2.34E-1	347

		al repressor			the EmrAB-TolC multidrug efflux pump in E. coli. Mutations lead to EmrAB-TolC overexpression.	one antibiotic	efflux	facilitator superfamily (MFS) antibiotic efflux pump		25	
gene-papX	papX	BAA31779.1	emrR	ARO:3000516	EmrR is a negative regulator for the EmrAB-TolC multidrug efflux pump in E. coli. Mutations lead to EmrAB-TolC overexpression.	fluoroquinolone antibiotic	antibiotic efflux	major facilitator superfamily (MFS) antibiotic efflux pump	44.1	2.19E-09	48.9
ECs_3156	rbn	ribonuclease Z	PNGM-1	ARO:3004571	PNGM-1 is a subclass B3 metallo-beta-lactamase first identified from a deep-sea sediment meta-genome. PNGM-1 is shown to reduce susceptibility to extended- and broad-spectrum cephalosporins, carbapenems, and penicillins. PNGM-1 is a novel metallo-beta-lactamase which predates the current antibiotic era.	carbapenem;cephalosporin;penam	antibiotic inactivation	PNGM beta-lactamase	22.7	8.29E-08	50.1
ECs_3553	csrA	pleiotropic regulatory protein for carbon source	rsmA	ARO:3005069	rsmA is a gene that regulates virulence of Pseudomonas aeruginosa. However, its negative effect on MexEF-OprN	diaminopyrimidine antibiotic;fluoroquinolo	antibiotic efflux	resistance-nodulation-cell division (RND)	85.2	1.08E-31	101

		metabolism			overexpression has been noted to confer resistance to various antibiotics. It's Escherichia coli homolog is csrA.	ne antibiotic;phenicol antibiotic		antibiotic efflux pump			
ECs_1010	ycbL	hypothetical protein	PEDO-2	ARO:3003714	PEDO-2 is a subclass B3 metallo-beta lactamase isolated from Pedobacter borealis exhibiting carbapenem resistance.	carbapenem	antibiotic inactivation	subclass B3 PEDO beta-lactamase	36	6.22E-14	66.2
ECs_3940	bacA	undecaprenyl pyrophosphate phosphatase	bacA	ARO:3002986	The bacA gene product (BacA) recycles undecaprenyl pyrophosphate during cell wall biosynthesis which confers resistance to bacitracin.	peptide antibiotic	antibiotic target alteration	undecaprenyl pyrophosphate related proteins	100	2.51E-193	527
ECs_3301	yfeW	penicillin binding protein PBP4B	PDC-130	ARO:3006507	PDC-130 is a PDC beta-lactamase.	carbapenem;cephalosporin;monobactam	antibiotic inactivation	PDC beta-lactamase	26.6	3.38E-11	61.6
ECs_4073	mlaE	ABC transporter permease	RanB	ARO:3005090	RanB is the determinant of antibiotic resistance within the RanARanB ABC-type efflux system. It confers resistance to aminoglycoside antibiotics.	aminoglycoside antibiotic	antibiotic efflux	ATP-binding cassette (ABC) antibiotic efflux pump	28.6	4.09E-23	92
ECs_1317	eptA	membrane-associated metal-dependent hydrolase	MCR-9.1	ARO:3004684	A mobilized and plasmid-mediated colistin resistance gene and phosphoethanolamine transferase identified from a Salmonella	peptide antibiotic	antibiotic target alteration	MCR phosphoethanolamine transferase	59.4	8.68E-239	664

enterica isolate.

The CARD database is a comprehensive database of antibiotic resistance genes, containing a wide range of antibiotic resistance-related reference genes from a variety of organisms, genomes, and plasmids, which can be used to guide the study of environmental, human, and animal bacterial resistance groups and antibiotic resistance mechanisms. Higher scores represent higher values and higher homology of the two sequences.

Table S3. Virulence factors of Pathogenic Bacteria (VFDB) annotation.

gene_id	gene_name	description	vf_id	vf_name	vf_function	database	species	vf_category	related_genes	product	identity	evalue	score
ECs_3317	eutE	aldehyde oxidoreductase	VFG019346 (gb|WP_003729668)	Lap (VF0444)	Promotes bacterial adhesion to intestinal cells	setA	Listeria monocytogenes HCC23	Adherence	lap	Listeria adhesion protein Lap	33.4	1.92E-43	163
ECs_3315	eutG	ethanolamine utilization protein EutG	VFG019346 (gb|WP_003729668)	Lap (VF0444)	Promotes bacterial adhesion to intestinal cells	setA	Listeria monocytogenes HCC23	Adherence	lap	Listeria adhesion protein Lap	32.5	2.03E-42	158
ECs_4466	yiaY	L-threonine dehydrogenase	VFG019346 (gb|WP_003729668)	Lap (VF0444)	Promotes bacterial adhesion to intestinal cells	setA	Listeria monocytogenes HCC23	Adherence	lap	Listeria adhesion protein Lap	33.8	7.51E-49	176
ECs_3659	fucO	L-1%2C2-propanediol oxidoreductase	VFG019346 (gb|WP_003729668)	Lap (VF0444)	Promotes bacterial adhesion to intestinal cells	setA	Listeria monocytogenes HCC23	Adherence	lap	Listeria adhesion protein Lap	35.9	2.43E-57	200

ECs_1741	adhE	acetaldehyde dehydrogenase	VFG031937 (gb|WP_003733217)	Lap (VF0444)	Promotes bacterial adhesion to intestinal cells	setA	Listeria monocytogenes SLCC5850	Adherence	lap	Listeria adhesion protein Lap	48.7	1.98E-273	781
ECs_3316	eutJ	ethanolamine utilization protein	VFG050314 (gb|YP_001086190.1)	TFP (VF1334)	Essential for twitching motility and natural competence, and contribute to host cell adherence	setA	Acinetobacter baumannii ATCC 17978	Adherence	pilM	pilus assembly protein PilM	25.3	2.09E-06	48.1
ECs_4509	coaD	pantetheine-phosphate adenylyltransferase	VFG000320 (gb|WP_001169234)	LPS (VF0056)	H. pylori LPS have much lower immunobiological activities than enterobacterial LPS, thus may prolong H. pylori infection for longer; mediates a lectin-like interaction with laminin, the binding may disrupt epithelial cell-basement membrane interactions contributing to the disruption of gastric mucosal integrity and the development of gastric leakiness associated with the bacterium	setA	Helicobacter pylori 26695	Immune modulation	kdtB	lipopolysaccharide core biosynthesis protein	49.7	2.53E-54	168
ECs_4508	waaA	3-deoxy-D-manno-octulosonic-acid transfera	VFG013312 (gb|WP_012055150)	LOS (VF0044)	Major immunogen; LOS phosphorylcholine (ChoP) may influence invasion via interaction with PAF receptor and stimulates of inflammatory signals; LPS phase	setA	Haemophilus influenzae PittGG	Immune modulation	kdtA	3-deoxy-d-manno-octulosonic-acid transferas	51.2	6.59E-145	419

		se			variation is characterized by the spontaneous loss and gain of oligosaccharide structures present in the outer core. the phase variable expression of LPS biosynthesis genes promotes evasion of antigen-specific host immune defences and allow colonization of different host microenvironments					e			
ECs_4754	rhtB	homoserine/homoserine lactone efflux protein	VFG001235 (gb|NP_249108)	Type IV pili (VF0082)	Attaches to host cells, but not to mucin, causing a twitching motility that allows the bacteria to move along the cell surface; biofilm formation	setA	Pseudomonas aeruginosa PAO1	Adherence	chpE	probable chemotaxis protein	32	4.64E-08	51.2
ECs_0382	yahN	neutral amino-acid efflux system	VFG001235 (gb|NP_249108)	Type IV pili (VF0082)	Attaches to host cells, but not to mucin, causing a twitching motility that allows the bacteria to move along the cell surface; biofilm formation	setA	Pseudomonas aeruginosa PAO1	Adherence	chpE	probable chemotaxis protein	31.5	8.77E-11	58.9
ECs_4502	waaR	lipopolysaccharide 1%2C2-glucosyltransferase WaaR	VFG013261 (gb|WP_011271948)	LOS (VF0044)	Major immunogen; LOS phosphorylcholine (ChoP) may influence invasion via interaction with PAF receptor and stimulates of inflammatory signals; LPS phase variation is characterized by the spontaneous loss and gain of	setA	Haemophilus influenzae 86-028NP	Immune modulation	lgtC	glycosyltransferase	24.4	1.32E-20	90.5

					oligosaccharide structures present in the outer core. the phase variable expression of LPS biosynthesis genes promotes evasion of antigen-specific host immune defences and allow colonization of different host microenvironments							
ECs_4504	waaO	UDP-D-galactose:(glucosyl)lipopolysaccharide-alpha-1%2C3-D-galactosyltransferase	VFG013261 (gb|WP_011271948)	LOS (VF0044)	Major immunogen; LOS phosphorylcholine (ChoP) may influence invasion via interaction with PAF receptor and stimulates of inflammatory signals; LPS phase variation is characterized by the spontaneous loss and gain of oligosaccharide structures present in the outer core. the phase variable expression of LPS biosynthesis genes promotes evasion of antigen-specific host immune defences and allow colonization of different host microenvironments	setA	Haemophilus influenzae 86-028NP	Immune modulation	lgtC	glycosyltransferase	29.7	3.04E-23	97.8
ECs_4501	waaD	lipopolysaccharide 1%2C2-N-acetylglucosamine	VFG005935 (gb|YP_001197936)	Capsule (VF0144)	Resistant to complement deposition and masks cell wall-associated complement from being recognized by the complement receptors on phagocytes	setA	Streptococcus suis 05ZYH33	Immune modulation	SSU05_0570	glycosyltransferase	21.6	2.84E-11	64.3

		transferase WaaD											
ECs_4507	waaQ	lipopolysaccharide core biosynthesis protein WaaQ	VFG011773 (gb|WP_011732192)	LOS (VF0326)	LOS diversity is important for the ability to colonize a wide variety of hosts and intestinal niches; the ability to generate variation at high frequency, the molecular mimicry evident in LOS structure support a role in the avoidance of host defences; the similarity of LOS structures to host gangliosides and the subsequent ability to generate crossreacting antibodies forms the pathological basis for the association of preceding C. jejuni infection with Guillain-Barre syndrome	setA	Campylobacter fetus subsp. fetus 82-40	Immune modulation	rfaQ	putative lipopolysaccharide heptosyltransferase III	29.3	4.51E-34	128
ECs_4499	waaC	heptosyltransferase I	VFG013152 (gb|WP_012341246)	LOS (VF0044)	Major immunogen; LOS phosphorylcholine (ChoP) may influence invasion via interaction with PAF receptor and stimulates of inflammatory signals; LPS phase variation is characterized by the spontaneous loss and gain of oligosaccharide structures present in the outer core. the phase variable expression of LPS biosynthesis genes	setA	Haemophilus somnus 2336	Immune modulation	opsX/rfaC	heptosyltransferase I	56	1.17E-117	342

ECs_4506	waaG	glucosyltransferase I	VFG000139 (gb|NP_253697)	LPS (VF0085)	promotes evasion of antigen-specific host immune defences and allow colonization of different host microenvironments Mediates biological effects including resistance to serum killing and phagocytosis; the binding to normal CFTR (cystic fibrosis transmembrane conductance regulator) and invasion of host cells may make a contribution to virulence in the human eye; internalization by binding to normal CFTR protein expressed by airway epithelial cells followed by desquamation of bacteria-laden epithelial cells, constitutes a host defense mechanism. If this mechanism fails to function properly, abnormally high bacterial carriage would promote the establishment of chronic bacterial infection	setA	Pseudomonas aeruginosa PAO1	Immune modulation	waaG	B-band O-antigen polymerase	50.8	7.69E-135	389
ECs_2849	wcaL	glycosyltransferase	VFG050281 (gb|WP_001909517)	VPS (VF0615)	Major component of the V. cholerae biofilm matrix; essential for development of three-dimensional biofilm structures	setA	Vibrio cholerae O1 biovar El Tor str. N16961	Biofilm	vpsI	exopolysaccharide biosynthesis glycosyltr	24.7	8.34E-15	75.1

										ansferase VpsI			
ECs_4505	waaP	kinase that phosphorylates core heptose of lipopolysaccharide	VFG000140 (gb|NP_253696)	LPS (VF0085)	Mediates biological effects including resistance to serum killing and phagocytosis; the binding to normal CFTR (cystic fibrosis transmembrane conductance regulator) and invasion of host cells may make a contribution to virulence in the human eye; internalization by binding to normal CFTR protein expressed by airway epithelial cells followed by desquamation of bacteria-laden epithelial cells, constitutes a host defense mechanism. If this mechanism fails to function properly, abnormally high bacterial carriage would promote the establishment of chronic bacterial infection	setA	Pseudomonas aeruginosa PAO1	Immune modulation	waaP	UDP-glucose:(heptosyl) LPS alpha 1,3-glucosyltransferase WaaG	56.4	1.50E-103	301
ECs_4698	hdfR	flhDC operon transcriptional repressor	VFG049114 (gb|WP_012737527)	Allantion utilization (VF0572)	Providing a nitrogen source to increase virulence in K. pneumoniae at certain sites of infection	setA	Klebsiella pneumoniae subsp. pneumoniae NTUH-K2044	Nutritional/Metabolic factor	allS	DNA-binding transcriptional activator AllS	47.1	6.63E-11	61.6
ECs_2133	yneJ	transcrip	VFG049114	Allantion	Providing a nitrogen source to	setA	Klebsiella	Nutritiona	allS	DNA-bindin	31	6.96E-	52.8

		tional regulator	(gb|WP_012737527)	utilization (VF0572)	increase virulence in K. pneumoniae at certain sites of infection		pneumoniae subsp. pneumoniae NTUH-K2044	l/Metabolic factor		g transcriptional activator AllS		08	
ECs_3403	hcaR	hca operon transcriptional regulator	VFG049114 (gb|WP_012737527)	Allantion utilization (VF0572)	Providing a nitrogen source to increase virulence in K. pneumoniae at certain sites of infection	setA	Klebsiella pneumoniae subsp. pneumoniae NTUH-K2044	Nutritional/Metabolic factor	allS	DNA-binding transcriptional activator AllS	25.2	1.44E-10	60.8
ECs_0565	allS	allD operon transcriptional activator	VFG049114 (gb|WP_012737527)	Allantion utilization (VF0572)	Providing a nitrogen source to increase virulence in K. pneumoniae at certain sites of infection	setA	Klebsiella pneumoniae subsp. pneumoniae NTUH-K2044	Nutritional/Metabolic factor	allS	DNA-binding transcriptional activator AllS	73.8	1.78E-167	467
ECs_4401	yhjC	LysR family transcriptional regulator	VFG049114 (gb|WP_012737527)	Allantion utilization (VF0572)	Providing a nitrogen source to increase virulence in K. pneumoniae at certain sites of infection	setA	Klebsiella pneumoniae subsp. pneumoniae NTUH-K2044	Nutritional/Metabolic factor	allS	DNA-binding transcriptional activator AllS	30.1	3.09E-16	77.8
ECs_1910	pgrR	murein peptide degradati	VFG049114 (gb|WP_012737527)	Allantion utilization	Providing a nitrogen source to increase virulence in K. pneumoniae at certain sites of infection	setA	Klebsiella pneumoniae subsp.	Nutritional/Metabolic factor	allS	DNA-binding transcript	26.5	1.19E-09	58.2

		on regulator		(VF0572)			pneumoniae NTUH-K2044			ional activator			
ECs_3443	yfiE	transcriptional regulator	VFG049114 (gb|WP_012737527)	Allantion utilization (VF0572)	Providing a nitrogen source to increase virulence in K. pneumoniae at certain sites of infection	setA	Klebsiella pneumoniae subsp. pneumoniae NTUH-K2044	Nutritional/Metabolic factor	allS	AllS DNA-binding transcriptional activator	24.2	3.01E-07	50.8
ECs_3668	gcvA	glycine cleavage system transcriptional activator	VFG049114 (gb|WP_012737527)	Allantion utilization (VF0572)	Providing a nitrogen source to increase virulence in K. pneumoniae at certain sites of infection	setA	Klebsiella pneumoniae subsp. pneumoniae NTUH-K2044	Nutritional/Metabolic factor	allS	AllS DNA-binding transcriptional activator	41	1.32E-08	55.1
ECs_2817	yeeY	LysR family transcriptional regulator	VFG049114 (gb|WP_012737527)	Allantion utilization (VF0572)	Providing a nitrogen source to increase virulence in K. pneumoniae at certain sites of infection	setA	Klebsiella pneumoniae subsp. pneumoniae NTUH-K2044	Nutritional/Metabolic factor	allS	AllS DNA-binding transcriptional activator	26.7	7.65E-34	125
ECs_3173	lrhA	transcriptional repressor	VFG049114 (gb|WP_012737527)	Allantion utilization (VF0572)	Providing a nitrogen source to increase virulence in K. pneumoniae at certain sites of infection	setA	Klebsiella pneumoniae subsp. pneumoniae NTUH-K2044	Nutritional/Metabolic factor	allS	AllS DNA-binding transcriptional activator	38.4	4.70E-06	47.4

ECs_2508	dmlR	transcriptional activator of dmlA	VFG049114 (gb|WP_012737527)	Allantion utilization (VF0572)	Providing a nitrogen source to increase virulence in K. pneumoniae at certain sites of infection	setA	Klebsiella pneumoniae subsp. pneumoniae NTUH-K2044	Nutritional/Metabolic factor	allS	AllS DNA-binding transcriptional activator	31.7	1.80E-08	54.7
ECs_2368	ydhB	LysR family transcriptional regulator	VFG049114 (gb|WP_012737527)	Allantion utilization (VF0572)	Providing a nitrogen source to increase virulence in K. pneumoniae at certain sites of infection	setA	Klebsiella pneumoniae subsp. pneumoniae NTUH-K2044	Nutritional/Metabolic factor	allS	AllS DNA-binding transcriptional activator	31.8	2.65E-44	153
ECs_3987	yhaJ	LysR family transcriptional regulator	VFG049114 (gb|WP_012737527)	Allantion utilization (VF0572)	Providing a nitrogen source to increase virulence in K. pneumoniae at certain sites of infection	setA	Klebsiella pneumoniae subsp. pneumoniae NTUH-K2044	Nutritional/Metabolic factor	allS	AllS DNA-binding transcriptional activator	27.6	4.03E-25	102
ECs_2301	ynfL	LysR family transcriptional regulator	VFG049114 (gb|WP_012737527)	Allantion utilization (VF0572)	Providing a nitrogen source to increase virulence in K. pneumoniae at certain sites of infection	setA	Klebsiella pneumoniae subsp. pneumoniae NTUH-K2044	Nutritional/Metabolic factor	allS	AllS DNA-binding transcriptional activator	28	7.29E-12	64.7
ECs_0796	ybhD	transcrip	VFG049114	Allantion	Providing a nitrogen source to	setA	Klebsiella	Nutritiona	allS	AllS DNA-bindin	25.6	5.69E-	59.3

		tional regulator	(gb|WP_012737527)	utilization (VF0572)	increase virulence in K. pneumoniae at certain sites of infection		pneumoniae subsp. pneumoniae NTUH-K2044	l/Metabolic factor		g transcriptional activator AllS		10	
ECs_4707	ilvY	transcriptional activator	VFG049114 (gb|WP_012737527)	Allantion utilization (VF0572)	Providing a nitrogen source to increase virulence in K. pneumoniae at certain sites of infection	setA	Klebsiella pneumoniae subsp. pneumoniae NTUH-K2044	Nutritional/Metabolic factor	allS	DNA-binding transcriptional activator AllS	37.5	1.33E-06	48.9
ECs_3943	ttdR	transcriptional activator	VFG049114 (gb|WP_012737527)	Allantion utilization (VF0572)	Providing a nitrogen source to increase virulence in K. pneumoniae at certain sites of infection	setA	Klebsiella pneumoniae subsp. pneumoniae NTUH-K2044	Nutritional/Metabolic factor	allS	DNA-binding transcriptional activator AllS	25.7	1.75E-09	57.8
ECs_0309	ECs_0309	transcriptional regulator	VFG049114 (gb|WP_012737527)	Allantion utilization (VF0572)	Providing a nitrogen source to increase virulence in K. pneumoniae at certain sites of infection	setA	Klebsiella pneumoniae subsp. pneumoniae NTUH-K2044	Nutritional/Metabolic factor	allS	DNA-binding transcriptional activator AllS	23.6	9.86E-08	52.4
ECs_0305	yagP	LysR family transcrip	VFG049114 (gb|WP_012737527)	Allantion utilization on	Providing a nitrogen source to increase virulence in K. pneumoniae at certain sites of infection	setA	Klebsiella pneumoniae subsp.	Nutritional/Metabolic factor	allS	DNA-binding transcript	25.7	8.83E-10	58.5

		tional regulator		(VF0572)			pneumoniae NTUH-K2044			ional activator AllS			
ECs_1847	cysB	N-acetylserine-responsive cysteine regulon transcriptional activator	VFG006042 (gb|WP_001222601)	Capsule (VF0274)	Inhibits the binding of the activated complement factor C3b to the surface of S. agalactiae, preventing the activation of the alternative complement pathway and inhibits complement-mediated opsonophagocytosis	setA	Streptococcus agalactiae A909	Immune modulation	SAK_RS06335	LysR family transcriptional regulator	26.6	1.68E-14	72.8
ECs_3122	yfaH	transcriptional regulator	VFG006042 (gb|WP_001222601)	Capsule (VF0274)	Inhibits the binding of the activated complement factor C3b to the surface of S. agalactiae, preventing the activation of the alternative complement pathway and inhibits complement-mediated opsonophagocytosis	setB	Streptococcus agalactiae A909	Immune modulation	SAK_RS06335	LysR family transcriptional regulator	30.9	1.72E-06	48.5
ECs_4890	oxyR	oxidative and nitrosative stress transcriptional regulator	VFG006042 (gb|WP_001222601)	Capsule (VF0274)	Inhibits the binding of the activated complement factor C3b to the surface of S. agalactiae, preventing the activation of the alternative complement pathway and inhibits complement-mediated opsonophagocytosis	setA	Streptococcus agalactiae A909	Immune modulation	SAK_RS06335	LysR family transcriptional regulator	22.6	2.62E-11	63.2
ECs_3998	tdcA	tdc	VFG006042	Capsule	Inhibits the binding of the activated	setB	Streptococ	Immune	SAK_RS0633	LysR family	22.7	7.74E-	55.8

		operon transcriptional activator	(gb|WP_001222601)	(VF0274)	complement factor C3b to the surface of S. agalactiae, preventing the activation of the alternative complement pathway and inhibits complement-mediated opsonophagocytosis		cus agalactiae A909	modulation	5	transcriptional regulator		09	
ECs_3049	yeiE	transcriptional regulator	VFG006042 (gb|WP_001222601)	Capsule (VF0274)	Inhibits the binding of the activated complement factor C3b to the surface of S. agalactiae, preventing the activation of the alternative complement pathway and inhibits complement-mediated opsonophagocytosis	setA	Streptococcus agalactiae A909	Immune modulation	SAK_RS06335	LysR family transcriptional regulator	19.7	6.20E-13	67.8
ECs_4693	rbsB	D-ribose transporter subunit RbsB	VFG049313 (gb|WP_002910453)	T6SS-III (VF0784)	-	setB	Klebsiella pneumoniae subsp. pneumoniae 1084	Effector delivery system	A79E_RS10215	sugar ABC transporter substrate-binding protein	28.8	1.36E-29	114
ECs_3042	mglB	methyl-galactoside transporter subunit	VFG049313 (gb|WP_002910453)	T6SS-III (VF0784)	-	setB	Klebsiella pneumoniae subsp. pneumoniae 1084	Effector delivery system	A79E_RS10215	sugar ABC transporter substrate-binding protein	29	1.09E-31	120
ECs_5071	ECs_5071	carbohydr	VFG049313	T6SS-III	-	setB	Klebsiella	Effector	A79E_RS102	sugar ABC	28.1	1.20E-	92.8

		ate binding protein	(gb|WP_002910453)	(VF0784)			pneumoniae subsp. pneumoniae 1084	delivery system	15	transporter substrate-binding protein		21	
ECs_5205	ytfQ	galactofuranose ABC transporter periplasmic binding protein	VFG049313 (gb|WP_002910453)	T6SS-III (VF0784)	-	setB	Klebsiella pneumoniae subsp. pneumoniae 1084	Effector delivery system	A79E_RS10215	sugar ABC transporter substrate-binding protein	30.1	2.63E-22	94.7
ECs_3414	yphF	sugar ABC transporter periplasmic binding protein	VFG049313 (gb|WP_002910453)	T6SS-III (VF0784)	-	setB	Klebsiella pneumoniae subsp. pneumoniae 1084	Effector delivery system	A79E_RS10215	sugar ABC transporter substrate-binding protein	23.9	3.52E-09	57
ECs_4691	rbsA	D-ribose ABC transporter ATPase	VFG007913 (gb|WP_011894249)	PDIM (VF0309)	Play a mainly structural role in providing a stable base for the insertion of other lipid and also play a role as a fluidity modifier, whose function could be to modulate cell wall	setA	Mycobacterium gilvum PYR-GCK	Immune modulation	ddrA	ATP-binding cassette domain-containing protein	31.9	1.11E-27	113

					viscosity;PDIM is crucial for infection by masking pathogen-associated molecular patterns (PAMP) of the cell wall from the innate immune system								
ECs_4302	livG	branched-chain amino acid ABC transporter ATPase	VFG007913 (gb|WP_011894249)	PDIM (VF0309)	Play a mainly structural role in providing a stable base for the insertion of other lipid and also play a role as a fluidity modifier, whose function could be to modulate cell wall viscosity;PDIM is crucial for infection by masking pathogen-associated molecular patterns (PAMP) of the cell wall from the innate immune system	setA	Mycobacterium gilvum PYR-GCK	Immune modulation	ddrA	ATP-binding cassette domain-containing protein	31.9	4.38E-30	114
ECs_4080	lptB	lipopolysaccharide export ABC transporter ATPase	VFG007913 (gb|WP_011894249)	PDIM (VF0309)	Play a mainly structural role in providing a stable base for the insertion of other lipid and also play a role as a fluidity modifier, whose function could be to modulate cell wall viscosity;PDIM is crucial for infection by masking pathogen-associated molecular patterns (PAMP) of the cell wall from the innate immune system	setA	Mycobacterium gilvum PYR-GCK	Immune modulation	ddrA	ATP-binding cassette domain-containing protein	31.2	1.49E-31	118
ECs_0872	ybhF	ABC	VFG007913	PDIM	Play a mainly structural role in	setA	Mycobacter	Immune	ddrA	ATP-bindin	35.1	7.96E-	153

		transporter ATPase	(gb|WP_011894249)	(VF0309)	providing a stable base for the insertion of other lipid and also play a role as a fluidity modifier, whose function could be to modulate cell wall viscosity;PDIM is crucial for infection by masking pathogen-associated molecular patterns (PAMP) of the cell wall from the innate immune system		ium gilvum PYR-GCK	modulation		g cassette domain-containing protein		42	
ECs_4450	xylG	D-xylose ABC transporter dual domain ATPase	VFG007913 (gb|WP_011894249)	PDIM (VF0309)	Play a mainly structural role in providing a stable base for the insertion of other lipid and also play a role as a fluidity modifier, whose function could be to modulate cell wall viscosity;PDIM is crucial for infection by masking pathogen-associated molecular patterns (PAMP) of the cell wall from the innate immune system	setA	Mycobacterium gilvum PYR-GCK	Immune modulation	ddrA	ATP-binding cassette domain-containing protein	29.1	1.49E-19	89.7
ECs_4359	rbbA	ribosome-associated ATPase: ATP-binding protein/ATP-bindin	VFG007914 (gb|WP_011780329)	PDIM (VF0309)	Play a mainly structural role in providing a stable base for the insertion of other lipid and also play a role as a fluidity modifier, whose function could be to modulate cell wall viscosity;PDIM is crucial for infection by masking	setA	Mycobacterium vanbaalenii PYR-1	Immune modulation	ddrA	ATP-binding cassette domain-containing protein	33.9	4.41E-35	136

		g membrane protein			pathogen-associated molecular patterns (PAMP) of the cell wall from the innate immune system								
ECs_0375	ECs_0375	ATP-binding component of sugar ABC transporter	VFG007914 (gb|WP_011780329)	PDIM (VF0309)	Play a mainly structural role in providing a stable base for the insertion of other lipid and also play a role as a fluidity modifier, whose function could be to modulate cell wall viscosity;PDIM is crucial for infection by masking pathogen-associated molecular patterns (PAMP) of the cell wall from the innate immune system	setA	Mycobacterium vanbaaleni i PYR-1	Immune modulation	ddrA	ATP-binding cassette domain-containing protein	27.5	4.64E-18	84.3
ECs_5080	phnK	carbon-phosphorus lyase complex subunit	VFG008680 (gb|WP_015309619)	PDIM (VF0309)	Play a mainly structural role in providing a stable base for the insertion of other lipid and also play a role as a fluidity modifier, whose function could be to modulate cell wall viscosity;PDIM is crucial for infection by masking pathogen-associated molecular patterns (PAMP) of the cell wall from the innate immune system	setA	Mycobacterium smegmatis JS623	Immune modulation	ddrA	ATP-binding cassette domain-containing protein	29	2.02E-23	96.7
ECs_4696	hsrA	multidrug or homocyste	VFG036957 (gb|WP_010951322)	FarAB (VF0450)	Mediates the resistance to antimicrobial long-chain fatty acids	setA	Neisseria gonorrhoeae FA 1090	Antimicrobial activity/C	farB	fatty acid efflux system	30	4.88E-38	145

		ine efflux system						ompetitive advantage		protein FarB			
ECs_2885	iceT	transporter	VFG036960 (gb|WP_003703449)	FarAB (VF0450)	Mediates the resistance to antimicrobial long-chain fatty acids	setA	Neisseria gonorrhoeae NCCP11945	Antimicrobial activity/Competitive advantage	farB	fatty acid efflux system protein FarB	29	1.47E-33	132
ECs_3548	emrY	multidrug efflux system	VFG036955 (gb|WP_002236670)	FarAB (VF0450)	Mediates the resistance to antimicrobial long-chain fatty acids	setA	Neisseria meningitidis Z2491	Antimicrobial activity/Competitive advantage	farB	fatty acid efflux system protein FarB	57.5	8.53E-208	585
ECs_3246	emrY	multidrug efflux system	VFG036955 (gb|WP_002236670)	FarAB (VF0450)	Mediates the resistance to antimicrobial long-chain fatty acids	setA	Neisseria meningitidis Z2491	Antimicrobial activity/Competitive advantage	farB	fatty acid efflux system protein FarB	51.5	4.72E-180	515
ECs_4695	rbsR	transcriptional repressor	VFG002197 (gb|WP_002387027)	BopD (VF0362)	Homologous to a sugar-binding transcriptional regulator involved in biofilm production; The actual role is unknown, but the association of enhanced biofilm formation in the presence of glucose and the possible involvement of a sugar-binding transcriptional regulator suggest a	setA	Enterococcus faecalis V583	Biofilm	bopD	sugar-binding transcriptional regulator, LacI family	25.7	1.33E-27	110

					linkage to increased biofilm production in E. faecalis in the presence of specific carbohydrates								
ECs_1899	ycjW	LacI family transcriptional repressor	VFG002197 (gb|WP_002387027)	BopD (VF0362)	Homologous to a sugar-binding transcriptional regulator involved in biofilm production; The actual role is unknown, but the association of enhanced biofilm formation in the presence of glucose and the possible involvement of a sugar-binding transcriptional regulator suggest a linkage to increased biofilm production in E. faecalis in the presence of specific carbohydrates	setA	Enterococcus faecalis V583	Biofilm	bopD	sugar-binding transcriptional regulator, LacI family	26.4	2.81E-31	120
ECs_3043	galS	transcriptional repressor	VFG002197 (gb|WP_002387027)	BopD (VF0362)	Homologous to a sugar-binding transcriptional regulator involved in biofilm production; The actual role is unknown, but the association of enhanced biofilm formation in the presence of glucose and the possible involvement of a sugar-binding transcriptional regulator suggest a linkage to increased biofilm production in E. faecalis in the presence of specific carbohydrates	setA	Enterococcus faecalis V583	Biofilm	bopD	sugar-binding transcriptional regulator, LacI family	26	7.34E-18	83.2
ECs_0084	fruR	DNA-bindi	VFG002197	BopD	Homologous to a sugar-binding	setA	Enterococc	Biofilm	bopD	sugar-bind	26.3	1.85E-	52

		ng transcriptional dual regulator FruR	(gb|WP_002387027)	(VF0362)	transcriptional regulator involved in biofilm production; The actual role is unknown, but the association of enhanced biofilm formation in the presence of glucose and the possible involvement of a sugar-binding transcriptional regulator suggest a linkage to increased biofilm production in E. faecalis in the presence of specific carbohydrates		us faecalis V583			ing transcriptional regulator, LacI family		07	
ECs_0398	lacI	lactose operon repressor	VFG002197 (gb|WP_002387027)	BopD (VF0362)	Homologous to a sugar-binding transcriptional regulator involved in biofilm production; The actual role is unknown, but the association of enhanced biofilm formation in the presence of glucose and the possible involvement of a sugar-binding transcriptional regulator suggest a linkage to increased biofilm production in E. faecalis in the presence of specific carbohydrates	setA	Enterococcus faecalis V583	Biofilm	bopD	sugar-binding transcriptional regulator, LacI family	26.4	3.01E-17	81.6
ECs_4287	gntR	d-gluconate inducible gluconate regulon	VFG002197 (gb|WP_002387027)	BopD (VF0362)	Homologous to a sugar-binding transcriptional regulator involved in biofilm production; The actual role is unknown, but the association of enhanced biofilm formation in the	setA	Enterococcus faecalis V583	Biofilm	bopD	sugar-binding transcriptional regulator,	24.2	3.07E-20	89.7

		transcriptional repressor			presence of glucose and the possible involvement of a sugar-binding transcriptional regulator suggest a linkage to increased biofilm production in E. faecalis in the presence of specific carbohydrates					LacI family			
ECs_2367	purR	transcriptional repressor	VFG002197 (gb|WP_002387027)	BopD (VF0362)	Homologous to a sugar-binding transcriptional regulator involved in biofilm production; The actual role is unknown, but the association of enhanced biofilm formation in the presence of glucose and the possible involvement of a sugar-binding transcriptional regulator suggest a linkage to increased biofilm production in E. faecalis in the presence of specific carbohydrates	setA	Enterococcus faecalis V583	Biofilm	bopD	sugar-binding transcriptional regulator, LacI family	28.5	1.58E-26	107
ECs_5218	treR	trehalose 6-phosphate-inducible trehalose regulon transcriptional repressor	VFG002197 (gb|WP_002387027)	BopD (VF0362)	Homologous to a sugar-binding transcriptional regulator involved in biofilm production; The actual role is unknown, but the association of enhanced biofilm formation in the presence of glucose and the possible involvement of a sugar-binding transcriptional regulator suggest a linkage to increased biofilm	setA	Enterococcus faecalis V583	Biofilm	bopD	sugar-binding transcriptional regulator, LacI family	23.7	2.30E-17	81.3

					production in E. faecalis in the presence of specific carbohydrates								
ECs_2328	malI	transcriptional repressor	VFG002197 (gb|WP_002387027)	BopD (VF0362)	Homologous to a sugar-binding transcriptional regulator involved in biofilm production; The actual role is unknown, but the association of enhanced biofilm formation in the presence of glucose and the possible involvement of a sugar-binding transcriptional regulator suggest a linkage to increased biofilm production in E. faecalis in the presence of specific carbohydrates	setA	Enterococcus faecalis V583	Biofilm	bopD	sugar-binding transcriptional regulator, LacI family	25.8	8.12E-17	80.1
ECs_3570	ascG	asc operon transcriptional repressor	VFG002197 (gb|WP_002387027)	BopD (VF0362)	Homologous to a sugar-binding transcriptional regulator involved in biofilm production; The actual role is unknown, but the association of enhanced biofilm formation in the presence of glucose and the possible involvement of a sugar-binding transcriptional regulator suggest a linkage to increased biofilm production in E. faecalis in the presence of specific carbohydrates	setA	Enterococcus faecalis V583	Biofilm	bopD	sugar-binding transcriptional regulator, LacI family	25.1	1.60E-19	87.8
ECs_4861	cytR	transcriptional	VFG002197 (gb|WP_00	BopD (VF0362)	Homologous to a sugar-binding transcriptional regulator involved in	setA	Enterococcus faecalis	Biofilm	bopD	sugar-binding	29.1	1.73E-29	115

		regulator	2387027)		biofilm production; The actual role is unknown, but the association of enhanced biofilm formation in the presence of glucose and the possible involvement of a sugar-binding transcriptional regulator suggest a linkage to increased biofilm production in E. faecalis in the presence of specific carbohydrates		V583			transcriptional regulator, LacI family			
ECs_3694	galR	galactose-inducible d-galactose regulon transcriptional repressor	VFG002197 (gb|WP_002387027)	BopD (VF0362)	Homologous to a sugar-binding transcriptional regulator involved in biofilm production; The actual role is unknown, but the association of enhanced biofilm formation in the presence of glucose and the possible involvement of a sugar-binding transcriptional regulator suggest a linkage to increased biofilm production in E. faecalis in the presence of specific carbohydrates	setA	Enterococcus faecalis V583	Biofilm	bopD	sugar-binding transcriptional regulator, LacI family	24	4.24E-15	75.1
ECs_3957	ebgR	transcriptional repressor	VFG002197 (gb|WP_002387027)	BopD (VF0362)	Homologous to a sugar-binding transcriptional regulator involved in biofilm production; The actual role is unknown, but the association of enhanced biofilm formation in the presence of glucose and the possible	setA	Enterococcus faecalis V583	Biofilm	bopD	sugar-binding transcriptional regulator, LacI family	22	1.22E-10	61.6

					involvement of a sugar-binding transcriptional regulator suggest a linkage to increased biofilm production in E. faecalis in the presence of specific carbohydrates								
ECs_3244	ECs_3244	sucrose operon repressor	VFG045668 (gb|WP_002358859)	BopD (VF0362)	Homologous to a sugar-binding transcriptional regulator involved in biofilm production; The actual role is unknown, but the association of enhanced biofilm formation in the presence of glucose and the possible involvement of a sugar-binding transcriptional regulator suggest a linkage to increased biofilm production in E. faecalis in the presence of specific carbohydrates	setB	Enterococcus faecalis D32	Biofilm	bopD	sugar-binding transcriptional regulator, LacI family	20.7	5.28E-06	47.4
ECs_0199	metQ	DL-methionine transporter subunit	VFG045346 (gb|WP_011149697)	IlpA (VF0513)	Dual functions as an adhesin and an immunostimulant	setA	Vibrio vulnificus YJ016	Adherence	IlpA	immunogenic lipoprotein A	67.2	2.31E-128	365
ECs_4595	nlpA	cytoplasmic membrane lipoprotein-28	VFG045346 (gb|WP_011149697)	IlpA (VF0513)	Dual functions as an adhesin and an immunostimulant	setA	Vibrio vulnificus YJ016	Adherence	IlpA	immunogenic lipoprotein A	54.3	2.36E-97	286

ECs_2138	marA	multiple antibiotic resistance transcriptional regulator	VFG042570 (gb|AAX78184)	Locus for diffuse adherence (lda), afimbrial adhesin (VF1148)	-	setA	Escherichia coli	Adherence	AAX78184	LdaA	40.6	5.85E-18	77.4
ECs_5044	soxS	transcriptional regulator	VFG042570 (gb|AAX78184)	Locus for diffuse adherence (lda), afimbrial adhesin (VF1148)	-	setA	Escherichia coli	Adherence	AAX78184	LdaA	39.4	7.52E-16	70.9
ECs_0068	araC	ara regulon transcriptional activator	VFG042570 (gb|AAX78184)	Locus for diffuse adherence (lda), afimbrial adhesin (VF1148)	-	setA	Escherichia coli	Adherence	AAX78184	LdaA	34.4	7.72E-11	61.6
ECs_5354	rob	transcriptional regulator	VFG042570 (gb|AAX78184)	Locus for diffuse adherence (lda),	-	setA	Escherichia coli	Adherence	AAX78184	LdaA	27.6	7.43E-20	87.4

ECs_0337	ykgA	transcriptional regulator	VFG042570 (gb|AAX78184)	afimbrial adhesin (VF1148) Locus for diffuse adherence (lda), afimbrial adhesin (VF1148)	-	setA	Escherichia coli	Adherence	AAX78184	LdaA	30.3	2.13E-31	118
ECs_4452	xylR	xylose divergent operon transcriptional activator	VFG042526 (gb|AAC28314)	Adhesive factor/rabbit 1 (AF/R1) pili (VF1146)	-	setA	Escherichia coli str. RDEC-1	Adherence	afrR	transcriptional activator AfrR	37.3	4.42E-07	50.8
ECs_2499	yeaM	transcriptional regulator	VFG019777 (gb|WP_012724142)	Type IV pili (VF0082)	Attaches to host cells, but not to mucin, causing a twitching motility that allows the bacteria to move along the cell surface; biofilm formation	setA	Pseudomonas fluorescens SBW25	Adherence	chpD	probable transcriptional regulator	37.3	5.05E-07	49.7
ECs_3102	ada	bifunctional transcriptional activator/DNA	VFG019777 (gb|WP_012724142)	Type IV pili (VF0082)	Attaches to host cells, but not to mucin, causing a twitching motility that allows the bacteria to move along the cell surface; biofilm formation	setA	Pseudomonas fluorescens SBW25	Adherence	chpD	probable transcriptional regulator	29.9	2.03E-06	48.5

		repair enzyme Ada										
ECs_0399	ECs_0399	transcriptional regulator	VFG019776 (gb|WP_003117898)	Type IV pili (VF0082)	Attaches to host cells, but not to mucin, causing a twitching motility that allows the bacteria to move along the cell surface; biofilm formation	setA	Pseudomonas aeruginosa LESB58	Adherence	chpD	probable transcriptional regulator	35.4	1.88E-09	57.4
ECs_4621	yidL	AraC family transcriptional regulator	VFG019776 (gb|WP_003117898)	Type IV pili (VF0082)	Attaches to host cells, but not to mucin, causing a twitching motility that allows the bacteria to move along the cell surface; biofilm formation	setA	Pseudomonas aeruginosa LESB58	Adherence	chpD	probable transcriptional regulator	33.3	1.20E-09	57.8
ECs_4116	aaeR	transcriptional regulator	VFG044083 (gb|NP_250948)	Pyoverdine (VF0094)	Effective at acquiring iron from transferrin and lactoferrin; cytotoxic due to its ability to stimulating the production of reactive oxygen species	setA	Pseudomonas aeruginosa PAO1	Nutritional/Metabolic factor	ptxR	transcriptional regulator PtxR	28.2	1.30E-35	130
ECs_0018	nhaR	transcriptional activator	VFG044083 (gb|NP_250948)	Pyoverdine (VF0094)	Effective at acquiring iron from transferrin and lactoferrin; cytotoxic due to its ability to stimulating the production of reactive oxygen species	setA	Pseudomonas aeruginosa PAO1	Nutritional/Metabolic factor	ptxR	transcriptional regulator PtxR	37.8	4.38E-06	47.4
ECs_0333	ycaN	transcriptional regulator	VFG044083 (gb|NP_250948)	Pyoverdine (VF0094)	Effective at acquiring iron from transferrin and lactoferrin; cytotoxic due to its ability to stimulating the production of reactive	setA	Pseudomonas aeruginosa PAO1	Nutritional/Metabolic factor	ptxR	transcriptional regulator PtxR	29.6	7.34E-40	141

					oxygen species								
ECs_0204	yafC	LysR family transcriptional regulator	VFG044083 (gb|NP_250948)	Pyoverdine (VF0094)	Effective at acquiring iron from transferrin and lactoferrin; cytotoxic due to its ability to stimulating the production of reactive oxygen species	setA	Pseudomonas aeruginosa PAO1	Nutritional/Metabolic factor	ptxR	transcriptional regulator PtxR	32.8	9.24E-40	141
ECs_0755	ECs_0755	transcription regulator	VFG044083 (gb|NP_250948)	Pyoverdine (VF0094)	Effective at acquiring iron from transferrin and lactoferrin; cytotoxic due to its ability to stimulating the production of reactive oxygen species	setA	Pseudomonas aeruginosa PAO1	Nutritional/Metabolic factor	ptxR	transcriptional regulator PtxR	35.2	2.49E-06	48.1
ECs_3400	yfhR	hypothetical protein	VFG007018 (gb|WP_011081430)	MARTX (VF0265)	Inducing cytopathic activities in host cells including actin depolymerization, Rho inactivation, caspase 3/7-dependent apoptosis, and induction of reactive oxygen species; important for evasion of phagocytes	setA	Vibrio vulnificus CMCP6	Exotoxin	rtxA	RTX toxin RtxA	22.4	7.42E-07	50.4
ECs_3407	hcaB	2%2C3-dihydroxy-2%2C3-dihydrophenylpropionate dehydrogenase	VFG007210 (gb|WP_011081751)	Vibriobactin/Vulnibactin (VF0626)	-	setA	Vibrio vulnificus CMCP6	Nutritional/Metabolic factor	vibA	2,3-dihydro-2,3-dihydroxybenzoate dehydrogenase	28.6	1.34E-14	71.6
ECs_1285	ECs_1285	3-oxoacyl-[acyl-ca	VFG038839 (gb|WP_01	Polar flagella	Necessary for motility, adhesion and invasion; glycosylation of the	setA	Aeromonas hydrophila	Motility	flmH	short chain dehydrogen	33.3	7.80E-39	135

		rrier protein] reductase	1707832)	(VF0473)	flagellin may play a role in provoking a proinflammatory response		subsp. hydrophila ATCC 7966			ase/reductase family oxidoreductase			
ECs_5226	bdcA	oxidoreductase	VFG038839 (gb|WP_011707832)	Polar flagella (VF0473)	Necessary for motility, adhesion and invasion; glycosylation of the flagellin may play a role in provoking a proinflammatory response	setA	Aeromonas hydrophila subsp. hydrophila ATCC 7966	Motility	flmH	short chain dehydrogenase/reductase family oxidoreductase	35.6	1.85E-24	97.4
ECs_4340	fabG	3-oxoacyl-[acyl-carrier-protein] reductase	VFG038839 (gb|WP_011707832)	Polar flagella (VF0473)	Necessary for motility, adhesion and invasion; glycosylation of the flagellin may play a role in provoking a proinflammatory response	setA	Aeromonas hydrophila subsp. hydrophila ATCC 7966	Motility	flmH	short chain dehydrogenase/reductase family oxidoreductase	35.1	2.21E-30	113
ECs_3024	yohF	oxidoreductase	VFG038839 (gb|WP_011707832)	Polar flagella (VF0473)	Necessary for motility, adhesion and invasion; glycosylation of the flagellin may play a role in provoking a proinflammatory response	setA	Aeromonas hydrophila subsp. hydrophila ATCC 7966	Motility	flmH	short chain dehydrogenase/reductase family oxidoreductase	32.4	6.01E-29	109
ECs_3887	yghA	oxidoreductase	VFG038839 (gb|WP_011707832)	Polar flagella (VF0473)	Necessary for motility, adhesion and invasion; glycosylation of the flagellin may play a role in provoking a proinflammatory response	setA	Aeromonas hydrophila subsp. hydrophila	Motility	flmH	short chain dehydrogenase/reductase family	30	6.59E-23	94.7

							ATCC 7966			oxidoreductase			
ECs_1275	ECs_1275	oxidoreductase	VFG038839 (gb|WP_011707832)	Polar flagella (VF0473)	Necessary for motility, adhesion and invasion; glycosylation of the flagellin may play a role in provoking a proinflammatory response	setA	Aeromonas hydrophila subsp. hydrophila ATCC 7966	Motility	flmH	short chain dehydrogenase/reductase family oxidoreductase	28.4	1.18E-24	98.6
ECs_1471	fabG_1	3-oxoacyl-[acyl-carrier-protein] reductase	VFG038839 (gb|WP_011707832)	Polar flagella (VF0473)	Necessary for motility, adhesion and invasion; glycosylation of the flagellin may play a role in provoking a proinflammatory response	setA	Aeromonas hydrophila subsp. hydrophila ATCC 7966	Motility	flmH	short chain dehydrogenase/reductase family oxidoreductase	38.3	9.24E-48	158
ECs_3561	srlD	sorbitol-6-phosphate dehydrogenase	VFG038839 (gb|WP_011707832)	Polar flagella (VF0473)	Necessary for motility, adhesion and invasion; glycosylation of the flagellin may play a role in provoking a proinflammatory response	setA	Aeromonas hydrophila subsp. hydrophila ATCC 7966	Motility	flmH	short chain dehydrogenase/reductase family oxidoreductase	27	1.96E-15	73.6
ECs_1861	fabI	enoyl-[acyl-carrier-protein] reductase	VFG038839 (gb|WP_011707832)	Polar flagella (VF0473)	Necessary for motility, adhesion and invasion; glycosylation of the flagellin may play a role in provoking a proinflammatory response	setA	Aeromonas hydrophila subsp. hydrophila ATCC 7966	Motility	flmH	short chain dehydrogenase/reductase family oxidoreductase	26.8	7.88E-16	74.7

ECs_2312	folM	dihydromonapterin reductase	VFG038839 (gb|WP_011707832)	Polar flagella (VF0473)	Necessary for motility, adhesion and invasion; glycosylation of the flagellin may play a role in provoking a proinflammatory response	setB	Aeromonas hydrophila subsp. hydrophila ATCC 7966	Motility	flmH	short chain dehydrogenase/reductase family oxidoreductase	24.6	6.30E-07	48.9
ECs_1843	yciK	EmrKY-TolC system oxoacyl-(acyl carrier protein) reductase	VFG038839 (gb|WP_011707832)	Polar flagella (VF0473)	Necessary for motility, adhesion and invasion; glycosylation of the flagellin may play a role in provoking a proinflammatory response	setA	Aeromonas hydrophila subsp. hydrophila ATCC 7966	Motility	flmH	short chain dehydrogenase/reductase family oxidoreductase	29.3	1.05E-20	87.8
ECs_2327	hdhA	7-alpha-hydroxysteroid dehydrogenase	VFG038839 (gb|WP_011707832)	Polar flagella (VF0473)	Necessary for motility, adhesion and invasion; glycosylation of the flagellin may play a role in provoking a proinflammatory response	setA	Aeromonas hydrophila subsp. hydrophila ATCC 7966	Motility	flmH	short chain dehydrogenase/reductase family oxidoreductase	33.6	1.90E-32	119
ECs_3699	kduD	2-dehydro-3-deoxy-D-gluconate 5-dehydrogenase	VFG038841 (gb|WP_005318273)	Polar flagella (VF0473)	Necessary for motility, adhesion and invasion; glycosylation of the flagellin may play a role in provoking a proinflammatory response	setA	Aeromonas salmonicida subsp. salmonicida A449	Motility	flmH	short chain dehydrogenase/reductase family oxidoreductase	33.3	6.50E-33	120
ECs_3297	ucpA	furfural	VFG005766	<beta>-ha	Forms pores in cell membrane;	setA	Streptococ	Exotoxin	cylG	3-ketoacyl	35.7	3.24E-	134

		resistance protein	(gb|WP_000861302)	emolysin/cytolysin (VF0279)	proinflammatory effects: inducing apoptosis, promoting cellular invasion, triggering iNOS and cytokine release		cus agalactiae NEM316			-ACP-reductase CylG		38	
ECs_5003	sorD	sorbitol-6-phosphate 2-dehydrogenase	VFG005766 (gb|WP_000861302)	<beta>-haemolysin/cytolysin (VF0279)	Forms pores in cell membrane; proinflammatory effects: inducing apoptosis, promoting cellular invasion, triggering iNOS and cytokine release	setA	Streptococcus agalactiae NEM316	Exotoxin	cylG	3-ketoacyl-ACP-reductase CylG	35.2	1.10E-32	119
ECs_3630	ygcW	SDR family oxidoreductase	VFG005766 (gb|WP_000861302)	<beta>-haemolysin/cytolysin (VF0279)	Forms pores in cell membrane; proinflammatory effects: inducing apoptosis, promoting cellular invasion, triggering iNOS and cytokine release	setA	Streptococcus agalactiae NEM316	Exotoxin	cylG	3-ketoacyl-ACP-reductase CylG	31.4	1.10E-34	124
ECs_3404	hcaE	3-phenylpropionate dioxygenase large subunit	VFG044121 (gb|WP_005013753)	Alcaligin (VF0688)	-	setB	Bordetella pertussis Tohama I	Nutritional/Metabolic factor	alcE	alcaligin siderophore biosynthesis protein AlcE	27	1.37E-10	62.8
ECs_2511	yeaW	dioxygenase alpha subunit	VFG044121 (gb|WP_005013753)	Alcaligin (VF0688)	-	setB	Bordetella pertussis Tohama I	Nutritional/Metabolic factor	alcE	alcaligin siderophore biosynthesis protein AlcE	24	4.76E-14	72.8

ECs_0998	lpxK	lipid A 4'kinase	VFG013244 (gb|WP_005687511)	LOS (VF0044)	Major immunogen; LOS phosphorylcholine (ChoP) may influence invasion via interaction with PAF receptor and stimulates of inflammatory signals; LPS phase variation is characterized by the spontaneous loss and gain of oligosaccharide structures present in the outer core. the phase variable expression of LPS biosynthesis genes promotes evasion of antigen-specific host immune defences and allow colonization of different host microenvironments	setA	Haemophilus influenzae PittEE	Immune modulation	lpxK	tetraacyldisaccharide 4'-kinase	51.3	6.73E-104	307
gene-katP	katP	BAA31833.1	VFG001861 (gb|WP_010945955)	KatAB (VF0168)	A periplasmic catalase, expressed maximally during the post-exponential phase; important for intracellular survival and transmission	setA	Legionella pneumophila subsp. pneumophila str. Philadelphia 1	Stress survival	katA	catalase/(hydro)peroxidase	62.1	0	943
ECs_4871	katG	catalase-peroxidase HPI	VFG009667 (gb|WP_011780414)	KatG (VF0303)	Catalase:peroxidase degrades H2O2 and organic peroxides, the major role is to catabolize the peroxides generated by phagocyte NADPH oxidase	setA	Mycobacterium vanbaaleni i PYR-1	Stress survival	katG	catalase/peroxidase HPI	61.5	1.19E-305	852
ECs_4190	tufB	translati	VFG046459	EF-Tu	Surface-expressed elongation	setA	Francisell	Adherence	tufA	elongation	81.4	9.26E-	677

		on elongation factor EF-Tu 1	(gb|WP_004287053)	(VF0460)	factor-Tu (EF-Tu) mediates attachment by interacting with host cell nucleolin		a philomiragia subsp. philomiragia ATCC 25017			factor Tu		248	
ECs_3435	lepA	back-translocating elongation factor EF4	VFG046459 (gb|WP_004287053)	EF-Tu (VF0460)	Surface-expressed elongation factor-Tu (EF-Tu) mediates attachment by interacting with host cell nucleolin	setA	Francisella philomiragia subsp. philomiragia ATCC 25017	Adherence	tufA	elongation factor Tu	27.1	3.14E-16	80.9
ECs_4903	tufB	translation elongation factor EF-Tu 1	VFG046459 (gb|WP_004287053)	EF-Tu (VF0460)	Surface-expressed elongation factor-Tu (EF-Tu) mediates attachment by interacting with host cell nucleolin	setA	Francisella philomiragia subsp. philomiragia ATCC 25017	Adherence	tufA	elongation factor Tu	81.7	4.59E-248	678
ECs_5333	prfC	peptide chain release factor RF-3	VFG046459 (gb|WP_004287053)	EF-Tu (VF0460)	Surface-expressed elongation factor-Tu (EF-Tu) mediates attachment by interacting with host cell nucleolin	setA	Francisella philomiragia subsp. philomiragia ATCC	Adherence	tufA	elongation factor Tu	30.1	9.83E-10	60.5

							25017						
ECs_4467	selB	selenocysteinyl-tRNA-specific translation factor	VFG046474 (gb|WP_014714676)	EF-Tu (VF0460)	Surface-expressed elongation factor-Tu (EF-Tu) mediates attachment by interacting with host cell nucleolin	setA	Francisella noatunensis subsp. orientalis str. Toba 04	Adherence	tufA	elongation factor Tu	31	2.86E-36	140
ECs_4793	typA	GTP-binding protein	VFG046467 (gb|WP_014548920)	EF-Tu (VF0460)	Surface-expressed elongation factor-Tu (EF-Tu) mediates attachment by interacting with host cell nucleolin	setA	Francisella cf. tularensis subsp. novicida 3523	Adherence	tufA	elongation factor Tu	31.9	6.94E-25	107
ECs_4926	zraS	two-component system sensor histidine kinase ZraS	VFG038759 (gb|WP_011706627)	Polar flagella (VF0473)	Necessary for motility, adhesion and invasion; glycosylation of the flagellin may play a role in provoking a proinflammatory response	setA	Aeromonas hydrophila subsp. hydrophila ATCC 7966	Motility	flrB	two-component sensor kinase	33.8	9.35E-26	107
ECs_5074	ECs_5074	histidine protein kinase	VFG038759 (gb|WP_011706627)	Polar flagella (VF0473)	Necessary for motility, adhesion and invasion; glycosylation of the flagellin may play a role in provoking a proinflammatory response	setA	Aeromonas hydrophila subsp. hydrophila ATCC 7966	Motility	flrB	two-component sensor kinase	30.5	4.03E-15	77.4

ECs_5107	dcuS	two-component system sensor histidine kinase DcuS	VFG015678 (gb|WP_012273512)	Flagella (VF0273)	Swimming motility; play a role in biofilm formation and other pathogenic adaptations	setA	Pseudomonas putida GB-1	Motility	fleS	two-component sensor	26.1	2.12E-14	75.1
ECs_0997	msbA	lipid ABC transporter permease/ATPase	VFG013253 (gb|WP_011609176)	LOS (VF0044)	Major immunogen; LOS phosphorylcholine (ChoP) may influence invasion via interaction with PAF receptor and stimulates of inflammatory signals; LPS phase variation is characterized by the spontaneous loss and gain of oligosaccharide structures present in the outer core. the phase variable expression of LPS biosynthesis genes promotes evasion of antigen-specific host immune defences and allow colonization of different host microenvironments	setA	Haemophilus somnus 129PT	Immune modulation	msbA	lipid transporter ATP-binding/permease	67.1	1.65E-275	763
ECs_0503	mdlB	multidrug ABC transporter ATPase	VFG013253 (gb|WP_011609176)	LOS (VF0044)	Major immunogen; LOS phosphorylcholine (ChoP) may influence invasion via interaction with PAF receptor and stimulates of inflammatory signals; LPS phase	setA	Haemophilus somnus 129PT	Immune modulation	msbA	lipid transporter ATP-binding/permease	29	4.45E-66	226

					variation is characterized by the spontaneous loss and gain of oligosaccharide structures present in the outer core. the phase variable expression of LPS biosynthesis genes promotes evasion of antigen-specific host immune defences and allow colonization of different host microenvironments								
ECs_0502	mdlA	multidrug ABC transporter ATPase	VFG013249 (gb|WP_011271803)	LOS (VF0044)	Major immunogen; LOS phosphorylcholine (ChoP) may influence invasion via interaction with PAF receptor and stimulates of inflammatory signals; LPS phase variation is characterized by the spontaneous loss and gain of oligosaccharide structures present in the outer core. the phase variable expression of LPS biosynthesis genes promotes evasion of antigen-specific host immune defences and allow colonization of different host microenvironments	setA	Haemophilus influenzae 86-028NP	Immune modulation	msbA	lipid transporter ATP-binding/permease	31.2	2.12E-72	243
ECs_0971	cydC	glutathione ABC transport	VFG013249 (gb|WP_011271803)	LOS (VF0044)	Major immunogen; LOS phosphorylcholine (ChoP) may influence invasion via interaction	setA	Haemophilus influenzae	Immune modulation	msbA	lipid transporter	28.6	9.87E-48	175

		er ATP-binding protein			with PAF receptor and stimulates of inflammatory signals; LPS phase variation is characterized by the spontaneous loss and gain of oligosaccharide structures present in the outer core. the phase variable expression of LPS biosynthesis genes promotes evasion of antigen-specific host immune defences and allow colonization of different host microenvironments		86-028NP			ATP-binding/permease			
ECs_3089	ccmB	heme export ABC transporter permease	VFG010866 (gb|WP_010946593)	CcmC (VF0292)	Required for cytochrome c production, promotes iron assimilation and intracellular infection	setA	Legionella pneumophila subsp. pneumophila str. Philadelphia 1	Nutritional/Metabolic factor	ccmB	ABC transporter involved in cytochrome c biogenesis, CcmB subunit	38.7	1.97E-40	137
ECs_3088	ccmC	heme export ABC transporter permease	VFG010872 (gb|WP_011215037)	CcmC (VF0292)	Required for cytochrome c production, promotes iron assimilation and intracellular infection	setA	Legionella pneumophila str. Lens	Nutritional/Metabolic factor	ccmC	cytochrome c-type biogenesis protein CcmC, putative	49	6.11E-84	250

ECs_3086	ccmE	cytochrome c-type biogenesis protein CcmE	VFG010881 (gb|WP_011945970)	CcmC (VF0292)	Required for cytochrome c production, promotes iron assimilation and intracellular infection	setA	Legionella pneumophila str. Corby	Nutritional/Metabolic factor	ccmE	heme lyase for CcmE cytochrome c-type biogenesis protein CcmE, heme chaperone	48.3	1.29E-36	123
ECs_3085	ccmF	heme lyase subunit CcmF	VFG045720 (gb|WP_012979217)	CcmC (VF0292)	Required for cytochrome c production, promotes iron assimilation and intracellular infection	setA	Legionella longbeachae NSW150	Nutritional/Metabolic factor	ccmF	cytochrome c heme lyase subunit CcmF	53.1	6.80E-225	640
ECs_5056	nrfE	heme lyase subunit NrfE	VFG045720 (gb|WP_012979217)	CcmC (VF0292)	Required for cytochrome c production, promotes iron assimilation and intracellular infection	setA	Legionella longbeachae NSW150	Nutritional/Metabolic factor	ccmF	cytochrome c heme lyase subunit CcmF	38	2.07E-114	353
ECs_3082	narP	two-component regulatory system response regulator NarP	VFG045728 (gb|WP_003635449)	LetA/S (VF0262)	Activates gene expression indirectly by counteracting the repressor of translation known as CsrA or RsmA	setA	Legionella longbeachae NSW150	Regulation	letA	UvrY/SirA/GacA family response regulator transcription factor	33.3	3.18E-32	116
ECs_4606	uhpA	two-compo	VFG045728	LetA/S	Activates gene expression indirectly	setA	Legionella	Regulation	letA	UvrY/SirA/	37.2	9.89E-	114

		nent regulatory system response regulator UhpA	(gb|WP_003635449)	(VF0262)	by counteracting the repressor of translation known as CsrA or RsmA		longbeachae NSW150			GacA family response regulator transcription factor		32	
ECs_3712	ECs_3712	transcriptional regulator	VFG041872 (gb|WP_115610154)	Cpi-2 encoded T3SS (SPI-2 like) (VF1260)	-	setA	Chromobacterium violaceum ATCC 12472	Effector delivery system	CV_RS12720	two component system response regulator	31.7	3.94E-27	102
ECs_4400	yhjB	transcriptional response regulator	VFG041872 (gb|WP_115610154)	Cpi-2 encoded T3SS (SPI-2 like) (VF1260)	-	setA	Chromobacterium violaceum ATCC 12472	Effector delivery system	CV_RS12720	two component system response regulator	21.1	1.19E-10	58.5
ECs_0418	ECs_0418	transcription regulator	VFG042242 (gb|AAG01455)	T3SS (VF1283)	-	setA	Pantoea stewartii subsp. stewartii str. SS104	Effector delivery system	hrpY	HrpY	31.6	1.87E-34	121
ECs_4260	malT	mal regulon transcrip	VFG024200 (gb|WP_008262428)	DevRS (VF0317)	Controls the global response to oxidative stress and low oxygen repsonse, key regulator in the oxygen	setA	Mycobacterium indicus pranii MTCC	Regulation	devR/dosR	response regulator transcript	45.5	3.63E-06	48.9

		tional activator			starvation-induced mycobacterial dormancy response		9506			ion factor			
ECs_1726	narL	two-component regulatory system response regulator NarL	VFG023572 (gb|WP_005156725)	TTSS (chromosomally encoded) (VF1023)	-	setA	Yersinia enterocolitica subsp. palearctica 105.5R(r)	Effector delivery system	YE105_RS01705	two component system response regulator	35	7.84E-38	130
ECs_3081	yejO	autotransporter outer membrane protein	VFG036042 (gb|WP_001045641)	AIDA-I type (VF1132)	-	setA	Escherichia coli O78:H11:K80 str. H10407	Adherence	tibA	autotransporter adhesin/invasin glycoprotein TibA	29.5	5.52E-65	235
ECs_2373	ydhQ	putative enzyme	VFG036042 (gb|WP_001045641)	AIDA-I type (VF1132)	-	setB	Escherichia coli O78:H11:K80 str. H10407	Adherence	tibA	autotransporter adhesin/invasin glycoprotein TibA	24.1	4.50E-09	58.5
ECs_3515	ypjA	adhesin autotransporter	VFG036042 (gb|WP_001045641)	AIDA-I type (VF1132)	-	setA	Escherichia coli O78:H11:K80 str. H10407	Adherence	tibA	autotransporter adhesin/invasin glycoprote	35.2	6.23E-64	237

										in TibA			
ECs_2682	fliK	flagellar hook-length control protein	VFG043112 (gb|WP_000620074)	Peritrichous flagella (VF1154)	-	setA	Escherichia coli O157:H7 str. EDL933	Motility	fliK	flagellar hook length control protein FliK	99.2	1.75E-247	675
ECs_2680	fliI	flagellum-specific ATP synthase	VFG043110 (gb|WP_000213294)	Peritrichous flagella (VF1154)	-	setA	Escherichia coli O157:H7 str. EDL933	Motility	fliI	flagellum-specific ATP synthase FliI	100	0	880
ECs_2681	fliJ	flagellar protein	VFG043111 (gb|WP_000807584)	Peritrichous flagella (VF1154)	-	setA	Escherichia coli O157:H7 str. EDL933	Motility	fliJ	flagella biosynthesis chaperone FliJ	100	2.62E-97	276
ECs_2686	fliO	flagellar biosynthesis protein	VFG043116 (gb|WP_001302429)	Peritrichous flagella (VF1154)	-	setA	Escherichia coli O157:H7 str. EDL933	Motility	fliO	flagellar type III secretion system protein FliO	100	1.21E-76	222
ECs_2687	fliP	flagellar biosynthesis protein	VFG043117 (gb|WP_001253441)	Peritrichous flagella (VF1154)	-	setA	Escherichia coli O157:H7 str. EDL933	Motility	fliP	flagellar type III secretion system pore	100	9.90E-166	457

										protein FliP			
ECs_2684	fliM	flagellar motor switching and energizing component	VFG043114 (gb|WP_010904778)	Peritrichous flagella (VF1154)	-	setA	Escherichia coli O157:H7 str. EDL933	Motility	fliM	flagellar motor switch protein FliM	99.7	6.39E-243	660
ECs_2866	wzb	colanic acid production protein-tyrosine-phosphatase	VFG048962 (gb|WP_015875029)	Capsule (VF0560)	Assisting in evading the host immune system by protecting bacteria from opsonophagocytosis and serum killing	setA	Klebsiella pneumoniae subsp. pneumoniae NTUH-K2044	Immune modulation	KP1_RS17335	protein-tyrosine-phosphatase	56.3	2.26E-56	172
ECs_1138	etp	O-antigen capsule forming protein-tyrosine-phosphatase	VFG048933 (gb|WP_038423093)	Capsule (VF0560)	Assisting in evading the host immune system by protecting bacteria from opsonophagocytosis and serum killing	setA	Klebsiella oxytoca KCTC 1686	Immune modulation	KOX_RS25030	protein-tyrosine-phosphatase	63.1	1.29E-60	183
ECs_2867	wza	colanic acid	VFG048832 (gb|WP_01	Capsule (VF0560)	Assisting in evading the host immune system by protecting bacteria from	setA	Klebsiella variicola	Immune modulation	KVAR_RS07835	polysaccharide export	66.5	1.70E-189	528

		export protein	2967585)		opsonophagocytosis and serum killing		At-22			protein			
ECs_1139	gfcE	O-antigen capsule outer membrane auxiliary protein export channel	VFG048832 (gb|WP_012967585)	Capsule (VF0560)	Assisting in evading the host immune system by protecting bacteria from opsonophagocytosis and serum killing	setA	Klebsiella variicola At-22	Immune modulation	KVAR_RS07835	polysaccharide export protein	74.9	1.46E-218	602
ECs_2688	fliQ	flagellar biosynthesis protein	VFG043118 (gb|WP_000187358)	Peritrichous flagella (VF1154)	-	setA	Escherichia coli O157:H7 str. EDL933	Motility	fliQ	flagellar biosynthesis protein FliQ	100	3.51E-52	157
ECs_2689	fliR	flagellar export pore protein	VFG043119 (gb|WP_000942326)	Peritrichous flagella (VF1154)	-	setA	Escherichia coli O157:H7 str. EDL933	Motility	fliR	flagellar type III secretion system protein FliR	100	5.69E-173	477
ECs_2862	wcaC	glycosyl transferase	VFG050198 (gb|WP_001265344)	Polysaccharide capsule (VF0659)	-	setB	Bacillus cereus AH187	Immune modulation	BCAH187_RS26515	glycosyltransferase	25.9	8.71E-27	110
ECs_2860	wcaE	glycosyl transfera	VFG017347 (gb|WP_01	O-antigen (VF0392)	LPS O antigen mutants were severely impaired in their ability to colonize	setA	Yersinia pseudotube	Immune modulation	YPSIP31758_RS16535	glycosyltransferase	28.6	5.29E-14	69.3

		se	2105562)		the Peyer's patches and did not colonize spleen and liver. The absence of O antigen in the outer membrane affects the expression of other Yersinia virulence factors.		rculosis IP 31758						
ECs_2843	wcaE	glycosyltransferase	VFG017347 (gb|WP_012105562)	O-antigen (VF0392)	LPS O antigen mutants were severely impaired in their ability to colonize the Peyer's patches and did not colonize spleen and liver. The absence of O antigen in the outer membrane affects the expression of other Yersinia virulence factors.	setA	Yersinia pseudotuberculosis IP 31758	Immune modulation	YPSIP31758_RS16535	glycosyltransferase	33.2	9.26E-20	85.1
ECs_4577	grlA	positive regulator GrlA	VFG000821 (gb|WP_000444180)	TTSS (VF0191)	Injects Tir and other effector molecules directly into the host cell. Effector molecules activate cell-signaling pathways, causing alterations in the host cell cytoskeleton and resulting in the depolymerization of actin and the loss of microvilli	setA	Escherichia coli O157:H7 str. EDL933	Effector delivery system	glrA	type III secretion system LEE transcriptional regulator GrlA	100	6.38E-100	282
ECs_2045	ydcT	ABC transporter ATPase	VFG030700 (gb|WP_005098896)	Trehalose-recycling ABC transporter (VF0842)	-	setA	Mycobacterium abscessus subsp. bolletii 50594	Nutritional/Metabolic factor	sugC	sn-glycerol-3-phosphate ABC transporter ATP-bindin	45.6	2.26E-64	208

										g protein UgpC			
ECs_4312	ftsE	cell division ATP-binding protein	VFG030700 (gb|WP_005098896)	Trehalose-recycling ABC transporter (VF0842)	-	setA	Mycobacterium abscessus subsp. bolletii 50594	Nutritional/Metabolic factor	sugC	sn-glycerol-3-phosphate ABC transporter ATP-binding protein UgpC	39.2	7.91E-33	122
ECs_0887	glnQ	ATP-binding component of glutamine high-affinity transport system	VFG030700 (gb|WP_005098896)	Trehalose-recycling ABC transporter (VF0842)	-	setA	Mycobacterium abscessus subsp. bolletii 50594	Nutritional/Metabolic factor	sugC	sn-glycerol-3-phosphate ABC transporter ATP-binding protein UgpC	35.4	1.98E-40	142
ECs_4347	nikE	nickel ABC transporter ATPase	VFG030700 (gb|WP_005098896)	Trehalose-recycling ABC transporter (VF0842)	-	setA	Mycobacterium abscessus subsp. bolletii 50594	Nutritional/Metabolic factor	sugC	sn-glycerol-3-phosphate ABC transporter ATP-binding protein	35.6	2.92E-31	119

ECs_3803	ECs_3803	ABC transporter ATP-binding protein	VFG030700 (gb|WP_005098896)	Trehalose-recycling ABC transporter (VF0842)	-	setA	Mycobacterium abscessus subsp. bolletii 50594	Nutritional/Metabolic factor	sugC	UgpC sn-glycerol-3-phosphate ABC transporter ATP-binding protein	28.9	1.31E-21	91.7
ECs_1016	ssuB	aliphatic sulfonate ABC transporter ATPase	VFG030700 (gb|WP_005098896)	Trehalose-recycling ABC transporter (VF0842)	-	setA	Mycobacterium abscessus subsp. bolletii 50594	Nutritional/Metabolic factor	sugC	UgpC sn-glycerol-3-phosphate ABC transporter ATP-binding protein	37.6	2.06E-31	119
ECs_0947	artP	arginine ABC transporter ATPase	VFG030700 (gb|WP_005098896)	Trehalose-recycling ABC transporter (VF0842)	-	setA	Mycobacterium abscessus subsp. bolletii 50594	Nutritional/Metabolic factor	sugC	UgpC sn-glycerol-3-phosphate ABC transporter ATP-binding protein	38.6	7.04E-35	128
ECs_4296	ugpC	sn-glycer	VFG030692	Trehalose	-	setA	Mycobacter	Nutritiona	sugC	UgpC sn-glycero	50.4	3.67E-	331

		ol-3-phosphate ABC transporter ATPase	(gb|WP_013828150)	-recycling ABC transporter (VF0842)			ium sp. JDM601	l/Metabolic factor		l-3-phosphate ABC transporter ATP-binding protein UgpC		112	
ECs_1897	ycjV	sugar transporter subunit	VFG030692 (gb|WP_013828150)	Trehalose-recycling ABC transporter (VF0842)	-	setA	Mycobacterium sp. JDM601	Nutritional/Metabolic factor	sugC	sn-glycerol-3-phosphate ABC transporter ATP-binding protein UgpC	50.3	2.11E-112	332
ECs_0935	potG	putrescine ABC transporter ATPase	VFG030692 (gb|WP_013828150)	Trehalose-recycling ABC transporter (VF0842)	-	setA	Mycobacterium sp. JDM601	Nutritional/Metabolic factor	sugC	sn-glycerol-3-phosphate ABC transporter ATP-binding protein UgpC	49.6	1.46E-71	228
ECs_5073	ECs_5073	ATP-binding component	VFG030692 (gb|WP_013828150)	Trehalose-recycling ABC	-	setA	Mycobacterium sp. JDM601	Nutritional/Metabolic factor	sugC	sn-glycerol-3-phosphate ABC	31	1.14E-21	96.7

		of sugar ABC transporter		transporter (VF0842)						transporter ATP-binding protein UgpC			
ECs_3540	proV	glycine betaine/proline ABC transporter periplasmic binding protein	VFG030692 (gb|WP_013828150)	Trehalose-recycling ABC transporter (VF0842)	-	setA	Mycobacterium sp. JDM601	Nutritional/Metabolic factor	sugC	sn-glycerol-3-phosphate ABC transporter ATP-binding protein UgpC	40.2	5.63E-49	170
ECs_5018	malK	maltose ABC transporter ATPase	VFG030680 (gb|WP_011856409)	Trehalose-recycling ABC transporter (VF0842)	-	setA	Mycobacterium sp. JLS	Nutritional/Metabolic factor	sugC	sn-glycerol-3-phosphate ABC transporter ATP-binding protein UgpC	52.7	4.86E-104	312
ECs_2046	ydcU	ABC transporter	VFG013191 (gb|WP_011608935)	HitABC (VF0268)	HitABC(fbpABC) operon encodes a periplasmic-binding protein-dependent iron transport	setA	Haemophilus somnus 2336	Nutritional/Metabolic factor	hitB	iron ABC transporter permease	26.9	2.20E-11	64.3

		permease			system necessary for the utilization of iron bound to transferrin or iron chelates								
ECs_3294	cysW	sulfate/thiosulfate ABC transporter permease	VFG013191 (gb|WP_011608935)	HitABC (VF0268)	HitABC(fbpABC) operon encodes a periplasmic-binding protein-dependent iron transport system necessary for the utilization of iron bound to transferrin or iron chelates	setB	Haemophilus somnus 2336	Nutritional/Metabolic factor	hitB	iron ABC transporter permease	22	3.15E-10	60.5
ECs_0792	modB	molybdate ABC transporter permease	VFG013191 (gb|WP_011608935)	HitABC (VF0268)	HitABC(fbpABC) operon encodes a periplasmic-binding protein-dependent iron transport system necessary for the utilization of iron bound to transferrin or iron chelates	setA	Haemophilus somnus 2336	Nutritional/Metabolic factor	hitB	iron ABC transporter permease	31.4	6.55E-17	79
ECs_0414	ECs_0414	ferric transport system permease	VFG006252 (gb|WP_002246860)	FbpABC (VF0272)	Encodes a periplasmic-binding protein-dependent iron transport system necessary for the utilization of iron bound to transferrin or iron chelates, FbpA is the periplasmic Fe3+ binding protein	setA	Neisseria meningitidis Z2491	Nutritional/Metabolic factor	fbpB	iron(III) ABC transporter, permease protein	25.1	3.39E-18	88.2
ECs_3295	cysU	sulfate/thiosulfate ABC transporter	VFG000249 (gb|WP_002214251)	FbpABC (VF0272)	Encodes a periplasmic-binding protein-dependent iron transport system necessary for the utilization of iron bound to transferrin or iron chelates, FbpA is the periplasmic Fe3+	setA	Neisseria meningitidis MC58	Nutritional/Metabolic factor	fbpB	iron(III) ABC transporter, permease protein	27.3	5.95E-15	74.3

		permease			binding protein								
ECs_2047	ydcV	ABC transporter permease	VFG030629 (gb|WP_094987104)	Trehalose-recycling ABC transporter (VF0842)	-	setB	Mycobacterium ulcerans Agy99	Nutritional/Metabolic factor	sugB	carbohydrate ABC transporter permease	27.5	5.40E-10	58.5
ECs_4297	ugpE	sn-glycerol-3-phosphate ABC transporter permease	VFG030637 (gb|WP_049785257)	Trehalose-recycling ABC transporter (VF0842)	-	setB	Mycobacterium marinum M	Nutritional/Metabolic factor	sugB	carbohydrate ABC transporter permease	24.8	5.50E-18	81.6
ECs_1891	ycjP	sugar ABC transporter permease	VFG030637 (gb|WP_049785257)	Trehalose-recycling ABC transporter (VF0842)	-	setB	Mycobacterium marinum M	Nutritional/Metabolic factor	sugB	carbohydrate ABC transporter permease	33.9	7.71E-38	134
ECs_0936	potH	putrescine ABC transporter permease	VFG030637 (gb|WP_049785257)	Trehalose-recycling ABC transporter (VF0842)	-	setA	Mycobacterium marinum M	Nutritional/Metabolic factor	sugB	carbohydrate ABC transporter permease	24.2	1.03E-06	49.3
ECs_1388	pchD	transcrip	VFG042578	Locus for	-	setB	Escherichi	Adherence	AAX78194	LdaB	74	1.15E-	106

		tional regulator PchD	(gb|AAX78194)	diffuse adherence (lda), afimbrial adhesin (VF1148)			a coli					31	
ECs_1437	solA	N-methyltryptophan oxidase	VFG015929 (gb|WP_011536481)	Hydrogen cyanide production (VF0920)	-	setB	Pseudomonas entomophila L48	Antimicrobial activity/Competitive advantage	hcnC	cyanide-forming glycine dehydrogenase subunit HcnC	21	2.71E-06	48.9
ECs_3126	glpA	anaerobic sn-glycerol-3-phosphate dehydrogenase large FAD/NAD(P)-binding subunit	VFG015929 (gb|WP_011536481)	Hydrogen cyanide production (VF0920)	-	setB	Pseudomonas entomophila L48	Antimicrobial activity/Competitive advantage	hcnC	cyanide-forming glycine dehydrogenase subunit HcnC	24.8	2.26E-15	78.2
ECs_2919	mrp	antiporter inner membrane protein	VFG043350 (gb|WP_011946724)	Flagella (VF0157)	Not required for intracellular growth but enhance the invasion capacity	setA	Legionella pneumophila subsp. pneumophil	Motility	fleN	flagellar synthesis regulator FleN	28.6	1.56E-14	73.2

							a str. Philadelphia 1						
gene-sopA	sopA	SopA protein	VFG014628 (gb|WP_011913681)	Flagella (VF0273)	Swimming motility; play a role in biofilm formation and other pathogenic adaptations	setA	Pseudomonas stutzeri A1501	Motility	fleN	flagellar synthesis regulator FleN	28.2	2.53E-06	48.5
ECs_2916	yehC	periplasmic pilin chaperone	VFG004271 (gb|WP_000698775)	Stc (VF0955)	-	setA	Salmonella enterica subsp. enterica serovar Typhi str. CT18	Adherence	stcB	fimbrial biogenesis chaperone StcB	64.3	3.65E-103	297
ECs_0023	ECs_0023	fimbrial chaperone	VFG004271 (gb|WP_000698775)	Stc (VF0955)	-	setA	Salmonella enterica subsp. enterica serovar Typhi str. CT18	Adherence	stcB	fimbrial biogenesis chaperone StcB	61	3.49E-97	282
ECs_2917	yehD	fimbrial-like adhesin protein	VFG021740 (gb|WP_000830689)	Peg (VF0965)	-	setB	Salmonella enterica subsp. enterica serovar	Adherence	pegA	fimbrial protein YehD	33.3	5.33E-18	77

							Newport str. SL254						
ECs_0024	ECs_0024	fimbrial protein	VFG004278 (gb|WP_000830701)	Peg (VF0965)	-	setB	Salmonella enterica subsp. enterica serovar Paratyphi A str. ATCC 9150	Adherence	pegA	fimbrial protein YehD	35.5	2.50E-18	77.8
ECs_2914	yehA	fimbrial-like adhesin protein	VFG021471 (gb|WP_000765278)	Stc (VF0955)	-	setB	Salmonella enterica subsp. enterica serovar Agona str. SL483	Adherence	stcD	putative fimbrial-like adhesin protein	48.4	3.12E-50	170
ECs_2915	yehB	fimbrial assembly protein	VFG018328 (gb|WP_000945363)	Stc (VF0955)	-	setA	Salmonella enterica subsp. enterica serovar Paratyphi B str. SPB7	Adherence	stcC	fimbrial biogenesis outer membrane usher protein	75	0	1320
ECs_0022	ECs_0022	fimbrial assembly	VFG018328 (gb|WP_00	Stc (VF0955)	-	setA	Salmonella enterica	Adherence	stcC	fimbrial biogenesis	59.1	0	1020

		protein	0945363)				subsp. enterica serovar Paratyphi B str. SPB7			outer membrane usher protein			
ECs_4923	hupA	transcriptional regulator HU subunit alpha	VFG043551 (gb|WP_010908478)	ML1683 (VF0867)	-	setB	Mycobacterium leprae TN	Adherence	ML_RS08565	HU family DNA-binding protein	41.6	1.03E-17	73.6
ECs_0494	hupB	transcriptional regulator HU subunit beta	VFG043551 (gb|WP_010908478)	ML1683 (VF0867)	-	setB	Mycobacterium leprae TN	Adherence	ML_RS08565	HU family DNA-binding protein	33.7	1.35E-13	62.8
ECs_2419	ihfA	integration host factor DNA-binding protein alpha subunit	VFG043551 (gb|WP_010908478)	ML1683 (VF0867)	-	setB	Mycobacterium leprae TN	Adherence	ML_RS08565	HU family DNA-binding protein	39.1	4.57E-15	67
ECs_2596	tsr_1	methyl-ac	VFG043092	Peritrich	-	setA	Escherichi	Motility	tar	methyl-acc	98.6	4.78e-	853

		cepting chemotaxis protein II	(gb|WP_010904770)	ous flagella (VF1154)			a coli O157:H7 str. EDL933			epting chemotaxis protein II		312	
ECs_2595	tap	methyl-accepting chemotaxis protein IV	VFG043092 (gb|WP_010904770)	Peritrichous flagella (VF1154)	-	setA	Escherichia coli O157:H7 str. EDL933	Motility	tar	methyl-accepting chemotaxis protein II	41.1	4.73E-73	242
ECs_2026	trg	methyl-accepting chemotaxis protein III	VFG043092 (gb|WP_010904770)	Peritrichous flagella (VF1154)	-	setA	Escherichia coli O157:H7 str. EDL933	Motility	tar	methyl-accepting chemotaxis protein II	62.6	2.39E-54	192
ECs_5315	tsr	methyl-accepting chemotaxis protein II	VFG043092 (gb|WP_010904770)	Peritrichous flagella (VF1154)	-	setA	Escherichia coli O157:H7 str. EDL933	Motility	tar	methyl-accepting chemotaxis protein II	54	2.87E-131	394
ECs_3954	aer	aerotaxis receptor	VFG043092 (gb|WP_010904770)	Peritrichous flagella (VF1154)	-	setA	Escherichia coli O157:H7 str. EDL933	Motility	tar	methyl-accepting chemotaxis protein II	55.3	1.13E-41	156
ECs_2594	cheR	chemotaxis protein methyltra	VFG043091 (gb|WP_000204340)	Peritrichous flagella	-	setA	Escherichia coli O157:H7	Motility	cheR	protein-glutamate O-methyltr	100	7.49E-211	575

		nsferase CheR		(VF1154)			str. EDL933			ansferase CheR			
ECs_0391	cynR	transcriptional activator of cyn operon	VFG037468 (gb|WP_001105542)	HemO cluster (VF0636)	Current studies show the hemO gene cluster is required for optimal utilization of heme in A. baumannii hypervirulent strains; The hemO gene cluster encodes a secreted hemophore.	setA	Acinetobacter baumannii AB0057	Nutritional/Metabolic factor	AB57_RS05095	LysR family transcriptional regulator	27.3	3.01E-21	91.3
ECs_1923	abgR	transcriptional regulator of abgABT operon	VFG037468 (gb|WP_001105542)	HemO cluster (VF0636)	Current studies show the hemO gene cluster is required for optimal utilization of heme in A. baumannii hypervirulent strains; The hemO gene cluster encodes a secreted hemophore.	setA	Acinetobacter baumannii AB0057	Nutritional/Metabolic factor	AB57_RS05095	LysR family transcriptional regulator	22.8	3.50E-10	59.7
ECs_2027	ydcI	transcriptional regulator	VFG037468 (gb|WP_001105542)	HemO cluster (VF0636)	Current studies show the hemO gene cluster is required for optimal utilization of heme in A. baumannii hypervirulent strains; The hemO gene cluster encodes a secreted hemophore.	setA	Acinetobacter baumannii AB0057	Nutritional/Metabolic factor	AB57_RS05095	LysR family transcriptional regulator	24	8.23E-11	61.6
ECs_3032	ECs_3032	regulatory protein	VFG037468 (gb|WP_001105542)	HemO cluster (VF0636)	Current studies show the hemO gene cluster is required for optimal utilization of heme in A. baumannii hypervirulent strains; The hemO gene cluster encodes a secreted hemophore.	setA	Acinetobacter baumannii AB0057	Nutritional/Metabolic factor	AB57_RS05095	LysR family transcriptional regulator	23.7	9.10E-09	55.5
ECs_2783	cbl	ssuEADCB/tauABCD operon transcrip	VFG037468 (gb|WP_001105542)	HemO cluster (VF0636)	Current studies show the hemO gene cluster is required for optimal utilization of heme in A. baumannii hypervirulent strains; The hemO gene	setA	Acinetobacter baumannii AB0057	Nutritional/Metabolic factor	AB57_RS05095	LysR family transcriptional regulator	24.4	5.29E-10	59.3

		tional activator			cluster encodes a secreted hemophore.							
ECs_2784	nac	nitrogen assimilation regulon transcriptional regulator	VFG037478 (gb|WP_001105560)	HemO cluster (VF0636)	Current studies show the hemO gene cluster is required for optimal utilization of heme in A. baumannii hypervirulent strains; The hemO gene cluster encodes a secreted hemophore.	setA	Acinetobacter baumannii MDR-ZJ06	Nutritional/Metabolic factor	ABZJ_RS11145	LysR family transcriptional regulator	24.9	1.66E-20	89.4
ECs_2591	cheZ	chemotaxis protein CheZ	VFG043088 (gb|WP_000983602)	Peritrichous flagella (VF1154)	-	setA	Escherichia coli O157:H7 str. EDL933	Motility	cheZ	protein phosphatase CheZ	100	2.88E-145	403
ECs_2598	cheA	chemotaxis protein CheA	VFG043094 (gb|WP_001322280)	Peritrichous flagella (VF1154)	-	setA	Escherichia coli O157:H7 str. EDL933	Motility	cheA	chemotaxis protein CheA	99.8	0	1223
ECs_2599	motB	chemotaxis protein MotB	VFG043095 (gb|WP_000795630)	Peritrichous flagella (VF1154)	-	setA	Escherichia coli O157:H7 str. EDL933	Motility	motB	flagellar motor protein MotB	100	2.32E-216	590
ECs_4465	ECs_4465	hypothetical protein	VFG039696 (gb|NP_821048)	T4SS secreted effectors (VF0696)	CBUA0020; CBU_0012*; CBU_0113; CBU_0122; CBU_0183; CBU_0201; CBU_0270; CBU_0295; CBU_0344*; CBU_0372; CBU_0375*; CBU_0469; CBU_0513; CBU_0534; CBU_0590;	setA	Coxiella burnetii RSA 493	Effector delivery system	coxFIC1	Coxiella Dot/Icm type IVB secretion system	33.8	6.96E-21	93.2

CBU_0635; CBU_0637; CBU_0820*; CBU_1048*; CBU_1079; CBU_1107*; CBU_1150*; CBU_1198; CBU_1268; CBU_1349; CBU_1370; CBU_1409; CBU_1434; CBU_1493; CBU_1495*; CBU_1525*; CBU_1530; CBU_1566; CBU_1576; CBU_1594; CBU_1607; CBU_1614; CBU_1639; CBU_1665; CBU_1677; CBU_1685; CBU_1752; CBU_1754; CBU_1789; CBU_1790; CBU_1794; CBU_1818; CBU_1819; CBU_1863; CBU_2016; CBU_2028; CBU_2056; CBU_2059*; CBU_2076; AnkA; AnkB; AnkF; AnkG (Interacts with host protein p32 to block apoptosis.); AnkH; AnkI; AnkM/cig58; AnkP; Cem1; Cem12; Cem13; Cem3; Cem4; Cem6; Cem9; CetCb1; CetCb2; CetCb3; CetCB4; CetCb5; CetCb6; CirA/coxCC1 (Phosphate transporter family protein.); CirB; CirC/coxDFB1; CoxCC10/cig49; CoxCC11; CoxCC12; CoxCC14; CoxCC15; CoxCC3; CoxCC4; CoxCC5; CoxCC6; CoxCC7/cig44; CoxCC8; CoxDFB3; CoxDFB4 (Surface antigen.); CoxDFB5/cig57; CoxDFB6; CoxFIC1;	translocated effector

					CoxH2/rimL (Acetyltransferase.); CoxH3; CoxH4/cig61; CoxK1 (Protein kinase, putative.); CoxK2; CoxTPR1 (Conserved domain protein.); CoxU1; CoxU2; CpeA; CpeB; CpeC/coxU3 (Hypothetical protein plasmid QpH1.); CpeD; CpeE; CpeF; CpeG; CpeH; CvpA; MceA; PhnB; CBUD_RS05145; CBUD_RS06720*; CBUD_RS08635; CBUD_RS11275; CBUD_RS12405; CBUG_RS02435; CBUK_RS06760								
ECs_3621	ygcN	FAD-dependent oxidoreductase	VFG044382 (gb|AAL65289)	Thioquinolobactin (VF0937)	-	setB	Pseudomonas fluorescens ATCC 17400	Nutritional/Metabolic factor	qbsG	QbsG	28.4	2.90E-07	52.4
ECs_4191	fusA	protein chain elongation factor EF-G	VFG016483 (gb|WP_010875022)	EF-Tu (VF0877)	-	setB	Mycoplasma pneumoniae M129	Adherence	tuf	elongation factor Tu	33.8	1.52E-09	60.5
ECs_3605	cysN	sulfate adenylyltransferase subunit 1	VFG016483 (gb|WP_010875022)	EF-Tu (VF0877)	-	setA	Mycoplasma pneumoniae M129	Adherence	tuf	elongation factor Tu	27.7	1.27E-19	90.5

ECs_4049	infB	translation initiation factor IF-2	VFG016491 (gb|WP_011076858)	EF-Tu (VF0877)	-	setB	Mycoplasma penetrans HF-2	Adherence	tuf	elongation factor Tu	32	7.57E-13	71.2
ECs_2462	ynjD	ABC transporter ATPase	VFG013571 (gb|WP_032821670)	HitABC (VF0268)	HitABC(fbpABC) operon encodes a periplasmic-binding protein-dependent iron transport system necessary for the utilization of iron bound to transferrin or iron chelates	setA	Haemophilus influenzae 86-028NP	Nutritional/Metabolic factor	hitC	iron(III) ABC transporter, ATP-binding protein	37.2	5.37E-33	121
ECs_5206	ytfR	sugar ABC transporter ATPase	VFG013571 (gb|WP_032821670)	HitABC (VF0268)	HitABC(fbpABC) operon encodes a periplasmic-binding protein-dependent iron transport system necessary for the utilization of iron bound to transferrin or iron chelates	setA	Haemophilus influenzae 86-028NP	Nutritional/Metabolic factor	hitC	iron(III) ABC transporter, ATP-binding protein	34.7	9.64E-29	116
ECs_4301	livF	branched-chain amino acid ABC transporter ATPase	VFG013571 (gb|WP_032821670)	HitABC (VF0268)	HitABC(fbpABC) operon encodes a periplasmic-binding protein-dependent iron transport system necessary for the utilization of iron bound to transferrin or iron chelates	setA	Haemophilus influenzae 86-028NP	Nutritional/Metabolic factor	hitC	iron(III) ABC transporter, ATP-binding protein	29.9	7.01E-23	95.1
ECs_1868	sapD	antimicrobial peptide	VFG013571 (gb|WP_032821670)	HitABC (VF0268)	HitABC(fbpABC) operon encodes a periplasmic-binding protein-dependent iron transport	setA	Haemophilus influenzae	Nutritional/Metabolic factor	hitC	iron(III) ABC transporte	25.5	1.91E-14	73.2

		ABC transporter ATPase			system necessary for the utilization of iron bound to transferrin or iron chelates		86-028NP			r, ATP-binding protein			
ECs_0201	metN	DL-methionine transporter subunit	VFG013571 (gb|WP_032821670)	HitABC (VF0268)	HitABC(fbpABC) operon encodes a periplasmic-binding protein-dependent iron transport system necessary for the utilization of iron bound to transferrin or iron chelates	setA	Haemophilus influenzae 86-028NP	Nutritional/Metabolic factor	hitC	iron(III) ABC transporter, ATP-binding protein	31.2	1.38E-34	129
ECs_4420	dppF	dipeptide/heme ABC transporter ATPase	VFG000344 (gb|WP_005693820)	HitABC (VF0268)	HitABC(fbpABC) operon encodes a periplasmic-binding protein-dependent iron transport system necessary for the utilization of iron bound to transferrin or iron chelates	setA	Haemophilus influenzae Rd KW20	Nutritional/Metabolic factor	hitC	iron(III) ABC transporter, ATP-binding protein	27.4	2.83E-28	112
ECs_2655	yecC	ABC transporter ATPase	VFG000344 (gb|WP_005693820)	HitABC (VF0268)	HitABC(fbpABC) operon encodes a periplasmic-binding protein-dependent iron transport system necessary for the utilization of iron bound to transferrin or iron chelates	setA	Haemophilus influenzae Rd KW20	Nutritional/Metabolic factor	hitC	iron(III) ABC transporter, ATP-binding protein	39.3	5.96E-43	149
ECs_0908	gsiA	glutathione ABC transporter ATPase	VFG000344 (gb|WP_005693820)	HitABC (VF0268)	HitABC(fbpABC) operon encodes a periplasmic-binding protein-dependent iron transport system necessary for the utilization of iron bound to transferrin or iron	setA	Haemophilus influenzae Rd KW20	Nutritional/Metabolic factor	hitC	iron(III) ABC transporter, ATP-bindin	31.8	3.07E-28	116

					chelates					g protein			
ECs_0691	gltL	ATP-binding protein of glutamate/aspartate transport system	VFG013572 (gb|WP_080334646)	HitABC (VF0268)	HitABC(fbpABC) operon encodes a periplasmic-binding protein-dependent iron transport system necessary for the utilization of iron bound to transferrin or iron chelates	setA	Haemophilus influenzae PittEE	Nutritional/Metabolic factor	hitC	iron(III) ABC transporter, ATP-binding protein	32.8	2.30E-36	131
ECs_0553	fetA	iron export ABC transporter ATPase	VFG013572 (gb|WP_080334646)	HitABC (VF0268)	HitABC(fbpABC) operon encodes a periplasmic-binding protein-dependent iron transport system necessary for the utilization of iron bound to transferrin or iron chelates	setA	Haemophilus influenzae PittEE	Nutritional/Metabolic factor	hitC	iron(III) ABC transporter, ATP-binding protein	31.8	1.92E-26	104
ECs_4661	pstB	phosphate ABC transporter ATPase	VFG013572 (gb|WP_080334646)	HitABC (VF0268)	HitABC(fbpABC) operon encodes a periplasmic-binding protein-dependent iron transport system necessary for the utilization of iron bound to transferrin or iron chelates	setA	Haemophilus influenzae PittEE	Nutritional/Metabolic factor	hitC	iron(III) ABC transporter, ATP-binding protein	30.2	4.47E-29	112
ECs_0793	modC	molybdate ABC transporter ATPase	VFG013572 (gb|WP_080334646)	HitABC (VF0268)	HitABC(fbpABC) operon encodes a periplasmic-binding protein-dependent iron transport system necessary for the utilization	setA	Haemophilus influenzae PittEE	Nutritional/Metabolic factor	hitC	iron(III) ABC transporter,	27.6	2.94E-32	123

					of iron bound to transferrin or iron chelates					ATP-binding protein			
ECs_2120	lsrA	autoinducer 2 import ATP-binding protein	VFG013192 (gb|WP_012340414)	HitABC (VF0268)	HitABC(fbpABC) operon encodes a periplasmic-binding protein-dependent iron transport system necessary for the utilization of iron bound to transferrin or iron chelates	setA	Haemophilus somnus 2336	Nutritional/Metabolic factor	hitC	iron(III) ABC transporter, ATP-binding protein	33.9	1.37E-24	104
ECs_1292	ECs_1292	ABC transporter ATP-binding protein	VFG013192 (gb|WP_012340414)	HitABC (VF0268)	HitABC(fbpABC) operon encodes a periplasmic-binding protein-dependent iron transport system necessary for the utilization of iron bound to transferrin or iron chelates	setA	Haemophilus somnus 2336	Nutritional/Metabolic factor	hitC	iron(III) ABC transporter, ATP-binding protein	38.1	8.12E-33	121
ECs_3072	yejF	microcin C ABC transporter ATPase	VFG013192 (gb|WP_012340414)	HitABC (VF0268)	HitABC(fbpABC) operon encodes a periplasmic-binding protein-dependent iron transport system necessary for the utilization of iron bound to transferrin or iron chelates	setA	Haemophilus somnus 2336	Nutritional/Metabolic factor	hitC	iron(III) ABC transporter, ATP-binding protein	31	3.93E-26	109
ECs_4346	nikD	nickel ABC transporter ATPase	VFG013192 (gb|WP_012340414)	HitABC (VF0268)	HitABC(fbpABC) operon encodes a periplasmic-binding protein-dependent iron transport system necessary for the utilization of iron bound to transferrin or iron chelates	setA	Haemophilus somnus 2336	Nutritional/Metabolic factor	hitC	iron(III) ABC transporter, ATP-binding protein	28	4.85E-22	93.2

ECs_0420	tauB	taurine ABC transporter ATPase	VFG013192 (gb|WP_012340414)	HitABC (VF0268)	HitABC(fbpABC) operon encodes a periplasmic-binding protein-dependent iron transport system necessary for the utilization of iron bound to transferrin or iron chelates	setA	Haemophilus somnus 2336	Nutritional/Metabolic factor	hitC	iron(III) ABC transporter, ATP-binding protein	41.5	1.26E-41	145
ECs_1746	oppD	oligopeptide ABC transporter ATPase	VFG013192 (gb|WP_012340414)	HitABC (VF0268)	HitABC(fbpABC) operon encodes a periplasmic-binding protein-dependent iron transport system necessary for the utilization of iron bound to transferrin or iron chelates	setA	Haemophilus somnus 2336	Nutritional/Metabolic factor	hitC	iron(III) ABC transporter, ATP-binding protein	30.6	1.24E-21	94
ECs_2660	fliZ	flagellar biosynthesis protein FliZ	VFG043100 (gb|WP_001302103)	Peritrichous flagella (VF1154)	-	setA	Escherichia coli O157:H7 str. EDL933	Motility	fliZ	flagella biosynthesis regulatory protein FliZ	100	4.61E-138	382
ECs_2661	fliA	RNA polymerase sigma 28 factor FilA	VFG043101 (gb|WP_001087467)	Peritrichous flagella (VF1154)	-	setA	Escherichia coli O157:H7 str. EDL933	Motility	fliA	RNA polymerase sigma factor FliA	99.6	1.47E-164	454
ECs_2662	fliC	flagellin	VFG043102 (gb|WP_000079723)	Peritrichous flagella	-	setA	Escherichia coli O157:H7	Motility	Z_RS14145	FliC/FljB family flagellin	99.8	0	954

				(VF1154)			str. EDL933						
ECs_2663	fliD	flagellar filament capping protein	VFG043103 (gb|WP_000146755)	Peritrichous flagella (VF1154)	-	setA	Escherichia coli O157:H7 str. EDL933	Motility	fliD	flagellar filament capping protein FliD	100	2.04E-280	766
ECs_2664	fliS	flagellar protein potentiates polymerization	VFG043104 (gb|WP_000270418)	Peritrichous flagella (VF1154)	-	setA	Escherichia coli O157:H7 str. EDL933	Motility	fliS	flagellar export chaperone FliS	99.3	2.70E-87	250
ECs_2665	fliT	flagellar synthesis and assembly chaperone	VFG043105 (gb|WP_001015033)	Peritrichous flagella (VF1154)	-	setA	Escherichia coli O157:H7 str. EDL933	Motility	fliT	flagella biosynthesis regulatory protein FliT	100	3.21E-81	233
ECs_3923	tolC	transport channel	VFG044140 (gb|WP_002212186)	HasA-type hemophore-mediated heme uptake system (VF1029)	-	setB	Yersinia pestis CO92	Nutritional/Metabolic factor	tolC	outer membrane channel protein TolC	74.3	3.25E-228	635
ECs_0540	ECs_0540	outer	VFG044140	HasA-type	-	setA	Yersinia	Nutritiona	tolC	outer	22	2.98E-	65.1

		membrane transport protein	(gb|WP_002212186)	hemophore-mediated heme uptake system (VF1029)			pestis CO92	l/Metabolic factor		membrane channel protein TolC		11	
ECs_3881	hybA	Hydrogenase-2 operon protein HybA precursor	VFG026784 (gb|YP_005359856)	Nitrate reductase (VF0302)	Nitrate respiration helps the bacteria to survive in O2-depleted areas of inflammatory or necrotic tissue	setA	Mycobacterium tuberculosis RGTB327	Nutritional/Metabolic factor	narH	respiratory nitrate reductase subunit beta narH	33.8	8.96E-16	77.8
ECs_0980	dmsB	anaerobic dimethyl sulfoxide reductase reductase subunit B	VFG026784 (gb|YP_005359856)	Nitrate reductase (VF0302)	Nitrate respiration helps the bacteria to survive in O2-depleted areas of inflammatory or necrotic tissue	setA	Mycobacterium tuberculosis RGTB327	Nutritional/Metabolic factor	narH	respiratory nitrate reductase subunit beta narH	43.4	1.15E-22	94.7
ECs_3383	ECs_3383	anaerobic dimethyl sulfoxide reductase chain B	VFG026784 (gb|YP_005359856)	Nitrate reductase (VF0302)	Nitrate respiration helps the bacteria to survive in O2-depleted areas of inflammatory or necrotic tissue	setA	Mycobacterium tuberculosis RGTB327	Nutritional/Metabolic factor	narH	respiratory nitrate reductase subunit beta narH	32.8	2.90E-17	79.3
ECs_2295	ynfG_2	oxidoreductase	VFG026784 (gb|YP_005359856)	Nitrate reductase (VF0302)	Nitrate respiration helps the bacteria to survive in O2-depleted areas of inflammatory or necrotic tissue	setA	Mycobacterium tuberculosis RGTB327	Nutritional/Metabolic factor	narH	respiratory nitrate reductase subunit	43.4	2.14E-22	94

ECs_2378	ydhX	oxidoreductase Fe-S subunit	VFG026784 (gb|YP_005359856)	Nitrate reductase (VF0302)	Nitrate respiration helps the bacteria to survive in O2-depleted areas of inflammatory or necrotic tissue	setA	Mycobacterium tuberculosis RGTB327	Nutritional/Metabolic factor	narH	beta narH respiratory nitrate reductase subunit beta narH	37.4	9.45E-15	72.4
ECs_5067	ECs_5067	regulatory protein	VFG038216 (gb|WP_000076440)	BfmRS (VF0463)	-	setA	Acinetobacter baumannii 1656-2	Regulation	bfmR	biofilm-controlling response regulator	40.8	5.65E-44	147
ECs_4838	cpxR	two-component regulatory system response regulator CpxR	VFG038216 (gb|WP_000076440)	BfmRS (VF0463)	-	setA	Acinetobacter baumannii 1656-2	Regulation	bfmR	biofilm-controlling response regulator	39	4.01E-52	168
ECs_2314	rstA	two-component regulatory system response regulator rstA	VFG038216 (gb|WP_000076440)	BfmRS (VF0463)	-	setA	Acinetobacter baumannii 1656-2	Regulation	bfmR	biofilm-controlling response regulator	44.8	1.10E-64	201
ECs_4247	ompR	two-component	VFG038216 (gb|WP_00	BfmRS (VF0463)	-	setA	Acinetobacter	Regulation	bfmR	biofilm-controlling	41.7	5.61E-54	173

		regulatory system response regulator OmpR	0076440)				baumannii 1656-2			response regulator			
ECs_5359	arcA	two-component regulatory system response regulator	VFG041119 (gb|WP_004528788)	T6SS-1 (VF0429)	Essential for virulence and plays an important role in the intracellular lifestyle of B. pseudomallei; In B. mallei, the T6SS cluster homologous to B. pseudomallei T6SS-1, is important for actin-based motility, multinucleated giant cell formation, intracellular growth in murine macrophages, and virulence in hamsters	setA	Burkholderia pseudomallei K96243	Effector delivery system	BPS_RS26875	response regulator VirG	34.6	2.61E-41	141
ECs_1150	torR	two-component regulatory system response regulator TorR	VFG041119 (gb|WP_004528788)	T6SS-1 (VF0429)	Essential for virulence and plays an important role in the intracellular lifestyle of B. pseudomallei; In B. mallei, the T6SS cluster homologous to B. pseudomallei T6SS-1, is important for actin-based motility, multinucleated giant cell formation, intracellular growth in murine macrophages, and virulence in hamsters	setA	Burkholderia pseudomallei K96243	Effector delivery system	BPS_RS26875	response regulator VirG	36.4	1.95E-38	133
ECs_1737	rssB	response regulator	VFG009862 (gb|WP_071498255)	MprAB (VF0298)	Required for establishment and maintenance of persistent infection	setA	Mycobacterium ulcerans	Regulation	mprA	response regulator transcript	37.6	1.01E-17	80.9

							Agy99			ion factor			
ECs_0609	cusR	two-component regulatory system response regulator CusR	VFG009862 (gb|WP_071498255)	MprAB (VF0298)	Required for establishment and maintenance of persistent infection	setA	Mycobacterium ulcerans Agy99	Regulation	mprA	response regulator transcription factor	44	1.00E-57	182
ECs_5064	mdtN	membrane fusion protein of efflux pump	VFG036944 (gb|WP_014573758)	FarAB (VF0450)	Mediates the resistance to antimicrobial long-chain fatty acids	setA	Neisseria meningitidis 8013	Antimicrobial activity/Competitive advantage	farA	fatty acid efflux system protein FarA	30.4	7.38E-29	114
ECs_0873	ybhG	membrane fusion protein component of efflux pump	VFG036944 (gb|WP_014573758)	FarAB (VF0450)	Mediates the resistance to antimicrobial long-chain fatty acids	setA	Neisseria meningitidis 8013	Antimicrobial activity/Competitive advantage	farA	fatty acid efflux system protein FarA	27.7	6.07E-12	65.9
ECs_3247	emrK	multidrug resistance efflux pump membrane fusion protein	VFG036944 (gb|WP_014573758)	FarAB (VF0450)	Mediates the resistance to antimicrobial long-chain fatty acids	setA	Neisseria meningitidis 8013	Antimicrobial activity/Competitive advantage	farA	fatty acid efflux system protein FarA	42.5	3.09E-87	269

ECs_2353	ydhJ	membrane fusion protein of YdhJK efflux pump	VFG036944 (gb|WP_014573758)	FarAB (VF0450)	Mediates the resistance to antimicrobial long-chain fatty acids	setA	Neisseria meningitidis 8013	Antimicrobial activity/Competitive advantage	farA	fatty acid efflux system protein FarA	28.1	4.10E-23	97.4
ECs_4114	aaeA	p-hydroxybenzoic acid efflux system component	VFG036944 (gb|WP_014573758)	FarAB (VF0450)	Mediates the resistance to antimicrobial long-chain fatty acids	setA	Neisseria meningitidis 8013	Antimicrobial activity/Competitive advantage	farA	fatty acid efflux system protein FarA	33.8	2.70E-12	66.6
ECs_3547	emrA	multidrug efflux system protein	VFG036944 (gb|WP_014573758)	FarAB (VF0450)	Mediates the resistance to antimicrobial long-chain fatty acids	setA	Neisseria meningitidis 8013	Antimicrobial activity/Competitive advantage	farA	fatty acid efflux system protein FarA	44.4	4.64E-96	291
ECs_5061	fdhF	formate dehydrogenase-H%2C selenopolypeptide subunit	VFG027924 (gb|WP_008259449)	NuoG (VF0833)	-	setB	Mycobacterium yongonense 05-1390	Immune modulation	nuoG	NADH-quinone oxidoreductase subunit G	24.6	3.69E-24	108
ECs_3095	napA	periplasmic nitrate	VFG027911 (gb|WP_005061212)	NuoG (VF0833)	-	setB	Mycobacterium abscessus	Immune modulation	nuoG	NADH-quinone oxidoreduc	28.1	4.25E-11	66.6

		reductase NapA					subsp. bolletii str. GO 06			tase subunit G			
ECs_3167	nuoG	NADH-quinone oxidoreductase subunit G	VFG027911 (gb|WP_005061212)	NuoG (VF0833)	-	setB	Mycobacterium abscessus subsp. bolletii str. GO 06	Immune modulation	nuoG	NADH-quinone oxidoreductase subunit G	31.7	5.48E-47	180
ECs_2483	ydjJ	Zn-dependent NAD(P)-binding oxidoreductase	VFG030301 (gb|WP_012392129)	MymA operon (VF0840)	-	setB	Mycobacterium marinum M	Immune modulation	adhD	NDMA-dependent alcohol dehydrogenase	27.8	2.21E-27	110
ECs_2082	adhP	alcohol dehydrogenase	VFG030301 (gb|WP_012392129)	MymA operon (VF0840)	-	setA	Mycobacterium marinum M	Immune modulation	adhD	NDMA-dependent alcohol dehydrogenase	29	7.72E-33	125
ECs_2894	gatD	galactitol-1-phosphate dehydrogenase	VFG030301 (gb|WP_012392129)	MymA operon (VF0840)	-	setB	Mycobacterium marinum M	Immune modulation	adhD	NDMA-dependent alcohol dehydrogenase	25.5	1.84E-19	88.2
ECs_5318	lgoD	L-galacto	VFG030301	MymA	-	setA	Mycobacter	Immune	adhD	NDMA-depen	24.9	9.14E-	89

		nate oxidoreductase	(gb|WP_012392129)	operon (VF0840)			ium marinum M	modulation		dent alcohol dehydrogenase		20	
ECs_0411	frmA	S-(hydroxymethyl)glutathione dehydrogenase	VFG030305 (gb|WP_016343680)	MymA operon (VF0840)	-	setB	Mycobacterium abscessus subsp. bolletii 50594	Immune modulation	adhD	NDMA-dependent alcohol dehydrogenase	36.7	5.32E-66	213
ECs_2485	ydjL	Zn-dependent NAD(P)-binding oxidoreductase	VFG030295 (gb|WP_011742363)	MymA operon (VF0840)	-	setB	Mycobacterium ulcerans Agy99	Immune modulation	adhD	Zn-dependent alcohol dehydrogenase	25.4	5.53E-13	69.3
ECs_0379	yahK	broad specificity NADPH-dependent aldehyde reductase	VFG030295 (gb|WP_011742363)	MymA operon (VF0840)	-	setB	Mycobacterium ulcerans Agy99	Immune modulation	adhD	Zn-dependent alcohol dehydrogenase	25.9	9.38E-17	80.5
ECs_0647	ybdR	Zn-dependent NAD(P)-bi	VFG030286 (gb|NP_217602)	MymA operon (VF0840)	-	setB	Mycobacterium tuberculos	Immune modulation	adhD	alcohol dehydrogenase D	26.3	1.86E-17	83.2

		nding oxidoreductase					is H37Rv						
ECs_4551	escG	T3SS component EscG	VFG000795 (gb|WP_000245816)	TTSS (VF0191)	Injects Tir and other effector molecules directly into the host cell. Effector molecules activate cell-signaling pathways, causing alterations in the host cell cytoskeleton and resulting in the depolymerization of actin and the loss of microvilli	setA	Escherichia coli O157:H7 str. EDL933	Effector delivery system	escG	chaperone for EscF	100	2.31E-65	191
ECs_4724	wecE	TDP-4-oxo-6-deoxy-D-glucose transaminase	VFG000036 (gb|WP_010929614)	LPS (VF0033)	Prevents clearance of the organism by host surfactant protein; confers protection to the bacterium from complement-mediated cell lysis	setA	Bordetella pertussis Tohama I	Immune modulation	bplC	lipopolysaccharide biosynthesis protein	34.5	7.74E-44	155
ECs_3141	arnB	uridine 5'-(beta-1-threo-pentapyranosyl-4-ulose diphosphate) aminotransferase	VFG011156 (gb|WP_010927317)	LPS (VF0033)	Prevents clearance of the organism by host surfactant protein; confers protection to the bacterium from complement-mediated cell lysis	setA	Bordetella parapertussis 12822	Immune modulation	bplF	lipopolysaccharide biosynthesis protein	40.8	3.91E-94	287

ECs_4124	csrD	regulatory protein CsrD	VFG006925 (gb|WP_011261157)	MSHA pili (VF0515)	Plays important role in biofilm formation; crucial for surface selection, irreversible attachment, and ultimately microcolony formation	setA	Vibrio fischeri ES114	Adherence	mshH	MSHA biogenesis protein MshH	32.3	7.74E-111	347
ECs_3365	yfgF	cyclic-di-GMP phosphodiesterase	VFG006922 (gb|WP_011106082)	MSHA pili (VF0515)	Plays important role in biofilm formation; crucial for surface selection, irreversible attachment, and ultimately microcolony formation	setA	Vibrio parahaemolyticus RIMD 2210633	Adherence	mshH	MSHA biogenesis protein MshH	19.3	5.53E-08	56.2
ECs_1360	ECs_1360	Iha adhesin	VFG001731 (gb|WP_001223352)	C3610 (VF1157)	-	setA	Escherichia coli CFT073	Adherence	iha	bifunctional siderophore receptor/adhesin Iha	99.9	0	1387
ECs_4897	btuB	vitamin B12/cobalamin outer membrane transporter	VFG001731 (gb|WP_001223352)	C3610 (VF1157)	-	setA	Escherichia coli CFT073	Adherence	iha	bifunctional siderophore receptor/adhesin Iha	26.6	1.55E-45	171
ECs_3047	cirA	colicin IA outer membrane receptor and	VFG007263 (gb|WP_000086048)	Enterobactin receptor IrgA (VF0628)	-	setA	Vibrio cholerae O1 biovar El Tor str. N16961	Nutritional/Metabolic factor	irgA	ligand-gated channel protein	36.9	1.32E-118	368

		translocator											
ECs_1693	ECs_1693	TonB dependent outer membrane receptor	VFG007263 (gb|WP_000086048)	Enterobactin receptor IrgA (VF0628)	-	setA	Vibrio cholerae O1 biovar El Tor str. N16961	Nutritional/Metabolic factor	irgA	ligand-gated channel protein	20.8	1.51E-11	67.4
ECs_2792	yoeE	hypothetical protein	VFG007263 (gb|WP_000086048)	Enterobactin receptor IrgA (VF0628)	-	setA	Vibrio cholerae O1 biovar El Tor str. N16961	Nutritional/Metabolic factor	irgA	ligand-gated channel protein	25	6.42E-20	94.4
ECs_4198	fkpA	FKBP-type peptidyl-prolyl cis-trans isomerase	VFG045705 (gb|WP_003636663)	Mip (VF0153)	Necessary for intracellular survival	setA	Legionella longbeachae NSW150	Post-translational modification	mip	macrophage infectivity potentiator Mip	36.2	1.98E-36	129
ECs_5185	fklB	FKBP-type peptidyl-prolyl cis-trans isomerase	VFG045705 (gb|WP_003636663)	Mip (VF0153)	Necessary for intracellular survival	setA	Legionella longbeachae NSW150	Post-translational modification	mip	macrophage infectivity potentiator Mip	39.6	5.37E-44	146
ECs_0560	rhsD	type IV secretion protein Rhs	VFG049917 (gb|WP_000508696)	T6SS (VF0579)	Plays a role in interbacterial competition and host colonization	setA	Shigella sonnei Ss046	Effector delivery system	rhs/PAAR	Type VI secretion system protein,	65.4	0	1480

										PAAR family			
ECs_2061	rhsC	type IV secretion protein Rhs	VFG049917 (gb|WP_000508696)	T6SS (VF0579)	Plays a role in interbacterial competition and host colonization	setA	Shigella sonnei Ss046	Effector delivery system	rhs/PAAR	Type VI secretion system protein, PAAR family	70.1	0	1474
ECs_0729	rhsC	type IV secretion protein Rhs	VFG049917 (gb|WP_000508696)	T6SS (VF0579)	Plays a role in interbacterial competition and host colonization	setA	Shigella sonnei Ss046	Effector delivery system	rhs/PAAR	Type VI secretion system protein, PAAR family	65.8	0	1384
ECs_0237	rhsC	type IV secretion protein Rhs	VFG049917 (gb|WP_000508696)	T6SS (VF0579)	Plays a role in interbacterial competition and host colonization	setA	Shigella sonnei Ss046	Effector delivery system	rhs/PAAR	Type VI secretion system protein, PAAR family	86.7	0	1839
ECs_0242	ECs_0242	type IV secretion protein Rhs	VFG049917 (gb|WP_000508696)	T6SS (VF0579)	Plays a role in interbacterial competition and host colonization	setA	Shigella sonnei Ss046	Effector delivery system	rhs/PAAR	Type VI secretion system protein, PAAR family	79	1.63E-240	702
ECs_4470	rhsC	type IV secretion protein Rhs	VFG049917 (gb|WP_000508696)	T6SS (VF0579)	Plays a role in interbacterial competition and host colonization	setA	Shigella sonnei Ss046	Effector delivery system	rhs/PAAR	Type VI secretion system protein, PAAR family	65.6	0	1375

ECs_4864	rhsD	type IV secretion protein Rhs	VFG049917 (gb|WP_000508696)	T6SS (VF0579)	Plays a role in interbacterial competition and host colonization	setA	Shigella sonnei Ss046	Effector delivery system	rhs/PAAR	Type VI secretion system protein, PAAR family	65.8	0	1380
ECs_0567	allR	glyoxylate-inducible transcriptional repressor of all and gcl operons	VFG049118 (gb|WP_012737529)	Allantion utilization (VF0572)	Providing a nitrogen source to increase virulence in K. pneumoniae at certain sites of infection	setA	Klebsiella pneumoniae subsp. pneumoniae NTUH-K2044	Nutritional/Metabolic factor	allR	DNA-binding transcriptional repressor AllR	86.2	9.53E-168	464
ECs_2537	kdgR	KDG regulon transcriptional repressor	VFG049118 (gb|WP_012737529)	Allantion utilization (VF0572)	Providing a nitrogen source to increase virulence in K. pneumoniae at certain sites of infection	setA	Klebsiella pneumoniae subsp. pneumoniae NTUH-K2044	Nutritional/Metabolic factor	allR	DNA-binding transcriptional repressor AllR	28.1	1.89E-26	103
ECs_4936	iclR	transcriptional repressor	VFG049118 (gb|WP_012737529)	Allantion utilization (VF0572)	Providing a nitrogen source to increase virulence in K. pneumoniae at certain sites of infection	setA	Klebsiella pneumoniae subsp. pneumoniae NTUH-K2044	Nutritional/Metabolic factor	allR	DNA-binding transcriptional repressor AllR	40.5	6.41E-65	204

ECs_0566	allA	ureidoglycolate hydrolase	VFG049116 (gb|WP_012737528)	Allantion utilization (VF0572)	Providing a nitrogen source to increase virulence in K. pneumoniae at certain sites of infection	setA	Klebsiella pneumoniae subsp. pneumoniae NTUH-K2044	Nutritional/Metabolic factor	allA	ureidoglycolate hydrolase	74.4	6.09E-91	261
ECs_1560	espX7	T3SS effector-like protein EspX	VFG034888 (gb|NP_309587)	TTSS secreted effectors (VF1110)	Cif (Deamidase. Induces cytopathic effects of actin stress fiber formation and cell cycle arrest.); EspB (Pore formation, actin disruption, microvilli effacement, anti-phagocytosis.); EspF (Inducing degradation of the aniapoptic protein AbcF2, tight junction disruption, microvilli effacement and elongation, mitochondrial dysfunction, N-WASP activation, SGLT-1 inactivation, pedestal maturation, inhibition of NHE3 activity, membrane remodelling; targets and disrupts the nucleolus late in infection, which is temporally controlled by host mitochondria.); EspFu/tccP (Inducing degradation of the aniapoptic protein AbcF2, tight junction disruption, microvilli effacement and elongation, mitochondrial dysfunction, N-WASP	setA	Escherichia coli O157:H7 str. Sakai	Effector delivery system	espX7/nleL	Type III secretion system effector NleL, putative E3 ubiquitin ligase	100	0	1411

activation, SGLT-1 inactivation, pedestal maturation, inhibition of NHE3 activity, membrane remodelling; targets and disrupts the nucleolus late in infection, which is temporally controlled by host mitochondria.); EspG (TBC-like GTPase activating protein. Efficiently catalyzes GTP hydrolysis in Rab1 to disrupt of Rab1-mediated ER-to-Golgi trafficking.); EspH (First bacterial effector acting directly on RhoGEFs, EspH directly binds to the DH-PH domain in RhoGEFs to disrupt RhoGEF-Rho signaling; critical for inhibiting macrophage phagocytosis.); EspJ (Inhibit both IgG- and complement receptor-mediated phagocytosis.); EspK; EspL1; EspL2 (Cysteine protease. Bounds F-actin-aggregating annexin 2 directly to increase annexin 2's ability to aggregate Tir-induced F-actin; block necroptosis and in flammation.); EspL4; EspM1 (GEF. Activates the RhoA signaling pathway and induce the formation of stress

fibres; inhibit pedestal formation and induce tight junction mislocalization.); EspM2 (GEF. Activates the RhoA signaling pathway and induce the formation of stress fibres; inhibit pedestal formation and induce tight junction mislocalization.); EspN; EspO1-1; EspO1-2; EspR1; EspR3; EspR4; EspT (GEF. Activates Rac1 and Cdc42 leading to formation of membrane ruffles and lamellipodia; induces membrane ruffles to facilitate bacterial invasion into non-phagocytic cells in a process involving Rac1 and Wave2.); EspW; EspX1; EspX2; EspX4; EspX5; EspX6; EspX7/nleL (E3 ubiquitin ligase, HECT-like. Modulates pedestal formation.); EspY1; EspY2; EspY3; EspY4; EspY5; Map (GEF. Mimics the host Dbl and catalyses the exchange of GDP for GTP in Cdc42, involved in effacement, SGLT1 inhibition, formation of filopodia and disruption of mitochondrial function.); NleA/espI (Disruption of tight

junctions by inhibition of host cell protein trafficking through COPII-dependent pathways.); NleB1 (Blocks translocation of the p65 and to the host cell nucleus to inhibit NF-<kappa>B pathway, but NleE and NleB act at different points in the NF-<kappa>B signaling pathway.); NleB2 (May also have anti-inflammatory activity.); NleC (Metalloprotease. Zn-dependent endopeptidases that specifically clip and inactivate RelA (p65), thus blocking NF-<kappa>B pathway.); NleD (Metalloprotease. Zn-dependent endopeptidases that specifically clip and inactivate JNK and p38, thus blocking AP-1 pathway.); NleE (PMN tran-epithelial migration; blocks translocation of the p65 to the host cell nucleus by preventing I<kappa>B degradation to inhibit NF-<kappa>B pathway.); NleF; NleG-1; NleG2-2; NleG2-3; NleG2-4; NleG5-1; NleG5-2; NleG6-1; NleG6-2; NleG6-3; NleG7 (U-box type E3 ubiquitin ligases.); NleG8-2; NleH1 (Ser/Thr

protein kinase. Binds directly to a subunit of NF-<kappa>B, the ribosomal protein S3 (RPS3), reducing the nuclear abundance of RPS3 to dampen host transcriptional outputs; interact with Bax inhibitor-1 to block apoptosis.); NleH2 (Putative kinase. Attenuates NF-<kappa>B pathway.); SepZ/espZ (EspZ interacts with CD98 in host cell membranes to promote host cell survival, therefore provide the pathogen with valuable time to colonize efficiently prior to dissemination.); TccP2; Tir (Mimics host immunoreceptor tyrosine-based inhibition motifs (ITIMs), also see helicobacter CagA. EHEC Tir lacks the Nck binding site. Conserved NPY (Asn-Pro-Tyr) motif recruits the adaptor protein IRTKS and/or IRSp53. IRTKS/IRSp53 link Tir and TccP/EspFu, which in turn activates N-WASP; Receptor for intimin; effacement; SGLT1 inhibition; recruits SHIP2 to control actin-pedestal morphology; maintains the integrity of the

ECs_0876	espX2	T3SS effector-like protein EspX	VFG034888 (gb|NP_309587)	TTSS secreted effectors (VF1110)	epithelium by keeping the destructive activity of EspG and EspG2 in check.) Cif (Deamidase. Induces cytopathic effects of actin stress fiber formation and cell cycle arrest.); EspB (Pore formation, actin disruption, microvilli effacement, anti-phagocytosis.); EspF (Inducing degradation of the aniapoptic protein AbcF2, tight junction disruption, microvilli effacement and elongation, mitochondrial dysfunction, N-WASP activation, SGLT-1 inactivation, pedestal maturation, inhibition of NHE3 activity, membrane remodelling; targets and disrupts the nucleolus late in infection, which is temporally controlled by host mitochondria.); EspFu/tccP (Inducing degradation of the aniapoptic protein AbcF2, tight junction disruption, microvilli effacement and elongation, mitochondrial dysfunction, N-WASP activation, SGLT-1 inactivation, pedestal maturation, inhibition of NHE3 activity, membrane remodelling;	setA	Escherichia coli O157:H7 str. Sakai	Effector delivery system	espX7/nleL	Type III secretion system effector NleL, putative E3 ubiquitin ligase	23.2	4.87E-13	72.4

targets and disrupts the nucleolus late in infection, which is temporally controlled by host mitochondria.); EspG (TBC-like GTPase activating protein. Efficiently catalyzes GTP hydrolysis in Rab1 to disrupt of Rab1-mediated ER-to-Golgi trafficking.); EspH (First bacterial effector acting directly on RhoGEFs, EspH directly binds to the DH-PH domain in RhoGEFs to disrupt RhoGEF-Rho signaling; critical for inhibiting macrophage phagocytosis.); EspJ (Inhibit both IgG- and complement receptor-mediated phagocytosis.); EspK; EspL1; EspL2 (Cysteine protease. Bounds F-actin-aggregating annexin 2 directly to increase annexin 2's ability to aggregate Tir-induced F-actin; block necroptosis and in flammation.); EspL4; EspM1 (GEF. Activates the RhoA signaling pathway and induce the formation of stress fibres; inhibit pedestal formation and induce tight junction mislocalization.); EspM2 (GEF.

Activates the RhoA signaling pathway and induce the formation of stress fibres; inhibit pedestal formation and induce tight junction mislocalization.); EspN; EspO1-1; EspO1-2; EspR1; EspR3; EspR4; EspT (GEF. Activates Rac1 and Cdc42 leading to formation of membrane ruffles and lamellipodia; induces membrane ruffles to facilitate bacterial invasion into non-phagocytic cells in a process involving Rac1 and Wave2.); EspW; EspX1; EspX2; EspX4; EspX5; EspX6; EspX7/nleL (E3 ubiquitin ligase, HECT-like. Modulates pedestal formation.); EspY1; EspY2; EspY3; EspY4; EspY5; Map (GEF. Mimics the host Dbl and catalyses the exchange of GDP for GTP in Cdc42, involved in effacement, SGLT1 inhibition, formation of filopodia and disruption of mitochondrial function.); NleA/espI (Disruption of tight junctions by inhibition of host cell protein trafficking through COPII-dependent pathways.); NleB1

(Blocks translocation of the p65 and to the host cell nucleus to inhibit NF-<kappa>B pathway, but NleE and NleB act at different points in the NF-<kappa>B signaling pathway.); NleB2 (May also have anti-inflammatory activity.); NleC (Metalloprotease. Zn-dependent endopeptidases that specifically clip and inactivate RelA (p65), thus blocking NF-<kappa>B pathway.); NleD (Metalloprotease. Zn-dependent endopeptidases that specifically clip and inactivate JNK and p38, thus blocking AP-1 pathway.); NleE (PMN tran-epithelial migration; blocks translocation of the p65 to the host cell nucleus by preventing I<kappa>B degradation to inhibit NF-<kappa>B pathway.); NleF; NleG-1; NleG2-2; NleG2-3; NleG2-4; NleG5-1; NleG5-2; NleG6-1; NleG6-2; NleG6-3; NleG7 (U-box type E3 ubiquitin ligases.); NleG8-2; NleH1 (Ser/Thr protein kinase. Binds directly to a subunit of NF-<kappa>B, the ribosomal protein S3 (RPS3), reducing the

					nuclear abundance of RPS3 to dampen host transcriptional outputs; interact with Bax inhibitor-1 to block apoptosis.); NleH2 (Putative kinase. Attenuates NF-<kappa>B pathway.); SepZ/espZ (EspZ interacts with CD98 in host cell membranes to promote host cell survival, therefore provide the pathogen with valuable time to colonize efficiently prior to dissemination.); TccP2; Tir (Mimics host immunoreceptor tyrosine-based inhibition motifs (ITIMs), also see helicobacter CagA. EHEC Tir lacks the Nck binding site. Conserved NPY (Asn-Pro-Tyr) motif recruits the adaptor protein IRTKS and/or IRSp53. IRTKS/IRSp53 link Tir and TccP/EspFu, which in turn activates N-WASP; Receptor for intimin; effacement; SGLT1 inhibition; recruits SHIP2 to control actin-pedestal morphology; maintains the integrity of the epithelium by keeping the destructive activity of EspG and EspG2 in check.)								
ECs_1561	espN	T3SS	VFG034796	TTSS	Cif (Deamidase. Induces cytopathic	setA	Escherichi	Effector	espN	Type III	100	0	2243

secreted effector EspN	(gb|NP_309588)	secreted effectors (VF1110)	effects of actin stress fiber formation and cell cycle arrest.); EspB (Pore formation, actin disruption, microvilli effacement, anti-phagocytosis.); EspF (Inducing degradation of the aniapoptic protein AbcF2, tight junction disruption, microvilli effacement and elongation, mitochondrial dysfunction, N-WASP activation, SGLT-1 inactivation, pedestal maturation, inhibition of NHE3 activity, membrane remodelling; targets and disrupts the nucleolus late in infection, which is temporally controlled by host mitochondria.); EspFu/tccP (Inducing degradation of the aniapoptic protein AbcF2, tight junction disruption, microvilli effacement and elongation, mitochondrial dysfunction, N-WASP activation, SGLT-1 inactivation, pedestal maturation, inhibition of NHE3 activity, membrane remodelling; targets and disrupts the nucleolus late in infection, which is temporally controlled by host mitochondria.);	a coli O157:H7 str. Sakai	delivery system	secretion system effector EspN

EspG (TBC-like GTPase activating protein. Efficiently catalyzes GTP hydrolysis in Rab1 to disrupt of Rab1-mediated ER-to-Golgi trafficking.); EspH (First bacterial effector acting directly on RhoGEFs, EspH directly binds to the DH-PH domain in RhoGEFs to disrupt RhoGEF-Rho signaling; critical for inhibiting macrophage phagocytosis.); EspJ (Inhibit both IgG- and complement receptor-mediated phagocytosis.); EspK; EspL1; EspL2 (Cysteine protease. Bounds F-actin-aggregating annexin 2 directly to increase annexin 2's ability to aggregate Tir-induced F-actin; block necroptosis and in flammation.); EspL4; EspM1 (GEF. Activates the RhoA signaling pathway and induce the formation of stress fibres; inhibit pedestal formation and induce tight junction mislocalization.); EspM2 (GEF. Activates the RhoA signaling pathway and induce the formation of stress fibres; inhibit pedestal formation and

induce tight junction mislocalization.); EspN; EspO1-1; EspO1-2; EspR1; EspR3; EspR4; EspT (GEF. Activates Rac1 and Cdc42 leading to formation of membrane ruffles and lamellipodia; induces membrane ruffles to facilitate bacterial invasion into non-phagocytic cells in a process involving Rac1 and Wave2.); EspW; EspX1; EspX2; EspX4; EspX5; EspX6; EspX7/nleL (E3 ubiquitin ligase, HECT-like. Modulates pedestal formation.); EspY1; EspY2; EspY3; EspY4; EspY5; Map (GEF. Mimics the host Dbl and catalyses the exchange of GDP for GTP in Cdc42, involved in effacement, SGLT1 inhibition, formation of filopodia and disruption of mitochondrial function.); NleA/espI (Disruption of tight junctions by inhibition of host cell protein trafficking through COPII-dependent pathways.); NleB1 (Blocks translocation of the p65 and to the host cell nucleus to inhibit NF-<kappa>B pathway, but NleE and NleB

act at different points in the NF-<kappa>B signaling pathway.); NleB2 (May also have anti-inflammatory activity.); NleC (Metalloprotease. Zn-dependent endopeptidases that specifically clip and inactivate RelA (p65), thus blocking NF-<kappa>B pathway.); NleD (Metalloprotease. Zn-dependent endopeptidases that specifically clip and inactivate JNK and p38, thus blocking AP-1 pathway.); NleE (PMN tran-epithelial migration; blocks translocation of the p65 to the host cell nucleus by preventing I<kappa>B degradation to inhibit NF-<kappa>B pathway.); NleF; NleG-1; NleG2-2; NleG2-3; NleG2-4; NleG5-1; NleG5-2; NleG6-1; NleG6-2; NleG6-3; NleG7 (U-box type E3 ubiquitin ligases.); NleG8-2; NleH1 (Ser/Thr protein kinase. Binds directly to a subunit of NF-<kappa>B, the ribosomal protein S3 (RPS3), reducing the nuclear abundance of RPS3 to dampen host transcriptional outputs; interact with Bax inhibitor-1 to block

					apoptosis.); NleH2 (Putative kinase. Attenuates NF-<kappa>B pathway.); SepZ/espZ (EspZ interacts with CD98 in host cell membranes to promote host cell survival, therefore provide the pathogen with valuable time to colonize efficiently prior to dissemination.); TccP2; Tir (Mimics host immunoreceptor tyrosine-based inhibition motifs (ITIMs), also see helicobacter CagA. EHEC Tir lacks the Nck binding site. Conserved NPY (Asn-Pro-Tyr) motif recruits the adaptor protein IRTKS and/or IRSp53. IRTKS/IRSp53 link Tir and TccP/EspFu, which in turn activates N-WASP; Receptor for intimin; effacement; SGLT1 inhibition; recruits SHIP2 to control actin-pedestal morphology; maintains the integrity of the epithelium by keeping the destructive activity of EspG and EspG2 in check.)								
ECs_1567	espO1-1	T3SS secreted effector EspO	VFG034803 (gb|NP_309594)	TTSS secreted effectors (VF1110)	Cif (Deamidase. Induces cytopathic effects of actin stress fiber formation and cell cycle arrest.); EspB (Pore formation, actin	setA	Escherichia coli O157:H7 str. Sakai	Effector delivery system	espO1-1	type III secreted effector	100	1.25E-64	189

disruption, microvilli effacement, anti-phagocytosis.); EspF (Inducing degradation of the aniapoptic protein AbcF2, tight junction disruption, microvilli effacement and elongation, mitochondrial dysfunction, N-WASP activation, SGLT-1 inactivation, pedestal maturation, inhibition of NHE3 activity, membrane remodelling; targets and disrupts the nucleolus late in infection, which is temporally controlled by host mitochondria.); EspFu/tccP (Inducing degradation of the aniapoptic protein AbcF2, tight junction disruption, microvilli effacement and elongation, mitochondrial dysfunction, N-WASP activation, SGLT-1 inactivation, pedestal maturation, inhibition of NHE3 activity, membrane remodelling; targets and disrupts the nucleolus late in infection, which is temporally controlled by host mitochondria.); EspG (TBC-like GTPase activating protein. Efficiently catalyzes GTP hydrolysis in Rab1 to disrupt of

Rab1-mediated ER-to-Golgi trafficking.); EspH (First bacterial effector acting directly on RhoGEFs, EspH directly binds to the DH-PH domain in RhoGEFs to disrupt RhoGEF-Rho signaling; critical for inhibiting macrophage phagocytosis.); EspJ (Inhibit both IgG- and complement receptor-mediated phagocytosis.); EspK; EspL1; EspL2 (Cysteine protease. Bounds F-actin-aggregating annexin 2 directly to increase annexin 2's ability to aggregate Tir-induced F-actin; block necroptosis and in flammation.); EspL4; EspM1 (GEF. Activates the RhoA signaling pathway and induce the formation of stress fibres; inhibit pedestal formation and induce tight junction mislocalization.); EspM2 (GEF. Activates the RhoA signaling pathway and induce the formation of stress fibres; inhibit pedestal formation and induce tight junction mislocalization.); EspN; EspO1-1; EspO1-2; EspR1; EspR3; EspR4; EspT

(GEF. Activates Rac1 and Cdc42 leading to formation of membrane ruffles and lamellipodia; induces membrane ruffles to facilitate bacterial invasion into non-phagocytic cells in a process involving Rac1 and Wave2.); EspW; EspX1; EspX2; EspX4; EspX5; EspX6; EspX7/nleL (E3 ubiquitin ligase, HECT-like. Modulates pedestal formation.); EspY1; EspY2; EspY3; EspY4; EspY5; Map (GEF. Mimics the host Dbl and catalyses the exchange of GDP for GTP in Cdc42, involved in effacement, SGLT1 inhibition, formation of filopodia and disruption of mitochondrial function.); NleA/espI (Disruption of tight junctions by inhibition of host cell protein trafficking through COPII-dependent pathways.); NleB1 (Blocks translocation of the p65 and to the host cell nucleus to inhibit NF-<kappa>B pathway, but NleE and NleB act at different points in the NF-<kappa>B signaling pathway.); NleB2 (May also have anti-inflammatory

activity.); NleC (Metalloprotease. Zn-dependent endopeptidases that specifically clip and inactivate RelA (p65), thus blocking NF-<kappa>B pathway.); NleD (Metalloprotease. Zn-dependent endopeptidases that specifically clip and inactivate JNK and p38, thus blocking AP-1 pathway.); NleE (PMN tran-epithelial migration; blocks translocation of the p65 to the host cell nucleus by preventing I<kappa>B degradation to inhibit NF-<kappa>B pathway.); NleF; NleG-1; NleG2-2; NleG2-3; NleG2-4; NleG5-1; NleG5-2; NleG6-1; NleG6-2; NleG6-3; NleG7 (U-box type E3 ubiquitin ligases.); NleG8-2; NleH1 (Ser/Thr protein kinase. Binds directly to a subunit of NF-<kappa>B, the ribosomal protein S3 (RPS3), reducing the nuclear abundance of RPS3 to dampen host transcriptional outputs; interact with Bax inhibitor-1 to block apoptosis.); NleH2 (Putative kinase. Attenuates NF-<kappa>B pathway.); SepZ/espZ (EspZ interacts with CD98 in

ECs_1821	espO1-2	T3SS secreted effector EspO	VFG034803 (gb|NP_309594)	TTSS secreted effectors (VF1110)	host cell membranes to promote host cell survival, therefore provide the pathogen with valuable time to colonize efficiently prior to dissemination.); TccP2; Tir (Mimics host immunoreceptor tyrosine-based inhibition motifs (ITIMs), also see helicobacter CagA. EHEC Tir lacks the Nck binding site. Conserved NPY (Asn-Pro-Tyr) motif recruits the adaptor protein IRTKS and/or IRSp53. IRTKS/IRSp53 link Tir and TccP/EspFu, which in turn activates N-WASP; Receptor for intimin; effacement; SGLT1 inhibition; recruits SHIP2 to control actin-pedestal morphology; maintains the integrity of the epithelium by keeping the destructive activity of EspG and EspG2 in check.) Cif (Deamidase. Induces cytopathic effects of actin stress fiber formation and cell cycle arrest.); EspB (Pore formation, actin disruption, microvilli effacement, anti-phagocytosis.); EspF (Inducing degradation of the aniapoptic protein	setA	Escherichia coli O157:H7 str. Sakai	Effector delivery system	espO1-1	type III secreted effector	61.5	1.15E-40	129

AbcF2, tight junction disruption, microvilli effacement and elongation, mitochondrial dysfunction, N-WASP activation, SGLT-1 inactivation, pedestal maturation, inhibition of NHE3 activity, membrane remodelling; targets and disrupts the nucleolus late in infection, which is temporally controlled by host mitochondria.); EspFu/tccP (Inducing degradation of the aniapoptic protein AbcF2, tight junction disruption, microvilli effacement and elongation, mitochondrial dysfunction, N-WASP activation, SGLT-1 inactivation, pedestal maturation, inhibition of NHE3 activity, membrane remodelling; targets and disrupts the nucleolus late in infection, which is temporally controlled by host mitochondria.); EspG (TBC-like GTPase activating protein. Efficiently catalyzes GTP hydrolysis in Rab1 to disrupt of Rab1-mediated ER-to-Golgi trafficking.); EspH (First bacterial effector acting directly on RhoGEFs,

EspH directly binds to the DH-PH domain in RhoGEFs to disrupt RhoGEF-Rho signaling; critical for inhibiting macrophage phagocytosis.); EspJ (Inhibit both IgG- and complement receptor-mediated phagocytosis.); EspK; EspL1; EspL2 (Cysteine protease. Bounds F-actin-aggregating annexin 2 directly to increase annexin 2's ability to aggregate Tir-induced F-actin; block necroptosis and in flammation.); EspL4; EspM1 (GEF. Activates the RhoA signaling pathway and induce the formation of stress fibres; inhibit pedestal formation and induce tight junction mislocalization.); EspM2 (GEF. Activates the RhoA signaling pathway and induce the formation of stress fibres; inhibit pedestal formation and induce tight junction mislocalization.); EspN; EspO1-1; EspO1-2; EspR1; EspR3; EspR4; EspT (GEF. Activates Rac1 and Cdc42 leading to formation of membrane ruffles and lamellipodia; induces membrane

ruffles to facilitate bacterial invasion into non-phagocytic cells in a process involving Rac1 and Wave2.); EspW; EspX1; EspX2; EspX4; EspX5; EspX6; EspX7/nleL (E3 ubiquitin ligase, HECT-like. Modulates pedestal formation.); EspY1; EspY2; EspY3; EspY4; EspY5; Map (GEF. Mimics the host Dbl and catalyses the exchange of GDP for GTP in Cdc42, involved in effacement, SGLT1 inhibition, formation of filopodia and disruption of mitochondrial function.); NleA/espI (Disruption of tight junctions by inhibition of host cell protein trafficking through COPII-dependent pathways.); NleB1 (Blocks translocation of the p65 and to the host cell nucleus to inhibit NF-<kappa>B pathway, but NleE and NleB act at different points in the NF-<kappa>B signaling pathway.); NleB2 (May also have anti-inflammatory activity.); NleC (Metalloprotease. Zn-dependent endopeptidases that specifically clip and inactivate RelA

(p65), thus blocking NF-<kappa>B pathway.); NleD (Metalloprotease. Zn-dependent endopeptidases that specifically clip and inactivate JNK and p38, thus blocking AP-1 pathway.); NleE (PMN tran-epithelial migration; blocks translocation of the p65 to the host cell nucleus by preventing I<kappa>B degradation to inhibit NF-<kappa>B pathway.); NleF; NleG-1; NleG2-2; NleG2-3; NleG2-4; NleG5-1; NleG5-2; NleG6-1; NleG6-2; NleG6-3; NleG7 (U-box type E3 ubiquitin ligases.); NleG8-2; NleH1 (Ser/Thr protein kinase. Binds directly to a subunit of NF-<kappa>B, the ribosomal protein S3 (RPS3), reducing the nuclear abundance of RPS3 to dampen host transcriptional outputs; interact with Bax inhibitor-1 to block apoptosis.); NleH2 (Putative kinase. Attenuates NF-<kappa>B pathway.); SepZ/espZ (EspZ interacts with CD98 in host cell membranes to promote host cell survival, therefore provide the pathogen with valuable time to

ECs_1568	espK	T3SS secreted effector EspK	VFG034746 (gb|WP_000938118)	TTSS secreted effectors (VF1110)	colonize efficiently prior to dissemination.); TccP2; Tir (Mimics host immunoreceptor tyrosine-based inhibition motifs (ITIMs), also see helicobacter CagA. EHEC Tir lacks the Nck binding site. Conserved NPY (Asn-Pro-Tyr) motif recruits the adaptor protein IRTKS and/or IRSp53. IRTKS/IRSp53 link Tir and TccP/EspFu, which in turn activates N-WASP; Receptor for intimin; effacement; SGLT1 inhibition; recruits SHIP2 to control actin-pedestal morphology; maintains the integrity of the epithelium by keeping the destructive activity of EspG and EspG2 in check.) Cif (Deamidase. Induces cytopathic effects of actin stress fiber formation and cell cycle arrest.); EspB (Pore formation, actin disruption, microvilli effacement, anti-phagocytosis.); EspF (Inducing degradation of the aniapoptic protein AbcF2, tight junction disruption, microvilli effacement and elongation, mitochondrial dysfunction, N-WASP	setA	Escherichia coli O157:H7 str. EDL933	Effector delivery system	espK	Type III secretion system effector EspK	100	0	902

activation, SGLT-1 inactivation, pedestal maturation, inhibition of NHE3 activity, membrane remodelling; targets and disrupts the nucleolus late in infection, which is temporally controlled by host mitochondria.); EspFu/tccP (Inducing degradation of the aniapoptic protein AbcF2, tight junction disruption, microvilli effacement and elongation, mitochondrial dysfunction, N-WASP activation, SGLT-1 inactivation, pedestal maturation, inhibition of NHE3 activity, membrane remodelling; targets and disrupts the nucleolus late in infection, which is temporally controlled by host mitochondria.); EspG (TBC-like GTPase activating protein. Efficiently catalyzes GTP hydrolysis in Rab1 to disrupt of Rab1-mediated ER-to-Golgi trafficking.); EspH (First bacterial effector acting directly on RhoGEFs, EspH directly binds to the DH-PH domain in RhoGEFs to disrupt RhoGEF-Rho signaling; critical for inhibiting

macrophage phagocytosis.); EspJ (Inhibit both IgG- and complement receptor-mediated phagocytosis.); EspK; EspL1; EspL2 (Cysteine protease. Bounds F-actin-aggregating annexin 2 directly to increase annexin 2's ability to aggregate Tir-induced F-actin; block necroptosis and in flammation.); EspL4; EspM1 (GEF. Activates the RhoA signaling pathway and induce the formation of stress fibres; inhibit pedestal formation and induce tight junction mislocalization.); EspM2 (GEF. Activates the RhoA signaling pathway and induce the formation of stress fibres; inhibit pedestal formation and induce tight junction mislocalization.); EspN; EspO1-1; EspO1-2; EspR1; EspR3; EspR4; EspT (GEF. Activates Rac1 and Cdc42 leading to formation of membrane ruffles and lamellipodia; induces membrane ruffles to facilitate bacterial invasion into non-phagocytic cells in a process involving Rac1 and Wave2.);

EspW; EspX1; EspX2; EspX4; EspX5; EspX6; EspX7/nleL (E3 ubiquitin ligase, HECT-like. Modulates pedestal formation.); EspY1; EspY2; EspY3; EspY4; EspY5; Map (GEF. Mimics the host Dbl and catalyses the exchange of GDP for GTP in Cdc42, involved in effacement, SGLT1 inhibition, formation of filopodia and disruption of mitochondrial function.); NleA/espI (Disruption of tight junctions by inhibition of host cell protein trafficking through COPII-dependent pathways.); NleB1 (Blocks translocation of the p65 and to the host cell nucleus to inhibit NF-<kappa>B pathway, but NleE and NleB act at different points in the NF-<kappa>B signaling pathway.); NleB2 (May also have anti-inflammatory activity.); NleC (Metalloprotease. Zn-dependent endopeptidases that specifically clip and inactivate RelA (p65), thus blocking NF-<kappa>B pathway.); NleD (Metalloprotease. Zn-dependent endopeptidases that

specifically clip and inactivate JNK and p38, thus blocking AP-1 pathway.); NleE (PMN tran-epithelial migration; blocks translocation of the p65 to the host cell nucleus by preventing I<kappa>B degradation to inhibit NF-<kappa>B pathway.); NleF; NleG-1; NleG2-2; NleG2-3; NleG2-4; NleG5-1; NleG5-2; NleG6-1; NleG6-2; NleG6-3; NleG7 (U-box type E3 ubiquitin ligases.); NleG8-2; NleH1 (Ser/Thr protein kinase. Binds directly to a subunit of NF-<kappa>B, the ribosomal protein S3 (RPS3), reducing the nuclear abundance of RPS3 to dampen host transcriptional outputs; interact with Bax inhibitor-1 to block apoptosis.); NleH2 (Putative kinase. Attenuates NF-<kappa>B pathway.); SepZ/espZ (EspZ interacts with CD98 in host cell membranes to promote host cell survival, therefore provide the pathogen with valuable time to colonize efficiently prior to dissemination.); TccP2; Tir (Mimics host immunoreceptor tyrosine-based

					inhibition motifs (ITIMs), also see helicobacter CagA. EHEC Tir lacks the Nck binding site. Conserved NPY (Asn-Pro-Tyr) motif recruits the adaptor protein IRTKS and/or IRSp53. IRTKS/IRSp53 link Tir and TccP/EspFu, which in turn activates N-WASP; Receptor for intimin; effacement; SGLT1 inhibition; recruits SHIP2 to control actin-pedestal morphology; maintains the integrity of the epithelium by keeping the destructive activity of EspG and EspG2 in check.)							
ECs_0964	macA	macrolide transporter membrane fusion protein component	VFG037711 (gb|WP_000010646)	AdeFGH efflux pump (VF0504)	Play a potential role in the synthesis and transport of autoinducer molecules during biofilm formation	setA	Acinetobacter baumannii D1279779	Biofilm	adeF	membrane-fusion protein	29.1	1.73E-25	106
ECs_2882	mdtA	multidrug efflux system subunit A	VFG037711 (gb|WP_000010646)	AdeFGH efflux pump (VF0504)	Play a potential role in the synthesis and transport of autoinducer molecules during biofilm formation	setA	Acinetobacter baumannii D1279779	Biofilm	adeF	membrane-fusion protein	32.4	9.82E-50	173
ECs_4473	yibH	membrane fusion	VFG037711 (gb|WP_00	AdeFGH efflux	Play a potential role in the synthesis and transport of autoinducer molecules	setA	Acinetobacter	Biofilm	adeF	membrane-fusion	23.8	2.06E-06	49.3

		protein component of efflux pump	0010646)	pump (VF0504)	during biofilm formation		baumannii D1279779			protein			
ECs_0217	ECs_0217	hypothetical protein	VFG035459 (gb|WP_001087743)	EHS (VF1176)	-	setA	Escherichia coli O157:H7 str. EDL933	Effector delivery system	aec31	type VI secretion system ImpA and VasL domain-containing protein	100	0	919
ECs_0216	ECs_0216	type VI secretion system effector Hcp1 family protein	VFG035434 (gb|WP_001284958)	ACE T6SS (VF1122)	-	setA	Escherichia coli O157:H7 str. EDL933	Effector delivery system	aec32	Hcp family type VI secretion system effector	100	1.37E-116	326
ECs_0218	impL	type VI secretion system protein ImpL	VFG035484 (gb|WP_001303798)	EHS (VF1176)	-	setA	Escherichia coli O157:H7 str. EDL933	Effector delivery system	aec30	type VI secretion system membrane subunit TssM	99.9	0	2291
ECs_0968	clpA	ATP-binding	VFG000079 (gb|NP_46	ClpC (VF0072)	An ATPase promoting early escape form the phagosome of macrophages; ClpC is	setA	Listeria monocytoge	Stress survival	clpC	endopeptidase Clp	37.5	5.74E-169	507

		component of serine protease	3763)		also required for adhesion and invasion, possibly by modulating the expression of InlA,InlB and ActA		nes EGD-e			ATP-binding chain C			
ECs_0492	clpX	ATP-dependent protease ATP-binding subunit ClpX	VFG000079 (gb|NP_463763)	ClpC (VF0072)	An ATPase promoting early escape form the phagosome of macrophages; ClpC is also required for adhesion and invasion, possibly by modulating the expression of InlA,InlB and ActA	setA	Listeria monocytogenes EGD-e	Stress survival	clpC	endopeptidase Clp ATP-binding chain C	25.3	1.65E-07	53.5
ECs_3455	clpB	chaperone protein ClpB	VFG049191 (gb|WP_014907101)	T6SS-II (VF0783)	-	setA	Klebsiella pneumoniae subsp. pneumoniae 1084	Effector delivery system	clpV	ATP-dependent chaperone ClpB	94.9	0	1550
ECs_1588	pchE	transcriptional regulator PchE	VFG000765 (gb|BAA84861)	Per (VF0190)	Up-regulates the expression of BFP and intimin and regulates Esps secretion in response to different environmental cues; activates the expression of ler, which then activates the expression of LEE2, LEE3, tir and LEE4 in a cascade fashion	setA	Escherichia coli B171	Regulation	bfpW	BfpW	39.3	9.55E-18	70.9
ECs_1091	pchA	transcriptional regulator PchA	VFG000765 (gb|BAA84861)	Per (VF0190)	Up-regulates the expression of BFP and intimin and regulates Esps secretion in response to different environmental cues; activates the expression of ler,	setA	Escherichia coli B171	Regulation	bfpW	BfpW	47.1	1.20E-19	76.3

					which then activates the expression of LEE2, LEE3, tir and LEE4 in a cascade fashion								
ECs_2182	pchB	PchABC family transcriptional regulator	VFG000765 (gb|BAA84861)	Per (VF0190)	Up-regulates the expression of BFP and intimin and regulates Esps secretion in response to different environmental cues; activates the expression of ler, which then activates the expression of LEE2, LEE3, tir and LEE4 in a cascade fashion	setA	Escherichia coli B171	Regulation	bfpW	BfpW	47.1	8.43E-20	76.6
ECs_2737	pchC	PchABC family transcriptional regulator	VFG000765 (gb|BAA84861)	Per (VF0190)	Up-regulates the expression of BFP and intimin and regulates Esps secretion in response to different environmental cues; activates the expression of ler, which then activates the expression of LEE2, LEE3, tir and LEE4 in a cascade fashion	setA	Escherichia coli B171	Regulation	bfpW	BfpW	47.1	1.69E-19	75.9
ECs_3646	barA	hybrid sensory histidine kinase BarA	VFG015075 (gb|WP_011532652)	GacS/GacA two-component system (VF0908)	-	setA	Pseudomonas entomophila L48	Regulation	gacS	response regulator	37	1.56E-177	538
ECs_3107	rcsC	hybrid sensory kinase in two-compo	VFG015075 (gb|WP_011532652)	GacS/GacA two-component system	-	setA	Pseudomonas entomophila L48	Regulation	gacS	response regulator	35.2	2.66E-64	234

		nent regulatory system with RcsB and YojN		(VF0908)								
ECs_0696	ybeX	ion transport	VFG040674 (gb|WP_011271490)	TlyC (VF0489)	Plays a role in phagosomal escape by rickettsiae	setA	Rickettsia felis URRWXCal2	Exoenzyme	tlyC	hemolysin C	32.8	4.27E-37	133
ECs_0694	gltI	glutamate/aspartate periplasmic binding protein	VFG011637 (gb|WP_002867450)	PEB1 (VF0327)	Cell-binding factor, enhancing bacterial adherence to and invasion of host cells	setA	Campylobacter jejuni RM1221	Adherence	pebA	bifunctional adhesin/ABC transporter aspartate/glutamate-binding protein	26.8	1.61E-19	85.9
ECs_3193	hisJ	histidine ABC transporter periplasmic binding protein	VFG011637 (gb|WP_002867450)	PEB1 (VF0327)	Cell-binding factor, enhancing bacterial adherence to and invasion of host cells	setB	Campylobacter jejuni RM1221	Adherence	pebA	bifunctional adhesin/ABC transporter aspartate/glutamate-	27.6	6.69E-09	55.1

										binding protein			
ECs_0943	artJ	arginine ABC transporter periplasmic binding protein	VFG011637 (gb|WP_002867450)	PEB1 (VF0327)	Cell-binding factor, enhancing bacterial adherence to and invasion of host cells	setA	Campylobacter jejuni RM1221	Adherence	pebA	bifunctional adhesin/ABC transporter aspartate/glutamate-binding protein	26.7	2.46E-12	64.7
ECs_4141	yhdW	periplasmic binding transport protein	VFG011637 (gb|WP_002867450)	PEB1 (VF0327)	Cell-binding factor, enhancing bacterial adherence to and invasion of host cells	setA	Campylobacter jejuni RM1221	Adherence	pebA	bifunctional adhesin/ABC transporter aspartate/glutamate-binding protein	25.3	1.01E-13	70.1
ECs_2659	fliY	cystine transporter subunit	VFG011638 (gb|WP_002865903)	PEB1 (VF0327)	Cell-binding factor, enhancing bacterial adherence to and invasion of host cells	setA	Campylobacter jejuni subsp. jejuni	Adherence	pebA	bifunctional adhesin/ABC	31.2	1.80E-23	96.3

							81-176			transporter aspartate/glutamate-binding protein			
ECs_3194	argT	lysine/arginine/ornithine transporter subunit	VFG043099 (gb|WP_001302033)	Peritrichous flagella (VF1154)	-	setB	Escherichia coli O157:H7 str. EDL933	Motility	tcyJ	cystine ABC transporter substrate-binding protein	31	5.93E-33	120
ECs_0889	glnH	periplasmic glutamine-binding protein	VFG043099 (gb|WP_001302033)	Peritrichous flagella (VF1154)	-	setA	Escherichia coli O157:H7 str. EDL933	Motility	tcyJ	cystine ABC transporter substrate-binding protein	33.3	9.45E-40	138
ECs_0946	artI	arginine transporter subunit	VFG043099 (gb|WP_001302033)	Peritrichous flagella (VF1154)	-	setA	Escherichia coli O157:H7 str. EDL933	Motility	tcyJ	cystine ABC transporter substrate-binding protein	30.6	1.34E-33	122
ECs_0693	gltJ	glutamate/aspartat	VFG002901 (gb|WP_00	O-antigen (VF0392)	LPS O antigen mutants were severely impaired in their ability to colonize	setB	Yersinia pestis	Immune modulation	YPA_RS10790	histidine ABC	26.6	4.23E-11	60.8

		e ABC transporter permease	2209736)		the Peyer's patches and did not colonize spleen and liver. The absence of O antigen in the outer membrane affects the expression of other Yersinia virulence factors.		Antiqua			transporter permease HisQ			
ECs_0692	gltK	glutamate/aspartate ABC transporter permease	VFG002901 (gb|WP_002209736)	O-antigen (VF0392)	LPS O antigen mutants were severely impaired in their ability to colonize the Peyer's patches and did not colonize spleen and liver. The absence of O antigen in the outer membrane affects the expression of other Yersinia virulence factors.	setB	Yersinia pestis Antiqua	Immune modulation	YPA_RS10790	histidine ABC transporter permease HisQ	26.9	1.94E-14	69.7
ECs_3191	hisM	histidine ABC transporter permease	VFG002901 (gb|WP_002209736)	O-antigen (VF0392)	LPS O antigen mutants were severely impaired in their ability to colonize the Peyer's patches and did not colonize spleen and liver. The absence of O antigen in the outer membrane affects the expression of other Yersinia virulence factors.	setB	Yersinia pestis Antiqua	Immune modulation	YPA_RS10790	histidine ABC transporter permease HisQ	32.9	5.96E-26	100
ECs_2656	yecS	ABC family inner membrane permease	VFG002901 (gb|WP_002209736)	O-antigen (VF0392)	LPS O antigen mutants were severely impaired in their ability to colonize the Peyer's patches and did not colonize spleen and liver. The absence of O antigen in the outer membrane affects the expression of other Yersinia virulence factors.	setB	Yersinia pestis Antiqua	Immune modulation	YPA_RS10790	histidine ABC transporter permease HisQ	30	1.39E-19	83.6

ECs_4143	yhdY	amino acid ABC transporter permease	VFG002901 (gb|WP_002209736)	O-antigen (VF0392)	LPS O antigen mutants were severely impaired in their ability to colonize the Peyer's patches and did not colonize spleen and liver. The absence of O antigen in the outer membrane affects the expression of other Yersinia virulence factors.	setB	Yersinia pestis Antiqua	Immune modulation	YPA_RS10790	histidine ABC transporter permease HisQ	29.8	5.45E-06	47
ECs_0888	glnP	glutamine ABC transporter permease protein	VFG002901 (gb|WP_002209736)	O-antigen (VF0392)	LPS O antigen mutants were severely impaired in their ability to colonize the Peyer's patches and did not colonize spleen and liver. The absence of O antigen in the outer membrane affects the expression of other Yersinia virulence factors.	setB	Yersinia pestis Antiqua	Immune modulation	YPA_RS10790	histidine ABC transporter permease HisQ	28	2.29E-22	90.9
ECs_0945	artQ	arginine ABC transporter permease	VFG002901 (gb|WP_002209736)	O-antigen (VF0392)	LPS O antigen mutants were severely impaired in their ability to colonize the Peyer's patches and did not colonize spleen and liver. The absence of O antigen in the outer membrane affects the expression of other Yersinia virulence factors.	setB	Yersinia pestis Antiqua	Immune modulation	YPA_RS10790	histidine ABC transporter permease HisQ	37	1.95E-36	128
ECs_3192	hisQ	histidine ABC transporter permease	VFG002901 (gb|WP_002209736)	O-antigen (VF0392)	LPS O antigen mutants were severely impaired in their ability to colonize the Peyer's patches and did not colonize spleen and liver. The absence of O antigen in the outer membrane	setB	Yersinia pestis Antiqua	Immune modulation	YPA_RS10790	histidine ABC transporter permease HisQ	77.6	1.20E-121	344

					affects the expression of other Yersinia virulence factors.							
ECs_0897	ybiT	ABC-F family regulatory ATPase	VFG036559 (gb|WP_013449339)	FbpABC (VF0272)	Encodes a periplasmic-binding protein-dependent iron transport system necessary for the utilization of iron bound to transferrin or iron chelates, FbpA is the periplasmic Fe3+ binding protein	setA	Neisseria lactamica 020-06	Nutritional/Metabolic factor	fbpC	iron(III) ABC transporter, ATP-binding protein	28.2	1.60E-18	87
ECs_3190	hisP	histidine ABC transporter ATP-binding protein	VFG036559 (gb|WP_013449339)	FbpABC (VF0272)	Encodes a periplasmic-binding protein-dependent iron transport system necessary for the utilization of iron bound to transferrin or iron chelates, FbpA is the periplasmic Fe3+ binding protein	setA	Neisseria lactamica 020-06	Nutritional/Metabolic factor	fbpC	iron(III) ABC transporter, ATP-binding protein	36.4	1.04E-35	130
ECs_3293	cysA	sulfate/thiosulfate transporter subunit	VFG036559 (gb|WP_013449339)	FbpABC (VF0272)	Encodes a periplasmic-binding protein-dependent iron transport system necessary for the utilization of iron bound to transferrin or iron chelates, FbpA is the periplasmic Fe3+ binding protein	setA	Neisseria lactamica 020-06	Nutritional/Metabolic factor	fbpC	iron(III) ABC transporter, ATP-binding protein	37.5	5.03E-61	199
ECs_3016	yehX	transporter subunit: ATP-binding	VFG036559 (gb|WP_013449339)	FbpABC (VF0272)	Encodes a periplasmic-binding protein-dependent iron transport system necessary for the utilization of iron bound to transferrin or iron chelates, FbpA is the periplasmic Fe3+	setA	Neisseria lactamica 020-06	Nutritional/Metabolic factor	fbpC	iron(III) ABC transporter, ATP-bindin	39.4	3.44E-50	169

		component of ABC superfamily protein			binding protein					g protein			
ECs_2608	araG	L-arabinose ABC transporter ATPase	VFG036559 (gb|WP_013449339)	FbpABC (VF0272)	Encodes a periplasmic-binding protein-dependent iron transport system necessary for the utilization of iron bound to transferrin or iron chelates, FbpA is the periplasmic Fe3+ binding protein	setA	Neisseria lactamica 020-06	Nutritional/Metabolic factor	fbpC	iron(III) ABC transporter, ATP-binding protein	32.7	2.99E-20	92
ECs_0070	thiQ	thiamine/thiamine pyrophosphate ABC transporter ATPase	VFG036559 (gb|WP_013449339)	FbpABC (VF0272)	Encodes a periplasmic-binding protein-dependent iron transport system necessary for the utilization of iron bound to transferrin or iron chelates, FbpA is the periplasmic Fe3+ binding protein	setA	Neisseria lactamica 020-06	Nutritional/Metabolic factor	fbpC	iron(III) ABC transporter, ATP-binding protein	38.4	8.77E-38	134
ECs_3802	ECs_3802	ABC transporter ATP-binding protein	VFG036559 (gb|WP_013449339)	FbpABC (VF0272)	Encodes a periplasmic-binding protein-dependent iron transport system necessary for the utilization of iron bound to transferrin or iron chelates, FbpA is the periplasmic Fe3+ binding protein	setA	Neisseria lactamica 020-06	Nutritional/Metabolic factor	fbpC	iron(III) ABC transporter, ATP-binding protein	31.8	4.07E-19	84.3
ECs_1495	lolD	lipoprotein-releasing	VFG036559 (gb|WP_013449339)	FbpABC (VF0272)	Encodes a periplasmic-binding protein-dependent iron transport system necessary for the utilization	setA	Neisseria lactamica 020-06	Nutritional/Metabolic factor	fbpC	iron(III) ABC transporte	36.7	1.10E-33	124

		system ATP-binding protein			of iron bound to transferrin or iron chelates, FbpA is the periplasmic Fe3+ binding protein					r, ATP-binding protein			
ECs_0788	modF	molybdate ABC transporter ATPase	VFG036559 (gb|WP_013449339)	FbpABC (VF0272)	Encodes a periplasmic-binding protein-dependent iron transport system necessary for the utilization of iron bound to transferrin or iron chelates, FbpA is the periplasmic Fe3+ binding protein	setA	Neisseria lactamica 020-06	Nutritional/Metabolic factor	fbpC	iron(III) ABC transporter, ATP-binding protein	33.2	1.81E-18	86.7
ECs_4074	mlaF	organic solvent ABC transporter ATP-binding protein	VFG006259 (gb|WP_003687540)	FbpABC (VF0272)	Encodes a periplasmic-binding protein-dependent iron transport system necessary for the utilization of iron bound to transferrin or iron chelates, FbpA is the periplasmic Fe3+ binding protein	setA	Neisseria gonorrhoeae FA 1090	Nutritional/Metabolic factor	fbpC	iron(III) ABC transporter, ATP-binding protein	37.9	1.19E-29	114
ECs_3041	mglA	methyl-galactoside ABC transporter ATPase	VFG006259 (gb|WP_003687540)	FbpABC (VF0272)	Encodes a periplasmic-binding protein-dependent iron transport system necessary for the utilization of iron bound to transferrin or iron chelates, FbpA is the periplasmic Fe3+ binding protein	setA	Neisseria gonorrhoeae FA 1090	Nutritional/Metabolic factor	fbpC	iron(III) ABC transporter, ATP-binding protein	30.1	2.76E-17	83.2
ECs_5079	phnL	ribophosphonate	VFG006259 (gb|WP_00	FbpABC (VF0272)	Encodes a periplasmic-binding protein-dependent iron transport	setA	Neisseria gonorrhoea	Nutritional/Metaboli	fbpC	iron(III) ABC	30.2	5.23E-18	81.3

		triphosphate synthase subunit	3687540)		system necessary for the utilization of iron bound to transferrin or iron chelates, FbpA is the periplasmic Fe3+ binding protein		e FA 1090	c factor		transporter, ATP-binding protein			
ECs_5349	ettA	energy-dependent translational throttle A	VFG006259 (gb|WP_003687540)	FbpABC (VF0272)	Encodes a periplasmic-binding protein-dependent iron transport system necessary for the utilization of iron bound to transferrin or iron chelates, FbpA is the periplasmic Fe3+ binding protein	setA	Neisseria gonorrhoeae FA 1090	Nutritional/Metabolic factor	fbpC	iron(III) ABC transporter, ATP-binding protein	33	3.31E-17	83.2
ECs_2087	ddpF	D%2CD-dipeptide ABC transporter ATPase	VFG006259 (gb|WP_003687540)	FbpABC (VF0272)	Encodes a periplasmic-binding protein-dependent iron transport system necessary for the utilization of iron bound to transferrin or iron chelates, FbpA is the periplasmic Fe3+ binding protein	setA	Neisseria gonorrhoeae FA 1090	Nutritional/Metabolic factor	fbpC	iron(III) ABC transporter, ATP-binding protein	35	8.11E-30	115
ECs_3413	yphE	sugar ABC transporter ATPase	VFG006259 (gb|WP_003687540)	FbpABC (VF0272)	Encodes a periplasmic-binding protein-dependent iron transport system necessary for the utilization of iron bound to transferrin or iron chelates, FbpA is the periplasmic Fe3+ binding protein	setA	Neisseria gonorrhoeae FA 1090	Nutritional/Metabolic factor	fbpC	iron(III) ABC transporter, ATP-binding protein	30.9	4.86E-21	94.4
ECs_0367	yahD	ankyrin domain-containing protein	VFG041401 (gb|WP_011452759)	T4SS (VF1269)	-	setB	Ehrlichia chaffeensis str. Arkansas	Effector delivery system	ECH_RS02870	ankyrin repeat domain-containing	26.2	3.14E-06	47

											protein			
ECs_0363	yahA	c-di-GMP-specific phosphodiesterase	VFG045467 (gb|WP_011204972)	CdpA (VF0432)	A major c-di-GMP-specific phosphodiesterase in regulating intracellular levels of c-di-GMP, affecting diverse phenotypes such as flagellum synthesis, bacterial motility, the production of exopolysaccharides, cell-to-cell aggregation, biofilm formation, cytotoxicity, and invasion of human cells.	setA	Burkholderia pseudomallei K96243	Regulation	cdpA		cyclic di-GMP phosphodiesterase	34.2	5.11E-37	140
ECs_2129	yneF	diguanylate cyclase	VFG045467 (gb|WP_011204972)	CdpA (VF0432)	A major c-di-GMP-specific phosphodiesterase in regulating intracellular levels of c-di-GMP, affecting diverse phenotypes such as flagellum synthesis, bacterial motility, the production of exopolysaccharides, cell-to-cell aggregation, biofilm formation, cytotoxicity, and invasion of human cells.	setA	Burkholderia pseudomallei K96243	Regulation	cdpA		cyclic di-GMP phosphodiesterase	22.8	1.75E-07	53.5
ECs_2694	yedQ	membrane-anchored diguanylate cyclase	VFG045467 (gb|WP_011204972)	CdpA (VF0432)	A major c-di-GMP-specific phosphodiesterase in regulating intracellular levels of c-di-GMP, affecting diverse phenotypes such as flagellum synthesis, bacterial	setA	Burkholderia pseudomallei K96243	Regulation	cdpA		cyclic di-GMP phosphodiesterase	25	2.34E-07	53.5

					motility, the production of exopolysaccharides, cell-to-cell aggregation, biofilm formation, cytotoxicity, and invasion of human cells.								
ECs_5043	yjcC	membrane-anchored cyclic-di-GMP phosphodiesterase	VFG045467 (gb|WP_011204972)	CdpA (VF0432)	A major c-di-GMP-specific phosphodiesterase in regulating intracellular levels of c-di-GMP, affecting diverse phenotypes such as flagellum synthesis, bacterial motility, the production of exopolysaccharides, cell-to-cell aggregation, biofilm formation, cytotoxicity, and invasion of human cells.	setA	Burkholderia pseudomallei K96243	Regulation	cdpA	cyclic di-GMP phosphodiesterase	32.7	1.36E-33	134
ECs_0510	ylaB	membrane-anchored cyclic-di-GMP phosphodiesterase	VFG045467 (gb|WP_011204972)	CdpA (VF0432)	A major c-di-GMP-specific phosphodiesterase in regulating intracellular levels of c-di-GMP, affecting diverse phenotypes such as flagellum synthesis, bacterial motility, the production of exopolysaccharides, cell-to-cell aggregation, biofilm formation, cytotoxicity, and invasion of human cells.	setA	Burkholderia pseudomallei K96243	Regulation	cdpA	cyclic di-GMP phosphodiesterase	34.3	1.33E-38	148
ECs_0435	yaiC	diguanyla	VFG045467	CdpA	A major c-di-GMP-specific	setA	Burkholder	Regulation	cdpA	cyclic	29.9	8.26E-	53.9

		te cyclase	(gb|WP_01 1204972)	(VF0432)	phosphodiesterase in regulating intracellular levels of c-di-GMP, affecting diverse phenotypes such as flagellum synthesis, bacterial motility, the production of exopolysaccharides, cell-to-cell aggregation, biofilm formation, cytotoxicity, and invasion of human cells.		ia pseudomall ei K96243			di-GMP phosphodie sterase		08	
ECs_2494	yeaI	membrane-anchored diguanyla te cyclase	VFG045467 (gb|WP_01 1204972)	CdpA (VF0432)	A major c-di-GMP-specific phosphodiesterase in regulating intracellular levels of c-di-GMP, affecting diverse phenotypes such as flagellum synthesis, bacterial motility, the production of exopolysaccharides, cell-to-cell aggregation, biofilm formation, cytotoxicity, and invasion of human cells.	setA	Burkholder ia pseudomall ei K96243	Regulation	cdpA	cyclic di-GMP phosphodie sterase	23.4	4.50E-06	48.9
ECs_3068	rtn	phage resistanc e protein	VFG045467 (gb|WP_01 1204972)	CdpA (VF0432)	A major c-di-GMP-specific phosphodiesterase in regulating intracellular levels of c-di-GMP, affecting diverse phenotypes such as flagellum synthesis, bacterial motility, the production of exopolysaccharides, cell-to-cell	setA	Burkholder ia pseudomall ei K96243	Regulation	cdpA	cyclic di-GMP phosphodie sterase	30.9	8.85E-34	134

					aggregation, biofilm formation, cytotoxicity, and invasion of human cells.								
ECs_3273	yfeA	diguanylate cyclase	VFG045467 (gb|WP_011204972)	CdpA (VF0432)	A major c-di-GMP-specific phosphodiesterase in regulating intracellular levels of c-di-GMP, affecting diverse phenotypes such as flagellum synthesis, bacterial motility, the production of exopolysaccharides, cell-to-cell aggregation, biofilm formation, cytotoxicity, and invasion of human cells.	setA	Burkholderia pseudomallei K96243	Regulation	cdpA	cyclic di-GMP phosphodiesterase	29	1.87E-21	99
ECs_1925	ydaM	diguanylate cyclase	VFG045467 (gb|WP_011204972)	CdpA (VF0432)	A major c-di-GMP-specific phosphodiesterase in regulating intracellular levels of c-di-GMP, affecting diverse phenotypes such as flagellum synthesis, bacterial motility, the production of exopolysaccharides, cell-to-cell aggregation, biofilm formation, cytotoxicity, and invasion of human cells.	setA	Burkholderia pseudomallei K96243	Regulation	cdpA	cyclic di-GMP phosphodiesterase	27.8	1.18E-10	63.2
ECs_2524	yoaD	membrane-anchored cyclic-di	VFG045467 (gb|WP_011204972)	CdpA (VF0432)	A major c-di-GMP-specific phosphodiesterase in regulating intracellular levels of c-di-GMP,	setA	Burkholderia pseudomall	Regulation	cdpA	cyclic di-GMP phosphodie	31	7.94E-31	126

		-GMP phosphodiesterase			affecting diverse phenotypes such as flagellum synthesis, bacterial motility, the production of exopolysaccharides, cell-to-cell aggregation, biofilm formation, cytotoxicity, and invasion of human cells.		ei K96243			sterase			
ECs_2095	dosC	diguanylate cyclase	VFG045467 (gb|WP_011204972)	CdpA (VF0432)	A major c-di-GMP-specific phosphodiesterase in regulating intracellular levels of c-di-GMP, affecting diverse phenotypes such as flagellum synthesis, bacterial motility, the production of exopolysaccharides, cell-to-cell aggregation, biofilm formation, cytotoxicity, and invasion of human cells.	setA	Burkholderia pseudomallei K96243	Regulation	cdpA	cyclic di-GMP phosphodiesterase	26.2	1.35E-08	57
ECs_0912	yliE	membrane-anchored cyclic-di-GMP phosphodiesterase	VFG045467 (gb|WP_011204972)	CdpA (VF0432)	A major c-di-GMP-specific phosphodiesterase in regulating intracellular levels of c-di-GMP, affecting diverse phenotypes such as flagellum synthesis, bacterial motility, the production of exopolysaccharides, cell-to-cell aggregation, biofilm formation, cytotoxicity, and invasion of human	setA	Burkholderia pseudomallei K96243	Regulation	cdpA	cyclic di-GMP phosphodiesterase	29.7	6.29E-31	128

					cells.								
ECs_1272	ECs_1272	diguanylate phosphodiesterase	VFG045467 (gb|WP_011204972)	CdpA (VF0432)	A major c-di-GMP-specific phosphodiesterase in regulating intracellular levels of c-di-GMP, affecting diverse phenotypes such as flagellum synthesis, bacterial motility, the production of exopolysaccharides, cell-to-cell aggregation, biofilm formation, cytotoxicity, and invasion of human cells.	setA	Burkholderia pseudomallei K96243	Regulation	cdpA	cyclic di-GMP phosphodiesterase	33.5	3.38E-36	142
ECs_1858	gmr	modulator of Rnase II stability	VFG045467 (gb|WP_011204972)	CdpA (VF0432)	A major c-di-GMP-specific phosphodiesterase in regulating intracellular levels of c-di-GMP, affecting diverse phenotypes such as flagellum synthesis, bacterial motility, the production of exopolysaccharides, cell-to-cell aggregation, biofilm formation, cytotoxicity, and invasion of human cells.	setA	Burkholderia pseudomallei K96243	Regulation	cdpA	cyclic di-GMP phosphodiesterase	32.5	8.99E-66	226
ECs_4409	yhjK	cyclic-di-GMP phosphodiesterase	VFG045467 (gb|WP_011204972)	CdpA (VF0432)	A major c-di-GMP-specific phosphodiesterase in regulating intracellular levels of c-di-GMP, affecting diverse phenotypes such as flagellum synthesis, bacterial	setA	Burkholderia pseudomallei K96243	Regulation	cdpA	cyclic di-GMP phosphodiesterase	30.9	5.00E-44	166

					motility, the production of exopolysaccharides, cell-to-cell aggregation, biofilm formation, cytotoxicity, and invasion of human cells.								
ECs_2495	yeaJ	diguanylate cyclase	VFG049933 (gb|WP_011199086)	PlcR-PapR quorum sensing (VF0660)	-	setB	Bacillus cereus E33L	Biofilm	papR	DUF4084 domain-containing protein	38.6	3.44E-12	68.9
ECs_3467	yfiN	membrane-anchored diguanylate cyclase	VFG049933 (gb|WP_011199086)	PlcR-PapR quorum sensing (VF0660)	-	setB	Bacillus cereus E33L	Biofilm	papR	DUF4084 domain-containing protein	40.4	2.12E-21	96.7
ECs_0913	yliF	membrane-anchored diguanylate cyclase	VFG049933 (gb|WP_011199086)	PlcR-PapR quorum sensing (VF0660)	-	setB	Bacillus cereus E33L	Biofilm	papR	DUF4084 domain-containing protein	33	3.94E-15	77.8
ECs_1271	ycdT	diguanylate cyclase	VFG049933 (gb|WP_011199086)	PlcR-PapR quorum sensing (VF0660)	-	setB	Bacillus cereus E33L	Biofilm	papR	DUF4084 domain-containing protein	31.8	8.35E-19	89.4
ECs_2144	dgcZ	diguanylate cyclase	VFG049933 (gb|WP_011199086)	PlcR-PapR quorum sensing	-	setB	Bacillus cereus E33L	Biofilm	papR	DUF4084 domain-containing	31.3	4.58E-18	84.3

				(VF0660)						protein			
ECs_0362	ECs_0362	AidA-I family adhesin	VFG035974 (gb|WP_001491890)	EhaA, AIDA-I type (VF1127)	-	setA	Escherichia coli O157:H7 str. EDL933	Adherence	ehaA	autotransporter adhesin EhaA	100	0	2400
ECs_2117	ydeK	lipoprotein	VFG035974 (gb|WP_001491890)	EhaA, AIDA-I type (VF1127)	-	setA	Escherichia coli O157:H7 str. EDL933	Adherence	ehaA	autotransporter adhesin EhaA	35	2.08E-51	199
ECs_3116	yfaL	adhesin	VFG035982 (gb|WP_012817848)	EhaA, AIDA-I type (VF1127)	-	setA	Escherichia coli O111:H- str. 11128	Adherence	ehaA	autotransporter adhesin EhaA	32.4	1.39E-52	202
ECs_0207	mltD	membrane-bound lytic murein transglycosylase D	VFG041913 (gb|WP_005763910)	P. syringae TTSS (VF0909)	-	setB	Pseudomonas syringae pv. tomato str. DC3000	Effector delivery system	PSPTO_RS07240	transglycosylase SLT domain-containing protein	41	2.49E-64	215
ECs_5350	slt	lytic murein transglycosylase	VFG041913 (gb|WP_005763910)	P. syringae TTSS (VF0909)	-	setB	Pseudomonas syringae pv. tomato str. DC3000	Effector delivery system	PSPTO_RS07240	transglycosylase SLT domain-containing protein	30.8	4.15E-06	49.7
ECs_3738	ygeR	hypothetical	VFG043453 (gb|WP_00	SE2319 (VF1000)	-	setB	Staphylococcus	Adherence	aae	autolysin/adhesin Aae	49.3	2.02E-08	53.9

		protein	1829380)				epidermidis ATCC 12228						
ECs_0183	lpxA	UDP-N-acetylglucosamine acetyltransferase	VFG013393 (gb|WP_010945086)	LOS (VF0044)	Major immunogen; LOS phosphorylcholine (ChoP) may influence invasion via interaction with PAF receptor and stimulates of inflammatory signals; LPS phase variation is characterized by the spontaneous loss and gain of oligosaccharide structures present in the outer core. the phase variable expression of LPS biosynthesis genes promotes evasion of antigen-specific host immune defences and allow colonization of different host microenvironments	setA	Haemophilus ducreyi 35000HP	Immune modulation	lpxA	UDP-N-acetylglucosamine acyltransferase	63.4	1.66E-103	301
ECs_0182	fabZ	(3R)-hydroxymyristoyl-[acyl carrier protein] dehydratase	VFG011402 (gb|WP_004688426)	LPS (VF0367)	Plays a role in entry and early survival inside macrophages; Resistance to innate-immunity anti-bacterial responses; a modulator of the immune response	setA	Brucella suis 1330	Immune modulation	fabZ	(3R)-hydroxymyristoyl ACP dehydratase	46	3.91E-39	129
ECs_1286	ECs_1286	3-hydroxyacyl-[acy	VFG011402 (gb|WP_00	LPS (VF0367)	Plays a role in entry and early survival inside macrophages;	setA	Brucella suis 1330	Immune modulation	fabZ	(3R)-hydroxymyristoy	47.7	1.82E-11	59.3

		l-carrier-protein] dehydratase	4688426)		Resistance to innate-immunity anti-bacterial responses; a modulator of the immune response					l ACP dehydratase			
ECs_0181	lpxD	UDP-3-O-(3-hydroxymyristoyl)-glucosamine N-acyltransferase	VFG013165 (gb|WP_012340599)	LOS (VF0044)	Major immunogen; LOS phosphorylcholine (ChoP) may influence invasion via interaction with PAF receptor and stimulates of inflammatory signals; LPS phase variation is characterized by the spontaneous loss and gain of oligosaccharide structures present in the outer core. the phase variable expression of LPS biosynthesis genes promotes evasion of antigen-specific host immune defences and allow colonization of different host microenvironments	setA	Haemophilus somnus 2336	Immune modulation	lpxD	UDP-3-O-(3-hydroxymyristoyl) glucosamine N-acyltransferase	60.2	6.35E-110	323
ECs_0184	lpxB	lipid-A-disaccharide synthase	VFG013386 (gb|WP_012054516)	LOS (VF0044)	Major immunogen; LOS phosphorylcholine (ChoP) may influence invasion via interaction with PAF receptor and stimulates of inflammatory signals; LPS phase variation is characterized by the spontaneous loss and gain of oligosaccharide structures present in	setA	Haemophilus influenzae PittEE	Immune modulation	lpxB	lipid-A-disaccharide synthase	63.9	6.25E-167	472

					the outer core. the phase variable expression of LPS biosynthesis genes promotes evasion of antigen-specific host immune defences and allow colonization of different host microenvironments								
ECs_4920	hemE	uroporphyrinogen decarboxylase	VFG013612 (gb|WP_011609686)	Heme biosynthesis (VF0758)	-	setB	Haemophilus somnus 129PT	Nutritional/Metabolic factor	hemE	uroporphyrinogen decarboxylase	79.4	1.41E-208	575
ECs_3436	rseC	SoxR iron-sulfur cluster reduction factor component	VFG014946 (gb|WP_003150212)	Alginate (VF0091)	Allows the bacteria form biofilm; contributes to the persistence of the bacteria in the CF lung: act as an adhesin, preventing the bacteria from being expelled from the lung, and alginate slime layer makes it more difficult for phagocytes to ingest and kill the bacteria	setA	Pseudomonas aeruginosa PA7	Biofilm	mucC	negative regulator for alginate biosynthesis MucB	32.9	1.74E-13	63.9
ECs_3437	rseB	anti-sigma E factor	VFG015767 (gb|WP_012312988)	Alginate (VF0091)	Allows the bacteria form biofilm; contributes to the persistence of the bacteria in the CF lung: act as an adhesin, preventing the bacteria from being expelled from the lung, and alginate slime layer makes it more difficult for phagocytes to ingest and kill the bacteria	setA	Pseudomonas putida W619	Biofilm	mucB	anti-sigma factor MucA, inhibitor of alg gene expression	27.6	1.07E-25	104

ECs_3432	era	GTP-binding protein Era	VFG047403 (gb|WP_014547384)	FupA (VF0550)	High-affinity iron transport Protein involved in ferrous iron acquisition	setB	Francisella cf. tularensis subsp. novicida 3523	Nutritional/Metabolic factor	feoB	Fe(2+) transporter permease subunit FeoB	25.8	5.20E-06	47.8
ECs_4251	feoB	ferrous iron transporter protein B and GTP-binding protein	VFG047403 (gb|WP_014547384)	FupA (VF0550)	High-affinity iron transport Protein involved in ferrous iron acquisition	setA	Francisella cf. tularensis subsp. novicida 3523	Nutritional/Metabolic factor	feoB	Fe(2+) transporter permease subunit FeoB	46.6	1.91E-230	662
ECs_3430	pdxJ	pyridoxine 5'-phosphate synthase	VFG043390 (gb|WP_001210849)	Flagella (VF0051)	Confers motility, allows the bacteria to penetrate and colonize the gastric mucus layer. The lumenal pH of the fasting human stomach is <2, but with the gastric mucus there is a pH gradient that ranges from pH 2 at the luminal surface to nearly neutral pH at the epithelial cell surface, so entry into the gastric mucus layer is important for H. pylori to escape extremely low pH	setA	Helicobacter pylori 26695	Motility	pdxJ	pyridoxine 5'-phosphate synthase	37.8	3.51E-44	149

ECs_4933	aceA	isocitrate lyase	VFG001381 (gb|YP_177728)	Isocitrate lyase (VF0253)	Required for persistent infection	setA	Mycobacterium tuberculosis H37Rv	Others	icl	Isocitrate lyase Icl (isocitrase) (isocitratase)	62.5	8.42E-178	503
ECs_3438	rseA	anti-sigma factor	VFG014931 (gb|WP_014596050)	Alginate (VF0091)	Allows the bacteria form biofilm; contributes to the persistence of the bacteria in the CF lung: act as an adhesin, preventing the bacteria from being expelled from the lung, and alginate slime layer makes it more difficult for phagocytes to ingest and kill the bacteria	setA	Pseudomonas stutzeri A1501	Biofilm	mucA	alkaline metalloproteinase precursor	31	6.35E-08	50.8
ECs_3219	yfcR	fimbrial-like adhesin protein	VFG021584 (gb|WP_001250321)	Stf (VF0958)	-	setB	Salmonella enterica subsp. enterica serovar Newport str. SL254	Adherence	stfE	fimbrial protein	49	4.06E-48	153
ECs_4020	yraH	fimbrial-like adhesin protein	VFG042675 (gb|BAC55512)	Putative fimbriae (VF1213)	-	setA	Edwardsiella tarda str. KG8401	Adherence	etfA	major fimbrial subunit protein	33	1.98E-18	78.6
ECs_4670	lpfA	type 1	VFG004337	Stg	-	setA	Salmonella	Adherence	stgA	fimbrial	58.4	1.35E-	223

		fimbrial protein	(gb|WP_000773772)	(VF0959)			enterica subsp. enterica serovar Typhi str. CT18			protein		74	
ECs_5054	nrfC	formate-dependent nitrite reductase 4Fe4S subunit	VFG009608 (gb|WP_011730341)	Nitrate reductase (VF0302)	Nitrate respiration helps the bacteria to survive in O2-depleted areas of inflammatory or necrotic tissue	setA	Mycobacterium smegmatis str. MC2 155	Nutritional/Metabolic factor	narH	nitrate reductase subunit beta	33	2.04E-13	68.6
ECs_2070	narY	nitrate reductase 1 beta (Fe-S) subunit	VFG009606 (gb|WP_003875456)	Nitrate reductase (VF0302)	Nitrate respiration helps the bacteria to survive in O2-depleted areas of inflammatory or necrotic tissue	setA	Mycobacterium avium subsp. paratuberculosis K-10	Nutritional/Metabolic factor	narH	nitrate reductase subunit beta	57.7	5.22E-212	597
ECs_0984	ycaM	transporter	VFG047682 (gb|WP_041263746)	ArgP (VF0557)	High affinity arginine transporter mediating arginine uptake that is crucial for efficient phagosomal escape	setA	Francisella sp. TX077308	Nutritional/Metabolic factor	argP	amino acid antiporter	21.9	3.82E-15	77.4
ECs_5097	adiC	arginine:agmatine antiporter	VFG047682 (gb|WP_041263746)	ArgP (VF0557)	High affinity arginine transporter mediating arginine uptake that is crucial for efficient phagosomal escape	setA	Francisella sp. TX077308	Nutritional/Metabolic factor	argP	amino acid antiporter	23.8	9.45E-12	66.6
ECs_2097	gadC	amino	VFG047682	ArgP	High affinity arginine transporter	setA	Francisell	Nutritiona	argP	amino acid	28.7	1.64E-	192

		acid antiporter	(gb|WP_041263746)	(VF0557)	mediating arginine uptake that is crucial for efficient phagosomal escape		a sp. TX077308	l/Metabolic factor		antiporter		55
ECs_2690	rcsA	transcriptional regulator	VFG049007 (gb|WP_002911561)	RcsAB (VF0571)	RcsB combined with the unstable auxiliary regulator RcsA to bind to an RcsAB box in the promoter region to upregulate the cps genes expression	setA	Klebsiella pneumoniae subsp. pneumoniae NTUH-K2044	Regulation	rcsA	transcriptional activator for ctr capsule biosynthesis	67.6	7.76E-96	277
ECs_2878	yegD	Hsp70 chaperone family protein	VFG043573 (gb|NP_219906)	Adherence; porin (VF0713)	-	setB	Chlamydia trachomatis D/UW-3/CX	Adherence	dnaK	chaperone protein DnaK	25.2	7.38E-09	57.8
ECs_0014	dnaK	chaperone Hsp70	VFG043573 (gb|NP_219906)	Adherence; porin (VF0713)	-	setB	Chlamydia trachomatis D/UW-3/CX	Adherence	dnaK	chaperone protein DnaK	60.4	9.69E-243	685
ECs_3392	hscA	chaperone protein HscA	VFG043573 (gb|NP_219906)	Adherence; porin (VF0713)	-	setB	Chlamydia trachomatis D/UW-3/CX	Adherence	dnaK	chaperone protein DnaK	42.4	1.24E-129	395
ECs_0689	hscC	Hsp70 family chaperone Hsc62	VFG043573 (gb|NP_219906)	Adherence; porin (VF0713)	-	setB	Chlamydia trachomatis D/UW-3/CX	Adherence	dnaK	chaperone protein DnaK	34.6	8.75E-81	266
ECs_2053	curA	curcumin/dihydrocu	VFG001408 (gb|NP_21	PDIM (VF0309)	Play a mainly structural role in providing a stable base for the	setA	Mycobacterium	Immune modulation	mas	multifunctional	29.4	7.03E-08	54.3

		rcumin reductase	7456)		insertion of other lipid and also play a role as a fluidity modifier, whose function could be to modulate cell wall viscosity;PDIM is crucial for infection by masking pathogen-associated molecular patterns (PAMP) of the cell wall from the innate immune system		tuberculosis H37Rv			mycocerosic acid synthase			
ECs_2055	yncD	iron outer membrane transporter	VFG048596 (gb|WP_014229387)	Ybt (VF0564)	The phenolate siderophore Ybt contributes to evasion of the activity of lipocalin2 in the lung 聳 a host factor which neutralizes enterobactin-based iron acquisition	setA	Klebsiella oxytoca KCTC 1686	Nutritional/Metabolic factor	fyuA	yersiniabactin receptor FyuA	25.3	1.74E-27	118
ECs_4022	fimD	outer membrane usher protein FimD	VFG021350 (gb|WP_000220316)	Lpf (VF0105)	Mediate attachment to the Peyer's patches	setA	Salmonella enterica subsp. enterica serovar Agona str. SL483	Adherence	lpfC	long polar fimbrial usher protein LpfC	42	3.85E-208	611
ECs_4667	fimD	outer membrane usher protein FimD	VFG021350 (gb|WP_000220316)	Lpf (VF0105)	Mediate attachment to the Peyer's patches	setA	Salmonella enterica subsp. enterica serovar Agona str.	Adherence	lpfC	long polar fimbrial usher protein LpfC	41.4	3.68E-213	624

							SL483						
ECs_2902	yegU	ADP-ribosylglycohydrolase family protein	VFG051976 (gb|WP_012272394.1)	Putida-T6SS secreted effectors (VF1354)	-	setB	Pseudomonas putida GB-1	Effector delivery system	tri1	ADP-ribosylglycohydrolase	25.3	4.74E-10	60.1
ECs_2589	flhA_2	flagellar export pore protein	VFG043086 (gb|WP_000067016)	Peritrichous flagella (VF1154)	-	setA	Escherichia coli O157:H7 str. EDL933	Motility	flhA	flagellar biosynthesis protein FlhA	100	0	1298
ECs_2588	flhE	flagellar protein FlhE	VFG043085 (gb|WP_001259575)	Peritrichous flagella (VF1154)	-	setA	Escherichia coli O157:H7 str. EDL933	Motility	flhe	flagellar protein FlhE	100	2.28E-90	257
ECs_5276	fimD	outer membrane usher protein FimD	VFG012300 (gb|NP_313303)	Type 1 fimbriae (VF0221)	Makes an important contribution to colonization of the bladder	setA	Escherichia coli O157:H7 str. Sakai	Adherence	fimD	Outer membrane usher protein fimD precursor	100	0	1774
ECs_5277	fimF	minor component of type 1 fimbriae	VFG012306 (gb|WP_001244826)	Type 1 fimbriae (VF0221)	Makes an important contribution to colonization of the bladder	setA	Escherichia coli O157:H7 str. EDL933	Adherence	fimF	FimF protein precursor	100	5.01E-125	348
ECs_2683	fliL	flagellar biosynthe	VFG043113 (gb|WP_00	Peritrichous	-	setA	Escherichia coli	Motility	fliL	flagellar basal	100	3.14E-106	299

		sis protein	0133106)	flagella (VF1154)			O157:H7 str. EDL933			body-associated protein FliL			
ECs_4833	rhaR	transcriptional activator RhaR	VFG041670 (gb|WP_012777215)	T3SS-1 (VF1286)	-	setB	Photorhabdus asymbiotica subsp. asymbiotica ATCC 43949	Effector delivery system	PAU_RS05110	AraC family transcriptional regulator	25	9.79E-06	46.2
ECs_4832	rhaS	transcriptional activator	VFG041670 (gb|WP_012777215)	T3SS-1 (VF1286)	-	setA	Photorhabdus asymbiotica subsp. asymbiotica ATCC 43949	Effector delivery system	PAU_RS05110	AraC family transcriptional regulator	29.3	7.01E-11	61.2
ECs_2403	ydiP	transcriptional regulator	VFG041670 (gb|WP_012777215)	T3SS-1 (VF1286)	-	setA	Photorhabdus asymbiotica subsp. asymbiotica ATCC 43949	Effector delivery system	PAU_RS05110	AraC family transcriptional regulator	27	1.07E-08	55.1
ECs_2868	yegH	inner	VFG038900	Hemolysin	-	setA	Aeromonas	Exotoxin	hlyA	Hemolysin A	28.2	1.12E-	112

		membrane protein	(gb|WP_011706614)	HlyA (VF0646)			hydrophila subsp. hydrophila ATCC 7966					26	
ECs_3475	yfjD	inner membrane protein	VFG038900 (gb|WP_011706614)	Hemolysin HlyA (VF0646)	-	setA	Aeromonas hydrophila subsp. hydrophila ATCC 7966	Exotoxin	hlyA	Hemolysin A	24	2.16E-29	118
ECs_2525	yoaE	membrane protein	VFG038900 (gb|WP_011706614)	Hemolysin HlyA (VF0646)	-	setA	Aeromonas hydrophila subsp. hydrophila ATCC 7966	Exotoxin	hlyA	Hemolysin A	27.3	2.29E-27	114
ECs_5196	ytfL	inner membrane protein	VFG038900 (gb|WP_011706614)	Hemolysin HlyA (VF0646)	-	setA	Aeromonas hydrophila subsp. hydrophila ATCC 7966	Exotoxin	hlyA	Hemolysin A	59.7	3.03E-179	508
ECs_2685	fliN	flagellar motor switching and energizing component	VFG043115 (gb|WP_001282098)	Peritrichous flagella (VF1154)	-	setA	Escherichia coli O157:H7 str. EDL933	Motility	fliN	flagellar motor switch protein FliN	100	3.16E-91	260

ECs_2865	wzc	colanic acid productio n tyrosine-protein kinase	VFG048971 (gb|WP_01 5958711)	Capsule (VF0560)	Assisting in evading the host immune system by protecting bacteria from opsonophagocytosis and serum killing	setA	Klebsiella pneumoniae subsp. pneumoniae MGH 78578	Immune modulation	KPN_RS1350 0	polysaccha ride biosynthes is tyrosine autokinase	54.6	8.80E-255	721
ECs_1137	wzc	colanic acid productio n tyrosine-protein kinase	VFG048971 (gb|WP_01 5958711)	Capsule (VF0560)	Assisting in evading the host immune system by protecting bacteria from opsonophagocytosis and serum killing	setA	Klebsiella pneumoniae subsp. pneumoniae MGH 78578	Immune modulation	KPN_RS1350 0	polysaccha ride biosynthes is tyrosine autokinase	61.6	2.14E-299	835
ECs_0556	ybbO	oxidoredu ctase	VFG030269 (gb|WP_01 5303737)	MymA operon (VF0840)	-	setA	Mycobacter ium canettii CIPT 140070008	Immune modulation	sadH	SDR family NAD(P)-dep endent oxidoreduc tase	28.4	1.17E-19	85.9
ECs_0306	ECs_0306	oxidoredu ctase	VFG030266 (gb|WP_01 4001574)	MymA operon (VF0840)	-	setA	Mycobacter ium canettii CIPT 140010059	Immune modulation	sadH	SDR family NAD(P)-dep endent oxidoreduc tase	33.7	6.43E-24	96.7
ECs_4935	espL4	T3SS effector-	VFG034772 (gb|WP_00	TTSS secreted	Cif (Deamidase. Induces cytopathic effects of actin stress fiber	setA	Escherichi a coli	Effector delivery	espL4	Type III secretion	100	6.48E-306	852

like protein EspL	0627910)	effectors (VF1110)	formation and cell cycle arrest.); EspB (Pore formation, actin disruption, microvilli effacement, anti-phagocytosis.); EspF (Inducing degradation of the aniapoptic protein AbcF2, tight junction disruption, microvilli effacement and elongation, mitochondrial dysfunction, N-WASP activation, SGLT-1 inactivation, pedestal maturation, inhibition of NHE3 activity, membrane remodelling; targets and disrupts the nucleolus late in infection, which is temporally controlled by host mitochondria.); EspFu/tccP (Inducing degradation of the aniapoptic protein AbcF2, tight junction disruption, microvilli effacement and elongation, mitochondrial dysfunction, N-WASP activation, SGLT-1 inactivation, pedestal maturation, inhibition of NHE3 activity, membrane remodelling; targets and disrupts the nucleolus late in infection, which is temporally controlled by host mitochondria.); EspG (TBC-like GTPase activating	O157:H7 str. EDL933	system	system effector EspL4

protein. Efficiently catalyzes GTP hydrolysis in Rab1 to disrupt of Rab1-mediated ER-to-Golgi trafficking.); EspH (First bacterial effector acting directly on RhoGEFs, EspH directly binds to the DH-PH domain in RhoGEFs to disrupt RhoGEF-Rho signaling; critical for inhibiting macrophage phagocytosis.); EspJ (Inhibit both IgG- and complement receptor-mediated phagocytosis.); EspK; EspL1; EspL2 (Cysteine protease. Bounds F-actin-aggregating annexin 2 directly to increase annexin 2's ability to aggregate Tir-induced F-actin; block necroptosis and in flammation.); EspL4; EspM1 (GEF. Activates the RhoA signaling pathway and induce the formation of stress fibres; inhibit pedestal formation and induce tight junction mislocalization.); EspM2 (GEF. Activates the RhoA signaling pathway and induce the formation of stress fibres; inhibit pedestal formation and induce tight junction

mislocalization.); EspN; EspO1-1; EspO1-2; EspR1; EspR3; EspR4; EspT (GEF. Activates Rac1 and Cdc42 leading to formation of membrane ruffles and lamellipodia; induces membrane ruffles to facilitate bacterial invasion into non-phagocytic cells in a process involving Rac1 and Wave2.); EspW; EspX1; EspX2; EspX4; EspX5; EspX6; EspX7/nleL (E3 ubiquitin ligase, HECT-like. Modulates pedestal formation.); EspY1; EspY2; EspY3; EspY4; EspY5; Map (GEF. Mimics the host Dbl and catalyses the exchange of GDP for GTP in Cdc42, involved in effacement, SGLT1 inhibition, formation of filopodia and disruption of mitochondrial function.); NleA/espI (Disruption of tight junctions by inhibition of host cell protein trafficking through COPII-dependent pathways.); NleB1 (Blocks translocation of the p65 and to the host cell nucleus to inhibit NF-<kappa>B pathway, but NleE and NleB act at different points in the

NF-<kappa>B signaling pathway.); NleB2 (May also have anti-inflammatory activity.); NleC (Metalloprotease. Zn-dependent endopeptidases that specifically clip and inactivate RelA (p65), thus blocking NF-<kappa>B pathway.); NleD (Metalloprotease. Zn-dependent endopeptidases that specifically clip and inactivate JNK and p38, thus blocking AP-1 pathway.); NleE (PMN tran-epithelial migration; blocks translocation of the p65 to the host cell nucleus by preventing I<kappa>B degradation to inhibit NF-<kappa>B pathway.); NleF; NleG-1; NleG2-2; NleG2-3; NleG2-4; NleG5-1; NleG5-2; NleG6-1; NleG6-2; NleG6-3; NleG7 (U-box type E3 ubiquitin ligases.); NleG8-2; NleH1 (Ser/Thr protein kinase. Binds directly to a subunit of NF-<kappa>B, the ribosomal protein S3 (RPS3), reducing the nuclear abundance of RPS3 to dampen host transcriptional outputs; interact with Bax inhibitor-1 to block apoptosis.); NleH2 (Putative kinase.

					Attenuates NF-<kappa>B pathway.); SepZ/espZ (EspZ interacts with CD98 in host cell membranes to promote host cell survival, therefore provide the pathogen with valuable time to colonize efficiently prior to dissemination.); TccP2; Tir (Mimics host immunoreceptor tyrosine-based inhibition motifs (ITIMs), also see helicobacter CagA. EHEC Tir lacks the Nck binding site. Conserved NPY (Asn-Pro-Tyr) motif recruits the adaptor protein IRTKS and/or IRSp53. IRTKS/IRSp53 link Tir and TccP/EspFu, which in turn activates N-WASP; Receptor for intimin; effacement; SGLT1 inhibition; recruits SHIP2 to control actin-pedestal morphology; maintains the integrity of the epithelium by keeping the destructive activity of EspG and EspG2 in check.)								
ECs_2427	espL1	T3SS effector-like protein EspL	VFG034772 (gb|WP_000627910)	TTSS secreted effectors (VF1110)	Cif (Deamidase. Induces cytopathic effects of actin stress fiber formation and cell cycle arrest.); EspB (Pore formation, actin disruption, microvilli effacement,	setA	Escherichia coli O157:H7 str. EDL933	Effector delivery system	espL4	Type III secretion system effector EspL4	33.4	2.29E-46	174

anti-phagocytosis.); EspF (Inducing degradation of the aniapoptic protein AbcF2, tight junction disruption, microvilli effacement and elongation, mitochondrial dysfunction, N-WASP activation, SGLT-1 inactivation, pedestal maturation, inhibition of NHE3 activity, membrane remodelling; targets and disrupts the nucleolus late in infection, which is temporally controlled by host mitochondria.); EspFu/tccP (Inducing degradation of the aniapoptic protein AbcF2, tight junction disruption, microvilli effacement and elongation, mitochondrial dysfunction, N-WASP activation, SGLT-1 inactivation, pedestal maturation, inhibition of NHE3 activity, membrane remodelling; targets and disrupts the nucleolus late in infection, which is temporally controlled by host mitochondria.); EspG (TBC-like GTPase activating protein. Efficiently catalyzes GTP hydrolysis in Rab1 to disrupt of Rab1-mediated ER-to-Golgi

trafficking.); EspH (First bacterial effector acting directly on RhoGEFs, EspH directly binds to the DH-PH domain in RhoGEFs to disrupt RhoGEF-Rho signaling; critical for inhibiting macrophage phagocytosis.); EspJ (Inhibit both IgG- and complement receptor-mediated phagocytosis.); EspK; EspL1; EspL2 (Cysteine protease. Bounds F-actin-aggregating annexin 2 directly to increase annexin 2's ability to aggregate Tir-induced F-actin; block necroptosis and in flammation.); EspL4; EspM1 (GEF. Activates the RhoA signaling pathway and induce the formation of stress fibres; inhibit pedestal formation and induce tight junction mislocalization.); EspM2 (GEF. Activates the RhoA signaling pathway and induce the formation of stress fibres; inhibit pedestal formation and induce tight junction mislocalization.); EspN; EspO1-1; EspO1-2; EspR1; EspR3; EspR4; EspT (GEF. Activates Rac1 and Cdc42 leading

to formation of membrane ruffles and lamellipodia; induces membrane ruffles to facilitate bacterial invasion into non-phagocytic cells in a process involving Rac1 and Wave2.); EspW; EspX1; EspX2; EspX4; EspX5; EspX6; EspX7/nleL (E3 ubiquitin ligase, HECT-like. Modulates pedestal formation.); EspY1; EspY2; EspY3; EspY4; EspY5; Map (GEF. Mimics the host Dbl and catalyses the exchange of GDP for GTP in Cdc42, involved in effacement, SGLT1 inhibition, formation of filopodia and disruption of mitochondrial function.); NleA/espI (Disruption of tight junctions by inhibition of host cell protein trafficking through COPII-dependent pathways.); NleB1 (Blocks translocation of the p65 and to the host cell nucleus to inhibit NF-<kappa>B pathway, but NleE and NleB act at different points in the NF-<kappa>B signaling pathway.); NleB2 (May also have anti-inflammatory activity.); NleC (Metalloprotease.

Zn-dependent endopeptidases that specifically clip and inactivate RelA (p65), thus blocking NF-<kappa>B pathway.); NleD (Metalloprotease. Zn-dependent endopeptidases that specifically clip and inactivate JNK and p38, thus blocking AP-1 pathway.); NleE (PMN tran-epithelial migration; blocks translocation of the p65 to the host cell nucleus by preventing I<kappa>B degradation to inhibit NF-<kappa>B pathway.); NleF; NleG-1; NleG2-2; NleG2-3; NleG2-4; NleG5-1; NleG5-2; NleG6-1; NleG6-2; NleG6-3; NleG7 (U-box type E3 ubiquitin ligases.); NleG8-2; NleH1 (Ser/Thr protein kinase. Binds directly to a subunit of NF-<kappa>B, the ribosomal protein S3 (RPS3), reducing the nuclear abundance of RPS3 to dampen host transcriptional outputs; interact with Bax inhibitor-1 to block apoptosis.); NleH2 (Putative kinase. Attenuates NF-<kappa>B pathway.); SepZ/espZ (EspZ interacts with CD98 in host cell membranes to promote host

					cell survival, therefore provide the pathogen with valuable time to colonize efficiently prior to dissemination.); TccP2; Tir (Mimics host immunoreceptor tyrosine-based inhibition motifs (ITIMs), also see helicobacter CagA. EHEC Tir lacks the Nck binding site. Conserved NPY (Asn-Pro-Tyr) motif recruits the adaptor protein IRTKS and/or IRSp53. IRTKS/IRSp53 link Tir and TccP/EspFu, which in turn activates N-WASP; Receptor for intimin; effacement; SGLT1 inhibition; recruits SHIP2 to control actin-pedestal morphology; maintains the integrity of the epithelium by keeping the destructive activity of EspG and EspG2 in check.)								
ECs_5326	bglJ	bgl operon transcriptional activator	VFG049018 (gb|WP_002913007)	RcsAB (VF0571)	RcsB combined with the unstable auxiliary regulator RcsA to bind to an RcsAB box in the promoter region to upregulate the cps genes expression	setA	Klebsiella pneumoniae subsp. pneumoniae NTUH-K2044	Regulation	rcsB	transcriptional regulator RcsB	32.7	1.19E-09	56.2
ECs_3106	rcsB	two-component regulator	VFG049018 (gb|WP_002913007)	RcsAB (VF0571)	RcsB combined with the unstable auxiliary regulator RcsA to bind to an RcsAB box in the promoter region to	setA	Klebsiella pneumoniae subsp.	Regulation	rcsB	transcriptional regulator	96.8	9.61E-145	402

		y system response regulator RcsB			upregulate the cps genes expression		pneumoniae NTUH-K2044			RcsB			
ECs_0597	sfmZ	response regulator	VFG000449 (gb|NP_459544)	Type 1 fimbriae (VF0102)	The adhesin FimH mediates T3SS1-independent uptake in murine DCs.	setA	Salmonella enterica subsp. enterica serovar Typhimurium str. LT2	Adherence	fimZ	DNA-binding response regulator	71.9	4.12E-104	298
ECs_3248	evgA	two-component regulatory system response regulator EvgA	VFG000449 (gb|NP_459544)	Type 1 fimbriae (VF0102)	The adhesin FimH mediates T3SS1-independent uptake in murine DCs.	setA	Salmonella enterica subsp. enterica serovar Typhimurium str. LT2	Adherence	fimZ	DNA-binding response regulator	32.2	3.79E-38	130
ECs_2863	wcaB	acyl transferase	VFG005975 (gb|WP_011226040)	Capsule (VF0144)	Resistant to complement deposition and masks cell wall-associated complement from being recognized by the complement receptors on phagocytes	setA	Streptococcus thermophilus LMG 18311	Immune modulation	STU_RS14610	serine acetyltransferase	38.6	2.01E-19	79.7
ECs_2638	ECs_2638	hypothetical protein	VFG005975 (gb|WP_011226040)	Capsule (VF0144)	Resistant to complement deposition and masks cell wall-associated complement from being recognized by the	setB	Streptococcus thermophil	Immune modulation	STU_RS14610	serine acetyltransferase	38	1.59E-10	57

<table>
<tr><th></th><th></th><th></th><th></th><th></th><th></th><th></th><th></th><th></th><th></th><th></th><th></th><th></th><th></th></tr>
<tr><td></td><td></td><td></td><td></td><td></td><td>complement receptors on phagocytes</td><td></td><td>us LMG 18311</td><td></td><td></td><td></td><td></td><td></td><td></td></tr>
<tr><td>ECs_4485</td><td>cysE</td><td>serine acetyltransferase</td><td>VFG005975 (gb|WP_011226040)</td><td>Capsule (VF0144)</td><td>Resistant to complement deposition and masks cell wall-associated complement from being recognized by the complement receptors on phagocytes</td><td>setA</td><td>Streptococcus thermophilus LMG 18311</td><td>Immune modulation</td><td>STU_RS14610</td><td>serine acetyltransferase</td><td>43.8</td><td>3.42E-15</td><td>71.2</td></tr>
<tr><td>ECs_0512</td><td>maa</td><td>maltose O-acetyltransferase</td><td>VFG016409 (gb|WP_001103738)</td><td>Polysaccharide capsule (VF0659)</td><td>-</td><td>setA</td><td>Bacillus thuringiensis serovar konkukian str. 97-27</td><td>Immune modulation</td><td>BT9727_RS25815</td><td>serine O-acetyltransferase</td><td>38</td><td>5.57E-08</td><td>49.7</td></tr>
<tr><td>ECs_5278</td><td>fimG</td><td>minor component of type 1 fimbriae</td><td>VFG012313 (gb|WP_000872005)</td><td>Type 1 fimbriae (VF0221)</td><td>Makes an important contribution to colonization of the bladder</td><td>setA</td><td>Escherichia coli O157:H7 str. EDL933</td><td>Adherence</td><td>fimG</td><td>FimG protein precursor</td><td>100</td><td>1.10E-110</td><td>311</td></tr>
<tr><td>ECs_2108</td><td>ydeR</td><td>fimbrial-like adhesin protein</td><td>VFG012313 (gb|WP_000872005)</td><td>Type 1 fimbriae (VF0221)</td><td>Makes an important contribution to colonization of the bladder</td><td>setA</td><td>Escherichia coli O157:H7 str. EDL933</td><td>Adherence</td><td>fimG</td><td>FimG protein precursor</td><td>57.8</td><td>3.61E-63</td><td>191</td></tr>
<tr><td>ECs_2679</td><td>fliH</td><td>negative regulator of FliI ATPase activity</td><td>VFG043109 (gb|WP_001282715)</td><td>Peritrichous flagella (VF1154)</td><td>-</td><td>setA</td><td>Escherichia coli O157:H7 str. EDL933</td><td>Motility</td><td>fliH</td><td>flagellar assembly protein FliH</td><td>100</td><td>2.61E-132</td><td>371</td></tr>
<tr><td>ECs_5332</td><td>yjjG</td><td>pyrimidin</td><td>VFG011675</td><td>Capsule</td><td>Play an important role in bacterial</td><td>setB</td><td>Campylobac</td><td>Immune</td><td>CJE_RS0797</td><td>HAD-IA</td><td>28.8</td><td>9.46E-</td><td>54.7</td></tr>
</table>

		e 5'-nucleotidase	(gb|WP_002867275)	(VF0323)	survival and persistence in the environment and evasion of host immune response; the presence of heptose residues in the capsule may be important for virulence. Heptose residues found in some cell surface-located glycoconjugates are required for adhesion		ter jejuni RM1221	modulation	5	family hydrolase		09	
ECs_2672	espR3	T3SS effector-like protein EspR	VFG034828 (gb|WP_000484277)	TTSS secreted effectors (VF1110)	Cif (Deamidase. Induces cytopathic effects of actin stress fiber formation and cell cycle arrest.); EspB (Pore formation, actin disruption, microvilli effacement, anti-phagocytosis.); EspF (Inducing degradation of the aniapoptic protein AbcF2, tight junction disruption, microvilli effacement and elongation, mitochondrial dysfunction, N-WASP activation, SGLT-1 inactivation, pedestal maturation, inhibition of NHE3 activity, membrane remodelling; targets and disrupts the nucleolus late in infection, which is temporally controlled by host mitochondria.); EspFu/tccP (Inducing degradation of the aniapoptic protein AbcF2, tight	setA	Escherichia coli O157:H7 str. EC4115	Effector delivery system	espR3	Type III secretion system effector espR3	100	6.95E-210	580

junction disruption, microvilli effacement and elongation, mitochondrial dysfunction, N-WASP activation, SGLT-1 inactivation, pedestal maturation, inhibition of NHE3 activity, membrane remodelling; targets and disrupts the nucleolus late in infection, which is temporally controlled by host mitochondria.); EspG (TBC-like GTPase activating protein. Efficiently catalyzes GTP hydrolysis in Rab1 to disrupt of Rab1-mediated ER-to-Golgi trafficking.); EspH (First bacterial effector acting directly on RhoGEFs, EspH directly binds to the DH-PH domain in RhoGEFs to disrupt RhoGEF-Rho signaling; critical for inhibiting macrophage phagocytosis.); EspJ (Inhibit both IgG- and complement receptor-mediated phagocytosis.); EspK; EspL1; EspL2 (Cysteine protease. Bounds F-actin-aggregating annexin 2 directly to increase annexin 2's ability to aggregate Tir-induced F-actin; block necroptosis and in

flammation.); EspL4; EspM1 (GEF. Activates the RhoA signaling pathway and induce the formation of stress fibres; inhibit pedestal formation and induce tight junction mislocalization.); EspM2 (GEF. Activates the RhoA signaling pathway and induce the formation of stress fibres; inhibit pedestal formation and induce tight junction mislocalization.); EspN; EspO1-1; EspO1-2; EspR1; EspR3; EspR4; EspT (GEF. Activates Rac1 and Cdc42 leading to formation of membrane ruffles and lamellipodia; induces membrane ruffles to facilitate bacterial invasion into non-phagocytic cells in a process involving Rac1 and Wave2.); EspW; EspX1; EspX2; EspX4; EspX5; EspX6; EspX7/nleL (E3 ubiquitin ligase, HECT-like. Modulates pedestal formation.); EspY1; EspY2; EspY3; EspY4; EspY5; Map (GEF. Mimics the host Dbl and catalyses the exchange of GDP for GTP in Cdc42, involved in effacement, SGLT1 inhibition,

formation of filopodia and disruption of mitochondrial function.); NleA/espI (Disruption of tight junctions by inhibition of host cell protein trafficking through COPII-dependent pathways.); NleB1 (Blocks translocation of the p65 and to the host cell nucleus to inhibit NF-<kappa>B pathway, but NleE and NleB act at different points in the NF-<kappa>B signaling pathway.); NleB2 (May also have anti-inflammatory activity.); NleC (Metalloprotease. Zn-dependent endopeptidases that specifically clip and inactivate RelA (p65), thus blocking NF-<kappa>B pathway.); NleD (Metalloprotease. Zn-dependent endopeptidases that specifically clip and inactivate JNK and p38, thus blocking AP-1 pathway.); NleE (PMN tran-epithelial migration; blocks translocation of the p65 to the host cell nucleus by preventing I<kappa>B degradation to inhibit NF-<kappa>B pathway.); NleF; NleG-1; NleG2-2; NleG2-3; NleG2-4; NleG5-1;

NleG5-2; NleG6-1; NleG6-2; NleG6-3; NleG7 (U-box type E3 ubiquitin ligases.); NleG8-2; NleH1 (Ser/Thr protein kinase. Binds directly to a subunit of NF-<kappa>B, the ribosomal protein S3 (RPS3), reducing the nuclear abundance of RPS3 to dampen host transcriptional outputs; interact with Bax inhibitor-1 to block apoptosis.); NleH2 (Putative kinase. Attenuates NF-<kappa>B pathway.); SepZ/espZ (EspZ interacts with CD98 in host cell membranes to promote host cell survival, therefore provide the pathogen with valuable time to colonize efficiently prior to dissemination.); TccP2; Tir (Mimics host immunoreceptor tyrosine-based inhibition motifs (ITIMs), also see helicobacter CagA. EHEC Tir lacks the Nck binding site. Conserved NPY (Asn-Pro-Tyr) motif recruits the adaptor protein IRTKS and/or IRSp53. IRTKS/IRSp53 link Tir and TccP/EspFu, which in turn activates N-WASP; Receptor for intimin; effacement;

ECs_2674	espR4	T3SS effector-like protein EspR	VFG034828 (gb|WP_000484277)	TTSS secreted effectors (VF1110)	SGLT1 inhibition; recruits SHIP2 to control actin-pedestal morphology; maintains the integrity of the epithelium by keeping the destructive activity of EspG and EspG2 in check.) Cif (Deamidase. Induces cytopathic effects of actin stress fiber formation and cell cycle arrest.); EspB (Pore formation, actin disruption, microvilli effacement, anti-phagocytosis.); EspF (Inducing degradation of the aniapoptic protein AbcF2, tight junction disruption, microvilli effacement and elongation, mitochondrial dysfunction, N-WASP activation, SGLT-1 inactivation, pedestal maturation, inhibition of NHE3 activity, membrane remodelling; targets and disrupts the nucleolus late in infection, which is temporally controlled by host mitochondria.); EspFu/tccP (Inducing degradation of the aniapoptic protein AbcF2, tight junction disruption, microvilli effacement and elongation, mitochondrial dysfunction, N-WASP	setA	Escherichia coli O157:H7 str. EC4115	Effector delivery system	espR3	Type III secretion system effector espR3	95.8	3.82E-200	555

activation, SGLT-1 inactivation, pedestal maturation, inhibition of NHE3 activity, membrane remodelling; targets and disrupts the nucleolus late in infection, which is temporally controlled by host mitochondria.); EspG (TBC-like GTPase activating protein. Efficiently catalyzes GTP hydrolysis in Rab1 to disrupt of Rab1-mediated ER-to-Golgi trafficking.); EspH (First bacterial effector acting directly on RhoGEFs, EspH directly binds to the DH-PH domain in RhoGEFs to disrupt RhoGEF-Rho signaling; critical for inhibiting macrophage phagocytosis.); EspJ (Inhibit both IgG- and complement receptor-mediated phagocytosis.); EspK; EspL1; EspL2 (Cysteine protease. Bounds F-actin-aggregating annexin 2 directly to increase annexin 2's ability to aggregate Tir-induced F-actin; block necroptosis and in flammation.); EspL4; EspM1 (GEF. Activates the RhoA signaling pathway and induce the formation of stress

fibres; inhibit pedestal formation and induce tight junction mislocalization.); EspM2 (GEF. Activates the RhoA signaling pathway and induce the formation of stress fibres; inhibit pedestal formation and induce tight junction mislocalization.); EspN; EspO1-1; EspO1-2; EspR1; EspR3; EspR4; EspT (GEF. Activates Rac1 and Cdc42 leading to formation of membrane ruffles and lamellipodia; induces membrane ruffles to facilitate bacterial invasion into non-phagocytic cells in a process involving Rac1 and Wave2.); EspW; EspX1; EspX2; EspX4; EspX5; EspX6; EspX7/nleL (E3 ubiquitin ligase, HECT-like. Modulates pedestal formation.); EspY1; EspY2; EspY3; EspY4; EspY5; Map (GEF. Mimics the host Dbl and catalyses the exchange of GDP for GTP in Cdc42, involved in effacement, SGLT1 inhibition, formation of filopodia and disruption of mitochondrial function.); NleA/espI (Disruption of tight

junctions by inhibition of host cell protein trafficking through COPII-dependent pathways.); NleB1 (Blocks translocation of the p65 and to the host cell nucleus to inhibit NF-<kappa>B pathway, but NleE and NleB act at different points in the NF-<kappa>B signaling pathway.); NleB2 (May also have anti-inflammatory activity.); NleC (Metalloprotease. Zn-dependent endopeptidases that specifically clip and inactivate RelA (p65), thus blocking NF-<kappa>B pathway.); NleD (Metalloprotease. Zn-dependent endopeptidases that specifically clip and inactivate JNK and p38, thus blocking AP-1 pathway.); NleE (PMN tran-epithelial migration; blocks translocation of the p65 to the host cell nucleus by preventing I<kappa>B degradation to inhibit NF-<kappa>B pathway.); NleF; NleG-1; NleG2-2; NleG2-3; NleG2-4; NleG5-1; NleG5-2; NleG6-1; NleG6-2; NleG6-3; NleG7 (U-box type E3 ubiquitin ligases.); NleG8-2; NleH1 (Ser/Thr

protein kinase. Binds directly to a subunit of NF-<kappa>B, the ribosomal protein S3 (RPS3), reducing the nuclear abundance of RPS3 to dampen host transcriptional outputs; interact with Bax inhibitor-1 to block apoptosis.); NleH2 (Putative kinase. Attenuates NF-<kappa>B pathway.); SepZ/espZ (EspZ interacts with CD98 in host cell membranes to promote host cell survival, therefore provide the pathogen with valuable time to colonize efficiently prior to dissemination.); TccP2; Tir (Mimics host immunoreceptor tyrosine-based inhibition motifs (ITIMs), also see helicobacter CagA. EHEC Tir lacks the Nck binding site. Conserved NPY (Asn-Pro-Tyr) motif recruits the adaptor protein IRTKS and/or IRSp53. IRTKS/IRSp53 link Tir and TccP/EspFu, which in turn activates N-WASP; Receptor for intimin; effacement; SGLT1 inhibition; recruits SHIP2 to control actin-pedestal morphology; maintains the integrity of the

					epithelium by keeping the destructive activity of EspG and EspG2 in check.)								
ECs_2677	fliF	flagellar basal-body MS-ring and collar protein	VFG043107 (gb|WP_000994443)	Peritrichous flagella (VF1154)	-	setA	Escherichia coli O157:H7 str. EDL933	Motility	fliF	flagellar M-ring protein FliF	100	0	1043
ECs_2676	fliE	flagellar basal-body component	VFG043106 (gb|WP_001274299)	Peritrichous flagella (VF1154)	-	setA	Escherichia coli O157:H7 str. EDL933	Motility	fliE	flagellar hook-basal body complex protein FliE	100	1.59E-64	190
ECs_0670	dacA	D-alanyl-D-alanine carboxypeptidase	VFG038250 (gb|YP_001083314)	PbpG (VF0468)	Penicillin-binding proteins (PBPs) are most commonly associated with binding to and inactivating b-lactam antibiotics; PBPs also participate in the final steps of the biosynthesis of the peptidoglycan layer and thus contribute to bacterial cell stability	setA	Acinetobacter baumannii ATCC 17978	Immune modulation	pbpG	D-alanyl-D-alanine endopeptidase	26.4	3.82E-17	80.5
ECs_0919	dacC	D-alanyl-D-alanine carboxypeptidase penicilli	VFG038250 (gb|YP_001083314)	PbpG (VF0468)	Penicillin-binding proteins (PBPs) are most commonly associated with binding to and inactivating b-lactam antibiotics; PBPs also participate in the final steps of the biosynthesis of	setA	Acinetobacter baumannii ATCC 17978	Immune modulation	pbpG	D-alanyl-D-alanine endopeptidase	29.3	1.25E-21	93.2

		n-binding protein 6a			the peptidoglycan layer and thus contribute to bacterial cell stability							
ECs_3021	pbpG	D-alanyl-D-alanine endopeptidase	VFG038250 (gb|YP_001083314)	PbpG (VF0468)	Penicillin-binding proteins (PBPs) are most commonly associated with binding to and inactivating b-lactam antibiotics; PBPs also participate in the final steps of the biosynthesis of the peptidoglycan layer and thus contribute to bacterial cell stability	setA	Acinetobacter baumannii ATCC 17978	Immune modulation	pbpG	D-alanyl-D-alanine endopeptidase	42.9	2.00E-57	185
ECs_4421	dppD	dipeptide/heme ABC transporter ATPase	VFG001206 (gb|WP_002219640)	FbpABC (VF0272)	Encodes a periplasmic-binding protein-dependent iron transport system necessary for the utilization of iron bound to transferrin or iron chelates, FbpA is the periplasmic Fe3+ binding protein	setA	Neisseria meningitidis MC58	Nutritional/Metabolic factor	fbpC	iron(III) ABC transporter, ATP-binding protein	29.3	1.35E-20	90.9
ECs_5285	iraD	DNA replication protein IraD	VFG041219 (gb|WP_012907481)	CTS2 (VF1264)	-	setB	Citrobacter rodentium ICC168	Effector delivery system	iraD	anti-adapter protein IraD	49.5	2.74E-27	97.8
ECs_0893	opgE	OPG biosynthetic transmembrane	VFG013382 (gb|WP_041174828)	LOS (VF0044)	Major immunogen; LOS phosphorylcholine (ChoP) may influence invasion via interaction with PAF receptor and stimulates of inflammatory signals; LPS phase	setA	Haemophilus influenzae PittGG	Immune modulation	yhbX	phosphoethanolamine transferase	32.7	2.61E-78	254

		phosphoethanolamine transferase			variation is characterized by the spontaneous loss and gain of oligosaccharide structures present in the outer core. the phase variable expression of LPS biosynthesis genes promotes evasion of antigen-specific host immune defences and allow colonization of different host microenvironments							
ECs_4053	yhbX	EptAB family phosphoethanolamine transferase	VFG013382 (gb|WP_041174828)	LOS (VF0044)	Major immunogen; LOS phosphorylcholine (ChoP) may influence invasion via interaction with PAF receptor and stimulates of inflammatory signals; LPS phase variation is characterized by the spontaneous loss and gain of oligosaccharide structures present in the outer core. the phase variable expression of LPS biosynthesis genes promotes evasion of antigen-specific host immune defences and allow colonization of different host microenvironments	setA	Haemophilus influenzae PittGG	Immune modulation	yhbX	phosphoethanolamine transferase	30.4	1.07E-31	127
ECs_5290	ECs_5290	invasin	VFG035938 (gb|WP_000907441)	Enteroaggregative immunoglo	-	setA	Escherichia coli O17:K52:H1	Adherence	air/eaeX	inverse autotransporter	41.5	9.75E-300	944

				bulin repeat protein (VF1124)			8 str. UMN026			adhesin EaeX/Air			
ECs_0336	ECs_0336	invasin	VFG034543 (gb|WP_000092594)	EaeH (VF1119)	-	setA	Escherichia coli O157:H7 str. EDL933	Adherence	eaeH	intimin-like adhesin FdeC	100	0	2704
ECs_5294	yjiK	hypothetical protein	VFG033711 (gb|WP_000823904)	TTSS (VF0191)	Injects Tir and other effector molecules directly into the host cell. Effector molecules activate cell-signaling pathways, causing alterations in the host cell cytoskeleton and resulting in the depolymerization of actin and the loss of microvilli	setA	Escherichia coli O127:H6 str. E2348/69	Effector delivery system	rorf1	YjiK family protein	33.2	2.25E-32	120
ECs_4591	ECs_4591	hypothetical protein	VFG033711 (gb|WP_000823904)	TTSS (VF0191)	Injects Tir and other effector molecules directly into the host cell. Effector molecules activate cell-signaling pathways, causing alterations in the host cell cytoskeleton and resulting in the depolymerization of actin and the loss of microvilli	setA	Escherichia coli O127:H6 str. E2348/69	Effector delivery system	rorf1	YjiK family protein	99.3	2.37E-191	524
ECs_5295	EspX6	T3SS effector-	VFG034881 (gb|NP_31	TTSS secreted	Cif (Deamidase. Induces cytopathic effects of actin stress fiber	setA	Escherichia coli	Effector delivery	espX6	Type III secretion	100	0	995

like protein EspX	3322)	effectors (VF1110)	formation and cell cycle arrest.); EspB (Pore formation, actin disruption, microvilli effacement, anti-phagocytosis.); EspF (Inducing degradation of the aniapoptic protein AbcF2, tight junction disruption, microvilli effacement and elongation, mitochondrial dysfunction, N-WASP activation, SGLT-1 inactivation, pedestal maturation, inhibition of NHE3 activity, membrane remodelling; targets and disrupts the nucleolus late in infection, which is temporally controlled by host mitochondria.); EspFu/tccP (Inducing degradation of the aniapoptic protein AbcF2, tight junction disruption, microvilli effacement and elongation, mitochondrial dysfunction, N-WASP activation, SGLT-1 inactivation, pedestal maturation, inhibition of NHE3 activity, membrane remodelling; targets and disrupts the nucleolus late in infection, which is temporally controlled by host mitochondria.); EspG (TBC-like GTPase activating	O157:H7 str. Sakai	system	system effector EspX6

protein. Efficiently catalyzes GTP hydrolysis in Rab1 to disrupt of Rab1-mediated ER-to-Golgi trafficking.); EspH (First bacterial effector acting directly on RhoGEFs, EspH directly binds to the DH-PH domain in RhoGEFs to disrupt RhoGEF-Rho signaling; critical for inhibiting macrophage phagocytosis.); EspJ (Inhibit both IgG- and complement receptor-mediated phagocytosis.); EspK; EspL1; EspL2 (Cysteine protease. Bounds F-actin-aggregating annexin 2 directly to increase annexin 2's ability to aggregate Tir-induced F-actin; block necroptosis and in flammation.); EspL4; EspM1 (GEF. Activates the RhoA signaling pathway and induce the formation of stress fibres; inhibit pedestal formation and induce tight junction mislocalization.); EspM2 (GEF. Activates the RhoA signaling pathway and induce the formation of stress fibres; inhibit pedestal formation and induce tight junction

mislocalization.); EspN; EspO1-1; EspO1-2; EspR1; EspR3; EspR4; EspT (GEF. Activates Rac1 and Cdc42 leading to formation of membrane ruffles and lamellipodia; induces membrane ruffles to facilitate bacterial invasion into non-phagocytic cells in a process involving Rac1 and Wave2.); EspW; EspX1; EspX2; EspX4; EspX5; EspX6; EspX7/nleL (E3 ubiquitin ligase, HECT-like. Modulates pedestal formation.); EspY1; EspY2; EspY3; EspY4; EspY5; Map (GEF. Mimics the host Dbl and catalyses the exchange of GDP for GTP in Cdc42, involved in effacement, SGLT1 inhibition, formation of filopodia and disruption of mitochondrial function.); NleA/espI (Disruption of tight junctions by inhibition of host cell protein trafficking through COPII-dependent pathways.); NleB1 (Blocks translocation of the p65 and to the host cell nucleus to inhibit NF-<kappa>B pathway, but NleE and NleB act at different points in the

NF-<kappa>B signaling pathway.); NleB2 (May also have anti-inflammatory activity.); NleC (Metalloprotease. Zn-dependent endopeptidases that specifically clip and inactivate RelA (p65), thus blocking NF-<kappa>B pathway.); NleD (Metalloprotease. Zn-dependent endopeptidases that specifically clip and inactivate JNK and p38, thus blocking AP-1 pathway.); NleE (PMN tran-epithelial migration; blocks translocation of the p65 to the host cell nucleus by preventing I<kappa>B degradation to inhibit NF-<kappa>B pathway.); NleF; NleG-1; NleG2-2; NleG2-3; NleG2-4; NleG5-1; NleG5-2; NleG6-1; NleG6-2; NleG6-3; NleG7 (U-box type E3 ubiquitin ligases.); NleG8-2; NleH1 (Ser/Thr protein kinase. Binds directly to a subunit of NF-<kappa>B, the ribosomal protein S3 (RPS3), reducing the nuclear abundance of RPS3 to dampen host transcriptional outputs; interact with Bax inhibitor-1 to block apoptosis.); NleH2 (Putative kinase.

					Attenuates NF-<kappa>B pathway.); SepZ/espZ (EspZ interacts with CD98 in host cell membranes to promote host cell survival, therefore provide the pathogen with valuable time to colonize efficiently prior to dissemination.); TccP2; Tir (Mimics host immunoreceptor tyrosine-based inhibition motifs (ITIMs), also see helicobacter CagA. EHEC Tir lacks the Nck binding site. Conserved NPY (Asn-Pro-Tyr) motif recruits the adaptor protein IRTKS and/or IRSp53. IRTKS/IRSp53 link Tir and TccP/EspFu, which in turn activates N-WASP; Receptor for intimin; effacement; SGLT1 inhibition; recruits SHIP2 to control actin-pedestal morphology; maintains the integrity of the epithelium by keeping the destructive activity of EspG and EspG2 in check.)								
ECs_1772	paa	porcine attaching-effacing associated protein	VFG000839 (gb|WP_001449026)	Paa (VF0194)	Involved in the initial bacterial adherence, required for the A/E activity	setA	Escherichia coli O157:H7 str. EDL933	Adherence	paa	outer membrane adhesin Paa	100	1.62E-188	515

		Paa/adherence factor AdfO											
ECs_1807	ECs_1807	outer membrane precursor Lom	VFG000442 (gb|NP_490501)	Rck (VF0108)	Confers serum resistance by binding human complement factor H.; Rck mediates cell invasion through a Zipper-like mechanism, as described for other bacteria such as Listeria and Yersinia. Salmonella seems to be the first bacterium found to be able to induce both Zipper-like mechanism and the Trigger mechanism mediated by its T3SS-1 apparatus.	setA	Salmonella enterica subsp. enterica serovar Typhimurium str. LT2	Immune modulation	rck	resistance to complement killing	38.5	3.20E-32	114
ECs_1122	ECs_1122	outer membrane precursor Lom	VFG000442 (gb|NP_490501)	Rck (VF0108)	Confers serum resistance by binding human complement factor H.; Rck mediates cell invasion through a Zipper-like mechanism, as described for other bacteria such as Listeria and Yersinia. Salmonella seems to be the first bacterium found to be able to induce both Zipper-like mechanism and the Trigger mechanism mediated by its T3SS-1 apparatus.	setA	Salmonella enterica subsp. enterica serovar Typhimurium str. LT2	Immune modulation	rck	resistance to complement killing	35.4	1.27E-31	113
ECs_3850	ECs_3850	PagC-like membrane	VFG000442 (gb|NP_49	Rck (VF0108)	Confers serum resistance by binding human complement factor H.; Rck	setA	Salmonella enterica	Immune modulation	rck	resistance to	39.2	1.78E-34	119

		protein	0501)		mediates cell invasion through a Zipper-like mechanism, as described for other bacteria such as Listeria and Yersinia. Salmonella seems to be the first bacterium found to be able to induce both Zipper-like mechanism and the Trigger mechanism mediated by its T3SS-1 apparatus.		subsp. enterica serovar Typhimurium str. LT2			complement killing			
ECs_2718	ECs_2718	outer membrane precursor Lom	VFG000442 (gb|NP_490501)	Rck (VF0108)	Confers serum resistance by binding human complement factor H.; Rck mediates cell invasion through a Zipper-like mechanism, as described for other bacteria such as Listeria and Yersinia. Salmonella seems to be the first bacterium found to be able to induce both Zipper-like mechanism and the Trigger mechanism mediated by its T3SS-1 apparatus.	setA	Salmonella enterica subsp. enterica serovar Typhimurium str. LT2	Immune modulation	rck	resistance to complement killing	37	2.53E-31	112
ECs_1649	ECs_1649	outer membrane precursor Lom	VFG000442 (gb|NP_490501)	Rck (VF0108)	Confers serum resistance by binding human complement factor H.; Rck mediates cell invasion through a Zipper-like mechanism, as described for other bacteria such as Listeria and Yersinia. Salmonella seems to be the first bacterium found to be able to induce both Zipper-like mechanism and	setA	Salmonella enterica subsp. enterica serovar Typhimurium str. LT2	Immune modulation	rck	resistance to complement killing	37	8.08E-33	116

					the Trigger mechanism mediated by its T3SS-1 apparatus.								
ECs_0843	ECs_0843	outer membrane precursor Lom	VFG000442 (gb|NP_490501)	Rck (VF0108)	Confers serum resistance by binding human complement factor H.; Rck mediates cell invasion through a Zipper-like mechanism, as described for other bacteria such as Listeria and Yersinia. Salmonella seems to be the first bacterium found to be able to induce both Zipper-like mechanism and the Trigger mechanism mediated by its T3SS-1 apparatus.	setA	Salmonella enterica subsp. enterica serovar Typhimurium str. LT2	Immune modulation	rck	resistance to complement killing	35.9	4.38E-29	106
ECs_1991	ECs_1991	outer membrane precursor Lom	VFG000442 (gb|NP_490501)	Rck (VF0108)	Confers serum resistance by binding human complement factor H.; Rck mediates cell invasion through a Zipper-like mechanism, as described for other bacteria such as Listeria and Yersinia. Salmonella seems to be the first bacterium found to be able to induce both Zipper-like mechanism and the Trigger mechanism mediated by its T3SS-1 apparatus.	setA	Salmonella enterica subsp. enterica serovar Typhimurium str. LT2	Immune modulation	rck	resistance to complement killing	37.3	7.10E-31	111
ECs_2942	ECs_2942	outer membrane precursor Lom	VFG000442 (gb|NP_490501)	Rck (VF0108)	Confers serum resistance by binding human complement factor H.; Rck mediates cell invasion through a Zipper-like mechanism, as described	setA	Salmonella enterica subsp. enterica	Immune modulation	rck	resistance to complement killing	38.5	1.61E-32	115

					for other bacteria such as Listeria and Yersinia. Salmonella seems to be the first bacterium found to be able to induce both Zipper-like mechanism and the Trigger mechanism mediated by its T3SS-1 apparatus.		serovar Typhimurium str. LT2					
ECs_2160	ECs_2160	outer membrane precursor Lom	VFG000442 (gb|NP_490501)	Rck (VF0108)	Confers serum resistance by binding human complement factor H.; Rck mediates cell invasion through a Zipper-like mechanism, as described for other bacteria such as Listeria and Yersinia. Salmonella seems to be the first bacterium found to be able to induce both Zipper-like mechanism and the Trigger mechanism mediated by its T3SS-1 apparatus.	setA	Salmonella enterica subsp. enterica serovar Typhimurium str. LT2	Immune modulation	rck	resistance to complement killing	37	2.53E-31	112
ECs_2232	ECs_2232	outer membrane precursor Lom	VFG000442 (gb|NP_490501)	Rck (VF0108)	Confers serum resistance by binding human complement factor H.; Rck mediates cell invasion through a Zipper-like mechanism, as described for other bacteria such as Listeria and Yersinia. Salmonella seems to be the first bacterium found to be able to induce both Zipper-like mechanism and the Trigger mechanism mediated by its T3SS-1 apparatus.	setA	Salmonella enterica subsp. enterica serovar Typhimurium str. LT2	Immune modulation	rck	resistance to complement killing	37	2.53E-31	112

ECs_1236	ECs_1236	outer membrane precursor protein Lom	VFG000442 (gb|NP_490501)	Rck (VF0108)	Confers serum resistance by binding human complement factor H.; Rck mediates cell invasion through a Zipper-like mechanism, as described for other bacteria such as Listeria and Yersinia. Salmonella seems to be the first bacterium found to be able to induce both Zipper-like mechanism and the Trigger mechanism mediated by its T3SS-1 apparatus.	setA	Salmonella enterica subsp. enterica serovar Typhimurium str. LT2	Immune modulation	rck	resistance to complement killing	35.6	6.00E-28	105
ECs_0892	ompX	outer membrane protein OmpX	VFG003379 (gb|WP_011192817)	Ail (VF0132)	Promoting attachment to and subsequent invasion of eukaryotic cells, but a less powerful adhesion than Inv; promotes Yop delivery into the primary target of T3SS--the phagocytic cells, as well as into epithelial cells; promotes resistance to complement killing	setA	Yersinia pseudotuberculosis IP 32953	Invasion	ail	attachment invasion locus protein	43	6.70E-35	120
ECs_0773	tolR	colicin transporter TolR	VFG018806 (gb|WP_002216614)	Ton system (VF0895)	-	setB	Neisseria meningitidis 053442	Nutritional/Metabolic factor	exbD	biopolymer transporter ExbD	33.3	2.64E-17	73.2
ECs_3889	exbD	biopolymer transport protein ExbD	VFG018806 (gb|WP_002216614)	Ton system (VF0895)	-	setB	Neisseria meningitidis 053442	Nutritional/Metabolic factor	exbD	biopolymer transporter ExbD	35.5	4.74E-21	82.8

ECs_5124	groL	molecular chaperone GroEL	VFG010484 (gb|WP_197535716)	Hsp60 (VF0159)	Mediate a complement-independent attachment to mammalian and amoebal host cells	setA	Legionella pneumophila str. Paris	Adherence	htpB	Hsp60, 60K heat shock protein HtpB	75	4.90E-272	751
ECs_1370	ECs_1370	glucosyl-transferase	VFG048549 (gb|WP_004890934)	Sal (VF0563)	A major trigger of inflammation and bacterial dissemination induced during K. pneuminiae lung infection	setA	Klebsiella pneumoniae subsp. pneumoniae 1084	Nutritional/Metabolic factor	iroB	glucosyltransferase IroB	85.5	3.26E-230	631
ECs_5331	rimI	ribosomal-protein-S18-alanine N-acetyltransferase	VFG039386 (gb|NP_819821)	T4SS secreted effectors (VF0696)	CBUA0020; CBU_0012*; CBU_0113; CBU_0122; CBU_0183; CBU_0201; CBU_0270; CBU_0295; CBU_0344*; CBU_0372; CBU_0375*; CBU_0469; CBU_0513; CBU_0534; CBU_0590; CBU_0635; CBU_0637; CBU_0820*; CBU_1048*; CBU_1079; CBU_1107*; CBU_1150*; CBU_1198; CBU_1268; CBU_1349; CBU_1370; CBU_1409; CBU_1434; CBU_1493; CBU_1495*; CBU_1525*; CBU_1530; CBU_1566; CBU_1576; CBU_1594; CBU_1607; CBU_1614; CBU_1639; CBU_1665; CBU_1677; CBU_1685; CBU_1752; CBU_1754; CBU_1789; CBU_1790; CBU_1794; CBU_1818; CBU_1819; CBU_1863; CBU_2016; CBU_2028;	setA	Coxiella burnetii RSA 493	Effector delivery system	coxH2/rimL	Coxiella Dot/Icm type IVB secretion system translocated effector	37.3	1.92E-25	94.4

					CBU_2056; CBU_2059*; CBU_2076; AnkA; AnkB; AnkF; AnkG (Interacts with host protein p32 to block apoptosis.); AnkH; AnkI; AnkM/cig58; AnkP; Cem1; Cem12; Cem13; Cem3; Cem4; Cem6; Cem9; CetCb1; CetCb2; CetCb3; CetCB4; CetCb5; CetCb6; CirA/coxCC1 (Phosphate transporter family protein.); CirB; CirC/coxDFB1; CoxCC10/cig49; CoxCC11; CoxCC12; CoxCC14; CoxCC15; CoxCC3; CoxCC4; CoxCC5; CoxCC6; CoxCC7/cig44; CoxCC8; CoxDFB3; CoxDFB4 (Surface antigen.); CoxDFB5/cig57; CoxDFB6; CoxFIC1; CoxH2/rimL (Acetyltransferase.); CoxH3; CoxH4/cig61; CoxK1 (Protein kinase, putative.); CoxK2; CoxTPR1 (Conserved domain protein.); CoxU1; CoxU2; CpeA; CpeB; CpeC/coxU3 (Hypothetical protein plasmid QpH1.); CpeD; CpeE; CpeF; CpeG; CpeH; CvpA; MceA; PhnB; CBUD_RS05145; CBUD_RS06720*; CBUD_RS08635; CBUD_RS11275; CBUD_RS12405; CBUG_RS02435; CBUK_RS06760								
ECs_4435	yiaC	acetyltra	VFG039386	T4SS	CBUA0020; CBU_0012*; CBU_0113;	setA	Coxiella	Effector	coxH2/rimL	Coxiella	32.6	2.66E-	44.3

nsferase	(gb|NP_819821)	secreted effectors (VF0696)	CBU_0122; CBU_0183; CBU_0201; CBU_0270; CBU_0295; CBU_0344*; CBU_0372; CBU_0375*; CBU_0469; CBU_0513; CBU_0534; CBU_0590; CBU_0635; CBU_0637; CBU_0820*; CBU_1048*; CBU_1079; CBU_1107*; CBU_1150*; CBU_1198; CBU_1268; CBU_1349; CBU_1370; CBU_1409; CBU_1434; CBU_1493; CBU_1495*; CBU_1525*; CBU_1530; CBU_1566; CBU_1576; CBU_1594; CBU_1607; CBU_1614; CBU_1639; CBU_1665; CBU_1677; CBU_1685; CBU_1752; CBU_1754; CBU_1789; CBU_1790; CBU_1794; CBU_1818; CBU_1819; CBU_1863; CBU_2016; CBU_2028; CBU_2056; CBU_2059*; CBU_2076; AnkA; AnkB; AnkF; AnkG (Interacts with host protein p32 to block apoptosis.); AnkH; AnkI; AnkM/cig58; AnkP; Cem1; Cem12; Cem13; Cem3; Cem4; Cem6; Cem9; CetCb1; CetCb2; CetCb3; CetCB4; CetCb5; CetCb6; CirA/coxCC1 (Phosphate transporter family protein.); CirB; CirC/coxDFB1; CoxCC10/cig49; CoxCC11; CoxCC12;	burnetii RSA 493	delivery system	Dot/Icm type IVB secretion system translocated effector	06

ECs_4274	glgA	glycogen synthase	VFG050270 (gb|NP_220318)	TTSS secreted effectors (VF0711)	CoxCC14; CoxCC15; CoxCC3; CoxCC4; CoxCC5; CoxCC6; CoxCC7/cig44; CoxCC8; CoxDFB3; CoxDFB4 (Surface antigen.); CoxDFB5/cig57; CoxDFB6; CoxFIC1; CoxH2/rimL (Acetyltransferase.); CoxH3; CoxH4/cig61; CoxK1 (Protein kinase, putative.); CoxK2; CoxTPR1 (Conserved domain protein.); CoxU1; CoxU2; CpeA; CpeB; CpeC/coxU3 (Hypothetical protein plasmid QpH1.); CpeD; CpeE; CpeF; CpeG; CpeH; CvpA; MceA; PhnB; CBUD_RS05145; CBUD_RS06720*; CBUD_RS08635; CBUD_RS11275; CBUD_RS12405; CBUG_RS02435; CBUK_RS06760 CadD (Iron-containing redox enzyme. Contains death domain motif and is capable of interacting with TNF family receptors.); CdsZ; CopN (Contribute to manipulation of microtubule networks, delays host cell division and supports the intracellular growth of Chlamydia.); CpoS (May play a role in regulating the intracellular trafficking or fusogenicity of the chlamydial inclusion.); CteG (Might	setA	Chlamydia trachomatis D/UW-3/CX	Effector delivery system	glgA	glycogen synthase	30.8	3.83E-58	199

function by subverting host cell vesicular transport.); FilF; InaC (Recruits and activates host ADP-ribosylation factor 1 (ARF1) and ARF4 to regulate microtubules.); IncA (SNARE mimicry. Associated with homotypic fusion of inclusions and SNARE recruitment in C. trachomatis.); IncB; IncC; IncD (Formation of ER-inclusion MCS, non-vesicular lipid acquisition.); IncE (Manipulates retromer-mediated transport.); IncG (Early-phase effector.); IncV (Formation of ER-inclusion membrane contact sites (MCS).); IPAM (Hijacks microtubule organizing functions and controls microtubule assembly.); Mcsc; MrcA (Promotes Chlamydia extrustion.); NUE (Histone methyltransferase.); Pkn5 (Ser/Thr kinase.); Tarp (Actin-binding protein. Recuitment and nucleation of actin to facillitate entry of Ebs into host cells; Tarp can be phosphorylated on tyrosine residues and is proposed to function similarly

					to EPEC Tir.); TepP (Regulates innate immune signaling early in infection.); TmeA (Host cell invasion.); CrpA; GlgA; GlgX; YycJ; CT_006; CT_053; CT_061; CT_082; CT_083; CT_117; CT_134; CT_135; CT_142; CT_143; CT_144; CT_147; CT_156; CT_161; CT_163; CT_179; CT_192; CT_203; CT_222; CT_224; CT_225; CT_226; CT_227; CT_228 (Inhibits chlamydial extrusion.); CT_249; CT_288; CT_345; CT_358; CT_383; CT_429; CT_440; CT_449; CT_473; CT_483; CT_529; CT_550; CT_565; CT_577; CT_606.1; CT_618; CT_619; CT_620; CT_621 (DUF582 domain protein, function unknown.); CT_622; CT_652.1; CT_656; CT_668; CT_695; CT_711; CT_712; CT_718; CT_847 (Interacts with human Grap2 Cyclin D-interacting protein (GCIP), and may contribute to observed degradation of GCIP during chlamydial infection.); CT_848; CT_849; CT_850; CT_863								
ECs_4578	grlR	negative regulator	VFG000822 (gb|WP_00	TTSS (VF0191)	Injects Tir and other effector molecules directly into the host cell.	setA	Escherichia coli	Effector delivery	glrR	type III secretion	100	1.77E-88	252

		GrlR	0605370)		Effector molecules activate cell-signaling pathways, causing alterations in the host cell cytoskeleton and resulting in the depolymerization of actin and the loss of microvilli		O157:H7 str. EDL933	system		system LEE GrlA-binding negative regulator GrlR			
ECs_1743	oppA	oligopeptide ABC transporter periplasmic binding protein	VFG032255 (gb|WP_003753364)	Oligopeptide-binding protein oppA (VF0786)	-	setB	Listeria seeligeri serovar 1/2b str. SLCC3954	Nutritional/Metabolic factor	oppA	peptide ABC transporter substrate-binding protein	30.7	9.45E-64	218
ECs_1911	mppA	murein tripeptide (L-ala-gamma-D-glutamyl-meso-DAP) transporter subunit	VFG032255 (gb|WP_003753364)	Oligopeptide-binding protein oppA (VF0786)	-	setB	Listeria seeligeri serovar 1/2b str. SLCC3954	Nutritional/Metabolic factor	oppA	peptide ABC transporter substrate-binding protein	31.2	8.54E-60	207
ECs_0909	gsiB	glutathione ABC	VFG019366 (gb|WP_00	Oligopeptide-bindi	-	setB	Listeria monocytoge	Nutritional/Metaboli	oppA	peptide ABC transporte	24.7	2.33E-14	75.5

		transporter periplasmic binding protein	3729598)	ng protein oppA (VF0786)			nes HCC23	c factor		r substrate-binding protein			
ECs_3904	ygiS	ABC transporter substrate-binding protein	VFG019366 (gb|WP_003729598)	Oligopeptide-binding protein oppA (VF0786)	-	setB	Listeria monocytogenes HCC23	Nutritional/Metabolic factor	oppA	peptide ABC transporter substrate-binding protein	30.4	7.08E-51	182
ECs_2091	ddpA	D%2CD-dipeptide ABC transporter periplasmic binding protein	VFG019366 (gb|WP_003729598)	Oligopeptide-binding protein oppA (VF0786)	-	setB	Listeria monocytogenes HCC23	Nutritional/Metabolic factor	oppA	peptide ABC transporter substrate-binding protein	23.5	8.70E-25	107
ECs_4343	nikA	nickel/heme ABC transporter periplasm	VFG006782 (gb|NP_465720)	Oligopeptide-binding protein oppA	-	setB	Listeria monocytogenes EGD-e	Nutritional/Metabolic factor	oppA	hypothetical protein	23.2	2.73E-11	65.9

		ic binding protein		(VF0786)									
ECs_0515	acrB_1	multidrug efflux system protein	VFG049144 (gb|WP_002892069)	AcrAB (VF0568)	May mediate resistance against host-derived antimicrobial peptides; associated with antibiotic resistance	setA	Klebsiella pneumoniae subsp. pneumoniae NTUH-K2044	Antimicrobial activity/Competitive advantage	acrB	acriflavine resistance protein B	91.3	0	1831
ECs_3332	acrB	multidrug efflux system protein	VFG049144 (gb|WP_002892069)	AcrAB (VF0568)	May mediate resistance against host-derived antimicrobial peptides; associated with antibiotic resistance	setA	Klebsiella pneumoniae subsp. pneumoniae NTUH-K2044	Antimicrobial activity/Competitive advantage	acrB	acriflavine resistance protein B	65	0	1291
ECs_4394	acrB_5	multidrug efflux system protein	VFG049144 (gb|WP_002892069)	AcrAB (VF0568)	May mediate resistance against host-derived antimicrobial peptides; associated with antibiotic resistance	setA	Klebsiella pneumoniae subsp. pneumoniae NTUH-K2044	Antimicrobial activity/Competitive advantage	acrB	acriflavine resistance protein B	70.8	0	1417
ECs_1864	acrB_2	multidrug efflux system protein	VFG049144 (gb|WP_002892069)	AcrAB (VF0568)	May mediate resistance against host-derived antimicrobial peptides; associated with antibiotic resistance	setA	Klebsiella pneumoniae subsp. pneumoniae NTUH-K2044	Antimicrobial activity/Competitive advantage	acrB	acriflavine resistance protein B	56.6	0	1036
ECs_0613	cusA	copper/silver efflux	VFG036998 (gb|WP_014573843)	MtrCDE (VF0451)	A tripartite multidrug efflux pump essential for resistance to <beta>-lactams (penicillin G and	setA	Neisseria meningitidis 8013	Antimicrobial activity/C	mtrD	multiple transferable	22.3	3.94E-56	211

		system			nafcillin), macrolides (erythromycin) and host-derived compounds (peptide LL-37) and progesterone, and essential for growth of gonococci in the lower genital tract of experimentally infected female mice			ompetitive advantage		resistance system protein MtrD			
ECs_0516	acrA	multidrug efflux system	VFG049125 (gb|WP_004136054)	AcrAB (VF0568)	May mediate resistance against host-derived antimicrobial peptides; associated with antibiotic resistance	setA	Klebsiella oxytoca E718	Antimicrobial activity/Competitive advantage	acrA	acriflavine resistance protein A	85.9	6.52E-238	652
ECs_4137	acrE	multidrug transporter	VFG049125 (gb|WP_004136054)	AcrAB (VF0568)	May mediate resistance against host-derived antimicrobial peptides; associated with antibiotic resistance	setA	Klebsiella oxytoca E718	Antimicrobial activity/Competitive advantage	acrA	acriflavine resistance protein A	67.7	2.08E-177	499
ECs_4393	mdtE	anaerobic multidrug efflux transporter	VFG049125 (gb|WP_004136054)	AcrAB (VF0568)	May mediate resistance against host-derived antimicrobial peptides; associated with antibiotic resistance	setA	Klebsiella oxytoca E718	Antimicrobial activity/Competitive advantage	acrA	acriflavine resistance protein A	54.1	3.40E-132	384
ECs_1863	eefA	multidrug-efflux transport protein precursor	VFG049125 (gb|WP_004136054)	AcrAB (VF0568)	May mediate resistance against host-derived antimicrobial peptides; associated with antibiotic resistance	setA	Klebsiella oxytoca E718	Antimicrobial activity/Competitive advantage	acrA	acriflavine resistance protein A	52.1	2.76E-107	320

ECs_0395	lacA	galactoside O-acetyltransferase	VFG001306 (gb|WP_000636149)	Capsule (VF0003)	Prevent phagocytosis	setA	Staphylococcus aureus subsp. aureus MW2	Immune modulation	cap8J	type 8 capsular polysaccharide synthesis protein Cap8J	37.6	1.69E-14	68.6
ECs_2859	wcaF	acyl transferase	VFG001306 (gb|WP_000636149)	Capsule (VF0003)	Prevent phagocytosis	setA	Staphylococcus aureus subsp. aureus MW2	Immune modulation	cap8J	type 8 capsular polysaccharide synthesis protein Cap8J	26.8	2.74E-09	53.9
ECs_4055	glmM	phosphoglucosamine mutase	VFG013514 (gb|WP_011272587)	Exopolysaccharide (VF0755)	-	setA	Haemophilus influenzae 86-028NP	Immune modulation	mrsA/glmM	phosphoglucosamine mutase	73.3	3.38E-234	647
ECs_1571	potA	spermidine/putrescine ABC transporter ATPase	VFG030696 (gb|WP_014711073)	Trehalose-recycling ABC transporter (VF0842)	-	setA	Mycobacterium intracellulare str. MOTT36Y	Nutritional/Metabolic factor	sugC	sn-glycerol-3-phosphate ABC transporter ATP-binding protein UgpC	53.2	2.50E-76	241

ECs_0413	afuC	ferric transporter subunit	VFG030696 (gb|WP_014711073)	Trehalose-recycling ABC transporter (VF0842)	-	setA	Mycobacterium intracellulare str. MOTT36Y	Nutritional/Metabolic factor	sugC	sn-glycerol-3-phosphate ABC transporter ATP-binding protein UgpC	43.8	1.68E-74	235
ECs_4144	yhdZ	amino acid ABC transporter ATPase	VFG030696 (gb|WP_014711073)	Trehalose-recycling ABC transporter (VF0842)	-	setA	Mycobacterium intracellulare str. MOTT36Y	Nutritional/Metabolic factor	sugC	sn-glycerol-3-phosphate ABC transporter ATP-binding protein UgpC	34.7	1.12E-39	141
ECs_1867	sapF	antimicrobial peptide ABC transporter ATPase	VFG030696 (gb|WP_014711073)	Trehalose-recycling ABC transporter (VF0842)	-	setA	Mycobacterium intracellulare str. MOTT36Y	Nutritional/Metabolic factor	sugC	sn-glycerol-3-phosphate ABC transporter ATP-binding protein UgpC	31.6	1.44E-27	109
ECs_4579	ipgF	hypothetical	VFG000823 (gb|WP_00	TTSS (VF0191)	Injects Tir and other effector molecules directly into the host cell.	setA	Escherichia coli	Effector delivery	etgA	T3SS-associated	100	1.19E-109	308

		protein	0720639)		Effector molecules activate cell-signaling pathways, causing alterations in the host cell cytoskeleton and resulting in the depolymerization of actin and the loss of microvilli		O157:H7 str. EDL933	system		peptidoglycan lytic enzyme			
ECs_0734	dtpD	dipeptide and tripeptide permease D	VFG045725 (gb|WP_003636639)	IraAB (VF0293)	IraA, a methyltransferase, is critical for virulence; IraB, a homologue of di- and tri-peptide transporters, may involve in a novel method of iron acquisition which may utilize iron-loaded peptides	setA	Legionella longbeachae NSW150	Nutritional/Metabolic factor	iraB	Di/tripeptide permease	20.9	6.19E-16	80.1
ECs_5112	dtpD	dipeptide and tripeptide permease D	VFG045725 (gb|WP_003636639)	IraAB (VF0293)	IraA, a methyltransferase, is critical for virulence; IraB, a homologue of di- and tri-peptide transporters, may involve in a novel method of iron acquisition which may utilize iron-loaded peptides	setA	Legionella longbeachae NSW150	Nutritional/Metabolic factor	iraB	Di/tripeptide permease	19.6	4.23E-14	74.3
ECs_4368	dtpB	dipeptide and tripeptide permease B	VFG045725 (gb|WP_003636639)	IraAB (VF0293)	IraA, a methyltransferase, is critical for virulence; IraB, a homologue of di- and tri-peptide transporters, may involve in a novel method of iron acquisition which may utilize iron-loaded peptides	setA	Legionella longbeachae NSW150	Nutritional/Metabolic factor	iraB	Di/tripeptide permease	25.2	2.59E-51	181
ECs_2343	dtpA	dipeptide and	VFG045725 (gb|WP_00	IraAB (VF0293)	IraA, a methyltransferase, is critical for virulence; IraB, a homologue of di-	setA	Legionella longbeacha	Nutritional/Metaboli	iraB	Di/tripeptide	28.6	3.06E-58	200

		tripeptide permease A	3636639)		and tri-peptide transporters, may involve in a novel method of iron acquisition which may utilize iron-loaded peptides		e NSW150	c factor		permease			
ECs_1602	phoP	two-component regulatory system response regulator PhoP	VFG004063 (gb|WP_001537766)	PhoPQ (VF0111)	Activated by low divalent cations magnesium and calcium levels; control expression of more than 40 genes; required for intracellular survival, cationic antimicrobial peptides (CAMPs) resistance, stimulation of cytokine secretion	setA	Salmonella enterica subsp. enterica serovar Paratyphi A str. ATCC 9150	Regulation	phoP	two-component system response regulator PhoP	93.3	2.17E-149	414
ECs_1601	phoQ	two-component system sensor histidine kinase PhoQ	VFG021077 (gb|WP_001031685)	PhoPQ (VF0111)	Activated by low divalent cations magnesium and calcium levels; control expression of more than 40 genes; required for intracellular survival, cationic antimicrobial peptides (CAMPs) resistance, stimulation of cytokine secretion	setA	Salmonella enterica subsp. enterica serovar Agona str. SL483	Regulation	phoQ	two-component system sensor histidine kinase PhoQ	85.4	7.25E-301	819
ECs_0228	impH	type VI secretion system protein ImpH	VFG035686 (gb|WP_000348794)	EHS (VF1176)	-	setA	Escherichia coli O157:H7 str. EDL933	Effector delivery system	aec22	type VI secretion system baseplate subunit TssG	100	1.24E-269	729
ECs_0229	ECs_0229	type VI	VFG035710	EHS	-	setA	Escherichi	Effector	tssF	type VI	100	0	1256

		secretion protein	(gb|WP_000393844)	(VF1176)			a coli O157:H7 str. EDL933	delivery system		secretion system baseplate subunit TssF			
ECs_4318	zntA	Cd2+/Zn2+-exporting ATPase	VFG031407 (gb|WP_015289456)	Copper exporter (VF0849)	-	setA	Mycobacterium canettii CIPT 140070010	Nutritional/Metabolic factor	ctpV	copper-translocating P-type ATPase	37.1	5.32E-100	325
ECs_0537	copA	copper transporter	VFG031407 (gb|WP_015289456)	Copper exporter (VF0849)	-	setA	Mycobacterium canettii CIPT 140070010	Nutritional/Metabolic factor	ctpV	copper-translocating P-type ATPase	44.9	9.92E-151	461
ECs_0725	kdpB	potassium translocating ATPase subunit B	VFG031392 (gb|WP_003404941)	Copper exporter (VF0849)	-	setA	Mycobacterium bovis AF2122/97	Nutritional/Metabolic factor	ctpV	copper-translocating P-type ATPase	32	1.45E-52	194
ECs_4232	aroK	shikimate kinase I	VFG042129 (gb|WP_157866201)	T3SS (VF1274)	-	setB	Mesorhizobium loti MAFF303099	Effector delivery system	MAFF_RS25805	hypothetical protein	36.3	1.75E-19	86.7
ECs_0438	aroL	shikimate kinase II	VFG042129 (gb|WP_157866201)	T3SS (VF1274)	-	setB	Mesorhizobium loti MAFF303099	Effector delivery system	MAFF_RS25805	hypothetical protein	31.5	1.37E-11	62

ECs_0423	hemB	5-aminolevulinate dehydratase	VFG013197 (gb|WP_011608705)	Heme biosynthesis (VF0758)	-	setB	Haemophilus somnus 2336	Nutritional/Metabolic factor	hemB	porphobilinogen synthase	42.5	1.72E-72	227
ECs_0424	yaiT	flagellin structural protein	VFG035991 (gb|NP_308451)	EhaB, AIDA-I type (VF1128)	-	setA	Escherichia coli O157:H7 str. Sakai	Adherence	ehaB	flagellin structural protein	100	0	1889
ECs_0426	ampH	D-alanyl-D-alanine-carboxypeptidase/endopeptidase	VFG043679 (gb|WP_002430641)	Colibactin (VF1179)	-	setA	Escherichia coli O18:K1:H7 str. IHE3034	Exotoxin	clbP	precolibactin peptidase ClbP	27.7	3.80E-10	61.2
ECs_5131	ampC	beta-lactamase	VFG043679 (gb|WP_002430641)	Colibactin (VF1179)	-	setA	Escherichia coli O18:K1:H7 str. IHE3034	Exotoxin	clbP	precolibactin peptidase ClbP	24.9	6.07E-22	97.1
ECs_3301	yfeW	penicillin binding protein PBP4B	VFG043679 (gb|WP_002430641)	Colibactin (VF1179)	-	setA	Escherichia coli O18:K1:H7 str. IHE3034	Exotoxin	clbP	precolibactin peptidase ClbP	29.5	2.30E-12	68.6
ECs_3331	narQ	sensory histidine	VFG009913 (gb|WP_01	DevRS (VF0317)	Controls the global response to oxidative stress and low oxygen	setA	Mycobacterium sp. JLS	Regulation	devS	GAF domain-con	32.7	9.65E-15	77

		kinase in two-component regulatory system with NarP	1856699)		repsonse, key regulator in the oxygen starvation-induced mycobacterial dormancy response					taining sensor histidine kinase			
ECs_1727	narX	two-component system sensor histidine kinase NarX	VFG041502 (gb|WP_004155353)	T3SS (VF1270)	-	setA	Erwinia amylovora CFBP1430	Effector delivery system	EAMY_RS19925	PAS domain-containing sensor histidine kinase	29.9	3.33E-21	97.1
ECs_0417	ECs_0417	sensor histidine protein kinase	VFG041502 (gb|WP_004155353)	T3SS (VF1270)	-	setB	Erwinia amylovora CFBP1430	Effector delivery system	EAMY_RS19925	PAS domain-containing sensor histidine kinase	31.1	1.66E-19	91.3
ECs_4605	uhpB	two-component system sensor histidine kinase UhpB	VFG041502 (gb|WP_004155353)	T3SS (VF1270)	-	setA	Erwinia amylovora CFBP1430	Effector delivery system	EAMY_RS19925	PAS domain-containing sensor histidine kinase	30.8	1.51E-16	82

ECs_0644	ahpC	alkyl hydroperoxide reductase	VFG009680 (gb|WP_011730169)	AhpC (VF0306)	Alkyl hydroperoxide reductase involved in protecting mycobacteria from the oxidative and nitrosative responses of macrophages	setA	Mycobacterium smegmatis str. MC2 155	Stress survival	ahpC	peroxiredoxin	36.5	2.89E-35	122
ECs_4494	tdh	L-threonine 3-dehydrogenase	VFG030297 (gb|YP_001702819)	MymA operon (VF0840)	-	setA	Mycobacterium abscessus ATCC 19977	Immune modulation	adhD	Probable zinc-type alcohol dehydrogenase AdhD	25.1	1.46E-18	85.5
ECs_4525	spoT	bifunctional (p)ppGpp synthase/ hydrolase SpoT	VFG009933 (gb|WP_085975473)	RelA (VF0287)	Associated with stationary phase adaptation and long-term survival	setA	Mycobacterium sp. MCS	Regulation	relA	Probable GTP pyrophosphokinase RelA (ATP:GTP 3’-pyrophosphotransferase) (PPGPP synthetase I) ((P)PPGPP synthetase) (GTP diphosphok	40.1	2.13E-164	492

										inase)			
ECs_4021	yraI	periplasmic pilin chaperone	VFG000455 (gb|NP_462540)	Lpf (VF0105)	Mediate attachment to the Peyer's patches	setA	Salmonella enterica subsp. enterica serovar Typhimurium str. LT2	Adherence	lpfB	long polar fimbrial chaperone protein LpfB	42.6	1.32E-49	161
ECs_4430	lpfB	fimbrial chaperone protein precursor	VFG000455 (gb|NP_462540)	Lpf (VF0105)	Mediate attachment to the Peyer's patches	setA	Salmonella enterica subsp. enterica serovar Typhimurium str. LT2	Adherence	lpfB	long polar fimbrial chaperone protein LpfB	70.4	2.93E-109	313
ECs_4076	kdsD	D-arabinose 5-phosphate isomerase	VFG011729 (gb|WP_002869182)	Capsule (VF0323)	Play an important role in bacterial survival and persistence in the environment and evasion of host immune response; the presence of heptose residues in the capsule may be important for virulence. Heptose residues found in some cell surface-located glycoconjugates are required for adhesion	setA	Campylobacter jejuni subsp. jejuni 81-176	Immune modulation	kpsF	KpsF/GutQ family sugar-phosphate isomerase	46.1	2.31E-87	265
ECs_2112	fimC	fimbrial chaperone	VFG042714 (gb|WP_00	F9 fimbriae	-	setA	Escherichia coli	Adherence	Z_RS10335	fimbria/pilus	100	3.64E-168	462

		protein precursor	1195169)	(VF1153)			O157:H7 str. EDL933			periplasmic chaperone			
ECs_2113	fmlA	type 1 fimbrial protein precursor	VFG042713 (gb|WP_001296758)	F9 fimbriae (VF1153)	-	setA	Escherichia coli O157:H7 str. EDL933	Adherence	fimA	type 1 fimbrial major subunit FimA	100	1.11E-122	343
ECs_5273	fimA	major type 1 subunit fimbrin	VFG012278 (gb|WP_000695554)	Type 1 fimbriae (VF0221)	Makes an important contribution to colonization of the bladder	setA	Escherichia coli O157:H7 str. EDL933	Adherence	fimA	Type-1 fimbrial protein, A chain precursor	99.5	1.58E-115	325
ECs_2110	fimD	outer membrane usher protein	VFG042715 (gb|WP_000123651)	F9 fimbriae (VF1153)	-	setA	Escherichia coli O157:H7 str. EDL933	Adherence	Z_RS10340	fimbrial biogenesis outer membrane usher protein	99.9	0	1731
ECs_3420	glrR	two-component regulatory system response regulator glrR	VFG043345 (gb|WP_011946713)	Flagella (VF0157)	Not required for intracellular growth but enhance the invasion capacity	setA	Legionella pneumophila subsp. pneumophila str. Philadelphia 1	Motility	fleR/flrC	sigma 54-dependent response regulator	42.5	9.29E-110	330
ECs_0659	citB	response	VFG013993	Type IV	Attaches to host cells, but not to	setA	Pseudomona	Adherence	pilR	two-compon	31.1	1.21E-	57.4

		regulator in two-component regulatory system with CitA	(gb|WP_026006503)	pili (VF0082)	mucin, causing a twitching motility that allows the bacteria to move along the cell surface; biofilm formation		s stutzeri A1501			ent response regulator PilR		09	
ECs_4790	glnG	nitrogen regulation protein NR(I)	VFG013993 (gb|WP_026006503)	Type IV pili (VF0082)	Attaches to host cells, but not to mucin, causing a twitching motility that allows the bacteria to move along the cell surface; biofilm formation	setA	Pseudomonas stutzeri A1501	Adherence	pilR	two-component response regulator PilR	39.3	4.82E-101	309
ECs_4927	zraR	transcriptional regulatory protein ZraR	VFG013993 (gb|WP_026006503)	Type IV pili (VF0082)	Attaches to host cells, but not to mucin, causing a twitching motility that allows the bacteria to move along the cell surface; biofilm formation	setA	Pseudomonas stutzeri A1501	Adherence	pilR	two-component response regulator PilR	43.9	1.72E-108	327
ECs_3742	ygeV	sigma-54-interacting transcriptional activator	VFG019760 (gb|WP_012722471)	Type IV pili (VF0082)	Attaches to host cells, but not to mucin, causing a twitching motility that allows the bacteria to move along the cell surface; biofilm formation	setA	Pseudomonas fluorescens SBW25	Adherence	pilR	two-component response regulator PilR	43.4	7.08E-77	251
ECs_3587	fhlA	formate hydrogenlyase	VFG019760 (gb|WP_012722471)	Type IV pili (VF0082)	Attaches to host cells, but not to mucin, causing a twitching motility that allows the bacteria to move along	setA	Pseudomonas fluorescen	Adherence	pilR	two-component response	42.4	1.58E-85	276

		transcriptional activator			the cell surface; biofilm formation		s SBW25			regulator PilR			
ECs_3353	hyfR	transcriptional activator HyfR	VFG019760 (gb|WP_012722471)	Type IV pili (VF0082)	Attaches to host cells, but not to mucin, causing a twitching motility that allows the bacteria to move along the cell surface; biofilm formation	setA	Pseudomonas fluorescens SBW25	Adherence	pilR	two-component response regulator PilR	42.7	1.14E-82	268
ECs_1902	tyrR	aromatic amino acid biosynthesis and transport regulon transcriptional regulator	VFG013985 (gb|WP_012077306)	Type IV pili (VF0082)	Attaches to host cells, but not to mucin, causing a twitching motility that allows the bacteria to move along the cell surface; biofilm formation	setA	Pseudomonas aeruginosa PA7	Adherence	pilR	two-component response regulator PilR	35.3	1.73E-65	218
ECs_3427	yfhH	transcriptional regulator	VFG013181 (gb|WP_012340544)	LOS (VF0044)	Major immunogen; LOS phosphorylcholine (ChoP) may influence invasion via interaction with PAF receptor and stimulates of inflammatory signals; LPS phase variation is characterized by the spontaneous loss and gain of oligosaccharide structures present in	setA	Haemophilus somnus 2336	Immune modulation	kpsF	KpsF/GutQ family sugar isomerase	24.3	4.80E-08	53.1

					the outer core. the phase variable expression of LPS biosynthesis genes promotes evasion of antigen-specific host immune defences and allow colonization of different host microenvironments								
ECs_3564	srlQ	D-arabinose 5-phosphate isomerase	VFG013481 (gb|WP_041604195)	LOS (VF0044)	Major immunogen; LOS phosphorylcholine (ChoP) may influence invasion via interaction with PAF receptor and stimulates of inflammatory signals; LPS phase variation is characterized by the spontaneous loss and gain of oligosaccharide structures present in the outer core. the phase variable expression of LPS biosynthesis genes promotes evasion of antigen-specific host immune defences and allow colonization of different host microenvironments	setA	Haemophilus somnus 129PT	Immune modulation	kpsF	KpsF/GutQ family sugar isomerase	40.4	5.26E-72	225
ECs_4572	rorf8	T3SS component	VFG000816 (gb|WP_001302733)	TTSS (VF0191)	Injects Tir and other effector molecules directly into the host cell. Effector molecules activate cell-signaling pathways, causing alterations in the host cell cytoskeleton and resulting in the	setA	Escherichia coli O157:H7 str. EDL933	Effector delivery system	escI	Type III secretion system inner rod component EscI	100	8.37E-79	228

					depolymerization of actin and the loss of microvilli								
ECs_0705	umpH	UMP phosphatase	VFG015888 (gb|WP_003381691)	Phytotoxin phaseolotoxin (VF0917)	-	setB	Pseudomonas syringae pv. phaseolicola 1448A	Exotoxin	PSPPH_RS21770	HAD-IIA family hydrolase	28.3	9.37E-30	112
ECs_5241	ahr	alcohol dehydrogenase	VFG026091 (gb|WP_004194140)	Capsule I (VF0436)	A key virulence determinant and that loss of capsule production results in severe attenuation in animal models of disease	setA	Burkholderia mallei ATCC 23344	Immune modulation	wcbR	capsular polysaccharide biosynthesis fatty acid synthase	30.1	3.50E-06	48.9
ECs_1892	ycjQ	Zn-dependent NAD(P)-binding oxidoreductase	VFG026091 (gb|WP_004194140)	Capsule I (VF0436)	A key virulence determinant and that loss of capsule production results in severe attenuation in animal models of disease	setA	Burkholderia mallei ATCC 23344	Immune modulation	wcbR	capsular polysaccharide biosynthesis fatty acid synthase	26.8	1.47E-10	62.8
ECs_0704	asnB	asparagine synthetase B	VFG011198 (gb|WP_003807086)	LPS (VF0033)	Prevents clearance of the organism by host surfactant protein; confers protection to the bacterium from complement-mediated cell lysis	setA	Bordetella parapertussis 12822	Immune modulation	wbmI	asparagine synthase (glutamine-hydrolyzing)	32.4	2.90E-45	169

ECs_5137	yjeM	transporter	VFG047654 (gb|WP_003020284)	GadC (VF0555)	Glutamate permease GadC helps the bacterium to resist the oxidative stress generated by the NADPH oxidase. This transporter is critically required for proper bacterial phagosomal escape	setA	Francisella tularensis subsp. tularensis SCHU S4	Nutritional/Metabolic factor	gadC	glutamate:gamma-aminobutyric acid antiporter family protein	25.1	3.17E-09	58.9
ECs_5122	yjeH	transporter	VFG047654 (gb|WP_003020284)	GadC (VF0555)	Glutamate permease GadC helps the bacterium to resist the oxidative stress generated by the NADPH oxidase. This transporter is critically required for proper bacterial phagosomal escape	setA	Francisella tularensis subsp. tularensis SCHU S4	Nutritional/Metabolic factor	gadC	glutamate:gamma-aminobutyric acid antiporter family protein	27.3	2.72E-06	49.3
ECs_4221	frlA	fructoselysine transporter	VFG047654 (gb|WP_003020284)	GadC (VF0555)	Glutamate permease GadC helps the bacterium to resist the oxidative stress generated by the NADPH oxidase. This transporter is critically required for proper bacterial phagosomal escape	setA	Francisella tularensis subsp. tularensis SCHU S4	Nutritional/Metabolic factor	gadC	glutamate:gamma-aminobutyric acid antiporter family protein	22	2.43E-07	52.8
ECs_3960	ygjI	transporter	VFG047647 (gb|WP_003033343)	GadC (VF0555)	Glutamate permease GadC helps the bacterium to resist the oxidative stress generated by the NADPH oxidase. This transporter is critically required for proper bacterial	setA	Francisella novicida U112	Nutritional/Metabolic factor	gadC	glutamate:gamma-aminobutyric acid antiporter	22.8	8.77E-14	73.2

					phagosomal escape					family protein		
ECs_2597	cheW	purine-binding chemotaxis protein	VFG043093 (gb|WP_000147304)	Peritrichous flagella (VF1154)	-	setA	Escherichia coli O157:H7 str. EDL933	Motility	cheW	chemotaxis protein CheW	99.4	1.29E-109	309
ECs_4573	escJ	T3SS structure protein EscJ	VFG000817 (gb|WP_000716189)	TTSS (VF0191)	Injects Tir and other effector molecules directly into the host cell. Effector molecules activate cell-signaling pathways, causing alterations in the host cell cytoskeleton and resulting in the depolymerization of actin and the loss of microvilli	setA	Escherichia coli O157:H7 str. EDL933	Effector delivery system	escJ	Type III secretion system inner MS ring protein EscJ	100	4.52E-128	357
ECs_2592	cheY	chemotaxis regulator transmitting signal to flagellar motor component	VFG043089 (gb|WP_000763867)	Peritrichous flagella (VF1154)	-	setA	Escherichia coli O157:H7 str. EDL933	Motility	cheY	chemotaxis response regulator CheY	100	1.61E-87	250
ECs_2590	flhB	flagellin export apparatus	VFG043087 (gb|WP_001278933)	Peritrichous flagella	-	setA	Escherichia coli O157:H7	Motility	flhB	flagellar type III secretion	99.5	2.42E-269	731

		substrate specificity protein		(VF1154)					str. EDL933			system protein FlhB			
ECs_3698	galP	D-galactose transporter	VFG044370 (gb|AAQ21378)	Thioquinolobactin (VF0937)	-			setB	Pseudomonas fluorescens ATCC 17400	Nutritional/Metabolic factor	qbsN	QbsN	31.4	8.12E-17	82.4
ECs_2484	ydjK	MFS sugar transporter	VFG044370 (gb|AAQ21378)	Thioquinolobactin (VF0937)	-			setB	Pseudomonas fluorescens ATCC 17400	Nutritional/Metabolic factor	qbsN	QbsN	26.5	5.52E-13	70.5
ECs_3454	kgtP	alpha-ketoglutarate transporter	VFG044370 (gb|AAQ21378)	Thioquinolobactin (VF0937)	-			setB	Pseudomonas fluorescens ATCC 17400	Nutritional/Metabolic factor	qbsN	QbsN	27.1	6.86E-07	51.2
ECs_4097	nanT	sialic acid transporter	VFG044370 (gb|AAQ21378)	Thioquinolobactin (VF0937)	-			setB	Pseudomonas fluorescens ATCC 17400	Nutritional/Metabolic factor	qbsN	QbsN	23	6.82E-15	76.6
ECs_0408	mhpT	3-hydroxyphenylpro	VFG044370 (gb|AAQ21	Thioquinolobactin	-			setB	Pseudomonas	Nutritional/Metaboli	qbsN	QbsN	28.8	1.93E-26	110

		pionic transporter	378)	(VF0937)			fluorescens ATCC 17400	c factor					
ECs_3819	galP_2	D-galactose transporter	VFG044370 (gb|AAQ21378)	Thioquinolobactin (VF0937)	-	setB	Pseudomonas fluorescens ATCC 17400	Nutritional/Metabolic factor	qbsN	QbsN	23.9	1.36E-17	84.7
ECs_3031	ECs_3031	transport protein	VFG044370 (gb|AAQ21378)	Thioquinolobactin (VF0937)	-	setA	Pseudomonas fluorescens ATCC 17400	Nutritional/Metabolic factor	qbsN	QbsN	28.9	3.82E-40	149
ECs_0048	yaaU	MFS sugar transporter	VFG044370 (gb|AAQ21378)	Thioquinolobactin (VF0937)	-	setB	Pseudomonas fluorescens ATCC 17400	Nutritional/Metabolic factor	qbsN	QbsN	27.3	4.78E-23	100
ECs_4647	mdtL	multidrug efflux system protein	VFG044370 (gb|AAQ21378)	Thioquinolobactin (VF0937)	-	setA	Pseudomonas fluorescens ATCC 17400	Nutritional/Metabolic factor	qbsN	QbsN	24.6	3.11E-06	48.9
ECs_5014	xylE	D-xylose transporter	VFG044370 (gb|AAQ21378)	Thioquinolobactin (VF0937)	-	setB	Pseudomonas fluorescen	Nutritional/Metabolic factor	qbsN	QbsN	24	2.05E-13	72

ECs_2302	ynfM	arabinose efflux transporter	VFG044370 (gb|AAQ21378)	Thioquinolobactin (VF0937)	-	setA	s ATCC 17400 Pseudomonas fluorescens ATCC 17400	Nutritional/Metabolic factor	qbsN	QbsN	28.8	1.76E-10	62.4
ECs_2067	yddE	H repeat-associated protein	VFG015519 (gb|WP_012074246)	Pyocyanin (VF0100)	Toxic to bacterial and eukaryotic cells due to the reactive oxygen intermediates it generates, such as superoxide radical and hydrogen peroxide	setA	Pseudomonas aeruginosa PA7	Nutritional/Metabolic factor	phzF1	phenazine biosynthesis protein PhzF, isomerase	31.8	8.81E-26	103
ECs_2060	vgrE	type VI secretion system Vgr family protein	VFG035834 (gb|NP_310087)	ACE T6SS (VF1122)	-	setA	Escherichia coli O157:H7 str. Sakai	Effector delivery system	aec15	type VI secretion system Vgr family protein	100	0	1192
ECs_0236	vgrE	type VI secretion system Vgr family protein	VFG035834 (gb|NP_310087)	ACE T6SS (VF1122)	-	setA	Escherichia coli O157:H7 str. Sakai	Effector delivery system	aec15	type VI secretion system Vgr family protein	91.7	0	1083
ECs_0607	ECs_0607	type VI secretion	VFG035846 (gb|WP_00	ACE T6SS (VF1122)	-	setA	Escherichia coli	Effector delivery	aec15	type VI secretion	95.7	0	1024

		system Vgr family protein	0103201)				O104:H4 str. 2009EL-2050	system		system tip protein VgrG			
ECs_2068	narV	nitrate reductase 2 gamma subunit	VFG009635 (gb|WP_011893047)	Nitrate reductase (VF0302)	Nitrate respiration helps the bacteria to survive in O2-depleted areas of inflammatory or necrotic tissue	setA	Mycobacterium gilvum PYR-GCK	Nutritional/Metabolic factor	narI	respiratory nitrate reductase subunit gamma	36.7	1.99E-36	128
ECs_1732	narI	nitrate reductase 1 gamma subunit	VFG009635 (gb|WP_011893047)	Nitrate reductase (VF0302)	Nitrate respiration helps the bacteria to survive in O2-depleted areas of inflammatory or necrotic tissue	setA	Mycobacterium gilvum PYR-GCK	Nutritional/Metabolic factor	narI	respiratory nitrate reductase subunit gamma	32.3	1.08E-31	115
ECs_2069	narW	nitrate reductase 2 delta subunit	VFG009622 (gb|WP_011893046)	Nitrate reductase (VF0302)	Nitrate respiration helps the bacteria to survive in O2-depleted areas of inflammatory or necrotic tissue	setA	Mycobacterium gilvum PYR-GCK	Nutritional/Metabolic factor	narJ	nitrate reductase molybdenum cofactor assembly chaperone	25	6.44E-09	54.3
ECs_1731	narJ	molybdenum-cofactor-assembly chaperone delta	VFG009622 (gb|WP_011893046)	Nitrate reductase (VF0302)	Nitrate respiration helps the bacteria to survive in O2-depleted areas of inflammatory or necrotic tissue	setA	Mycobacterium gilvum PYR-GCK	Nutritional/Metabolic factor	narJ	nitrate reductase molybdenum cofactor assembly chaperone	29.9	1.95E-12	64.3

		subunit of nitrate reductase 1											
ECs_4072	mlaD	ABC-type organic solvent transporter	VFG023043 (gb|WP_012394582)	Mce3 (VF0826)	-	setB	Mycobacterium marinum M	Immune modulation	mce3B	virulence factor Mce family protein	34.3	3.40E-10	57.8
ECs_4228	rpe	D-ribulose-5-phosphate 3-epimerase	VFG046604 (gb|WP_012280804)	Capsule (VF0543)	Providing a stealth shield that prevents the host immune system from detecting this potent pathogen	setA	Francisella philomiragia subsp. philomiragia ATCC 25017	Immune modulation	rpe	ribulose-phosphate 3-epimerase	65.3	1.43E-99	288
ECs_3601	ispD	4-diphosphocytidyl-2C-methyl-D-erythritol synthase	VFG000688 (gb|AAP42181)	Capsule (VF0043)	Protecting the organism from phagocytosis and the bactericidal activity of complement	setA	Haemophilus influenzae str. 1007	Immune modulation	bcs1’	Bcs1’	27.2	3.35E-13	68.2
ECs_3604	cysC	adenosine 5’-phosphosulfate	VFG015885 (gb|WP_003381688)	Phytotoxin phaseolot	-	setA	Pseudomonas syringae pv.	Exotoxin	cysC1	adenylyl-sulfate kinase	53.8	1.00E-64	198

		kinase		oxin (VF0917)			phaseolicola 1448A						
ECs_3743	ygeW	carbamoyl transferase	VFG015903 (gb|WP_011169509)	Phytotoxin phaseolotoxin (VF0917)	-	setA	Pseudomonas syringae pv. phaseolicola 1448A	Exotoxin	argK	ornithine carbamoyltransferase	24.5	3.62E-14	72.8
ECs_5231	argI	ornithine carbamoyl transferase 1	VFG015903 (gb|WP_011169509)	Phytotoxin phaseolotoxin (VF0917)	-	setA	Pseudomonas syringae pv. phaseolicola 1448A	Exotoxin	argK	ornithine carbamoyltransferase	45	4.71E-86	262
ECs_5222	pyrB	aspartate carbamoyl transferase	VFG047700 (gb|WP_013921811)	Pyrimidine biosynthesis (VF0558)	Mediating bacterial resistance to reactive oxygen species (ROS) that is important for phagosomal escape	setA	Francisella sp. TX077308	Nutritional/Metabolic factor	pyrB	aspartate carbamoyltransferase	43.8	1.98E-68	215
gene-hlyD	hlyD	Hemolysin D	VFG000843 (gb|WP_001213545)	Hemolysin (VF0207)	Cytotoxic to many types of cells: erythrocytes, granulocytes, monocytes, endothelial cells and renal epithelial cells; stimulating the release of IL-1<beta> and TNF	setA	Escherichia coli O157:H7 str. EDL933	Exotoxin	hlyD	Hemolysin D	100	0	905
ECs_4561	tir	T3SS translocated intimin	VFG000805 (gb|WP_001301454)	TTSS secreted effectors (VF1110)	Cif (Deamidase. Induces cytopathic effects of actin stress fiber formation and cell cycle arrest.); EspB (Pore formation, actin	setA	Escherichia coli O157:H7 str. EDL933	Effector delivery system	tir	translocated intimin receptor protein	100	0	984

receptor Tir	disruption, microvilli effacement, anti-phagocytosis.); EspF (Inducing degradation of the aniapoptic protein AbcF2, tight junction disruption, microvilli effacement and elongation, mitochondrial dysfunction, N-WASP activation, SGLT-1 inactivation, pedestal maturation, inhibition of NHE3 activity, membrane remodelling; targets and disrupts the nucleolus late in infection, which is temporally controlled by host mitochondria.); EspFu/tccP (Inducing degradation of the aniapoptic protein AbcF2, tight junction disruption, microvilli effacement and elongation, mitochondrial dysfunction, N-WASP activation, SGLT-1 inactivation, pedestal maturation, inhibition of NHE3 activity, membrane remodelling; targets and disrupts the nucleolus late in infection, which is temporally controlled by host mitochondria.); EspG (TBC-like GTPase activating protein. Efficiently catalyzes GTP hydrolysis in Rab1 to disrupt of

Rab1-mediated ER-to-Golgi trafficking.); EspH (First bacterial effector acting directly on RhoGEFs, EspH directly binds to the DH-PH domain in RhoGEFs to disrupt RhoGEF-Rho signaling; critical for inhibiting macrophage phagocytosis.); EspJ (Inhibit both IgG- and complement receptor-mediated phagocytosis.); EspK; EspL1; EspL2 (Cysteine protease. Bounds F-actin-aggregating annexin 2 directly to increase annexin 2's ability to aggregate Tir-induced F-actin; block necroptosis and in flammation.); EspL4; EspM1 (GEF. Activates the RhoA signaling pathway and induce the formation of stress fibres; inhibit pedestal formation and induce tight junction mislocalization.); EspM2 (GEF. Activates the RhoA signaling pathway and induce the formation of stress fibres; inhibit pedestal formation and induce tight junction mislocalization.); EspN; EspO1-1; EspO1-2; EspR1; EspR3; EspR4; EspT

(GEF. Activates Rac1 and Cdc42 leading to formation of membrane ruffles and lamellipodia; induces membrane ruffles to facilitate bacterial invasion into non-phagocytic cells in a process involving Rac1 and Wave2.); EspW; EspX1; EspX2; EspX4; EspX5; EspX6; EspX7/nleL (E3 ubiquitin ligase, HECT-like. Modulates pedestal formation.); EspY1; EspY2; EspY3; EspY4; EspY5; Map (GEF. Mimics the host Dbl and catalyses the exchange of GDP for GTP in Cdc42, involved in effacement, SGLT1 inhibition, formation of filopodia and disruption of mitochondrial function.); NleA/espI (Disruption of tight junctions by inhibition of host cell protein trafficking through COPII-dependent pathways.); NleB1 (Blocks translocation of the p65 and to the host cell nucleus to inhibit NF-<kappa>B pathway, but NleE and NleB act at different points in the NF-<kappa>B signaling pathway.); NleB2 (May also have anti-inflammatory

activity.); NleC (Metalloprotease. Zn-dependent endopeptidases that specifically clip and inactivate RelA (p65), thus blocking NF-<kappa>B pathway.); NleD (Metalloprotease. Zn-dependent endopeptidases that specifically clip and inactivate JNK and p38, thus blocking AP-1 pathway.); NleE (PMN tran-epithelial migration; blocks translocation of the p65 to the host cell nucleus by preventing I<kappa>B degradation to inhibit NF-<kappa>B pathway.); NleF; NleG-1; NleG2-2; NleG2-3; NleG2-4; NleG5-1; NleG5-2; NleG6-1; NleG6-2; NleG6-3; NleG7 (U-box type E3 ubiquitin ligases.); NleG8-2; NleH1 (Ser/Thr protein kinase. Binds directly to a subunit of NF-<kappa>B, the ribosomal protein S3 (RPS3), reducing the nuclear abundance of RPS3 to dampen host transcriptional outputs; interact with Bax inhibitor-1 to block apoptosis.); NleH2 (Putative kinase. Attenuates NF-<kappa>B pathway.); SepZ/espZ (EspZ interacts with CD98 in

					host cell membranes to promote host cell survival, therefore provide the pathogen with valuable time to colonize efficiently prior to dissemination.); TccP2; Tir (Mimics host immunoreceptor tyrosine-based inhibition motifs (ITIMs), also see helicobacter CagA. EHEC Tir lacks the Nck binding site. Conserved NPY (Asn-Pro-Tyr) motif recruits the adaptor protein IRTKS and/or IRSp53. IRTKS/IRSp53 link Tir and TccP/EspFu, which in turn activates N-WASP; Receptor for intimin; effacement; SGLT1 inhibition; recruits SHIP2 to control actin-pedestal morphology; maintains the integrity of the epithelium by keeping the destructive activity of EspG and EspG2 in check.)								
ECs_2487	msrB	methionine sulfoxide reductase B	VFG037105 (gb|WP_002233356)	MsrAB (VF0456)	Repairs oxidized proteins	setA	Neisseria meningitidis WUE 2594	Stress survival	msrA/BpilB	trifunctional thioredoxin/methionine sulfoxide reductase	50.8	1.15E-37	133

										A/B protein			
ECs_5197	msrA	methionine sulfoxide reductase A	VFG037105 (gb|WP_002233356)	MsrAB (VF0456)	Repairs oxidized proteins	setA	Neisseria meningitidis WUE 2594	Stress survival	msrA/BpilB	trifunctional thioredoxin/methionine sulfoxide reductase A/B protein	42.5	9.78E-33	123
ECs_2488	gapA	glyceraldehyde-3-phosphate dehydrogenase A	VFG005359 (gb|WP_002899102)	Streptococcal plasmin receptor/GAPDH (VF1042)	-	setB	Streptococcus sanguinis SK36	Adherence	plr/gapA	type I glyceraldehyde-3-phosphate dehydrogenase	50.7	3.17E-111	326
ECs_2022	gapC	glyceraldehyde-3-phosphate dehydrogenase C	VFG005356 (gb|WP_000260666)	Streptococcal plasmin receptor/GAPDH (VF1042)	-	setB	Streptococcus pneumoniae D39	Adherence	plr/gapA	type I glyceraldehyde-3-phosphate dehydrogenase	58.9	4.05E-132	379
ECs_3798	epd	D-erythrose 4-phosphate dehydroge	VFG005356 (gb|WP_000260666)	Streptococcal plasmin receptor/GAPDH	-	setB	Streptococcus pneumoniae D39	Adherence	plr/gapA	type I glyceraldehyde-3-phosphate dehydrogen	38.6	9.63E-72	226

		nase		(VF1042)						ase			
ECs_0131	yadG	ABC transporter ATPase	VFG007909 (gb|WP_003876146)	PDIM (VF0309)	Play a mainly structural role in providing a stable base for the insertion of other lipid and also play a role as a fluidity modifier, whose function could be to modulate cell wall viscosity;PDIM is crucial for infection by masking pathogen-associated molecular patterns (PAMP) of the cell wall from the innate immune system	setA	Mycobacterium avium subsp. paratuberculosis K-10	Immune modulation	ddrA	daunorubicin resistance protein DrrA family ABC transporter ATP-binding protein	34.7	4.02E-49	166
ECs_4425	eptB	KDO phosphoethanolamine transferase	VFG036812 (gb|WP_002216735)	Phosphoethanolamine modification (VF0899)	-	setA	Neisseria meningitidis Z2491	Post-translational modification	lptA	lipooligosaccharide phosphoethanolamine transferase LptA	26.5	5.19E-35	138
ECs_5096	eptA	lipid A phosphoethanolamine transferase	VFG036812 (gb|WP_002216735)	Phosphoethanolamine modification (VF0899)	-	setA	Neisseria meningitidis Z2491	Post-translational modification	lptA	lipooligosaccharide phosphoethanolamine transferase LptA	44.1	2.81E-147	434
ECs_1317	eptA	membrane-associated	VFG036812 (gb|WP_002216735)	Phosphoethanolamine	-	setA	Neisseria meningitidis Z2491	Post-translational modificati	lptA	lipooligosaccharide phosphoeth	40.1	1.34E-128	386

		metal-dependent hydrolase		modification (VF0899)				on		anolamine transferase LptA			
ECs_2088	ddpD	D%2CD-dipeptide ABC transporter ATPase	VFG016532 (gb|NP_975942)	Capsule (VF0881)	-	setA	Mycoplasma mycoides subsp. mycoides SC str. PG1	Immune modulation	oppF	oligopeptide ABC transporter permease	36.2	2.21E-23	98.2
ECs_1747	oppF	oligopeptide ABC transporter ATPase	VFG016532 (gb|NP_975942)	Capsule (VF0881)	-	setA	Mycoplasma mycoides subsp. mycoides SC str. PG1	Immune modulation	oppF	oligopeptide ABC transporter permease	51.1	2.29E-40	144
ECs_3468	yfiB	OM lipoprotein positive effector of YfiN activity	VFG031435 (gb|WP_158019550)	Pore-forming protein (VF0850)	-	setA	Mycobacterium sp. JDM601	Stress survival	ompA	OmpA family protein	36.7	5.21E-18	78.6
ECs_4437	yiaD	multicopy suppressor of bamB	VFG031435 (gb|WP_158019550)	Pore-forming protein (VF0850)	-	setA	Mycobacterium sp. JDM601	Stress survival	ompA	OmpA family protein	48.1	9.87E-23	93.2
ECs_1866	eefD	membrane transport	VFG047349 (gb|WP_00	Rhizoferrin	Siderophore-mediated Iron acquisition	setA	Francisella novicida	Nutritional/Metaboli	fslD	MFS transporte	31.4	9.74E-34	129

		protein	3041328)	(VF0551)			U112	c factor		r, inner membrane protein responsible for uptake of the ferric-siderophore complex			
ECs_3074	bcr	drug efflux system	VFG047349 (gb|WP_003041328)	Rhizoferrin (VF0551)	Siderophore-mediated Iron acquisition	setA	Francisella novicida U112	Nutritional/Metabolic factor	fslD	MFS transporter, inner membrane protein responsible for uptake of the ferric-siderophore complex	21.4	4.25E-25	105
ECs_4614	emrD	multidrug efflux system protein	VFG047349 (gb|WP_003041328)	Rhizoferrin (VF0551)	Siderophore-mediated Iron acquisition	setA	Francisella novicida U112	Nutritional/Metabolic factor	fslD	MFS transporter, inner membrane	23	8.90E-20	90.1

										protein responsible for uptake of the ferric-siderophore complex			
ECs_2369	ydhC	arabinose efflux transporter	VFG047349 (gb|WP_003041328)	Rhizoferrin (VF0551)	Siderophore-mediated Iron acquisition	setA	Francisella novicida U112	Nutritional/Metabolic factor	fslD	MFS transporter, inner membrane protein responsible for uptake of the ferric-siderophore complex	25.9	5.60E-24	102
ECs_0922	mdfA	multidrug efflux system protein	VFG047349 (gb|WP_003041328)	Rhizoferrin (VF0551)	Siderophore-mediated Iron acquisition	setA	Francisella novicida U112	Nutritional/Metabolic factor	fslD	MFS transporter, inner membrane protein responsibl	20.9	4.69E-11	63.9

										e for uptake of the ferric-siderophore complex			
ECs_1297	ECs_1297	hypothetical protein	VFG007874 (gb|WP_011780330)	PDIM (VF0309)	Play a mainly structural role in providing a stable base for the insertion of other lipid and also play a role as a fluidity modifier, whose function could be to modulate cell wall viscosity;PDIM is crucial for infection by masking pathogen-associated molecular patterns (PAMP) of the cell wall from the innate immune system	setA	Mycobacterium vanbaaleni i PYR-1	Immune modulation	ppsE	type I polyketide synthase	27.7	1.00E-20	92.8
ECs_1470	fabD	malonyl-CoA-[acyl-carrier-protein] transacylase	VFG007874 (gb|WP_011780330)	PDIM (VF0309)	Play a mainly structural role in providing a stable base for the insertion of other lipid and also play a role as a fluidity modifier, whose function could be to modulate cell wall viscosity;PDIM is crucial for infection by masking pathogen-associated molecular patterns (PAMP) of the cell wall from the innate immune system	setA	Mycobacterium vanbaaleni i PYR-1	Immune modulation	ppsE	type I polyketide synthase	33.8	3.48E-30	120

ECs_1290	ECs_1290	beta-keto acyl synthase	VFG026442 (gb|ACA57618)	Mycolactone (VF0817)	-	setB	Mycobacterium liflandii 128FXT	Exotoxin	mlsB	type I modular polyketide synthase, MlsB	24.1	1.06E-10	63.9
ECs_3701	yqeF	short chain acyltransferase	VFG009412 (gb|WP_011178521)	Mycolactone (VF0817)	-	setB	Mycobacterium ulcerans Agy99	Exotoxin	mlsA1	SDR family NAD(P)-dependent oxidoreductase	26.5	1.26E-06	50.8
ECs_0558	ybbA	ABC transporter ATPase	VFG030678 (gb|WP_011894706)	Trehalose-recycling ABC transporter (VF0842)	-	setA	Mycobacterium gilvum PYR-GCK	Nutritional/Metabolic factor	sugC	sn-glycerol-3-phosphate ABC transporter ATP-binding protein UgpC	35.3	1.24E-30	116
ECs_4387	ECs_4387	ATP-binding component of hemin transport system	VFG044236 (gb|WP_011815405)	Direct heme uptake system (VF1031)	-	setA	Yersinia enterocolitica subsp. enterocolitica 8081	Nutritional/Metabolic factor	YE_RS01715	heme ABC transporter ATP-binding protein	58.3	9.96E-104	301
ECs_2416	btuD	vitamin B12 ABC	VFG044135 (gb|WP_00	Direct heme	-	setA	Yersinia pestis CO92	Nutritional/Metaboli	YPO_RS02410	heme ABC transporte	34.5	4.37E-27	105

		transporter ATPase	2209058)	uptake system (VF1030)				c factor		r ATP-binding protein			
ECs_1880	pspF	psp operon transcriptional activator	VFG001214 (gb|NP_253237)	Type IV pili (VF0082)	Attaches to host cells, but not to mucin, causing a twitching motility that allows the bacteria to move along the cell surface; biofilm formation	setA	Pseudomonas aeruginosa PAO1	Adherence	pilR	two-component response regulator PilR	40.1	1.99E-65	212
ECs_4480	ECs_4480	adhesin	VFG035941 (gb|WP_001033191)	UpaG adhesin, trimeric AT (VF1125)	-	setA	Escherichia coli O157:H7 str. EDL933	Adherence	upaG/ehaG	trimeric autotransporter adhesin EhaG	100	0	2025
ECs_0206	yafE	S-adenosyl-L-methionine-dependent methyltransferase	VFG029672 (gb|WP_015304385)	GPL locus (VF0841)	-	setB	Mycobacterium smegmatis JS623	Immune modulation	fmt	class I SAM-dependent methyltransferase	28.4	4.60E-10	58.5
ECs_4763	ubiE	ubiquinone/menaquinone biosynthesis methyltransferase	VFG029669 (gb|WP_003407670)	GPL locus (VF0841)	-	setB	Mycobacterium tuberculosis str. Beijing/NITR203	Immune modulation	fmt	class I SAM-dependent methyltransferase	31.7	7.36E-07	49.3

ECs_0723	kdpD	sensory histidine kinase in two-component regulatory system	VFG009817 (gb|WP_011738793)	PhoP (VF0286)	Senses Mg2+ starvation, controls expression of genes involved in surface remodeling and adaptation to intracellular growth	setA	Mycobacterium ulcerans Agy99	Regulation	phoR	Possible two component system response sensor kinase membrane associated PhoR	31.8	2.80E-23	104
ECs_2706	yedV	two-component system sensor histidine kinase YedV	VFG027254 (gb|WP_015309037)	MprAB (VF0298)	Required for establishment and maintenance of persistent infection	setA	Mycobacterium smegmatis JS623	Regulation	mprB	HAMP domain-containing histidine kinase	28.5	1.80E-21	96.7
ECs_5357	creC	two-component system sensor histidine kinase CreC	VFG027254 (gb|WP_015309037)	MprAB (VF0298)	Required for establishment and maintenance of persistent infection	setA	Mycobacterium smegmatis JS623	Regulation	mprB	HAMP domain-containing histidine kinase	29.3	1.50E-26	112
ECs_3105	rcsD	phosphotransfer	VFG001747 (gb|WP_00	TTSS (chromoso	-	setA	Yersinia pestis CO92	Effector delivery	YPO_RS02285	two component	33	2.38E-07	54.7

		intermediate protein in two-component regulatory system with RcsBC	2209035)	mally encoded) (VF1023)				system		system sensor kinase			
ECs_0722	kdpE	two-component regulatory system response regulator KdpE	VFG009867 (gb|WP_041309847)	MprAB (VF0298)	Required for establishment and maintenance of persistent infection	setA	Mycobacterium sp. KMS	Regulation	mprA	response regulator transcription factor	40.5	1.52E-50	164
ECs_2707	yedW	response regulator	VFG027229 (gb|WP_041312672)	MprAB (VF0298)	Required for establishment and maintenance of persistent infection	setA	Mycobacterium smegmatis JS623	Regulation	mprA	response regulator transcription factor	40.3	4.23E-51	166
ECs_3907	qseB	quorum sensing DNA-binding response	VFG027229 (gb|WP_041312672)	MprAB (VF0298)	Required for establishment and maintenance of persistent infection	setA	Mycobacterium smegmatis JS623	Regulation	mprA	response regulator transcription factor	42.2	9.62E-53	169

		regulator in two-component regulatory system with QseC											
ECs_5095	basR	two-component regulatory system response regulator BasR	VFG027229 (gb|WP_041312672)	MprAB (VF0298)	Required for establishment and maintenance of persistent infection	setA	Mycobacterium smegmatis JS623	Regulation	mprA	response regulator transcription factor	39.9	3.54E-42	142
ECs_4731	aslA	Ser-type periplasmic non-aryl sulfatase	VFG001444 (gb|AAG10151)	AslA (VF0238)	Contributes to brain microvascular endothelial cells (BMECs) invasion	setA	Escherichia coli O18:K1:H7 str. RS218	Invasion	aslA	putative arylsulfatase	97	0	912
ECs_2103	ydeN	Ser-type periplasmic non-aryl sulfatase	VFG001444 (gb|AAG10151)	AslA (VF0238)	Contributes to brain microvascular endothelial cells (BMECs) invasion	setA	Escherichia coli O18:K1:H7 str. RS218	Invasion	aslA	putative arylsulfatase	26.4	8.89E-34	134
ECs_4732	hemY	protoheme IX	VFG013642 (gb|WP_01	Heme biosynthe	-	setB	Haemophilus somnus	Nutritional/Metaboli	hemY	heme biosynthes	37.5	1.10E-79	251

		synthesis protein	1608206)	sis (VF0758)			129PT	c factor		is protein HemY			
ECs_2937	yehU	histidine kinase	VFG014977 (gb|WP_012077870)	Alginate (VF0091)	Allows the bacteria form biofilm; contributes to the persistence of the bacteria in the CF lung: act as an adhesin, preventing the bacteria from being expelled from the lung, and alginate slime layer makes it more difficult for phagocytes to ingest and kill the bacteria	setA	Pseudomonas aeruginosa PA7	Biofilm	algZ	sigma factor AlgU	32.7	1.90E-22	99
ECs_3260	ypdA	sensor kinase regulating yhjX	VFG014983 (gb|WP_013981628)	Alginate (VF0091)	Allows the bacteria form biofilm; contributes to the persistence of the bacteria in the CF lung: act as an adhesin, preventing the bacteria from being expelled from the lung, and alginate slime layer makes it more difficult for phagocytes to ingest and kill the bacteria	setA	Pseudomonas stutzeri A1501	Biofilm	algZ	sigma factor AlgU	31.2	2.12E-20	92.8
ECs_4734	hemD	uroporphyrinogen III synthase	VFG013199 (gb|WP_012341015)	Heme biosynthesis (VF0758)	-	setB	Haemophilus somnus 2336	Nutritional/Metabolic factor	hemD	uroporphyrinogen-III synthase	41.9	1.76E-56	180
ECs_4735	hemC	porphobilinogen deaminase	VFG013198 (gb|WP_011608203)	Heme biosynthesis (VF0758)	-	setB	Haemophilus somnus 2336	Nutritional/Metabolic factor	hemC	hydroxymethylbilane synthase	62.8	1.45E-130	374

ECs_4089	arcB	aerobic respiration control sensor histidine protein kinase	VFG011123 (gb|WP_010926708)	BvgAS (VF0336)	Positively regulate virulence genes by binding to promoter sequences, negatively regulate virulence repressed genes (vrgs) via BvgR; mediate transition between infectious (Bvg+) and non-infectious (Bvg-) phase; regulating biofilm formation	setA	Bordetella bronchiseptica RB50	Regulation	bvgS	virulence factors two-component system sensor histidine kinase BvgS	31.7	5.70E-52	196
ECs_3249	evgS	hybrid sensory histidine kinase in two-component regulatory system with EvgA	VFG011123 (gb|WP_010926708)	BvgAS (VF0336)	Positively regulate virulence genes by binding to promoter sequences, negatively regulate virulence repressed genes (vrgs) via BvgR; mediate transition between infectious (Bvg+) and non-infectious (Bvg-) phase; regulating biofilm formation	setA	Bordetella bronchiseptica RB50	Regulation	bvgS	virulence factors two-component system sensor histidine kinase BvgS	28.7	1.58E-137	449
ECs_1697	ECs_1697	ferric enterobactin transport ATP-binding protein	VFG044110 (gb|WP_010929751)	Direct heme uptake system (VF0689)	-	setA	Bordetella pertussis Tohama I	Nutritional/Metabolic factor	BP_RS01740	heme ABC transporter ATP-binding protein	36.9	6.85E-36	128
ECs_4081	rpoN	RNA polymeras	VFG042736 (gb|NP_25	Type IV pili	Attaches to host cells, but not to mucin, causing a twitching motility	setA	Pseudomonas	Adherence	rpoN	RNA polymerase	56.2	3.47E-155	450

		e sigma 54 factor RpoN	3152)	(VF0082)	that allows the bacteria to move along the cell surface; biofilm formation		aeruginosa PAO1			factor sigma-54			
ECs_5198	tamA	outer membrane protein	VFG016675 (gb|WP_005766757)	Omp89 (VF0676)	-	setB	Bartonella bacilliformis KC583	Adherence	omp89	outer membrane protein assembly factor BamA	21.7	7.01E-08	55.5
ECs_0179	bamA	BamABCDE complex OM biogenesis outer membrane pore-forming assembly factor	VFG016675 (gb|WP_005766757)	Omp89 (VF0676)	-	setB	Bartonella bacilliformis KC583	Adherence	omp89	outer membrane protein assembly factor BamA	27.1	2.27E-74	258
ECs_0436	proC	pyrroline-5-carboxylate reductase	VFG022639 (gb|WP_013827437)	Proline synthesis (VF0812)	-	setB	Mycobacterium sp. JDM601	Nutritional/Metabolic factor	proC	pyrroline-5-carboxylate reductase	35.6	1.73E-41	144
ECs_0658	citA	sensory histidine kinase in two-compo	VFG024170 (gb|WP_014381773)	PhoP (VF0286)	Senses Mg2+ starvation, controls expression of genes involved in surface remodeling and adaptation to intracellular growth	setA	Mycobacterium indicus pranii MTCC 9506	Regulation	phoR	Possible two component system	28.9	3.06E-09	59.3

		nent regulatory system with CitB								response sensor kinase membrane associated PhoR			
ECs_2886	baeS	two-component system sensor histidine kinase BaeS	VFG024170 (gb|WP_014381773)	PhoP (VF0286)	Senses Mg2+ starvation, controls expression of genes involved in surface remodeling and adaptation to intracellular growth	setA	Mycobacterium indicus pranii MTCC 9506	Regulation	phoR	Possible two component system response sensor kinase membrane associated PhoR	27.8	4.84E-33	130
ECs_0655	citE	citrate lyase beta chain	VFG044282 (gb|WP_012367517)	Proteobactin (VF1252)	-	setB	Proteus mirabilis HI4320	Nutritional/Metabolic factor	PMI_RS01120	CoA ester lyase	28.6	3.32E-21	90.9
ECs_4554	espB	T3SS translocator EspB	VFG012362 (gb|WP_001092000)	TTSS (VF0191)	Injects Tir and other effector molecules directly into the host cell. Effector molecules activate cell-signaling pathways, causing alterations in the host cell cytoskeleton and resulting in the	setA	Escherichia coli O157:H7 str. EDL933	Effector delivery system	espB	Type III secretion system translocator protein EspB, pore	100	3.57E-134	383

					depolymerization of actin and the loss of microvilli					protein			
ECs_4107	degQ	serine endoprotease	VFG014962 (gb|WP_011912408)	Alginate (VF0091)	Allows the bacteria form biofilm; contributes to the persistence of the bacteria in the CF lung: act as an adhesin, preventing the bacteria from being expelled from the lung, and alginate slime layer makes it more difficult for phagocytes to ingest and kill the bacteria	setA	Pseudomonas stutzeri A1501	Biofilm	mucD	serine protease MucD precursor	36.5	5.31E-80	255
ECs_0165	degP	serine endoprotease	VFG015769 (gb|WP_012312989)	Alginate (VF0091)	Allows the bacteria form biofilm; contributes to the persistence of the bacteria in the CF lung: act as an adhesin, preventing the bacteria from being expelled from the lung, and alginate slime layer makes it more difficult for phagocytes to ingest and kill the bacteria	setA	Pseudomonas putida W619	Biofilm	mucD	serine protease MucD precursor	38.4	1.65E-82	263
ECs_4556	espA	T3SS translocator EspA	VFG000800 (gb|WP_000381516)	TTSS (VF0191)	Injects Tir and other effector molecules directly into the host cell. Effector molecules activate cell-signaling pathways, causing alterations in the host cell cytoskeleton and resulting in the depolymerization of actin and the loss of microvilli	setA	Escherichia coli O157:H7 str. EDL933	Effector delivery system	espA	Type III secretion system translocator protein EspA	100	5.41E-121	340

ECs_4557	sepL	T3SS secretion switching protein SepL	VFG000801 (gb|WP_001273445)	TTSS (VF0191)	Injects Tir and other effector molecules directly into the host cell. Effector molecules activate cell-signaling pathways, causing alterations in the host cell cytoskeleton and resulting in the depolymerization of actin and the loss of microvilli	setA	Escherichia coli O157:H7 str. EDL933	Effector delivery system	sepL	Type III secretion system secretion gatekeeper SepL	100	4.84E-242	659
ECs_4550	espF	T3SS secreted effector EspF	VFG033742 (gb|WP_014714127)	TTSS secreted effectors (VF1110)	Cif (Deamidase. Induces cytopathic effects of actin stress fiber formation and cell cycle arrest.); EspB (Pore formation, actin disruption, microvilli effacement, anti-phagocytosis.); EspF (Inducing degradation of the aniapoptic protein AbcF2, tight junction disruption, microvilli effacement and elongation, mitochondrial dysfunction, N-WASP activation, SGLT-1 inactivation, pedestal maturation, inhibition of NHE3 activity, membrane remodelling; targets and disrupts the nucleolus late in infection, which is temporally controlled by host mitochondria.); EspFu/tccP (Inducing degradation of the aniapoptic protein AbcF2, tight	setA	Escherichia coli O157:H7 str. Xuzhou21	Effector delivery system	espF	Type III secretion system effector EspF	100	1.77E-71	215

junction disruption, microvilli effacement and elongation, mitochondrial dysfunction, N-WASP activation, SGLT-1 inactivation, pedestal maturation, inhibition of NHE3 activity, membrane remodelling; targets and disrupts the nucleolus late in infection, which is temporally controlled by host mitochondria.); EspG (TBC-like GTPase activating protein. Efficiently catalyzes GTP hydrolysis in Rab1 to disrupt of Rab1-mediated ER-to-Golgi trafficking.); EspH (First bacterial effector acting directly on RhoGEFs, EspH directly binds to the DH-PH domain in RhoGEFs to disrupt RhoGEF-Rho signaling; critical for inhibiting macrophage phagocytosis.); EspJ (Inhibit both IgG- and complement receptor-mediated phagocytosis.); EspK; EspL1; EspL2 (Cysteine protease. Bounds F-actin-aggregating annexin 2 directly to increase annexin 2's ability to aggregate Tir-induced F-actin; block necroptosis and in

flammation.); EspL4; EspM1 (GEF. Activates the RhoA signaling pathway and induce the formation of stress fibres; inhibit pedestal formation and induce tight junction mislocalization.); EspM2 (GEF. Activates the RhoA signaling pathway and induce the formation of stress fibres; inhibit pedestal formation and induce tight junction mislocalization.); EspN; EspO1-1; EspO1-2; EspR1; EspR3; EspR4; EspT (GEF. Activates Rac1 and Cdc42 leading to formation of membrane ruffles and lamellipodia; induces membrane ruffles to facilitate bacterial invasion into non-phagocytic cells in a process involving Rac1 and Wave2.); EspW; EspX1; EspX2; EspX4; EspX5; EspX6; EspX7/nleL (E3 ubiquitin ligase, HECT-like. Modulates pedestal formation.); EspY1; EspY2; EspY3; EspY4; EspY5; Map (GEF. Mimics the host Dbl and catalyses the exchange of GDP for GTP in Cdc42, involved in effacement, SGLT1 inhibition,

formation of filopodia and disruption of mitochondrial function.); NleA/espI (Disruption of tight junctions by inhibition of host cell protein trafficking through COPII-dependent pathways.); NleB1 (Blocks translocation of the p65 and to the host cell nucleus to inhibit NF-<kappa>B pathway, but NleE and NleB act at different points in the NF-<kappa>B signaling pathway.); NleB2 (May also have anti-inflammatory activity.); NleC (Metalloprotease. Zn-dependent endopeptidases that specifically clip and inactivate RelA (p65), thus blocking NF-<kappa>B pathway.); NleD (Metalloprotease. Zn-dependent endopeptidases that specifically clip and inactivate JNK and p38, thus blocking AP-1 pathway.); NleE (PMN tran-epithelial migration; blocks translocation of the p65 to the host cell nucleus by preventing I<kappa>B degradation to inhibit NF-<kappa>B pathway.); NleF; NleG-1; NleG2-2; NleG2-3; NleG2-4; NleG5-1;

NleG5-2; NleG6-1; NleG6-2; NleG6-3; NleG7 (U-box type E3 ubiquitin ligases.); NleG8-2; NleH1 (Ser/Thr protein kinase. Binds directly to a subunit of NF-<kappa>B, the ribosomal protein S3 (RPS3), reducing the nuclear abundance of RPS3 to dampen host transcriptional outputs; interact with Bax inhibitor-1 to block apoptosis.); NleH2 (Putative kinase. Attenuates NF-<kappa>B pathway.); SepZ/espZ (EspZ interacts with CD98 in host cell membranes to promote host cell survival, therefore provide the pathogen with valuable time to colonize efficiently prior to dissemination.); TccP2; Tir (Mimics host immunoreceptor tyrosine-based inhibition motifs (ITIMs), also see helicobacter CagA. EHEC Tir lacks the Nck binding site. Conserved NPY (Asn-Pro-Tyr) motif recruits the adaptor protein IRTKS and/or IRSp53. IRTKS/IRSp53 link Tir and TccP/EspFu, which in turn activates N-WASP; Receptor for intimin; effacement;

					SGLT1 inhibition; recruits SHIP2 to control actin-pedestal morphology; maintains the integrity of the epithelium by keeping the destructive activity of EspG and EspG2 in check.)								
ECs_3826	yggR	twitching motility protein PilT	VFG042879 (gb|WP_011079462)	ChiRP (VF0609)	PilA is involved in biofilm formation, adherence to human epithelial cells.	setA	Vibrio vulnificus CMCP6	Adherence	VV1_RS07215	type IV pilus twitching motility protein PilT	49.2	2.03E-100	299
ECs_4552	escF	T3SS structure protein EscF	VFG000796 (gb|WP_001053840)	TTSS (VF0191)	Injects Tir and other effector molecules directly into the host cell. Effector molecules activate cell-signaling pathways, causing alterations in the host cell cytoskeleton and resulting in the depolymerization of actin and the loss of microvilli	setA	Escherichia coli O157:H7 str. EDL933	Effector delivery system	escF	Type III secretion system needle filament protein EscF	100	2.89E-46	141
ECs_4558	escD	T3SS structure protein EscD	VFG000802 (gb|WP_000953255)	TTSS (VF0191)	Injects Tir and other effector molecules directly into the host cell. Effector molecules activate cell-signaling pathways, causing alterations in the host cell cytoskeleton and resulting in the depolymerization of actin and the loss	setA	Escherichia coli O157:H7 str. EDL933	Effector delivery system	escD	Type III secretion system outer MS ring protein EscD	100	9.33E-302	815

					of microvilli								
ECs_3857	nleB1	T3SS secreted effector NleB	VFG034932 (gb|WP_000953022)	TTSS secreted effectors (VF1110)	Cif (Deamidase. Induces cytopathic effects of actin stress fiber formation and cell cycle arrest.); EspB (Pore formation, actin disruption, microvilli effacement, anti-phagocytosis.); EspF (Inducing degradation of the aniapoptic protein AbcF2, tight junction disruption, microvilli effacement and elongation, mitochondrial dysfunction, N-WASP activation, SGLT-1 inactivation, pedestal maturation, inhibition of NHE3 activity, membrane remodelling; targets and disrupts the nucleolus late in infection, which is temporally controlled by host mitochondria.); EspFu/tccP (Inducing degradation of the aniapoptic protein AbcF2, tight junction disruption, microvilli effacement and elongation, mitochondrial dysfunction, N-WASP activation, SGLT-1 inactivation, pedestal maturation, inhibition of NHE3 activity, membrane remodelling; targets and disrupts the nucleolus	setA	Escherichia coli O157:H7 str. EDL933	Effector delivery system	nleB1	Type III secretion system effector nleB1	100	1.35E-246	669

late in infection, which is temporally controlled by host mitochondria.); EspG (TBC-like GTPase activating protein. Efficiently catalyzes GTP hydrolysis in Rab1 to disrupt of Rab1-mediated ER-to-Golgi trafficking.); EspH (First bacterial effector acting directly on RhoGEFs, EspH directly binds to the DH-PH domain in RhoGEFs to disrupt RhoGEF-Rho signaling; critical for inhibiting macrophage phagocytosis.); EspJ (Inhibit both IgG- and complement receptor-mediated phagocytosis.); EspK; EspL1; EspL2 (Cysteine protease. Bounds F-actin-aggregating annexin 2 directly to increase annexin 2's ability to aggregate Tir-induced F-actin; block necroptosis and in flammation.); EspL4; EspM1 (GEF. Activates the RhoA signaling pathway and induce the formation of stress fibres; inhibit pedestal formation and induce tight junction mislocalization.); EspM2 (GEF. Activates the RhoA signaling pathway

and induce the formation of stress fibres; inhibit pedestal formation and induce tight junction mislocalization.); EspN; EspO1-1; EspO1-2; EspR1; EspR3; EspR4; EspT (GEF. Activates Rac1 and Cdc42 leading to formation of membrane ruffles and lamellipodia; induces membrane ruffles to facilitate bacterial invasion into non-phagocytic cells in a process involving Rac1 and Wave2.); EspW; EspX1; EspX2; EspX4; EspX5; EspX6; EspX7/nleL (E3 ubiquitin ligase, HECT-like. Modulates pedestal formation.); EspY1; EspY2; EspY3; EspY4; EspY5; Map (GEF. Mimics the host Dbl and catalyses the exchange of GDP for GTP in Cdc42, involved in effacement, SGLT1 inhibition, formation of filopodia and disruption of mitochondrial function.); NleA/espI (Disruption of tight junctions by inhibition of host cell protein trafficking through COPII-dependent pathways.); NleB1 (Blocks translocation of the p65 and to

the host cell nucleus to inhibit NF-<kappa>B pathway, but NleE and NleB act at different points in the NF-<kappa>B signaling pathway.); NleB2 (May also have anti-inflammatory activity.); NleC (Metalloprotease. Zn-dependent endopeptidases that specifically clip and inactivate RelA (p65), thus blocking NF-<kappa>B pathway.); NleD (Metalloprotease. Zn-dependent endopeptidases that specifically clip and inactivate JNK and p38, thus blocking AP-1 pathway.); NleE (PMN tran-epithelial migration; blocks translocation of the p65 to the host cell nucleus by preventing I<kappa>B degradation to inhibit NF-<kappa>B pathway.); NleF; NleG-1; NleG2-2; NleG2-3; NleG2-4; NleG5-1; NleG5-2; NleG6-1; NleG6-2; NleG6-3; NleG7 (U-box type E3 ubiquitin ligases.); NleG8-2; NleH1 (Ser/Thr protein kinase. Binds directly to a subunit of NF-<kappa>B, the ribosomal protein S3 (RPS3), reducing the nuclear abundance of RPS3 to dampen

					host transcriptional outputs; interact with Bax inhibitor-1 to block apoptosis.); NleH2 (Putative kinase. Attenuates NF-<kappa>B pathway.); SepZ/espZ (EspZ interacts with CD98 in host cell membranes to promote host cell survival, therefore provide the pathogen with valuable time to colonize efficiently prior to dissemination.); TccP2; Tir (Mimics host immunoreceptor tyrosine-based inhibition motifs (ITIMs), also see helicobacter CagA. EHEC Tir lacks the Nck binding site. Conserved NPY (Asn-Pro-Tyr) motif recruits the adaptor protein IRTKS and/or IRSp53. IRTKS/IRSp53 link Tir and TccP/EspFu, which in turn activates N-WASP; Receptor for intimin; effacement; SGLT1 inhibition; recruits SHIP2 to control actin-pedestal morphology; maintains the integrity of the epithelium by keeping the destructive activity of EspG and EspG2 in check.)								
ECs_0846	nleB2-1	T3SS secreted	VFG034932 (gb|WP_00	TTSS secreted	Cif (Deamidase. Induces cytopathic effects of actin stress fiber	setA	Escherichia coli	Effector delivery	nleB1	Type III secretion	61.5	2.94E-143	407

effector NleB	0953022)	effectors (VF1110)	formation and cell cycle arrest.); EspB (Pore formation, actin disruption, microvilli effacement, anti-phagocytosis.); EspF (Inducing degradation of the aniapoptic protein AbcF2, tight junction disruption, microvilli effacement and elongation, mitochondrial dysfunction, N-WASP activation, SGLT-1 inactivation, pedestal maturation, inhibition of NHE3 activity, membrane remodelling; targets and disrupts the nucleolus late in infection, which is temporally controlled by host mitochondria.); EspFu/tccP (Inducing degradation of the aniapoptic protein AbcF2, tight junction disruption, microvilli effacement and elongation, mitochondrial dysfunction, N-WASP activation, SGLT-1 inactivation, pedestal maturation, inhibition of NHE3 activity, membrane remodelling; targets and disrupts the nucleolus late in infection, which is temporally controlled by host mitochondria.); EspG (TBC-like GTPase activating	O157:H7 str. EDL933	system	system effector nleB1

protein. Efficiently catalyzes GTP hydrolysis in Rab1 to disrupt of Rab1-mediated ER-to-Golgi trafficking.); EspH (First bacterial effector acting directly on RhoGEFs, EspH directly binds to the DH-PH domain in RhoGEFs to disrupt RhoGEF-Rho signaling; critical for inhibiting macrophage phagocytosis.); EspJ (Inhibit both IgG- and complement receptor-mediated phagocytosis.); EspK; EspL1; EspL2 (Cysteine protease. Bounds F-actin-aggregating annexin 2 directly to increase annexin 2's ability to aggregate Tir-induced F-actin; block necroptosis and in flammation.); EspL4; EspM1 (GEF. Activates the RhoA signaling pathway and induce the formation of stress fibres; inhibit pedestal formation and induce tight junction mislocalization.); EspM2 (GEF. Activates the RhoA signaling pathway and induce the formation of stress fibres; inhibit pedestal formation and induce tight junction

mislocalization.); EspN; EspO1-1; EspO1-2; EspR1; EspR3; EspR4; EspT (GEF. Activates Rac1 and Cdc42 leading to formation of membrane ruffles and lamellipodia; induces membrane ruffles to facilitate bacterial invasion into non-phagocytic cells in a process involving Rac1 and Wave2.); EspW; EspX1; EspX2; EspX4; EspX5; EspX6; EspX7/nleL (E3 ubiquitin ligase, HECT-like. Modulates pedestal formation.); EspY1; EspY2; EspY3; EspY4; EspY5; Map (GEF. Mimics the host Dbl and catalyses the exchange of GDP for GTP in Cdc42, involved in effacement, SGLT1 inhibition, formation of filopodia and disruption of mitochondrial function.); NleA/espI (Disruption of tight junctions by inhibition of host cell protein trafficking through COPII-dependent pathways.); NleB1 (Blocks translocation of the p65 and to the host cell nucleus to inhibit NF-<kappa>B pathway, but NleE and NleB act at different points in the

NF-<kappa>B signaling pathway.); NleB2 (May also have anti-inflammatory activity.); NleC (Metalloprotease. Zn-dependent endopeptidases that specifically clip and inactivate RelA (p65), thus blocking NF-<kappa>B pathway.); NleD (Metalloprotease. Zn-dependent endopeptidases that specifically clip and inactivate JNK and p38, thus blocking AP-1 pathway.); NleE (PMN tran-epithelial migration; blocks translocation of the p65 to the host cell nucleus by preventing I<kappa>B degradation to inhibit NF-<kappa>B pathway.); NleF; NleG-1; NleG2-2; NleG2-3; NleG2-4; NleG5-1; NleG5-2; NleG6-1; NleG6-2; NleG6-3; NleG7 (U-box type E3 ubiquitin ligases.); NleG8-2; NleH1 (Ser/Thr protein kinase. Binds directly to a subunit of NF-<kappa>B, the ribosomal protein S3 (RPS3), reducing the nuclear abundance of RPS3 to dampen host transcriptional outputs; interact with Bax inhibitor-1 to block apoptosis.); NleH2 (Putative kinase.

					Attenuates NF-<kappa>B pathway.); SepZ/espZ (EspZ interacts with CD98 in host cell membranes to promote host cell survival, therefore provide the pathogen with valuable time to colonize efficiently prior to dissemination.); TccP2; Tir (Mimics host immunoreceptor tyrosine-based inhibition motifs (ITIMs), also see helicobacter CagA. EHEC Tir lacks the Nck binding site. Conserved NPY (Asn-Pro-Tyr) motif recruits the adaptor protein IRTKS and/or IRSp53. IRTKS/IRSp53 link Tir and TccP/EspFu, which in turn activates N-WASP; Receptor for intimin; effacement; SGLT1 inhibition; recruits SHIP2 to control actin-pedestal morphology; maintains the integrity of the epithelium by keeping the destructive activity of EspG and EspG2 in check.)								
ECs_0231	impC	type VI secretion system protein ImpC	VFG035760 (gb|WP_000056994)	EHS (VF1176)	-	setA	Escherichia coli O157:H7 str. EDL933	Effector delivery system	aec18	type VI secretion system contractile sheath	100	0	933

ECs_0230	ECs_0230	type VI secretion protein	VFG035735 (gb|WP_000599596)	EHS (VF1176)	-	setA	Escherichia coli O157:H7 str. EDL933	Effector delivery system	aec19	large subunit type VI secretion system baseplate subunit TssE	100	1.19E-95	271
ECs_0233	impB	type VI secretion system protein ImpB	VFG035783 (gb|WP_000037399)	EHS (VF1176)	-	setA	Escherichia coli O157:H7 str. EDL933	Effector delivery system	aec17	type VI secretion system contractile sheath small subunit	100	7.50E-113	317
ECs_0232	ECs_0232	hypothetical protein	VFG041049 (gb|WP_000123970)	EHS (VF1176)	-	setB	Escherichia coli O157:H7 str. EDL933	Effector delivery system	Z_RS01215	hypothetical protein	100	1.65E-50	152
ECs_0234	ECs_0234	Hcp-like protein	VFG035808 (gb|WP_001142958)	EHS (VF1176)	-	setA	Escherichia coli O157:H7 str. EDL933	Effector delivery system	aec16	Hcp family type VI secretion system effector	100	2.41E-130	362
ECs_2104	ydeO	UV-inducible	VFG018546 (gb|WP_00	TTSS (SPI-1	Delivers at least 13 effector proteins through the host cell plasma membrane,	setA	Salmonella enterica	Effector delivery	hilD	transcriptional	38.7	7.32E-21	89.4

		global regulator	0432694)	encode) (VF0116)	most of them are involved in actin cytoskeleton rearrangements, leading to membrane ruffling and Salmonella invasion		subsp. arizonae serovar 62:z4,z23:-- str. RSK2980	system		regulator HilD			
ECs_4396	gadX	acid resistance regulon transcriptional activator	VFG018546 (gb|WP_000432694)	TTSS (SPI-1 encode) (VF0116)	Delivers at least 13 effector proteins through the host cell plasma membrane, most of them are involved in actin cytoskeleton rearrangements, leading to membrane ruffling and Salmonella invasion	setA	Salmonella enterica subsp. arizonae serovar 62:z4,z23:-- str. RSK2980	Effector delivery system	hilD	transcriptional regulator HilD	37	9.19E-18	81.3
ECs_4395	gadW	transcriptional activator	VFG018546 (gb|WP_000432694)	TTSS (SPI-1 encode) (VF0116)	Delivers at least 13 effector proteins through the host cell plasma membrane, most of them are involved in actin cytoskeleton rearrangements, leading to membrane ruffling and Salmonella invasion	setA	Salmonella enterica subsp. arizonae serovar 62:z4,z23:-- str. RSK2980	Effector delivery system	hilD	transcriptional regulator HilD	36	1.52E-20	88.2
ECs_1087	ECs_1087	AraC-family transcriptional	VFG000537 (gb|NP_461796)	TTSS (SPI-1 encode) (VF0116)	Delivers at least 13 effector proteins through the host cell plasma membrane, most of them are involved in actin cytoskeleton rearrangements, leading	setA	Salmonella enterica subsp. enterica	Effector delivery system	hilD	AraC family transcriptional regulator	38.1	9.61E-15	70.9

		regulator			to membrane ruffling and Salmonella invasion		serovar Typhimurium str. LT2						
ECs_2191	ECs_2191	phage regulatory protein	VFG000537 (gb|NP_461796)	TTSS (SPI-1 encode) (VF0116)	Delivers at least 13 effector proteins through the host cell plasma membrane, most of them are involved in actin cytoskeleton rearrangements, leading to membrane ruffling and Salmonella invasion	setA	Salmonella enterica subsp. enterica serovar Typhimurium str. LT2	Effector delivery system	hilD	AraC family transcriptional regulator	33.8	3.71E-15	72
ECs_2107	ydeQ	fimbrial-like adhesin protein	VFG042718 (gb|WP_000520684)	F9 fimbriae (VF1153)	-	setA	Escherichia coli O157:H7 str. EDL933	Adherence	ydeQ	fimbrial protein	100	5.54E-222	604
ECs_2101	yddA	multidrug ABC transporter permease/ATPase	VFG035923 (gb|WP_000621745)	Dispersin (VF0215)	Promotes dispersal of EAEC on the intestinal mucosa to establish new foci of infection and facilitate efficient colonization via bacterial dispersal	setA	Escherichia coli 044:H18 042	Others	aatC	ATP-binding protein AatC	28.2	2.01E-11	63.5
ECs_5040	uvrA	excinuclease ABC system protein UvrA	VFG035923 (gb|WP_000621745)	Dispersin (VF0215)	Promotes dispersal of EAEC on the intestinal mucosa to establish new foci of infection and facilitate efficient colonization via bacterial dispersal	setA	Escherichia coli 044:H18 042	Others	aatC	ATP-binding protein AatC	32.4	4.79E-09	57.4
gene-hlyB	hlyB	Hemolysin	VFG033885	Hemolysin	Cytotoxic to many types of cells:	setA	Escherichi	Exotoxin	hlyB	Hemolysin B	73.4	0	1030

		B	(gb|WP_000376538)	(VF0207)	erythrocytes, granulocytes, monocytes, endothelial cells and renal epithelial cells; stimulating the release of IL-1<beta> and TNF		a coli UMNK88						
ECs_1033	uup	replication regulatory ABC-F family DNA-binding ATPase	VFG000907 (gb|WP_000376545)	<alpha>-Hemolysin (VF0225)	Cytotoxic to many types of cells: erythrocytes, granulocytes, monocytes, endothelial cells and renal epithelial cells; stimulating the release of IL-1<beta> and TNF	setA	Escherichia coli CFT073	Exotoxin	hlyB	Hemolysin B	29.7	2.83E-15	79.3
ECs_0543	lapB	ABC transporter ATP-binding protein	VFG000907 (gb|WP_000376545)	<alpha>-Hemolysin (VF0225)	Cytotoxic to many types of cells: erythrocytes, granulocytes, monocytes, endothelial cells and renal epithelial cells; stimulating the release of IL-1<beta> and TNF	setA	Escherichia coli CFT073	Exotoxin	hlyB	Hemolysin B	25.8	1.44E-71	246
ECs_2109	ydeS	fimbrial-like adhesin protein	VFG033311 (gb|WP_000876770)	Type 1 fimbriae (VF0221)	Makes an important contribution to colonization of the bladder	setA	Escherichia coli O44:H18 042	Adherence	fimF	FimF protein precursor	100	8.32E-124	345
ECs_3148	menE	O-succinylbenzoate-CoA ligase	VFG030387 (gb|WP_015304695)	MymA operon (VF0840)	-	setA	Mycobacterium smegmatis JS623	Immune modulation	tgs4	long-chain-fatty-acid--CoA ligase	26.1	4.29E-29	119
ECs_5051	acs	acetyl-Co	VFG030387	MymA	-	setA	Mycobacter	Immune	tgs4	long-chain	26.9	5.24E-	124

		A synthetase	(gb|WP_015304695)	operon (VF0840)			ium smegmatis JS623	modulation		-fatty-acid--CoA ligase		30	
ECs_0388	prpE	propionate--CoA ligase	VFG030387 (gb|WP_015304695)	MymA operon (VF0840)	-	setA	Mycobacterium smegmatis JS623	Immune modulation	tgs4	long-chain-fatty-acid--CoA ligase	24.2	4.59E-30	124
ECs_2514	fadD	long-chain-fatty-acid--CoA ligase	VFG030387 (gb|WP_015304695)	MymA operon (VF0840)	-	setA	Mycobacterium smegmatis JS623	Immune modulation	tgs4	long-chain-fatty-acid--CoA ligase	30.3	1.76E-61	211
ECs_0040	caiC	crotonobetaine/carnitine-CoA ligase	VFG030387 (gb|WP_015304695)	MymA operon (VF0840)	-	setA	Mycobacterium smegmatis JS623	Immune modulation	tgs4	long-chain-fatty-acid--CoA ligase	31.8	7.92E-62	211
ECs_3693	aas	acyl-ACP synthetase	VFG030400 (gb|WP_081465621)	MymA operon (VF0840)	-	setA	Mycobacterium sp. JDM601	Immune modulation	fadD13	long-chain fatty acid--CoA ligase	25.6	7.24E-26	112
ECs_3292	cysM	cysteine synthase B	VFG044254 (gb|WP_000570808)	Staphyloferrin B (VF1014)	-	setB	Staphylococcus aureus subsp. aureus str. Newman	Nutritional/Metabolic factor	sbnA	2,3-diaminopropionate biosynthesis protein SbnA	35.1	5.02E-38	137
ECs_3286	cysK	cysteine	VFG044254	Staphylof	-	setB	Staphyloco	Nutritiona	sbnA	2,3-diamin	32.3	1.07E-	142

		synthase A	(gb|WP_000570808)	errin B (VF1014)			ccus aureus subsp. aureus str. Newman	l/Metabolic factor		opropionate biosynthesis protein SbnA		39	
ECs_3997	tdcB	L-threonine dehydratase	VFG044286 (gb|WP_012367521)	Proteobactin (VF1252)	-	setB	Proteus mirabilis HI4320	Nutritional/Metabolic factor	PMI_RS01140	cysteine synthase family protein	26.3	5.72E-07	50.4
ECs_4706	ilvA	l-threonine dehydratase	VFG044286 (gb|WP_012367521)	Proteobactin (VF1252)	-	setB	Proteus mirabilis HI4320	Nutritional/Metabolic factor	PMI_RS01140	cysteine synthase family protein	24.9	9.51E-08	53.9
ECs_4298	ugpA	sn-glycerol-3-phosphate ABC transporter permease	VFG030565 (gb|WP_003411940)	Trehalose-recycling ABC transporter (VF0842)	-	setB	Mycobacterium bovis AF2122/97	Nutritional/Metabolic factor	sugA	sugar ABC transporter permease	32.9	9.10E-39	137
ECs_0937	potI	putrescine ABC transporter permease	VFG013568 (gb|WP_005687630)	HitABC (VF0268)	HitABC(fbpABC) operon encodes a periplasmic-binding protein-dependent iron transport system necessary for the utilization of iron bound to transferrin or iron chelates	setA	Haemophilus influenzae 86-028NP	Nutritional/Metabolic factor	hitB	iron ABC transporter permease	28.9	8.60E-10	58.9
ECs_0071	thiP	thiamine	VFG030623	Trehalose	-	setB	Mycobacter	Nutritiona	sugB	carbohydra	26.3	2.26E-	52.4

		transport system permease	(gb|WP_011894705)	-recycling ABC transporter (VF0842)			ium gilvum PYR-GCK	l/Metabolic factor		te ABC transporter permease		07	
ECs_1500	potC	spermidine/putrescine ABC transporter permease	VFG030623 (gb|WP_011894705)	Trehalose-recycling ABC transporter (VF0842)	-	setB	Mycobacterium gilvum PYR-GCK	Nutritional/Metabolic factor	sugB	carbohydrate ABC transporter permease	25.5	1.04E-08	54.7
ECs_3473	ffh	signal recognition particle protein	VFG043311 (gb|WP_011860708)	Peritrichous flagella (VF0732)	-	setA	Clostridium difficile 630	Motility	flhF	flagellar biosynthesis protein FlhF	26.2	2.41E-12	67.8
ECs_2071	narG	nitrate reductase 1 alpha subunit	VFG009599 (gb|WP_011558674)	Nitrate reductase (VF0302)	Nitrate respiration helps the bacteria to survive in O2-depleted areas of inflammatory or necrotic tissue	setA	Mycobacterium sp. KMS	Nutritional/Metabolic factor	narG	nitrate reductase subunit alpha	48.5	0	1192
ECs_1729	narG	nitrate reductase 1 alpha subunit	VFG009599 (gb|WP_011558674)	Nitrate reductase (VF0302)	Nitrate respiration helps the bacteria to survive in O2-depleted areas of inflammatory or necrotic tissue	setA	Mycobacterium sp. KMS	Nutritional/Metabolic factor	narG	nitrate reductase subunit alpha	48.7	0	1199
ECs_3384	ynfF_4	S- and N-oxide	VFG009599 (gb|WP_01	Nitrate reductase	Nitrate respiration helps the bacteria to survive in O2-depleted areas of	setA	Mycobacterium sp. KMS	Nutritional/Metaboli	narG	nitrate reductase	26.6	3.60E-18	89.7

		reductase subunit A	1558674)	(VF0302)	inflammatory or necrotic tissue			c factor		subunit alpha			
ECs_2293	ynfF_2	S- and N-oxide reductase subunit A	VFG009599 (gb|WP_011558674)	Nitrate reductase (VF0302)	Nitrate respiration helps the bacteria to survive in O2-depleted areas of inflammatory or necrotic tissue	setB	Mycobacterium sp. KMS	Nutritional/Metabolic factor	narG	nitrate reductase subunit alpha	29.9	4.45E-06	50.4
ECs_2294	ynfF_3	S- and N-oxide reductase subunit A	VFG009599 (gb|WP_011558674)	Nitrate reductase (VF0302)	Nitrate respiration helps the bacteria to survive in O2-depleted areas of inflammatory or necrotic tissue	setA	Mycobacterium sp. KMS	Nutritional/Metabolic factor	narG	nitrate reductase subunit alpha	25.4	1.37E-12	71.6
ECs_0979	dmsA	anaerobic dimethyl sulfoxide reductase subunit A	VFG009599 (gb|WP_011558674)	Nitrate reductase (VF0302)	Nitrate respiration helps the bacteria to survive in O2-depleted areas of inflammatory or necrotic tissue	setA	Mycobacterium sp. KMS	Nutritional/Metabolic factor	narG	nitrate reductase subunit alpha	28.4	8.80E-07	52.8
ECs_1730	narY	nitrate reductase 1 beta (Fe-S) subunit	VFG024081 (gb|WP_014941434)	Nitrate reductase (VF0302)	Nitrate respiration helps the bacteria to survive in O2-depleted areas of inflammatory or necrotic tissue	setA	Mycobacterium indicus pranii MTCC 9506	Nutritional/Metabolic factor	narH	nitrate reductase subunit beta	57	2.22E-203	575
ECs_2073	espR1	T3SS effector-like protein EspR	VFG034815 (gb|WP_000671689)	TTSS secreted effectors (VF1110)	Cif (Deamidase. Induces cytopathic effects of actin stress fiber formation and cell cycle arrest.); EspB (Pore formation, actin disruption, microvilli effacement, anti-phagocytosis.); EspF (Inducing	setA	Escherichia coli O157:H7 str. EDL933	Effector delivery system	espR1	Type III secretion system effector espR1	100	2.39E-258	706

degradation of the aniapoptic protein AbcF2, tight junction disruption, microvilli effacement and elongation, mitochondrial dysfunction, N-WASP activation, SGLT-1 inactivation, pedestal maturation, inhibition of NHE3 activity, membrane remodelling; targets and disrupts the nucleolus late in infection, which is temporally controlled by host mitochondria.); EspFu/tccP (Inducing degradation of the aniapoptic protein AbcF2, tight junction disruption, microvilli effacement and elongation, mitochondrial dysfunction, N-WASP activation, SGLT-1 inactivation, pedestal maturation, inhibition of NHE3 activity, membrane remodelling; targets and disrupts the nucleolus late in infection, which is temporally controlled by host mitochondria.); EspG (TBC-like GTPase activating protein. Efficiently catalyzes GTP hydrolysis in Rab1 to disrupt of Rab1-mediated ER-to-Golgi trafficking.); EspH (First bacterial

effector acting directly on RhoGEFs, EspH directly binds to the DH-PH domain in RhoGEFs to disrupt RhoGEF-Rho signaling; critical for inhibiting macrophage phagocytosis.); EspJ (Inhibit both IgG- and complement receptor-mediated phagocytosis.); EspK; EspL1; EspL2 (Cysteine protease. Bounds F-actin-aggregating annexin 2 directly to increase annexin 2's ability to aggregate Tir-induced F-actin; block necroptosis and in flammation.); EspL4; EspM1 (GEF. Activates the RhoA signaling pathway and induce the formation of stress fibres; inhibit pedestal formation and induce tight junction mislocalization.); EspM2 (GEF. Activates the RhoA signaling pathway and induce the formation of stress fibres; inhibit pedestal formation and induce tight junction mislocalization.); EspN; EspO1-1; EspO1-2; EspR1; EspR3; EspR4; EspT (GEF. Activates Rac1 and Cdc42 leading to formation of membrane ruffles and

lamellipodia; induces membrane ruffles to facilitate bacterial invasion into non-phagocytic cells in a process involving Rac1 and Wave2.); EspW; EspX1; EspX2; EspX4; EspX5; EspX6; EspX7/nleL (E3 ubiquitin ligase, HECT-like. Modulates pedestal formation.); EspY1; EspY2; EspY3; EspY4; EspY5; Map (GEF. Mimics the host Dbl and catalyses the exchange of GDP for GTP in Cdc42, involved in effacement, SGLT1 inhibition, formation of filopodia and disruption of mitochondrial function.); NleA/espI (Disruption of tight junctions by inhibition of host cell protein trafficking through COPII-dependent pathways.); NleB1 (Blocks translocation of the p65 and to the host cell nucleus to inhibit NF-<kappa>B pathway, but NleE and NleB act at different points in the NF-<kappa>B signaling pathway.); NleB2 (May also have anti-inflammatory activity.); NleC (Metalloprotease. Zn-dependent endopeptidases that

specifically clip and inactivate RelA (p65), thus blocking NF-<kappa>B pathway.); NleD (Metalloprotease. Zn-dependent endopeptidases that specifically clip and inactivate JNK and p38, thus blocking AP-1 pathway.); NleE (PMN tran-epithelial migration; blocks translocation of the p65 to the host cell nucleus by preventing I<kappa>B degradation to inhibit NF-<kappa>B pathway.); NleF; NleG-1; NleG2-2; NleG2-3; NleG2-4; NleG5-1; NleG5-2; NleG6-1; NleG6-2; NleG6-3; NleG7 (U-box type E3 ubiquitin ligases.); NleG8-2; NleH1 (Ser/Thr protein kinase. Binds directly to a subunit of NF-<kappa>B, the ribosomal protein S3 (RPS3), reducing the nuclear abundance of RPS3 to dampen host transcriptional outputs; interact with Bax inhibitor-1 to block apoptosis.); NleH2 (Putative kinase. Attenuates NF-<kappa>B pathway.); SepZ/espZ (EspZ interacts with CD98 in host cell membranes to promote host cell survival, therefore provide the

					pathogen with valuable time to colonize efficiently prior to dissemination.); TccP2; Tir (Mimics host immunoreceptor tyrosine-based inhibition motifs (ITIMs), also see helicobacter CagA. EHEC Tir lacks the Nck binding site. Conserved NPY (Asn-Pro-Tyr) motif recruits the adaptor protein IRTKS and/or IRSp53. IRTKS/IRSp53 link Tir and TccP/EspFu, which in turn activates N-WASP; Receptor for intimin; effacement; SGLT1 inhibition; recruits SHIP2 to control actin-pedestal morphology; maintains the integrity of the epithelium by keeping the destructive activity of EspG and EspG2 in check.)								
ECs_2072	narU	nitrate/nitrite transporter	VFG022816 (gb|YP_001704253)	Nitrate/nitrite transporter (VF0821)	-	setB	Mycobacterium abscessus ATCC 19977	Nutritional/Metabolic factor	narK2	Probable integral membrane nitrite extrusion protein NarK3	34.2	7.13E-73	237
ECs_1728	narU	nitrate/nitrite	VFG022816 (gb|YP_00	Nitrate/nitrite	-	setB	Mycobacterium	Nutritional/Metaboli	narK2	Probable integral	35.1	7.61E-81	258

		transporter	1704253)	transporter (VF0821)			abscessus ATCC 19977	c factor		membrane nitrite extrusion protein NarK3			
ECs_2076	ECs_2076	outer membrane porin protein	VFG043568 (gb|NP_460531)	OmpD (VF0969)	-	setA	Salmonella enterica subsp. enterica serovar Typhimurium str. LT2	Adherence	nmpC	phosphoporin PhoE	72	1.12E-196	545
ECs_0268	phoE	outer membrane phosphoporin protein E	VFG043568 (gb|NP_460531)	OmpD (VF0969)	-	setA	Salmonella enterica subsp. enterica serovar Typhimurium str. LT2	Adherence	nmpC	phosphoporin PhoE	61.7	5.90E-155	439
ECs_3104	ompC	outer membrane porin OmpC	VFG043568 (gb|NP_460531)	OmpD (VF0969)	-	setA	Salmonella enterica subsp. enterica serovar Typhimurium str. LT2	Adherence	nmpC	phosphoporin PhoE	63.1	1.93E-162	459

ECs_1012	ompF	outer membrane porin OmpF	VFG043568 (gb|NP_460531)	OmpD (VF0969)	-	setA	Salmonella enterica subsp. enterica serovar Typhimurium str. LT2	Adherence	nmpC	phosphoporin PhoE	62	1.30E-149	426
ECs_0481	yajR	transporter	VFG044123 (gb|WP_010930940)	Alcaligin (VF0688)	-	setA	Bordetella pertussis Tohama I	Nutritional/Metabolic factor	bcr	alcaligin siderophore export MFS transporter Bcr	26.4	1.95E-12	68.6
ECs_4310	rpoH	RNA polymerase sigma 32 factor RpoH	VFG043648 (gb|NP_252312)	Type IV pili (VF0082)	Attaches to host cells, but not to mucin, causing a twitching motility that allows the bacteria to move along the cell surface; biofilm formation	setA	Pseudomonas aeruginosa PAO1	Adherence	rpoS	RNA polymerase sigma factor RpoS	30.2	2.93E-22	94.4
ECs_3595	rpoS	RNA polymerase sigma S factor RpoS	VFG043648 (gb|NP_252312)	Type IV pili (VF0082)	Attaches to host cells, but not to mucin, causing a twitching motility that allows the bacteria to move along the cell surface; biofilm formation	setA	Pseudomonas aeruginosa PAO1	Adherence	rpoS	RNA polymerase sigma factor RpoS	76.4	3.84E-141	402
ECs_3670	csdA	cysteine sulfinate desulfina	VFG043141 (gb|WP_005478185)	Lateral flagella (VF0625)	-	setB	Vibrio parahaemolyticus RIMD	Biofilm	VP_RS22400	aminotransferase class	26.9	1.51E-07	53.1

		se					2210633			V-fold PLP-dependent enzyme			
ECs_2387	sufS	cysteine desulfurase	VFG043141 (gb|WP_005478185)	Lateral flagella (VF0625)	-	setB	Vibrio parahaemolyticus RIMD 2210633	Biofilm	VP_RS22400	aminotransferase class V-fold PLP-dependent enzyme	31.7	1.01E-15	78.6
ECs_3396	iscS	cysteine desulfurase	VFG043141 (gb|WP_005478185)	Lateral flagella (VF0625)	-	setB	Vibrio parahaemolyticus RIMD 2210633	Biofilm	VP_RS22400	aminotransferase class V-fold PLP-dependent enzyme	29.9	1.53E-07	53.1
ECs_3672	tcdA	tRNA threonylcarbamoyladenosine dehydratase	VFG044389 (gb|AAF33130)	Pyridine-2,6-dithiocarboxylic acid (PDTC) (VF0938)	-	setB	Pseudomonas stutzeri KC	Nutritional/Metabolic factor	pdtorfF	putative sulfurylase	30.1	3.03E-06	47.8
ECs_4915	thiF	adenylyltransferase	VFG044389 (gb|AAF33130)	Pyridine-2,6-dithiocarboxylic acid (PDTC)	-	setB	Pseudomonas stutzeri KC	Nutritional/Metabolic factor	pdtorfF	putative sulfurylase	38.3	2.36E-43	150

				(VF0938)									
ECs_0904	moeB	molybdopterin synthase sulfurylase	VFG044389 (gb|AAF33130)	Pyridine-2,6-dithiocarboxylic acid (PDTC) (VF0938)	-	setB	Pseudomonas stutzeri KC	Nutritional/Metabolic factor	pdtorfF	putative sulfurylase	38.9	6.75E-51	170
ECs_3529	ygaP	membrane protein	VFG044378 (gb|AAL65285)	Thioquinolobactin (VF0937)	-	setB	Pseudomonas fluorescens ATCC 17400	Nutritional/Metabolic factor	qbsC	QbsC	31.2	8.83E-06	44.7
ECs_4328	ECs_4328	acyl carrier protein	VFG005769 (gb|WP_000611493)	<beta>-haemolysin/cytolysin (VF0279)	Forms pores in cell membrane; proinflammatory effects: inducing apoptosis, promoting cellular invasion, triggering iNOS and cytokine release	setA	Streptococcus agalactiae NEM316	Exotoxin	acpC	acyl carrier protein AcpC	43.2	2.14E-09	49.7
ECs_1287	ECs_1287	acyl carrier protein	VFG005769 (gb|WP_000611493)	<beta>-haemolysin/cytolysin (VF0279)	Forms pores in cell membrane; proinflammatory effects: inducing apoptosis, promoting cellular invasion, triggering iNOS and cytokine release	setA	Streptococcus agalactiae NEM316	Exotoxin	acpC	acyl carrier protein AcpC	38.4	7.85E-12	56.2
ECs_2652	uvrY	two-component regulatory system	VFG015080 (gb|NP_251276)	GacS/GacA two-component system	-	setA	Pseudomonas aeruginosa PAO1	Regulation	gacA	response regulator GacA	59.9	8.07E-86	253

		response regulator UvrY		(VF0908)									
ECs_2654	sdiA	quorum-sensing transcriptional activator	VFG000152 (gb|NP_252167)	Quorum sensing (VF0093)	Both the las and rhl systems regulate the production of multiple virulence factors; crucial for proper biofilm formation; 3O-C12-HSL also directly acts as a virulence factor:; activates extracellular-signal-regulated kinases(ERKs) that subsequently induced the activation of the transcription factor NF-<kappa>B. This activation of NF-<kappa>B was essential for maximal production of IL-8; a potent inducer of IFN-<gamma> production in T cells; upregulate the expression of cyclooxygenase-2(Cox-2), which results in the increased production of PGE2. PGE2 has many functions, one of which is the induction of endothelial permeability. This vasorelaxant activity might lead to increased local blood flow, edema and cellular migration to the site of infection	setA	Pseudomonas aeruginosa PAO1	Biofilm	rhlR	transcriptional regulator RhlR	40.8	9.90E-56	178

ECs_3703	yqeH	LuxR family transcriptional regulator	VFG034392 (gb|WP_001323479)	ECP (VF0404)	ECP, composed of a 21-kDa pilin subunit EspA, is a pilus-adherence factor that is crucial to the virulence of E. coli O157 in humans, and is also carried by commensal strains of E. coli.; It is suggested that pathogenic E. coli strains may use ECP to mimic commensal E. coli and provide themselves with an ecological advantage for host colonization and evasion of the immune system.	setA	Escherichia coli E24377A	Adherence	ykgK/ecpR	regulator protein EcpR	26.2	1.78E-13	65.9
ECs_0324	ecpR	transcriptional regulator for the ecp operon	VFG034378 (gb|WP_001301675)	ECP (VF0404)	ECP, composed of a 21-kDa pilin subunit EspA, is a pilus-adherence factor that is crucial to the virulence of E. coli O157 in humans, and is also carried by commensal strains of E. coli.; It is suggested that pathogenic E. coli strains may use ECP to mimic commensal E. coli and provide themselves with an ecological advantage for host colonization and evasion of the immune system.	setA	Escherichia coli O157:H7 str. TW14359	Adherence	ykgK/ecpR	regulator protein EcpR	99.4	6.06E-128	356
ECs_5184	ytfB	OapA family protein	VFG013213 (gb|WP_010944629)	OapA (VF0040)	Required for efficient colonization of the nasopharynx, may play a minor role in adherence	setB	Haemophilus ducreyi 35000HP	Adherence	oapA	opacity associated protein (oapA)	35	1.60E-06	47.8

ECs_2841	arnB	uridine 5'-(beta-1-threo-pentapyranosyl-4-ulose diphosphate) aminotransferase	VFG002226 (gb|WP_002963679)	LPS (VF0367)	Plays a role in entry and early survival inside macrophages; Resistance to innate-immunity anti-bacterial responses; a modulator of the immune response	setA	Brucella melitensis bv. 1 str. 16M	Immune modulation	per	perosamine synthetase	52.6	8.10E-138	396
ECs_1283	ECs_1283	hemolysin activator-related protein	VFG042362 (gb|AAC31980)	HecA-HecB (VF1273)	-	setA	Erwinia chrysanthemi EC16	Effector delivery system	hecB	HecB	29	8.15E-53	188
ECs_1382	ECs_1382	HecB-like protein	VFG042362 (gb|AAC31980)	HecA-HecB (VF1273)	-	setA	Erwinia chrysanthemi EC16	Effector delivery system	hecB	HecB	28.7	1.29E-13	71.6
ECs_1282	ECs_1282	hemagglutinin/hemolysin-related protein	VFG049838 (gb|WP_011335122)	Exolysin (VF0582)	ExlA has a pore-forming activity responsible for host cell membrane disruption.; ExlA requires the outer membrane protein ExlB for export into extracellular medium.	setA	Pseudomonas fluorescens Pf0-1	Effector delivery system	exlA	hemolysin	39.4	5.03E-37	152
ECs_1280	ECs_1280	major pilin protein	VFG042693 (gb|NP_250818)	CupA fimbriae (VF0924)	-	setB	Pseudomonas aeruginosa PAO1	Biofilm	cupA1	fimbrial subunit CupA1	40.9	2.21E-28	104

ECs_2602	flhD	flagellar class II regulon transcriptional activator	VFG043098 (gb|WP_001302091)	Peritrichous flagella (VF1154)	-	setA	Escherichia coli O157:H7 str. EDL933	Motility	flhD	flagellar transcriptional regulator FlhD	100	2.05E-77	223
ECs_1289	ECs_1289	3-oxoacyl-[acyl-carrier-protein] synthase	VFG005786 (gb|WP_000118223)	<beta>-haemolysin/cytolysin (VF0279)	Forms pores in cell membrane; proinflammatory effects: inducing apoptosis, promoting cellular invasion, triggering iNOS and cytokine release	setA	Streptococcus agalactiae A909	Exotoxin	cylI	putative 3-ketoacyl-ACP synthase CylI	25.5	2.07E-44	171
ECs_4338	fabF	synthase 3-oxoacyl-(acyl-carrier-protein) synthase II	VFG005786 (gb|WP_000118223)	<beta>-haemolysin/cytolysin (VF0279)	Forms pores in cell membrane; proinflammatory effects: inducing apoptosis, promoting cellular invasion, triggering iNOS and cytokine release	setA	Streptococcus agalactiae A909	Exotoxin	cylI	putative 3-ketoacyl-ACP synthase CylI	30.9	5.15E-45	165
ECs_4341	fabF	3-oxoacyl-(acyl-carrier-protein) synthase II	VFG022573 (gb|WP_013828959)	FAS-II (VF0809)	-	setA	Mycobacterium sp. JDM601	Nutritional/Metabolic factor	kasB	beta-ketoacyl-ACP synthase	32.2	7.28E-52	179
ECs_1473	fabF_2	3-oxoacyl-[acyl-ca	VFG022573 (gb|WP_01	FAS-II (VF0809)	-	setA	Mycobacterium sp.	Nutritional/Metaboli	kasB	beta-ketoacyl-ACP	38.9	4.51E-81	255

		rrier-protein] synthase II	3828959)				JDM601	c factor		synthase			
ECs_3207	fabB	3-oxoacyl-[acyl-carrier-protein] synthase I	VFG009141 (gb|WP_015307816)	FAS-II (VF0809)	-	setA	Mycobacterium smegmatis JS623	Nutritional/Metabolic factor	kasB	beta-ketoacyl-ACP synthase	33.8	5.16E-52	179
ECs_1288	ECs_1288	aminomethyltransferase	VFG005784 (gb|WP_001092618)	<beta>-haemolysin/cytolysin (VF0279)	Forms pores in cell membrane; proinflammatory effects: inducing apoptosis, promoting cellular invasion, triggering iNOS and cytokine release	setA	Streptococcus agalactiae NEM316	Exotoxin	cylF	putative aminomethyltransferase CylF	20.9	5.36E-06	47.8
ECs_3776	gcvT	aminomethyltransferase	VFG005784 (gb|WP_001092618)	<beta>-haemolysin/cytolysin (VF0279)	Forms pores in cell membrane; proinflammatory effects: inducing apoptosis, promoting cellular invasion, triggering iNOS and cytokine release	setA	Streptococcus agalactiae NEM316	Exotoxin	cylF	putative aminomethyltransferase CylF	23.6	1.15E-06	49.7
ECs_2846	wcaN	UTP--glucose-1-phosphate uridylyltransferase	VFG048990 (gb|WP_001741945)	Capsule (VF0560)	Assisting in evading the host immune system by protecting bacteria from opsonophagocytosis and serum killing	setA	Klebsiella pneumoniae subsp. pneumoniae NTUH-K2044	Immune modulation	galF	GalU regulator GalF	89.5	3.45E-194	533

ECs_2847	gne	UDP-galactose 4-epimerase	VFG019018 (gb|WP_192575220)	O-antigen (VF0392)	LPS O antigen mutants were severely impaired in their ability to colonize the Peyer's patches and did not colonize spleen and liver. The absence of O antigen in the outer membrane affects the expression of other Yersinia virulence factors.	setA	Yersinia pseudotuberculosis YPIII	Immune modulation	YPK_RS15930	NAD-dependent epimerase/dehydratase family protein	49.2	1.38E-101	301
ECs_0955	ybjT	NAD-dependent oxidoreductase	VFG014133 (gb|WP_012075037)	LPS (VF0085)	Mediates biological effects including resistance to serum killing and phagocytosis; the binding to normal CFTR (cystic fibrosis transmembrane conductance regulator) and invasion of host cells may make a contribution to virulence in the human eye; internalization by binding to normal CFTR protein expressed by airway epithelial cells followed by desquamation of bacteria-laden epithelial cells, constitutes a host defense mechanism. If this mechanism fails to function properly, abnormally high bacterial carriage would promote the establishment of chronic bacterial infection	setA	Pseudomonas aeruginosa PA7	Immune modulation	PSPA7_RS09485	SDR family oxidoreductase	24.7	4.20E-11	63.9
ECs_1120	sodC_1	copper/zinc-supero	VFG000463 (gb|NP_46	SodCI (VF0109)	Contribute to survival during systemic phase of infection	setA	Salmonella enterica	Stress survival	sodCI	Gifsy-2 prophage:	58.6	4.11E-64	194

		xide dismutase	0019)				subsp. enterica serovar Typhimurium str. LT2			superoxide dismutase precursor (Cu-Zn)			
ECs_2355	sodC	Cu-Zn family superoxide dismutase	VFG000463 (gb|NP_460019)	SodCI (VF0109)	Contribute to survival during systemic phase of infection	setA	Salmonella enterica subsp. enterica serovar Typhimurium str. LT2	Stress survival	sodCI	Gifsy-2 prophage: superoxide dismutase precursor (Cu-Zn)	58.3	7.38E-57	176
ECs_1989	ECs_1989	copper/zinc-superoxide dismutase	VFG000463 (gb|NP_460019)	SodCI (VF0109)	Contribute to survival during systemic phase of infection	setA	Salmonella enterica subsp. enterica serovar Typhimurium str. LT2	Stress survival	sodCI	Gifsy-2 prophage: superoxide dismutase precursor (Cu-Zn)	59.3	1.76E-65	198
ECs_5062	mdtP	outer membrane factor of efflux pump	VFG034674 (gb|WP_000074221)	Ibes (VF0237)	Contributes to brain microvascular endothelial cells (BMECs) invasion via a ligand-receptor interaction	setA	Escherichia coli NA114	Invasion	ibeB	Cu(+)/Ag(+) efflux RND transporter outer membrane channel	27.5	1.62E-38	145

ECs_0610	cusC	outer membrane component of copper/silver efflux system	VFG034670 (gb|WP_000074194)	Ibes (VF0237)	Contributes to brain microvascular endothelial cells (BMECs) invasion via a ligand-receptor interaction	setA	Escherichia coli 044:H18 042	Invasion	ibeB	CusC Cu(+)/Ag(+) efflux RND transporter outer membrane channel CusC	98.9	3.1400000228e-316	856
ECs_1865	eefC	outer membrane channel protein	VFG034652 (gb|WP_000074204)	Ibes (VF0237)	Contributes to brain microvascular endothelial cells (BMECs) invasion via a ligand-receptor interaction	setA	Escherichia coli 0157:H7 str. EDL933	Invasion	ibeB	Cu(+)/Ag(+) efflux RND transporter outer membrane channel CusC	36.1	3.34E-74	240
ECs_1915	fnr	fumarate/nitrate reduction transcriptional regulator	VFG042734 (gb|NP_249343)	Type IV pili (VF0082)	Attaches to host cells, but not to mucin, causing a twitching motility that allows the bacteria to move along the cell surface; biofilm formation	setA	Pseudomonas aeruginosa PAO1	Adherence	vfr	cAMP-regulatory protein	23.7	2.18E-07	50.1
ECs_4208	crp	cAMP-activated global	VFG042734 (gb|NP_249343)	Type IV pili (VF0082)	Attaches to host cells, but not to mucin, causing a twitching motility that allows the bacteria to move along	setA	Pseudomonas aeruginosa	Adherence	vfr	cAMP-regulatory protein	66.8	1.82E-94	274

		transcription factor			the cell surface; biofilm formation		PAO1						
ECs_0533	ushA	UDP-sugar hydrolase	VFG002420 (gb|WP_000645781)	AdsA (VF0422)	Evasion from innate immunity	setA	Staphylococcus aureus subsp. aureus MW2	Immune modulation	adsA	Adenosine synthase A	28.3	3.37E-43	164
ECs_5191	cpdB	2':3'-cyclic-nucleotide 2'-phosphodiesterase	VFG002420 (gb|WP_000645781)	AdsA (VF0422)	Evasion from innate immunity	setA	Staphylococcus aureus subsp. aureus MW2	Immune modulation	adsA	Adenosine synthase A	21.7	9.44E-12	68.2
ECs_0531	ybaL	inner membrane NAD(P)-binding transporter	VFG030136 (gb|WP_003902432)	Potassium/proton antiporter (VF0838)	-	setB	Mycobacterium tuberculosis CCDC5079	Immune modulation	kefB	cation:proton antiporter	29.4	4.61E-16	80.1
ECs_0050	kefC	glutathione-regulated potassium-efflux system protein	VFG030136 (gb|WP_003902432)	Potassium/proton antiporter (VF0838)	-	setB	Mycobacterium tuberculosis CCDC5079	Immune modulation	kefB	cation:proton antiporter	28.5	5.69E-06	48.9

ECs_4201	kefB	glutathione-regulated potassium-efflux system protein	VFG030136 (gb|WP_003902432)	Potassium/proton antiporter (VF0838)	-	setB	Mycobacterium tuberculosis CCDC5079	Immune modulation	kefB	cation:proton antiporter	26.2	1.14E-09	60.5
ECs_0536	ybaQ	transcriptional regulator	VFG016797 (gb|WP_011180278)	Virulence-associated proteins (VF0679)	-	setB	Bartonella henselae str. Houston-1	Others	vapA1	HigA family addiction module antidote protein	44.9	8.62E-15	65.1
ECs_2081	yddM	transcriptional regulator	VFG016799 (gb|WP_011180601)	Virulence-associated proteins (VF0679)	-	setB	Bartonella henselae str. Houston-1	Others	vapA3	HigA family addiction module antidote protein	27.7	8.58E-08	45.8
ECs_4728	wecG	UDP-N-acetyl-D-mannosaminuronic acid transferase	VFG007687 (gb|WP_001245629)	Capsular polysaccharide (VF0624)	-	setB	Vibrio cholerae O395	Immune modulation	cpsF	exopolysaccharide biosynthesis glycosyltransferase VpsK	39.4	5.07E-34	122
ECs_4555	espD	T3SS transloca	VFG000799 (gb|WP_00	TTSS (VF0191)	Injects Tir and other effector molecules directly into the host cell.	setA	Escherichia coli	Effector delivery	espD	Type III secretion	100	6.85E-233	638

		tor EspD	0935759)		Effector molecules activate cell-signaling pathways, causing alterations in the host cell cytoskeleton and resulting in the depolymerization of actin and the loss of microvilli		O157:H7 str. EDL933	system		system translocator protein EspD, pore protein			
ECs_0883	fiu	catecholate siderophore receptor	VFG044178 (gb|WP_005477483)	Vibrioferrin (VF0631)	-	setA	Vibrio parahaemolyticus RIMD 2210633	Nutritional/Metabolic factor	VP_RS23090	TonB-dependent siderophore receptor	26.4	4.97E-45	172
ECs_0154	fhuA	ferrichrome outer membrane transporter	VFG044178 (gb|WP_005477483)	Vibrioferrin (VF0631)	-	setA	Vibrio parahaemolyticus RIMD 2210633	Nutritional/Metabolic factor	VP_RS23090	TonB-dependent siderophore receptor	26	4.70E-41	160
ECs_3917	fitA	ferrichrome iron receptor precursor	VFG044178 (gb|WP_005477483)	Vibrioferrin (VF0631)	-	setA	Vibrio parahaemolyticus RIMD 2210633	Nutritional/Metabolic factor	VP_RS23090	TonB-dependent siderophore receptor	27.1	1.10E-36	146
ECs_0661	pagP	phospholipid:lipid A palmitoyltransferase	VFG011228 (gb|WP_082011711)	LPS-modifying enzyme (VF0684)	-	setB	Bordetella avium 197N	Immune modulation	pagP	lipid IV(A) palmitoyltransferase PagP	45.6	1.05E-40	136

ECs_4004	garL	alpha-dehydro-beta-deoxy-D-glucarate aldolase	VFG044353 (gb|WP_013317296)	Achromobactin (VF1243)	-	setB	Dickeya dadantii 3937	Nutritional/Metabolic factor	DDA3937_RS07740	siderophore biosynthesis protein SbnG	32.9	3.53E-36	129
ECs_3130	rhmA	2-keto-3-deoxy-L-rhamnonate aldolase	VFG044353 (gb|WP_013317296)	Achromobactin (VF1243)	-	setB	Dickeya dadantii 3937	Nutritional/Metabolic factor	DDA3937_RS07740	siderophore biosynthesis protein SbnG	30.5	2.11E-25	100
ECs_2143	ftrA	transcriptional regulator	VFG041884 (gb|WP_080509009)	Cpi-2 encoded T3SS (SPI-2 like) (VF1260)	-	setB	Chromobacterium violaceum ATCC 12472	Effector delivery system	CV_RS12640	AraC family transcriptional regulator	28.9	1.33E-08	55.1
ECs_0394	cynX	cyanate transporter	VFG044371 (gb|AAL65278)	Thioquinolobactin (VF0937)	-	setB	Pseudomonas fluorescens ATCC 17400	Nutritional/Metabolic factor	qbsM	QbsM	33.9	8.70E-43	154
ECs_2500	yeaN	MFS transporter	VFG044371 (gb|AAL65278)	Thioquinolobactin (VF0937)	-	setB	Pseudomonas fluorescens ATCC 17400	Nutritional/Metabolic factor	qbsM	QbsM	31.9	2.60E-50	174

ECs_4250	feoA	ferrous iron transporter	VFG045722 (gb|WP_003633215)	FeoAB (VF0160)	A feoB mutant of L. pneumophila has a lowered Fe2+ uptake and is attenuated for intracellular growth	setA	Legionella longbeachae NSW150	Nutritional/Metabolic factor	feoA	ferrous iron transporter A	53.8	4.62E-12	55.5
ECs_1814	nleH1_2	T3SS secreted effector NleH	VFG035073 (gb|WP_000950979)	TTSS secreted effectors (VF1110)	Cif (Deamidase. Induces cytopathic effects of actin stress fiber formation and cell cycle arrest.); EspB (Pore formation, actin disruption, microvilli effacement, anti-phagocytosis.); EspF (Inducing degradation of the aniapoptic protein AbcF2, tight junction disruption, microvilli effacement and elongation, mitochondrial dysfunction, N-WASP activation, SGLT-1 inactivation, pedestal maturation, inhibition of NHE3 activity, membrane remodelling; targets and disrupts the nucleolus late in infection, which is temporally controlled by host mitochondria.); EspFu/tccP (Inducing degradation of the aniapoptic protein AbcF2, tight junction disruption, microvilli effacement and elongation, mitochondrial dysfunction, N-WASP activation, SGLT-1 inactivation,	setA	Escherichia coli O157:H7 str. EDL933	Effector delivery system	nleH2	Type III secretion system effector NleH2	100	6.05E-205	561

pedestal maturation, inhibition of NHE3 activity, membrane remodelling; targets and disrupts the nucleolus late in infection, which is temporally controlled by host mitochondria.); EspG (TBC-like GTPase activating protein. Efficiently catalyzes GTP hydrolysis in Rab1 to disrupt of Rab1-mediated ER-to-Golgi trafficking.); EspH (First bacterial effector acting directly on RhoGEFs, EspH directly binds to the DH-PH domain in RhoGEFs to disrupt RhoGEF-Rho signaling; critical for inhibiting macrophage phagocytosis.); EspJ (Inhibit both IgG- and complement receptor-mediated phagocytosis.); EspK; EspL1; EspL2 (Cysteine protease. Bounds F-actin-aggregating annexin 2 directly to increase annexin 2's ability to aggregate Tir-induced F-actin; block necroptosis and in flammation.); EspL4; EspM1 (GEF. Activates the RhoA signaling pathway and induce the formation of stress fibres; inhibit pedestal formation and

induce tight junction mislocalization.); EspM2 (GEF. Activates the RhoA signaling pathway and induce the formation of stress fibres; inhibit pedestal formation and induce tight junction mislocalization.); EspN; EspO1-1; EspO1-2; EspR1; EspR3; EspR4; EspT (GEF. Activates Rac1 and Cdc42 leading to formation of membrane ruffles and lamellipodia; induces membrane ruffles to facilitate bacterial invasion into non-phagocytic cells in a process involving Rac1 and Wave2.); EspW; EspX1; EspX2; EspX4; EspX5; EspX6; EspX7/nleL (E3 ubiquitin ligase, HECT-like. Modulates pedestal formation.); EspY1; EspY2; EspY3; EspY4; EspY5; Map (GEF. Mimics the host Dbl and catalyses the exchange of GDP for GTP in Cdc42, involved in effacement, SGLT1 inhibition, formation of filopodia and disruption of mitochondrial function.); NleA/espI (Disruption of tight junctions by inhibition of host cell

protein trafficking through COPII-dependent pathways.); NleB1 (Blocks translocation of the p65 and to the host cell nucleus to inhibit NF-<kappa>B pathway, but NleE and NleB act at different points in the NF-<kappa>B signaling pathway.); NleB2 (May also have anti-inflammatory activity.); NleC (Metalloprotease. Zn-dependent endopeptidases that specifically clip and inactivate RelA (p65), thus blocking NF-<kappa>B pathway.); NleD (Metalloprotease. Zn-dependent endopeptidases that specifically clip and inactivate JNK and p38, thus blocking AP-1 pathway.); NleE (PMN tran-epithelial migration; blocks translocation of the p65 to the host cell nucleus by preventing I<kappa>B degradation to inhibit NF-<kappa>B pathway.); NleF; NleG-1; NleG2-2; NleG2-3; NleG2-4; NleG5-1; NleG5-2; NleG6-1; NleG6-2; NleG6-3; NleG7 (U-box type E3 ubiquitin ligases.); NleG8-2; NleH1 (Ser/Thr protein kinase. Binds directly to a

subunit of NF-<kappa>B, the ribosomal protein S3 (RPS3), reducing the nuclear abundance of RPS3 to dampen host transcriptional outputs; interact with Bax inhibitor-1 to block apoptosis.); NleH2 (Putative kinase. Attenuates NF-<kappa>B pathway.); SepZ/espZ (EspZ interacts with CD98 in host cell membranes to promote host cell survival, therefore provide the pathogen with valuable time to colonize efficiently prior to dissemination.); TccP2; Tir (Mimics host immunoreceptor tyrosine-based inhibition motifs (ITIMs), also see helicobacter CagA. EHEC Tir lacks the Nck binding site. Conserved NPY (Asn-Pro-Tyr) motif recruits the adaptor protein IRTKS and/or IRSp53. IRTKS/IRSp53 link Tir and TccP/EspFu, which in turn activates N-WASP; Receptor for intimin; effacement; SGLT1 inhibition; recruits SHIP2 to control actin-pedestal morphology; maintains the integrity of the epithelium by keeping the destructive

					activity of EspG and EspG2 in check.)								
ECs_0848	nleH1-1	T3SS secreted effector NleH	VFG035070 (gb|WP_000950986)	TTSS secreted effectors (VF1110)	Cif (Deamidase. Induces cytopathic effects of actin stress fiber formation and cell cycle arrest.); EspB (Pore formation, actin disruption, microvilli effacement, anti-phagocytosis.); EspF (Inducing degradation of the aniapoptic protein AbcF2, tight junction disruption, microvilli effacement and elongation, mitochondrial dysfunction, N-WASP activation, SGLT-1 inactivation, pedestal maturation, inhibition of NHE3 activity, membrane remodelling; targets and disrupts the nucleolus late in infection, which is temporally controlled by host mitochondria.); EspFu/tccP (Inducing degradation of the aniapoptic protein AbcF2, tight junction disruption, microvilli effacement and elongation, mitochondrial dysfunction, N-WASP activation, SGLT-1 inactivation, pedestal maturation, inhibition of NHE3 activity, membrane remodelling; targets and disrupts the nucleolus	setA	Escherichia coli O103:H2 str. 12009	Effector delivery system	nleH1	Type III secretion system effector NleH1	92.5	3.20E-195	536

late in infection, which is temporally controlled by host mitochondria.); EspG (TBC-like GTPase activating protein. Efficiently catalyzes GTP hydrolysis in Rab1 to disrupt of Rab1-mediated ER-to-Golgi trafficking.); EspH (First bacterial effector acting directly on RhoGEFs, EspH directly binds to the DH-PH domain in RhoGEFs to disrupt RhoGEF-Rho signaling; critical for inhibiting macrophage phagocytosis.); EspJ (Inhibit both IgG- and complement receptor-mediated phagocytosis.); EspK; EspL1; EspL2 (Cysteine protease. Bounds F-actin-aggregating annexin 2 directly to increase annexin 2's ability to aggregate Tir-induced F-actin; block necroptosis and in flammation.); EspL4; EspM1 (GEF. Activates the RhoA signaling pathway and induce the formation of stress fibres; inhibit pedestal formation and induce tight junction mislocalization.); EspM2 (GEF. Activates the RhoA signaling pathway

and induce the formation of stress fibres; inhibit pedestal formation and induce tight junction mislocalization.); EspN; EspO1-1; EspO1-2; EspR1; EspR3; EspR4; EspT (GEF. Activates Rac1 and Cdc42 leading to formation of membrane ruffles and lamellipodia; induces membrane ruffles to facilitate bacterial invasion into non-phagocytic cells in a process involving Rac1 and Wave2.); EspW; EspX1; EspX2; EspX4; EspX5; EspX6; EspX7/nleL (E3 ubiquitin ligase, HECT-like. Modulates pedestal formation.); EspY1; EspY2; EspY3; EspY4; EspY5; Map (GEF. Mimics the host Dbl and catalyses the exchange of GDP for GTP in Cdc42, involved in effacement, SGLT1 inhibition, formation of filopodia and disruption of mitochondrial function.); NleA/espI (Disruption of tight junctions by inhibition of host cell protein trafficking through COPII-dependent pathways.); NleB1 (Blocks translocation of the p65 and to

the host cell nucleus to inhibit NF-<kappa>B pathway, but NleE and NleB act at different points in the NF-<kappa>B signaling pathway.); NleB2 (May also have anti-inflammatory activity.); NleC (Metalloprotease. Zn-dependent endopeptidases that specifically clip and inactivate RelA (p65), thus blocking NF-<kappa>B pathway.); NleD (Metalloprotease. Zn-dependent endopeptidases that specifically clip and inactivate JNK and p38, thus blocking AP-1 pathway.); NleE (PMN tran-epithelial migration; blocks translocation of the p65 to the host cell nucleus by preventing I<kappa>B degradation to inhibit NF-<kappa>B pathway.); NleF; NleG-1; NleG2-2; NleG2-3; NleG2-4; NleG5-1; NleG5-2; NleG6-1; NleG6-2; NleG6-3; NleG7 (U-box type E3 ubiquitin ligases.); NleG8-2; NleH1 (Ser/Thr protein kinase. Binds directly to a subunit of NF-<kappa>B, the ribosomal protein S3 (RPS3), reducing the nuclear abundance of RPS3 to dampen

					host transcriptional outputs; interact with Bax inhibitor-1 to block apoptosis.); NleH2 (Putative kinase. Attenuates NF-<kappa>B pathway.); SepZ/espZ (EspZ interacts with CD98 in host cell membranes to promote host cell survival, therefore provide the pathogen with valuable time to colonize efficiently prior to dissemination.); TccP2; Tir (Mimics host immunoreceptor tyrosine-based inhibition motifs (ITIMs), also see helicobacter CagA. EHEC Tir lacks the Nck binding site. Conserved NPY (Asn-Pro-Tyr) motif recruits the adaptor protein IRTKS and/or IRSp53. IRTKS/IRSp53 link Tir and TccP/EspFu, which in turn activates N-WASP; Receptor for intimin; effacement; SGLT1 inhibition; recruits SHIP2 to control actin-pedestal morphology; maintains the integrity of the epithelium by keeping the destructive activity of EspG and EspG2 in check.)								
ECs_1815	nleF	T3SS secreted	VFG034966 (gb|WP_00	TTSS secreted	Cif (Deamidase. Induces cytopathic effects of actin stress fiber	setA	Escherichia coli	Effector delivery	nleF	Type III secretion	100	9.23E-141	389

effector NleF	0938103)	effectors (VF1110)	formation and cell cycle arrest.); EspB (Pore formation, actin disruption, microvilli effacement, anti-phagocytosis.); EspF (Inducing degradation of the aniapoptic protein AbcF2, tight junction disruption, microvilli effacement and elongation, mitochondrial dysfunction, N-WASP activation, SGLT-1 inactivation, pedestal maturation, inhibition of NHE3 activity, membrane remodelling; targets and disrupts the nucleolus late in infection, which is temporally controlled by host mitochondria.); EspFu/tccP (Inducing degradation of the aniapoptic protein AbcF2, tight junction disruption, microvilli effacement and elongation, mitochondrial dysfunction, N-WASP activation, SGLT-1 inactivation, pedestal maturation, inhibition of NHE3 activity, membrane remodelling; targets and disrupts the nucleolus late in infection, which is temporally controlled by host mitochondria.); EspG (TBC-like GTPase activating	O157:H7 str. EDL933	system	system effector NleF, caspase inhibitor

protein. Efficiently catalyzes GTP hydrolysis in Rab1 to disrupt of Rab1-mediated ER-to-Golgi trafficking.); EspH (First bacterial effector acting directly on RhoGEFs, EspH directly binds to the DH-PH domain in RhoGEFs to disrupt RhoGEF-Rho signaling; critical for inhibiting macrophage phagocytosis.); EspJ (Inhibit both IgG- and complement receptor-mediated phagocytosis.); EspK; EspL1; EspL2 (Cysteine protease. Bounds F-actin-aggregating annexin 2 directly to increase annexin 2's ability to aggregate Tir-induced F-actin; block necroptosis and in flammation.); EspL4; EspM1 (GEF. Activates the RhoA signaling pathway and induce the formation of stress fibres; inhibit pedestal formation and induce tight junction mislocalization.); EspM2 (GEF. Activates the RhoA signaling pathway and induce the formation of stress fibres; inhibit pedestal formation and induce tight junction

mislocalization.); EspN; EspO1-1; EspO1-2; EspR1; EspR3; EspR4; EspT (GEF. Activates Rac1 and Cdc42 leading to formation of membrane ruffles and lamellipodia; induces membrane ruffles to facilitate bacterial invasion into non-phagocytic cells in a process involving Rac1 and Wave2.); EspW; EspX1; EspX2; EspX4; EspX5; EspX6; EspX7/nleL (E3 ubiquitin ligase, HECT-like. Modulates pedestal formation.); EspY1; EspY2; EspY3; EspY4; EspY5; Map (GEF. Mimics the host Dbl and catalyses the exchange of GDP for GTP in Cdc42, involved in effacement, SGLT1 inhibition, formation of filopodia and disruption of mitochondrial function.); NleA/espI (Disruption of tight junctions by inhibition of host cell protein trafficking through COPII-dependent pathways.); NleB1 (Blocks translocation of the p65 and to the host cell nucleus to inhibit NF-<kappa>B pathway, but NleE and NleB act at different points in the

NF-<kappa>B signaling pathway.); NleB2 (May also have anti-inflammatory activity.); NleC (Metalloprotease. Zn-dependent endopeptidases that specifically clip and inactivate RelA (p65), thus blocking NF-<kappa>B pathway.); NleD (Metalloprotease. Zn-dependent endopeptidases that specifically clip and inactivate JNK and p38, thus blocking AP-1 pathway.); NleE (PMN tran-epithelial migration; blocks translocation of the p65 to the host cell nucleus by preventing I<kappa>B degradation to inhibit NF-<kappa>B pathway.); NleF; NleG-1; NleG2-2; NleG2-3; NleG2-4; NleG5-1; NleG5-2; NleG6-1; NleG6-2; NleG6-3; NleG7 (U-box type E3 ubiquitin ligases.); NleG8-2; NleH1 (Ser/Thr protein kinase. Binds directly to a subunit of NF-<kappa>B, the ribosomal protein S3 (RPS3), reducing the nuclear abundance of RPS3 to dampen host transcriptional outputs; interact with Bax inhibitor-1 to block apoptosis.); NleH2 (Putative kinase.

					Attenuates NF-<kappa>B pathway.); SepZ/espZ (EspZ interacts with CD98 in host cell membranes to promote host cell survival, therefore provide the pathogen with valuable time to colonize efficiently prior to dissemination.); TccP2; Tir (Mimics host immunoreceptor tyrosine-based inhibition motifs (ITIMs), also see helicobacter CagA. EHEC Tir lacks the Nck binding site. Conserved NPY (Asn-Pro-Tyr) motif recruits the adaptor protein IRTKS and/or IRSp53. IRTKS/IRSp53 link Tir and TccP/EspFu, which in turn activates N-WASP; Receptor for intimin; effacement; SGLT1 inhibition; recruits SHIP2 to control actin-pedestal morphology; maintains the integrity of the epithelium by keeping the destructive activity of EspG and EspG2 in check.)								
ECs_4559	eae	T3SS intimin	VFG000803 (gb|WP_000627885)	Intimin (VF0202)	Binding of intimin to Tir triggers dramatic intracellular changes including reorganization of cytoskeletal proteins, actin polymerization at the site of intimate	setA	Escherichia coli O157:H7 str. EDL933	Effector delivery system	eae	intimin type gamma	100	0	1790

					bacterial contact, and formation of a characteristic attaching and effacing (A/E) lesion; alternative intimin receptor may be <beta>1-integrin, but intimin-<beta>1-integrin association is not essential for EHEC adhesin; Another intimin binding protein recently identified as nucleolin								
ECs_0719	pgm	phosphoglucomutase	VFG018259 (gb|WP_006133060)	LPS (VF0367)	Plays a role in entry and early survival inside macrophages; Resistance to innate-immunity anti-bacterial responses; a modulator of the immune response	setA	Brucella canis ATCC 23365	Immune modulation	pgm	phosphoglucomutase	28	2.47E-21	97.4
ECs_0248	fadE	acyl coenzyme A dehydrogenase	VFG043689 (gb|WP_000337350)	Colibactin (VF1179)	-	setA	Escherichia coli O18:K1:H7 str. IHE3034	Exotoxin	clbF	colibactin biosynthesis dehydrogenase ClbF	32.1	1.79E-10	63.5
ECs_0042	caiA	crotonobetaine reductase subunit II	VFG044398 (gb|AAF33139)	Pyridine-2,6-dithiocarboxylic acid (PDTC) (VF0938)	-	setA	Pseudomonas stutzeri KC	Nutritional/Metabolic factor	pdtorf0	putative acyl-CoA dehydrogenase	27.7	3.92E-33	129
ECs_2402	ydiO	acyl-CoA dehydroge	VFG044398 (gb|AAF33	Pyridine-2,6-dithi	-	setA	Pseudomonas stutzeri	Nutritional/Metaboli	pdtorf0	putative acyl-CoA	27.7	6.25E-39	145

		nase	139)	ocarboxylic acid (PDTC) (VF0938)			KC	c factor		dehydrogenase			
ECs_0249	gmhA	D-sedoheptulose 7-phosphate isomerase	VFG013422 (gb|WP_010945115)	LOS (VF0044)	Major immunogen; LOS phosphorylcholine (ChoP) may influence invasion via interaction with PAF receptor and stimulates of inflammatory signals; LPS phase variation is characterized by the spontaneous loss and gain of oligosaccharide structures present in the outer core. the phase variable expression of LPS biosynthesis genes promotes evasion of antigen-specific host immune defences and allow colonization of different host microenvironments	setA	Haemophilus ducreyi 35000HP	Immune modulation	gmhA/lpcA	phosphoheptose isomerase	75	2.62E-101	290
ECs_2835	cpsG	phosphomannomutase	VFG049042 (gb|WP_012967600)	LPS (VF0561)	Resistant to serum complement; also play a role in protecting bacteria from antimicrobial peptides, including polymyxin antibiotics	setA	Klebsiella variicola At-22	Immune modulation	rfbK2	O9 family phosphomannomutase RfbK2	76.1	2.11E-260	714
ECs_2853	cpsG	phosphomannomutase	VFG049042 (gb|WP_012967600)	LPS (VF0561)	Resistant to serum complement; also play a role in protecting bacteria from antimicrobial peptides, including polymyxin antibiotics	setA	Klebsiella variicola At-22	Immune modulation	rfbK2	O9 family phosphomannomutase RfbK2	75.7	4.25E-260	714

ECs_2837	ECs_2837	GDP-mannose mannosyl hydrolase	VFG048863 (gb|WP_014907229)	Capsule (VF0560)	Assisting in evading the host immune system by protecting bacteria from opsonophagocytosis and serum killing	setA	Klebsiella pneumoniae subsp. pneumoniae NTUH-K2044	Immune modulation	KP1_RS17300	GDP-mannose mannosyl hydrolase	50	4.46E-54	168
ECs_2856	ECs_2856	GDP-mannose mannosyl hydrolase	VFG048863 (gb|WP_014907229)	Capsule (VF0560)	Assisting in evading the host immune system by protecting bacteria from opsonophagocytosis and serum killing	setA	Klebsiella pneumoniae subsp. pneumoniae NTUH-K2044	Immune modulation	KP1_RS17300	GDP-mannose mannosyl hydrolase	47	7.87E-55	169
ECs_3283	ligA	DNA ligase	VFG043491 (gb|WP_001124831)	LigA (VF1220)	-	setB	Leptospira interrogans serovar Lai str. 56601	Adherence	ligA	NAD-dependent DNA ligase LigA	34.3	1.01E-110	348
ECs_4522	ligB	DNA ligase	VFG043491 (gb|WP_001124831)	LigA (VF1220)	-	setB	Leptospira interrogans serovar Lai str. 56601	Adherence	ligA	NAD-dependent DNA ligase LigA	21.4	1.79E-17	85.9
ECs_2836	cpsB	mannose-1-phosphate guanyltransferase	VFG023783 (gb|WP_020283358)	O-antigen (VF0392)	LPS O antigen mutants were severely impaired in their ability to colonize the Peyer's patches and did not colonize spleen and liver. The absence of O antigen in the outer membrane affects the expression of other	setA	Yersinia enterocolitica subsp. palearctica 105.5R(r)	Immune modulation	YE105_RS07900	mannose-1-phosphate guanylyltransferase/ mannose-6-phosphate	57.7	6.63E-198	558

					Yersinia virulence factors.					isomerase			
ECs_2854	cpsB	mannose-1-phosphate guanyltransferase	VFG023783 (gb|WP_020283358)	O-antigen (VF0392)	LPS O antigen mutants were severely impaired in their ability to colonize the Peyer's patches and did not colonize spleen and liver. The absence of O antigen in the outer membrane affects the expression of other Yersinia virulence factors.	setA	Yersinia enterocolitica subsp. palearctica 105.5R(r)	Immune modulation	YE105_RS07900	mannose-1-phosphate guanylyltransferase/ mannose-6-phosphate isomerase	63.1	5.92E-217	606
ECs_2831	neuD	shikimate 5-dehydrogenase	VFG037941 (gb|WP_000177817)	Capsule (VF0465)	Plays an important role in protecting bacteria from the host innate immune response	setB	Acinetobacter baumannii AB307-0294	Immune modulation	ABBFA_RS17130	acetyltransferase	50.9	1.92E-68	209
ECs_2006	ECs_2006	BigA-like protein	VFG035969 (gb|WP_011579121)	UpaH, AIDA-I type (VF1126)	-	setB	Escherichia coli 536	Biofilm	upaH	autotransporter adhesin	57.2	7.87E-135	449
ECs_2007	ECs_2007	BigB-like protein	VFG035969 (gb|WP_011579121)	UpaH, AIDA-I type (VF1126)	-	setB	Escherichia coli 536	Biofilm	upaH	autotransporter adhesin	60.4	1.36E-225	696
ECs_3067	mepS	murein DD-endopeptidase	VFG006734 (gb|WP_010990501)	P60 (VF0068)	Murein hydrolase activity required for normal septum formation and essential for cell viability; may play a role in intestinal invasion and in vivo survival	setA	Listeria innocua Clip11262	Invasion	iap/cwhA	P60 extracellular protein, invasion associated	37.8	5.41E-13	66.2

									protein Iap			
ECs_2364	mepH	endopeptidase	VFG019355 (gb|WP_012581841)	P60 (VF0068)	Murein hydrolase activity required for normal septum formation and essential for cell viability; may play a role in intestinal invasion and in vivo survival	setA	Listeria monocytogenes HCC23	Invasion	iap/cwhA	P60 extracellular protein, invasion associated protein Iap	39.1	3.79E-15	74.7
ECs_0867	clsB	cardiolipin synthase 2	VFG037201 (gb|WP_000079186)	Phospholipase D (VF0469)	Contribute to the pathogenesis by aiding in the lysis of host cells, via cleavage of phospholipids present in the host cell membrane, and by degrading phospholipids present at mucosal barriers to facilitate bacterial invasion	setA	Acinetobacter baumannii AB0057	Exotoxin	plcD	phosphatidylserine/phosphatidylglycerophosphate/cardiolipin synthase	21.7	2.78E-11	65.1
ECs_1424	clsC	stationary phase cardiolipin synthase 3	VFG037201 (gb|WP_000079186)	Phospholipase D (VF0469)	Contribute to the pathogenesis by aiding in the lysis of host cells, via cleavage of phospholipids present in the host cell membrane, and by degrading phospholipids present at mucosal barriers to facilitate bacterial invasion	setA	Acinetobacter baumannii AB0057	Exotoxin	plcD	phosphatidylserine/phosphatidylglycerophosphate/cardiolipin synthase	38.4	2.65E-111	340
ECs_3855	espL2	T3SS secreted effector EspL	VFG034762 (gb|NP_311882)	TTSS secreted effectors (VF1110)	Cif (Deamidase. Induces cytopathic effects of actin stress fiber formation and cell cycle arrest.); EspB (Pore formation, actin	setA	Escherichia coli O157:H7 str. Sakai	Effector delivery system	espL2	Type III secretion system secreted	100	0	905

disruption, microvilli effacement, anti-phagocytosis.); EspF (Inducing degradation of the aniapoptic protein AbcF2, tight junction disruption, microvilli effacement and elongation, mitochondrial dysfunction, N-WASP activation, SGLT-1 inactivation, pedestal maturation, inhibition of NHE3 activity, membrane remodelling; targets and disrupts the nucleolus late in infection, which is temporally controlled by host mitochondria.); EspFu/tccP (Inducing degradation of the aniapoptic protein AbcF2, tight junction disruption, microvilli effacement and elongation, mitochondrial dysfunction, N-WASP activation, SGLT-1 inactivation, pedestal maturation, inhibition of NHE3 activity, membrane remodelling; targets and disrupts the nucleolus late in infection, which is temporally controlled by host mitochondria.); EspG (TBC-like GTPase activating protein. Efficiently catalyzes GTP hydrolysis in Rab1 to disrupt of	effector EspL2, cysteine protease

Rab1-mediated ER-to-Golgi trafficking.); EspH (First bacterial effector acting directly on RhoGEFs, EspH directly binds to the DH-PH domain in RhoGEFs to disrupt RhoGEF-Rho signaling; critical for inhibiting macrophage phagocytosis.); EspJ (Inhibit both IgG- and complement receptor-mediated phagocytosis.); EspK; EspL1; EspL2 (Cysteine protease. Bounds F-actin-aggregating annexin 2 directly to increase annexin 2's ability to aggregate Tir-induced F-actin; block necroptosis and in flammation.); EspL4; EspM1 (GEF. Activates the RhoA signaling pathway and induce the formation of stress fibres; inhibit pedestal formation and induce tight junction mislocalization.); EspM2 (GEF. Activates the RhoA signaling pathway and induce the formation of stress fibres; inhibit pedestal formation and induce tight junction mislocalization.); EspN; EspO1-1; EspO1-2; EspR1; EspR3; EspR4; EspT

(GEF. Activates Rac1 and Cdc42 leading to formation of membrane ruffles and lamellipodia; induces membrane ruffles to facilitate bacterial invasion into non-phagocytic cells in a process involving Rac1 and Wave2.); EspW; EspX1; EspX2; EspX4; EspX5; EspX6; EspX7/nleL (E3 ubiquitin ligase, HECT-like. Modulates pedestal formation.); EspY1; EspY2; EspY3; EspY4; EspY5; Map (GEF. Mimics the host Dbl and catalyses the exchange of GDP for GTP in Cdc42, involved in effacement, SGLT1 inhibition, formation of filopodia and disruption of mitochondrial function.); NleA/espI (Disruption of tight junctions by inhibition of host cell protein trafficking through COPII-dependent pathways.); NleB1 (Blocks translocation of the p65 and to the host cell nucleus to inhibit NF-<kappa>B pathway, but NleE and NleB act at different points in the NF-<kappa>B signaling pathway.); NleB2 (May also have anti-inflammatory

activity.); NleC (Metalloprotease. Zn-dependent endopeptidases that specifically clip and inactivate RelA (p65), thus blocking NF-<kappa>B pathway.); NleD (Metalloprotease. Zn-dependent endopeptidases that specifically clip and inactivate JNK and p38, thus blocking AP-1 pathway.); NleE (PMN tran-epithelial migration; blocks translocation of the p65 to the host cell nucleus by preventing I<kappa>B degradation to inhibit NF-<kappa>B pathway.); NleF; NleG-1; NleG2-2; NleG2-3; NleG2-4; NleG5-1; NleG5-2; NleG6-1; NleG6-2; NleG6-3; NleG7 (U-box type E3 ubiquitin ligases.); NleG8-2; NleH1 (Ser/Thr protein kinase. Binds directly to a subunit of NF-<kappa>B, the ribosomal protein S3 (RPS3), reducing the nuclear abundance of RPS3 to dampen host transcriptional outputs; interact with Bax inhibitor-1 to block apoptosis.); NleH2 (Putative kinase. Attenuates NF-<kappa>B pathway.); SepZ/espZ (EspZ interacts with CD98 in

					host cell membranes to promote host cell survival, therefore provide the pathogen with valuable time to colonize efficiently prior to dissemination.); TccP2; Tir (Mimics host immunoreceptor tyrosine-based inhibition motifs (ITIMs), also see helicobacter CagA. EHEC Tir lacks the Nck binding site. Conserved NPY (Asn-Pro-Tyr) motif recruits the adaptor protein IRTKS and/or IRSp53. IRTKS/IRSp53 link Tir and TccP/EspFu, which in turn activates N-WASP; Receptor for intimin; effacement; SGLT1 inhibition; recruits SHIP2 to control actin-pedestal morphology; maintains the integrity of the epithelium by keeping the destructive activity of EspG and EspG2 in check.)								
ECs_2714	espJ	T3SS secreted effector EspJ	VFG012438 (gb|WP_000022458)	TTSS secreted effectors (VF1110)	Cif (Deamidase. Induces cytopathic effects of actin stress fiber formation and cell cycle arrest.); EspB (Pore formation, actin disruption, microvilli effacement, anti-phagocytosis.); EspF (Inducing degradation of the aniapoptic protein	setA	Escherichia coli O157:H7 str. EDL933	Effector delivery system	espJ	Type III secretion system effector EspJ, adenosine diphosphat	100	6.33E-155	427

AbcF2, tight junction disruption, microvilli effacement and elongation, mitochondrial dysfunction, N-WASP activation, SGLT-1 inactivation, pedestal maturation, inhibition of NHE3 activity, membrane remodelling; targets and disrupts the nucleolus late in infection, which is temporally controlled by host mitochondria.); EspFu/tccP (Inducing degradation of the aniapoptic protein AbcF2, tight junction disruption, microvilli effacement and elongation, mitochondrial dysfunction, N-WASP activation, SGLT-1 inactivation, pedestal maturation, inhibition of NHE3 activity, membrane remodelling; targets and disrupts the nucleolus late in infection, which is temporally controlled by host mitochondria.); EspG (TBC-like GTPase activating protein. Efficiently catalyzes GTP hydrolysis in Rab1 to disrupt of Rab1-mediated ER-to-Golgi trafficking.); EspH (First bacterial effector acting directly on RhoGEFs,	e (ADP) ribosyltra nsferase

EspH directly binds to the DH-PH domain in RhoGEFs to disrupt RhoGEF-Rho signaling; critical for inhibiting macrophage phagocytosis.); EspJ (Inhibit both IgG- and complement receptor-mediated phagocytosis.); EspK; EspL1; EspL2 (Cysteine protease. Bounds F-actin-aggregating annexin 2 directly to increase annexin 2's ability to aggregate Tir-induced F-actin; block necroptosis and in flammation.); EspL4; EspM1 (GEF. Activates the RhoA signaling pathway and induce the formation of stress fibres; inhibit pedestal formation and induce tight junction mislocalization.); EspM2 (GEF. Activates the RhoA signaling pathway and induce the formation of stress fibres; inhibit pedestal formation and induce tight junction mislocalization.); EspN; EspO1-1; EspO1-2; EspR1; EspR3; EspR4; EspT (GEF. Activates Rac1 and Cdc42 leading to formation of membrane ruffles and lamellipodia; induces membrane

ruffles to facilitate bacterial invasion into non-phagocytic cells in a process involving Rac1 and Wave2.); EspW; EspX1; EspX2; EspX4; EspX5; EspX6; EspX7/nleL (E3 ubiquitin ligase, HECT-like. Modulates pedestal formation.); EspY1; EspY2; EspY3; EspY4; EspY5; Map (GEF. Mimics the host Dbl and catalyses the exchange of GDP for GTP in Cdc42, involved in effacement, SGLT1 inhibition, formation of filopodia and disruption of mitochondrial function.); NleA/espI (Disruption of tight junctions by inhibition of host cell protein trafficking through COPII-dependent pathways.); NleB1 (Blocks translocation of the p65 and to the host cell nucleus to inhibit NF-<kappa>B pathway, but NleE and NleB act at different points in the NF-<kappa>B signaling pathway.); NleB2 (May also have anti-inflammatory activity.); NleC (Metalloprotease. Zn-dependent endopeptidases that specifically clip and inactivate RelA

(p65), thus blocking NF-<kappa>B pathway.); NleD (Metalloprotease. Zn-dependent endopeptidases that specifically clip and inactivate JNK and p38, thus blocking AP-1 pathway.); NleE (PMN tran-epithelial migration; blocks translocation of the p65 to the host cell nucleus by preventing I<kappa>B degradation to inhibit NF-<kappa>B pathway.); NleF; NleG-1; NleG2-2; NleG2-3; NleG2-4; NleG5-1; NleG5-2; NleG6-1; NleG6-2; NleG6-3; NleG7 (U-box type E3 ubiquitin ligases.); NleG8-2; NleH1 (Ser/Thr protein kinase. Binds directly to a subunit of NF-<kappa>B, the ribosomal protein S3 (RPS3), reducing the nuclear abundance of RPS3 to dampen host transcriptional outputs; interact with Bax inhibitor-1 to block apoptosis.); NleH2 (Putative kinase. Attenuates NF-<kappa>B pathway.); SepZ/espZ (EspZ interacts with CD98 in host cell membranes to promote host cell survival, therefore provide the pathogen with valuable time to

ECs_2715	tccP/espFU/espF2-2	T3SS secreted effector TccP	VFG033773 (gb|WP_013009221)	TTSS secreted effectors (VF1111)	colonize efficiently prior to dissemination.); TccP2; Tir (Mimics host immunoreceptor tyrosine-based inhibition motifs (ITIMs), also see helicobacter CagA. EHEC Tir lacks the Nck binding site. Conserved NPY (Asn-Pro-Tyr) motif recruits the adaptor protein IRTKS and/or IRSp53. IRTKS/IRSp53 link Tir and TccP/EspFu, which in turn activates N-WASP; Receptor for intimin; effacement; SGLT1 inhibition; recruits SHIP2 to control actin-pedestal morphology; maintains the integrity of the epithelium by keeping the destructive activity of EspG and EspG2 in check.) EspF (Inducing degradation of the aniapoptic protein AbcF2, tight junction disruption, microvilli effacement and elongation, mitochondrial dysfunction, N-WASP activation, SGLT-1 inactivation, pedestal maturation, inhibition of NHE3 activity, membrane remodelling; targets and disrupts the nucleolus late in infection, which is temporally	setA	Escherichia coli O55:H7 str. CB9615	Effector delivery system	espFu/tccP	Type III secretion system effector TccP	100	2.03E-76	236

controlled by host mitochondria.); EspFu/tccP (Inducing degradation of the aniapoptic protein AbcF2, tight junction disruption, microvilli effacement and elongation, mitochondrial dysfunction, N-WASP activation, SGLT-1 inactivation, pedestal maturation, inhibition of NHE3 activity, membrane remodelling; targets and disrupts the nucleolus late in infection, which is temporally controlled by host mitochondria.); EspG (TBC-like GTPase activating protein. Efficiently catalyzes GTP hydrolysis in Rab1 to disrupt of Rab1-mediated ER-to-Golgi trafficking.); EspH (First bacterial effector acting directly on RhoGEFs, EspH directly binds to the DH-PH domain in RhoGEFs to disrupt RhoGEF-Rho signaling; critical for inhibiting macrophage phagocytosis.); EspJ (Inhibit both IgG- and complement receptor-mediated phagocytosis.); EspL1; EspL2 (Cysteine protease. Bounds F-actin-aggregating annexin 2

directly to increase annexin 2's ability to aggregate Tir-induced F-actin; block necroptosis and in flammation.); EspM1 (GEF. Activates the RhoA signaling pathway and induce the formation of stress fibres; inhibit pedestal formation and induce tight junction mislocalization.); EspM2 (GEF. Activates the RhoA signaling pathway and induce the formation of stress fibres; inhibit pedestal formation and induce tight junction mislocalization.); EspO1-1; EspR1; EspR3; EspR4; EspV (Inducing radical morphological changes in host cells.); EspW; EspX1; EspX2; EspX4; EspX5; EspX6; EspY1; EspY2; EspY3; EspY4; Map (GEF. Mimics the host Dbl and catalyses the exchange of GDP for GTP in Cdc42, involved in effacement, SGLT1 inhibition, formation of filopodia and disruption of mitochondrial function.); NleA/espI (Disruption of tight junctions by inhibition of host cell protein trafficking through COPII-dependent

pathways.); NleB2 (May also have anti-inflammatory activity.); NleB2-2; NleC (Metalloprotease. Zn-dependent endopeptidases that specifically clip and inactivate RelA (p65), thus blocking NF-<kappa>B pathway.); NleD (Metalloprotease. Zn-dependent endopeptidases that specifically clip and inactivate JNK and p38, thus blocking AP-1 pathway.); NleE (PMN tran-epithelial migration; blocks translocation of the p65 to the host cell nucleus by preventing I<kappa>B degradation to inhibit NF-<kappa>B pathway.); NleF; NleG-1; NleG-2; NleG-3; NleG2-2; NleG2-4; NleG5-1; NleG6-1; NleG7 (U-box type E3 ubiquitin ligases.); NleG8-2; NleH1 (Ser/Thr protein kinase. Binds directly to a subunit of NF-<kappa>B, the ribosomal protein S3 (RPS3), reducing the nuclear abundance of RPS3 to dampen host transcriptional outputs; interact with Bax inhibitor-1 to block apoptosis.); NleH2 (Putative kinase. Attenuates NF-<kappa>B

					pathway.); SepZ/espZ (EspZ interacts with CD98 in host cell membranes to promote host cell survival, therefore provide the pathogen with valuable time to colonize efficiently prior to dissemination.); Tir (Mimics host immunoreceptor tyrosine-based inhibition motifs (ITIMs), also see helicobacter CagA. EHEC Tir lacks the Nck binding site. Conserved NPY (Asn-Pro-Tyr) motif recruits the adaptor protein IRTKS and/or IRSp53. IRTKS/IRSp53 link Tir and TccP/EspFu, which in turn activates N-WASP; Receptor for intimin; effacement; SGLT1 inhibition; recruits SHIP2 to control actin-pedestal morphology; maintains the integrity of the epithelium by keeping the destructive activity of EspG and EspG2 in check.)								
ECs_2828	wzzB	O-antigen chain length determinant protein	VFG023778 (gb|WP_005181087)	O-antigen (VF0392)	LPS O antigen mutants were severely impaired in their ability to colonize the Peyer's patches and did not colonize spleen and liver. The absence of O antigen in the outer membrane affects the expression of other	setA	Yersinia enterocolitica subsp. palearctica 105.5R(r)	Immune modulation	wzzE	ECA polysaccharide chain length modulation protein	24.8	5.44E-21	92

					Yersinia virulence factors.								
ECs_4718	wzzE	enterobacterial common antigen polysaccharide chain length modulation protein	VFG023778 (gb|WP_005181087)	O-antigen (VF0392)	LPS O antigen mutants were severely impaired in their ability to colonize the Peyer's patches and did not colonize spleen and liver. The absence of O antigen in the outer membrane affects the expression of other Yersinia virulence factors.	setA	Yersinia enterocolitica subsp. palearctica 105.5R(r)	Immune modulation	wzzE	ECA polysaccharide chain length modulation protein	69.9	1.23E-171	481
ECs_0626	fepE	regulator of length of O-antigen component of lipopolysaccharide chains	VFG000927 (gb|WP_000096764)	Enterobactin (VF0228)	Iron uptake: the siderophore enterobactin imported through the FepA receptor and the FepBCDG system	setA	Escherichia coli CFT073	Nutritional/Metabolic factor	fepE	LPS O-antigen length regulator	96	1.92E-252	687
ECs_2829	ugd	UDP-glucose 6-dehydrogenase	VFG048789 (gb|WP_014838943)	Capsule (VF0560)	Assisting in evading the host immune system by protecting bacteria from opsonophagocytosis and serum killing	setA	Klebsiella oxytoca E718	Immune modulation	ugd	UDP-glucose 6-dehydrogenase	84	1.98E-239	655
ECs_5094	basS	two-component	VFG022914 (gb|WP_01	PhoP (VF0286)	Senses Mg2+ starvation, controls expression of genes involved in	setA	Mycobacterium	Regulation	phoR	Possible two	29.9	3.20E-25	106

		system sensor histidine kinase BasS	4000393)		surface remodeling and adaptation to intracellular growth		canettii CIPT 140010059			component system response sensor kinase membrane associated PhoR			
ECs_5115	cadC	cadBA operon transcriptional activator	VFG041619 (gb|WP_015835582)	T3SS-2 (VF1287)	-	setA	Photorhabdus asymbiotica subsp. asymbiotica ATCC 43949	Effector delivery system	PAU_RS17165	winged helix-turn-helix domain-containing protein	33.7	9.74E-08	52
ECs_0449	phoB	two-component regulatory system response regulator PhoB	VFG031730 (gb|WP_014876914)	RegX3 (VF0858)	-	setA	Mycobacterium smegmatis str. MC2 155	Regulation	regX3	two-component sensory transduction protein RegX	40.7	6.93E-50	162
ECs_2887	baeR	two-component regulatory system	VFG031730 (gb|WP_014876914)	RegX3 (VF0858)	-	setA	Mycobacterium smegmatis str. MC2	Regulation	regX3	two-component sensory transduction protein	37.9	3.17E-48	158

		response regulator BaeR					155			RegX			
ECs_5356	creB	two-component regulatory system response regulator CreR	VFG031729 (gb|WP_011891085)	RegX3 (VF0858)	-	setA	Mycobacterium gilvum PYR-GCK	Regulation	regX3	two-component sensory transduction protein RegX	37.8	7.03E-47	154
ECs_1714	lolB	lipoprotein localization factor	VFG013624 (gb|WP_011609152)	Heme biosynthesis (VF0758)	-	setB	Haemophilus somnus 129PT	Nutritional/Metabolic factor	hemM	lipoprotein insertase outer membrane protein LolB	29.4	4.92E-33	117
ECs_3713	ygeM	hypothetical protein	VFG042093 (gb|NP_311740)	ETT2 (VF1161)	-	setB	Escherichia coli O157:H7 str. Sakai	Effector delivery system	ygeM	hypothetical protein	100	2.27E-105	296
ECs_3715	ECs_3715	type III secretion apparatus protein OrgA/MxiK	VFG042091 (gb|NP_311742)	ETT2 (VF1161)	-	setA	Escherichia coli O157:H7 str. Sakai	Effector delivery system	ECs_3715	type III secretion apparatus protein OrgA/MxiK	100	4.74E-145	400
ECs_4665	lpfD	fimbrial	VFG042593	Stg	-	setA	Escherichi	Adherence	stgD	StgD	41.6	2.05E-	247

		protein	(gb|AAS99232)	fimbriae (VF1150)			a coli O78:K80:H9 chi7122					79	
ECs_4666	lpfD	fimbrial protein	VFG042593 (gb|AAS99232)	Stg fimbriae (VF1150)	-	setA	Escherichia coli O78:K80:H9 chi7122	Adherence	stgD	StgD	37.6	2.53E-63	205
gene-espP	espP	Lipid A biosynthesis (Kdo)2-(lauroyl)-lipid IVA acyltransferase	VFG000844 (gb|WP_001034100)	EspP (VF0208)	Serine protease; cleaves coagulation factor V	setA	Escherichia coli O157:H7 str. EDL933	Effector delivery system	espP	autotransporter, serine protease	100	0	2561
ECs_2538	yebQ	transporter	VFG044351 (gb|WP_033111749)	Achromobactin (VF1243)	-	setA	Dickeya dadantii 3937	Nutritional/Metabolic factor	DDA3937_RS07730	DHA2 family efflux MFS transporter permease subunit	26.8	1.59E-30	123
ECs_5300	mdtM	multidrug efflux system protein	VFG044351 (gb|WP_033111749)	Achromobactin (VF1243)	-	setA	Dickeya dadantii 3937	Nutritional/Metabolic factor	DDA3937_RS07730	DHA2 family efflux MFS transporter permease subunit	24.7	3.09E-09	58.5
ECs_3728	eivJ1	type III	VFG042079	ETT2	-	setB	Escherichi	Effector	eivJ1	type III	100	6.86E-	148

		secretion system protein EivJ1	(gb|NP_311755)	(VF1161)			a coli O157:H7 str. Sakai	delivery system		secretion system protein EivJ1		49	
ECs_4379	ECs_4379	hemin transporter	VFG012591 (gb|WP_001017167)	Chu (VF0234)	Iron uptake: the ability to use heme and/or hemoglobin might be especially advantageous to pathogenic bacteria. These pathogens often secrete cytotoxins, which gain access to the intracellular heme reservoir besides initiating tissue invasion. Cytotoxin production coupled with the capability to utilize heme and/or hemoglobin could serve as an effective iron acquisition strategy during the progression of infection	setA	Escherichia coli O157:H7 str. EDL933	Nutritional/Metabolic factor	chuS	heme oxygenase ChuS	100	6.51E-256	693
ECs_4153	fmt	L-methionyl-tRNA(fMet) N-formyltransferase	VFG021856 (gb|WP_029104465)	Exochelin (smegmatis) (VF0819)	-	setA	Mycobacterium smegmatis str. MC2 155	Nutritional/Metabolic factor	fxbA	methionyl-tRNA formyltransferase	32.2	9.59E-34	125
ECs_1734	purU	formyltetrahydrofolate hydrolase	VFG021856 (gb|WP_029104465)	Exochelin (smegmatis) (VF0819)	-	setA	Mycobacterium smegmatis str. MC2	Nutritional/Metabolic factor	fxbA	methionyl-tRNA formyltransferase	28.6	3.77E-07	50.4

							155						
ECs_3362	purN	phosphoribosylglycinamide formyltransferase 1	VFG021856 (gb|WP_029104465)	Exochelin (smegmatis) (VF0819)	-	setA	Mycobacterium smegmatis str. MC2 155	Nutritional/Metabolic factor	fxbA	methionyl-tRNA formyltransferase	35.6	1.16E-08	53.9
ECs_3143	arnA	UDP-4-amino-4-deoxy-L-arabinose formyltransferase	VFG021856 (gb|WP_029104465)	Exochelin (smegmatis) (VF0819)	-	setA	Mycobacterium smegmatis str. MC2 155	Nutritional/Metabolic factor	fxbA	methionyl-tRNA formyltransferase	31.8	1.79E-37	142
ECs_0594	fimD	outer membrane usher protein FimD	VFG042682 (gb|CAD56973)	Type 1 fimbriae (VF1211)	-	setA	Citrobacter freundii str. 3009	Adherence	fimD	gene) outer membrane usher protein	71.8	0	1315
ECs_0595	sfmH	fimbrial-like adhesin protein	VFG021168 (gb|WP_000708656)	Type 1 fimbriae (VF0102)	The adhesin FimH mediates T3SS1-independent uptake in murine DCs.	setA	Salmonella enterica subsp. enterica serovar Enteritidis str. P125109	Adherence	fimH	type I fimbriae minor fimbrial subunit FimH, adhesin	71	7.28E-169	473

ECs_0596	sfmF	fimbrial protein	VFG042684 (gb|CAD56975)	Type 1 fimbriae (VF1211)	-	setA	Citrobacter freundii str. 3009	Adherence	fimF	gene) major fimbrial subunit	62.5	2.00E-65	197
ECs_5327	fhuF	ferric iron reductase involved in ferric hydroxamate transport	VFG044312 (gb|WP_006488656)	Ornibactin (VF0701)	-	setB	Burkholderia cenocepacia J2315	Nutritional/Metabolic factor	fhuF	siderophore-iron reductase FhuF	31.3	1.17E-18	82.8
ECs_3720	etrA	transcriptional regulator	VFG042088 (gb|NP_311747)	ETT2 (VF1161)	-	setB	Escherichia coli O157:H7 str. Sakai	Effector delivery system	etrA	transcriptional regulator	100	5.10E-120	335
ECs_3721	eprS	T3SS component EprS	VFG042086 (gb|NP_311748)	ETT2 (VF1161)	-	setA	Escherichia coli O157:H7 str. Sakai	Effector delivery system	eprS	T3SS component EprS	100	8.67E-263	713
gene-etpD	etpD	Type II secretion pathway related protein	VFG040932 (gb|WP_001302177)	Etp (VF1178)	-	setA	Escherichia coli O157:H7 str. EDL933	Effector delivery system	etpD	variant type II secretion system secretin EtpD	100	0	1101
ECs_5154	nsrR	nitric	VFG011941	Flagella	Penetrating the mucus barrier;	setB	Campylobac	Motility	CFF8240_RS	Rrf2 family	23.7	3.96E-	43.5

		oxide-sensitive repressor for NO regulon	(gb|WP_002849697)	(VF0114)	chemotaxis is also important for intestinal colonization; In the absence of a type III secretion system, the flagellar secretion apparatus appears to secrete several putative virulence proteins including FspA, FlaC and up to eight Cia proteins		ter fetus subsp. fetus 82-40		05385	transcriptional regulator		06	
ECs_3397	iscR	transcriptional regulator	VFG011941 (gb|WP_002849697)	Flagella (VF0114)	Penetrating the mucus barrier; chemotaxis is also important for intestinal colonization; In the absence of a type III secretion system, the flagellar secretion apparatus appears to secrete several putative virulence proteins including FspA, FlaC and up to eight Cia proteins	setA	Campylobacter fetus subsp. fetus 82-40	Motility	CFF8240_RS05385	Rrf2 family transcriptional regulator	25.4	4.18E-10	54.7
ECs_0592	sfmA	type-1 fimbrial protein subunit A	VFG042679 (gb|CAD56970)	Type 1 fimbriae (VF1211)	-	setA	Citrobacter freundii str. 3009	Adherence	fimA	gene) major fimbrial subunit	66.3	1.75E-75	224
ECs_0593	sfmC	periplasmic pilus chaperone	VFG042681 (gb|CAD56972)	Type 1 fimbriae (VF1211)	-	setA	Citrobacter freundii str. 3009	Adherence	fimC	gene) fimbrial chaperone protein	67.7	6.57E-106	305
ECs_1279	ECs_1279	chaperone protein	VFG042681 (gb|CAD56972)	Type 1 fimbriae (VF1211)	-	setA	Citrobacter freundii str. 3009	Adherence	fimC	gene) fimbrial chaperone	35.7	7.28E-39	134

										protein			
ECs_4567	ECs_4567	T3SS component	VFG000811 (gb|WP_001062956)	TTSS (VF0191)	Injects Tir and other effector molecules directly into the host cell. Effector molecules activate cell-signaling pathways, causing alterations in the host cell cytoskeleton and resulting in the depolymerization of actin and the loss of microvilli	setA	Escherichia coli O157:H7 str. EDL933	Effector delivery system	escO	positive regulator EscO	100	1.02E-61	183
ECs_1432	lpxL	lauryl-acyl carrier protein-dependent acyltransferase	VFG013437 (gb|WP_005656817)	LOS (VF0044)	Major immunogen; LOS phosphorylcholine (ChoP) may influence invasion via interaction with PAF receptor and stimulates of inflammatory signals; LPS phase variation is characterized by the spontaneous loss and gain of oligosaccharide structures present in the outer core. the phase variable expression of LPS biosynthesis genes promotes evasion of antigen-specific host immune defences and allow colonization of different host microenvironments	setA	Haemophilus influenzae 86-028NP	Immune modulation	htrB	lipid A biosynthesis lauroyl acyltransferase	56.5	1.11E-121	351
ECs_3258	lpxP	palmitoleoyl-acyl carrier	VFG013437 (gb|WP_005656817)	LOS (VF0044)	Major immunogen; LOS phosphorylcholine (ChoP) may influence invasion via interaction	setA	Haemophilus influenzae	Immune modulation	htrB	lipid A biosynthesis lauroyl	48.5	2.15E-99	294

		protein-dependent acyltransferase			with PAF receptor and stimulates of inflammatory signals; LPS phase variation is characterized by the spontaneous loss and gain of oligosaccharide structures present in the outer core. the phase variable expression of LPS biosynthesis genes promotes evasion of antigen-specific host immune defences and allow colonization of different host microenvironments		86-028NP			acyltransferase			
ECs_4003	garR	tartronate semialdehyde reductase	VFG048959 (gb|WP_020956638)	Capsule (VF0560)	Assisting in evading the host immune system by protecting bacteria from opsonophagocytosis and serum killing	setA	Klebsiella pneumoniae JM45	Immune modulation	gndA	NADP-dependent phosphogluconate dehydrogenase	24.1	1.33E-08	55.5
ECs_2830	gnd	6-phosphogluconate dehydrogenase	VFG048959 (gb|WP_020956638)	Capsule (VF0560)	Assisting in evading the host immune system by protecting bacteria from opsonophagocytosis and serum killing	setA	Klebsiella pneumoniae JM45	Immune modulation	gndA	NADP-dependent phosphogluconate dehydrogenase	95.3	0	885
ECs_0570	glxR	tartronate semialdeh	VFG048844 (gb|WP_014838945)	Capsule (VF0560)	Assisting in evading the host immune system by protecting bacteria from opsonophagocytosis and serum killing	setA	Klebsiella oxytoca E718	Immune modulation	gndA	NADP-dependent phosphoglu	23.5	4.10E-06	47.8

		yde reductase								conate dehydrogenase			
ECs_0521	ybaN	inner membrane protein	VFG037537 (gb|WP_000359621)	HemO cluster (VF0636)	Current studies show the hemO gene cluster is required for optimal utilization of heme in A. baumannii hypervirulent strains; The hemO gene cluster encodes a secreted hemophore.	setA	Acinetobacter baumannii ACICU	Nutritional/Metabolic factor	ACICU_RS04605	YbaN family protein	32.4	7.80E-15	66.2
ECs_0529	aes	acetyl esterase	VFG016039 (gb|WP_012075665)	Pyoverdine (VF0094)	Effective at acquiring iron from transferrin and lactoferrin; cytotoxic due to its ability to stimulating the production of reactive oxygen species	setA	Pseudomonas aeruginosa PA7	Nutritional/Metabolic factor	pvdJ	pyoverdine biosynthesis protein PvdJ	30	1.61E-30	117
ECs_0528	hemH	ferrochelatase	VFG013202 (gb|WP_167591114)	Heme biosynthesis (VF0758)	-	setB	Haemophilus somnus 2336	Nutritional/Metabolic factor	hemH	ferrochelatase	50.9	5.76E-115	335
ECs_1725	ychO	invasin	VFG048218 (gb|WP_112473261)	Invasin D (VF0576)	Modulates Ig functions in the intestine and affects direct interactions with a subset of cell surface-exposed B-cell receptors	setA	Yersinia pseudotuberculosis YPIII	Invasion	invD	invasin D protein	33.7	8.72E-67	233
ECs_1720	kdsA	3-deoxy-D-manno-octulosonate 8-phospha	VFG013466 (gb|WP_005654784)	LOS (VF0044)	Major immunogen; LOS phosphorylcholine (ChoP) may influence invasion via interaction with PAF receptor and stimulates of inflammatory signals; LPS phase	setA	Haemophilus influenzae 86-028NP	Immune modulation	kdsA	2-dehydro-3-deoxyphosphooctonate aldolase	82	7.75E-171	473

		te synthase			variation is characterized by the spontaneous loss and gain of oligosaccharide structures present in the outer core. the phase variable expression of LPS biosynthesis genes promotes evasion of antigen-specific host immune defences and allow colonization of different host microenvironments							
ECs_0091	mraY	phospho-N-acetylmuramoyl-pentapeptide-transferase	VFG014146 (gb|WP_003138482)	LPS (VF0085)	Mediates biological effects including resistance to serum killing and phagocytosis; the binding to normal CFTR (cystic fibrosis transmembrane conductance regulator) and invasion of host cells may make a contribution to virulence in the human eye; internalization by binding to normal CFTR protein expressed by airway epithelial cells followed by desquamation of bacteria-laden epithelial cells, constitutes a host defense mechanism. If this mechanism fails to function properly, abnormally high bacterial carriage would promote the establishment of chronic bacterial infection	setA	Pseudomonas aeruginosa UCBPP-PA14	Immune modulation	PA14_RS09495	glycosyltransferase family 4 protein	30.2	4.03E-17	81.3

ECs_4716	rho	transcription termination factor Rho	VFG041866 (gb|WP_011136150)	Cpi-2 encoded T3SS (SPI-2 like) (VF1260)	-	setA	Chromobacterium violaceum ATCC 12472	Effector delivery system	CV_RS12740	EscN/YscN/HrcN family type III secretion system ATPase	27.9	3.82E-19	89
ECs_4717	wecA	UDP-GlcNAc:undecaprenylphosphate GlcNAc-1-phosphate transferase	VFG013509 (gb|WP_005688044)	LOS (VF0044)	Major immunogen; LOS phosphorylcholine (ChoP) may influence invasion via interaction with PAF receptor and stimulates of inflammatory signals; LPS phase variation is characterized by the spontaneous loss and gain of oligosaccharide structures present in the outer core. the phase variable expression of LPS biosynthesis genes promotes evasion of antigen-specific host immune defences and allow colonization of different host microenvironments	setA	Haemophilus influenzae 86-028NP	Immune modulation	wecA	undecaprenyl-phosphate alpha-N-acetylglucosaminyltransferase	55.9	8.63E-123	358
ECs_4719	wecB	UDP-N-acetyl glucosamine-2-epimerase	VFG007640 (gb|WP_043877039)	Capsular polysaccharide (VF0624)	-	setA	Vibrio vulnificus YJ016	Immune modulation	wbjD/wecB	UDP-N-acetylglucosamine 2-epimerase (non-hydro	72	2.56E-196	545

										lyzing)			
ECs_0415	ECs_0415	periplasmic-iron-binding protein	VFG001205 (gb|WP_002214250)	FbpABC (VF0272)	Encodes a periplasmic-binding protein-dependent iron transport system necessary for the utilization of iron bound to transferrin or iron chelates, FbpA is the periplasmic Fe3+ binding protein	setA	Neisseria meningitidis MC58	Nutritional/Metabolic factor	fbpA	iron(III) ABC transporter, periplasmic binding protein	25	7.77E-10	59.3
ECs_0416	ECs_0416	regulatory protein	VFG032173 (gb|WP_014092267)	Hpt (VF0264)	The mammalian G6PT is responsible for the uptake of G6P from the cytosol into the endoplasmic reticulum for its conversion into the central fueling metabolite, glucose. Hpt mimics the function of the mammalian G6PT to steal fueling metabolites from host cell cytosol for the benefit of the microbe.	setA	Listeria ivanovii subsp. ivanovii PAM 55	Nutritional/Metabolic factor	hpt	hexose phosphate transport protein	26.6	2.41E-42	155
ECs_5316	lgoT	L-galactonate transporter	VFG032173 (gb|WP_014092267)	Hpt (VF0264)	The mammalian G6PT is responsible for the uptake of G6P from the cytosol into the endoplasmic reticulum for its conversion into the central fueling metabolite, glucose. Hpt mimics the function of the mammalian G6PT to steal fueling metabolites from host cell cytosol for the benefit of the microbe.	setA	Listeria ivanovii subsp. ivanovii PAM 55	Nutritional/Metabolic factor	hpt	hexose phosphate transport protein	23.1	6.64E-10	60.8
ECs_3125	glpT	sn-glycerol-3-phos	VFG032173 (gb|WP_01	Hpt (VF0264)	The mammalian G6PT is responsible for the uptake of G6P from the cytosol into	setA	Listeria ivanovii	Nutritional/Metaboli	hpt	hexose phosphate	28.7	4.96E-50	176

		phate transporter	4092267)		the endoplasmic reticulum for its conversion into the central fueling metabolite, glucose. Hpt mimics the function of the mammalian G6PT to steal fueling metabolites from host cell cytosol for the benefit of the microbe.		subsp. ivanovii PAM 55	c factor		transport protein			
ECs_4603	uhpT	hexose phosphate transporter	VFG032173 (gb|WP_014092267)	Hpt (VF0264)	The mammalian G6PT is responsible for the uptake of G6P from the cytosol into the endoplasmic reticulum for its conversion into the central fueling metabolite, glucose. Hpt mimics the function of the mammalian G6PT to steal fueling metabolites from host cell cytosol for the benefit of the microbe.	setA	Listeria ivanovii subsp. ivanovii PAM 55	Nutritional/Metabolic factor	hpt	hexose phosphate transport protein	46.6	4.20E-132	389
ECs_4604	uhpC	regulatory protein	VFG032173 (gb|WP_014092267)	Hpt (VF0264)	The mammalian G6PT is responsible for the uptake of G6P from the cytosol into the endoplasmic reticulum for its conversion into the central fueling metabolite, glucose. Hpt mimics the function of the mammalian G6PT to steal fueling metabolites from host cell cytosol for the benefit of the microbe.	setA	Listeria ivanovii subsp. ivanovii PAM 55	Nutritional/Metabolic factor	hpt	hexose phosphate transport protein	29.3	1.33E-51	180
ECs_3975	exuT	hexuronate transporter	VFG032174 (gb|WP_012985162)	Hpt (VF0264)	The mammalian G6PT is responsible for the uptake of G6P from the cytosol into the endoplasmic reticulum for its conversion into the central fueling	setB	Listeria seeligeri serovar 1/2b str.	Nutritional/Metabolic factor	hpt	hexose phosphate transport protein	25.1	1.96E-10	62.4

					metabolite, glucose. Hpt mimics the function of the mammalian G6PT to steal fueling metabolites from host cell cytosol for the benefit of the microbe.		SLCC3954						
ECs_4397	gadA_2	glutamate decarboxylase B	VFG041318 (gb|WP_010947885)	Dot/Icm T4SS secreted effectors (VF0798)	AnkB/legAU13/ceg27 (E3 Ubiquitin Ligase Activity, bounds Skp1, targets host protein parvin B.); AnkC/legA12 (Ankyrin repeat.); AnkD/legA15 (Ankyrin repeat.); AnkF/legA14/ceg31 (Ankyrin repeat.); AnkG/ankZ/legA7 (Ankyrin repeat.); AnkH/legA3/ankW (Ankyrin repeat, NF-<kappa>B inhibitor.); AnkI/legAS4 (Ankyrin repeat.); AnkJ/legA11 (Ankyrin repeat.); AnkK/legA5 (Ankyrin repeat.); AnkN/ankX/legA8 (Phosphocholination of Rab1 and Rab35 to regulate their activity; modulation of endosomal trafficking.); AnkQ/legA10; AnkY/legA9 (Ankyrin repeat, STPK, Enhancer of autophagy.); Ceg10; Ceg14/sidL (Inhibition of host protein synthesis leading to activation of the NF-<kappa>B pathway.); Ceg15; Ceg17; Ceg18; Ceg19 (Vesicle trafficking.);	setA	Legionella pneumophila subsp. pneumophila str. Philadelphia 1	Effector delivery system	legS2	Dot/Icm type IV secretion system effector LegS2, targeting mitochondria	23.7	4.66E-09	58.5

Ceg23; Ceg25; Ceg28; Ceg29; Ceg3; Ceg30; Ceg32/sidI (Interacts with eEF1A to inhibit host protein synthesis.); Ceg33; Ceg34; Ceg4; Ceg5; Ceg7; Ceg8; Ceg9 (Vesicle trafficking.); CegC1 (Zinc metallophospholipase C. Zinc metallophospholipase C.); CegC2 (Ninein domain.); CegC3; CegC4; DrrA/sidM (Rab1-GEF and GDF (RabGDI displacement factor) activity responsible for Rab1 recruitment to LCV, C-terminal PI4P binding domain responsible for membrane binding; N-terminal AMPylation activity.); LaiE (SidE paralog.); LegA1; LegA2; LegA6; LegA7; LegC1; LegC3/ppeA (Vesicle trafficking.); LegC4 (Coiled-coil.); LegC6 (Coiled-coil.); LegD1; LegD2; LegG2 (Ras GEF.); LegK1 (Eukaryotic-like Ser/Thr kinase activity, directly activates NF-<kappa>B pathway by phosphorylating the I<kappa>B family of inhibitors.); LegK2 (Ser/Thr kinase.); LegK3 (STPK.); LegL1

(Leucine-rich repeats.); LegL2
(Leucine-rich repeats.); LegL3
(Leucine-rich repeats.); LegL5
(Leucine-rich repeats.); LegL6
(Leucine-rich repeats.); LegL7
(Leucine-rich repeats.); LegLC4
(Leucine-rich repeats,
coiled-coil.); LegLC8 (Leucine-rich
repeats, coiled-coil.); LegN; LegP
(Astacin protease.); LegS1; LegS2
(Putative Sphingosine-1-phosphate
lyase 1 (SP-lyase).); LegT (Thaumatin
domain.); LegU1 (E3 Ubiquitin Ligase,
targets host chaperone protein
BAT3.); LegY; Lem1; Lem10; Lem11;
Lem12; Lem14; Lem15; Lem16; Lem17;
Lem19; Lem2; Lem20; Lem21; Lem22;
Lem23; Lem24; Lem25; Lem26; Lem27;
Lem28; Lem29; Lem3
(Dephosphoryl-cholinase relieving the
AnkX-mediated modification on Rab1.);
Lem4/smdA (PI4P-binding protein.);
Lem5; Lem6; Lem7; Lem8; Lem9; LepA
(Nonlytic release from protozoa.);
LepB (Rab1 GAP, vesicle trafficking
and bacterial egress.); Lgt2/legC8

(Glucosyltransferase, inhibits host protein systhesis by glucosylating mammalian elongation factor eEF1A at serine-53.); Lgt3/legC5 (Glucosyltransferase, inhibits host protein systhesis by glucosylating mammalian elongation factor eEF1A at serine-53.); LidA (Promotion of Rab1 recruitment and tethering of ER derived vesicles to the LCV; stabilization of Rab guanosine nucleotide complex.); LidL (EnhC paralogue.); LirA; LirB (Peptidyl-prolyl cis-trans isomerase A (rotamase A).); Lpg0045; Lpg0081; Lpg0294; Lpg0365; Lpg0518; Lpg0634; Lpg0963; Lpg1148; Lpg1158; Lpg1273; Lpg1689; Lpg1717; Lpg1751; Lpg2160 (Associates with BAT3 independently of LegU1; LegU1 and Lpg2160 may function redundantly or in concert to modulate BAT3 activity during the course of infection.); Lpg2327; Lpg2407; Lpg2525 (F-box protein.); Lpg2527; Lpg2744; LpnE (Putative Beta-lactamase.); LubX/legU2 (E3

ubiquitin ligase, targets another effector protein SidH to proteasome-mediated protein degradation in the host cells; cell cycle modulation via Clk1.); MavA; MavB; MavC; MavE; MavF; MavG; MavH; MavI; MavJ; MavL; MavM; MavN; MavV; PieA/lirC; PieB/lirD; PieC/lirE; PieD/lirF; PieE; PieF; PieG/legG1 (Regulator of chromosome condensation RCC.); PpeB; PpgA (Regulator of chromosome condensation.); RalF (Arf-GEF; Arf1 recuitment to LCV.); RavE; RavF; RavG; RavH; RavI; RavJ; RavK; RavL; RavM; RavN; RavO; RavP; RavQ; RavR; RavS; RavT; RavW; RavX; RavY; RavZ (Cysteine protease. Inhibits host autophagy by cleaving and deconjugating LC3-PE.); RvfA; SdbA (Contributes to sustained NF-<kappa>B activation.); SdbB (SidB paralog.); SdbC (SidB paralog.); SdcA (SidC paralog, anchors to PtdIns(4)P on LCVs.); SdeA/laiA (Adherence and/or uptake.); SdeB/laiB; SdeC/laiC; SdeD/laiF (SidE paralog.);

SdhA (Maintenance of LCV integrity preventing cell death and type I interferon induction.); SdhB (Paralog of sidH, ANTH domain.); SdjA; SetA (Vesicle trafficking.); SidA; SidB (Rtx toxin, lipase.); SidC (ER recuitment.); SidD; SidE/laiD; SidF (Anti-apoptosis by targeting pro-death members of the Bcl2 protein family.); SidG (Coiled-coil.); SidH (A substrate of LubX E3 ubiquitin ligase.); SidJ (ER recuitment.); SidK (Interacting with VatA, a key component of the proton pump. Inhibition of LCV acidification.); VipA (Actin nucleator contributing to modulate organelle trafficking.); VipD (Phospholipase A1, removes PI(3)P from early endosomes.); VipE; VipF (N-terminal acetyltransferase, GNAT family.); VpdA/vipD2 (VipD paralog, Acyl transferase/acyl hydrolase/lysophospholipase.); VpdB/vipD3 (VipD paralog, phospholipase.); VpdC; WipA; WipB; YlfA/legC7 (Vesicle trafficking.);

YlfB/legC2 (Vesicle trafficking.);

CegC4; MesI; LPG_RS00040;

LPG_RS00105; LPG_RS00150;

LPG_RS00200; LPG_RS00235;

LPG_RS00300; LPG_RS00665

(PI-3-phosphatase.); LPG_RS00825;

LPG_RS00870; LPG_RS00880;

LPG_RS00925; LPG_RS01290;

LPG_RS01305; LPG_RS01820;

LPG_RS01880; LPG_RS02025;

LPG_RS03550; LPG_RS03935;

LPG_RS04800; LPG_RS05365;

LPG_RS05490; LPG_RS05590;

LPG_RS05660; LPG_RS05710;

LPG_RS05830; LPG_RS07260;

LPG_RS07280; LPG_RS07435;

LPG_RS07905; LPG_RS08210;

LPG_RS08285; LPG_RS08320;

LPG_RS08345; LPG_RS08350;

LPG_RS08370; LPG_RS08445;

LPG_RS08450; LPG_RS08485;

LPG_RS08595; LPG_RS08775;

LPG_RS08895; LPG_RS09040;

LPG_RS09470; LPG_RS09565;

LPG_RS09650; LPG_RS09825;

LPG_RS09915; LPG_RS09965;

					LPG_RS10290; LPG_RS10795; LPG_RS10800; LPG_RS11170; LPG_RS11255; LPG_RS11415; LPG_RS11860; LPG_RS11920; LPG_RS11940; LPG_RS11990; LPG_RS12190; LPG_RS12265; LPG_RS12310; LPG_RS12405; LPG_RS12815; LPG_RS12820; LPG_RS12830; LPG_RS12855; LPG_RS12885; LPG_RS12900; LPG_RS13265; LPG_RS13310; LPG_RS13590; LPG_RS13860; LPG_RS14265; LPG_RS14285; LPG_RS14345; LPG_RS14500; LPG_RS14525; LPG_RS14550; LPG_RS14555; LPG_RS14570; LPG_RS14710; LPG_RS14840; LPG_RS15050; LPG_RS15170								
ECs_2098	gadA	glutamate decarboxylase B	VFG041318 (gb|WP_010947885)	Dot/Icm T4SS secreted effectors (VF0798)	AnkB/legAU13/ceg27 (E3 Ubiquitin Ligase Activity, bounds Skp1, targets host protein parvin B.); AnkC/legA12 (Ankyrin repeat.); AnkD/legA15 (Ankyrin repeat.); AnkF/legA14/ceg31 (Ankyrin repeat.); AnkG/ankZ/legA7 (Ankyrin repeat.); AnkH/legA3/ankW (Ankyrin repeat, NF-<kappa>B	setA	Legionella pneumophila subsp. pneumophila str. Philadelphia 1	Effector delivery system	legS2	Dot/Icm type IV secretion system effector LegS2, targeting mitochondr	23.7	4.66E-09	58.5

inhibitor.); AnkI/legAS4 (Ankyrin repeat.); AnkJ/legA11 (Ankyrin repeat.); AnkK/legA5 (Ankyrin repeat.); AnkN/ankX/legA8 (Phosphocholination of Rab1 and Rab35 to regulate their activity; modulation of endosomal trafficking.); AnkQ/legA10; AnkY/legA9 (Ankyrin repeat, STPK, Enhancer of autophagy.); Ceg10; Ceg14/sidL (Inhibition of host protein synthesis leading to activation of the NF-<kappa>B pathway.); Ceg15; Ceg17; Ceg18; Ceg19 (Vesicle trafficking.); Ceg23; Ceg25; Ceg28; Ceg29; Ceg3; Ceg30; Ceg32/sidI (Interacts with eEF1A to inhibit host protein synthesis.); Ceg33; Ceg34; Ceg4; Ceg5; Ceg7; Ceg8; Ceg9 (Vesicle trafficking.); CegC1 (Zinc metallophospholipase C. Zinc metallophospholipase C.); CegC2 (Ninein domain.); CegC3; CegC4; DrrA/sidM (Rab1-GEF and GDF (RabGDI displacement factor) activity responsible for Rab1 recruitment to

ia

LCV, C-terminal PI4P binding domain responsible for membrane binding; N-terminal AMPylation activity.); LaiE (SidE paralog.); LegA1; LegA2; LegA6; LegA7; LegC1; LegC3/ppeA (Vesicle trafficking.); LegC4 (Coiled-coil.); LegC6 (Coiled-coil.); LegD1; LegD2; LegG2 (Ras GEF.); LegK1 (Eukaryotic-like Ser/Thr kinase activity, directly activates NF-<kappa>B pathway by phosphorylating the I<kappa>B family of inhibitors.); LegK2 (Ser/Thr kinase.); LegK3 (STPK.); LegL1 (Leucine-rich repeats.); LegL2 (Leucine-rich repeats.); LegL3 (Leucine-rich repeats.); LegL5 (Leucine-rich repeats.); LegL6 (Leucine-rich repeats.); LegL7 (Leucine-rich repeats.); LegLC4 (Leucine-rich repeats, coiled-coil.); LegLC8 (Leucine-rich repeats, coiled-coil.); LegN; LegP (Astacin protease.); LegS1; LegS2 (Putative Sphingosine-1-phosphate lyase 1 (SP-lyase).); LegT (Thaumatin

domain.); LegU1 (E3 Ubiquitin Ligase, targets host chaperone protein BAT3.); LegY; Lem1; Lem10; Lem11; Lem12; Lem14; Lem15; Lem16; Lem17; Lem19; Lem2; Lem20; Lem21; Lem22; Lem23; Lem24; Lem25; Lem26; Lem27; Lem28; Lem29; Lem3 (Dephosphoryl-cholinase relieving the AnkX-mediated modification on Rab1.); Lem4/smdA (PI4P-binding protein.); Lem5; Lem6; Lem7; Lem8; Lem9; LepA (Nonlytic release from protozoa.); LepB (Rab1 GAP, vesicle trafficking and bacterial egress.); Lgt2/legC8 (Glucosyltransferase, inhibits host protein systhesis by glucosylating mammalian elongation factor eEF1A at serine-53.); Lgt3/legC5 (Glucosyltransferase, inhibits host protein systhesis by glucosylating mammalian elongation factor eEF1A at serine-53.); LidA (Promotion of Rab1 recruitment and tethering of ER derived vesicles to the LCV; stabilization of Rab guanosine nucleotide complex.); LidL (EnhC

paralogue.); LirA; LirB (Peptidyl-prolyl cis-trans isomerase A (rotamase A).); Lpg0045; Lpg0081; Lpg0294; Lpg0365; Lpg0518; Lpg0634; Lpg0963; Lpg1148; Lpg1158; Lpg1273; Lpg1689; Lpg1717; Lpg1751; Lpg2160 (Associates with BAT3 independently of LegU1; LegU1 and Lpg2160 may function redundantly or in concert to modulate BAT3 activity during the course of infection.); Lpg2327; Lpg2407; Lpg2525 (F-box protein.); Lpg2527; Lpg2744; LpnE (Putative Beta-lactamase.); LubX/legU2 (E3 ubiquitin ligase, targets another effector protein SidH to proteasome-mediated protein degradation in the host cells; cell cycle modulation via Clk1.); MavA; MavB; MavC; MavE; MavF; MavG; MavH; MavI; MavJ; MavL; MavM; MavN; MavV; PieA/lirC; PieB/lirD; PieC/lirE; PieD/lirF; PieE; PieF; PieG/legG1 (Regulator of chromosome condensation RCC.); PpeB; PpgA (Regulator of chromosome condensation.); RalF

(Arf-GEF; Arf1 recuitment to LCV.);
RavE; RavF; RavG; RavH; RavI; RavJ;
RavK; RavL; RavM; RavN; RavO; RavP;
RavQ; RavR; RavS; RavT; RavW; RavX;
RavY; RavZ (Cysteine protease.
Inhibits host autophagy by cleaving
and deconjugating LC3-PE.); RvfA;
SdbA (Contributes to sustained
NF-<kappa>B activation.); SdbB (SidB
paralog.); SdbC (SidB paralog.); SdcA
(SidC paralog, anchors to PtdIns(4)P
on LCVs.); SdeA/laiA (Adherence
and/or uptake.); SdeB/laiB;
SdeC/laiC; SdeD/laiF (SidE paralog.);
SdhA (Maintenance of LCV integrity
preventing cell death and type I
interferon induction.); SdhB (Paralog
of sidH, ANTH domain.); SdjA; SetA
(Vesicle trafficking.); SidA; SidB
(Rtx toxin, lipase.); SidC (ER
recuitment.); SidD; SidE/laiD; SidF
(Anti-apoptosis by targeting
pro-death members of the Bcl2 protein
family.); SidG (Coiled-coil.); SidH
(A substrate of LubX E3 ubiquitin
ligase.); SidJ (ER recuitment.); SidK

(Interacting with VatA, a key
component of the proton pump.
Inhibition of LCV acidification.);
VipA (Actin nucleator contributing to
modulate organelle trafficking.);
VipD (Phospholipase A1, removes PI(3)P
from early endosomes.); VipE; VipF
(N-terminal acetyltransferase, GNAT
family.); VpdA/vipD2 (VipD paralog,
Acyl transferase/acyl
hydrolase/lysophospholipase.);
VpdB/vipD3 (VipD paralog,
phospholipase.); VpdC; WipA; WipB;
YlfA/legC7 (Vesicle trafficking.);
YlfB/legC2 (Vesicle trafficking.);
CegC4; MesI; LPG_RS00040;
LPG_RS00105; LPG_RS00150;
LPG_RS00200; LPG_RS00235;
LPG_RS00300; LPG_RS00665
(PI-3-phosphatase.); LPG_RS00825;
LPG_RS00870; LPG_RS00880;
LPG_RS00925; LPG_RS01290;
LPG_RS01305; LPG_RS01820;
LPG_RS01880; LPG_RS02025;
LPG_RS03550; LPG_RS03935;
LPG_RS04800; LPG_RS05365;

LPG_RS05490; LPG_RS05590;

LPG_RS05660; LPG_RS05710;

LPG_RS05830; LPG_RS07260;

LPG_RS07280; LPG_RS07435;

LPG_RS07905; LPG_RS08210;

LPG_RS08285; LPG_RS08320;

LPG_RS08345; LPG_RS08350;

LPG_RS08370; LPG_RS08445;

LPG_RS08450; LPG_RS08485;

LPG_RS08595; LPG_RS08775;

LPG_RS08895; LPG_RS09040;

LPG_RS09470; LPG_RS09565;

LPG_RS09650; LPG_RS09825;

LPG_RS09915; LPG_RS09965;

LPG_RS10290; LPG_RS10795;

LPG_RS10800; LPG_RS11170;

LPG_RS11255; LPG_RS11415;

LPG_RS11860; LPG_RS11920;

LPG_RS11940; LPG_RS11990;

LPG_RS12190; LPG_RS12265;

LPG_RS12310; LPG_RS12405;

LPG_RS12815; LPG_RS12820;

LPG_RS12830; LPG_RS12855;

LPG_RS12885; LPG_RS12900;

LPG_RS13265; LPG_RS13310;

LPG_RS13590; LPG_RS13860;

					LPG_RS14265; LPG_RS14285; LPG_RS14345; LPG_RS14500; LPG_RS14525; LPG_RS14550; LPG_RS14555; LPG_RS14570; LPG_RS14710; LPG_RS14840; LPG_RS15050; LPG_RS15170								
ECs_0890	dps	Fe-binding and storage protein	VFG006371 (gb|WP_000846480)	HP-NAP (VF0052)	H.pylori Neutrophil activating protein, promoting the adhesion of human neutrophils to endothelial cells and the production of reactive oxygen radicals; inducing a very moderate state of inflammation that would promote H. pylori growth by the release of nutrient factors from the inflamed tissue	setA	Helicobacter pylori J99	Immune modulation	napA	neutrophil activating protein NapA	25.5	1.52E-08	50.8
ECs_4571	espZ	T3SS secreted effector EspZ	VFG000815 (gb|WP_000386949)	TTSS secreted effectors (VF1110)	Cif (Deamidase. Induces cytopathic effects of actin stress fiber formation and cell cycle arrest.); EspB (Pore formation, actin disruption, microvilli effacement, anti-phagocytosis.); EspF (Inducing degradation of the aniapoptic protein AbcF2, tight junction disruption, microvilli effacement and elongation, mitochondrial dysfunction, N-WASP activation, SGLT-1 inactivation,	setA	Escherichia coli O157:H7 str. EDL933	Effector delivery system	sepZ/espZ	Type III secretion system effector EspZ	100	3.43E-59	176

pedestal maturation, inhibition of NHE3 activity, membrane remodelling; targets and disrupts the nucleolus late in infection, which is temporally controlled by host mitochondria.); EspFu/tccP (Inducing degradation of the aniapoptic protein AbcF2, tight junction disruption, microvilli effacement and elongation, mitochondrial dysfunction, N-WASP activation, SGLT-1 inactivation, pedestal maturation, inhibition of NHE3 activity, membrane remodelling; targets and disrupts the nucleolus late in infection, which is temporally controlled by host mitochondria.); EspG (TBC-like GTPase activating protein. Efficiently catalyzes GTP hydrolysis in Rab1 to disrupt of Rab1-mediated ER-to-Golgi trafficking.); EspH (First bacterial effector acting directly on RhoGEFs, EspH directly binds to the DH-PH domain in RhoGEFs to disrupt RhoGEF-Rho signaling; critical for inhibiting macrophage phagocytosis.); EspJ

(Inhibit both IgG- and complement receptor-mediated phagocytosis.); EspK; EspL1; EspL2 (Cysteine protease. Bounds F-actin-aggregating annexin 2 directly to increase annexin 2's ability to aggregate Tir-induced F-actin; block necroptosis and in flammation.); EspL4; EspM1 (GEF. Activates the RhoA signaling pathway and induce the formation of stress fibres; inhibit pedestal formation and induce tight junction mislocalization.); EspM2 (GEF. Activates the RhoA signaling pathway and induce the formation of stress fibres; inhibit pedestal formation and induce tight junction mislocalization.); EspN; EspO1-1; EspO1-2; EspR1; EspR3; EspR4; EspT (GEF. Activates Rac1 and Cdc42 leading to formation of membrane ruffles and lamellipodia; induces membrane ruffles to facilitate bacterial invasion into non-phagocytic cells in a process involving Rac1 and Wave2.); EspW; EspX1; EspX2; EspX4; EspX5;

EspX6; EspX7/nleL (E3 ubiquitin ligase, HECT-like. Modulates pedestal formation.); EspY1; EspY2; EspY3; EspY4; EspY5; Map (GEF. Mimics the host Dbl and catalyses the exchange of GDP for GTP in Cdc42, involved in effacement, SGLT1 inhibition, formation of filopodia and disruption of mitochondrial function.); NleA/espI (Disruption of tight junctions by inhibition of host cell protein trafficking through COPII-dependent pathways.); NleB1 (Blocks translocation of the p65 and to the host cell nucleus to inhibit NF-<kappa>B pathway, but NleE and NleB act at different points in the NF-<kappa>B signaling pathway.); NleB2 (May also have anti-inflammatory activity.); NleC (Metalloprotease. Zn-dependent endopeptidases that specifically clip and inactivate RelA (p65), thus blocking NF-<kappa>B pathway.); NleD (Metalloprotease. Zn-dependent endopeptidases that specifically clip and inactivate JNK

and p38, thus blocking AP-1 pathway.); NleE (PMN tran-epithelial migration; blocks translocation of the p65 to the host cell nucleus by preventing I<kappa>B degradation to inhibit NF-<kappa>B pathway.); NleF; NleG-1; NleG2-2; NleG2-3; NleG2-4; NleG5-1; NleG5-2; NleG6-1; NleG6-2; NleG6-3; NleG7 (U-box type E3 ubiquitin ligases.); NleG8-2; NleH1 (Ser/Thr protein kinase. Binds directly to a subunit of NF-<kappa>B, the ribosomal protein S3 (RPS3), reducing the nuclear abundance of RPS3 to dampen host transcriptional outputs; interact with Bax inhibitor-1 to block apoptosis.); NleH2 (Putative kinase. Attenuates NF-<kappa>B pathway.); SepZ/espZ (EspZ interacts with CD98 in host cell membranes to promote host cell survival, therefore provide the pathogen with valuable time to colonize efficiently prior to dissemination.); TccP2; Tir (Mimics host immunoreceptor tyrosine-based inhibition motifs (ITIMs), also see

					helicobacter CagA. EHEC Tir lacks the Nck binding site. Conserved NPY (Asn-Pro-Tyr) motif recruits the adaptor protein IRTKS and/or IRSp53. IRTKS/IRSp53 link Tir and TccP/EspFu, which in turn activates N-WASP; Receptor for intimin; effacement; SGLT1 inhibition; recruits SHIP2 to control actin-pedestal morphology; maintains the integrity of the epithelium by keeping the destructive activity of EspG and EspG2 in check.)								
ECs_4574	sepD	T3SS secretion switching protein SepD	VFG000818 (gb|WP_001063681)	TTSS (VF0191)	Injects Tir and other effector molecules directly into the host cell. Effector molecules activate cell-signaling pathways, causing alterations in the host cell cytoskeleton and resulting in the depolymerization of actin and the loss of microvilli	setA	Escherichia coli O157:H7 str. EDL933	Effector delivery system	sepD	Type III secretion system secretion switch protein SepD	100	6.91E-113	316
ECs_4575	escC	T3SS structure protein EscC	VFG000819 (gb|WP_000723942)	TTSS (VF0191)	Injects Tir and other effector molecules directly into the host cell. Effector molecules activate cell-signaling pathways, causing alterations in the host cell cytoskeleton and resulting in the	setA	Escherichia coli O157:H7 str. EDL933	Effector delivery system	escC	Type III secretion system secretin EscC	100	0	949

					depolymerization of actin and the loss of microvilli								
ECs_0385	prpB	2-methylisocitrate lyase	VFG017665 (gb|WP_005062139)	Isocitrate lyase (VF0253)	Required for persistent infection	setB	Mycobacterium abscessus subsp. bolletii 50594	Others	icl	Isocitrate lyase Icl (isocitrase) (isocitratase)	29.1	4.19E-17	80.9
ECs_0384	prpR	propionate catabolism operon regulatory protein	VFG050705 (gb|WP_00840555.1)	TFP (VF1334)	Essential for twitching motility and natural competence, and contribute to host cell adherence	setA	Acinetobacter baumannii D1279779	Adherence	pilR	sigma-54 dependent transcriptional regulator	42.2	4.48E-67	224
ECs_4265	rtcR	transcriptional regulatory protein RtcR	VFG050705 (gb|WP_00840555.1)	TFP (VF1334)	Essential for twitching motility and natural competence, and contribute to host cell adherence	setA	Acinetobacter baumannii D1279779	Adherence	pilR	sigma-54 dependent transcriptional regulator	32.1	2.23E-39	149
ECs_4553	cesD	T3SS chaperone CesD2	VFG000797 (gb|WP_000228583)	TTSS (VF0191)	Injects Tir and other effector molecules directly into the host cell. Effector molecules activate cell-signaling pathways, causing alterations in the host cell cytoskeleton and resulting in the depolymerization of actin and the loss	setA	Escherichia coli O157:H7 str. EDL933	Effector delivery system	cesD2	chaperone for EspD	100	2.52E-96	273

					of microvilli								
ECs_0257	mbhA	flagellar system protein MbhA	VFG043246 (gb|WP_002211411)	Lateral flagella (VF1028)	-	setA	Yersinia pestis C092	Others	lafU	putative lateral flagellar export/assembly protein LafU	58.9	1.69E-68	211
ECs_0256	flhA	flagellar export pore protein	VFG038861 (gb|WP_005314296)	Lateral flagella (VF0474)	Inducible lateral peritrichous flagellar system is responsible for movement across solid surfaces or through viscous environments known as swarming motility; glycosylation of the lateral flagellin is essential for swarming motility over surfaces	setA	Aeromonas salmonicida subsp. salmonicida A449	Motility	lfhA	lateral flagellar biosynthesis protein	54.6	3.06E-213	609
ECs_0254	yafL	endopeptidase	VFG050290 (gb|WP_010934947)	DIP1281 (VF0602)	Involved in cell surface organization, adhesion and internalization in epithelial cells	setA	Corynebacterium diphtheriae NCTC 13129	Adherence	DIP_RS17590	C40 family peptidase	33.3	2.18E-13	69.3
ECs_2415	nlpC	endopeptidase	VFG043441 (gb|WP_002287386)	Fibronectin-binding protein (VF0747)	-	setA	Enterococcus faecium U0317	Adherence	EFMU0317_RS16950	C40 family peptidase	36.7	1.99E-08	52
ECs_4809	yihY	inner membrane	VFG011133 (gb|WP_01	Brk (VF0034)	Outer membrane protein that mediates adherence and resists complement	setA	Bordetella parapertus	Immune modulation	brkB	serum resistance	24.8	8.71E-08	52.4

		protein	0927719)				sis 12822			protein			
ECs_2019	ECs_2019	hypothetical protein	VFG041175 (gb|WP_000622532)	T6SS (VF0975)	-	setB	Salmonella enterica subsp. enterica serovar Gallinarum str. 287/91	Effector delivery system	SG_RS05320	hypothetical protein	47.2	1.55E-34	116
ECs_0963	ybjX	hypothetical protein	VFG013079 (gb|WP_004970430)	VirK (VF0980)	-	setB	Shigella dysenteriae Sd197	Others	virK	virulence factor VirK	38.7	2.78E-49	167
ECs_2011	ynbB	CDP-diglyceride synthase	VFG002189 (gb|WP_002359680)	Capsule (VF0361)	Contributes to host immune evasion	setA	Enterococcus faecalis V583	Immune modulation	cpsB/cdsA	phosphatidate cytidylyltransferase	41.1	2.67E-25	101
ECs_0177	cdsA	CDP-diglyceride synthase	VFG002189 (gb|WP_002359680)	Capsule (VF0361)	Contributes to host immune evasion	setA	Enterococcus faecalis V583	Immune modulation	cpsB/cdsA	phosphatidate cytidylyltransferase	32.7	1.92E-30	114
ECs_0879	ybiC	dehydrogenase	VFG049124 (gb|WP_012737540)	Allantion utilization (VF0572)	Providing a nitrogen source to increase virulence in K. pneumoniae at certain sites of infection	setA	Klebsiella pneumoniae subsp. pneumoniae NTUH-K2044	Nutritional/Metabolic factor	allD	ureidoglycolate dehydrogenase	32	1.71E-30	119
ECs_0579	allD	ureidoglycolate	VFG049124 (gb|WP_01	Allantion utilizati	Providing a nitrogen source to increase virulence in K. pneumoniae at	setA	Klebsiella pneumoniae	Nutritional/Metaboli	allD	ureidoglycolate	83.4	2.33E-216	594

		dehydrogenase	2737540)	on (VF0572)	certain sites of infection		subsp. pneumoniae NTUH-K2044	c factor		dehydrogenase			
ECs_3380	ndk	multifunctional nucleoside diphosphate kinase and apyrimidinic endonuclease and 3'-phosphodiesterase	VFG031464 (gb|WP_011893513)	Nucleoside diphosphate kinase (VF0851)	-	setB	Mycobacterium gilvum PYR-GCK	Immune modulation	ndk	nucleoside-diphosphate kinase	48.9	1.47E-37	124
ECs_0965	macB	macrolide ABC transporter permease/ATPase	VFG015379 (gb|WP_011979124)	HSI-1 (VF0334)	Play role in chronic P. aeruginosa infections	setA	Pseudomonas aeruginosa PA7	Effector delivery system	tagT	type VI secretion associated protein TagT, ATP-binding component of ABC transporte	40.1	1.72E-32	125

										r			
ECs_1684	dadA	D-amino acid dehydrogenase	VFG015927 (gb|WP_012075860)	Hydrogen cyanide production (VF0920)	-	setB	Pseudomonas aeruginosa PA7	Antimicrobial activity/Competitive advantage	hcnC	cyanide-forming glycine dehydrogenase subunit HcnC	24.1	3.86E-08	55.1
ECs_3222	yfcV	fimbrial-like adhesin protein	VFG021557 (gb|WP_001090556)	Stf (VF0958)	-	setB	Salmonella enterica subsp. enterica serovar Enteritidis str. P125109	Adherence	stfA	fimbrial major subunit StfA	38.7	3.82E-27	101
ECs_2839	gmd	GDP-D-mannose dehydratase	VFG048885 (gb|WP_014907227)	Capsule (VF0560)	Assisting in evading the host immune system by protecting bacteria from opsonophagocytosis and serum killing	setA	Klebsiella pneumoniae subsp. pneumoniae NTUH-K2044	Immune modulation	gmd	GDP-mannose 4,6-dehydratase	85.9	6.50E-242	660
ECs_2858	gmd	GDP-D-mannose dehydratase	VFG048885 (gb|WP_014907227)	Capsule (VF0560)	Assisting in evading the host immune system by protecting bacteria from opsonophagocytosis and serum killing	setA	Klebsiella pneumoniae subsp. pneumoniae NTUH-K2044	Immune modulation	gmd	GDP-mannose 4,6-dehydratase	88.9	1.63E-249	680
ECs_2838	wcaG	GDP-L-fuc	VFG048868	Capsule	Assisting in evading the host immune	setA	Klebsiella	Immune	A79E_RS082	GDP-L-fuco	73	3.75E-	490

		ose synthetase	(gb|WP_014907228)	(VF0560)	system by protecting bacteria from opsonophagocytosis and serum killing		pneumoniae subsp. pneumoniae 1084	modulation	35	se synthase		176	
ECs_2857	wcaG	GDP-L-fucose synthetase	VFG048868 (gb|WP_014907228)	Capsule (VF0560)	Assisting in evading the host immune system by protecting bacteria from opsonophagocytosis and serum killing	setA	Klebsiella pneumoniae subsp. pneumoniae 1084	Immune modulation	A79E_RS08235	GDP-L-fucose synthase	78	4.99E-188	520
ECs_2389	sufC	SufBCD Fe-S cluster assembly scaffold protein	VFG013715 (gb|WP_010934432)	ABC-type heme transporter (VF0739)	-	setA	Corynebacterium diphtheriae NCTC 13129	Nutritional/Metabolic factor	hmuV	heme ABC transporter ATP-binding protein	29.1	6.88E-17	77.8
ECs_5381	ybdZ	hypothetical protein	VFG009450 (gb|WP_011893579)	Mycobactin (VF0299)	Cell-association mycobactin participates in iron internalization and/or to serve as a temporary iron-holding molecule to prevent sudden influx of excess iron if the metal suddenly becomes available after a period of iron limitation	setA	Mycobacterium gilvum PYR-GCK	Nutritional/Metabolic factor	mbtH	putative protein MbtH	38.2	9.57E-15	62
ECs_2634	stbA	plasmid partition protein	VFG017868 (gb|WP_041175668)	Icm/dot type IVB locus (VF1026)	-	setB	Yersinia pseudotuberculosis IP 31758	Effector delivery system	YPSIP31758_RS00975	ParM/StbA family protein	21.7	2.29E-08	54.7

ECs_3726	epaO	type III secretion protein EpaO	VFG042083 (gb|NP_311753)	ETT2 (VF1161)	-	setA	Escherichia coli O157:H7 str. Sakai	Effector delivery system	epaO	type III secretion protein EpaO	100	6.64E-242	657
ECs_3727	eivJ2	type III secretion protein	VFG042082 (gb|NP_311754)	ETT2 (VF1161)	-	setA	Escherichia coli O157:H7 str. Sakai	Effector delivery system	eivJ2	type III secretion protein	100	1.78E-129	361
ECs_3724	epaQ	type III secretion protein EpaQ	VFG042085 (gb|NP_311751)	ETT2 (VF1161)	-	setA	Escherichia coli O157:H7 str. Sakai	Effector delivery system	epaQ	type III secretion protein EpaQ	100	3.71E-57	170
ECs_3725	escR	T3SS structure protein EscR	VFG042084 (gb|NP_311752)	ETT2 (VF1161)	-	setA	Escherichia coli O157:H7 str. Sakai	Effector delivery system	escR	T3SS structure protein EscR	100	1.71E-145	404
ECs_3722	epaR2	type III secretion protein EpaR	VFG018530 (gb|WP_000498972)	TTSS (SPI-1 encode) (VF0116)	Delivers at least 13 effector proteins through the host cell plasma membrane, most of them are involved in actin cytoskeleton rearrangements, leading to membrane ruffling and Salmonella invasion	setA	Salmonella enterica subsp. arizonae serovar 62:z4,z23:-- str. RSK2980	Effector delivery system	spaR	type III secretion system minor export apparatus protein SpaR	58.5	4.54E-17	72.4
ECs_3723	epaR1	type III secretion	VFG041854 (gb|WP_01	Cpi-1a + Cpi-1	-	setA	Chromobacterium	Effector delivery	CV_RS12835	SpaR/YscT/HrcT type	46.5	6.09E-40	134

		protein EpaR	1136169)	(SPI-1 like) (VF1259)			violaceum ATCC 12472	system		III secretion system export apparatus protein			
ECs_5093	proP	proline/glycine betaine transporter	VFG045330 (gb|WP_010947054)	Legiobactin (VF0291)	A nonhydroxamate, nonphenolate siderophore	setA	Legionella pneumophila subsp. pneumophila str. Philadelphia 1	Nutritional/Metabolic factor	lbtC	LbtC inner membrane protein required for import of Legiobactin	25.3	3.16E-06	49.3
ECs_2883	mdtC_1	multidrug efflux system subunit B	VFG037720 (gb|WP_001027056)	AdeFGH efflux pump (VF0504)	Play a potential role in the synthesis and transport of autoinducer molecules during biofilm formation	setA	Acinetobacter baumannii ACICU	Biofilm	adeG	cation/multidrug efflux pump	30.1	2.74E-121	397
ECs_2884	mdtC_2	multidrug efflux system subunit B	VFG037720 (gb|WP_001027056)	AdeFGH efflux pump (VF0504)	Play a potential role in the synthesis and transport of autoinducer molecules during biofilm formation	setA	Acinetobacter baumannii ACICU	Biofilm	adeG	cation/multidrug efflux pump	29.8	1.88E-114	378
ECs_5098	adiY	transcriptional activator	VFG000763 (gb|BAA84859)	Per (VF0190)	Up-regulates the expression of BFP and intimin and regulates Esps secretion in response to different environmental cues; activates the expression of ler,	setA	Escherichia coli B171	Regulation	bfpT	BfpT	36.7	2.19E-19	84.7

					which then activates the expression of LEE2, LEE3, tir and LEE4 in a cascade fashion								
ECs_0598	envY	porin thermoregulatory transcriptional activator	VFG000763 (gb|BAA84859)	Per (VF0190)	Up-regulates the expression of BFP and intimin and regulates Esps secretion in response to different environmental cues; activates the expression of ler, which then activates the expression of LEE2, LEE3, tir and LEE4 in a cascade fashion	setA	Escherichia coli B171	Regulation	bfpT	BfpT	36.1	2.19E-19	84.7
ECs_0287	ECs_0287	transcription regulator	VFG000531 (gb|NP_461788)	TTSS (SPI-1 encode) (VF0116)	Delivers at least 13 effector proteins through the host cell plasma membrane, most of them are involved in actin cytoskeleton rearrangements, leading to membrane ruffling and Salmonella invasion	setA	Salmonella enterica subsp. enterica serovar Typhimurium str. LT2	Effector delivery system	hilC	AraC family transcriptional regulator	43.5	8.91E-18	80.5
ECs_2521	pabB	aminodeoxychorismate synthase subunit I	VFG026700 (gb|YP_007957699)	Mycobactin (VF0299)	Cell-association mycobactin participates in iron internalization and/or to serve as a temporary iron-holding molecule to prevent sudden influx of excess iron if the metal suddenly becomes available after a period of iron limitation	setA	Mycobacterium tuberculosis str. Haarlem/NITR202	Nutritional/Metabolic factor	mbtI	Isochorismate synthase MbtI	32	1.78E-60	205
ECs_1836	trpE	component I of	VFG026700 (gb|YP_00	Mycobactin	Cell-association mycobactin participates in iron internalization	setA	Mycobacterium	Nutritional/Metaboli	mbtI	Isochorismate	43.3	5.54E-78	253

		anthranilate synthase	7957699)	(VF0299)	and/or to serve as a temporary iron-holding molecule to prevent sudden influx of excess iron if the metal suddenly becomes available after a period of iron limitation		tuberculosis str. Haarlem/NITR202	c factor		synthase MbtI			
ECs_4789	hemN	coproporphyrinogen III oxidase	VFG013205 (gb|WP_012341592)	Heme biosynthesis (VF0758)	-	setA	Haemophilus somnus 2336	Nutritional/Metabolic factor	hemN	oxygen-independent coproporphyrinogen III oxidase	69.2	9.05E-240	662
ECs_3831	yggW	oxidoreductase	VFG013205 (gb|WP_012341592)	Heme biosynthesis (VF0758)	-	setA	Haemophilus somnus 2336	Nutritional/Metabolic factor	hemN	oxygen-independent coproporphyrinogen III oxidase	30.6	1.78E-28	115
ECs_5163	aidB	isovaleryl-CoA dehydrogenase	VFG029774 (gb|WP_071497566)	GPL locus (VF0841)	-	setB	Mycobacterium ulcerans Agy99	Immune modulation	fadE5	acyl-CoA dehydrogenase	28.8	1.63E-20	95.1
ECs_5021	espX4	T3SS effector-like protein EspX	VFG034866 (gb|WP_000900547)	TTSS secreted effectors (VF1110)	Cif (Deamidase. Induces cytopathic effects of actin stress fiber formation and cell cycle arrest.); EspB (Pore formation, actin disruption, microvilli effacement, anti-phagocytosis.); EspF (Inducing degradation of the aniapoptic protein	setA	Escherichia coli O157:H7 str. EDL933	Effector delivery system	espX4	Type III secretion system effector EspX4	100	0	976

AbcF2, tight junction disruption, microvilli effacement and elongation, mitochondrial dysfunction, N-WASP activation, SGLT-1 inactivation, pedestal maturation, inhibition of NHE3 activity, membrane remodelling; targets and disrupts the nucleolus late in infection, which is temporally controlled by host mitochondria.); EspFu/tccP (Inducing degradation of the aniapoptic protein AbcF2, tight junction disruption, microvilli effacement and elongation, mitochondrial dysfunction, N-WASP activation, SGLT-1 inactivation, pedestal maturation, inhibition of NHE3 activity, membrane remodelling; targets and disrupts the nucleolus late in infection, which is temporally controlled by host mitochondria.); EspG (TBC-like GTPase activating protein. Efficiently catalyzes GTP hydrolysis in Rab1 to disrupt of Rab1-mediated ER-to-Golgi trafficking.); EspH (First bacterial effector acting directly on RhoGEFs,

EspH directly binds to the DH-PH domain in RhoGEFs to disrupt RhoGEF-Rho signaling; critical for inhibiting macrophage phagocytosis.); EspJ (Inhibit both IgG- and complement receptor-mediated phagocytosis.); EspK; EspL1; EspL2 (Cysteine protease. Bounds F-actin-aggregating annexin 2 directly to increase annexin 2's ability to aggregate Tir-induced F-actin; block necroptosis and in flammation.); EspL4; EspM1 (GEF. Activates the RhoA signaling pathway and induce the formation of stress fibres; inhibit pedestal formation and induce tight junction mislocalization.); EspM2 (GEF. Activates the RhoA signaling pathway and induce the formation of stress fibres; inhibit pedestal formation and induce tight junction mislocalization.); EspN; EspO1-1; EspO1-2; EspR1; EspR3; EspR4; EspT (GEF. Activates Rac1 and Cdc42 leading to formation of membrane ruffles and lamellipodia; induces membrane

ruffles to facilitate bacterial invasion into non-phagocytic cells in a process involving Rac1 and Wave2.); EspW; EspX1; EspX2; EspX4; EspX5; EspX6; EspX7/nleL (E3 ubiquitin ligase, HECT-like. Modulates pedestal formation.); EspY1; EspY2; EspY3; EspY4; EspY5; Map (GEF. Mimics the host Dbl and catalyses the exchange of GDP for GTP in Cdc42, involved in effacement, SGLT1 inhibition, formation of filopodia and disruption of mitochondrial function.); NleA/espI (Disruption of tight junctions by inhibition of host cell protein trafficking through COPII-dependent pathways.); NleB1 (Blocks translocation of the p65 and to the host cell nucleus to inhibit NF-<kappa>B pathway, but NleE and NleB act at different points in the NF-<kappa>B signaling pathway.); NleB2 (May also have anti-inflammatory activity.); NleC (Metalloprotease. Zn-dependent endopeptidases that specifically clip and inactivate RelA

(p65), thus blocking NF-<kappa>B pathway.); NleD (Metalloprotease. Zn-dependent endopeptidases that specifically clip and inactivate JNK and p38, thus blocking AP-1 pathway.); NleE (PMN tran-epithelial migration; blocks translocation of the p65 to the host cell nucleus by preventing I<kappa>B degradation to inhibit NF-<kappa>B pathway.); NleF; NleG-1; NleG2-2; NleG2-3; NleG2-4; NleG5-1; NleG5-2; NleG6-1; NleG6-2; NleG6-3; NleG7 (U-box type E3 ubiquitin ligases.); NleG8-2; NleH1 (Ser/Thr protein kinase. Binds directly to a subunit of NF-<kappa>B, the ribosomal protein S3 (RPS3), reducing the nuclear abundance of RPS3 to dampen host transcriptional outputs; interact with Bax inhibitor-1 to block apoptosis.); NleH2 (Putative kinase. Attenuates NF-<kappa>B pathway.); SepZ/espZ (EspZ interacts with CD98 in host cell membranes to promote host cell survival, therefore provide the pathogen with valuable time to

					colonize efficiently prior to dissemination.); TccP2; Tir (Mimics host immunoreceptor tyrosine-based inhibition motifs (ITIMs), also see helicobacter CagA. EHEC Tir lacks the Nck binding site. Conserved NPY (Asn-Pro-Tyr) motif recruits the adaptor protein IRTKS and/or IRSp53. IRTKS/IRSp53 link Tir and TccP/EspFu, which in turn activates N-WASP; Receptor for intimin; effacement; SGLT1 inhibition; recruits SHIP2 to control actin-pedestal morphology; maintains the integrity of the epithelium by keeping the destructive activity of EspG and EspG2 in check.)							
ECs_1420	csgA	curlin major subunit CsgA	VFG042730 (gb|WP_000771428)	Curli fibers (VF1138)	In E. coli, curli fibers compose up to 85% of the biofilm biomass. Curli acts in vitro as an essential scaffold protein during biofilm formation.;In vivo, curli also directly regulates the immune system and is known to induce inflammation by activating the immune Toll-like receptors (TLRs).	setA	Escherichia coli O157:H7 str. EDL933	Adherence	csgA	curlin major subunit CsgA	100	1.15E-20	82.4
ECs_1812	nleA/espI	T3SS secreted	VFG002121 (gb|WP_00	TTSS secreted	Cif (Deamidase. Induces cytopathic effects of actin stress fiber	setA	Escherichia coli	Effector delivery	nleA/espI	Type III secretion	100	0	877

effector NleA/EspI	1025672)	effectors (VF1110)	formation and cell cycle arrest.); EspB (Pore formation, actin disruption, microvilli effacement, anti-phagocytosis.); EspF (Inducing degradation of the aniapoptic protein AbcF2, tight junction disruption, microvilli effacement and elongation, mitochondrial dysfunction, N-WASP activation, SGLT-1 inactivation, pedestal maturation, inhibition of NHE3 activity, membrane remodelling; targets and disrupts the nucleolus late in infection, which is temporally controlled by host mitochondria.); EspFu/tccP (Inducing degradation of the aniapoptic protein AbcF2, tight junction disruption, microvilli effacement and elongation, mitochondrial dysfunction, N-WASP activation, SGLT-1 inactivation, pedestal maturation, inhibition of NHE3 activity, membrane remodelling; targets and disrupts the nucleolus late in infection, which is temporally controlled by host mitochondria.); EspG (TBC-like GTPase activating	O157:H7 str. EDL933	system	system effector NleA

protein. Efficiently catalyzes GTP hydrolysis in Rab1 to disrupt of Rab1-mediated ER-to-Golgi trafficking.); EspH (First bacterial effector acting directly on RhoGEFs, EspH directly binds to the DH-PH domain in RhoGEFs to disrupt RhoGEF-Rho signaling; critical for inhibiting macrophage phagocytosis.); EspJ (Inhibit both IgG- and complement receptor-mediated phagocytosis.); EspK; EspL1; EspL2 (Cysteine protease. Bounds F-actin-aggregating annexin 2 directly to increase annexin 2's ability to aggregate Tir-induced F-actin; block necroptosis and in flammation.); EspL4; EspM1 (GEF. Activates the RhoA signaling pathway and induce the formation of stress fibres; inhibit pedestal formation and induce tight junction mislocalization.); EspM2 (GEF. Activates the RhoA signaling pathway and induce the formation of stress fibres; inhibit pedestal formation and induce tight junction

mislocalization.); EspN; EspO1-1; EspO1-2; EspR1; EspR3; EspR4; EspT (GEF. Activates Rac1 and Cdc42 leading to formation of membrane ruffles and lamellipodia; induces membrane ruffles to facilitate bacterial invasion into non-phagocytic cells in a process involving Rac1 and Wave2.); EspW; EspX1; EspX2; EspX4; EspX5; EspX6; EspX7/nleL (E3 ubiquitin ligase, HECT-like. Modulates pedestal formation.); EspY1; EspY2; EspY3; EspY4; EspY5; Map (GEF. Mimics the host Dbl and catalyses the exchange of GDP for GTP in Cdc42, involved in effacement, SGLT1 inhibition, formation of filopodia and disruption of mitochondrial function.); NleA/espI (Disruption of tight junctions by inhibition of host cell protein trafficking through COPII-dependent pathways.); NleB1 (Blocks translocation of the p65 and to the host cell nucleus to inhibit NF-<kappa>B pathway, but NleE and NleB act at different points in the

NF-<kappa>B signaling pathway.); NleB2 (May also have anti-inflammatory activity.); NleC (Metalloprotease. Zn-dependent endopeptidases that specifically clip and inactivate RelA (p65), thus blocking NF-<kappa>B pathway.); NleD (Metalloprotease. Zn-dependent endopeptidases that specifically clip and inactivate JNK and p38, thus blocking AP-1 pathway.); NleE (PMN tran-epithelial migration; blocks translocation of the p65 to the host cell nucleus by preventing I<kappa>B degradation to inhibit NF-<kappa>B pathway.); NleF; NleG-1; NleG2-2; NleG2-3; NleG2-4; NleG5-1; NleG5-2; NleG6-1; NleG6-2; NleG6-3; NleG7 (U-box type E3 ubiquitin ligases.); NleG8-2; NleH1 (Ser/Thr protein kinase. Binds directly to a subunit of NF-<kappa>B, the ribosomal protein S3 (RPS3), reducing the nuclear abundance of RPS3 to dampen host transcriptional outputs; interact with Bax inhibitor-1 to block apoptosis.); NleH2 (Putative kinase.

					Attenuates NF-<kappa>B pathway.); SepZ/espZ (EspZ interacts with CD98 in host cell membranes to promote host cell survival, therefore provide the pathogen with valuable time to colonize efficiently prior to dissemination.); TccP2; Tir (Mimics host immunoreceptor tyrosine-based inhibition motifs (ITIMs), also see helicobacter CagA. EHEC Tir lacks the Nck binding site. Conserved NPY (Asn-Pro-Tyr) motif recruits the adaptor protein IRTKS and/or IRSp53. IRTKS/IRSp53 link Tir and TccP/EspFu, which in turn activates N-WASP; Receptor for intimin; effacement; SGLT1 inhibition; recruits SHIP2 to control actin-pedestal morphology; maintains the integrity of the epithelium by keeping the destructive activity of EspG and EspG2 in check.)								
ECs_1738	galU	glucose-1-phosphate uridylyltransferas	VFG013348 (gb|WP_012054583)	LOS (VF0044)	Major immunogen; LOS phosphorylcholine (ChoP) may influence invasion via interaction with PAF receptor and stimulates of inflammatory signals; LPS phase	setA	Haemophilus influenzae PittEE	Immune modulation	galU	glucosephosphate uridylyltransferase	73.6	1.68E-149	421

		e			variation is characterized by the spontaneous loss and gain of oligosaccharide structures present in the outer core. the phase variable expression of LPS biosynthesis genes promotes evasion of antigen-specific host immune defences and allow colonization of different host microenvironments								
ECs_0061	espY1	T3SS effector-like protein EspY	VFG034895 (gb|WP_001304001)	TTSS secreted effectors (VF1110)	Cif (Deamidase. Induces cytopathic effects of actin stress fiber formation and cell cycle arrest.); EspB (Pore formation, actin disruption, microvilli effacement, anti-phagocytosis.); EspF (Inducing degradation of the aniapoptic protein AbcF2, tight junction disruption, microvilli effacement and elongation, mitochondrial dysfunction, N-WASP activation, SGLT-1 inactivation, pedestal maturation, inhibition of NHE3 activity, membrane remodelling; targets and disrupts the nucleolus late in infection, which is temporally controlled by host mitochondria.); EspFu/tccP (Inducing degradation of	setA	Escherichia coli O157:H7 str. EDL933	Effector delivery system	espY1	Type III secretion system effector EspY1	100	9.55E-171	470

the aniapoptic protein AbcF2, tight junction disruption, microvilli effacement and elongation, mitochondrial dysfunction, N-WASP activation, SGLT-1 inactivation, pedestal maturation, inhibition of NHE3 activity, membrane remodelling; targets and disrupts the nucleolus late in infection, which is temporally controlled by host mitochondria.); EspG (TBC-like GTPase activating protein. Efficiently catalyzes GTP hydrolysis in Rab1 to disrupt of Rab1-mediated ER-to-Golgi trafficking.); EspH (First bacterial effector acting directly on RhoGEFs, EspH directly binds to the DH-PH domain in RhoGEFs to disrupt RhoGEF-Rho signaling; critical for inhibiting macrophage phagocytosis.); EspJ (Inhibit both IgG- and complement receptor-mediated phagocytosis.); EspK; EspL1; EspL2 (Cysteine protease. Bounds F-actin-aggregating annexin 2 directly to increase annexin 2's ability to aggregate Tir-induced

F-actin; block necroptosis and in flammation.); EspL4; EspM1 (GEF. Activates the RhoA signaling pathway and induce the formation of stress fibres; inhibit pedestal formation and induce tight junction mislocalization.); EspM2 (GEF. Activates the RhoA signaling pathway and induce the formation of stress fibres; inhibit pedestal formation and induce tight junction mislocalization.); EspN; EspO1-1; EspO1-2; EspR1; EspR3; EspR4; EspT (GEF. Activates Rac1 and Cdc42 leading to formation of membrane ruffles and lamellipodia; induces membrane ruffles to facilitate bacterial invasion into non-phagocytic cells in a process involving Rac1 and Wave2.); EspW; EspX1; EspX2; EspX4; EspX5; EspX6; EspX7/nleL (E3 ubiquitin ligase, HECT-like. Modulates pedestal formation.); EspY1; EspY2; EspY3; EspY4; EspY5; Map (GEF. Mimics the host Dbl and catalyses the exchange of GDP for GTP in Cdc42, involved in

effacement, SGLT1 inhibition, formation of filopodia and disruption of mitochondrial function.); NleA/espI (Disruption of tight junctions by inhibition of host cell protein trafficking through COPII-dependent pathways.); NleB1 (Blocks translocation of the p65 and to the host cell nucleus to inhibit NF-<kappa>B pathway, but NleE and NleB act at different points in the NF-<kappa>B signaling pathway.); NleB2 (May also have anti-inflammatory activity.); NleC (Metalloprotease. Zn-dependent endopeptidases that specifically clip and inactivate RelA (p65), thus blocking NF-<kappa>B pathway.); NleD (Metalloprotease. Zn-dependent endopeptidases that specifically clip and inactivate JNK and p38, thus blocking AP-1 pathway.); NleE (PMN tran-epithelial migration; blocks translocation of the p65 to the host cell nucleus by preventing I<kappa>B degradation to inhibit NF-<kappa>B pathway.); NleF; NleG-1;

NleG2-2; NleG2-3; NleG2-4; NleG5-1; NleG5-2; NleG6-1; NleG6-2; NleG6-3; NleG7 (U-box type E3 ubiquitin ligases.); NleG8-2; NleH1 (Ser/Thr protein kinase. Binds directly to a subunit of NF-<kappa>B, the ribosomal protein S3 (RPS3), reducing the nuclear abundance of RPS3 to dampen host transcriptional outputs; interact with Bax inhibitor-1 to block apoptosis.); NleH2 (Putative kinase. Attenuates NF-<kappa>B pathway.); SepZ/espZ (EspZ interacts with CD98 in host cell membranes to promote host cell survival, therefore provide the pathogen with valuable time to colonize efficiently prior to dissemination.); TccP2; Tir (Mimics host immunoreceptor tyrosine-based inhibition motifs (ITIMs), also see helicobacter CagA. EHEC Tir lacks the Nck binding site. Conserved NPY (Asn-Pro-Tyr) motif recruits the adaptor protein IRTKS and/or IRSp53. IRTKS/IRSp53 link Tir and TccP/EspFu, which in turn activates N-WASP;

ECs_0073	espY2	T3SS effector-like protein EspY	VFG034895 (gb|WP_001304001)	TTSS secreted effectors (VF1110)	Receptor for intimin; effacement; SGLT1 inhibition; recruits SHIP2 to control actin-pedestal morphology; maintains the integrity of the epithelium by keeping the destructive activity of EspG and EspG2 in check.) Cif (Deamidase. Induces cytopathic effects of actin stress fiber formation and cell cycle arrest.); EspB (Pore formation, actin disruption, microvilli effacement, anti-phagocytosis.); EspF (Inducing degradation of the aniapoptic protein AbcF2, tight junction disruption, microvilli effacement and elongation, mitochondrial dysfunction, N-WASP activation, SGLT-1 inactivation, pedestal maturation, inhibition of NHE3 activity, membrane remodelling; targets and disrupts the nucleolus late in infection, which is temporally controlled by host mitochondria.); EspFu/tccP (Inducing degradation of the aniapoptic protein AbcF2, tight junction disruption, microvilli effacement and elongation,	setA	Escherichia coli O157:H7 str. EDL933	Effector delivery system	espY1	Type III secretion system effector EspY1	41.7	2.45E-26	100

mitochondrial dysfunction, N-WASP activation, SGLT-1 inactivation, pedestal maturation, inhibition of NHE3 activity, membrane remodelling; targets and disrupts the nucleolus late in infection, which is temporally controlled by host mitochondria.); EspG (TBC-like GTPase activating protein. Efficiently catalyzes GTP hydrolysis in Rab1 to disrupt of Rab1-mediated ER-to-Golgi trafficking.); EspH (First bacterial effector acting directly on RhoGEFs, EspH directly binds to the DH-PH domain in RhoGEFs to disrupt RhoGEF-Rho signaling; critical for inhibiting macrophage phagocytosis.); EspJ (Inhibit both IgG- and complement receptor-mediated phagocytosis.); EspK; EspL1; EspL2 (Cysteine protease. Bounds F-actin-aggregating annexin 2 directly to increase annexin 2's ability to aggregate Tir-induced F-actin; block necroptosis and in flammation.); EspL4; EspM1 (GEF. Activates the RhoA signaling pathway

and induce the formation of stress fibres; inhibit pedestal formation and induce tight junction mislocalization.); EspM2 (GEF. Activates the RhoA signaling pathway and induce the formation of stress fibres; inhibit pedestal formation and induce tight junction mislocalization.); EspN; EspO1-1; EspO1-2; EspR1; EspR3; EspR4; EspT (GEF. Activates Rac1 and Cdc42 leading to formation of membrane ruffles and lamellipodia; induces membrane ruffles to facilitate bacterial invasion into non-phagocytic cells in a process involving Rac1 and Wave2.); EspW; EspX1; EspX2; EspX4; EspX5; EspX6; EspX7/nleL (E3 ubiquitin ligase, HECT-like. Modulates pedestal formation.); EspY1; EspY2; EspY3; EspY4; EspY5; Map (GEF. Mimics the host Dbl and catalyses the exchange of GDP for GTP in Cdc42, involved in effacement, SGLT1 inhibition, formation of filopodia and disruption of mitochondrial function.);

NleA/espI (Disruption of tight junctions by inhibition of host cell protein trafficking through COPII-dependent pathways.); NleB1 (Blocks translocation of the p65 and to the host cell nucleus to inhibit NF-<kappa>B pathway, but NleE and NleB act at different points in the NF-<kappa>B signaling pathway.); NleB2 (May also have anti-inflammatory activity.); NleC (Metalloprotease. Zn-dependent endopeptidases that specifically clip and inactivate RelA (p65), thus blocking NF-<kappa>B pathway.); NleD (Metalloprotease. Zn-dependent endopeptidases that specifically clip and inactivate JNK and p38, thus blocking AP-1 pathway.); NleE (PMN tran-epithelial migration; blocks translocation of the p65 to the host cell nucleus by preventing I<kappa>B degradation to inhibit NF-<kappa>B pathway.); NleF; NleG-1; NleG2-2; NleG2-3; NleG2-4; NleG5-1; NleG5-2; NleG6-1; NleG6-2; NleG6-3; NleG7 (U-box type E3 ubiquitin

ligases.); NleG8-2; NleH1 (Ser/Thr protein kinase. Binds directly to a subunit of NF-<kappa>B, the ribosomal protein S3 (RPS3), reducing the nuclear abundance of RPS3 to dampen host transcriptional outputs; interact with Bax inhibitor-1 to block apoptosis.); NleH2 (Putative kinase. Attenuates NF-<kappa>B pathway.); SepZ/espZ (EspZ interacts with CD98 in host cell membranes to promote host cell survival, therefore provide the pathogen with valuable time to colonize efficiently prior to dissemination.); TccP2; Tir (Mimics host immunoreceptor tyrosine-based inhibition motifs (ITIMs), also see helicobacter CagA. EHEC Tir lacks the Nck binding site. Conserved NPY (Asn-Pro-Tyr) motif recruits the adaptor protein IRTKS and/or IRSp53. IRTKS/IRSp53 link Tir and TccP/EspFu, which in turn activates N-WASP; Receptor for intimin; effacement; SGLT1 inhibition; recruits SHIP2 to control actin-pedestal morphology;

					maintains the integrity of the epithelium by keeping the destructive activity of EspG and EspG2 in check.)								
ECs_3894	yqhC	transcriptional activator of yqhD	VFG007177 (gb|WP_005464548)	T3SS1 (VF0408)	T3SS1 uses a multifaceted mechanism to cause the cytotoxicity of host cells, involving the induction of autophagy, cell rounding, and cell lysis.	setA	Vibrio parahaemolyticus RIMD 2210633	Effector delivery system	exsA	transcriptional regulator ExsA	26.8	7.23E-09	55.8
ECs_4128	accC	acetyl-CoA carboxylase biotin carboxylase subunit	VFG047710 (gb|WP_014547360)	Pyrimidine biosynthesis (VF0558)	Mediating bacterial resistance to reactive oxygen species (ROS) that is important for phagosomal escape	setA	Francisella cf. tularensis subsp. novicida 3523	Nutritional/Metabolic factor	carB	carbamoyl phosphate synthase large subunit	26.4	4.74E-11	65.1
ECs_0036	carB	carbamoyl-phosphate synthase large subunit	VFG047710 (gb|WP_014547360)	Pyrimidine biosynthesis (VF0558)	Mediating bacterial resistance to reactive oxygen species (ROS) that is important for phagosomal escape	setA	Francisella cf. tularensis subsp. novicida 3523	Nutritional/Metabolic factor	carB	carbamoyl phosphate synthase large subunit	57.9	0	1157
ECs_4385	ECs_4385	hypothetical protein	VFG012621 (gb|WP_000189352)	Chu (VF0234)	Iron uptake: the ability to use heme and/or hemoglobin might be especially advantageous to pathogenic bacteria. These pathogens often secrete cytotoxins, which gain access to the intracellular heme reservoir besides	setA	Escherichia coli O157:H7 str. EDL933	Nutritional/Metabolic factor	chuY	ChuY	100	2.91E-146	405

					initiating tissue invasion. Cytotoxin production coupled with the capability to utilize heme and/or hemoglobin could serve as an effective iron acquisition strategy during the progression of infection								
ECs_4384	ECs_4384	heme utilization carrier protein	VFG012615 (gb|WP_000020049)	Chu (VF0234)	Iron uptake: the ability to use heme and/or hemoglobin might be especially advantageous to pathogenic bacteria. These pathogens often secrete cytotoxins, which gain access to the intracellular heme reservoir besides initiating tissue invasion. Cytotoxin production coupled with the capability to utilize heme and/or hemoglobin could serve as an effective iron acquisition strategy during the progression of infection	setA	Escherichia coli O157:H7 str. EDL933	Nutritional/Metabolic factor	chuX	putative heme-binding protein ChuX	100	6.22E-120	335
ECs_3090	ccmA	heme export ABC transporter ATPase	VFG013070 (gb|WP_001626196)	Shu (VF0256)	Shu heme utilization locus includes:; ShuA, TonB-denpendent heme receptor which is critical for the uptake of heme into the periplasm,; ShuT, putative periplasmic binding protein which specifically binds heme and acts as a soluble receptor for active uptake of heme into the cytoplasm; ShuU and	setA	Shigella dysenteriae Sd197	Nutritional/Metabolic factor	shuV	ATP-binding component of hemin transport system	30.1	2.07E-17	77.8

					ShuV comprise the cytoplasmic membrane permease and ATPase; ShuS, soluble cytoplasmic protein may function as a heme storage proteins during periods of active heme transport and as a DNA binding protein to protect the DNA from heme mediated oxidative damage								
ECs_4386	fecD	ferric citrate ABC transporter permease	VFG012628 (gb|NP_312413)	Chu (VF0234)	Iron uptake: the ability to use heme and/or hemoglobin might be especially advantageous to pathogenic bacteria. These pathogens often secrete cytotoxins, which gain access to the intracellular heme reservoir besides initiating tissue invasion. Cytotoxin production coupled with the capability to utilize heme and/or hemoglobin could serve as an effective iron acquisition strategy during the progression of infection	setA	Escherichia coli O157:H7 str. Sakai	Nutritional/Metabolic factor	chuU	heme permease protein ChuU	100	9.10E-219	597
ECs_1698	fecD	ferric citrate ABC transporter permease	VFG012627 (gb|WP_000910392)	Chu (VF0234)	Iron uptake: the ability to use heme and/or hemoglobin might be especially advantageous to pathogenic bacteria. These pathogens often secrete cytotoxins, which gain access to the intracellular heme reservoir besides initiating tissue invasion. Cytotoxin	setA	Escherichia coli O157:H7 str. EDL933	Nutritional/Metabolic factor	chuU	heme permease protein ChuU	39	1.43E-59	194

					production coupled with the capability to utilize heme and/or hemoglobin could serve as an effective iron acquisition strategy during the progression of infection								
ECs_4383	ECs_4383	coproporphyrinogen oxidase	VFG012609 (gb|WP_000993317)	Chu (VF0234)	Iron uptake: the ability to use heme and/or hemoglobin might be especially advantageous to pathogenic bacteria. These pathogens often secrete cytotoxins, which gain access to the intracellular heme reservoir besides initiating tissue invasion. Cytotoxin production coupled with the capability to utilize heme and/or hemoglobin could serve as an effective iron acquisition strategy during the progression of infection	setA	Escherichia coli O157:H7 str. EDL933	Nutritional/Metabolic factor	chuW	Putative oxygen independent coproporphyrinogen III oxidase	100	0	912
ECs_4382	ECs_4382	ABC transporter substrate-binding protein	VFG012603 (gb|WP_001302516)	Chu (VF0234)	Iron uptake: the ability to use heme and/or hemoglobin might be especially advantageous to pathogenic bacteria. These pathogens often secrete cytotoxins, which gain access to the intracellular heme reservoir besides initiating tissue invasion. Cytotoxin production coupled with the capability to utilize heme and/or hemoglobin	setA	Escherichia coli O157:H7 str. EDL933	Nutritional/Metabolic factor	chuT	periplasmic heme-binding protein ChuT	100	7.12E-209	571

					could serve as an effective iron acquisition strategy during the progression of infection								
ECs_4388	yhiD	Mg(2+) transport ATPase	VFG022673 (gb|YP_001704322)	MgtC (VF0289)	Magnesium acquisition	setA	Mycobacterium abscessus ATCC 19977	Nutritional/Metabolic factor	mgtC	Possible Mg2+ transport P-type ATPase C MgtC	46.9	2.41E-24	95.5
ECs_0605	ECs_0605	type IV secretion protein Rhs	VFG051988 (gb|WP_012314380.1)	Putida-T6SS secreted effectors (VF1354)	-	setA	Pseudomonas putida W619	Effector delivery system	tke4	T6SS Rhs-type effector Tke4	27.1	6.21E-58	221
ECs_0603	ECs_0603	type IV secretion protein Rhs	VFG051598 (gb|WP_011535065.1)	HSI-2 T6SS secreted effectors (VF0915)	PldA/tle5a (Phospholipase D. Promote invasion of host cells through the activation of the PI3K/Akt signalling pathway.); RhsP2 (ADP-ribosyltransferase. RhsP2 ADP-ribosylates 2'-hydroxyl groups of double-stranded RNA.); VgrG2b (Zinc-metallopeptidase (C-terminal effector domain). Anti-host effector that subverts the cytoskeleton and bacteriolytic activity.)	setA	Pseudomonas entomophila L48	Effector delivery system	rhsP2	ADP-ribosyltransferase toxin	34.9	8.74E-26	110
ECs_0601	nfrB	glycosyl	VFG038591	MSHA type	MSHA bundle-forming pilus is the major	setA	Aeromonas	Adherence	mshE	ATPase	30	2.42E-	60.5

		transferase family 2	(gb|WP_005348611)	IV pili (VF0477)	filamentous appendage responsible for tissue adherence and colonisation; essential for biofilm formation		veronii B565			protein MshE, GspE-like		09	
ECs_4565	sepQ	T3SS structure protein SepQ	VFG000809 (gb|WP_000803687)	TTSS (VF0191)	Injects Tir and other effector molecules directly into the host cell. Effector molecules activate cell-signaling pathways, causing alterations in the host cell cytoskeleton and resulting in the depolymerization of actin and the loss of microvilli	setA	Escherichia coli O157:H7 str. EDL933	Effector delivery system	sepQ/escQ	Type III secetion system C ring protein EscQ	100	3.00E-231	628
ECs_4564	espH	T3SS secreted effector EspH	VFG000808 (gb|WP_001302249)	TTSS secreted effectors (VF1110)	Cif (Deamidase. Induces cytopathic effects of actin stress fiber formation and cell cycle arrest.); EspB (Pore formation, actin disruption, microvilli effacement, anti-phagocytosis.); EspF (Inducing degradation of the aniapoptic protein AbcF2, tight junction disruption, microvilli effacement and elongation, mitochondrial dysfunction, N-WASP activation, SGLT-1 inactivation, pedestal maturation, inhibition of NHE3 activity, membrane remodelling; targets and disrupts the nucleolus late in infection, which is temporally	setA	Escherichia coli O157:H7 str. EDL933	Effector delivery system	espH	Type III secretion system effector EspH, RhoGEF Inhibitor	100	8.78E-122	340

controlled by host mitochondria.); EspFu/tccP (Inducing degradation of the aniapoptic protein AbcF2, tight junction disruption, microvilli effacement and elongation, mitochondrial dysfunction, N-WASP activation, SGLT-1 inactivation, pedestal maturation, inhibition of NHE3 activity, membrane remodelling; targets and disrupts the nucleolus late in infection, which is temporally controlled by host mitochondria.); EspG (TBC-like GTPase activating protein. Efficiently catalyzes GTP hydrolysis in Rab1 to disrupt of Rab1-mediated ER-to-Golgi trafficking.); EspH (First bacterial effector acting directly on RhoGEFs, EspH directly binds to the DH-PH domain in RhoGEFs to disrupt RhoGEF-Rho signaling; critical for inhibiting macrophage phagocytosis.); EspJ (Inhibit both IgG- and complement receptor-mediated phagocytosis.); EspK; EspL1; EspL2 (Cysteine protease. Bounds F-actin-aggregating annexin 2

directly to increase annexin 2's ability to aggregate Tir-induced F-actin; block necroptosis and in flammation.); EspL4; EspM1 (GEF. Activates the RhoA signaling pathway and induce the formation of stress fibres; inhibit pedestal formation and induce tight junction mislocalization.); EspM2 (GEF. Activates the RhoA signaling pathway and induce the formation of stress fibres; inhibit pedestal formation and induce tight junction mislocalization.); EspN; EspO1-1; EspO1-2; EspR1; EspR3; EspR4; EspT (GEF. Activates Rac1 and Cdc42 leading to formation of membrane ruffles and lamellipodia; induces membrane ruffles to facilitate bacterial invasion into non-phagocytic cells in a process involving Rac1 and Wave2.); EspW; EspX1; EspX2; EspX4; EspX5; EspX6; EspX7/nleL (E3 ubiquitin ligase, HECT-like. Modulates pedestal formation.); EspY1; EspY2; EspY3; EspY4; EspY5; Map (GEF. Mimics the host

Dbl and catalyses the exchange of GDP for GTP in Cdc42, involved in effacement, SGLT1 inhibition, formation of filopodia and disruption of mitochondrial function.); NleA/espI (Disruption of tight junctions by inhibition of host cell protein trafficking through COPII-dependent pathways.); NleB1 (Blocks translocation of the p65 and to the host cell nucleus to inhibit NF-<kappa>B pathway, but NleE and NleB act at different points in the NF-<kappa>B signaling pathway.); NleB2 (May also have anti-inflammatory activity.); NleC (Metalloprotease. Zn-dependent endopeptidases that specifically clip and inactivate RelA (p65), thus blocking NF-<kappa>B pathway.); NleD (Metalloprotease. Zn-dependent endopeptidases that specifically clip and inactivate JNK and p38, thus blocking AP-1 pathway.); NleE (PMN tran-epithelial migration; blocks translocation of the p65 to the host cell nucleus by preventing

I<kappa>B degradation to inhibit NF-<kappa>B pathway.); NleF; NleG-1; NleG2-2; NleG2-3; NleG2-4; NleG5-1; NleG5-2; NleG6-1; NleG6-2; NleG6-3; NleG7 (U-box type E3 ubiquitin ligases.); NleG8-2; NleH1 (Ser/Thr protein kinase. Binds directly to a subunit of NF-<kappa>B, the ribosomal protein S3 (RPS3), reducing the nuclear abundance of RPS3 to dampen host transcriptional outputs; interact with Bax inhibitor-1 to block apoptosis.); NleH2 (Putative kinase. Attenuates NF-<kappa>B pathway.); SepZ/espZ (EspZ interacts with CD98 in host cell membranes to promote host cell survival, therefore provide the pathogen with valuable time to colonize efficiently prior to dissemination.); TccP2; Tir (Mimics host immunoreceptor tyrosine-based inhibition motifs (ITIMs), also see helicobacter CagA. EHEC Tir lacks the Nck binding site. Conserved NPY (Asn-Pro-Tyr) motif recruits the adaptor protein IRTKS and/or IRSp53.

					IRTKS/IRSp53 link Tir and TccP/EspFu, which in turn activates N-WASP; Receptor for intimin; effacement; SGLT1 inhibition; recruits SHIP2 to control actin-pedestal morphology; maintains the integrity of the epithelium by keeping the destructive activity of EspG and EspG2 in check.)								
ECs_0608	cusS	copper-sensing histidine kinase in two-component regulatory system with CusR	VFG022917 (gb|WP_013830538)	PhoP (VF0286)	Senses Mg2+ starvation, controls expression of genes involved in surface remodeling and adaptation to intracellular growth	setA	Mycobacterium sp. JDM601	Regulation	phoR	Possible two component system response sensor kinase membrane associated PhoR	33.7	1.09E-34	135
ECs_0450	phoR	two-component system sensor histidine kinase PhoR	VFG009820 (gb|WP_036377494)	PhoP (VF0286)	Senses Mg2+ starvation, controls expression of genes involved in surface remodeling and adaptation to intracellular growth	setA	Mycobacterium vanbaalenii PYR-1	Regulation	phoR	Possible two component system response sensor kinase membrane	37.5	7.51E-36	137

										associated PhoR			
ECs_4563	cesF	T3SS chaperone CesF	VFG000807 (gb|WP_001003065)	TTSS (VF0191)	Injects Tir and other effector molecules directly into the host cell. Effector molecules activate cell-signaling pathways, causing alterations in the host cell cytoskeleton and resulting in the depolymerization of actin and the loss of microvilli	setA	Escherichia coli O157:H7 str. EDL933	Effector delivery system	cesF	CesF, chaperone for EspF	100	1.75E-90	257
ECs_4562	map	T3SS secreted effector Map	VFG000806 (gb|WP_000492638)	TTSS secreted effectors (VF1110)	Cif (Deamidase. Induces cytopathic effects of actin stress fiber formation and cell cycle arrest.); EspB (Pore formation, actin disruption, microvilli effacement, anti-phagocytosis.); EspF (Inducing degradation of the aniapoptic protein AbcF2, tight junction disruption, microvilli effacement and elongation, mitochondrial dysfunction, N-WASP activation, SGLT-1 inactivation, pedestal maturation, inhibition of NHE3 activity, membrane remodelling; targets and disrupts the nucleolus late in infection, which is temporally controlled by host mitochondria.);	setA	Escherichia coli O157:H7 str. EDL933	Effector delivery system	map	Type III secretion system effector Map, Rho guanine exchange factor	100	5.67E-142	394

EspFu/tccP (Inducing degradation of the aniapoptic protein AbcF2, tight junction disruption, microvilli effacement and elongation, mitochondrial dysfunction, N-WASP activation, SGLT-1 inactivation, pedestal maturation, inhibition of NHE3 activity, membrane remodelling; targets and disrupts the nucleolus late in infection, which is temporally controlled by host mitochondria.); EspG (TBC-like GTPase activating protein. Efficiently catalyzes GTP hydrolysis in Rab1 to disrupt of Rab1-mediated ER-to-Golgi trafficking.); EspH (First bacterial effector acting directly on RhoGEFs, EspH directly binds to the DH-PH domain in RhoGEFs to disrupt RhoGEF-Rho signaling; critical for inhibiting macrophage phagocytosis.); EspJ (Inhibit both IgG- and complement receptor-mediated phagocytosis.); EspK; EspL1; EspL2 (Cysteine protease. Bounds F-actin-aggregating annexin 2 directly to increase annexin 2's

ability to aggregate Tir-induced F-actin; block necroptosis and in flammation.); EspL4; EspM1 (GEF. Activates the RhoA signaling pathway and induce the formation of stress fibres; inhibit pedestal formation and induce tight junction mislocalization.); EspM2 (GEF. Activates the RhoA signaling pathway and induce the formation of stress fibres; inhibit pedestal formation and induce tight junction mislocalization.); EspN; EspO1-1; EspO1-2; EspR1; EspR3; EspR4; EspT (GEF. Activates Rac1 and Cdc42 leading to formation of membrane ruffles and lamellipodia; induces membrane ruffles to facilitate bacterial invasion into non-phagocytic cells in a process involving Rac1 and Wave2.); EspW; EspX1; EspX2; EspX4; EspX5; EspX6; EspX7/nleL (E3 ubiquitin ligase, HECT-like. Modulates pedestal formation.); EspY1; EspY2; EspY3; EspY4; EspY5; Map (GEF. Mimics the host Dbl and catalyses the exchange of GDP

for GTP in Cdc42, involved in effacement, SGLT1 inhibition, formation of filopodia and disruption of mitochondrial function.); NleA/espI (Disruption of tight junctions by inhibition of host cell protein trafficking through COPII-dependent pathways.); NleB1 (Blocks translocation of the p65 and to the host cell nucleus to inhibit NF-<kappa>B pathway, but NleE and NleB act at different points in the NF-<kappa>B signaling pathway.); NleB2 (May also have anti-inflammatory activity.); NleC (Metalloprotease. Zn-dependent endopeptidases that specifically clip and inactivate RelA (p65), thus blocking NF-<kappa>B pathway.); NleD (Metalloprotease. Zn-dependent endopeptidases that specifically clip and inactivate JNK and p38, thus blocking AP-1 pathway.); NleE (PMN tran-epithelial migration; blocks translocation of the p65 to the host cell nucleus by preventing I<kappa>B degradation to inhibit

NF-<kappa>B pathway.); NleF; NleG-1; NleG2-2; NleG2-3; NleG2-4; NleG5-1; NleG5-2; NleG6-1; NleG6-2; NleG6-3; NleG7 (U-box type E3 ubiquitin ligases.); NleG8-2; NleH1 (Ser/Thr protein kinase. Binds directly to a subunit of NF-<kappa>B, the ribosomal protein S3 (RPS3), reducing the nuclear abundance of RPS3 to dampen host transcriptional outputs; interact with Bax inhibitor-1 to block apoptosis.); NleH2 (Putative kinase. Attenuates NF-<kappa>B pathway.); SepZ/espZ (EspZ interacts with CD98 in host cell membranes to promote host cell survival, therefore provide the pathogen with valuable time to colonize efficiently prior to dissemination.); TccP2; Tir (Mimics host immunoreceptor tyrosine-based inhibition motifs (ITIMs), also see helicobacter CagA. EHEC Tir lacks the Nck binding site. Conserved NPY (Asn-Pro-Tyr) motif recruits the adaptor protein IRTKS and/or IRSp53. IRTKS/IRSp53 link Tir and TccP/EspFu,

					which in turn activates N-WASP; Receptor for intimin; effacement; SGLT1 inhibition; recruits SHIP2 to control actin-pedestal morphology; maintains the integrity of the epithelium by keeping the destructive activity of EspG and EspG2 in check.)								
ECs_3771	yqfA	hemolysin III family HylIII inner membrane protein	VFG038907 (gb|WP_005354346)	Hemolysin III (VF0647)	-	setB	Aeromonas veronii B565	Exotoxin	B565_RS04085	hemolysin III	67	1.90E-93	272
ECs_0110	hofC	assembly protein in type IV pilin biogenesis transmembrane protein	VFG042798 (gb|WP_000157271)	Hemorrhagic E.coli pilus (HCP) (VF1141)	-	setA	Escherichia coli O157:H7 str. EDL933	Adherence	hcpC	protein transport protein HofC	100	2.15E-288	780
ECs_0111	hofB	type II secretion system	VFG042799 (gb|WP_001025202)	Hemorrhagic E.coli pilus	-	setA	Escherichia coli O157:H7	Adherence	hcpB	type II secretion system	100	0	913

		protein HofB		(HCP) (VF1141)			str. EDL933			protein GspE			
ECs_0112	ppdD	prepilin peptidase-dependent pilin	VFG042800 (gb|WP_000360900)	Hemorrhagic E.coli pilus (HCP) (VF1141)	-	setA	Escherichia coli O157:H7 str. EDL933	Adherence	hcpA	prepilin peptidase-dependent pilin	100	5.53E-103	290
ECs_3261	ypdB	response regulator	VFG012195 (gb|WP_003449818)	VirR/VirS two component system (VF0729)	-	setA	Clostridium perfringens str. 13	Regulation	virR	LytTR family DNA-binding domain-containing protein	28.1	6.82E-22	90.5
ECs_2024	ydcA	hypothetical protein	VFG035328 (gb|WP_001403987)	SCI-I T6SS (VF1120)	-	setB	Escherichia coli 55989	Effector delivery system	EC55989_RS17055	hypothetical protein	65.5	1.05E-20	78.2
ECs_1421	csgC	curli production protein	VFG045794 (gb|WP_000992818)	Curli fibers (VF1138)	In E. coli, curli fibers compose up to 85% of the biofilm biomass. Curli acts in vitro as an essential scaffold protein during biofilm formation.;In vivo, curli also directly regulates the immune system and is known to induce inflammation by activating the immune Toll-like receptors (TLRs).	setA	Escherichia coli O25b:H4-ST131	Adherence	csgC	curli assembly protein CsgC	100	8.26E-72	209
ECs_0847	nleC	T3SS	VFG002122	TTSS	Cif (Deamidase. Induces cytopathic	setA	Escherichi	Effector	nleC	Type III	100	9.97E-	687

secreted effector NleC	(gb|WP_115801843)	secreted effectors (VF1110)	effects of actin stress fiber formation and cell cycle arrest.); EspB (Pore formation, actin disruption, microvilli effacement, anti-phagocytosis.); EspF (Inducing degradation of the aniapoptic protein AbcF2, tight junction disruption, microvilli effacement and elongation, mitochondrial dysfunction, N-WASP activation, SGLT-1 inactivation, pedestal maturation, inhibition of NHE3 activity, membrane remodelling; targets and disrupts the nucleolus late in infection, which is temporally controlled by host mitochondria.); EspFu/tccP (Inducing degradation of the aniapoptic protein AbcF2, tight junction disruption, microvilli effacement and elongation, mitochondrial dysfunction, N-WASP activation, SGLT-1 inactivation, pedestal maturation, inhibition of NHE3 activity, membrane remodelling; targets and disrupts the nucleolus late in infection, which is temporally controlled by host mitochondria.);	a coli O157:H7 str. EDL933	delivery system	secretion system effector NleC, zinc metallopro tease	254

EspG (TBC-like GTPase activating protein. Efficiently catalyzes GTP hydrolysis in Rab1 to disrupt of Rab1-mediated ER-to-Golgi trafficking.); EspH (First bacterial effector acting directly on RhoGEFs, EspH directly binds to the DH-PH domain in RhoGEFs to disrupt RhoGEF-Rho signaling; critical for inhibiting macrophage phagocytosis.); EspJ (Inhibit both IgG- and complement receptor-mediated phagocytosis.); EspK; EspL1; EspL2 (Cysteine protease. Bounds F-actin-aggregating annexin 2 directly to increase annexin 2's ability to aggregate Tir-induced F-actin; block necroptosis and in flammation.); EspL4; EspM1 (GEF. Activates the RhoA signaling pathway and induce the formation of stress fibres; inhibit pedestal formation and induce tight junction mislocalization.); EspM2 (GEF. Activates the RhoA signaling pathway and induce the formation of stress fibres; inhibit pedestal formation and

induce tight junction mislocalization.); EspN; EspO1-1; EspO1-2; EspR1; EspR3; EspR4; EspT (GEF. Activates Rac1 and Cdc42 leading to formation of membrane ruffles and lamellipodia; induces membrane ruffles to facilitate bacterial invasion into non-phagocytic cells in a process involving Rac1 and Wave2.); EspW; EspX1; EspX2; EspX4; EspX5; EspX6; EspX7/nleL (E3 ubiquitin ligase, HECT-like. Modulates pedestal formation.); EspY1; EspY2; EspY3; EspY4; EspY5; Map (GEF. Mimics the host Dbl and catalyses the exchange of GDP for GTP in Cdc42, involved in effacement, SGLT1 inhibition, formation of filopodia and disruption of mitochondrial function.); NleA/espI (Disruption of tight junctions by inhibition of host cell protein trafficking through COPII-dependent pathways.); NleB1 (Blocks translocation of the p65 and to the host cell nucleus to inhibit NF-<kappa>B pathway, but NleE and NleB

act at different points in the NF-<kappa>B signaling pathway.); NleB2 (May also have anti-inflammatory activity.); NleC (Metalloprotease. Zn-dependent endopeptidases that specifically clip and inactivate RelA (p65), thus blocking NF-<kappa>B pathway.); NleD (Metalloprotease. Zn-dependent endopeptidases that specifically clip and inactivate JNK and p38, thus blocking AP-1 pathway.); NleE (PMN tran-epithelial migration; blocks translocation of the p65 to the host cell nucleus by preventing I<kappa>B degradation to inhibit NF-<kappa>B pathway.); NleF; NleG-1; NleG2-2; NleG2-3; NleG2-4; NleG5-1; NleG5-2; NleG6-1; NleG6-2; NleG6-3; NleG7 (U-box type E3 ubiquitin ligases.); NleG8-2; NleH1 (Ser/Thr protein kinase. Binds directly to a subunit of NF-<kappa>B, the ribosomal protein S3 (RPS3), reducing the nuclear abundance of RPS3 to dampen host transcriptional outputs; interact with Bax inhibitor-1 to block

					apoptosis.); NleH2 (Putative kinase. Attenuates NF-<kappa>B pathway.); SepZ/espZ (EspZ interacts with CD98 in host cell membranes to promote host cell survival, therefore provide the pathogen with valuable time to colonize efficiently prior to dissemination.); TccP2; Tir (Mimics host immunoreceptor tyrosine-based inhibition motifs (ITIMs), also see helicobacter CagA. EHEC Tir lacks the Nck binding site. Conserved NPY (Asn-Pro-Tyr) motif recruits the adaptor protein IRTKS and/or IRSp53. IRTKS/IRSp53 link Tir and TccP/EspFu, which in turn activates N-WASP; Receptor for intimin; effacement; SGLT1 inhibition; recruits SHIP2 to control actin-pedestal morphology; maintains the integrity of the epithelium by keeping the destructive activity of EspG and EspG2 in check.)								
ECs_1835	trpD	anthranilate synthase component	VFG009360 (gb|WP_094987183)	Tryptophan synthesis (VF0813)	-	setA	Mycobacterium ulcerans Agy99	Nutritional/Metabolic factor	trpD	anthranilate phosphoribosyltransf	33	3.27E-44	160

		II								erase			
ECs_3487	espW	T3SS secreted effector EspW	VFG034840 (gb|WP_001301665)	TTSS secreted effectors (VF1110)	Cif (Deamidase. Induces cytopathic effects of actin stress fiber formation and cell cycle arrest.); EspB (Pore formation, actin disruption, microvilli effacement, anti-phagocytosis.); EspF (Inducing degradation of the aniapoptic protein AbcF2, tight junction disruption, microvilli effacement and elongation, mitochondrial dysfunction, N-WASP activation, SGLT-1 inactivation, pedestal maturation, inhibition of NHE3 activity, membrane remodelling; targets and disrupts the nucleolus late in infection, which is temporally controlled by host mitochondria.); EspFu/tccP (Inducing degradation of the aniapoptic protein AbcF2, tight junction disruption, microvilli effacement and elongation, mitochondrial dysfunction, N-WASP activation, SGLT-1 inactivation, pedestal maturation, inhibition of NHE3 activity, membrane remodelling; targets and disrupts the nucleolus	setA	Escherichia coli O157:H7 str. EDL933	Effector delivery system	espW	Type III secretion system effector EspW	100	5.18E-258	699

late in infection, which is temporally controlled by host mitochondria.); EspG (TBC-like GTPase activating protein. Efficiently catalyzes GTP hydrolysis in Rab1 to disrupt of Rab1-mediated ER-to-Golgi trafficking.); EspH (First bacterial effector acting directly on RhoGEFs, EspH directly binds to the DH-PH domain in RhoGEFs to disrupt RhoGEF-Rho signaling; critical for inhibiting macrophage phagocytosis.); EspJ (Inhibit both IgG- and complement receptor-mediated phagocytosis.); EspK; EspL1; EspL2 (Cysteine protease. Bounds F-actin-aggregating annexin 2 directly to increase annexin 2's ability to aggregate Tir-induced F-actin; block necroptosis and in flammation.); EspL4; EspM1 (GEF. Activates the RhoA signaling pathway and induce the formation of stress fibres; inhibit pedestal formation and induce tight junction mislocalization.); EspM2 (GEF. Activates the RhoA signaling pathway

and induce the formation of stress fibres; inhibit pedestal formation and induce tight junction mislocalization.); EspN; EspO1-1; EspO1-2; EspR1; EspR3; EspR4; EspT (GEF. Activates Rac1 and Cdc42 leading to formation of membrane ruffles and lamellipodia; induces membrane ruffles to facilitate bacterial invasion into non-phagocytic cells in a process involving Rac1 and Wave2.); EspW; EspX1; EspX2; EspX4; EspX5; EspX6; EspX7/nleL (E3 ubiquitin ligase, HECT-like. Modulates pedestal formation.); EspY1; EspY2; EspY3; EspY4; EspY5; Map (GEF. Mimics the host Dbl and catalyses the exchange of GDP for GTP in Cdc42, involved in effacement, SGLT1 inhibition, formation of filopodia and disruption of mitochondrial function.); NleA/espI (Disruption of tight junctions by inhibition of host cell protein trafficking through COPII-dependent pathways.); NleB1 (Blocks translocation of the p65 and to

the host cell nucleus to inhibit NF-<kappa>B pathway, but NleE and NleB act at different points in the NF-<kappa>B signaling pathway.); NleB2 (May also have anti-inflammatory activity.); NleC (Metalloprotease. Zn-dependent endopeptidases that specifically clip and inactivate RelA (p65), thus blocking NF-<kappa>B pathway.); NleD (Metalloprotease. Zn-dependent endopeptidases that specifically clip and inactivate JNK and p38, thus blocking AP-1 pathway.); NleE (PMN tran-epithelial migration; blocks translocation of the p65 to the host cell nucleus by preventing I<kappa>B degradation to inhibit NF-<kappa>B pathway.); NleF; NleG-1; NleG2-2; NleG2-3; NleG2-4; NleG5-1; NleG5-2; NleG6-1; NleG6-2; NleG6-3; NleG7 (U-box type E3 ubiquitin ligases.); NleG8-2; NleH1 (Ser/Thr protein kinase. Binds directly to a subunit of NF-<kappa>B, the ribosomal protein S3 (RPS3), reducing the nuclear abundance of RPS3 to dampen

					host transcriptional outputs; interact with Bax inhibitor-1 to block apoptosis.); NleH2 (Putative kinase. Attenuates NF-<kappa>B pathway.); SepZ/espZ (EspZ interacts with CD98 in host cell membranes to promote host cell survival, therefore provide the pathogen with valuable time to colonize efficiently prior to dissemination.); TccP2; Tir (Mimics host immunoreceptor tyrosine-based inhibition motifs (ITIMs), also see helicobacter CagA. EHEC Tir lacks the Nck binding site. Conserved NPY (Asn-Pro-Tyr) motif recruits the adaptor protein IRTKS and/or IRSp53. IRTKS/IRSp53 link Tir and TccP/EspFu, which in turn activates N-WASP; Receptor for intimin; effacement; SGLT1 inhibition; recruits SHIP2 to control actin-pedestal morphology; maintains the integrity of the epithelium by keeping the destructive activity of EspG and EspG2 in check.)								
ECs_3486	nleG8-2	T3SS secreted	VFG035053 (gb|WP_00	TTSS secreted	Cif (Deamidase. Induces cytopathic effects of actin stress fiber	setA	Escherichi a coli	Effector delivery	nleG8-2	DUF1076 domain-con	100	1.90E-160	441

effector NleG	1144077)	effectors (VF1110)	formation and cell cycle arrest.); EspB (Pore formation, actin disruption, microvilli effacement, anti-phagocytosis.); EspF (Inducing degradation of the aniapoptic protein AbcF2, tight junction disruption, microvilli effacement and elongation, mitochondrial dysfunction, N-WASP activation, SGLT-1 inactivation, pedestal maturation, inhibition of NHE3 activity, membrane remodelling; targets and disrupts the nucleolus late in infection, which is temporally controlled by host mitochondria.); EspFu/tccP (Inducing degradation of the aniapoptic protein AbcF2, tight junction disruption, microvilli effacement and elongation, mitochondrial dysfunction, N-WASP activation, SGLT-1 inactivation, pedestal maturation, inhibition of NHE3 activity, membrane remodelling; targets and disrupts the nucleolus late in infection, which is temporally controlled by host mitochondria.); EspG (TBC-like GTPase activating	O157:H7 str. EDL933	system	taining protein

protein. Efficiently catalyzes GTP hydrolysis in Rab1 to disrupt of Rab1-mediated ER-to-Golgi trafficking.); EspH (First bacterial effector acting directly on RhoGEFs, EspH directly binds to the DH-PH domain in RhoGEFs to disrupt RhoGEF-Rho signaling; critical for inhibiting macrophage phagocytosis.); EspJ (Inhibit both IgG- and complement receptor-mediated phagocytosis.); EspK; EspL1; EspL2 (Cysteine protease. Bounds F-actin-aggregating annexin 2 directly to increase annexin 2's ability to aggregate Tir-induced F-actin; block necroptosis and in flammation.); EspL4; EspM1 (GEF. Activates the RhoA signaling pathway and induce the formation of stress fibres; inhibit pedestal formation and induce tight junction mislocalization.); EspM2 (GEF. Activates the RhoA signaling pathway and induce the formation of stress fibres; inhibit pedestal formation and induce tight junction

mislocalization.); EspN; EspO1-1; EspO1-2; EspR1; EspR3; EspR4; EspT (GEF. Activates Rac1 and Cdc42 leading to formation of membrane ruffles and lamellipodia; induces membrane ruffles to facilitate bacterial invasion into non-phagocytic cells in a process involving Rac1 and Wave2.); EspW; EspX1; EspX2; EspX4; EspX5; EspX6; EspX7/nleL (E3 ubiquitin ligase, HECT-like. Modulates pedestal formation.); EspY1; EspY2; EspY3; EspY4; EspY5; Map (GEF. Mimics the host Dbl and catalyses the exchange of GDP for GTP in Cdc42, involved in effacement, SGLT1 inhibition, formation of filopodia and disruption of mitochondrial function.); NleA/espI (Disruption of tight junctions by inhibition of host cell protein trafficking through COPII-dependent pathways.); NleB1 (Blocks translocation of the p65 and to the host cell nucleus to inhibit NF-<kappa>B pathway, but NleE and NleB act at different points in the

NF-<kappa>B signaling pathway.); NleB2 (May also have anti-inflammatory activity.); NleC (Metalloprotease. Zn-dependent endopeptidases that specifically clip and inactivate RelA (p65), thus blocking NF-<kappa>B pathway.); NleD (Metalloprotease. Zn-dependent endopeptidases that specifically clip and inactivate JNK and p38, thus blocking AP-1 pathway.); NleE (PMN tran-epithelial migration; blocks translocation of the p65 to the host cell nucleus by preventing I<kappa>B degradation to inhibit NF-<kappa>B pathway.); NleF; NleG-1; NleG2-2; NleG2-3; NleG2-4; NleG5-1; NleG5-2; NleG6-1; NleG6-2; NleG6-3; NleG7 (U-box type E3 ubiquitin ligases.); NleG8-2; NleH1 (Ser/Thr protein kinase. Binds directly to a subunit of NF-<kappa>B, the ribosomal protein S3 (RPS3), reducing the nuclear abundance of RPS3 to dampen host transcriptional outputs; interact with Bax inhibitor-1 to block apoptosis.); NleH2 (Putative kinase.

ECs_1824	nleG	T3SS secreted effector NleG	VFG035053 (gb|WP_001144077)	TTSS secreted effectors (VF1110)	Attenuates NF-<kappa>B pathway.); SepZ/espZ (EspZ interacts with CD98 in host cell membranes to promote host cell survival, therefore provide the pathogen with valuable time to colonize efficiently prior to dissemination.); TccP2; Tir (Mimics host immunoreceptor tyrosine-based inhibition motifs (ITIMs), also see helicobacter CagA. EHEC Tir lacks the Nck binding site. Conserved NPY (Asn-Pro-Tyr) motif recruits the adaptor protein IRTKS and/or IRSp53. IRTKS/IRSp53 link Tir and TccP/EspFu, which in turn activates N-WASP; Receptor for intimin; effacement; SGLT1 inhibition; recruits SHIP2 to control actin-pedestal morphology; maintains the integrity of the epithelium by keeping the destructive activity of EspG and EspG2 in check.) Cif (Deamidase. Induces cytopathic effects of actin stress fiber formation and cell cycle arrest.); EspB (Pore formation, actin disruption, microvilli effacement,	setA	Escherichia coli O157:H7 str. EDL933	Effector delivery system	nleG8-2	DUF1076 domain-containing protein	63.6	4.28E-90	263

anti-phagocytosis.); EspF (Inducing degradation of the aniapoptic protein AbcF2, tight junction disruption, microvilli effacement and elongation, mitochondrial dysfunction, N-WASP activation, SGLT-1 inactivation, pedestal maturation, inhibition of NHE3 activity, membrane remodelling; targets and disrupts the nucleolus late in infection, which is temporally controlled by host mitochondria.); EspFu/tccP (Inducing degradation of the aniapoptic protein AbcF2, tight junction disruption, microvilli effacement and elongation, mitochondrial dysfunction, N-WASP activation, SGLT-1 inactivation, pedestal maturation, inhibition of NHE3 activity, membrane remodelling; targets and disrupts the nucleolus late in infection, which is temporally controlled by host mitochondria.); EspG (TBC-like GTPase activating protein. Efficiently catalyzes GTP hydrolysis in Rab1 to disrupt of Rab1-mediated ER-to-Golgi

trafficking.); EspH (First bacterial effector acting directly on RhoGEFs, EspH directly binds to the DH-PH domain in RhoGEFs to disrupt RhoGEF-Rho signaling; critical for inhibiting macrophage phagocytosis.); EspJ (Inhibit both IgG- and complement receptor-mediated phagocytosis.); EspK; EspL1; EspL2 (Cysteine protease. Bounds F-actin-aggregating annexin 2 directly to increase annexin 2's ability to aggregate Tir-induced F-actin; block necroptosis and in flammation.); EspL4; EspM1 (GEF. Activates the RhoA signaling pathway and induce the formation of stress fibres; inhibit pedestal formation and induce tight junction mislocalization.); EspM2 (GEF. Activates the RhoA signaling pathway and induce the formation of stress fibres; inhibit pedestal formation and induce tight junction mislocalization.); EspN; EspO1-1; EspO1-2; EspR1; EspR3; EspR4; EspT (GEF. Activates Rac1 and Cdc42 leading

to formation of membrane ruffles and lamellipodia; induces membrane ruffles to facilitate bacterial invasion into non-phagocytic cells in a process involving Rac1 and Wave2.); EspW; EspX1; EspX2; EspX4; EspX5; EspX6; EspX7/nleL (E3 ubiquitin ligase, HECT-like. Modulates pedestal formation.); EspY1; EspY2; EspY3; EspY4; EspY5; Map (GEF. Mimics the host Dbl and catalyses the exchange of GDP for GTP in Cdc42, involved in effacement, SGLT1 inhibition, formation of filopodia and disruption of mitochondrial function.); NleA/espI (Disruption of tight junctions by inhibition of host cell protein trafficking through COPII-dependent pathways.); NleB1 (Blocks translocation of the p65 and to the host cell nucleus to inhibit NF-<kappa>B pathway, but NleE and NleB act at different points in the NF-<kappa>B signaling pathway.); NleB2 (May also have anti-inflammatory activity.); NleC (Metalloprotease.

Zn-dependent endopeptidases that specifically clip and inactivate RelA (p65), thus blocking NF-<kappa>B pathway.); NleD (Metalloprotease. Zn-dependent endopeptidases that specifically clip and inactivate JNK and p38, thus blocking AP-1 pathway.); NleE (PMN tran-epithelial migration; blocks translocation of the p65 to the host cell nucleus by preventing I<kappa>B degradation to inhibit NF-<kappa>B pathway.); NleF; NleG-1; NleG2-2; NleG2-3; NleG2-4; NleG5-1; NleG5-2; NleG6-1; NleG6-2; NleG6-3; NleG7 (U-box type E3 ubiquitin ligases.); NleG8-2; NleH1 (Ser/Thr protein kinase. Binds directly to a subunit of NF-<kappa>B, the ribosomal protein S3 (RPS3), reducing the nuclear abundance of RPS3 to dampen host transcriptional outputs; interact with Bax inhibitor-1 to block apoptosis.); NleH2 (Putative kinase. Attenuates NF-<kappa>B pathway.); SepZ/espZ (EspZ interacts with CD98 in host cell membranes to promote host

ECs_3485	espM2	T3SS secreted effector EspM	VFG034786 (gb|WP_001132157)	TTSS secreted effectors (VF1110)	cell survival, therefore provide the pathogen with valuable time to colonize efficiently prior to dissemination.); TccP2; Tir (Mimics host immunoreceptor tyrosine-based inhibition motifs (ITIMs), also see helicobacter CagA. EHEC Tir lacks the Nck binding site. Conserved NPY (Asn-Pro-Tyr) motif recruits the adaptor protein IRTKS and/or IRSp53. IRTKS/IRSp53 link Tir and TccP/EspFu, which in turn activates N-WASP; Receptor for intimin; effacement; SGLT1 inhibition; recruits SHIP2 to control actin-pedestal morphology; maintains the integrity of the epithelium by keeping the destructive activity of EspG and EspG2 in check.) Cif (Deamidase. Induces cytopathic effects of actin stress fiber formation and cell cycle arrest.); EspB (Pore formation, actin disruption, microvilli effacement, anti-phagocytosis.); EspF (Inducing degradation of the aniapoptic protein AbcF2, tight junction disruption,	setA	Escherichia coli O157:H7 str. EDL933	Effector delivery system	espM2	Type III secretion system effector EspM2, Rho guanine exchange factor	100	3.72E-139	386

microvilli effacement and elongation, mitochondrial dysfunction, N-WASP activation, SGLT-1 inactivation, pedestal maturation, inhibition of NHE3 activity, membrane remodelling; targets and disrupts the nucleolus late in infection, which is temporally controlled by host mitochondria.); EspFu/tccP (Inducing degradation of the aniapoptic protein AbcF2, tight junction disruption, microvilli effacement and elongation, mitochondrial dysfunction, N-WASP activation, SGLT-1 inactivation, pedestal maturation, inhibition of NHE3 activity, membrane remodelling; targets and disrupts the nucleolus late in infection, which is temporally controlled by host mitochondria.); EspG (TBC-like GTPase activating protein. Efficiently catalyzes GTP hydrolysis in Rab1 to disrupt of Rab1-mediated ER-to-Golgi trafficking.); EspH (First bacterial effector acting directly on RhoGEFs, EspH directly binds to the DH-PH domain

in RhoGEFs to disrupt RhoGEF-Rho signaling; critical for inhibiting macrophage phagocytosis.); EspJ (Inhibit both IgG- and complement receptor-mediated phagocytosis.); EspK; EspL1; EspL2 (Cysteine protease. Bounds F-actin-aggregating annexin 2 directly to increase annexin 2's ability to aggregate Tir-induced F-actin; block necroptosis and in flammation.); EspL4; EspM1 (GEF. Activates the RhoA signaling pathway and induce the formation of stress fibres; inhibit pedestal formation and induce tight junction mislocalization.); EspM2 (GEF. Activates the RhoA signaling pathway and induce the formation of stress fibres; inhibit pedestal formation and induce tight junction mislocalization.); EspN; EspO1-1; EspO1-2; EspR1; EspR3; EspR4; EspT (GEF. Activates Rac1 and Cdc42 leading to formation of membrane ruffles and lamellipodia; induces membrane ruffles to facilitate bacterial

invasion into non-phagocytic cells in a process involving Rac1 and Wave2.); EspW; EspX1; EspX2; EspX4; EspX5; EspX6; EspX7/nleL (E3 ubiquitin ligase, HECT-like. Modulates pedestal formation.); EspY1; EspY2; EspY3; EspY4; EspY5; Map (GEF. Mimics the host Dbl and catalyses the exchange of GDP for GTP in Cdc42, involved in effacement, SGLT1 inhibition, formation of filopodia and disruption of mitochondrial function.); NleA/espI (Disruption of tight junctions by inhibition of host cell protein trafficking through COPII-dependent pathways.); NleB1 (Blocks translocation of the p65 and to the host cell nucleus to inhibit NF-<kappa>B pathway, but NleE and NleB act at different points in the NF-<kappa>B signaling pathway.); NleB2 (May also have anti-inflammatory activity.); NleC (Metalloprotease. Zn-dependent endopeptidases that specifically clip and inactivate RelA (p65), thus blocking NF-<kappa>B

pathway.); NleD (Metalloprotease. Zn-dependent endopeptidases that specifically clip and inactivate JNK and p38, thus blocking AP-1 pathway.); NleE (PMN tran-epithelial migration; blocks translocation of the p65 to the host cell nucleus by preventing I<kappa>B degradation to inhibit NF-<kappa>B pathway.); NleF; NleG-1; NleG2-2; NleG2-3; NleG2-4; NleG5-1; NleG5-2; NleG6-1; NleG6-2; NleG6-3; NleG7 (U-box type E3 ubiquitin ligases.); NleG8-2; NleH1 (Ser/Thr protein kinase. Binds directly to a subunit of NF-<kappa>B, the ribosomal protein S3 (RPS3), reducing the nuclear abundance of RPS3 to dampen host transcriptional outputs; interact with Bax inhibitor-1 to block apoptosis.); NleH2 (Putative kinase. Attenuates NF-<kappa>B pathway.); SepZ/espZ (EspZ interacts with CD98 in host cell membranes to promote host cell survival, therefore provide the pathogen with valuable time to colonize efficiently prior to

ECs_1825	espM1	T3SS secreted effector EspM	VFG034786 (gb|WP_001132157)	TTSS secreted effectors (VF1110)	dissemination.); TccP2; Tir (Mimics host immunoreceptor tyrosine-based inhibition motifs (ITIMs), also see helicobacter CagA. EHEC Tir lacks the Nck binding site. Conserved NPY (Asn-Pro-Tyr) motif recruits the adaptor protein IRTKS and/or IRSp53. IRTKS/IRSp53 link Tir and TccP/EspFu, which in turn activates N-WASP; Receptor for intimin; effacement; SGLT1 inhibition; recruits SHIP2 to control actin-pedestal morphology; maintains the integrity of the epithelium by keeping the destructive activity of EspG and EspG2 in check.) Cif (Deamidase. Induces cytopathic effects of actin stress fiber formation and cell cycle arrest.); EspB (Pore formation, actin disruption, microvilli effacement, anti-phagocytosis.); EspF (Inducing degradation of the aniapoptic protein AbcF2, tight junction disruption, microvilli effacement and elongation, mitochondrial dysfunction, N-WASP activation, SGLT-1 inactivation,	setA	Escherichia coli O157:H7 str. EDL933	Effector delivery system	espM2	Type III secretion system effector EspM2, Rho guanine exchange factor	76	8.87E-106	301

pedestal maturation, inhibition of NHE3 activity, membrane remodelling; targets and disrupts the nucleolus late in infection, which is temporally controlled by host mitochondria.); EspFu/tccP (Inducing degradation of the aniapoptic protein AbcF2, tight junction disruption, microvilli effacement and elongation, mitochondrial dysfunction, N-WASP activation, SGLT-1 inactivation, pedestal maturation, inhibition of NHE3 activity, membrane remodelling; targets and disrupts the nucleolus late in infection, which is temporally controlled by host mitochondria.); EspG (TBC-like GTPase activating protein. Efficiently catalyzes GTP hydrolysis in Rab1 to disrupt of Rab1-mediated ER-to-Golgi trafficking.); EspH (First bacterial effector acting directly on RhoGEFs, EspH directly binds to the DH-PH domain in RhoGEFs to disrupt RhoGEF-Rho signaling; critical for inhibiting macrophage phagocytosis.); EspJ

(Inhibit both IgG- and complement receptor-mediated phagocytosis.); EspK; EspL1; EspL2 (Cysteine protease. Bounds F-actin-aggregating annexin 2 directly to increase annexin 2's ability to aggregate Tir-induced F-actin; block necroptosis and in flammation.); EspL4; EspM1 (GEF. Activates the RhoA signaling pathway and induce the formation of stress fibres; inhibit pedestal formation and induce tight junction mislocalization.); EspM2 (GEF. Activates the RhoA signaling pathway and induce the formation of stress fibres; inhibit pedestal formation and induce tight junction mislocalization.); EspN; EspO1-1; EspO1-2; EspR1; EspR3; EspR4; EspT (GEF. Activates Rac1 and Cdc42 leading to formation of membrane ruffles and lamellipodia; induces membrane ruffles to facilitate bacterial invasion into non-phagocytic cells in a process involving Rac1 and Wave2.); EspW; EspX1; EspX2; EspX4; EspX5;

EspX6; EspX7/nleL (E3 ubiquitin ligase, HECT-like. Modulates pedestal formation.); EspY1; EspY2; EspY3; EspY4; EspY5; Map (GEF. Mimics the host Dbl and catalyses the exchange of GDP for GTP in Cdc42, involved in effacement, SGLT1 inhibition, formation of filopodia and disruption of mitochondrial function.); NleA/espI (Disruption of tight junctions by inhibition of host cell protein trafficking through COPII-dependent pathways.); NleB1 (Blocks translocation of the p65 and to the host cell nucleus to inhibit NF-<kappa>B pathway, but NleE and NleB act at different points in the NF-<kappa>B signaling pathway.); NleB2 (May also have anti-inflammatory activity.); NleC (Metalloprotease. Zn-dependent endopeptidases that specifically clip and inactivate RelA (p65), thus blocking NF-<kappa>B pathway.); NleD (Metalloprotease. Zn-dependent endopeptidases that specifically clip and inactivate JNK

and p38, thus blocking AP-1 pathway.); NleE (PMN tran-epithelial migration; blocks translocation of the p65 to the host cell nucleus by preventing I<kappa>B degradation to inhibit NF-<kappa>B pathway.); NleF; NleG-1; NleG2-2; NleG2-3; NleG2-4; NleG5-1; NleG5-2; NleG6-1; NleG6-2; NleG6-3; NleG7 (U-box type E3 ubiquitin ligases.); NleG8-2; NleH1 (Ser/Thr protein kinase. Binds directly to a subunit of NF-<kappa>B, the ribosomal protein S3 (RPS3), reducing the nuclear abundance of RPS3 to dampen host transcriptional outputs; interact with Bax inhibitor-1 to block apoptosis.); NleH2 (Putative kinase. Attenuates NF-<kappa>B pathway.); SepZ/espZ (EspZ interacts with CD98 in host cell membranes to promote host cell survival, therefore provide the pathogen with valuable time to colonize efficiently prior to dissemination.); TccP2; Tir (Mimics host immunoreceptor tyrosine-based inhibition motifs (ITIMs), also see

					helicobacter CagA. EHEC Tir lacks the Nck binding site. Conserved NPY (Asn-Pro-Tyr) motif recruits the adaptor protein IRTKS and/or IRSp53. IRTKS/IRSp53 link Tir and TccP/EspFu, which in turn activates N-WASP; Receptor for intimin; effacement; SGLT1 inhibition; recruits SHIP2 to control actin-pedestal morphology; maintains the integrity of the epithelium by keeping the destructive activity of EspG and EspG2 in check.)								
ECs_3729	eivI	type III secretion apparatus protein EivI	VFG042080 (gb|NP_311756)	ETT2 (VF1161)	-	setB	Escherichia coli O157:H7 str. Sakai	Effector delivery system	eivI	type III secretion apparatus protein EivI	99.3	1.09E-96	275
ECs_4145	yrdA	transferase	VFG045340 (gb|WP_002964382)	T4SS secreted effectors (VF0695)	BPE005 (Might have an effect on cAMP-dependent signaling pathways in the host cell blocking the binding between cAMP and PKA.); BPE043; BPE123; BPE275; BspA; BspB (Promotes rBCV biogenesis and intracellular proliferation.); BspC; BspE; BspF (Contributes to bacterial growth within the replication-permissive	setA	Brucella melitensis bv. 1 str. 16M	Effector delivery system	ricA	type IV secretion system effector RicA, Rab2 interacting conserved protein A	42.5	3.55E-24	93.2

					rBCVs.); BspL (Regulates the late stages of the Brucella intracellular cycle.); RicA (Modulates rBCV biogenesis.); SepA (Endosome-like Brucella-containing vacuole (eBCV) trafficking.); VceA; VceC (Activating ER stress and further manipulating UPR to inhibit GTC apoptosis.)								
ECs_0038	caiE	carnitine operon protein CaiE	VFG045340 (gb|WP_002964382)	T4SS secreted effectors (VF0695)	BPE005 (Might have an effect on cAMP-dependent signaling pathways in the host cell blocking the binding between cAMP and PKA.); BPE043; BPE123; BPE275; BspA; BspB (Promotes rBCV biogenesis and intracellular proliferation.); BspC; BspE; BspF (Contributes to bacterial growth within the replication-permissive rBCVs.); BspL (Regulates the late stages of the Brucella intracellular cycle.); RicA (Modulates rBCV biogenesis.); SepA (Endosome-like Brucella-containing vacuole (eBCV) trafficking.); VceA; VceC (Activating ER stress and further manipulating UPR to inhibit GTC apoptosis.)	setA	Brucella melitensis bv. 1 str. 16M	Effector delivery system	ricA	type IV secretion system effector RicA, Rab2 interacting conserved protein A	34.1	5.47E-18	77.4
ECs_5048	EspX5	T3SS	VFG034873	TTSS	Cif (Deamidase. Induces cytopathic	setA	Escherichi	Effector	espX5	Type III	100	3.99E-	618

effector-like protein EspX	(gb|WP_001270084)	secreted effectors (VF1110)	effects of actin stress fiber formation and cell cycle arrest.); EspB (Pore formation, actin disruption, microvilli effacement, anti-phagocytosis.); EspF (Inducing degradation of the aniapoptic protein AbcF2, tight junction disruption, microvilli effacement and elongation, mitochondrial dysfunction, N-WASP activation, SGLT-1 inactivation, pedestal maturation, inhibition of NHE3 activity, membrane remodelling; targets and disrupts the nucleolus late in infection, which is temporally controlled by host mitochondria.); EspFu/tccP (Inducing degradation of the aniapoptic protein AbcF2, tight junction disruption, microvilli effacement and elongation, mitochondrial dysfunction, N-WASP activation, SGLT-1 inactivation, pedestal maturation, inhibition of NHE3 activity, membrane remodelling; targets and disrupts the nucleolus late in infection, which is temporally controlled by host mitochondria.);	a coli O157:H7 str. EDL933	delivery system	secretion system effector EspX5	223

EspG (TBC-like GTPase activating protein. Efficiently catalyzes GTP hydrolysis in Rab1 to disrupt of Rab1-mediated ER-to-Golgi trafficking.); EspH (First bacterial effector acting directly on RhoGEFs, EspH directly binds to the DH-PH domain in RhoGEFs to disrupt RhoGEF-Rho signaling; critical for inhibiting macrophage phagocytosis.); EspJ (Inhibit both IgG- and complement receptor-mediated phagocytosis.); EspK; EspL1; EspL2 (Cysteine protease. Bounds F-actin-aggregating annexin 2 directly to increase annexin 2's ability to aggregate Tir-induced F-actin; block necroptosis and in flammation.); EspL4; EspM1 (GEF. Activates the RhoA signaling pathway and induce the formation of stress fibres; inhibit pedestal formation and induce tight junction mislocalization.); EspM2 (GEF. Activates the RhoA signaling pathway and induce the formation of stress fibres; inhibit pedestal formation and

induce tight junction mislocalization.); EspN; EspO1-1; EspO1-2; EspR1; EspR3; EspR4; EspT (GEF. Activates Rac1 and Cdc42 leading to formation of membrane ruffles and lamellipodia; induces membrane ruffles to facilitate bacterial invasion into non-phagocytic cells in a process involving Rac1 and Wave2.); EspW; EspX1; EspX2; EspX4; EspX5; EspX6; EspX7/nleL (E3 ubiquitin ligase, HECT-like. Modulates pedestal formation.); EspY1; EspY2; EspY3; EspY4; EspY5; Map (GEF. Mimics the host Dbl and catalyses the exchange of GDP for GTP in Cdc42, involved in effacement, SGLT1 inhibition, formation of filopodia and disruption of mitochondrial function.); NleA/espI (Disruption of tight junctions by inhibition of host cell protein trafficking through COPII-dependent pathways.); NleB1 (Blocks translocation of the p65 and to the host cell nucleus to inhibit NF-<kappa>B pathway, but NleE and NleB

act at different points in the NF-<kappa>B signaling pathway.); NleB2 (May also have anti-inflammatory activity.); NleC (Metalloprotease. Zn-dependent endopeptidases that specifically clip and inactivate RelA (p65), thus blocking NF-<kappa>B pathway.); NleD (Metalloprotease. Zn-dependent endopeptidases that specifically clip and inactivate JNK and p38, thus blocking AP-1 pathway.); NleE (PMN tran-epithelial migration; blocks translocation of the p65 to the host cell nucleus by preventing I<kappa>B degradation to inhibit NF-<kappa>B pathway.); NleF; NleG-1; NleG2-2; NleG2-3; NleG2-4; NleG5-1; NleG5-2; NleG6-1; NleG6-2; NleG6-3; NleG7 (U-box type E3 ubiquitin ligases.); NleG8-2; NleH1 (Ser/Thr protein kinase. Binds directly to a subunit of NF-<kappa>B, the ribosomal protein S3 (RPS3), reducing the nuclear abundance of RPS3 to dampen host transcriptional outputs; interact with Bax inhibitor-1 to block

					apoptosis.); NleH2 (Putative kinase. Attenuates NF-<kappa>B pathway.); SepZ/espZ (EspZ interacts with CD98 in host cell membranes to promote host cell survival, therefore provide the pathogen with valuable time to colonize efficiently prior to dissemination.); TccP2; Tir (Mimics host immunoreceptor tyrosine-based inhibition motifs (ITIMs), also see helicobacter CagA. EHEC Tir lacks the Nck binding site. Conserved NPY (Asn-Pro-Tyr) motif recruits the adaptor protein IRTKS and/or IRSp53. IRTKS/IRSp53 link Tir and TccP/EspFu, which in turn activates N-WASP; Receptor for intimin; effacement; SGLT1 inhibition; recruits SHIP2 to control actin-pedestal morphology; maintains the integrity of the epithelium by keeping the destructive activity of EspG and EspG2 in check.)								
ECs_3731	eivA	type III secretion protein EivA	VFG042078 (gb|NP_311758)	ETT2 (VF1161)	-	setA	Escherichia coli O157:H7 str. Sakai	Effector delivery system	eivA	type III secretion protein EivA	100	0	1262

ECs_3730	eivC	type III secretion apparatus protein EivC	VFG042081 (gb|NP_311757)	ETT2 (VF1161)	-	setA	Escherichia coli O157:H7 str. Sakai	Effector delivery system	eivC	type III secretion apparatus protein EivC	100	2.17e-310	838
ECs_3732	eivE	type III secretion protein EivE	VFG042077 (gb|NP_311759)	ETT2 (VF1161)	-	setA	Escherichia coli O157:H7 str. Sakai	Effector delivery system	eivE	type III secretion protein EivE	100	1.51E-267	726
ECs_3735	ECs_3735	hypothetical protein	VFG042074 (gb|NP_311762)	ETT2 (VF1161)	-	setB	Escherichia coli O157:H7 str. Sakai	Effector delivery system	ECs_3735	hypothetical protein	100	2.45E-32	105
ECs_5775	ghoT	toxin of GhoTS toxin-antitoxin pair	VFG042074 (gb|NP_311762)	ETT2 (VF1161)	-	setB	Escherichia coli O157:H7 str. Sakai	Effector delivery system	ECs_3735	hypothetical protein	40.9	2.67E-06	39.7
ECs_3734	eivF	type III secretion protein EivF	VFG042073 (gb|NP_311761)	ETT2 (VF1161)	-	setA	Escherichia coli O157:H7 str. Sakai	Effector delivery system	eivF	type III secretion protein EivF	100	1.42E-176	485
ECs_5089	yjdN	metalloprotein superfamily	VFG039368 (gb|WP_005768971)	T4SS secreted effectors (VF0696)	CBUA0020; CBU_0012*; CBU_0113; CBU_0122; CBU_0183; CBU_0201; CBU_0270; CBU_0295; CBU_0344*; CBU_0372; CBU_0375*; CBU_0469;	setA	Coxiella burnetii CbuK_Q154	Effector delivery system	phnB	Coxiella Dot/Icm type IVB secretion	27	6.16E-07	46.2

protein	CBU_0513; CBU_0534; CBU_0590; CBU_0635; CBU_0637; CBU_0820*; CBU_1048*; CBU_1079; CBU_1107*; CBU_1150*; CBU_1198; CBU_1268; CBU_1349; CBU_1370; CBU_1409; CBU_1434; CBU_1493; CBU_1495*; CBU_1525*; CBU_1530; CBU_1566; CBU_1576; CBU_1594; CBU_1607; CBU_1614; CBU_1639; CBU_1665; CBU_1677; CBU_1685; CBU_1752; CBU_1754; CBU_1789; CBU_1790; CBU_1794; CBU_1818; CBU_1819; CBU_1863; CBU_2016; CBU_2028; CBU_2056; CBU_2059*; CBU_2076; AnkA; AnkB; AnkF; AnkG (Interacts with host protein p32 to block apoptosis.); AnkH; AnkI; AnkM/cig58; AnkP; Cem1; Cem12; Cem13; Cem3; Cem4; Cem6; Cem9; CetCb1; CetCb2; CetCb3; CetCB4; CetCb5; CetCb6; CirA/coxCC1 (Phosphate transporter family protein.); CirB; CirC/coxDFB1; CoxCC10/cig49; CoxCC11; CoxCC12; CoxCC14; CoxCC15; CoxCC3; CoxCC4; CoxCC5; CoxCC6; CoxCC7/cig44; CoxCC8; CoxDFB3; CoxDFB4 (Surface antigen.);	system translocated effector

					CoxDFB5/cig57; CoxDFB6; CoxFIC1; CoxH2/rimL (Acetyltransferase.); CoxH3; CoxH4/cig61; CoxK1 (Protein kinase, putative.); CoxK2; CoxTPR1 (Conserved domain protein.); CoxU1; CoxU2; CpeA; CpeB; CpeC/coxU3 (Hypothetical protein plasmid QpH1.); CpeD; CpeE; CpeF; CpeG; CpeH; CvpA; MceA; PhnB; CBUD_RS05145; CBUD_RS06720*; CBUD_RS08635; CBUD_RS11275; CBUD_RS12405; CBUG_RS02435; CBUK_RS06760								
ECs_5088	phnC	phosphonate ABC transporter ATPase	VFG048481 (gb|WP_014228352)	Ent (VF0562)	May be necessary for penetration to deeper tissues	setA	Klebsiella oxytoca KCTC 1686	Nutritional/Metabolic factor	fepC	iron-enterobactin transporter ATP-binding protein	31.1	7.12E-30	112
ECs_0155	fhuC	iron(3+)-hydroxamate import ABC transporter ATPase	VFG048481 (gb|WP_014228352)	Ent (VF0562)	May be necessary for penetration to deeper tissues	setA	Klebsiella oxytoca KCTC 1686	Nutritional/Metabolic factor	fepC	iron-enterobactin transporter ATP-binding protein	44.5	1.57E-69	215
ECs_0627	fepC	ferrienterobactin	VFG020177 (gb|WP_00	Enterobactin	-	setA	Shigella boydii CDC	Nutritional/Metaboli	fepC	iron-enterobactin ABC	98.9	2.78E-194	531

		ABC transporter ATPase	0140632)	synthesis and transport (VF0984)			3083-94	c factor		transporter ATP-binding protein			
ECs_4188	hopD	leader peptidase HopD	VFG013806 (gb|WP_011920936)	Type IV pili (VF0082)	Attaches to host cells, but not to mucin, causing a twitching motility that allows the bacteria to move along the cell surface; biofilm formation	setA	Pseudomonas mendocina ymp	Adherence	xcpA/pilD	type 4 prepilin peptidase PilD	44	2.70E-34	121
gene-etpN	etpN	Type II secretion pathway related protein	VFG038316 (gb|WP_011707571)	Tap type IV pili (VF0475)	The role of the Tap pilus remains to be elucidated, its biogenesis has strong implications in virulence in other ways. TapD, a prepilin peptidase is not only required for the processing of the prepilins before they are incorporated into the pilus filament but also for the processing of the pseudopilins of the T2SS, that are essential for the secretion of many virulence factors	setA	Aeromonas hydrophila subsp. hydrophila ATCC 7966	Adherence	tapD/pilD	prepilin peptidase	50	4.91E-75	229
ECs_2845	ECs_2845	glycosyl transferase	VFG005021 (gb|WP_011302065)	Capsule (VF0003)	Prevent phagocytosis	setA	Staphylococcus saprophyticus subsp. saprophyticus ATCC 15305	Immune modulation	SSP_RS00325	glycosyltransferase family 2 protein	34.5	9.53E-44	148
ECs_2864	wcaA	glycosyl	VFG005021	Capsule	Prevent phagocytosis	setA	Staphyloco	Immune	SSP_RS0032	glycosyltr	30.4	2.40E-	79.3

		transferase	(gb|WP_011302065)	(VF0003)			ccus saprophyticus subsp. saprophyticus ATCC 15305	modulation	5	ansferase family 2 protein		17	
ECs_4108	degS	serine endoprotease	VFG014995 (gb|WP_011921011)	Alginate (VF0091)	Allows the bacteria form biofilm; contributes to the persistence of the bacteria in the CF lung: act as an adhesin, preventing the bacteria from being expelled from the lung, and alginate slime layer makes it more difficult for phagocytes to ingest and kill the bacteria	setA	Pseudomonas mendocina ymp	Biofilm	algW	AlgW protein	44.9	1.36E-84	261
gene-etpH	etpH	Type II secretion pathway related protein	VFG040936 (gb|WP_000082929)	Etp (VF1178)	-	setA	Escherichia coli O157:H7 str. EDL933	Effector delivery system	gspH	type II secretion system minor pseudopilin GspH	100	2.66E-133	370
ECs_2512	yeaX	putative dioxygenase beta subunit	VFG002705 (gb|WP_002208598)	O-antigen (VF0392)	LPS O antigen mutants were severely impaired in their ability to colonize the Peyer's patches and did not colonize spleen and liver. The absence of O antigen in the outer membrane affects the expression of other	setB	Yersinia pestis CO92	Immune modulation	YPO_RS16520	CDP-6-deoxy-delta-3,4-glucoseen reductase	36.7	9.42E-07	49.7

					Yersinia virulence factors.								
ECs_3119	yfaE	ferredoxin involved with ribonucleotide reductase	VFG002705 (gb|WP_002208598)	O-antigen (VF0392)	LPS O antigen mutants were severely impaired in their ability to colonize the Peyer's patches and did not colonize spleen and liver. The absence of O antigen in the outer membrane affects the expression of other Yersinia virulence factors.	setB	Yersinia pestis CO92	Immune modulation	YPO_RS16520	CDP-6-deoxy-delta-3, 4-glucoseen reductase	42.2	5.38E-12	59.3
ECs_4772	fre	NAD(P)H-flavin reductase	VFG002705 (gb|WP_002208598)	O-antigen (VF0392)	LPS O antigen mutants were severely impaired in their ability to colonize the Peyer's patches and did not colonize spleen and liver. The absence of O antigen in the outer membrane affects the expression of other Yersinia virulence factors.	setB	Yersinia pestis CO92	Immune modulation	YPO_RS16520	CDP-6-deoxy-delta-3, 4-glucoseen reductase	30.9	7.76E-32	118
ECs_1031	ycbX	6-N-hydroxylaminopurine detoxification oxidoreductase	VFG002705 (gb|WP_002208598)	O-antigen (VF0392)	LPS O antigen mutants were severely impaired in their ability to colonize the Peyer's patches and did not colonize spleen and liver. The absence of O antigen in the outer membrane affects the expression of other Yersinia virulence factors.	setB	Yersinia pestis CO92	Immune modulation	YPO_RS16520	CDP-6-deoxy-delta-3, 4-glucoseen reductase	30.5	2.91E-06	48.5
ECs_0958	hcr	HCP oxidoreductase	VFG002705 (gb|WP_002208598)	O-antigen (VF0392)	LPS O antigen mutants were severely impaired in their ability to colonize the Peyer's patches and did not colonize spleen and liver. The absence	setB	Yersinia pestis CO92	Immune modulation	YPO_RS16520	CDP-6-deoxy-delta-3, 4-glucoseen reductase	25.7	2.93E-08	54.3

					of O antigen in the outer membrane affects the expression of other Yersinia virulence factors.							
ECs_1416	csgE	curlin secretion specificity factor	VFG045790 (gb|WP_000833288)	Curli fibers (VF1138)	In E. coli, curli fibers compose up to 85% of the biofilm biomass. Curli acts in vitro as an essential scaffold protein during biofilm formation.;In vivo, curli also directly regulates the immune system and is known to induce inflammation by activating the immune Toll-like receptors (TLRs).	setA	Escherichia coli O25b:H4-ST131	Adherence	cgsE	curli production assembly/transport protein CsgE	100	6.29E-94	266
ECs_1417	csgD	transcriptional regulator	VFG045791 (gb|WP_000481500)	Curli fibers (VF1138)	In E. coli, curli fibers compose up to 85% of the biofilm biomass. Curli acts in vitro as an essential scaffold protein during biofilm formation.;In vivo, curli also directly regulates the immune system and is known to induce inflammation by activating the immune Toll-like receptors (TLRs).	setA	Escherichia coli O25b:H4-ST131	Adherence	cgsD	transcriptional regulator CsgD	100	3.53E-156	431
ECs_1414	csgG	curli production assembly/transport outer membrane	VFG042725 (gb|WP_001189321)	Curli fibers (VF1138)	In E. coli, curli fibers compose up to 85% of the biofilm biomass. Curli acts in vitro as an essential scaffold protein during biofilm formation.;In vivo, curli also directly regulates the immune system and is known to induce inflammation by activating the	setA	Escherichia coli O157:H7 str. EDL933	Adherence	csgG	curli production assembly/transport protein CsgG	100	5.62E-197	539

		lipoprotein			immune Toll-like receptors (TLRs).							
ECs_1415	csgF	curli nucleation outer membrane protein	VFG046069 (gb|WP_001264088)	Curli fibers (VF1138)	In E. coli, curli fibers compose up to 85% of the biofilm biomass. Curli acts in vitro as an essential scaffold protein during biofilm formation.;In vivo, curli also directly regulates the immune system and is known to induce inflammation by activating the immune Toll-like receptors (TLRs).	setA	Escherichia coli VR50	Adherence	cgsF	curli production assembly/transport protein CsgF	100	6.36E-96	272
ECs_1419	csgB	curlin nucleator protein	VFG045792 (gb|WP_000791650)	Curli fibers (VF1138)	In E. coli, curli fibers compose up to 85% of the biofilm biomass. Curli acts in vitro as an essential scaffold protein during biofilm formation.;In vivo, curli also directly regulates the immune system and is known to induce inflammation by activating the immune Toll-like receptors (TLRs).	setA	Escherichia coli O25b:H4-ST131	Adherence	csgB	curlin minor subunit CsgB	100	1.32E-38	128
gene-sopB	sopB	BAA31798.2	VFG012636 (gb|NP_858256)	TTSS (VF0118)	Mxi-Spa system secretes approximately 20 proteins with the four Ipas A, B,C,D and IpgD being the most abundant; Recent findings indicate that TTSS in Shigella is responsible not only for protein secretion, but that it is also involved in the control mechanisms of transcription of other target genes	setA	Shigella flexneri 2a str. 301	Effector delivery system	virB	transcriptional activator VirB	33.3	8.66E-13	67.8

					located on the virulence plasmid, virA and ipaH9.8, these proteins were not constitutively synthesized and stored in the bacterial cytoplasm; their expression was markedly increased after initial activation of the secretion system, virA is not required for entry;Upon contact of the tip of the needle with the plasma membrane, the injectisome secretes its protein substrates into host cells. Some of these substrates act as translocators or effectors whose functions are key to the invasion of the cytosol and the cell-to-cell spread characterizing the lifestyle of Shigella spp.								
ECs_1257	rutB	ureidoacrylate amidohydrolase	VFG007198 (gb|WP_011081755)	Vibriobactin/Vulnibactin (VF0626)	-	setA	Vibrio vulnificus CMCP6	Nutritional/Metabolic factor	vibB	isochorismatase family protein	29.4	1.26E-09	57
ECs_0634	entB	isochorismatase	VFG000933 (gb|WP_001007140)	Enterobactin (VF0228)	Iron uptake: the siderophore enterobactin imported through the FepA receptor and the FepBCDG system	setA	Escherichia coli CFT073	Nutritional/Metabolic factor	entB	isochorismatase	99.6	1.46E-212	579
ECs_1258	rutA	pyrimidine oxygenase	VFG029361 (gb|WP_003876826)	PDIM (VF0309)	Play a mainly structural role in providing a stable base for the insertion of other lipid and also play	setB	Mycobacterium avium subsp.	Immune modulation	MAP4_RS19005	LLM class flavin-dependent	28.7	1.40E-09	58.9

					a role as a fluidity modifier, whose function could be to modulate cell wall viscosity;PDIM is crucial for infection by masking pathogen-associated molecular patterns (PAMP) of the cell wall from the innate immune system		paratuberculosis MAP4			oxidoreductase			
ECs_0075	leuD	3-isopropylmalate dehydratase small subunit	VFG009376 (gb|WP_003893754)	Leucine synthesis (VF0814)	-	setB	Mycobacterium smegmatis str. MC2 155	Nutritional/Metabolic factor	leuD	3-isopropylmalate dehydratase small subunit	47.2	2.70E-56	176
ECs_1077	ECs_1077	hypothetical protein	VFG048530 (gb|WP_014838862)	Sal (VF0563)	A major trigger of inflammation and bacterial dissemination induced during K. pneuminiae lung infection	setA	Klebsiella oxytoca E718	Nutritional/Metabolic factor	iroE	siderophore esterase IroE	29.5	3.21E-26	103
ECs_1890	ycjO	sugar ABC transporter permease	VFG030584 (gb|WP_013828148)	Trehalose-recycling ABC transporter (VF0842)	-	setB	Mycobacterium sp. JDM601	Nutritional/Metabolic factor	sugA	sugar ABC transporter permease	31.2	8.82E-34	124
ECs_1894	ycjS	NADH-binding oxidoreductase	VFG014100 (gb|NP_251848)	LPS (VF0085)	Mediates biological effects including resistance to serum killing and phagocytosis; the binding to normal CFTR (cystic fibrosis transmembrane conductance regulator) and invasion of	setA	Pseudomonas aeruginosa PAO1	Immune modulation	wbpB	UDP-N-acetyl-2-amino-2-deoxy-D-glucuronate oxidase	31.1	1.05E-08	55.8

					host cells may make a contribution to virulence in the human eye; internalization by binding to normal CFTR protein expressed by airway epithelial cells followed by desquamation of bacteria-laden epithelial cells, constitutes a host defense mechanism. If this mechanism fails to function properly, abnormally high bacterial carriage would promote the establishment of chronic bacterial infection								
ECs_0474	dxs	1-deoxy-D-xylulose-5-phosphate synthase	VFG016499 (gb|WP_011206101)	PDH-B (VF0878)	-	setB	Mycoplasma hyopneumoniae 232	Adherence	pdhB	alpha-keto acid dehydrogenase subunit beta	24	3.93E-08	55.5
ECs_0472	espY3	T3SS effector-like protein EspY	VFG034908 (gb|WP_000978349)	TTSS secreted effectors (VF1110)	Cif (Deamidase. Induces cytopathic effects of actin stress fiber formation and cell cycle arrest.); EspB (Pore formation, actin disruption, microvilli effacement, anti-phagocytosis.); EspF (Inducing degradation of the aniapoptic protein AbcF2, tight junction disruption, microvilli effacement and elongation,	setA	Escherichia coli O157:H7 str. EDL933	Effector delivery system	espY3	Type III secretion system effector EspY3	100	0	947

mitochondrial dysfunction, N-WASP activation, SGLT-1 inactivation, pedestal maturation, inhibition of NHE3 activity, membrane remodelling; targets and disrupts the nucleolus late in infection, which is temporally controlled by host mitochondria.); EspFu/tccP (Inducing degradation of the aniapoptic protein AbcF2, tight junction disruption, microvilli effacement and elongation, mitochondrial dysfunction, N-WASP activation, SGLT-1 inactivation, pedestal maturation, inhibition of NHE3 activity, membrane remodelling; targets and disrupts the nucleolus late in infection, which is temporally controlled by host mitochondria.); EspG (TBC-like GTPase activating protein. Efficiently catalyzes GTP hydrolysis in Rab1 to disrupt of Rab1-mediated ER-to-Golgi trafficking.); EspH (First bacterial effector acting directly on RhoGEFs, EspH directly binds to the DH-PH domain in RhoGEFs to disrupt RhoGEF-Rho

signaling; critical for inhibiting macrophage phagocytosis.); EspJ (Inhibit both IgG- and complement receptor-mediated phagocytosis.); EspK; EspL1; EspL2 (Cysteine protease. Bounds F-actin-aggregating annexin 2 directly to increase annexin 2's ability to aggregate Tir-induced F-actin; block necroptosis and in flammation.); EspL4; EspM1 (GEF. Activates the RhoA signaling pathway and induce the formation of stress fibres; inhibit pedestal formation and induce tight junction mislocalization.); EspM2 (GEF. Activates the RhoA signaling pathway and induce the formation of stress fibres; inhibit pedestal formation and induce tight junction mislocalization.); EspN; EspO1-1; EspO1-2; EspR1; EspR3; EspR4; EspT (GEF. Activates Rac1 and Cdc42 leading to formation of membrane ruffles and lamellipodia; induces membrane ruffles to facilitate bacterial invasion into non-phagocytic cells in

a process involving Rac1 and Wave2.); EspW; EspX1; EspX2; EspX4; EspX5; EspX6; EspX7/nleL (E3 ubiquitin ligase, HECT-like. Modulates pedestal formation.); EspY1; EspY2; EspY3; EspY4; EspY5; Map (GEF. Mimics the host Dbl and catalyses the exchange of GDP for GTP in Cdc42, involved in effacement, SGLT1 inhibition, formation of filopodia and disruption of mitochondrial function.); NleA/espI (Disruption of tight junctions by inhibition of host cell protein trafficking through COPII-dependent pathways.); NleB1 (Blocks translocation of the p65 and to the host cell nucleus to inhibit NF-<kappa>B pathway, but NleE and NleB act at different points in the NF-<kappa>B signaling pathway.); NleB2 (May also have anti-inflammatory activity.); NleC (Metalloprotease. Zn-dependent endopeptidases that specifically clip and inactivate RelA (p65), thus blocking NF-<kappa>B pathway.); NleD (Metalloprotease.

Zn-dependent endopeptidases that specifically clip and inactivate JNK and p38, thus blocking AP-1 pathway.); NleE (PMN tran-epithelial migration; blocks translocation of the p65 to the host cell nucleus by preventing I<kappa>B degradation to inhibit NF-<kappa>B pathway.); NleF; NleG-1; NleG2-2; NleG2-3; NleG2-4; NleG5-1; NleG5-2; NleG6-1; NleG6-2; NleG6-3; NleG7 (U-box type E3 ubiquitin ligases.); NleG8-2; NleH1 (Ser/Thr protein kinase. Binds directly to a subunit of NF-<kappa>B, the ribosomal protein S3 (RPS3), reducing the nuclear abundance of RPS3 to dampen host transcriptional outputs; interact with Bax inhibitor-1 to block apoptosis.); NleH2 (Putative kinase. Attenuates NF-<kappa>B pathway.); SepZ/espZ (EspZ interacts with CD98 in host cell membranes to promote host cell survival, therefore provide the pathogen with valuable time to colonize efficiently prior to dissemination.); TccP2; Tir (Mimics

					host immunoreceptor tyrosine-based inhibition motifs (ITIMs), also see helicobacter CagA. EHEC Tir lacks the Nck binding site. Conserved NPY (Asn-Pro-Tyr) motif recruits the adaptor protein IRTKS and/or IRSp53. IRTKS/IRSp53 link Tir and TccP/EspFu, which in turn activates N-WASP; Receptor for intimin; effacement; SGLT1 inhibition; recruits SHIP2 to control actin-pedestal morphology; maintains the integrity of the epithelium by keeping the destructive activity of EspG and EspG2 in check.)								
ECs_3439	rpoE	RNA polymerase sigma E factor	VFG000121 (gb|NP_249453)	Alginate (VF0091)	Allows the bacteria form biofilm; contributes to the persistence of the bacteria in the CF lung: act as an adhesin, preventing the bacteria from being expelled from the lung, and alginate slime layer makes it more difficult for phagocytes to ingest and kill the bacteria	setA	Pseudomonas aeruginosa PAO1	Biofilm	algU	alginate biosynthesis protein AlgZ/FimS	66	1.65E-85	250
ECs_4791	glnL	two-component system sensor	VFG013998 (gb|WP_003403505)	Type IV pili (VF0082)	Attaches to host cells, but not to mucin, causing a twitching motility that allows the bacteria to move along the cell surface; biofilm formation	setA	Pseudomonas syringae pv. syringae	Adherence	pilS	two-component sensor PilS	25.9	7.59E-19	87.4

		histidine kinase GlnL					B728a					
ECs_0612	cusB	membrane fusion protein of copper/silver efflux system	VFG036978 (gb|WP_004465401)	MtrCDE (VF0451)	A tripartite multidrug efflux pump essential for resistance to <beta>-lactams (penicillin G and nafcillin), macrolides (erythromycin) and host-derived compounds (peptide LL-37) and progesterone, and essential for growth of gonococci in the lower genital tract of experimentally infected female mice	setA	Neisseria gonorrhoeae NCCP11945	Antimicrobial activity/Competitive advantage	mtrC	membrane fusion protein MtrC	27.7	1.94E-08	55.8
ECs_3858	nleE	T3SS secreted effector NleE	VFG034954 (gb|WP_000609742)	TTSS secreted effectors (VF1110)	Cif (Deamidase. Induces cytopathic effects of actin stress fiber formation and cell cycle arrest.); EspB (Pore formation, actin disruption, microvilli effacement, anti-phagocytosis.); EspF (Inducing degradation of the aniapoptic protein AbcF2, tight junction disruption, microvilli effacement and elongation, mitochondrial dysfunction, N-WASP activation, SGLT-1 inactivation, pedestal maturation, inhibition of NHE3 activity, membrane remodelling; targets and disrupts the nucleolus	setA	Escherichia coli O157:H7 str. EDL933	Effector delivery system	nleE	Type III secretion system effector NleE	100	1.69E-167	460

late in infection, which is temporally controlled by host mitochondria.); EspFu/tccP (Inducing degradation of the aniapoptic protein AbcF2, tight junction disruption, microvilli effacement and elongation, mitochondrial dysfunction, N-WASP activation, SGLT-1 inactivation, pedestal maturation, inhibition of NHE3 activity, membrane remodelling; targets and disrupts the nucleolus late in infection, which is temporally controlled by host mitochondria.); EspG (TBC-like GTPase activating protein. Efficiently catalyzes GTP hydrolysis in Rab1 to disrupt of Rab1-mediated ER-to-Golgi trafficking.); EspH (First bacterial effector acting directly on RhoGEFs, EspH directly binds to the DH-PH domain in RhoGEFs to disrupt RhoGEF-Rho signaling; critical for inhibiting macrophage phagocytosis.); EspJ (Inhibit both IgG- and complement receptor-mediated phagocytosis.); EspK; EspL1; EspL2 (Cysteine protease.

Bounds F-actin-aggregating annexin 2 directly to increase annexin 2's ability to aggregate Tir-induced F-actin; block necroptosis and in flammation.); EspL4; EspM1 (GEF. Activates the RhoA signaling pathway and induce the formation of stress fibres; inhibit pedestal formation and induce tight junction mislocalization.); EspM2 (GEF. Activates the RhoA signaling pathway and induce the formation of stress fibres; inhibit pedestal formation and induce tight junction mislocalization.); EspN; EspO1-1; EspO1-2; EspR1; EspR3; EspR4; EspT (GEF. Activates Rac1 and Cdc42 leading to formation of membrane ruffles and lamellipodia; induces membrane ruffles to facilitate bacterial invasion into non-phagocytic cells in a process involving Rac1 and Wave2.); EspW; EspX1; EspX2; EspX4; EspX5; EspX6; EspX7/nleL (E3 ubiquitin ligase, HECT-like. Modulates pedestal formation.); EspY1; EspY2; EspY3;

EspY4; EspY5; Map (GEF. Mimics the host Dbl and catalyses the exchange of GDP for GTP in Cdc42, involved in effacement, SGLT1 inhibition, formation of filopodia and disruption of mitochondrial function.); NleA/espI (Disruption of tight junctions by inhibition of host cell protein trafficking through COPII-dependent pathways.); NleB1 (Blocks translocation of the p65 and to the host cell nucleus to inhibit NF-<kappa>B pathway, but NleE and NleB act at different points in the NF-<kappa>B signaling pathway.); NleB2 (May also have anti-inflammatory activity.); NleC (Metalloprotease. Zn-dependent endopeptidases that specifically clip and inactivate RelA (p65), thus blocking NF-<kappa>B pathway.); NleD (Metalloprotease. Zn-dependent endopeptidases that specifically clip and inactivate JNK and p38, thus blocking AP-1 pathway.); NleE (PMN tran-epithelial migration; blocks translocation of the p65 to the

host cell nucleus by preventing I<kappa>B degradation to inhibit NF-<kappa>B pathway.); NleF; NleG-1; NleG2-2; NleG2-3; NleG2-4; NleG5-1; NleG5-2; NleG6-1; NleG6-2; NleG6-3; NleG7 (U-box type E3 ubiquitin ligases.); NleG8-2; NleH1 (Ser/Thr protein kinase. Binds directly to a subunit of NF-<kappa>B, the ribosomal protein S3 (RPS3), reducing the nuclear abundance of RPS3 to dampen host transcriptional outputs; interact with Bax inhibitor-1 to block apoptosis.); NleH2 (Putative kinase. Attenuates NF-<kappa>B pathway.); SepZ/espZ (EspZ interacts with CD98 in host cell membranes to promote host cell survival, therefore provide the pathogen with valuable time to colonize efficiently prior to dissemination.); TccP2; Tir (Mimics host immunoreceptor tyrosine-based inhibition motifs (ITIMs), also see helicobacter CagA. EHEC Tir lacks the Nck binding site. Conserved NPY (Asn-Pro-Tyr) motif recruits the

					adaptor protein IRTKS and/or IRSp53. IRTKS/IRSp53 link Tir and TccP/EspFu, which in turn activates N-WASP; Receptor for intimin; effacement; SGLT1 inhibition; recruits SHIP2 to control actin-pedestal morphology; maintains the integrity of the epithelium by keeping the destructive activity of EspG and EspG2 in check.)								
ECs_1677	hlyE	hemolysin E	VFG036044 (gb|WP_001304191)	Hemolysin/cytolysin A (VF1134)	-	setB	Escherichia coli O157:H7 str. EDL933	Exotoxin	hlyE/clyA	hemolysin HlyE	100	4.84E-211	577
ECs_0102	secA	preprotein translocase subunit	VFG009161 (gb|WP_005067995)	Accessory secretion factor (VF0808)	-	setB	Mycobacterium abscessus subsp. bolletii 50594	Others	secA2	accessory Sec system translocase SecA2	35.6	1.08E-130	410
ECs_0100	lpxC	UDP-3-O-acyl N-acetylglucosamine deacetylase	VFG013414 (gb|WP_012054478)	LOS (VF0044)	Major immunogen; LOS phosphorylcholine (ChoP) may influence invasion via interaction with PAF receptor and stimulates of inflammatory signals; LPS phase variation is characterized by the spontaneous loss and gain of	setA	Haemophilus influenzae PittEE	Immune modulation	lpxC	UDP-3-O-(R-3-hydroxymyristoyl)-N-acetylglucosamine deacetylase	77.3	8.05E-172	478

					oligosaccharide structures present in the outer core. the phase variable expression of LPS biosynthesis genes promotes evasion of antigen-specific host immune defences and allow colonization of different host microenvironments								
ECs_4918	rsd	anti-RNA polymerase sigma 70 factor	VFG019929 (gb|WP_015886487)	Alginate (VF0091)	Allows the bacteria form biofilm; contributes to the persistence of the bacteria in the CF lung: act as an adhesin, preventing the bacteria from being expelled from the lung, and alginate slime layer makes it more difficult for phagocytes to ingest and kill the bacteria	setA	Pseudomonas fluorescens SBW25	Biofilm	algQ	Alginate regulatory protein AlgQ	34.8	1.08E-19	80.1
ECs_3719	ECs_3719	type III secretion protein EprH	VFG042087 (gb|NP_311746)	ETT2 (VF1161)	-	setA	Escherichia coli O157:H7 str. Sakai	Effector delivery system	ECs_3719	type III secretion protein EprH	100	4.60E-175	481
ECs_0850	nleD	T3SS secreted effector NleD	VFG012445 (gb|NP_308877)	TTSS secreted effectors (VF1110)	Cif (Deamidase. Induces cytopathic effects of actin stress fiber formation and cell cycle arrest.); EspB (Pore formation, actin disruption, microvilli effacement, anti-phagocytosis.); EspF (Inducing degradation of the aniapoptic protein	setA	Escherichia coli O157:H7 str. Sakai	Effector delivery system	nleD	Type III secretion system effector NleD	100	1.05E-165	456

AbcF2, tight junction disruption, microvilli effacement and elongation, mitochondrial dysfunction, N-WASP activation, SGLT-1 inactivation, pedestal maturation, inhibition of NHE3 activity, membrane remodelling; targets and disrupts the nucleolus late in infection, which is temporally controlled by host mitochondria.); EspFu/tccP (Inducing degradation of the aniapoptic protein AbcF2, tight junction disruption, microvilli effacement and elongation, mitochondrial dysfunction, N-WASP activation, SGLT-1 inactivation, pedestal maturation, inhibition of NHE3 activity, membrane remodelling; targets and disrupts the nucleolus late in infection, which is temporally controlled by host mitochondria.); EspG (TBC-like GTPase activating protein. Efficiently catalyzes GTP hydrolysis in Rab1 to disrupt of Rab1-mediated ER-to-Golgi trafficking.); EspH (First bacterial effector acting directly on RhoGEFs,

EspH directly binds to the DH-PH domain in RhoGEFs to disrupt RhoGEF-Rho signaling; critical for inhibiting macrophage phagocytosis.); EspJ (Inhibit both IgG- and complement receptor-mediated phagocytosis.); EspK; EspL1; EspL2 (Cysteine protease. Bounds F-actin-aggregating annexin 2 directly to increase annexin 2's ability to aggregate Tir-induced F-actin; block necroptosis and in flammation.); EspL4; EspM1 (GEF. Activates the RhoA signaling pathway and induce the formation of stress fibres; inhibit pedestal formation and induce tight junction mislocalization.); EspM2 (GEF. Activates the RhoA signaling pathway and induce the formation of stress fibres; inhibit pedestal formation and induce tight junction mislocalization.); EspN; EspO1-1; EspO1-2; EspR1; EspR3; EspR4; EspT (GEF. Activates Rac1 and Cdc42 leading to formation of membrane ruffles and lamellipodia; induces membrane

ruffles to facilitate bacterial invasion into non-phagocytic cells in a process involving Rac1 and Wave2.); EspW; EspX1; EspX2; EspX4; EspX5; EspX6; EspX7/nleL (E3 ubiquitin ligase, HECT-like. Modulates pedestal formation.); EspY1; EspY2; EspY3; EspY4; EspY5; Map (GEF. Mimics the host Dbl and catalyses the exchange of GDP for GTP in Cdc42, involved in effacement, SGLT1 inhibition, formation of filopodia and disruption of mitochondrial function.); NleA/espI (Disruption of tight junctions by inhibition of host cell protein trafficking through COPII-dependent pathways.); NleB1 (Blocks translocation of the p65 and to the host cell nucleus to inhibit NF-<kappa>B pathway, but NleE and NleB act at different points in the NF-<kappa>B signaling pathway.); NleB2 (May also have anti-inflammatory activity.); NleC (Metalloprotease. Zn-dependent endopeptidases that specifically clip and inactivate RelA

(p65), thus blocking NF-<kappa>B pathway.); NleD (Metalloprotease. Zn-dependent endopeptidases that specifically clip and inactivate JNK and p38, thus blocking AP-1 pathway.); NleE (PMN tran-epithelial migration; blocks translocation of the p65 to the host cell nucleus by preventing I<kappa>B degradation to inhibit NF-<kappa>B pathway.); NleF; NleG-1; NleG2-2; NleG2-3; NleG2-4; NleG5-1; NleG5-2; NleG6-1; NleG6-2; NleG6-3; NleG7 (U-box type E3 ubiquitin ligases.); NleG8-2; NleH1 (Ser/Thr protein kinase. Binds directly to a subunit of NF-<kappa>B, the ribosomal protein S3 (RPS3), reducing the nuclear abundance of RPS3 to dampen host transcriptional outputs; interact with Bax inhibitor-1 to block apoptosis.); NleH2 (Putative kinase. Attenuates NF-<kappa>B pathway.); SepZ/espZ (EspZ interacts with CD98 in host cell membranes to promote host cell survival, therefore provide the pathogen with valuable time to

					colonize efficiently prior to dissemination.); TccP2; Tir (Mimics host immunoreceptor tyrosine-based inhibition motifs (ITIMs), also see helicobacter CagA. EHEC Tir lacks the Nck binding site. Conserved NPY (Asn-Pro-Tyr) motif recruits the adaptor protein IRTKS and/or IRSp53. IRTKS/IRSp53 link Tir and TccP/EspFu, which in turn activates N-WASP; Receptor for intimin; effacement; SGLT1 inhibition; recruits SHIP2 to control actin-pedestal morphology; maintains the integrity of the epithelium by keeping the destructive activity of EspG and EspG2 in check.)								
ECs_0853	bioB	biotin synthase	VFG047505 (gb|WP_003026016)	Biotin synthesis (VF0552)	Critical for rapid phagosomal escape. Sequestration of biotin could restrict Francisella to the phagosome, blocking their escape and preventing them from reaching their replicative niche in the cytoplasm	setA	Francisella novicida U112	Nutritional/Metabolic factor	bioB	biotin synthase	50.7	1.49E-110	324
ECs_0852	bioA	7%2C8-diaminopelargonic acid	VFG047527 (gb|WP_012429464)	Biotin synthesis (VF0552)	Critical for rapid phagosomal escape. Sequestration of biotin could restrict Francisella to the phagosome, blocking their escape and preventing them from	setA	Francisella tularensis subsp.	Nutritional/Metabolic factor	bioA	adenosylmethionine-8-amino-7-oxononanoat	31.7	1.47E-59	200

		synthase			reaching their replicative niche in the cytoplasm		mediasiatica FSC147			e aminotransferase			
ECs_2454	astC	succinylornithine transaminase	VFG047534 (gb|WP_014549695)	Biotin synthesis (VF0552)	Critical for rapid phagosomal escape. Sequestration of biotin could restrict Francisella to the phagosome, blocking their escape and preventing them from reaching their replicative niche in the cytoplasm	setA	Francisella cf. novicida Fx1	Nutritional/Metabolic factor	bioA	adenosylmethionine-8-amino-7-oxononanoate aminotransferase	28.4	1.49E-43	157
ECs_0855	bioC	malonyl-ACP O-methyltransferase	VFG007971 (gb|WP_010907549)	PDIM (VF0309)	Play a mainly structural role in providing a stable base for the insertion of other lipid and also play a role as a fluidity modifier, whose function could be to modulate cell wall viscosity;PDIM is crucial for infection by masking pathogen-associated molecular patterns (PAMP) of the cell wall from the innate immune system	setB	Mycobacterium leprae TN	Immune modulation	ML_RS00650	class I SAM-dependent methyltransferase	33	2.56E-06	47.4
ECs_0854	bioF	8-amino-7-oxononanoate synthase	VFG047493 (gb|WP_012429462)	Biotin synthesis (VF0552)	Critical for rapid phagosomal escape. Sequestration of biotin could restrict Francisella to the phagosome, blocking their escape and preventing them from reaching their replicative niche in the cytoplasm	setA	Francisella tularensis subsp. mediasiatica FSC147	Nutritional/Metabolic factor	bioF	8-amino-7-oxononanoate synthase	33.8	8.30E-56	187

ECs_0856	bioD	dethiobiotin synthetase	VFG047470 (gb|WP_013923074)	Biotin synthesis (VF0552)	Critical for rapid phagosomal escape. Sequestration of biotin could restrict Francisella to the phagosome, blocking their escape and preventing them from reaching their replicative niche in the cytoplasm	setA	Francisella sp. TX077308	Nutritional/Metabolic factor	bioD	dethiobiotin synthetase	36.9	1.19E-29	110
ECs_2299	ynfK	dethiobiotin synthetase	VFG047470 (gb|WP_013923074)	Biotin synthesis (VF0552)	Critical for rapid phagosomal escape. Sequestration of biotin could restrict Francisella to the phagosome, blocking their escape and preventing them from reaching their replicative niche in the cytoplasm	setA	Francisella sp. TX077308	Nutritional/Metabolic factor	bioD	dethiobiotin synthetase	27.4	4.44E-19	82.4
gene-etpJ	etpJ	Type II secretion pathway related protein	VFG040938 (gb|WP_000082782)	Etp (VF1178)	-	setA	Escherichia coli O157:H7 str. EDL933	Effector delivery system	gspJ	type II secretion system minor pseudopilin GspJ	100	2.07E-142	394
gene-etpK	etpK	Type II secretion pathway related protein	VFG040939 (gb|WP_000776550)	Etp (VF1178)	-	setA	Escherichia coli O157:H7 str. EDL933	Effector delivery system	gspK	type II secretion system minor pseudopilin GspK	100	9.61E-229	623
gene-etpI	etpI	Type II secretion	VFG040937 (gb|WP_00	Etp (VF1178)	-	setA	Escherichia coli	Effector delivery	gspI	type II secretion	100	2.23E-76	221

		pathway related protein	0173396)				O157:H7 str. EDL933	system		system minor pseudopilin GspI			
gene-etpO	etpO	Type II secretion pathway related protein	VFG040943 (gb|WP_000971918)	Etp (VF1178)	-	setA	Escherichia coli O157:H7 str. EDL933	Effector delivery system	eptO	GspS family T2SS pilot lipoprotein variant EptO	100	5.11E-95	269
gene-etpL	etpL	Type II secretion pathway related protein	VFG040940 (gb|WP_071525076)	Etp (VF1178)	-	setA	Escherichia coli O157:H7 str. EDL933	Effector delivery system	gspL	type II secretion system protein GspL	100	2.29E-206	567
gene-etpM	etpM	Type II secretion pathway related protein	VFG040941 (gb|WP_001004187)	Etp (VF1178)	-	setA	Escherichia coli O157:H7 str. EDL933	Effector delivery system	Z_RS28445	type II secretion system protein M	100	1.98E-119	334
gene-etpC	etpC	Type II secretion pathway related protein	VFG040931 (gb|WP_001302175)	Etp (VF1178)	-	setA	Escherichia coli O157:H7 str. EDL933	Effector delivery system	gspC	type II secretion system protein GspC	100	2.79E-204	558
gene-etpF	etpF	Type II secretion	VFG040934 (gb|WP_00	Etp (VF1178)	-	setA	Escherichia coli	Effector delivery	gspF	type II secretion	100	7.34E-279	757

		pathway related protein	1173152)				O157:H7 str. EDL933	system		system inner membrane protein GspF			
gene-etpG	etpG	Type II secretion pathway related protein	VFG040935 (gb|WP_001231211)	Etp (VF1178)	-	setA	Escherichia coli O157:H7 str. EDL933	Effector delivery system	gspG	type II secretion system major pseudopilin GspG	100	1.17E-103	292
gene-etpE	etpE	Type II secretion pathway related protein	VFG040933 (gb|WP_000092338)	Etp (VF1178)	-	setA	Escherichia coli O157:H7 str. EDL933	Effector delivery system	gspE	type II secretion system ATPase GspE	100	0	959
ECs_5325	yjjQ	transcriptional regulator	VFG042668 (gb|WP_006485047)	Cable pilus (VF0700)	-	setB	Burkholderia cenocepacia J2315	Adherence	QU43_RS68240	response regulator transcription factor	49.1	7.46E-06	45.8
ECs_1148	torS	hybrid sensory histidine kinase in two-component	VFG015078 (gb|WP_012018243)	GacS/GacA two-component system (VF0908)	-	setA	Pseudomonas mendocina ymp	Regulation	gacS	response regulator	35.6	4.10E-53	200

		regulatory system with TorR											
ECs_3100	yojI	microcin J25 efflux ABC transporter permease/ATPase	VFG016024 (gb|WP_011267350)	Pyoverdine (VF0094)	Effective at acquiring iron from transferrin and lactoferrin; cytotoxic due to its ability to stimulating the production of reactive oxygen species	setA	Pseudomonas syringae pv. syringae B728a	Nutritional/Metabolic factor	pvdE	pyoverdine biosynthesis protein PvdE	33.8	3.97E-89	285
ECs_0015	dnaJ	chaperone Hsp40	VFG015291 (gb|WP_011268967)	P. syringae TTSS effectors (VF0911)	-	setB	Pseudomonas syringae pv. syringae B728a	Effector delivery system	hopI1	DnaJ domain-containing protein	49.2	1.31E-08	55.8
ECs_1155	cbpA	DNA-binding protein	VFG015291 (gb|WP_011268967)	P. syringae TTSS effectors (VF0911)	-	setB	Pseudomonas syringae pv. syringae B728a	Effector delivery system	hopI1	DnaJ domain-containing protein	41.8	3.68E-06	47.8
ECs_4741	xerC	tyrosine recombinase XerC	VFG000872 (gb|WP_000044711)	Type 1 fimbriae (VF0221)	Makes an important contribution to colonization of the bladder	setA	Escherichia coli CFT073	Adherence	fimE	Type 1 fimbriae Regulatory protein fimE	29.7	3.17E-17	78.2

ECs_5272	fimE	type 1 fimbriae regulatory protein FimE	VFG000872 (gb|WP_000044711)	Type 1 fimbriae (VF0221)	Makes an important contribution to colonization of the bladder	setA	Escherichia coli CFT073	Adherence	fimE	Type 1 fimbriae Regulatory protein fimE	99	3.45E-145	401
ECs_3766	xerD	tyrosine recombinase XerD	VFG042630 (gb|AAK82422)	Mannose-resistant fimbriae, mrf (VF1232)	-	setA	Photorhabdus luminescens str. K122	Adherence	mrfI	MrfI	32.9	3.57E-16	75.1
ECs_4405	yhjH	cyclic-di-GMP phosphodiesterase	VFG048341 (gb|WP_032694725)	Type 3 fimbriae (VF0567)	Mediating the formation of biofilms on biotic and abiotic surfaces	setA	Klebsiella oxytoca E718	Biofilm	mrkJ	phosphodiesterase	29.2	1.08E-26	103
ECs_3338	purC	phosphoribosylaminoimidazole-succinocarboxamide synthetase	VFG007789 (gb|WP_005064149)	Purine synthesis (VF0811)	-	setB	Mycobacterium abscessus subsp. bolletii 50594	Nutritional/Metabolic factor	purC	phosphoribosylaminoimidazolesuccinocarboxamide synthase	30.7	2.86E-14	70.5
ECs_2973	stx1B	Shiga toxin 1 subunit B	VFG000836 (gb|WP_000752026)	Stx (VF0206)	Important factors in disease pathogenesis and are responsible for the haemolytic uremic syndrome (HUS)	setA	Escherichia coli O157:H7 str. EDL933	Exotoxin	stx1B	shiga-like toxin 1 subunit B encoded	100	2.87E-60	178

										within prophage CP-933V			
ECs_1206	stx2B	Shiga toxin 2 subunit B	VFG043764 (gb|CAA90631)	SLTII (VF1315)	-	setA	Enterobacter cloacae str. 95MV2	Exotoxin	CAA90631	Shiga-like toxin II B subunit	98.9	1.00E-60	179
ECs_2974	stx1A	Shiga toxin 1 subunit A	VFG000835 (gb|WP_000691354)	Stx (VF0206)	Important factors in disease pathogenesis and are responsible for the haemolytic uremic syndrome (HUS)	setA	Escherichia coli O157:H7 str. EDL933	Exotoxin	stx1A	shiga-like toxin 1 subunit A encoded within prophage CP-933V	100	8.78E-220	600
ECs_1205	stx2A	Shiga toxin 2 subunit A	VFG043755 (gb|AAM88302)	Stx (VF0206)	Important factors in disease pathogenesis and are responsible for the haemolytic uremic syndrome (HUS)	setA	Escherichia coli O91:H21 str. B2F1	Exotoxin	stx2d1A	Shiga toxin 2 variant d A subunit	99.1	1.59E-224	612
ECs_2347	pdxH	pyridoxine 5'-phosphate oxidase	VFG015521 (gb|NP_252906)	Pyocyanin (VF0100)	Toxic to bacterial and eukaryotic cells due to the reactive oxygen intermediates it generates, such as superoxide radical and hydrogen peroxide	setA	Pseudomonas aeruginosa PAO1	Nutritional/Metabolic factor	phzG1	phenazine biosynthesis protein PhzG, pyridoxamine 5'-phosphate oxidase	33	3.93E-33	118
ECs_3058	psuK	pseudouri	VFG011809	LOS	LOS diversity is important for the	setA	Campylobac	Immune	rfaE1	D-glycero-	21.2	3.62E-	51.2

		dine kinase	(gb|WP_011732197)	(VF0326)	ability to colonize a wide variety of hosts and intestinal niches; the ability to generate variation at high frequency, the molecular mimicry evident in LOS structure support a role in the avoidance of host defences; the similarity of LOS structures to host gangliosides and the subsequent ability to generate crossreacting antibodies forms the pathological basis for the association of preceding C. jejuni infection with Guillain-Barre syndrome		ter fetus subsp. fetus 82-40	modulation		beta-D-manno-heptose-7-phosphate kinase		07	
ECs_3052	yeiI	carbohydrate kinase	VFG011809 (gb|WP_011732197)	LOS (VF0326)	LOS diversity is important for the ability to colonize a wide variety of hosts and intestinal niches; the ability to generate variation at high frequency, the molecular mimicry evident in LOS structure support a role in the avoidance of host defences; the similarity of LOS structures to host gangliosides and the subsequent ability to generate crossreacting antibodies forms the pathological basis for the association of preceding C. jejuni infection with	setB	Campylobacter fetus subsp. fetus 82-40	Immune modulation	rfaE1	D-glycero-beta-D-manno-heptose-7-phosphate kinase	23.9	8.46E-06	47.4

					Guillain-Barre syndrome								
ECs_3935	hldE	heptose 7-phosphate kinase and heptose 1-phosphate adenyltransferase	VFG013431 (gb|WP_005656815)	LOS (VF0044)	Major immunogen; LOS phosphorylcholine (ChoP) may influence invasion via interaction with PAF receptor and stimulates of inflammatory signals; LPS phase variation is characterized by the spontaneous loss and gain of oligosaccharide structures present in the outer core. the phase variable expression of LPS biosynthesis genes promotes evasion of antigen-specific host immune defences and allow colonization of different host microenvironments	setA	Haemophilus influenzae 86-028NP	Immune modulation	rfaE	ADP-heptose synthase	70.4	9.35E-236	654
ECs_3746	hyuA	D-stereospecific phenylhydantoinase	VFG049120 (gb|WP_012737535)	Allantion utilization (VF0572)	Providing a nitrogen source to increase virulence in K. pneumoniae at certain sites of infection	setA	Klebsiella pneumoniae subsp. pneumoniae NTUH-K2044	Nutritional/Metabolic factor	allB	allantoinase	29	2.13E-47	169
ECs_0548	ECs_0548	adhesin	VFG042350 (gb|WP_041786631)	Hemagglutinin (Hag)/M.catarrhalis IgD binding	-	setB	Moraxella catarrhalis RH4	Effector delivery system	MCR_RS02830	YadA-like family protein	40.4	2.86E-08	55.5

				protein (MID) (VF1281)									
ECs_4569	escV	T3SS structure protein EscV	VFG000813 (gb|WP_001037814)	TTSS (VF0191)	Injects Tir and other effector molecules directly into the host cell. Effector molecules activate cell-signaling pathways, causing alterations in the host cell cytoskeleton and resulting in the depolymerization of actin and the loss of microvilli	setA	Escherichia coli O157:H7 str. EDL933	Effector delivery system	escV	Type III secretion system major export apparatus protein	100	0	1199
ECs_4568	escN	T3SS structure protein EscN	VFG000812 (gb|WP_000622545)	TTSS (VF0191)	Injects Tir and other effector molecules directly into the host cell. Effector molecules activate cell-signaling pathways, causing alterations in the host cell cytoskeleton and resulting in the depolymerization of actin and the loss of microvilli	setA	Escherichia coli O157:H7 str. EDL933	Effector delivery system	escN	ATPase EscN	100	2.95001656475e-319	862
ECs_3708	ygeG	SycD-like chaperone family TPR-repeat-containing protein	VFG042096 (gb|NP_311735)	ETT2 (VF1161)	-	setA	Escherichia coli O157:H7 str. Sakai	Effector delivery system	ygeG	SycD-like chaperone family TPR-repeat-containing protein	100	9.96E-121	337

ECs_4653	espY4	T3SS effector-like protein EspY	VFG034915 (gb|WP_000225947)	TTSS secreted effectors (VF1110)	Cif (Deamidase. Induces cytopathic effects of actin stress fiber formation and cell cycle arrest.); EspB (Pore formation, actin disruption, microvilli effacement, anti-phagocytosis.); EspF (Inducing degradation of the aniapoptic protein AbcF2, tight junction disruption, microvilli effacement and elongation, mitochondrial dysfunction, N-WASP activation, SGLT-1 inactivation, pedestal maturation, inhibition of NHE3 activity, membrane remodelling; targets and disrupts the nucleolus late in infection, which is temporally controlled by host mitochondria.); EspFu/tccP (Inducing degradation of the aniapoptic protein AbcF2, tight junction disruption, microvilli effacement and elongation, mitochondrial dysfunction, N-WASP activation, SGLT-1 inactivation, pedestal maturation, inhibition of NHE3 activity, membrane remodelling; targets and disrupts the nucleolus late in infection, which is temporally	setA	Escherichia coli O157:H7 str. EDL933	Effector delivery system	espY4	Type III secretion system effector EspY4	100	0	1517

controlled by host mitochondria.); EspG (TBC-like GTPase activating protein. Efficiently catalyzes GTP hydrolysis in Rab1 to disrupt of Rab1-mediated ER-to-Golgi trafficking.); EspH (First bacterial effector acting directly on RhoGEFs, EspH directly binds to the DH-PH domain in RhoGEFs to disrupt RhoGEF-Rho signaling; critical for inhibiting macrophage phagocytosis.); EspJ (Inhibit both IgG- and complement receptor-mediated phagocytosis.); EspK; EspL1; EspL2 (Cysteine protease. Bounds F-actin-aggregating annexin 2 directly to increase annexin 2's ability to aggregate Tir-induced F-actin; block necroptosis and in flammation.); EspL4; EspM1 (GEF. Activates the RhoA signaling pathway and induce the formation of stress fibres; inhibit pedestal formation and induce tight junction mislocalization.); EspM2 (GEF. Activates the RhoA signaling pathway and induce the formation of stress

fibres; inhibit pedestal formation and induce tight junction mislocalization.); EspN; EspO1-1; EspO1-2; EspR1; EspR3; EspR4; EspT (GEF. Activates Rac1 and Cdc42 leading to formation of membrane ruffles and lamellipodia; induces membrane ruffles to facilitate bacterial invasion into non-phagocytic cells in a process involving Rac1 and Wave2.); EspW; EspX1; EspX2; EspX4; EspX5; EspX6; EspX7/nleL (E3 ubiquitin ligase, HECT-like. Modulates pedestal formation.); EspY1; EspY2; EspY3; EspY4; EspY5; Map (GEF. Mimics the host Dbl and catalyses the exchange of GDP for GTP in Cdc42, involved in effacement, SGLT1 inhibition, formation of filopodia and disruption of mitochondrial function.); NleA/espI (Disruption of tight junctions by inhibition of host cell protein trafficking through COPII-dependent pathways.); NleB1 (Blocks translocation of the p65 and to the host cell nucleus to inhibit

NF-<kappa>B pathway, but NleE and NleB act at different points in the NF-<kappa>B signaling pathway.); NleB2 (May also have anti-inflammatory activity.); NleC (Metalloprotease. Zn-dependent endopeptidases that specifically clip and inactivate RelA (p65), thus blocking NF-<kappa>B pathway.); NleD (Metalloprotease. Zn-dependent endopeptidases that specifically clip and inactivate JNK and p38, thus blocking AP-1 pathway.); NleE (PMN tran-epithelial migration; blocks translocation of the p65 to the host cell nucleus by preventing I<kappa>B degradation to inhibit NF-<kappa>B pathway.); NleF; NleG-1; NleG2-2; NleG2-3; NleG2-4; NleG5-1; NleG5-2; NleG6-1; NleG6-2; NleG6-3; NleG7 (U-box type E3 ubiquitin ligases.); NleG8-2; NleH1 (Ser/Thr protein kinase. Binds directly to a subunit of NF-<kappa>B, the ribosomal protein S3 (RPS3), reducing the nuclear abundance of RPS3 to dampen host transcriptional outputs;

					interact with Bax inhibitor-1 to block apoptosis.); NleH2 (Putative kinase. Attenuates NF-<kappa>B pathway.); SepZ/espZ (EspZ interacts with CD98 in host cell membranes to promote host cell survival, therefore provide the pathogen with valuable time to colonize efficiently prior to dissemination.); TccP2; Tir (Mimics host immunoreceptor tyrosine-based inhibition motifs (ITIMs), also see helicobacter CagA. EHEC Tir lacks the Nck binding site. Conserved NPY (Asn-Pro-Tyr) motif recruits the adaptor protein IRTKS and/or IRSp53. IRTKS/IRSp53 link Tir and TccP/EspFu, which in turn activates N-WASP; Receptor for intimin; effacement; SGLT1 inhibition; recruits SHIP2 to control actin-pedestal morphology; maintains the integrity of the epithelium by keeping the destructive activity of EspG and EspG2 in check.)								
ECs_5346	serB	3-phosphoserine phosphata	VFG043287 (gb|WP_003425253)	Peritrichous flagella	-	setA	Clostridium difficile 630	Motility	CD630_RS01730	HAD-IB family phosphatas	23.8	1.61E-08	53.9

		se SerB		(VF0732)						e			
ECs_5345	lplA	lipoate-protein ligase A	VFG006776 (gb|WP_003771179)	LplA1 (VF0347)	Necessary for efficient intracellular proliferation of L. monocytogenes	setA	Listeria innocua Clip11262	Nutritional/Metabolic factor	lplA1	lipoate protein ligase	34.2	6.53E-49	172
ECs_3717	eprJ	type III secretion protein EprJ	VFG042089 (gb|NP_311744)	ETT2 (VF1161)	-	setA	Escherichia coli O157:H7 str. Sakai	Effector delivery system	eprJ	type III secretion protein EprJ	100	2.03E-72	210
ECs_4203	yheS	ABC-F family regulatory ATPase	VFG026222 (gb|WP_012327958)	Capsule I (VF0436)	A key virulence determinant and that loss of capsule production results in severe attenuation in animal models of disease	setA	Burkholderia cenocepacia MCO-3	Immune modulation	wzt2	ATP-binding ABC transporter capsular polysaccharide export protein	33	4.86E-17	80.5
ECs_3478	recN	recombination and repair protein RecN	VFG037126 (gb|WP_014575255)	RecN (VF0457)	Recombinational repair protein that protects against ROS and non-oxidative killing by neutrophils	setA	Neisseria meningitidis alpha710	Stress survival	recN	DNA repair protein RecN	38.3	2.39E-108	335
ECs_3716	eprK	type III secretion system lipoprotein precursor	VFG042092 (gb|NP_311743)	ETT2 (VF1161)	-	setA	Escherichia coli O157:H7 str. Sakai	Effector delivery system	eprK	type III secretion system lipoprotein precursor EprK	100	3.48E-170	468

		EprK											
ECs_1715	hemA	glutamyl tRNA reductase	VFG013196 (gb|WP_012340438)	Heme biosynthesis (VF0758)	-	setB	Haemophilus somnus 2336	Nutritional/Metabolic factor	hemA	glutamyl-tRNA reductase	48	1.09E-127	375
ECs_0041	caiB	crotonobetainyl CoA:carnitine CoA transferase	VFG044392 (gb|AAF33133)	Pyridine-2,6-dithiocarboxylic acid (PDTC) (VF0938)	-	setB	Pseudomonas stutzeri KC	Nutritional/Metabolic factor	pdtorfI	putative racemase	31.1	4.18E-07	52
ECs_3254	frc	formyl-CoA transferase	VFG044392 (gb|AAF33133)	Pyridine-2,6-dithiocarboxylic acid (PDTC) (VF0938)	-	setB	Pseudomonas stutzeri KC	Nutritional/Metabolic factor	pdtorfI	putative racemase	31	1.12E-16	82
ECs_0446	araJ	L-arabinose-inducible transporter	VFG002097 (gb|WP_010946287)	Pht (VF0337)	Enables transmissive L. pneumophila to assess their phagosomal nutrient supply before reentering the cell cycle	setA	Legionella pneumophila subsp. pneumophila str. Philadelphia 1	Nutritional/Metabolic factor	phtA	major facilitator family transporter, phagosomal transporter	26.4	2.85E-08	55.5
ECs_4005	garP	(D)-galac	VFG002097	Pht	Enables transmissive L. pneumophila to	setA	Legionella	Nutritiona	phtA	major	20.1	7.30E-	54.3

		tarate transporter	(gb|WP_010946287)	(VF0337)	assess their phagosomal nutrient supply before reentering the cell cycle		pneumophila subsp. pneumophila str. Philadelphia 1	l/Metabolic factor		facilitator family transporter, phagosomal transporter		08	
ECs_3649	gudP	D-glucarate transporter	VFG002097 (gb|WP_010946287)	Pht (VF0337)	Enables transmissive L. pneumophila to assess their phagosomal nutrient supply before reentering the cell cycle	setA	Legionella pneumophila subsp. pneumophila str. Philadelphia 1	Nutritional/Metabolic factor	phtA	major facilitator family transporter, phagosomal transporter	22.8	8.85E-15	75.9
ECs_4030	diaA	DnaA initiator-associating factor for replication initiation	VFG011801 (gb|WP_002850280)	LOS (VF0326)	LOS diversity is important for the ability to colonize a wide variety of hosts and intestinal niches; the ability to generate variation at high frequency, the molecular mimicry evident in LOS structure support a role in the avoidance of host defences; the similarity of LOS structures to host gangliosides and the subsequent ability to generate crossreacting antibodies forms the pathological	setA	Campylobacter fetus subsp. fetus 82-40	Immune modulation	gmhA	D-sedoheptulose 7-phosphate isomerase	44.8	9.98E-45	146

					basis for the association of preceding C. jejuni infection with Guillain-Barre syndrome								
ECs_4582	escS	T3SS structure protein EscS	VFG000826 (gb|WP_000379425)	TTSS (VF0191)	Injects Tir and other effector molecules directly into the host cell. Effector molecules activate cell-signaling pathways, causing alterations in the host cell cytoskeleton and resulting in the depolymerization of actin and the loss of microvilli	setA	Escherichia coli O157:H7 str. EDL933	Effector delivery system	escS	Type III secretion systemminor export apparatus protein EscS	100	4.60E-57	170
ECs_4581	escT	T3SS structure protein EscT	VFG000825 (gb|WP_001002833)	TTSS (VF0191)	Injects Tir and other effector molecules directly into the host cell. Effector molecules activate cell-signaling pathways, causing alterations in the host cell cytoskeleton and resulting in the depolymerization of actin and the loss of microvilli	setA	Escherichia coli O157:H7 str. EDL933	Effector delivery system	escT	Type III secretion system minor export apparatus protein EscT	100	9.12E-173	476
ECs_4580	escU	T3SS structure protein EscU	VFG000824 (gb|WP_001291694)	TTSS (VF0191)	Injects Tir and other effector molecules directly into the host cell. Effector molecules activate cell-signaling pathways, causing alterations in the host cell cytoskeleton and resulting in the depolymerization of actin and the loss	setA	Escherichia coli O157:H7 str. EDL933	Effector delivery system	escU	Type III secretion system export apparatus switch protein	100	2.67E-236	644

					of microvilli					EscU			
ECs_4587	ECs_4587	T3SS component	VFG000831 (gb|WP_000628731)	TTSS (VF0191)	Injects Tir and other effector molecules directly into the host cell. Effector molecules activate cell-signaling pathways, causing alterations in the host cell cytoskeleton and resulting in the depolymerization of actin and the loss of microvilli	setA	Escherichia coli O157:H7 str. EDL933	Effector delivery system	escE	chaperone for EscF	100	9.05E-45	137
ECs_4586	ECs_4586	T3SS component	VFG000830 (gb|WP_001301632)	TTSS (VF0191)	Injects Tir and other effector molecules directly into the host cell. Effector molecules activate cell-signaling pathways, causing alterations in the host cell cytoskeleton and resulting in the depolymerization of actin and the loss of microvilli	setA	Escherichia coli O157:H7 str. EDL933	Effector delivery system	cesAB	chaperone for EspA and EspB	99	8.85E-62	183
ECs_4585	ECs_4585	T3SS component	VFG000829 (gb|WP_000151812)	TTSS (VF0191)	Injects Tir and other effector molecules directly into the host cell. Effector molecules activate cell-signaling pathways, causing alterations in the host cell cytoskeleton and resulting in the depolymerization of actin and the loss of microvilli	setA	Escherichia coli O157:H7 str. EDL933	Effector delivery system	escK	type III secretion system sorting platform component	100	2.33E-148	409
ECs_4584	ECs_4584	T3SS	VFG000828	TTSS	Injects Tir and other effector	setA	Escherichi	Effector	escL	negative	100	3.62E-	403

		component	(gb|WP_000780681)	(VF0191)	molecules directly into the host cell. Effector molecules activate cell-signaling pathways, causing alterations in the host cell cytoskeleton and resulting in the depolymerization of actin and the loss of microvilli		a coli O157:H7 str. EDL933	delivery system		regulator EscL, stator protein		145	
ECs_4588	ler	transcription regulator Ler	VFG000832 (gb|WP_001055728)	Ler (VF0192)	Activates the LEE2, LEE3, tir, and orf19 promoters; required for LEE4 expression; not activates the LEE1 promoter; controls genes located outside the LEE, such as espC and genes encoding several morphologically distinct types of fimbriae	setA	Escherichia coli O157:H7 str. EDL933	Regulation	ler	transcription regulator Ler	100	1.15E-84	242
ECs_4342	acpT	4'-phosphopantetheinyl transferase	VFG044305 (gb|WP_000071884)	Yersiniabactin-related (VF1251)	-	setA	Proteus mirabilis HI4320	Nutritional/Metabolic factor	PMI_RS12865	4'-phosphopantetheinyl transferase superfamily protein	33.9	7.21E-10	56.6
ECs_5274	fimI	fimbrial protein involved in type 1 pilus	VFG012286 (gb|NP_313301)	Type 1 fimbriae (VF0221)	Makes an important contribution to colonization of the bladder	setA	Escherichia coli O157:H7 str. Sakai	Adherence	fimI	Fimbrin-like protein fimI precursor	100	2.44E-122	341

		biosynthesis											
ECs_1669	minD	cell division inhibitor MinD	VFG011850 (gb|YP_002343527)	Flagella (VF0114)	Penetrating the mucus barrier; chemotaxis is also important for intestinal colonization; In the absence of a type III secretion system, the flagellar secretion apparatus appears to secrete several putative virulence proteins including FspA, FlaC and up to eight Cia proteins	setA	Campylobacter jejuni subsp. jejuni NCTC 11168	Motility	flhG	ATP-binding protein	35.5	5.15E-19	84.3
ECs_4299	ugpB	sn-glycerol-3-phosphate ABC transporter periplasmic binding protein	VFG043534 (gb|WP_004681934)	SP41 (VF0694)	-	setB	Brucella melitensis bv. 1 str. 16M	Adherence	ugpB	sn-glycerol-3-phosphate ABC transporter substrate-binding protein UgpB	47.3	2.34E-137	400
ECs_4293	ggt	gamma-glutamyltranspeptidase	VFG047605 (gb|WP_080558930)	GGT (VF0553)	Cleavage of cysteine containing peptides (glutathione and <gamma>-glutamyl-cysteine peptides) by GGT activity thus provides the essential source of cysteine required for intracellular multiplication	setA	Francisella cf. novicida Fx1	Nutritional/Metabolic factor	ggt	gamma-glutamyltranspeptidase	52.4	2.16E-186	537
ECs_1663	ompT	outer	VFG012928	IcsP	Involved in the cleavage of IcsA, may	setA	Shigella	Exoenzyme	icsP/sopA	outer	57.4	4.51E-	378

		membrane protease VII	(gb|WP_005005342)	(SopA) (VF0122)	contribute to the polar distribution of IcsA on the lateral side of the bacterial surface		boydii Sb227			membrane protease of the OmpP family, involved in cleavage of surface exposed IcsA		132	
ECs_1662	ECs_1662	hydrolase	VFG030164 (gb|YP_001702655)	Carboxylesterase (VF0839)	-	setB	Mycobacterium abscessus ATCC 19977	Effector delivery system	caeA	Conserved hypothetical protein (possible hydrolase)	32.5	2.65E-64	216
ECs_1396	ECs_1396	AidA-I family adhesin	VFG012454 (gb|WP_000820483)	Cah, AIDA-I type (VF1129)	-	setA	Escherichia coli O157:H7 str. EDL933	Biofilm	cah	calcium-binding autotransporter Cah	100	0	1412
ECs_5279	fimH	minor component of type 1 fimbriae	VFG012320 (gb|WP_000832221)	Type 1 fimbriae (VF0221)	Makes an important contribution to colonization of the bladder	setA	Escherichia coli O157:H7 str. EDL933	Adherence	fimH	FimH protein precursor	100	2.52E-216	590
ECs_0139	yadC	fimbrial protein	VFG004221 (gb|WP_000924983)	Sta (VF0953)	-	setB	Salmonella enterica subsp. enterica	Adherence	staG	fimbrial-like adhesin	26.3	4.79E-09	57.4

							serovar Typhi str. CT18						
ECs_0137	panC	pantothenate synthetase	VFG009302 (gb|WP_011892321)	PanC/PanD (VF0319)	Lipid biosynthesis and metabolism play a pivotal role in the intracellular replication and persistence of M. tuberculosis. Pantothenic acid (vitamin B5) is an essential molecule required for the synthesis of coenzyme A and acyl carrier protein (ACP). PanC (pantothenate synthetase) and PanD (aspartate-1-decarboxylase) involved in pantothenate biosynthesis.	setA	Mycobacterium gilvum PYR-GCK	Nutritional/Metabolic factor	panC	pantoate--beta-alanine ligase	48.6	1.61E-72	225
ECs_0134	yadE	polysaccharide deacetylase lipoprotein	VFG011777 (gb|WP_002850271)	LOS (VF0326)	LOS diversity is important for the ability to colonize a wide variety of hosts and intestinal niches; the ability to generate variation at high frequency, the molecular mimicry evident in LOS structure support a role in the avoidance of host defences; the similarity of LOS structures to host gangliosides and the subsequent ability to generate crossreacting antibodies forms the pathological basis for the association of preceding C. jejuni infection with	setA	Campylobacter fetus subsp. fetus 82-40	Immune modulation	CFF8240_RS06855	polysaccharide deacetylase family protein	29.9	9.88E-26	104

					Guillain-Barre syndrome								
ECs_0135	panD	aspartate 1-decarboxylase	VFG022623 (gb|WP_013830813)	PanC/PanD (VF0319)	Lipid biosynthesis and metabolism play a pivotal role in the intracellular replication and persistence of M. tuberculosis. Pantothenic acid (vitamin B5) is an essential molecule required for the synthesis of coenzyme A and acyl carrier protein (ACP). PanC (pantothenate synthetase) and PanD (aspartate-1-decarboxylase) involved in pantothenate biosynthesis.	setA	Mycobacterium sp. JDM601	Nutritional/Metabolic factor	panD	aspartate 1-decarboxylase	51.8	1.45E-38	126
ECs_4900	birA	bifunctional protein BirA	VFG047443 (gb|WP_012281080)	Biotin synthesis (VF0552)	Critical for rapid phagosomal escape. Sequestration of biotin could restrict Francisella to the phagosome, blocking their escape and preventing them from reaching their replicative niche in the cytoplasm	setA	Francisella philomiragia subsp. philomiragia ATCC 25017	Nutritional/Metabolic factor	birA	biotin--[acetyl-CoA-carboxylase] ligase	29.4	1.09E-46	160
ECs_3661	fucP	L-fucose transporter	VFG000313 (gb|WP_001174195)	LPS (VF0056)	H. pylori LPS have much lower immunobiological activities than enterobacterial LPS, thus may prolong H. pylori infection for longer; mediates a lectin-like interaction with laminin, the binding may disrupt epithelial cell-basement membrane interactions contributing to the	setA	Helicobacter pylori 26695	Immune modulation	gluP	glucose/galactose transporter	35.4	3.90E-65	214

					disruption of gastric mucosal integrity and the development of gastric leakiness associated with the bacterium								
ECs_0628	fepG	iron-enterobactin ABC transporter permease	VFG013038 (gb|WP_000640963)	Enterobactin synthesis and transport (VF0984)	-	setA	Shigella sonnei Ss046	Nutritional/Metabolic factor	fepG	iron-enterobactin ABC transporter permease	99.7	1.69E-220	603
ECs_0629	fepD	ferrienterobactin ABC transporter permease	VFG013029 (gb|WP_001298899)	Enterobactin synthesis and transport (VF0984)	-	setA	Shigella flexneri 2a str. 2457T	Nutritional/Metabolic factor	fepD	Fe(3+)-siderophore ABC transporter permease	100	4.44E-218	597
ECs_0622	entD	4'-phosphopantetheinyl transferase entD	VFG013004 (gb|NP_706438)	Enterobactin synthesis and transport (VF0984)	-	setA	Shigella flexneri 2a str. 301	Nutritional/Metabolic factor	entD	enterobactin synthase multienzyme complex phosphopantetheinyltransferase	100	7.89E-155	428
ECs_0623	fepA	ferrienterobactin outer	VFG013009 (gb|WP_001034900)	Enterobactin synthesis	-	setA	Shigella dysenteriae Sd197	Nutritional/Metabolic factor	fepA	siderophore enterobact	99.6	0	1513

		membrane transporter		and transport (VF0984)						in receptor FepA			
ECs_0624	fes	enterobactin/ferri enterobactin esterase	VFG044159 (gb|WP_000125846)	Enterobactin (VF0228)	Iron uptake: the siderophore enterobactin imported through the FepA receptor and the FepBCDG system	setA	Escherichia coli CFT073	Nutritional/Metabolic factor	fes	enterobactin/ferric enterobactin esterase	95.8	3.82E-307	828
ECs_0625	entF	enterobactin synthase multienzyme complex component	VFG013002 (gb|WP_000077683)	Enterobactin synthesis and transport (VF0984)	-	setA	Shigella sonnei Ss046	Nutritional/Metabolic factor	entF	enterobactin non-ribosomal peptide synthetase EntF	98	0	2513
ECs_3464	aroF	3-deoxy-D-arabino-heptulosonate-7-phosphate synthase	VFG044185 (gb|WP_011152542)	Vibriobactin/Vulnibactin (VF0626)	-	setB	Vibrio vulnificus YJ016	Nutritional/Metabolic factor	BJE04_RS21750	3-deoxy-7-phosphoheptulonate synthase	43.8	2.15E-97	293
ECs_2411	aroH	3-deoxy-D-arabino-heptulosonate-7-phosphate	VFG044185 (gb|WP_011152542)	Vibriobactin/Vulnibactin (VF0626)	-	setB	Vibrio vulnificus YJ016	Nutritional/Metabolic factor	BJE04_RS21750	3-deoxy-7-phosphoheptulonate synthase	55.6	1.03E-116	342

		synthase											
ECs_0782	aroG	3-deoxy-D-arabino-heptulosonate-7-phosphate synthase	VFG044185 (gb|WP_011152542)	Vibriobactin/Vulnibactin (VF0626)	-	setB	Vibrio vulnificus YJ016	Nutritional/Metabolic factor	BJE04_RS21750	3-deoxy-7-phosphoheptulonate synthase	50.2	3.29E-108	320
ECs_3025	mdtQ	channel/filament proteins	VFG037739 (gb|WP_038346720)	AdeFGH efflux pump (VF0504)	Play a potential role in the synthesis and transport of autoinducer molecules during biofilm formation	setA	Acinetobacter baumannii BJAB0715	Biofilm	adeH	outer membrane protein	24.9	1.27E-24	106
ECs_3027	ECs_3027	salicylate hydroxylase	VFG000173 (gb|NP_252907)	Pyocyanin (VF0100)	Toxic to bacterial and eukaryotic cells due to the reactive oxygen intermediates it generates, such as superoxide radical and hydrogen peroxide	setA	Pseudomonas aeruginosa PAO1	Nutritional/Metabolic factor	phzS	flavin dependent hydroxylase PhzS	30.5	1.33E-25	107
ECs_5100	melR	melibiose operon transcriptional regulator	VFG000208 (gb|NP_250404)	TTSS (VF0083)	Transports four known effector proteins: ExoS, ExoT, ExoU and ExoY, although most strains generally do not express all four at the same time	setA	Pseudomonas aeruginosa PAO1	Effector delivery system	exsA	type III secretion system regulatory protein ExsA	27.1	2.20E-06	48.1
ECs_4837	cpxA	two-component system sensor	VFG038235 (gb|YP_001083791)	BfmRS (VF0463)	-	setA	Acinetobacter baumannii ATCC 17978	Regulation	bfmS	signal transduction histidine	30.1	4.33E-34	133

		histidine kinase CpxA								kinase			
ECs_4246	envZ	two-component system sensor histidine kinase EnvZ	VFG038235 (gb|YP_001083791)	BfmRS (VF0463)	-	setA	Acinetobacter baumannii ATCC 17978	Regulation	bfmS	signal transduction histidine kinase	31.6	2.31E-32	128
ECs_2315	rstB	two-component system sensor histidine kinase RstB	VFG038235 (gb|YP_001083791)	BfmRS (VF0463)	-	setA	Acinetobacter baumannii ATCC 17978	Regulation	bfmS	signal transduction histidine kinase	32.2	8.96E-49	172
ECs_0829	ECs_0829	phage protease/scaffold protein	VFG000077 (gb|NP_465991)	ClpP (VF0074)	Serine protease involved in proteolysis and is required for growth under stress conditions	setA	Listeria monocytogenes EGD-e	Stress survival	clpP	ATP-dependent Clp protease proteolytic subunit	30.7	8.13E-10	58.9
ECs_0491	clpP	ATP-dependent Clp protease proteolyt	VFG000077 (gb|NP_465991)	ClpP (VF0074)	Serine protease involved in proteolysis and is required for growth under stress conditions	setA	Listeria monocytogenes EGD-e	Stress survival	clpP	ATP-dependent Clp protease proteolyti	66.5	2.97E-91	265

		ic subunit								c subunit			
ECs_4834	sodA	Fe-Mn family superoxide dismutase	VFG001867 (gb|WP_016357051)	SodB (VF0169)	A cytoplasmic iron superoxide dismutase; important for intracellular survival and transmission	setA	Legionella pneumophila subsp. pneumophila str. Philadelphia 1	Stress survival	sodB	superoxide dismutase	43.8	8.33E-53	167
ECs_2365	sodB	Fe-Mn family superoxide dismutase	VFG001867 (gb|WP_016357051)	SodB (VF0169)	A cytoplasmic iron superoxide dismutase; important for intracellular survival and transmission	setA	Legionella pneumophila subsp. pneumophila str. Philadelphia 1	Stress survival	sodB	superoxide dismutase	67.5	4.89E-99	284
ECs_3262	ypdC	DNA-binding protein	VFG024949 (gb|WP_011204592)	Bsa T3SS (VF0428)	Delivering effector proteins into host cells to manipulate host cell functions	setA	Burkholderia mallei ATCC 23344	Effector delivery system	bprC	AraC family transcriptional regulator, regulates basal T6SS1 expression	25.3	1.74E-08	54.7
ECs_4883	yijO	AraC family transcrip	VFG024949 (gb|WP_011204592)	Bsa T3SS (VF0428)	Delivering effector proteins into host cells to manipulate host cell functions	setA	Burkholderia mallei ATCC 23344	Effector delivery system	bprC	AraC family transcriptional	33.3	7.57E-07	49.7

		tional activator								regulator, regulates basal T6SS1 expression			
ECs_0343	rclR	reactive chlorine species-specific activator of the rcl genes	VFG024949 (gb|WP_011204592)	Bsa T3SS (VF0428)	Delivering effector proteins into host cells to manipulate host cell functions	setA	Burkholderia mallei ATCC 23344	Effector delivery system	bprC	AraC family transcriptional regulator, regulates basal T6SS1 expression	34	5.81E-06	47
ECs_2370	cfa	cyclopropane fatty acyl phospholipid synthase	VFG009071 (gb|WP_015289097)	Mycolic acid trans-cyclopropane synthetase (VF0802)	-	setA	Mycobacterium canettii CIPT 140070010	Others	cmaA2	class I SAM-dependent methyltransferase	34.7	4.07E-43	152
ECs_2574	yebC	hypothetical protein	VFG039536 (gb|NP_820549)	T4SS secreted effectors (VF0696)	CBUA0020; CBU_0012*; CBU_0113; CBU_0122; CBU_0183; CBU_0201; CBU_0270; CBU_0295; CBU_0344*; CBU_0372; CBU_0375*; CBU_0469; CBU_0513; CBU_0534; CBU_0590; CBU_0635; CBU_0637; CBU_0820*; CBU_1048*; CBU_1079; CBU_1107*; CBU_1150*; CBU_1198; CBU_1268;	setA	Coxiella burnetii RSA 493	Effector delivery system	CBU_1566	Coxiella Dot/Icm type IVB secretion system translocated effector	59.3	9.54E-99	288

CBU_1349; CBU_1370; CBU_1409;
CBU_1434; CBU_1493; CBU_1495*;
CBU_1525*; CBU_1530; CBU_1566;
CBU_1576; CBU_1594; CBU_1607;
CBU_1614; CBU_1639; CBU_1665;
CBU_1677; CBU_1685; CBU_1752;
CBU_1754; CBU_1789; CBU_1790;
CBU_1794; CBU_1818; CBU_1819;
CBU_1863; CBU_2016; CBU_2028;
CBU_2056; CBU_2059*; CBU_2076; AnkA;
AnkB; AnkF; AnkG (Interacts with host
protein p32 to block apoptosis.);
AnkH; AnkI; AnkM/cig58; AnkP; Cem1;
Cem12; Cem13; Cem3; Cem4; Cem6; Cem9;
CetCb1; CetCb2; CetCb3; CetCB4;
CetCb5; CetCb6; CirA/coxCC1
(Phosphate transporter family
protein.); CirB; CirC/coxDFB1;
CoxCC10/cig49; CoxCC11; CoxCC12;
CoxCC14; CoxCC15; CoxCC3; CoxCC4;
CoxCC5; CoxCC6; CoxCC7/cig44; CoxCC8;
CoxDFB3; CoxDFB4 (Surface antigen.);
CoxDFB5/cig57; CoxDFB6; CoxFIC1;
CoxH2/rimL (Acetyltransferase.);
CoxH3; CoxH4/cig61; CoxK1 (Protein
kinase, putative.); CoxK2; CoxTPR1

ECs_2780	yeeN	hypothetical protein	VFG039536 (gb|NP_820549)	T4SS secreted effectors (VF0696)	(Conserved domain protein.); CoxU1; CoxU2; CpeA; CpeB; CpeC/coxU3 (Hypothetical protein plasmid QpH1.); CpeD; CpeE; CpeF; CpeG; CpeH; CvpA; MceA; PhnB; CBUD_RS05145; CBUD_RS06720*; CBUD_RS08635; CBUD_RS11275; CBUD_RS12405; CBUG_RS02435; CBUK_RS06760 CBUA0020; CBU_0012*; CBU_0113; CBU_0122; CBU_0183; CBU_0201; CBU_0270; CBU_0295; CBU_0344*; CBU_0372; CBU_0375*; CBU_0469; CBU_0513; CBU_0534; CBU_0590; CBU_0635; CBU_0637; CBU_0820*; CBU_1048*; CBU_1079; CBU_1107*; CBU_1150*; CBU_1198; CBU_1268; CBU_1349; CBU_1370; CBU_1409; CBU_1434; CBU_1493; CBU_1495*; CBU_1525*; CBU_1530; CBU_1566; CBU_1576; CBU_1594; CBU_1607; CBU_1614; CBU_1639; CBU_1665; CBU_1677; CBU_1685; CBU_1752; CBU_1754; CBU_1789; CBU_1790; CBU_1794; CBU_1818; CBU_1819; CBU_1863; CBU_2016; CBU_2028; CBU_2056; CBU_2059*; CBU_2076; AnkA;	setA	Coxiella burnetii RSA 493	Effector delivery system	CBU_1566	Coxiella Dot/Icm type IVB secretion system translocated effector	41	6.42E-42	142

					AnkB; AnkF; AnkG (Interacts with host protein p32 to block apoptosis.); AnkH; AnkI; AnkM/cig58; AnkP; Cem1; Cem12; Cem13; Cem3; Cem4; Cem6; Cem9; CetCb1; CetCb2; CetCb3; CetCB4; CetCb5; CetCb6; CirA/coxCC1 (Phosphate transporter family protein.); CirB; CirC/coxDFB1; CoxCC10/cig49; CoxCC11; CoxCC12; CoxCC14; CoxCC15; CoxCC3; CoxCC4; CoxCC5; CoxCC6; CoxCC7/cig44; CoxCC8; CoxDFB3; CoxDFB4 (Surface antigen.); CoxDFB5/cig57; CoxDFB6; CoxFIC1; CoxH2/rimL (Acetyltransferase.); CoxH3; CoxH4/cig61; CoxK1 (Protein kinase, putative.); CoxK2; CoxTPR1 (Conserved domain protein.); CoxU1; CoxU2; CpeA; CpeB; CpeC/coxU3 (Hypothetical protein plasmid QpH1.); CpeD; CpeE; CpeF; CpeG; CpeH; CvpA; MceA; PhnB; CBUD_RS05145; CBUD_RS06720*; CBUD_RS08635; CBUD_RS11275; CBUD_RS12405; CBUG_RS02435; CBUK_RS06760								
ECs_2577	yecD	isochorismatase	VFG044368 (gb|WP_01	Chrysobactin	-	setA	Dickeya dadantii	Nutritional/Metaboli	DDA3937_RS14715	isochorismatase	29.2	6.57E-14	68.2

		family protein	3318792)	(VF1242)			3937	c factor					
ECs_0742	ybgP	periplasmic pilin chaperone	VFG018320 (gb|WP_001044460)	Ste (VF0957)	-	setA	Salmonella enterica subsp. enterica serovar Paratyphi B str. SPB7	Adherence	steC	fimbria/pilus periplasmic chaperone	41.5	8.89E-53	171
ECs_3220	yfcS	periplasmic pilin chaperone	VFG018314 (gb|WP_000982729)	Stf (VF0958)	-	setA	Salmonella enterica subsp. enterica serovar Paratyphi B str. SPB7	Adherence	stfD	fimbrial chaperone StfD	60.5	2.08E-98	287
ECs_3839	mltC	membrane-bound lytic murein transglycosylase C	VFG041578 (gb|WP_012847749)	T3SS (VF1266)	-	setB	Edwardsiella tarda EIB202	Effector delivery system	ETAE_RS04220	transglycosylase SLT domain-containing protein	30.8	3.90E-11	62
ECs_2148	ydfG	NAD(P)-dependent oxidoreductase	VFG049994 (gb|WP_001048422)	Bacillibactin (VF0586)	Disruption of the siderophore bacillibactin production drastically reduces the ability of B. cereus to utilize ferritin for growth and	setA	Bacillus cereus ATCC 10987	Nutritional/Metabolic factor	dhbA	2,3-dihydroxybenzoate-2,3-dehydrogenase,	32.9	1.14E-29	111

					results in attenuated bacterial virulence in insects.					DhbA			
ECs_4233	hofQ	DNA catabolic fimbrial transporter	VFG013118 (gb|WP_005693728)	Type IV pili (VF0753)	-	setA	Haemophilus influenzae Rd KW20	Adherence	comE/pilQ	type IV pilus secretin PilQ family protein	40.3	7.28E-84	263
gene-hlyC	hlyC	Hemolysin C	VFG012488 (gb|NP_052623)	Hemolysin (VF0207)	Cytotoxic to many types of cells: erythrocytes, granulocytes, monocytes, endothelial cells and renal epithelial cells; stimulating the release of IL-1<beta> and TNF	setA	Escherichia coli O157:H7 str. Sakai	Exotoxin	hlyC	Hemolysin C	100	7.29E-123	342
ECs_2408	fadK	short chain acyl-CoA synthetase	VFG050149 (gb|WP_001972441)	Bacillibactin (VF0586)	Disruption of the siderophore bacillibactin production drastically reduces the ability of B. cereus to utilize ferritin for growth and results in attenuated bacterial virulence in insects.	setA	Bacillus cereus G9241	Nutritional/Metabolic factor	dhbE	2,3-dihydroxybenzoate adenylase DhbE	31.6	1.78E-79	259
ECs_2409	ppsA	phosphoenolpyruvate synthase	VFG015891 (gb|WP_003381696)	Phytotoxin phaseolotoxin (VF0917)	-	setB	Pseudomonas syringae pv. phaseolicola 1448A	Exotoxin	PSPPH_RS21790	pyruvate, phosphate dikinase	38.4	3.05E-07	53.9
ECs_0744	ybgD	fimbrial-like adhesin	VFG042651 (gb|CAC85333)	Pix pilus (VF1151)	-	setA	Escherichia coli str. X2194	Adherence	pixA	PixA protein	33.3	7.28E-23	89.7

		protein											
ECs_4433	yhjY	autotransporter beta-domain protein	VFG042313 (gb|NP_459562)	ApeE (VF0973)	-	setB	Salmonella enterica subsp. enterica serovar Typhimurium str. LT2	Effector delivery system	apeE	autotransporter outer membrane beta-barrel domain-containing protein	32.7	1.77E-21	92.4
ECs_4313	ftsY	signal recognition particle receptor	VFG007589 (gb|WP_011079849)	Flagella (VF0519)	Contributes to the virulence of pathogenic Vibrio through adhesion or biofilm formation	setA	Vibrio vulnificus CMCP6	Motility	flhF	flagellar biosynthesis protein	32.5	3.66E-18	87
ECs_3205	flk	flagella assembly protein	VFG043120 (gb|WP_000615801)	Peritrichous flagella (VF1154)	-	setB	Escherichia coli O157:H7 str. EDL933	Motility	flk	flagella biosynthesis regulator Flk	100	3.76E-229	625
ECs_4431	lpfA_1	fimbrial major protein precursor	VFG021364 (gb|WP_001747709)	Lpf (VF0105)	Mediate attachment to the Peyer's patches	setA	Salmonella enterica subsp. enterica serovar Agona str. SL483	Adherence	lpfA	long polar fimbria protein LpfA	73	2.70E-87	253

ECs_1276	ECs_1276	chaperone protein	VFG042697 (gb|NP_250822)	CupA fimbriae (VF0924)	-	setA	Pseudomonas aeruginosa PAO1	Biofilm	cupA5	chaperone CupA5	34.7	4.46E-41	140
ECs_1277	ECs_1277	outer membrane protein	VFG042696 (gb|NP_250821)	CupA fimbriae (VF0924)	-	setB	Pseudomonas aeruginosa PAO1	Biofilm	cupA4	fimbrial subunit CupA4	31.2	6.93E-40	148
ECs_1270	pgaA	poly-beta-1%2C6-N-acetyl-D-glucosamine export protein	VFG037656 (gb|WP_000913297)	PNAG (VF0472)	Critical for biofilm formation	setB	Acinetobacter baumannii D1279779	Biofilm	pgaA	poly-beta-1,6 N-acetyl-D-glucosamine export porin PgaA	24.7	3.55E-50	189
ECs_3218	yfcQ	fimbrial-like adhesin protein	VFG004333 (gb|WP_000737997)	Stf (VF0958)	-	setA	Salmonella enterica subsp. enterica serovar Choleraesuis str. SC-B67	Adherence	stfF	fimbrial minor subunit StfF	55.8	9.36E-48	151
ECs_1278	ECs_1278	outer membrane usher protein	VFG042695 (gb|NP_250820)	CupA fimbriae (VF0924)	-	setA	Pseudomonas aeruginosa PAO1	Biofilm	cupA3	usher CupA3	45.1	2.86E-239	692

ECs_0144	yadV	periplasmic pilin chaperone	VFG021423 (gb|WP_000882257)	Sta (VF0953)	-	setA	Salmonella enterica subsp. enterica serovar Agona str. SL483	Adherence	staB	fimbrial chaperone	59.8	5.16E-93	273
ECs_0587	ppiB	peptidyl-prolyl cis-trans isomerase B	VFG005372 (gb|WP_000731920)	Streptococcal lipoprotein rotamase A (VF1047)	-	setA	Streptococcus pneumoniae D39	Adherence	slrA	peptidylprolyl isomerase	36.5	1.78E-24	95.5
ECs_4214	ppiA	peptidyl-prolyl cis-trans isomerase A	VFG005372 (gb|WP_000731920)	Streptococcal lipoprotein rotamase A (VF1047)	-	setA	Streptococcus pneumoniae D39	Adherence	slrA	peptidylprolyl isomerase	34.7	1.55E-22	91.3
ECs_0586	lpxH	UDP-2%2C3-diacylglucosamine pyrophosphohydrola	VFG013326 (gb|WP_011608655)	LOS (VF0044)	Major immunogen; LOS phosphorylcholine (ChoP) may influence invasion via interaction with PAF receptor and stimulates of inflammatory signals; LPS phase	setA	Haemophilus somnus 129PT	Immune modulation	lpxH	UDP-2,3-diacylglucosamine pyrophosphohydrolase	49.6	2.75E-77	233

		se			variation is characterized by the spontaneous loss and gain of oligosaccharide structures present in the outer core. the phase variable expression of LPS biosynthesis genes promotes evasion of antigen-specific host immune defences and allow colonization of different host microenvironments								
ECs_1874	puuA	glutamate--putrescine ligase	VFG026433 (gb|YP_005360830)	Glutamine synthesis (VF0816)	-	setB	Mycobacterium tuberculosis RGTB327	Nutritional/Metabolic factor	glnA1	glutamine synthetase	26.6	2.93E-27	113
ECs_1879	puuE	4-aminobutyrate aminotransferase	VFG044201 (gb|WP_011267793)	Achromobactin biosynthesis and transport (VF0922)	-	setA	Pseudomonas syringae pv. syringae B728a	Nutritional/Metabolic factor	PSYR_RS13390	diaminobutyrate--2-oxoglutarate transaminase	36.6	2.12E-66	218
ECs_0158	hemL	glutamate-1-semialdehyde 2%2C1-aminomutase	VFG044201 (gb|WP_011267793)	Achromobactin biosynthesis and transport (VF0922)	-	setA	Pseudomonas syringae pv. syringae B728a	Nutritional/Metabolic factor	PSYR_RS13390	diaminobutyrate--2-oxoglutarate transaminase	32	5.61E-31	123
ECs_3523	gabT	4-aminobu	VFG044201	Achromoba	-	setA	Pseudomona	Nutritiona	PSYR_RS133	diaminobut	36.3	1.86E-	216

		tyrate aminotransferase	(gb|WP_011267793)	ctin biosynthesis and transport (VF0922)			s syringae pv. syringae B728a	l/Metabolic factor	90	yrate--2-oxoglutarate transaminase		65	
ECs_3955	patA	putrescine:2-oxoglutaric acid aminotransferase	VFG044201 (gb|WP_011267793)	Achromobactin biosynthesis and transport (VF0922)	-	setA	Pseudomonas syringae pv. syringae B728a	Nutritional/Metabolic factor	PSYR_RS13390	diaminobutyrate--2-oxoglutarate transaminase	35.5	7.53E-67	222
ECs_4626	ibpB	heat shock chaperone	VFG045327 (gb|WP_015444188)	GspA (VF0167)	19-kDa GroEL-like; dispensable for bacterial survival and growth in macrophage infection	setA	Legionella pneumophila subsp. pneumophila str. Philadelphia 1	Stress survival	LPG_RS11000	Hsp20 family protein	37.2	5.41E-25	93.6
ECs_4627	ibpA	heat shock chaperone	VFG045327 (gb|WP_015444188)	GspA (VF0167)	19-kDa GroEL-like; dispensable for bacterial survival and growth in macrophage infection	setA	Legionella pneumophila subsp. pneumophila str. Philadelphia 1	Stress survival	LPG_RS11000	Hsp20 family protein	42.8	2.55E-27	99.4
ECs_3422	glrK	two-compo	VFG031677	SenX3	-	setA	Mycobacter	Regulation	senX3	two-compon	27.4	1.95E-	92.8

		nent system sensor histidine kinase GlrK	(gb|WP_014876913)	(VF0857)			ium smegmatis str. MC2 155			ent system sensor histidine kinase SenX3		20	
ECs_1451	flgB	flagellar component of cell-proximal portion of basal-body rod	VFG043074 (gb|WP_000884708)	Peritrichous flagella (VF1154)	-	setA	Escherichia coli O157:H7 str. EDL933	Motility	flgB	flagellar basal body rod protein FlgB	100	2.39E-91	260
ECs_1456	flgG	flagellar component of cell-distal portion of basal-body rod	VFG043079 (gb|WP_000625837)	Peritrichous flagella (VF1154)	-	setA	Escherichia coli O157:H7 str. EDL933	Motility	flgG	flagellar basal-body rod protein FlgG	100	5.36E-182	499
ECs_1472	acpP	acyl carrier	VFG011430 (gb|WP_00	LPS (VF0367)	Plays a role in entry and early survival inside macrophages;	setA	Brucella melitensis	Immune modulation	acpXL	acyl carrier	63.5	1.07E-23	85.1

		protein	2963616)		Resistance to innate-immunity anti-bacterial responses; a modulator of the immune response		bv. 1 str. 16M			protein			
ECs_1457	flgH	flagellar protein of basal-body outer-membrane L ring	VFG043080 (gb|WP_001295442)	Peritrichous flagella (VF1154)	-	setA	Escherichia coli O157:H7 str. EDL933	Motility	flgH	flagellar basal body L-ring protein FlgH	100	1.81E-166	458
ECs_1454	flgE	flagellar hook protein	VFG043077 (gb|WP_000885860)	Peritrichous flagella (VF1154)	-	setA	Escherichia coli O157:H7 str. EDL933	Motility	flgE	flagellar hook protein FlgE	100	6.54E-279	756
ECs_1455	flgF	flagellar component of cell-proximal portion of basal-body rod	VFG043078 (gb|WP_000349287)	Peritrichous flagella (VF1154)	-	setA	Escherichia coli O157:H7 str. EDL933	Motility	flgF	flagellar basal-body rod protein FlgF	100	4.38E-172	474
ECs_2678	fliG	flagellar motor	VFG043108 (gb|WP_00	Peritrichous	-	setA	Escherichia coli	Motility	fliG	flagellar motor	99.7	4.90E-225	614

		switching and energizing component	0067959)	flagella (VF1154)			O157:H7 str. EDL933			switch protein FliG			
ECs_4331	ECs_4331	surfactin synthetase	VFG029731 (gb|WP_016342994)	GPL locus (VF0841)	-	setA	Mycobacterium abscessus subsp. bolletii 50594	Immune modulation	mps2	non-ribosomal peptide synthetase	27.4	4.74E-09	58.9
ECs_0058	surA	peptidyl-prolyl cis-trans isomerase	VFG043537 (gb|YP_002344026)	Adherence and biofilm formation (VF0723)	-	setA	Campylobacter jejuni subsp. jejuni NCTC 11168	Biofilm	PEB4	major antigenic peptide PEB-cell binding factor	29.7	2.66E-09	57.8
ECs_0495	ppiD	peptidyl-prolyl cis-trans isomerase D	VFG043537 (gb|YP_002344026)	Adherence and biofilm formation (VF0723)	-	setA	Campylobacter jejuni subsp. jejuni NCTC 11168	Biofilm	PEB4	major antigenic peptide PEB-cell binding factor	31	4.93E-06	48.5
ECs_4709	ppiC	peptidyl-prolyl cis-trans	VFG032880 (gb|WP_011702987)	PrsA2 (VF0449)	Required for virulence and contributes to the integrity of the L.monocytogenes cell wall as well as	setA	Listeria welshimeri serovar 6b	Post-translational modificati	prsA2	post translocation	41	1.91E-13	63.5

		isomerase C			swimming motility and bacterial resistance to osmotic stress		str. SLCC5334	on		chaperone PrsA2			
ECs_0057	pdxA	4-hydroxy-L-threonine phosphate dehydrogenase	VFG043391 (gb|WP_001075036)	Flagella (VF0051)	Confers motility, allows the bacteria to penetrate and colonize the gastric mucus layer. The lumenal pH of the fasting human stomach is <2, but with the gastric mucus there is a pH gradient that ranges from pH 2 at the luminal surface to nearly neutral pH at the epithelial cell surface, so entry into the gastric mucus layer is important for H. pylori to escape extremely low pH	setA	Helicobacter pylori 26695	Motility	pdxA	4-hydroxythreonine-4-phosphate dehydrogenase	32.5	1.66E-38	138
ECs_0490	tig	peptidyl-prolyl cis/trans isomerase	VFG005552 (gb|WP_011837486)	Trigger factor (VF1056)	-	setB	Streptococcus sanguinis SK36	Stress survival	tig/ropA	trigger factor	31.8	6.09E-51	177
ECs_0496	ybaV	competence-suppressing periplasmic helix-hairpin-helix DNA-bindi	VFG043478 (gb|WP_005752194)	ComE1 (VF1230)	-	setB	Pasteurella multocida subsp. multocida str. Pm70	Adherence	PM_RS08640	ComEA family DNA-binding protein	42.9	8.06E-14	62.8

		ng protein											
ECs_1140	gfcD	O-antigen capsule production periplasmic protein	VFG007643 (gb|WP_011261015)	Capsular polysaccharide (VF0624)	-	setB	Vibrio fischeri ES114	Immune modulation	wbfB	YjbH domain-containing protein	44.3	9.77E-206	597
ECs_5012	gfcD_2	O-antigen capsule production periplasmic protein	VFG007643 (gb|WP_011261015)	Capsular polysaccharide (VF0624)	-	setB	Vibrio fischeri ES114	Immune modulation	wbfB	YjbH domain-containing protein	46.6	1.30E-219	632
ECs_0635	entA	2%2C3-dihydro-2%2C3-dihydroxybenzoate dehydrogenase	VFG012977 (gb|WP_000347664)	Enterobactin synthesis and transport (VF0984)	-	setA	Shigella boydii Sb227	Nutritional/Metabolic factor	entA	2,3-dihydro-2,3-dihydroxybenzoate dehydrogenase EntA	98.8	1.42E-171	472
ECs_0631	fepB	ferrienterobactin ABC	VFG000924 (gb|WP_001234311)	Enterobactin (VF0228)	Iron uptake: the siderophore enterobactin imported through the FepA receptor and the FepBCDG system	setA	Escherichia coli CFT073	Nutritional/Metabolic factor	fepB	ferrienterobactin ABC transporte	98.4	5.49E-220	600

		transporter periplasmic binding protein								r periplasmic binding protein			
ECs_0630	entS	enterobactin exporter	VFG044165 (gb|WP_001041793)	Enterobactin (VF0228)	Iron uptake: the siderophore enterobactin imported through the FepA receptor and the FepBCDG system	setA	Escherichia coli CFT073	Nutritional/Metabolic factor	entS	enterobactin exporter, iron-regulated	99.3	3.80E-281	763
ECs_0633	entE	2%2C3-dihydroxybenzoate-AMP ligase component of enterobactin synthase multienzyme complex	VFG012990 (gb|WP_000026810)	Enterobactin synthesis and transport (VF0984)	-	setA	Shigella sonnei Ss046	Nutritional/Metabolic factor	entE	(2,3-dihydroxybenzoyl)adenylate synthase EntE	98.9	0	1060
ECs_0632	entC	isochorismate synthase	VFG012991 (gb|WP_000381303)	Enterobactin synthesis	-	setA	Shigella dysenteriae Sd197	Nutritional/Metabolic factor	entC	isochorismate synthase	100	5.41E-282	763

		1		and transport (VF0984)						EntC			
ECs_3583	hypB	GTP hydrolase involved in nickel liganding into hydrogenases	VFG006496 (gb|WP_011115254)	Urease (VF0050)	An important colonization factor, contributes to acid resistance, epithelial cell damage, chemotactic behavior, and nitrogen metabolism	setA	Helicobacter hepaticus ATCC 51449	Stress survival	ureG	urease accessory protein (ureG)	27.9	4.21E-10	58.2
ECs_1327	ureG	urease accessory protein	VFG006496 (gb|WP_011115254)	Urease (VF0050)	An important colonization factor, contributes to acid resistance, epithelial cell damage, chemotactic behavior, and nitrogen metabolism	setA	Helicobacter hepaticus ATCC 51449	Stress survival	ureG	urease accessory protein (ureG)	61.2	5.94E-91	265
ECs_4720	wecC	UDP-N-acetyl-D-mannosaminuronic acid dehydrogenase	VFG014138 (gb|WP_003138473)	LPS (VF0085)	Mediates biological effects including resistance to serum killing and phagocytosis; the binding to normal CFTR (cystic fibrosis transmembrane conductance regulator) and invasion of host cells may make a contribution to virulence in the human eye; internalization by binding to normal CFTR protein expressed by airway epithelial cells followed by desquamation of bacteria-laden	setA	Pseudomonas aeruginosa UCBPP-PA14	Immune modulation	wecC	UDP-N-acetyl-D-mannosamine dehydrogenase	67.4	7.68E-207	576

					epithelial cells, constitutes a host defense mechanism. If this mechanism fails to function properly, abnormally high bacterial carriage would promote the establishment of chronic bacterial infection							
ECs_4725	wzxE	O-antigen translocase WzxE	VFG038013 (gb|WP_001050695)	Capsule (VF0465)	Plays an important role in protecting bacteria from the host innate immune response	setB	Acinetobacter baumannii TYTH-1	Immune modulation	M3Q_RS01470	O-antigen translocase	28.3	1.43E-55	189
ECs_4576	cesD	T3SS chaperone CesD	VFG000820 (gb|WP_000087467)	TTSS (VF0191)	Injects Tir and other effector molecules directly into the host cell. Effector molecules activate cell-signaling pathways, causing alterations in the host cell cytoskeleton and resulting in the depolymerization of actin and the loss of microvilli	setA	Escherichia coli O157:H7 str. EDL933	Effector delivery system	cesD	chaperone for EspB and EspD	100	4.68E-111	311
ECs_3565	norR	anaerobic nitric oxide reductase DNA-binding transcriptional	VFG014388 (gb|WP_010955091)	Flagella (VF0273)	Swimming motility; play a role in biofilm formation and other pathogenic adaptations	setA	Pseudomonas putida KT2440	Motility	fleR	two-component response regulator	51.4	5.23E-73	238

		activator											
ECs_5216	treC	trehalose-6-P hydrolase	VFG050222 (gb|NP_219545)	TTSS secreted effectors (VF0711)	CadD (Iron-containing redox enzyme. Contains death domain motif and is capable of interacting with TNF family receptors.); CdsZ; CopN (Contribute to manipulation of microtubule networks, delays host cell division and supports the intracellular growth of Chlamydia.); CpoS (May play a role in regulating the intracellular trafficking or fusogenicity of the chlamydial inclusion.); CteG (Might function by subverting host cell vesicular transport.); FilF; InaC (Recruits and activates host ADP-ribosylation factor 1 (ARF1) and ARF4 to regulate microtubules.); IncA (SNARE mimicry. Associated with homotypic fusion of inclusions and SNARE recruitment in C. trachomatis.); IncB; IncC; IncD (Formation of ER-inclusion MCS, non-vesicular lipid acquisition.); IncE (Manipulates retromer-mediated transport.); IncG (Early-phase effector.); IncV (Formation of	setA	Chlamydia trachomatis D/UW-3/CX	Effector delivery system	glgX	glycogen hydrolase	25.6	7.28E-07	52

ER-inclusion membrane contact sites (MCS).); IPAM (Hijacks microtubule organizing functions and controls microtubule assembly.); Mcsc; MrcA (Promotes Chlamydia extrustion.); NUE (Histone methyltransferase.); Pkn5 (Ser/Thr kinase.); Tarp (Actin-binding protein. Recuitment and nucleation of actin to facillitate entry of Ebs into host cells; Tarp can be phosphorylated on tyrosine residues and is proposed to function similarly to EPEC Tir.); TepP (Regulates innate immune signaling early in infection.); TmeA (Host cell invasion.); CrpA; GlgA; GlgX; YycJ; CT_006; CT_053; CT_061; CT_082; CT_083; CT_117; CT_134; CT_135; CT_142; CT_143; CT_144; CT_147; CT_156; CT_161; CT_163; CT_179; CT_192; CT_203; CT_222; CT_224; CT_225; CT_226; CT_227; CT_228 (Inhibits chlamydial extrusion.); CT_249; CT_288; CT_345; CT_358; CT_383; CT_429; CT_440; CT_449; CT_473; CT_483; CT_529; CT_550;

ECs_4454	malS	alpha-amylase	VFG050222 (gb|NP_219545)	TTSS secreted effectors (VF0711)	CT_565; CT_577; CT_606.1; CT_618; CT_619; CT_620; CT_621 (DUF582 domain protein, function unknown.); CT_622; CT_652.1; CT_656; CT_668; CT_695; CT_711; CT_712; CT_718; CT_847 (Interacts with human Grap2 Cyclin D-interacting protein (GCIP), and may contribute to observed degradation of GCIP during chlamydial infection.); CT_848; CT_849; CT_850; CT_863 CadD (Iron-containing redox enzyme. Contains death domain motif and is capable of interacting with TNF family receptors.); CdsZ; CopN (Contribute to manipulation of microtubule networks, delays host cell division and supports the intracellular growth of Chlamydia.); CpoS (May play a role in regulating the intracellular trafficking or fusogenicity of the chlamydial inclusion.); CteG (Might function by subverting host cell vesicular transport.); FilF; InaC (Recruits and activates host ADP-ribosylation factor 1 (ARF1) and ARF4 to regulate microtubules.); IncA	setA	Chlamydia trachomatis D/UW-3/CX	Effector delivery system	glgX	glycogen hydrolase	34	9.84E-07	52

(SNARE mimicry. Associated with homotypic fusion of inclusions and SNARE recruitment in C. trachomatis.); IncB; IncC; IncD (Formation of ER-inclusion MCS, non-vesicular lipid acquisition.); IncE (Manipulates retromer-mediated transport.); IncG (Early-phase effector.); IncV (Formation of ER-inclusion membrane contact sites (MCS).); IPAM (Hijacks microtubule organizing functions and controls microtubule assembly.); Mcsc; MrcA (Promotes Chlamydia extrustion.); NUE (Histone methyltransferase.); Pkn5 (Ser/Thr kinase.); Tarp (Actin-binding protein. Recuitment and nucleation of actin to facillitate entry of Ebs into host cells; Tarp can be phosphorylated on tyrosine residues and is proposed to function similarly to EPEC Tir.); TepP (Regulates innate immune signaling early in infection.); TmeA (Host cell invasion.); CrpA; GlgA; GlgX; YycJ; CT_006; CT_053; CT_061; CT_082;

ECs_4276	glgX	glycogen debranching enzyme	VFG050222 (gb|NP_219545)	TTSS secreted effectors (VF0711)	CT_083; CT_117; CT_134; CT_135; CT_142; CT_143; CT_144; CT_147; CT_156; CT_161; CT_163; CT_179; CT_192; CT_203; CT_222; CT_224; CT_225; CT_226; CT_227; CT_228 (Inhibits chlamydial extrusion.); CT_249; CT_288; CT_345; CT_358; CT_383; CT_429; CT_440; CT_449; CT_473; CT_483; CT_529; CT_550; CT_565; CT_577; CT_606.1; CT_618; CT_619; CT_620; CT_621 (DUF582 domain protein, function unknown.); CT_622; CT_652.1; CT_656; CT_668; CT_695; CT_711; CT_712; CT_718; CT_847 (Interacts with human Grap2 Cyclin D-interacting protein (GCIP), and may contribute to observed degradation of GCIP during chlamydial infection.); CT_848; CT_849; CT_850; CT_863 CadD (Iron-containing redox enzyme. Contains death domain motif and is capable of interacting with TNF family receptors.); CdsZ; CopN (Contribute to manipulation of microtubule networks, delays host cell division and supports the intracellular growth	setA	Chlamydia trachomatis D/UW-3/CX	Effector delivery system	glgX	glycogen hydrolase	36.6	4.43E-107	338

of Chlamydia.); CpoS (May play a role in regulating the intracellular trafficking or fusogenicity of the chlamydial inclusion.); CteG (Might function by subverting host cell vesicular transport.); FilF; InaC (Recruits and activates host ADP-ribosylation factor 1 (ARF1) and ARF4 to regulate microtubules.); IncA (SNARE mimicry. Associated with homotypic fusion of inclusions and SNARE recruitment in C. trachomatis.); IncB; IncC; IncD (Formation of ER-inclusion MCS, non-vesicular lipid acquisition.); IncE (Manipulates retromer-mediated transport.); IncG (Early-phase effector.); IncV (Formation of ER-inclusion membrane contact sites (MCS).); IPAM (Hijacks microtubule organizing functions and controls microtubule assembly.); Mcsc; MrcA (Promotes Chlamydia extrustion.); NUE (Histone methyltransferase.); Pkn5 (Ser/Thr kinase.); Tarp (Actin-binding protein. Recuitment

and nucleation of actin to facillitate entry of Ebs into host cells; Tarp can be phosphorylated on tyrosine residues and is proposed to function similarly to EPEC Tir.); TepP (Regulates innate immune signaling early in infection.); TmeA (Host cell invasion.); CrpA; GlgA; GlgX; YycJ; CT_006; CT_053; CT_061; CT_082; CT_083; CT_117; CT_134; CT_135; CT_142; CT_143; CT_144; CT_147; CT_156; CT_161; CT_163; CT_179; CT_192; CT_203; CT_222; CT_224; CT_225; CT_226; CT_227; CT_228 (Inhibits chlamydial extrusion.); CT_249; CT_288; CT_345; CT_358; CT_383; CT_429; CT_440; CT_449; CT_473; CT_483; CT_529; CT_550; CT_565; CT_577; CT_606.1; CT_618; CT_619; CT_620; CT_621 (DUF582 domain protein, function unknown.); CT_622; CT_652.1; CT_656; CT_668; CT_695; CT_711; CT_712; CT_718; CT_847 (Interacts with human Grap2 Cyclin D-interacting protein (GCIP), and may contribute to observed degradation of

					GCIP during chlamydial infection.); CT_848; CT_849; CT_850; CT_863								
ECs_5219	mgtA	magnesium transporter	VFG018402 (gb|WP_000131305)	MgtBC (VF0106)	MgtA and MgtB are not required for intracellular survival or for virulence, MgtC is essential for both functions	setA	Salmonella enterica subsp. arizonae serovar 62:z4,z23:-- str. RSK2980	Nutritional/Metabolic factor	mgtB	Mg2+ transport protein	52.6	9.31E-308	870
ECs_2567	znuA	zinc ABC transporter periplasmic binding protein	VFG005232 (gb|WP_002913975)	Lmb (VF0275)	34 kDa lipoprotein mediates the attachment to human laminin, a major component of the basement membrane, which may be essential for the bacterial colonization; also important for the invasion of human brain endothelial cells	setA	Streptococcus sanguinis SK36	Adherence	lmb	laminin-binding surface protein	25.2	1.81E-19	87
ECs_2566	mepM	murein DD-endopeptidase	VFG042906 (gb|WP_012908549)	Colonization factor Citrobacter (CFC) type IV fimbriae (VF1209)	-	setB	Citrobacter rodentium ICC168	Adherence	ROD_RS25695	M23 family metallopeptidase	34.9	3.68E-24	103
ECs_2565	lpxM	myristoyl	VFG020187	MsbB2	-	setA	Shigella	Others	msbB2	lauroyl-Kd	70.2	4.12E-	467

		-acyl carrier protein ACP-dependent acyltransferase	(gb|WP_012421740)	(VF0981)			boydii CDC 3083-94			o(2)-lipid IV(A) myristoyltransferase		167	
ECs_2569	znuB	zinc ABC transporter permease	VFG012582 (gb|WP_001101732)	Iron/manganese transport (VF1116)	-	setA	Escherichia coli 536	Nutritional/Metabolic factor	sitC	iron/manganese ABC transporter permease subunit SitC	27.6	1.64E-20	88.2
ECs_2568	znuC	zinc ABC transporter ATPase	VFG044077 (gb|YP_002344985)	Direct heme uptake system (VF0724)	-	setA	Campylobacter jejuni subsp. jejuni NCTC 11168	Nutritional/Metabolic factor	chuC	hemin uptake ABC transporter ATP-binding protein	35.7	7.07E-34	122
ECs_2852	wcaJ	colanic biosynthesis UDP-glucose lipid carrier transfera	VFG048942 (gb|WP_012967595)	Capsule (VF0560)	Assisting in evading the host immune system by protecting bacteria from opsonophagocytosis and serum killing	setA	Klebsiella variicola At-22	Immune modulation	wcaJ	undecaprenyl-phosphate glucose phosphotransferase	66.2	6.52E-230	638

		se											
ECs_2851	wzxC	colanic acid exporter	VFG048850 (gb|WP_004899428)	Capsule (VF0560)	Assisting in evading the host immune system by protecting bacteria from opsonophagocytosis and serum killing	setB	Klebsiella pneumoniae KCTC 2242	Immune modulation	KPN2242_RS16275	MOP flippase family protein	45.8	2.09E-136	402
gene-hlyA	hlyA	Hemolysin A	VFG000840 (gb|WP_000217739)	Hemolysin (VF0207)	Cytotoxic to many types of cells: erythrocytes, granulocytes, monocytes, endothelial cells and renal epithelial cells; stimulating the release of IL-1<beta> and TNF	setA	Escherichia coli O157:H7 str. EDL933	Exotoxin	hlyA	Hemolysin A	100	0	1573
ECs_5033	qorA	quinone oxidoreductase	VFG008554 (gb|WP_015291763)	Sulfolipid-1 biosynthesis and transport (VF0803)	Sulfoglycolipids inhibit NF-<kappa>B activation and subsequent cytokine production or costimulatory molecule expression by acting as competitive antagonists of Toll-like receptor 2	setA	Mycobacterium canettii CIPT 140070010	Immune modulation	pks2	phthioceranic/hydroxyphthioceranic acid synthase	29.5	3.59E-26	109
ECs_2855	wcaI	glycosyl transferase	VFG048852 (gb|WP_015875022)	Capsule (VF0560)	Assisting in evading the host immune system by protecting bacteria from opsonophagocytosis and serum killing	setA	Klebsiella pneumoniae subsp. pneumoniae NTUH-K2044	Immune modulation	KP1_RS17295	glycosyltransferase WbuB	61.9	5.95E-182	511
ECs_2418	btuC	vitamin B12 ABC transporter permease	VFG044101 (gb|NP_253395)	Direct heme uptake system (VF0941)	-	setA	Pseudomonas aeruginosa PAO1	Nutritional/Metabolic factor	PA4707	ABC transporter permease	46.9	1.76E-66	211

ECs_4426	lpfE	fimbrial adhesin protein	VFG000452 (gb|NP_462537)	Lpf (VF0105)	Mediate attachment to the Peyer's patches	setA	Salmonella enterica subsp. enterica serovar Typhimurium str. LT2	Adherence	lpfE	long polar fimbrial minor subunit LpfE, adhesin	50.9	3.38E-48	154
ECs_2412	ydiE	hemin uptake protein	VFG044241 (gb|WP_011815407)	Direct heme uptake system (VF1031)	-	setB	Yersinia enterocolitica subsp. enterocolitica 8081	Nutritional/Metabolic factor	YE_RS01740	hemin uptake protein HemP	55.6	3.27E-07	42.7
ECs_2414	ydiV	cyclic di-GMP regulator CdgR	VFG042389 (gb|WP_000012013)	S fimbriae (VF0222)	S fimbriae binds to receptors containing sialic acid sugar moieties, the sialic acid residues are presented on UP3, one of four integral membrane uroplakin proteins	setA	Escherichia coli UTI89	Adherence	sfaY	regulatory protein SfaY	20.1	1.85E-09	56.2
ECs_4427	lpfD_1	minor fimbrial subunit	VFG021344 (gb|WP_000914254)	Lpf (VF0105)	Mediate attachment to the Peyer's patches	setA	Salmonella enterica subsp. enterica serovar Dublin str. CT_02021853	Adherence	lpfD	long polar fimbrial protein LpfD	41.6	1.40E-86	265
ECs_3644	relA	(p)ppGpp	VFG010924	RelA	Dispensable for intracellular growth	setA	Legionella	Regulation	relA	GTP	45.2	1.82E-	640

		synthetase RelA	(gb|WP_011215608)	(VF0260)	within amoebae or the alveolar macrophages, but dedicate to convert from a replicated to a virulent state		pneumophila str. Lens			diphosphokinase		222	
ECs_3217	yfcP	fimbrial-like adhesin protein	VFG018311 (gb|WP_001129318)	Stf (VF0958)	-	setB	Salmonella enterica subsp. enterica serovar Paratyphi B str. SPB7	Adherence	stfG	fimbrial protein	47	1.29E-39	132
ECs_1269	pgaB	poly-beta-1%2C6-N-acetyl-D-glucosamine N-deacetylase outer membrane export lipoprotein	VFG037662 (gb|WP_001061302)	PNAG (VF0472)	Critical for biofilm formation	setA	Acinetobacter baumannii AB0057	Biofilm	pgaB	poly-beta-1,6-N-acetyl-D-glucosamine N-deacetylase PgaB	32.6	1.20E-59	211
ECs_1268	pgaC	in biofilm PGA synthesis N-glycosy	VFG037682 (gb|WP_000866236)	PNAG (VF0472)	Critical for biofilm formation	setA	Acinetobacter baumannii BJAB0715	Biofilm	pgaC	poly-beta-1,6 N-acetyl-D-glucosami	52.5	1.57E-144	418

		ltransferase PgaC								ne synthase			
ECs_4413	bcsA	cellulose synthase catalytic subunit	VFG004543 (gb|WP_001159430)	Intercellular adhesion proteins (VF0014)	PNSG is critical to biofilm elaboration, allowing bacteria to adhere to one another, and may also promote adherence to other molecules, such as ECM components	setA	Staphylococcus aureus subsp. aureus USA300_FPR3757	Biofilm	icaA	N-acetylglucosaminyltransferase, involved in polysaccharide intercellular adhesin(PIA) synthesis	21.2	5.83E-07	52.8
ECs_1267	pgaD	poly-beta-1%2C6-N-acetyl-D-glucosamine biosynthesis	VFG037692 (gb|WP_000786639)	PNAG (VF0472)	Critical for biofilm formation	setA	Acinetobacter baumannii ACICU	Biofilm	pgaD	poly-beta-1,6-N-acetyl-D-glucosamine biosynthesis protein PgaD	32.7	4.83E-07	46.2
ECs_1041	ompA	outer membrane protein OmpA	VFG043544 (gb|WP_000750416)	Z1307 (VF1158)	-	setA	Escherichia coli O157:H7 str. EDL933	Adherence	ompA	porin OmpA	100	3.48E-253	687
ECs_4774	fadB	multifunc	VFG043691	Colibacti	-	setA	Escherichi	Exotoxin	clbD	colibactin	31.4	1.05E-	125

		tional fatty acid oxidation complex subunit alpha	(gb|WP_000982270)	n (VF1179)			a coli O18:K1:H7 str. IHE3034			biosynthesis dehydrogenase ClbD		31	
ECs_3224	fadJ	fatty acid oxidation complex subunit alpha	VFG043691 (gb|WP_000982270)	Colibactin (VF1179)	-	setA	Escherichia coli O18:K1:H7 str. IHE3034	Exotoxin	clbD	colibactin biosynthesis dehydrogenase ClbD	30.3	6.28E-33	128
ECs_5011	yjbG	extracellular polysaccharide export OMA protein	VFG007644 (gb|WP_011261016)	Capsular polysaccharide (VF0624)	-	setB	Vibrio fischeri ES114	Immune modulation	wbfC	capsule biosynthesis GfcC family protein	31	1.02E-13	68.6
ECs_1461	flgL	flagellar hook-filament junction protein	VFG043084 (gb|WP_001212790)	Peritrichous flagella (VF1154)	-	setA	Escherichia coli O157:H7 str. EDL933	Motility	flgL	flagellar hook-associated protein FlgL	100	3.14E-214	586
ECs_1460	flgK	flagellar	VFG043083	Peritrich	-	setA	Escherichi	Motility	flgK	flagellar	100	0	1011

		hook-filament junction protein 1	(gb|WP_000096470)	ous flagella (VF1154)			a coli O157:H7 str. EDL933			hook-associated protein FlgK			
ECs_0487	ampG	muropeptide transporter	VFG044297 (gb|WP_000974234)	Yersiniabactin-related (VF1251)	-	setA	Proteus mirabilis HI4320	Nutritional/Metabolic factor	PMI_RS12825	MFS transporter	22.9	5.46E-17	82.8
ECs_4327	ECs_4327	acyltransferase	VFG015608 (gb|WP_011920370)	Acylhomoserine lactone synthase (VF0907)	-	setB	Pseudomonas mendocina ymp	Biofilm	hdtS	1-acyl-sn-glycerol-3-phosphate acyltransferase	33	4.21E-11	61.6
ECs_3902	plsC	1-acyl-sn-glycerol-3-phosphate acyltransferase	VFG015608 (gb|WP_011920370)	Acylhomoserine lactone synthase (VF0907)	-	setB	Pseudomonas mendocina ymp	Biofilm	hdtS	1-acyl-sn-glycerol-3-phosphate acyltransferase	31.8	2.57E-10	58.9
ECs_4323	yhhT	transporter	VFG044071 (gb|NP_214719)	Heme uptake (VF0859)	-	setB	Mycobacterium tuberculosis H37Rv	Nutritional/Metabolic factor	Rv0205	transmembrane protein	26.1	1.36E-14	73.9
ECs_2307	tqsA	pheromone AI-2 transport	VFG044071 (gb|NP_214719)	Heme uptake (VF0859)	-	setB	Mycobacterium tuberculos	Nutritional/Metabolic factor	Rv0205	transmembrane protein	31.7	6.76E-07	50.4

		er					is H37Rv						
ECs_1996	nleG5-1	T3SS secreted effector NleG	VFG035012 (gb|WP_001143784)	TTSS secreted effectors (VF1110)	Cif (Deamidase. Induces cytopathic effects of actin stress fiber formation and cell cycle arrest.); EspB (Pore formation, actin disruption, microvilli effacement, anti-phagocytosis.); EspF (Inducing degradation of the aniapoptic protein AbcF2, tight junction disruption, microvilli effacement and elongation, mitochondrial dysfunction, N-WASP activation, SGLT-1 inactivation, pedestal maturation, inhibition of NHE3 activity, membrane remodelling; targets and disrupts the nucleolus late in infection, which is temporally controlled by host mitochondria.); EspFu/tccP (Inducing degradation of the aniapoptic protein AbcF2, tight junction disruption, microvilli effacement and elongation, mitochondrial dysfunction, N-WASP activation, SGLT-1 inactivation, pedestal maturation, inhibition of NHE3 activity, membrane remodelling; targets and disrupts the nucleolus	setA	Escherichia coli O157:H7 str. EDL933	Effector delivery system	nleG5-1	DUF1076 domain-containing protein	100	4.47E-153	422

late in infection, which is temporally controlled by host mitochondria.); EspG (TBC-like GTPase activating protein. Efficiently catalyzes GTP hydrolysis in Rab1 to disrupt of Rab1-mediated ER-to-Golgi trafficking.); EspH (First bacterial effector acting directly on RhoGEFs, EspH directly binds to the DH-PH domain in RhoGEFs to disrupt RhoGEF-Rho signaling; critical for inhibiting macrophage phagocytosis.); EspJ (Inhibit both IgG- and complement receptor-mediated phagocytosis.); EspK; EspL1; EspL2 (Cysteine protease. Bounds F-actin-aggregating annexin 2 directly to increase annexin 2's ability to aggregate Tir-induced F-actin; block necroptosis and in flammation.); EspL4; EspM1 (GEF. Activates the RhoA signaling pathway and induce the formation of stress fibres; inhibit pedestal formation and induce tight junction mislocalization.); EspM2 (GEF. Activates the RhoA signaling pathway

and induce the formation of stress fibres; inhibit pedestal formation and induce tight junction mislocalization.); EspN; EspO1-1; EspO1-2; EspR1; EspR3; EspR4; EspT (GEF. Activates Rac1 and Cdc42 leading to formation of membrane ruffles and lamellipodia; induces membrane ruffles to facilitate bacterial invasion into non-phagocytic cells in a process involving Rac1 and Wave2.); EspW; EspX1; EspX2; EspX4; EspX5; EspX6; EspX7/nleL (E3 ubiquitin ligase, HECT-like. Modulates pedestal formation.); EspY1; EspY2; EspY3; EspY4; EspY5; Map (GEF. Mimics the host Dbl and catalyses the exchange of GDP for GTP in Cdc42, involved in effacement, SGLT1 inhibition, formation of filopodia and disruption of mitochondrial function.); NleA/espI (Disruption of tight junctions by inhibition of host cell protein trafficking through COPII-dependent pathways.); NleB1 (Blocks translocation of the p65 and to

the host cell nucleus to inhibit NF-<kappa>B pathway, but NleE and NleB act at different points in the NF-<kappa>B signaling pathway.); NleB2 (May also have anti-inflammatory activity.); NleC (Metalloprotease. Zn-dependent endopeptidases that specifically clip and inactivate RelA (p65), thus blocking NF-<kappa>B pathway.); NleD (Metalloprotease. Zn-dependent endopeptidases that specifically clip and inactivate JNK and p38, thus blocking AP-1 pathway.); NleE (PMN tran-epithelial migration; blocks translocation of the p65 to the host cell nucleus by preventing I<kappa>B degradation to inhibit NF-<kappa>B pathway.); NleF; NleG-1; NleG2-2; NleG2-3; NleG2-4; NleG5-1; NleG5-2; NleG6-1; NleG6-2; NleG6-3; NleG7 (U-box type E3 ubiquitin ligases.); NleG8-2; NleH1 (Ser/Thr protein kinase. Binds directly to a subunit of NF-<kappa>B, the ribosomal protein S3 (RPS3), reducing the nuclear abundance of RPS3 to dampen

					host transcriptional outputs; interact with Bax inhibitor-1 to block apoptosis.); NleH2 (Putative kinase. Attenuates NF-<kappa>B pathway.); SepZ/espZ (EspZ interacts with CD98 in host cell membranes to promote host cell survival, therefore provide the pathogen with valuable time to colonize efficiently prior to dissemination.); TccP2; Tir (Mimics host immunoreceptor tyrosine-based inhibition motifs (ITIMs), also see helicobacter CagA. EHEC Tir lacks the Nck binding site. Conserved NPY (Asn-Pro-Tyr) motif recruits the adaptor protein IRTKS and/or IRSp53. IRTKS/IRSp53 link Tir and TccP/EspFu, which in turn activates N-WASP; Receptor for intimin; effacement; SGLT1 inhibition; recruits SHIP2 to control actin-pedestal morphology; maintains the integrity of the epithelium by keeping the destructive activity of EspG and EspG2 in check.)								
ECs_2154	nleG5-2	T3SS secreted	VFG035012 (gb|WP_00	TTSS secreted	Cif (Deamidase. Induces cytopathic effects of actin stress fiber	setA	Escherichi a coli	Effector delivery	nleG5-1	DUF1076 domain-con	92	1.17E-139	389

effector NleG	1143784)	effectors (VF1110)	formation and cell cycle arrest.); EspB (Pore formation, actin disruption, microvilli effacement, anti-phagocytosis.); EspF (Inducing degradation of the aniapoptic protein AbcF2, tight junction disruption, microvilli effacement and elongation, mitochondrial dysfunction, N-WASP activation, SGLT-1 inactivation, pedestal maturation, inhibition of NHE3 activity, membrane remodelling; targets and disrupts the nucleolus late in infection, which is temporally controlled by host mitochondria.); EspFu/tccP (Inducing degradation of the aniapoptic protein AbcF2, tight junction disruption, microvilli effacement and elongation, mitochondrial dysfunction, N-WASP activation, SGLT-1 inactivation, pedestal maturation, inhibition of NHE3 activity, membrane remodelling; targets and disrupts the nucleolus late in infection, which is temporally controlled by host mitochondria.); EspG (TBC-like GTPase activating	O157:H7 str. EDL933	system	taining protein

protein. Efficiently catalyzes GTP hydrolysis in Rab1 to disrupt of Rab1-mediated ER-to-Golgi trafficking.); EspH (First bacterial effector acting directly on RhoGEFs, EspH directly binds to the DH-PH domain in RhoGEFs to disrupt RhoGEF-Rho signaling; critical for inhibiting macrophage phagocytosis.); EspJ (Inhibit both IgG- and complement receptor-mediated phagocytosis.); EspK; EspL1; EspL2 (Cysteine protease. Bounds F-actin-aggregating annexin 2 directly to increase annexin 2's ability to aggregate Tir-induced F-actin; block necroptosis and in flammation.); EspL4; EspM1 (GEF. Activates the RhoA signaling pathway and induce the formation of stress fibres; inhibit pedestal formation and induce tight junction mislocalization.); EspM2 (GEF. Activates the RhoA signaling pathway and induce the formation of stress fibres; inhibit pedestal formation and induce tight junction

mislocalization.); EspN; EspO1-1; EspO1-2; EspR1; EspR3; EspR4; EspT (GEF. Activates Rac1 and Cdc42 leading to formation of membrane ruffles and lamellipodia; induces membrane ruffles to facilitate bacterial invasion into non-phagocytic cells in a process involving Rac1 and Wave2.); EspW; EspX1; EspX2; EspX4; EspX5; EspX6; EspX7/nleL (E3 ubiquitin ligase, HECT-like. Modulates pedestal formation.); EspY1; EspY2; EspY3; EspY4; EspY5; Map (GEF. Mimics the host Dbl and catalyses the exchange of GDP for GTP in Cdc42, involved in effacement, SGLT1 inhibition, formation of filopodia and disruption of mitochondrial function.); NleA/espI (Disruption of tight junctions by inhibition of host cell protein trafficking through COPII-dependent pathways.); NleB1 (Blocks translocation of the p65 and to the host cell nucleus to inhibit NF-<kappa>B pathway, but NleE and NleB act at different points in the

NF-<kappa>B signaling pathway.); NleB2 (May also have anti-inflammatory activity.); NleC (Metalloprotease. Zn-dependent endopeptidases that specifically clip and inactivate RelA (p65), thus blocking NF-<kappa>B pathway.); NleD (Metalloprotease. Zn-dependent endopeptidases that specifically clip and inactivate JNK and p38, thus blocking AP-1 pathway.); NleE (PMN tran-epithelial migration; blocks translocation of the p65 to the host cell nucleus by preventing I<kappa>B degradation to inhibit NF-<kappa>B pathway.); NleF; NleG-1; NleG2-2; NleG2-3; NleG2-4; NleG5-1; NleG5-2; NleG6-1; NleG6-2; NleG6-3; NleG7 (U-box type E3 ubiquitin ligases.); NleG8-2; NleH1 (Ser/Thr protein kinase. Binds directly to a subunit of NF-<kappa>B, the ribosomal protein S3 (RPS3), reducing the nuclear abundance of RPS3 to dampen host transcriptional outputs; interact with Bax inhibitor-1 to block apoptosis.); NleH2 (Putative kinase.

ECs_1995	nleG6-1	T3SS secreted effector NleG	VFG035025 (gb|WP_001443810)	TTSS secreted effectors (VF1110)	Attenuates NF-<kappa>B pathway.); SepZ/espZ (EspZ interacts with CD98 in host cell membranes to promote host cell survival, therefore provide the pathogen with valuable time to colonize efficiently prior to dissemination.); TccP2; Tir (Mimics host immunoreceptor tyrosine-based inhibition motifs (ITIMs), also see helicobacter CagA. EHEC Tir lacks the Nck binding site. Conserved NPY (Asn-Pro-Tyr) motif recruits the adaptor protein IRTKS and/or IRSp53. IRTKS/IRSp53 link Tir and TccP/EspFu, which in turn activates N-WASP; Receptor for intimin; effacement; SGLT1 inhibition; recruits SHIP2 to control actin-pedestal morphology; maintains the integrity of the epithelium by keeping the destructive activity of EspG and EspG2 in check.) Cif (Deamidase. Induces cytopathic effects of actin stress fiber formation and cell cycle arrest.); EspB (Pore formation, actin disruption, microvilli effacement,	setA	Escherichia coli O157:H7 str. EDL933	Effector delivery system	nleG6-1	DUF1076 domain-containing protein	100	2.22E-151	418

anti-phagocytosis.); EspF (Inducing degradation of the aniapoptic protein AbcF2, tight junction disruption, microvilli effacement and elongation, mitochondrial dysfunction, N-WASP activation, SGLT-1 inactivation, pedestal maturation, inhibition of NHE3 activity, membrane remodelling; targets and disrupts the nucleolus late in infection, which is temporally controlled by host mitochondria.); EspFu/tccP (Inducing degradation of the aniapoptic protein AbcF2, tight junction disruption, microvilli effacement and elongation, mitochondrial dysfunction, N-WASP activation, SGLT-1 inactivation, pedestal maturation, inhibition of NHE3 activity, membrane remodelling; targets and disrupts the nucleolus late in infection, which is temporally controlled by host mitochondria.); EspG (TBC-like GTPase activating protein. Efficiently catalyzes GTP hydrolysis in Rab1 to disrupt of Rab1-mediated ER-to-Golgi

trafficking.); EspH (First bacterial effector acting directly on RhoGEFs, EspH directly binds to the DH-PH domain in RhoGEFs to disrupt RhoGEF-Rho signaling; critical for inhibiting macrophage phagocytosis.); EspJ (Inhibit both IgG- and complement receptor-mediated phagocytosis.); EspK; EspL1; EspL2 (Cysteine protease. Bounds F-actin-aggregating annexin 2 directly to increase annexin 2's ability to aggregate Tir-induced F-actin; block necroptosis and in flammation.); EspL4; EspM1 (GEF. Activates the RhoA signaling pathway and induce the formation of stress fibres; inhibit pedestal formation and induce tight junction mislocalization.); EspM2 (GEF. Activates the RhoA signaling pathway and induce the formation of stress fibres; inhibit pedestal formation and induce tight junction mislocalization.); EspN; EspO1-1; EspO1-2; EspR1; EspR3; EspR4; EspT (GEF. Activates Rac1 and Cdc42 leading

to formation of membrane ruffles and lamellipodia; induces membrane ruffles to facilitate bacterial invasion into non-phagocytic cells in a process involving Rac1 and Wave2.); EspW; EspX1; EspX2; EspX4; EspX5; EspX6; EspX7/nleL (E3 ubiquitin ligase, HECT-like. Modulates pedestal formation.); EspY1; EspY2; EspY3; EspY4; EspY5; Map (GEF. Mimics the host Dbl and catalyses the exchange of GDP for GTP in Cdc42, involved in effacement, SGLT1 inhibition, formation of filopodia and disruption of mitochondrial function.); NleA/espI (Disruption of tight junctions by inhibition of host cell protein trafficking through COPII-dependent pathways.); NleB1 (Blocks translocation of the p65 and to the host cell nucleus to inhibit NF-<kappa>B pathway, but NleE and NleB act at different points in the NF-<kappa>B signaling pathway.); NleB2 (May also have anti-inflammatory activity.); NleC (Metalloprotease.

Zn-dependent endopeptidases that specifically clip and inactivate RelA (p65), thus blocking NF-<kappa>B pathway.); NleD (Metalloprotease. Zn-dependent endopeptidases that specifically clip and inactivate JNK and p38, thus blocking AP-1 pathway.); NleE (PMN tran-epithelial migration; blocks translocation of the p65 to the host cell nucleus by preventing I<kappa>B degradation to inhibit NF-<kappa>B pathway.); NleF; NleG-1; NleG2-2; NleG2-3; NleG2-4; NleG5-1; NleG5-2; NleG6-1; NleG6-2; NleG6-3; NleG7 (U-box type E3 ubiquitin ligases.); NleG8-2; NleH1 (Ser/Thr protein kinase. Binds directly to a subunit of NF-<kappa>B, the ribosomal protein S3 (RPS3), reducing the nuclear abundance of RPS3 to dampen host transcriptional outputs; interact with Bax inhibitor-1 to block apoptosis.); NleH2 (Putative kinase. Attenuates NF-<kappa>B pathway.); SepZ/espZ (EspZ interacts with CD98 in host cell membranes to promote host

ECs_2155	nleG6-2	T3SS secreted effector NleG	VFG035025 (gb|WP_001443810)	TTSS secreted effectors (VF1110)	cell survival, therefore provide the pathogen with valuable time to colonize efficiently prior to dissemination.); TccP2; Tir (Mimics host immunoreceptor tyrosine-based inhibition motifs (ITIMs), also see helicobacter CagA. EHEC Tir lacks the Nck binding site. Conserved NPY (Asn-Pro-Tyr) motif recruits the adaptor protein IRTKS and/or IRSp53. IRTKS/IRSp53 link Tir and TccP/EspFu, which in turn activates N-WASP; Receptor for intimin; effacement; SGLT1 inhibition; recruits SHIP2 to control actin-pedestal morphology; maintains the integrity of the epithelium by keeping the destructive activity of EspG and EspG2 in check.) Cif (Deamidase. Induces cytopathic effects of actin stress fiber formation and cell cycle arrest.); EspB (Pore formation, actin disruption, microvilli effacement, anti-phagocytosis.); EspF (Inducing degradation of the aniapoptic protein AbcF2, tight junction disruption,	setA	Escherichia coli O157:H7 str. EDL933	Effector delivery system	nleG6-1	DUF1076 domain-containing protein	96.2	1.38E-145	403

microvilli effacement and elongation, mitochondrial dysfunction, N-WASP activation, SGLT-1 inactivation, pedestal maturation, inhibition of NHE3 activity, membrane remodelling; targets and disrupts the nucleolus late in infection, which is temporally controlled by host mitochondria.); EspFu/tccP (Inducing degradation of the aniapoptic protein AbcF2, tight junction disruption, microvilli effacement and elongation, mitochondrial dysfunction, N-WASP activation, SGLT-1 inactivation, pedestal maturation, inhibition of NHE3 activity, membrane remodelling; targets and disrupts the nucleolus late in infection, which is temporally controlled by host mitochondria.); EspG (TBC-like GTPase activating protein. Efficiently catalyzes GTP hydrolysis in Rab1 to disrupt of Rab1-mediated ER-to-Golgi trafficking.); EspH (First bacterial effector acting directly on RhoGEFs, EspH directly binds to the DH-PH domain

in RhoGEFs to disrupt RhoGEF-Rho signaling; critical for inhibiting macrophage phagocytosis.); EspJ (Inhibit both IgG- and complement receptor-mediated phagocytosis.); EspK; EspL1; EspL2 (Cysteine protease. Bounds F-actin-aggregating annexin 2 directly to increase annexin 2's ability to aggregate Tir-induced F-actin; block necroptosis and in flammation.); EspL4; EspM1 (GEF. Activates the RhoA signaling pathway and induce the formation of stress fibres; inhibit pedestal formation and induce tight junction mislocalization.); EspM2 (GEF. Activates the RhoA signaling pathway and induce the formation of stress fibres; inhibit pedestal formation and induce tight junction mislocalization.); EspN; EspO1-1; EspO1-2; EspR1; EspR3; EspR4; EspT (GEF. Activates Rac1 and Cdc42 leading to formation of membrane ruffles and lamellipodia; induces membrane ruffles to facilitate bacterial

invasion into non-phagocytic cells in a process involving Rac1 and Wave2.); EspW; EspX1; EspX2; EspX4; EspX5; EspX6; EspX7/nleL (E3 ubiquitin ligase, HECT-like. Modulates pedestal formation.); EspY1; EspY2; EspY3; EspY4; EspY5; Map (GEF. Mimics the host Dbl and catalyses the exchange of GDP for GTP in Cdc42, involved in effacement, SGLT1 inhibition, formation of filopodia and disruption of mitochondrial function.); NleA/espI (Disruption of tight junctions by inhibition of host cell protein trafficking through COPII-dependent pathways.); NleB1 (Blocks translocation of the p65 and to the host cell nucleus to inhibit NF-<kappa>B pathway, but NleE and NleB act at different points in the NF-<kappa>B signaling pathway.); NleB2 (May also have anti-inflammatory activity.); NleC (Metalloprotease. Zn-dependent endopeptidases that specifically clip and inactivate RelA (p65), thus blocking NF-<kappa>B

pathway.); NleD (Metalloprotease. Zn-dependent endopeptidases that specifically clip and inactivate JNK and p38, thus blocking AP-1 pathway.); NleE (PMN tran-epithelial migration; blocks translocation of the p65 to the host cell nucleus by preventing I<kappa>B degradation to inhibit NF-<kappa>B pathway.); NleF; NleG-1; NleG2-2; NleG2-3; NleG2-4; NleG5-1; NleG5-2; NleG6-1; NleG6-2; NleG6-3; NleG7 (U-box type E3 ubiquitin ligases.); NleG8-2; NleH1 (Ser/Thr protein kinase. Binds directly to a subunit of NF-<kappa>B, the ribosomal protein S3 (RPS3), reducing the nuclear abundance of RPS3 to dampen host transcriptional outputs; interact with Bax inhibitor-1 to block apoptosis.); NleH2 (Putative kinase. Attenuates NF-<kappa>B pathway.); SepZ/espZ (EspZ interacts with CD98 in host cell membranes to promote host cell survival, therefore provide the pathogen with valuable time to colonize efficiently prior to

ECs_1994	nleG2-2	T3SS secreted effector NleG	VFG034985 (gb|WP_001131659)	TTSS secreted effectors (VF1110)	dissemination.); TccP2; Tir (Mimics host immunoreceptor tyrosine-based inhibition motifs (ITIMs), also see helicobacter CagA. EHEC Tir lacks the Nck binding site. Conserved NPY (Asn-Pro-Tyr) motif recruits the adaptor protein IRTKS and/or IRSp53. IRTKS/IRSp53 link Tir and TccP/EspFu, which in turn activates N-WASP; Receptor for intimin; effacement; SGLT1 inhibition; recruits SHIP2 to control actin-pedestal morphology; maintains the integrity of the epithelium by keeping the destructive activity of EspG and EspG2 in check.) Cif (Deamidase. Induces cytopathic effects of actin stress fiber formation and cell cycle arrest.); EspB (Pore formation, actin disruption, microvilli effacement, anti-phagocytosis.); EspF (Inducing degradation of the aniapoptic protein AbcF2, tight junction disruption, microvilli effacement and elongation, mitochondrial dysfunction, N-WASP activation, SGLT-1 inactivation,	setA	Escherichia coli O157:H7 str. EDL933	Effector delivery system	nleG2-2	DUF1076 domain-containing protein	100	2.34E-135	376

pedestal maturation, inhibition of NHE3 activity, membrane remodelling; targets and disrupts the nucleolus late in infection, which is temporally controlled by host mitochondria.); EspFu/tccP (Inducing degradation of the aniapoptic protein AbcF2, tight junction disruption, microvilli effacement and elongation, mitochondrial dysfunction, N-WASP activation, SGLT-1 inactivation, pedestal maturation, inhibition of NHE3 activity, membrane remodelling; targets and disrupts the nucleolus late in infection, which is temporally controlled by host mitochondria.); EspG (TBC-like GTPase activating protein. Efficiently catalyzes GTP hydrolysis in Rab1 to disrupt of Rab1-mediated ER-to-Golgi trafficking.); EspH (First bacterial effector acting directly on RhoGEFs, EspH directly binds to the DH-PH domain in RhoGEFs to disrupt RhoGEF-Rho signaling; critical for inhibiting macrophage phagocytosis.); EspJ

(Inhibit both IgG- and complement receptor-mediated phagocytosis.); EspK; EspL1; EspL2 (Cysteine protease. Bounds F-actin-aggregating annexin 2 directly to increase annexin 2's ability to aggregate Tir-induced F-actin; block necroptosis and in flammation.); EspL4; EspM1 (GEF. Activates the RhoA signaling pathway and induce the formation of stress fibres; inhibit pedestal formation and induce tight junction mislocalization.); EspM2 (GEF. Activates the RhoA signaling pathway and induce the formation of stress fibres; inhibit pedestal formation and induce tight junction mislocalization.); EspN; EspO1-1; EspO1-2; EspR1; EspR3; EspR4; EspT (GEF. Activates Rac1 and Cdc42 leading to formation of membrane ruffles and lamellipodia; induces membrane ruffles to facilitate bacterial invasion into non-phagocytic cells in a process involving Rac1 and Wave2.); EspW; EspX1; EspX2; EspX4; EspX5;

EspX6; EspX7/nleL (E3 ubiquitin ligase, HECT-like. Modulates pedestal formation.); EspY1; EspY2; EspY3; EspY4; EspY5; Map (GEF. Mimics the host Dbl and catalyses the exchange of GDP for GTP in Cdc42, involved in effacement, SGLT1 inhibition, formation of filopodia and disruption of mitochondrial function.); NleA/espI (Disruption of tight junctions by inhibition of host cell protein trafficking through COPII-dependent pathways.); NleB1 (Blocks translocation of the p65 and to the host cell nucleus to inhibit NF-<kappa>B pathway, but NleE and NleB act at different points in the NF-<kappa>B signaling pathway.); NleB2 (May also have anti-inflammatory activity.); NleC (Metalloprotease. Zn-dependent endopeptidases that specifically clip and inactivate RelA (p65), thus blocking NF-<kappa>B pathway.); NleD (Metalloprotease. Zn-dependent endopeptidases that specifically clip and inactivate JNK

and p38, thus blocking AP-1 pathway.); NleE (PMN tran-epithelial migration; blocks translocation of the p65 to the host cell nucleus by preventing I<kappa>B degradation to inhibit NF-<kappa>B pathway.); NleF; NleG-1; NleG2-2; NleG2-3; NleG2-4; NleG5-1; NleG5-2; NleG6-1; NleG6-2; NleG6-3; NleG7 (U-box type E3 ubiquitin ligases.); NleG8-2; NleH1 (Ser/Thr protein kinase. Binds directly to a subunit of NF-<kappa>B, the ribosomal protein S3 (RPS3), reducing the nuclear abundance of RPS3 to dampen host transcriptional outputs; interact with Bax inhibitor-1 to block apoptosis.); NleH2 (Putative kinase. Attenuates NF-<kappa>B pathway.); SepZ/espZ (EspZ interacts with CD98 in host cell membranes to promote host cell survival, therefore provide the pathogen with valuable time to colonize efficiently prior to dissemination.); TccP2; Tir (Mimics host immunoreceptor tyrosine-based inhibition motifs (ITIMs), also see

ECs_2156	nleG2-3	T3SS secreted effector NleG	VFG034985 (gb|WP_001131659)	TTSS secreted effectors (VF1110)	helicobacter CagA. EHEC Tir lacks the Nck binding site. Conserved NPY (Asn-Pro-Tyr) motif recruits the adaptor protein IRTKS and/or IRSp53. IRTKS/IRSp53 link Tir and TccP/EspFu, which in turn activates N-WASP; Receptor for intimin; effacement; SGLT1 inhibition; recruits SHIP2 to control actin-pedestal morphology; maintains the integrity of the epithelium by keeping the destructive activity of EspG and EspG2 in check.) Cif (Deamidase. Induces cytopathic effects of actin stress fiber formation and cell cycle arrest.); EspB (Pore formation, actin disruption, microvilli effacement, anti-phagocytosis.); EspF (Inducing degradation of the aniapoptic protein AbcF2, tight junction disruption, microvilli effacement and elongation, mitochondrial dysfunction, N-WASP activation, SGLT-1 inactivation, pedestal maturation, inhibition of NHE3 activity, membrane remodelling; targets and disrupts the nucleolus	setA	Escherichia coli O157:H7 str. EDL933	Effector delivery system	nleG2-2	DUF1076 domain-containing protein	99.5	3.32E-135	375

late in infection, which is temporally controlled by host mitochondria.); EspFu/tccP (Inducing degradation of the aniapoptic protein AbcF2, tight junction disruption, microvilli effacement and elongation, mitochondrial dysfunction, N-WASP activation, SGLT-1 inactivation, pedestal maturation, inhibition of NHE3 activity, membrane remodelling; targets and disrupts the nucleolus late in infection, which is temporally controlled by host mitochondria.); EspG (TBC-like GTPase activating protein. Efficiently catalyzes GTP hydrolysis in Rab1 to disrupt of Rab1-mediated ER-to-Golgi trafficking.); EspH (First bacterial effector acting directly on RhoGEFs, EspH directly binds to the DH-PH domain in RhoGEFs to disrupt RhoGEF-Rho signaling; critical for inhibiting macrophage phagocytosis.); EspJ (Inhibit both IgG- and complement receptor-mediated phagocytosis.); EspK; EspL1; EspL2 (Cysteine protease.

Bounds F-actin-aggregating annexin 2 directly to increase annexin 2's ability to aggregate Tir-induced F-actin; block necroptosis and in flammation.); EspL4; EspM1 (GEF. Activates the RhoA signaling pathway and induce the formation of stress fibres; inhibit pedestal formation and induce tight junction mislocalization.); EspM2 (GEF. Activates the RhoA signaling pathway and induce the formation of stress fibres; inhibit pedestal formation and induce tight junction mislocalization.); EspN; EspO1-1; EspO1-2; EspR1; EspR3; EspR4; EspT (GEF. Activates Rac1 and Cdc42 leading to formation of membrane ruffles and lamellipodia; induces membrane ruffles to facilitate bacterial invasion into non-phagocytic cells in a process involving Rac1 and Wave2.); EspW; EspX1; EspX2; EspX4; EspX5; EspX6; EspX7/nleL (E3 ubiquitin ligase, HECT-like. Modulates pedestal formation.); EspY1; EspY2; EspY3;

EspY4; EspY5; Map (GEF. Mimics the host Dbl and catalyses the exchange of GDP for GTP in Cdc42, involved in effacement, SGLT1 inhibition, formation of filopodia and disruption of mitochondrial function.); NleA/espI (Disruption of tight junctions by inhibition of host cell protein trafficking through COPII-dependent pathways.); NleB1 (Blocks translocation of the p65 and to the host cell nucleus to inhibit NF-<kappa>B pathway, but NleE and NleB act at different points in the NF-<kappa>B signaling pathway.); NleB2 (May also have anti-inflammatory activity.); NleC (Metalloprotease. Zn-dependent endopeptidases that specifically clip and inactivate RelA (p65), thus blocking NF-<kappa>B pathway.); NleD (Metalloprotease. Zn-dependent endopeptidases that specifically clip and inactivate JNK and p38, thus blocking AP-1 pathway.); NleE (PMN tran-epithelial migration; blocks translocation of the p65 to the

host cell nucleus by preventing I<kappa>B degradation to inhibit NF-<kappa>B pathway.); NleF; NleG-1; NleG2-2; NleG2-3; NleG2-4; NleG5-1; NleG5-2; NleG6-1; NleG6-2; NleG6-3; NleG7 (U-box type E3 ubiquitin ligases.); NleG8-2; NleH1 (Ser/Thr protein kinase. Binds directly to a subunit of NF-<kappa>B, the ribosomal protein S3 (RPS3), reducing the nuclear abundance of RPS3 to dampen host transcriptional outputs; interact with Bax inhibitor-1 to block apoptosis.); NleH2 (Putative kinase. Attenuates NF-<kappa>B pathway.); SepZ/espZ (EspZ interacts with CD98 in host cell membranes to promote host cell survival, therefore provide the pathogen with valuable time to colonize efficiently prior to dissemination.); TccP2; Tir (Mimics host immunoreceptor tyrosine-based inhibition motifs (ITIMs), also see helicobacter CagA. EHEC Tir lacks the Nck binding site. Conserved NPY (Asn-Pro-Tyr) motif recruits the

ECs_0025	espX1	T3SS effector-like protein EspX	VFG034849 (gb|WP_000129338)	TTSS secreted effectors (VF1110)	adaptor protein IRTKS and/or IRSp53. IRTKS/IRSp53 link Tir and TccP/EspFu, which in turn activates N-WASP; Receptor for intimin; effacement; SGLT1 inhibition; recruits SHIP2 to control actin-pedestal morphology; maintains the integrity of the epithelium by keeping the destructive activity of EspG and EspG2 in check.) Cif (Deamidase. Induces cytopathic effects of actin stress fiber formation and cell cycle arrest.); EspB (Pore formation, actin disruption, microvilli effacement, anti-phagocytosis.); EspF (Inducing degradation of the aniapoptic protein AbcF2, tight junction disruption, microvilli effacement and elongation, mitochondrial dysfunction, N-WASP activation, SGLT-1 inactivation, pedestal maturation, inhibition of NHE3 activity, membrane remodelling; targets and disrupts the nucleolus late in infection, which is temporally controlled by host mitochondria.); EspFu/tccP (Inducing degradation of	setA	Escherichia coli O157:H7 str. EDL933	Effector delivery system	espX1	Type III secretion system effector EspX1	100	4.7700000000 2e-313	849

the aniapoptic protein AbcF2, tight junction disruption, microvilli effacement and elongation, mitochondrial dysfunction, N-WASP activation, SGLT-1 inactivation, pedestal maturation, inhibition of NHE3 activity, membrane remodelling; targets and disrupts the nucleolus late in infection, which is temporally controlled by host mitochondria.); EspG (TBC-like GTPase activating protein. Efficiently catalyzes GTP hydrolysis in Rab1 to disrupt of Rab1-mediated ER-to-Golgi trafficking.); EspH (First bacterial effector acting directly on RhoGEFs, EspH directly binds to the DH-PH domain in RhoGEFs to disrupt RhoGEF-Rho signaling; critical for inhibiting macrophage phagocytosis.); EspJ (Inhibit both IgG- and complement receptor-mediated phagocytosis.); EspK; EspL1; EspL2 (Cysteine protease. Bounds F-actin-aggregating annexin 2 directly to increase annexin 2's ability to aggregate Tir-induced

F-actin; block necroptosis and inflammation.); EspL4; EspM1 (GEF. Activates the RhoA signaling pathway and induce the formation of stress fibres; inhibit pedestal formation and induce tight junction mislocalization.); EspM2 (GEF. Activates the RhoA signaling pathway and induce the formation of stress fibres; inhibit pedestal formation and induce tight junction mislocalization.); EspN; EspO1-1; EspO1-2; EspR1; EspR3; EspR4; EspT (GEF. Activates Rac1 and Cdc42 leading to formation of membrane ruffles and lamellipodia; induces membrane ruffles to facilitate bacterial invasion into non-phagocytic cells in a process involving Rac1 and Wave2.); EspW; EspX1; EspX2; EspX4; EspX5; EspX6; EspX7/nleL (E3 ubiquitin ligase, HECT-like. Modulates pedestal formation.); EspY1; EspY2; EspY3; EspY4; EspY5; Map (GEF. Mimics the host Dbl and catalyses the exchange of GDP for GTP in Cdc42, involved in

effacement, SGLT1 inhibition, formation of filopodia and disruption of mitochondrial function.); NleA/espI (Disruption of tight junctions by inhibition of host cell protein trafficking through COPII-dependent pathways.); NleB1 (Blocks translocation of the p65 and to the host cell nucleus to inhibit NF-<kappa>B pathway, but NleE and NleB act at different points in the NF-<kappa>B signaling pathway.); NleB2 (May also have anti-inflammatory activity.); NleC (Metalloprotease. Zn-dependent endopeptidases that specifically clip and inactivate RelA (p65), thus blocking NF-<kappa>B pathway.); NleD (Metalloprotease. Zn-dependent endopeptidases that specifically clip and inactivate JNK and p38, thus blocking AP-1 pathway.); NleE (PMN tran-epithelial migration; blocks translocation of the p65 to the host cell nucleus by preventing I<kappa>B degradation to inhibit NF-<kappa>B pathway.); NleF; NleG-1;

NleG2-2; NleG2-3; NleG2-4; NleG5-1; NleG5-2; NleG6-1; NleG6-2; NleG6-3; NleG7 (U-box type E3 ubiquitin ligases.); NleG8-2; NleH1 (Ser/Thr protein kinase. Binds directly to a subunit of NF-<kappa>B, the ribosomal protein S3 (RPS3), reducing the nuclear abundance of RPS3 to dampen host transcriptional outputs; interact with Bax inhibitor-1 to block apoptosis.); NleH2 (Putative kinase. Attenuates NF-<kappa>B pathway.); SepZ/espZ (EspZ interacts with CD98 in host cell membranes to promote host cell survival, therefore provide the pathogen with valuable time to colonize efficiently prior to dissemination.); TccP2; Tir (Mimics host immunoreceptor tyrosine-based inhibition motifs (ITIMs), also see helicobacter CagA. EHEC Tir lacks the Nck binding site. Conserved NPY (Asn-Pro-Tyr) motif recruits the adaptor protein IRTKS and/or IRSp53. IRTKS/IRSp53 link Tir and TccP/EspFu, which in turn activates N-WASP;

					Receptor for intimin; effacement; SGLT1 inhibition; recruits SHIP2 to control actin-pedestal morphology; maintains the integrity of the epithelium by keeping the destructive activity of EspG and EspG2 in check.)								
ECs_0156	fhuD	iron(3+)-hydroxamate import ABC transporter periplasmic binding protein	VFG044209 (gb|WP_000722819)	Petrobactin (VF0584)	Required for virulence in murine models of inhalational anthrax;Protects against oxidative stress and improve sporulation	setB	Bacillus anthracis str. Ames Ancestor	Nutritional/Metabolic factor	GBAA_RS23155	iron-siderophore ABC transporter substrate-binding protein	23.9	2.36E-18	83.6
ECs_0157	fhuB	iron(3+)-hydroxamate import ABC transporter permease	VFG044210 (gb|WP_001061683)	Petrobactin (VF0584)	Required for virulence in murine models of inhalational anthrax;Protects against oxidative stress and improve sporulation	setA	Bacillus anthracis str. Ames Ancestor	Nutritional/Metabolic factor	fhuB	Fe(3+)-hydroxamate ABC transporter permease FhuB	31.7	1.50E-84	279
ECs_3914	fitD	permease of ferrichro	VFG044210 (gb|WP_001061683)	Petrobactin (VF0584)	Required for virulence in murine models of inhalational anthrax;Protects against oxidative	setA	Bacillus anthracis str. Ames	Nutritional/Metabolic factor	fhuB	Fe(3+)-hydroxamate ABC	38.1	1.28E-48	173

		me ABC transporter			stress and improve sporulation		Ancestor			transporter permease FhuB			
ECs_4210	argD	acetylornithine delta-aminotransferase	VFG015824 (gb|WP_012273379)	Pyoverdine (VF0094)	Effective at acquiring iron from transferrin and lactoferrin; cytotoxic due to its ability to stimulating the production of reactive oxygen species	setA	Pseudomonas putida GB-1	Nutritional/Metabolic factor	pvdH	diaminobutyrate-2-oxoglutarate aminotransferase PvdH	31.4	1.91E-49	173
ECs_3221	yfcU	outer membrane usher protein	VFG021563 (gb|WP_000951668)	Stf (VF0958)	-	setA	Salmonella enterica subsp. enterica serovar Agona str. SL483	Adherence	stfC	fimbrial biogenesis outer membrane usher protein	57.3	0	1075
ECs_3373	der	ribosome-associated GTPase	VFG047396 (gb|WP_003014101)	FupA (VF0550)	High-affinity iron transport Protein involved in ferrous iron acquisition	setA	Francisella tularensis subsp. holarctica FTNF002-00	Nutritional/Metabolic factor	feoB	Fe(2+) transporter permease subunit FeoB	28	2.51E-06	50.1
ECs_3913	fitE	ferrichrome-binding protein	VFG013752 (gb|WP_011015613)	ABC transporter (VF0741)	-	setB	Corynebacterium glutamicum ATCC 13032 (DSM 20300)	Nutritional/Metabolic factor	fagD	iron-siderophore ABC transporter substrate-	25.6	4.38E-12	65.9

										binding protein			
ECs_3915	fitC	permease of ferrichrome ABC transporter	VFG013743 (gb|WP_005325331)	ABC transporter (VF0741)	-	setA	Corynebacterium jeikeium K411	Nutritional/Metabolic factor	fagA	iron ABC transporter permease	43.4	1.89E-61	200
ECs_3916	fepC_2	ABC-type iron-siderophore transport system ATP-binding protein	VFG007238 (gb|WP_000379797)	ViuPDGC system (VF0627)	-	setA	Vibrio cholerae O1 biovar El Tor str. N16961	Nutritional/Metabolic factor	viuC	ABC transporter ATP-binding protein	49.2	2.58E-88	263
ECs_5275	fimC	periplasmic chaperone	VFG012292 (gb|WP_000066545)	Type 1 fimbriae (VF0221)	Makes an important contribution to colonization of the bladder	setA	Escherichia coli O157:H7 str. EDL933	Adherence	fimC	Chaperone protein fimC precursor	99.6	1.17E-171	472
ECs_4792	glnA	glutamine synthetase	VFG009407 (gb|WP_011893806)	Glutamine synthesis (VF0816)	-	setB	Mycobacterium gilvum PYR-GCK	Nutritional/Metabolic factor	glnA1	type I glutamate--ammonia ligase	50.2	3.78E-168	482
ECs_0714	fur	ferric iron	VFG000478 (gb|NP_45	Fur (VF0113)	Repress the expression of iron-regulated genes; also required	setA	Salmonella enterica	Regulation	fur	ferric iron uptake	99.3	4.49E-108	303

		uptake regulon transcriptional repressor	9678)		for acid-induced activation of atr genes		subsp. enterica serovar Typhimurium str. LT2			transcriptional regulator			
ECs_2311	ydgI	arginine/ornithine antiporter transporter	VFG047663 (gb|WP_014715113)	GadC (VF0555)	Glutamate permease GadC helps the bacterium to resist the oxidative stress generated by the NADPH oxidase. This transporter is critically required for proper bacterial phagosomal escape	setB	Francisella noatunensis subsp. orientalis str. Toba 04	Nutritional/Metabolic factor	gadC	glutamate:gamma-aminobutyric acid antiporter family protein	22	7.89E-07	51.2
ECs_2319	manA	mannose-6-phosphate isomerase	VFG013187 (gb|WP_012341760)	Exopolysaccharide (VF0755)	-	setB	Haemophilus somnus 2336	Immune modulation	manA	mannose-6-phosphate isomerase, class I	36	3.17E-62	205
ECs_2604	otsA	trehalose-6-phosphate synthase	VFG042019 (gb|WP_011004029)	T3SS (VF1295)	-	setB	Ralstonia solanacearum GMI1000	Effector delivery system	otsA	alpha,alpha-trehalose-phosphate synthase (UDP-forming)	39.6	6.71E-104	320
ECs_3733	eivG	type III secretion protein EivG	VFG042076 (gb|NP_311760)	ETT2 (VF1161)	-	setA	Escherichia coli O157:H7 str. Sakai	Effector delivery system	eivG	type III secretion protein EivG	100	0	1087

ECs_3363	ppk	polyphosphate kinase	VFG042931 (gb|WP_012031410)	Type IV pili (VF1212)	-	setB	Dichelobacter nodosus VCS1703A	Adherence	ppk1	polyphosphate kinase 1	34.5	7.40E-113	356
ECs_3361	purM	phosphoribosylaminoimidazole synthetase	VFG047558 (gb|WP_012279909)	Purine biosynthesis (VF0559)	Required for bacterial cytosolic replication	setA	Francisella philomiragia subsp. philomiragia ATCC 25017	Nutritional/Metabolic factor	purM	phosphoribosylaminoimidazole synthetase	45.1	4.37E-89	271
ECs_3369	guaA	glutamine aminotransferase	VFG047735 (gb|WP_014714142)	Pyrimidine biosynthesis (VF0558)	Mediating bacterial resistance to reactive oxygen species (ROS) that is important for phagosomal escape	setA	Francisella noatunensis subsp. orientalis str. Toba 04	Nutritional/Metabolic factor	carA	carbamoyl phosphate synthase small subunit	24	1.26E-09	60.1
ECs_0035	carA	carbamoyl phosphate synthetase small subunit	VFG047728 (gb|WP_014547361)	Pyrimidine biosynthesis (VF0558)	Mediating bacterial resistance to reactive oxygen species (ROS) that is important for phagosomal escape	setA	Francisella cf. tularensis subsp. novicida 3523	Nutritional/Metabolic factor	carA	carbamoyl phosphate synthase small subunit	49.2	2.95E-125	366
ECs_4560	cesT	T3SS chaperone CesT	VFG000804 (gb|WP_000098793)	TTSS (VF0191)	Injects Tir and other effector molecules directly into the host cell. Effector molecules activate	setA	Escherichia coli O157:H7	Effector delivery system	cesT	multieffector chaperone	100	2.04E-112	315

					cell-signaling pathways, causing alterations in the host cell cytoskeleton and resulting in the depolymerization of actin and the loss of microvilli		str. EDL933					
ECs_4462	yibH	membrane fusion protein component of efflux pump	VFG036945 (gb|WP_015815890)	FarAB (VF0450)	Mediates the resistance to antimicrobial long-chain fatty acids	setA	Neisseria meningitidis alpha14	Antimicrobial activity/Competitive advantage	farA	fatty acid efflux system protein FarA	25.6	2.74E-07	52
ECs_1035	pqiB	paraquat-inducible protein B	VFG045345 (gb|WP_005457104)	MAM7 (VF0512)	Mediating attachment for the bacterium to a host cells	setA	Vibrio parahaemolyticus RIMD 2210633	Adherence	mam7	multivalent adhesion molecule MAM7	30.4	1.31E-44	169
ECs_2544	yebT	hypothetical protein	VFG045345 (gb|WP_005457104)	MAM7 (VF0512)	Mediating attachment for the bacterium to a host cells	setA	Vibrio parahaemolyticus RIMD 2210633	Adherence	mam7	multivalent adhesion molecule MAM7	35.6	1.65E-188	563
ECs_1452	flgC	flagellar component of cell-proximal portion of	VFG043075 (gb|WP_001196460)	Peritrichous flagella (VF1154)	-	setA	Escherichia coli O157:H7 str. EDL933	Motility	flgC	flagellar basal body rod protein FlgC	100	4.89E-89	254

		basal-body rod											
ECs_1321	ureD	urease accessory protein	VFG006499 (gb|WP_001099421)	Urease (VF0050)	An important colonization factor, contributes to acid resistance, epithelial cell damage, chemotactic behavior, and nitrogen metabolism	setA	Helicobacter pylori HPAG1	Stress survival	ureH	urease accessory protein (ureH)	25.7	4.08E-12	63.9
ECs_1322	ureA	urease subunit gamma	VFG006471 (gb|WP_011115249)	Urease (VF0050)	An important colonization factor, contributes to acid resistance, epithelial cell damage, chemotactic behavior, and nitrogen metabolism	setA	Helicobacter hepaticus ATCC 51449	Stress survival	ureA	urease alpha subunit UreA	60	3.50E-34	117
ECs_1323	ureB	urease subunit beta	VFG006470 (gb|WP_011578335)	Urease (VF0050)	An important colonization factor, contributes to acid resistance, epithelial cell damage, chemotactic behavior, and nitrogen metabolism	setA	Helicobacter acinonychis str. Sheeba	Stress survival	ureA	urease alpha subunit UreA	53.8	3.35E-30	107
ECs_1324	ureC	urease subunit alpha	VFG006476 (gb|WP_011115250)	Urease (VF0050)	An important colonization factor, contributes to acid resistance, epithelial cell damage, chemotactic behavior, and nitrogen metabolism	setA	Helicobacter hepaticus ATCC 51449	Stress survival	ureB	urease beta subunit UreB, urea amidohydrolase	62.6	4.79E-263	730
ECs_1458	flgI	flagellar basal body protein	VFG043081 (gb|WP_000589319)	Peritrichous flagella (VF1154)	-	setA	Escherichia coli O157:H7 str. EDL933	Motility	flgI	flagellar basal body P-ring protein FlgI	100	2.82E-248	676
ECs_1459	flgJ	flagellar	VFG043082	Peritrich	-	setA	Escherichi	Motility	flgJ	flagellar	100	1.11E-	604

		rod assembly protein and murein hydrolase	(gb|WP_001301817)	ous flagella (VF1154)			a coli O157:H7 str. EDL933			assembly peptidoglycan hydrolase FlgJ		221	
ECs_0323	ecpA	ECP pilin	VFG002414 (gb|WP_000730972)	ECP (VF0404)	ECP, composed of a 21-kDa pilin subunit EspA, is a pilus-adherence factor that is crucial to the virulence of E. coli O157 in humans, and is also carried by commensal strains of E. coli.; It is suggested that pathogenic E. coli strains may use ECP to mimic commensal E. coli and provide themselves with an ecological advantage for host colonization and evasion of the immune system.	setA	Escherichia coli O157:H7 str. EDL933	Adherence	yagZ/ecpA	E. coli common pilus structural subunit EcpA	100	1.97E-129	361
ECs_0322	ecpB	ECP production pilus chaperone	VFG002412 (gb|WP_000716386)	ECP (VF0404)	ECP, composed of a 21-kDa pilin subunit EspA, is a pilus-adherence factor that is crucial to the virulence of E. coli O157 in humans, and is also carried by commensal strains of E. coli.; It is suggested that pathogenic E. coli strains may use ECP to mimic commensal E. coli and provide themselves with an ecological advantage for host	setA	Escherichia coli O157:H7 str. EDL933	Adherence	yagY/ecpB	E. coli common pilus chaperone EcpB	100	1.38E-156	432

					colonization and evasion of the immune system.									
ECs_0321	ecpC	ECP production outer membrane protein	VFG002415 (gb|WP_001131063)	ECP (VF0404)	ECP, composed of a 21-kDa pilin subunit EspA, is a pilus-adherence factor that is crucial to the virulence of E. coli O157 in humans, and is also carried by commensal strains of E. coli.; It is suggested that pathogenic E. coli strains may use ECP to mimic commensal E. coli and provide themselves with an ecological advantage for host colonization and evasion of the immune system.	setA	Escherichia coli O157:H7 str. EDL933	Adherence	yagX/ecpC	E. coli common pilus usher EcpC	100	0	1644
ECs_0320	ecpD	polymerized tip adhesin of ECP fibers	VFG002416 (gb|WP_001265657)	ECP (VF0404)	ECP, composed of a 21-kDa pilin subunit EspA, is a pilus-adherence factor that is crucial to the virulence of E. coli O157 in humans, and is also carried by commensal strains of E. coli.; It is suggested that pathogenic E. coli strains may use ECP to mimic commensal E. coli and provide themselves with an ecological advantage for host colonization and evasion of the immune system.	setA	Escherichia coli O157:H7 str. EDL933	Adherence	yagW/ecpD	polymerized tip adhesin of ECP fibers	100	0	1103
ECs_0030	lspA	prolipoprotein	VFG006811 (gb|WP_00	Lsp (VF0351)	A lipoprotein-specific signal peptidase SPase II, responsible for	setA	Listeria monocytoge	Post-translational	lspA	signal peptidase	32	2.43E-20	82

		signal peptidase	3726602)		the maturation of lipoproteins in listerial pathogenesis; the deletion mutant failed to process several lipoproteins and the mutant bacteria are clearly impaired in phagosomal escape		nes serotype 4b str. F2365	modification		II			
ECs_0145	yadN	fimbrial protein	VFG004233 (gb|WP_000709193)	Sta (VF0953)	-	setB	Salmonella enterica subsp. enterica serovar Typhi str. CT18	Adherence	staA	fimbrial protein	43.6	2.51E-33	117
ECs_0143	htrE	outer membrane usher protein	VFG004230 (gb|WP_001125008)	Sta (VF0953)	-	setA	Salmonella enterica subsp. enterica serovar Typhi str. Ty2	Adherence	staC	outer membrane usher protein	64.6	0	1150
ECs_0142	yadM	fimbrial protein	VFG004227 (gb|WP_000827925)	Sta (VF0953)	-	setB	Salmonella enterica subsp. enterica serovar Typhi str.	Adherence	staD	fimbrial protein	49.7	2.01E-46	150

							CT18						
ECs_0141	yadL	fimbrial protein	VFG018339 (gb|WP_000745164)	Sta (VF0953)	-	setB	Salmonella enterica subsp. enterica serovar Paratyphi B str. SPB7	Adherence	staE	fimbrial protein StaE	52.9	5.85E-54	171
ECs_0140	yadK	fimbrial protein	VFG021415 (gb|WP_000957977)	Sta (VF0953)	-	setB	Salmonella enterica subsp. enterica serovar Agona str. SL483	Adherence	staF	fimbrial protein	47.3	3.40E-53	168
ECs_3142	arnC	undecaprenyl phosphate-L-Ara4FN transferase	VFG046913 (gb|WP_013922683)	LPS (VF0542)	A key virulence factor does not signal through, and is not an agonist of, toll like receptor 4 (TLR-4) and has little endotoxic activity	setA	Francisella sp. TX077308	Immune modulation	flmF2	glycosyl transferase family protein	33.6	1.70E-43	151
ECs_2600	motA	flagellar motor protein MotA	VFG043096 (gb|WP_000906325)	Peritrichous flagella (VF1154)	-	setA	Escherichia coli O157:H7 str. EDL933	Motility	motA	flagellar motor stator protein MotA	100	2.02E-199	546

ECs_4051	rimP	ribosome maturation factor for 30S subunits	VFG039487 (gb|WP_012569825)	T4SS secreted effectors (VF0696)	CBUA0020; CBU_0012*; CBU_0113; CBU_0122; CBU_0183; CBU_0201; CBU_0270; CBU_0295; CBU_0344*; CBU_0372; CBU_0375*; CBU_0469; CBU_0513; CBU_0534; CBU_0590; CBU_0635; CBU_0637; CBU_0820*; CBU_1048*; CBU_1079; CBU_1107*; CBU_1150*; CBU_1198; CBU_1268; CBU_1349; CBU_1370; CBU_1409; CBU_1434; CBU_1493; CBU_1495*; CBU_1525*; CBU_1530; CBU_1566; CBU_1576; CBU_1594; CBU_1607; CBU_1614; CBU_1639; CBU_1665; CBU_1677; CBU_1685; CBU_1752; CBU_1754; CBU_1789; CBU_1790; CBU_1794; CBU_1818; CBU_1819; CBU_1863; CBU_2016; CBU_2028; CBU_2056; CBU_2059*; CBU_2076; AnkA; AnkB; AnkF; AnkG (Interacts with host protein p32 to block apoptosis.); AnkH; AnkI; AnkM/cig58; AnkP; Cem1; Cem12; Cem13; Cem3; Cem4; Cem6; Cem9; CetCb1; CetCb2; CetCb3; CetCB4; CetCb5; CetCb6; CirA/coxCC1 (Phosphate transporter family protein.); CirB; CirC/coxDFB1;	setA	Coxiella burnetii CbuG_Q212	Effector delivery system	rimP	Coxiella Dot/Icm type IVB secretion system translocated effector	51	5.47E-43	139

					CoxCC10/cig49; CoxCC11; CoxCC12; CoxCC14; CoxCC15; CoxCC3; CoxCC4; CoxCC5; CoxCC6; CoxCC7/cig44; CoxCC8; CoxDFB3; CoxDFB4 (Surface antigen.); CoxDFB5/cig57; CoxDFB6; CoxFIC1; CoxH2/rimL (Acetyltransferase.); CoxH3; CoxH4/cig61; CoxK1 (Protein kinase, putative.); CoxK2; CoxTPR1 (Conserved domain protein.); CoxU1; CoxU2; CpeA; CpeB; CpeC/coxU3 (Hypothetical protein plasmid QpH1.); CpeD; CpeE; CpeF; CpeG; CpeH; CvpA; MceA; PhnB; CBUD_RS05145; CBUD_RS06720*; CBUD_RS08635; CBUD_RS11275; CBUD_RS12405; CBUG_RS02435; CBUK_RS06760							
ECs_5211	yjgA	ribosome-associated UPF0307 family protein	VFG045476 (gb|WP_010945783)	Dot/Icm T4SS secreted effectors (VF0798)	AnkB/legAU13/ceg27 (E3 Ubiquitin Ligase Activity, bounds Skp1, targets host protein parvin B.); AnkC/legA12 (Ankyrin repeat.); AnkD/legA15 (Ankyrin repeat.); AnkF/legA14/ceg31 (Ankyrin repeat.); AnkG/ankZ/legA7 (Ankyrin repeat.); AnkH/legA3/ankW (Ankyrin repeat, NF-<kappa>B inhibitor.); AnkI/legAS4 (Ankyrin repeat.); AnkJ/legA11 (Ankyrin	setA	Legionella pneumophila subsp. pneumophila str. Philadelphia 1	Effector delivery system	LPG_RS00105	ribosome-associated protein	41.2	4.25E-28	102

repeat.); AnkK/legA5 (Ankyrin repeat.); AnkN/ankX/legA8 (Phosphocholination of Rab1 and Rab35 to regulate their activity; modulation of endosomal trafficking.); AnkQ/legA10; AnkY/legA9 (Ankyrin repeat, STPK, Enhancer of autophagy.); Ceg10; Ceg14/sidL (Inhibition of host protein synthesis leading to activation of the NF-<kappa>B pathway.); Ceg15; Ceg17; Ceg18; Ceg19 (Vesicle trafficking.); Ceg23; Ceg25; Ceg28; Ceg29; Ceg3; Ceg30; Ceg32/sidI (Interacts with eEF1A to inhibit host protein synthesis.); Ceg33; Ceg34; Ceg4; Ceg5; Ceg7; Ceg8; Ceg9 (Vesicle trafficking.); CegC1 (Zinc metallophospholipase C. Zinc metallophospholipase C.); CegC2 (Ninein domain.); CegC3; CegC4; DrrA/sidM (Rab1-GEF and GDF (RabGDI displacement factor) activity responsible for Rab1 recruitment to LCV, C-terminal PI4P binding domain responsible for membrane binding;

N-terminal AMPylation activity.); LaiE (SidE paralog.); LegA1; LegA2; LegA6; LegA7; LegC1; LegC3/ppeA (Vesicle trafficking.); LegC4 (Coiled-coil.); LegC6 (Coiled-coil.); LegD1; LegD2; LegG2 (Ras GEF.); LegK1 (Eukaryotic-like Ser/Thr kinase activity, directly activates NF-<kappa>B pathway by phosphorylating the I<kappa>B family of inhibitors.); LegK2 (Ser/Thr kinase.); LegK3 (STPK.); LegL1 (Leucine-rich repeats.); LegL2 (Leucine-rich repeats.); LegL3 (Leucine-rich repeats.); LegL5 (Leucine-rich repeats.); LegL6 (Leucine-rich repeats.); LegL7 (Leucine-rich repeats.); LegLC4 (Leucine-rich repeats, coiled-coil.); LegLC8 (Leucine-rich repeats, coiled-coil.); LegN; LegP (Astacin protease.); LegS1; LegS2 (Putative Sphingosine-1-phosphate lyase 1 (SP-lyase).); LegT (Thaumatin domain.); LegU1 (E3 Ubiquitin Ligase, targets host chaperone protein

BAT3.); LegY; Lem1; Lem10; Lem11; Lem12; Lem14; Lem15; Lem16; Lem17; Lem19; Lem2; Lem20; Lem21; Lem22; Lem23; Lem24; Lem25; Lem26; Lem27; Lem28; Lem29; Lem3 (Dephosphoryl-cholinase relieving the AnkX-mediated modification on Rab1.); Lem4/smdA (PI4P-binding protein.); Lem5; Lem6; Lem7; Lem8; Lem9; LepA (Nonlytic release from protozoa.); LepB (Rab1 GAP, vesicle trafficking and bacterial egress.); Lgt2/legC8 (Glucosyltransferase, inhibits host protein systhesis by glucosylating mammalian elongation factor eEF1A at serine-53.); Lgt3/legC5 (Glucosyltransferase, inhibits host protein systhesis by glucosylating mammalian elongation factor eEF1A at serine-53.); LidA (Promotion of Rab1 recruitment and tethering of ER derived vesicles to the LCV; stabilization of Rab guanosine nucleotide complex.); LidL (EnhC paralogue.); LirA; LirB (Peptidyl-prolyl cis-trans isomerase

A (rotamase A).); Lpg0045; Lpg0081; Lpg0294; Lpg0365; Lpg0518; Lpg0634; Lpg0963; Lpg1148; Lpg1158; Lpg1273; Lpg1689; Lpg1717; Lpg1751; Lpg2160 (Associates with BAT3 independently of LegU1; LegU1 and Lpg2160 may function redundantly or in concert to modulate BAT3 activity during the course of infection.); Lpg2327; Lpg2407; Lpg2525 (F-box protein.); Lpg2527; Lpg2744; LpnE (Putative Beta-lactamase.); LubX/legU2 (E3 ubiquitin ligase, targets another effector protein SidH to proteasome-mediated protein degradation in the host cells; cell cycle modulation via Clk1.); MavA; MavB; MavC; MavE; MavF; MavG; MavH; MavI; MavJ; MavL; MavM; MavN; MavV; PieA/lirC; PieB/lirD; PieC/lirE; PieD/lirF; PieE; PieF; PieG/legG1 (Regulator of chromosome condensation RCC.); PpeB; PpgA (Regulator of chromosome condensation.); RalF (Arf-GEF; Arf1 recuitment to LCV.); RavE; RavF; RavG; RavH; RavI; RavJ;

RavK; RavL; RavM; RavN; RavO; RavP; RavQ; RavR; RavS; RavT; RavW; RavX; RavY; RavZ (Cysteine protease. Inhibits host autophagy by cleaving and deconjugating LC3-PE.); RvfA; SdbA (Contributes to sustained NF-<kappa>B activation.); SdbB (SidB paralog.); SdbC (SidB paralog.); SdcA (SidC paralog, anchors to PtdIns(4)P on LCVs.); SdeA/laiA (Adherence and/or uptake.); SdeB/laiB; SdeC/laiC; SdeD/laiF (SidE paralog.); SdhA (Maintenance of LCV integrity preventing cell death and type I interferon induction.); SdhB (Paralog of sidH, ANTH domain.); SdjA; SetA (Vesicle trafficking.); SidA; SidB (Rtx toxin, lipase.); SidC (ER recuitment.); SidD; SidE/laiD; SidF (Anti-apoptosis by targeting pro-death members of the Bcl2 protein family.); SidG (Coiled-coil.); SidH (A substrate of LubX E3 ubiquitin ligase.); SidJ (ER recuitment.); SidK (Interacting with VatA, a key component of the proton pump.

Inhibition of LCV acidification.);
VipA (Actin nucleator contributing to modulate organelle trafficking.);
VipD (Phospholipase A1, removes PI(3)P from early endosomes.); VipE; VipF (N-terminal acetyltransferase, GNAT family.); VpdA/vipD2 (VipD paralog, Acyl transferase/acyl hydrolase/lysophospholipase.);
VpdB/vipD3 (VipD paralog, phospholipase.); VpdC; WipA; WipB;
YlfA/legC7 (Vesicle trafficking.);
YlfB/legC2 (Vesicle trafficking.);
CegC4; MesI; LPG_RS00040;
LPG_RS00105; LPG_RS00150;
LPG_RS00200; LPG_RS00235;
LPG_RS00300; LPG_RS00665 (PI-3-phosphatase.); LPG_RS00825;
LPG_RS00870; LPG_RS00880;
LPG_RS00925; LPG_RS01290;
LPG_RS01305; LPG_RS01820;
LPG_RS01880; LPG_RS02025;
LPG_RS03550; LPG_RS03935;
LPG_RS04800; LPG_RS05365;
LPG_RS05490; LPG_RS05590;
LPG_RS05660; LPG_RS05710;

LPG_RS05830; LPG_RS07260;

LPG_RS07280; LPG_RS07435;

LPG_RS07905; LPG_RS08210;

LPG_RS08285; LPG_RS08320;

LPG_RS08345; LPG_RS08350;

LPG_RS08370; LPG_RS08445;

LPG_RS08450; LPG_RS08485;

LPG_RS08595; LPG_RS08775;

LPG_RS08895; LPG_RS09040;

LPG_RS09470; LPG_RS09565;

LPG_RS09650; LPG_RS09825;

LPG_RS09915; LPG_RS09965;

LPG_RS10290; LPG_RS10795;

LPG_RS10800; LPG_RS11170;

LPG_RS11255; LPG_RS11415;

LPG_RS11860; LPG_RS11920;

LPG_RS11940; LPG_RS11990;

LPG_RS12190; LPG_RS12265;

LPG_RS12310; LPG_RS12405;

LPG_RS12815; LPG_RS12820;

LPG_RS12830; LPG_RS12855;

LPG_RS12885; LPG_RS12900;

LPG_RS13265; LPG_RS13310;

LPG_RS13590; LPG_RS13860;

LPG_RS14265; LPG_RS14285;

LPG_RS14345; LPG_RS14500;

					LPG_RS14525; LPG_RS14550; LPG_RS14555; LPG_RS14570; LPG_RS14710; LPG_RS14840; LPG_RS15050; LPG_RS15170								
ECs_0226	ECs_0226	lipoprotein	VFG035635 (gb|WP_001080153)	EHS (VF1176)	-	setA	Escherichia coli O157:H7 str. EDL933	Effector delivery system	aec24	type VI secretion system lipoprotein TssJ	100	2.39E-122	342
ECs_0227	imp	type VI secretion system protein ImpI	VFG035661 (gb|WP_001113707)	EHS (VF1176)	-	setA	Escherichia coli O157:H7 str. EDL933	Effector delivery system	aec23	type VI secretion system-associated FHA domain protein TagH	100	1.93e-311	841
ECs_3140	ais	LPS core heptose(II)-phosphate phosphatase	VFG042525 (gb|AAC28313)	Adhesive factor/rabbit 1 (AF/R1) pili (VF1146)	-	setB	Escherichia coli str. RDEC-1	Adherence	afrS	transcriptional activator precursor AfrS	43.2	1.64E-40	136
ECs_0224	impK	type VI secretion system protein	VFG035584 (gb|WP_000343292)	EHS (VF1176)	-	setA	Escherichia coli O157:H7 str. EDL933	Effector delivery system	aec26	type IVB secretion system protein	100	2.49E-188	515

		ImpK								IcmH/DotU			
ECs_3145	arnT	4-amino-4-deoxy-L-arabinose transferase	VFG046926 (gb|WP_014549484)	LPS (VF0542)	A key virulence factor does not signal through, and is not an agonist of, toll like receptor 4 (TLR-4) and has little endotoxic activity	setA	Francisella cf. novicida Fx1	Immune modulation	flmK	glycosyltransferase family 39 protein	29.4	1.95E-35	140
ECs_0225	impJ	type VI secretion system protein ImpJ	VFG035610 (gb|WP_000246434)	EHS (VF1176)	-	setA	Escherichia coli O157:H7 str. EDL933	Effector delivery system	aec25	type VI secretion system baseplate subunit TssK	100	0	885
ECs_0222	ECs_0222	type VI secretion protein	VFG035532 (gb|WP_000088854)	EHS (VF1176)	-	setA	Escherichia coli O157:H7 str. EDL933	Effector delivery system	aec28	type VI secretion system-associated protein TagO	100	8.59E-177	485
ECs_0223	ECs_0223	ATP-dependent Clp proteinase	VFG035558 (gb|WP_000614374)	EHS (VF1176)	-	setA	Escherichia coli O157:H7 str. EDL933	Effector delivery system	aec27/clpV	type VI secretion system ATPase TssH	100	0	1751
ECs_2601	flhC	flagellar class II regulon transcrip	VFG043097 (gb|WP_001291603)	Peritrichous flagella (VF1154)	-	setA	Escherichia coli O157:H7 str. EDL933	Motility	flhC	flagellar transcriptional regulator	100	6.72E-140	387

		tional activator								FlhC			
ECs_3546	mprA	transcriptional repressor	VFG042569 (gb|WP_001445604)	F1C fimbriae (VF0224)	Adhesin	setA	Escherichia coli CFT073	Adherence	sfaX	HTH-type transcriptional regulator SfaX	36.5	9.20E-31	110
gene-papX	papX	BAA31779.1	VFG042390 (gb|WP_011478152)	S fimbriae (VF0222)	S fimbriae binds to receptors containing sialic acid sugar moieties, the sialic acid residues are presented on UP3, one of four integral membrane uroplakin proteins	setA	Escherichia coli UTI89	Adherence	sfaX	regulatory protein SfaX	47.5	4.39E-18	74.3
ECs_2593	cheB	chemotaxis-specific methylesterase	VFG043090 (gb|WP_000036413)	Peritrichous flagella (VF1154)	-	setA	Escherichia coli O157:H7 str. EDL933	Motility	cheB	protein-glutamate methylesterase/protein glutamine deamidase	99.7	5.26E-245	666
gene-toxB	toxB	Toxin B	VFG012204 (gb|NP_052665)	ToxB (VF0203)	Important for full expression of adherence by affecting the production and secretion of some virulence factors required for the development of A/E lesions	setA	Escherichia coli O157:H7 str. Sakai	Effector delivery system	toxB	cytotoxin	100	0	6194
ECs_3830	rdgB	dITP/XTP pyrophosp	VFG013266 (gb|WP_01	LOS (VF0044)	Major immunogen; LOS phosphorylcholine (ChoP) may	setA	Haemophilus	Immune modulation	orfM	deoxyribonucleotide	58	2.40E-75	224

		hatase	1271949)		influence invasion via interaction with PAF receptor and stimulates of inflammatory signals; LPS phase variation is characterized by the spontaneous loss and gain of oligosaccharide structures present in the outer core. the phase variable expression of LPS biosynthesis genes promotes evasion of antigen-specific host immune defences and allow colonization of different host microenvironments		influenzae 86-028NP			triphosphate pyrophosphatase			
ECs_2438	katE	catalase HPII	VFG037029 (gb|WP_010951343)	KatA (VF0454)	Detoxifies H2O2; Protects against ROS (Reactive oxygen species), a family of chemical that are oxidized version of molecular oxygen, including hydrogen peroxide, superoxide and hydroxyl radicals	setA	Neisseria gonorrhoeae FA 1090	Stress survival	katA	catalase	41.8	7.20E-118	364
ECs_4125	acuI	acryloyl-CoA reductase	VFG029822 (gb|WP_011891406)	GPL locus (VF0841)	-	setA	Mycobacterium gilvum PYR-GCK	Immune modulation	pks	type I polyketide synthase	31.3	1.49E-08	56.2
ECs_1027	ycbV	fimbrial-like adhesin protein	VFG042403 (gb|WP_000919494)	E.coli laminin-binding fimbriae (ELF)	-	setA	Escherichia coli O157:H7 str. EDL933	Adherence	ycbV	type 1 fimbrial protein	99.4	3.51E-120	337

				(VF1140)									
ECs_1026	ycbU	fimbrial protein	VFG042402 (gb|WP_077626202)	E.coli laminin-binding fimbriae (ELF) (VF1140)	-	setA	Escherichia coli O157:H7 str. EDL933	Adherence	ycbU	fimbrial protein	100	5.84E-110	310
ECs_1025	elfG	fimbrial-like adhesin protein	VFG042401 (gb|WP_001165677)	E.coli laminin-binding fimbriae (ELF) (VF1140)	-	setA	Escherichia coli O157:H7 str. EDL933	Adherence	elfG	fimbrial protein	100	2.93E-244	662
ECs_1022	elfD	periplasmic pilin chaperone	VFG042399 (gb|WP_001303867)	E.coli laminin-binding fimbriae (ELF) (VF1140)	-	setA	Escherichia coli O157:H7 str. EDL933	Adherence	elfD	molecular chaperone	100	4.10E-168	462
ECs_1021	elfA	laminin-binding fimbrin subunit	VFG042398 (gb|WP_000750295)	E.coli laminin-binding fimbriae (ELF) (VF1140)	-	setA	Escherichia coli O157:H7 str. EDL933	Adherence	elfA	fimbrial protein	100	2.76E-119	334
ECs_4778	hemG	protoporp	VFG013201	Heme	-	setB	Haemophilu	Nutritiona	hemG	menaquinon	45.8	8.60E-	168

		hyrinogen oxidase	(gb|WP_012341283)	biosynthesis (VF0758)			s somnus 2336	l/Metabolic factor		e-dependent protoporphyrinogen IX dehydrogenase		54	
ECs_1447	murJ	lipid II flippase	VFG016412 (gb|WP_000720341)	Polysaccharide capsule (VF0659)	-	setB	Bacillus thuringiensis serovar konkukian str. 97-27	Immune modulation	murJ	murein biosynthesis integral membrane protein MurJ	24.2	7.89E-28	116
ECs_1449	flgM	anti-sigma factor for FliA	VFG043020 (gb|NP_460143)	Peritrichous flagella (VF0967)	-	setA	Salmonella enterica subsp. enterica serovar Typhimurium str. LT2	Motility	flgM	anti-sigma-28 factor FlgM	82.3	6.42E-45	140
ECs_1448	flgN	flagella synthesis protein FlgN	VFG043072 (gb|WP_000197360)	Peritrichous flagella (VF1154)	-	setA	Escherichia coli O157:H7 str. EDL933	Motility	flgN	flagella biosynthesis chaperone FlgN	100	2.49E-93	265
ECs_1152	torA_1	biotin sulfoxide	VFG026753 (gb|WP_01	Nitrate reductase	Nitrate respiration helps the bacteria to survive in O2-depleted areas of	setA	Mycobacterium	Nutritional/Metaboli	narG	nitrate reductase	37	8.05E-08	56.2

		reductase	5302870)	(VF0302)	inflammatory or necrotic tissue		canettii CIPT 140070008	c factor		subunit alpha			
ECs_0319	ecpE	ECP production pilus chaperone	VFG002417 (gb|WP_001301550)	ECP (VF0404)	ECP, composed of a 21-kDa pilin subunit EspA, is a pilus-adherence factor that is crucial to the virulence of E. coli O157 in humans, and is also carried by commensal strains of E. coli.; It is suggested that pathogenic E. coli strains may use ECP to mimic commensal E. coli and provide themselves with an ecological advantage for host colonization and evasion of the immune system.	setA	Escherichia coli O157:H7 str. EDL933	Adherence	yagV/ecpE	E. coli common pilus chaperone EcpE	100	1.44E-174	479
ECs_4498	waaF	heptosyltransferase II	VFG013400 (gb|WP_005693429)	LOS (VF0044)	Major immunogen; LOS phosphorylcholine (ChoP) may influence invasion via interaction with PAF receptor and stimulates of inflammatory signals; LPS phase variation is characterized by the spontaneous loss and gain of oligosaccharide structures present in the outer core. the phase variable expression of LPS biosynthesis genes promotes evasion of antigen-specific host immune defences and allow	setA	Haemophilus influenzae Rd KW20	Immune modulation	rfaF	ADP-heptose-LPS heptosyltransferase II	63.2	3.29E-152	431

					colonization of different host microenvironments							
ECs_4495	kbl	glycine C-acetyltransferase	VFG026063 (gb|WP_006484929)	Capsule I (VF0436)	A key virulence determinant and that loss of capsule production results in severe attenuation in animal models of disease	setA	Burkholderia cenocepacia J2315	Immune modulation	wcbT	acyl-CoA transferase	36.5	9.52E-68	221
ECs_4497	hldD	ADP-L-glycero-D-mannoheptose-6-epimerase	VFG000332 (gb|WP_005632797)	LOS (VF0044)	Major immunogen; LOS phosphorylcholine (ChoP) may influence invasion via interaction with PAF receptor and stimulates of inflammatory signals; LPS phase variation is characterized by the spontaneous loss and gain of oligosaccharide structures present in the outer core. the phase variable expression of LPS biosynthesis genes promotes evasion of antigen-specific host immune defences and allow colonization of different host microenvironments	setA	Haemophilus influenzae Rd KW20	Immune modulation	rfaD	ADP-L-glycero-D-mannoheptose-6-epimerase	78.2	3.24E-180	499
ECs_4496	yibB	hypothetical protein	VFG018015 (gb|WP_012006735)	LOS (VF0326)	LOS diversity is important for the ability to colonize a wide variety of hosts and intestinal niches; the ability to generate variation at high frequency, the molecular mimicry evident in LOS structure support a role	setB	Campylobacter jejuni subsp. jejuni 81116	Immune modulation	yibB	protein YibB	27.8	2.04E-23	97.4

					in the avoidance of host defences; the similarity of LOS structures to host gangliosides and the subsequent ability to generate crossreacting antibodies forms the pathological basis for the association of preceding C. jejuni infection with Guillain-Barre syndrome								
ECs_4493	waaH	LPS(HepIII)-glucuronic acid glycosyltransferase	VFG006083 (gb|WP_011681443)	Capsule (VF0144)	Resistant to complement deposition and masks cell wall-associated complement from being recognized by the complement receptors on phagocytes	setA	Streptococcus thermophilus LMD-9	Immune modulation	STER_RS07090	glycosyltransferase	28.3	9.02E-29	113
ECs_0954	ybjS	NAD(P)H-dependent oxidoreductase	VFG045309 (gb|WP_010946498)	LPS (VF0171)	Phase variation associated with changes in virulence	setA	Legionella pneumophila subsp. pneumophila str. Philadelphia 1	Immune modulation	LPG_RS03775	NAD-dependent epimerase/dehydratase family protein	22.9	6.25E-12	65.5
ECs_0772	tolQ	colicin transporter TolQ	VFG036648 (gb|WP_003706999)	Ton system (VF0895)	-	setB	Neisseria lactamica 020-06	Nutritional/Metabolic factor	exbB	TonB-system energizer ExbB	30.9	1.96E-20	85.9
ECs_3890	exbB	biopolymer	VFG036648 (gb|WP_00	Ton system	-	setB	Neisseria lactamica	Nutritional/Metaboli	exbB	TonB-system energizer	31.8	1.41E-18	81.3

		transport protein ExbB	3706999)	(VF0895)			020-06	c factor		ExbB			
ECs_0776	pal	peptidoglycan-associated outer membrane lipoprotein	VFG039074 (gb|WP_012880578)	OmpA (VF0499)	Binds to <alpha>2,3-sialic acid of sialyl Lewis x, this receptor-ligand interaction is required for efficient cellular entry	setA	Anaplasma centrale str. Israel	Invasion	ompA	outer membrane protein OmpA	39.4	1.17E-18	79.3
ECs_4275	glgC	glucose-1-phosphate adenylyltransferase	VFG005874 (gb|WP_002986125)	Capsule (VF0144)	Resistant to complement deposition and masks cell wall-associated complement from being recognized by the complement receptors on phagocytes	setA	Streptococcus pyogenes MGAS10750	Immune modulation	galU	UTP--glucose-1-phosphate uridylyltransferase GalU	24.1	4.25E-10	60.5
ECs_3549	luxS	S-ribosylhomocysteine lyase	VFG018243 (gb|WP_005462534)	AI-2 (VF0406)	LuxPQ receptor complex responds to the AI-2 molecule. Information from AI-2 is transduced through the LuxO protein to control the levels of the master transcription factor HapR. At low cell density, in the absence of autoinducers, HapR is not produced, so virulence factors are expressed and biofilms are formed. At high cell density, in the presence of	setA	Vibrio parahaemolyticus RIMD 2210633	Biofilm	luxS	S-ribosylhomocysteinase	75.4	1.05E-92	266

ECs_3822	rsmE	16S rRNA m(3)U1498 methyltransferase	VFG045607 (gb|WP_010948621)	Dot/Icm T4SS secreted effectors (VF0798)	autoinducers, LuxO is inactivated, HapR is produced, and it represses genes for virulence factor production and biofilm formation. These events are proposed to allow V. cholerae to leave the host, re-enter the environment in large numbers and initiate a new cycle of infection. AnkB/legAU13/ceg27 (E3 Ubiquitin Ligase Activity, bounds Skp1, targets host protein parvin B.); AnkC/legA12 (Ankyrin repeat.); AnkD/legA15 (Ankyrin repeat.); AnkF/legA14/ceg31 (Ankyrin repeat.); AnkG/ankZ/legA7 (Ankyrin repeat.); AnkH/legA3/ankW (Ankyrin repeat, NF-<kappa>B inhibitor.); AnkI/legAS4 (Ankyrin repeat.); AnkJ/legA11 (Ankyrin repeat.); AnkK/legA5 (Ankyrin repeat.); AnkN/ankX/legA8 (Phosphocholination of Rab1 and Rab35 to regulate their activity; modulation of endosomal trafficking.); AnkQ/legA10; AnkY/legA9 (Ankyrin repeat, STPK, Enhancer of autophagy.); Ceg10; Ceg14/sidL	setA	Legionella pneumophila subsp. pneumophila str. Philadelphia 1	Effector delivery system	LPG_RS14840	Dot/Icm type IV secretion system effector	48.3	9.08E-74	224

(Inhibition of host protein synthesis leading to activation of the NF-<kappa>B pathway.); Ceg15; Ceg17; Ceg18; Ceg19 (Vesicle trafficking.); Ceg23; Ceg25; Ceg28; Ceg29; Ceg3; Ceg30; Ceg32/sidI (Interacts with eEF1A to inhibit host protein synthesis.); Ceg33; Ceg34; Ceg4; Ceg5; Ceg7; Ceg8; Ceg9 (Vesicle trafficking.); CegC1 (Zinc metallophospholipase C. Zinc metallophospholipase C.); CegC2 (Ninein domain.); CegC3; CegC4; DrrA/sidM (Rab1-GEF and GDF (RabGDI displacement factor) activity responsible for Rab1 recruitment to LCV, C-terminal PI4P binding domain responsible for membrane binding; N-terminal AMPylation activity.); LaiE (SidE paralog.); LegA1; LegA2; LegA6; LegA7; LegC1; LegC3/ppeA (Vesicle trafficking.); LegC4 (Coiled-coil.); LegC6 (Coiled-coil.); LegD1; LegD2; LegG2 (Ras GEF.); LegK1 (Eukaryotic-like Ser/Thr kinase activity, directly

activates NF-<kappa>B pathway by phosphorylating the I<kappa>B family of inhibitors.); LegK2 (Ser/Thr kinase.); LegK3 (STPK.); LegL1 (Leucine-rich repeats.); LegL2 (Leucine-rich repeats.); LegL3 (Leucine-rich repeats.); LegL5 (Leucine-rich repeats.); LegL6 (Leucine-rich repeats.); LegL7 (Leucine-rich repeats.); LegLC4 (Leucine-rich repeats, coiled-coil.); LegLC8 (Leucine-rich repeats, coiled-coil.); LegN; LegP (Astacin protease.); LegS1; LegS2 (Putative Sphingosine-1-phosphate lyase 1 (SP-lyase).); LegT (Thaumatin domain.); LegU1 (E3 Ubiquitin Ligase, targets host chaperone protein BAT3.); LegY; Lem1; Lem10; Lem11; Lem12; Lem14; Lem15; Lem16; Lem17; Lem19; Lem2; Lem20; Lem21; Lem22; Lem23; Lem24; Lem25; Lem26; Lem27; Lem28; Lem29; Lem3 (Dephosphoryl-cholinase relieving the AnkX-mediated modification on Rab1.); Lem4/smdA (PI4P-binding protein.);

Lem5; Lem6; Lem7; Lem8; Lem9; LepA (Nonlytic release from protozoa.); LepB (Rab1 GAP, vesicle trafficking and bacterial egress.); Lgt2/legC8 (Glucosyltransferase, inhibits host protein systhesis by glucosylating mammalian elongation factor eEF1A at serine-53.); Lgt3/legC5 (Glucosyltransferase, inhibits host protein systhesis by glucosylating mammalian elongation factor eEF1A at serine-53.); LidA (Promotion of Rab1 recruitment and tethering of ER derived vesicles to the LCV; stabilization of Rab guanosine nucleotide complex.); LidL (EnhC paralogue.); LirA; LirB (Peptidyl-prolyl cis-trans isomerase A (rotamase A).); Lpg0045; Lpg0081; Lpg0294; Lpg0365; Lpg0518; Lpg0634; Lpg0963; Lpg1148; Lpg1158; Lpg1273; Lpg1689; Lpg1717; Lpg1751; Lpg2160 (Associates with BAT3 independently of LegU1; LegU1 and Lpg2160 may function redundantly or in concert to modulate BAT3 activity during the course of

infection.); Lpg2327; Lpg2407; Lpg2525 (F-box protein.); Lpg2527; Lpg2744; LpnE (Putative Beta-lactamase.); LubX/legU2 (E3 ubiquitin ligase, targets another effector protein SidH to proteasome-mediated protein degradation in the host cells; cell cycle modulation via Clk1.); MavA; MavB; MavC; MavE; MavF; MavG; MavH; MavI; MavJ; MavL; MavM; MavN; MavV; PieA/lirC; PieB/lirD; PieC/lirE; PieD/lirF; PieE; PieF; PieG/legG1 (Regulator of chromosome condensation RCC.); PpeB; PpgA (Regulator of chromosome condensation.); RalF (Arf-GEF; Arf1 recuitment to LCV.); RavE; RavF; RavG; RavH; RavI; RavJ; RavK; RavL; RavM; RavN; RavO; RavP; RavQ; RavR; RavS; RavT; RavW; RavX; RavY; RavZ (Cysteine protease. Inhibits host autophagy by cleaving and deconjugating LC3-PE.); RvfA; SdbA (Contributes to sustained NF-<kappa>B activation.); SdbB (SidB paralog.); SdbC (SidB paralog.); SdcA

(SidC paralog, anchors to PtdIns(4)P on LCVs.); SdeA/laiA (Adherence and/or uptake.); SdeB/laiB; SdeC/laiC; SdeD/laiF (SidE paralog.); SdhA (Maintenance of LCV integrity preventing cell death and type I interferon induction.); SdhB (Paralog of sidH, ANTH domain.); SdjA; SetA (Vesicle trafficking.); SidA; SidB (Rtx toxin, lipase.); SidC (ER recuitment.); SidD; SidE/laiD; SidF (Anti-apoptosis by targeting pro-death members of the Bcl2 protein family.); SidG (Coiled-coil.); SidH (A substrate of LubX E3 ubiquitin ligase.); SidJ (ER recuitment.); SidK (Interacting with VatA, a key component of the proton pump. Inhibition of LCV acidification.); VipA (Actin nucleator contributing to modulate organelle trafficking.); VipD (Phospholipase A1, removes PI(3)P from early endosomes.); VipE; VipF (N-terminal acetyltransferase, GNAT family.); VpdA/vipD2 (VipD paralog, Acyl transferase/acyl

hydrolase/lysophospholipase.);
VpdB/vipD3 (VipD paralog,
phospholipase.); VpdC; WipA; WipB;
YlfA/legC7 (Vesicle trafficking.);
YlfB/legC2 (Vesicle trafficking.);
CegC4; MesI; LPG_RS00040;
LPG_RS00105; LPG_RS00150;
LPG_RS00200; LPG_RS00235;
LPG_RS00300; LPG_RS00665
(PI-3-phosphatase.); LPG_RS00825;
LPG_RS00870; LPG_RS00880;
LPG_RS00925; LPG_RS01290;
LPG_RS01305; LPG_RS01820;
LPG_RS01880; LPG_RS02025;
LPG_RS03550; LPG_RS03935;
LPG_RS04800; LPG_RS05365;
LPG_RS05490; LPG_RS05590;
LPG_RS05660; LPG_RS05710;
LPG_RS05830; LPG_RS07260;
LPG_RS07280; LPG_RS07435;
LPG_RS07905; LPG_RS08210;
LPG_RS08285; LPG_RS08320;
LPG_RS08345; LPG_RS08350;
LPG_RS08370; LPG_RS08445;
LPG_RS08450; LPG_RS08485;
LPG_RS08595; LPG_RS08775;

					LPG_RS08895; LPG_RS09040; LPG_RS09470; LPG_RS09565; LPG_RS09650; LPG_RS09825; LPG_RS09915; LPG_RS09965; LPG_RS10290; LPG_RS10795; LPG_RS10800; LPG_RS11170; LPG_RS11255; LPG_RS11415; LPG_RS11860; LPG_RS11920; LPG_RS11940; LPG_RS11990; LPG_RS12190; LPG_RS12265; LPG_RS12310; LPG_RS12405; LPG_RS12815; LPG_RS12820; LPG_RS12830; LPG_RS12855; LPG_RS12885; LPG_RS12900; LPG_RS13265; LPG_RS13310; LPG_RS13590; LPG_RS13860; LPG_RS14265; LPG_RS14285; LPG_RS14345; LPG_RS14500; LPG_RS14525; LPG_RS14550; LPG_RS14555; LPG_RS14570; LPG_RS14710; LPG_RS14840; LPG_RS15050; LPG_RS15170								
ECs_4932	aceB	malate synthase A	VFG043448 (gb|NP_216353)	Rv1837c (VF0864)	-	setB	Mycobacterium tuberculosis H37Rv	Adherence	glcB	malate synthase	22.4	7.90E-08	55.1

ECs_0178	rseP	metallopeptidase	VFG015004 (gb|WP_004406254)	Alginate (VF0091)	Allows the bacteria form biofilm; contributes to the persistence of the bacteria in the CF lung: act as an adhesin, preventing the bacteria from being expelled from the lung, and alginate slime layer makes it more difficult for phagocytes to ingest and kill the bacteria	setA	Pseudomonas syringae pv. syringae B728a	Biofilm	mucP	metalloprotease protease	48.1	3.13E-137	401
ECs_0176	ispU	undecaprenyl pyrophosphate synthase	VFG045688 (gb|WP_002294134)	Capsule (VF0361)	Contributes to host immune evasion	setA	Enterococcus faecium Aus0004	Immune modulation	cpsA/uppS	undecaprenyl diphosphate synthase	46.1	2.99E-70	216
ECs_2936	yehT	two-component regulatory system response regulator YehT	VFG014966 (gb|WP_007247212)	Alginate (VF0091)	Allows the bacteria form biofilm; contributes to the persistence of the bacteria in the CF lung: act as an adhesin, preventing the bacteria from being expelled from the lung, and alginate slime layer makes it more difficult for phagocytes to ingest and kill the bacteria	setA	Pseudomonas syringae pv. tomato str. DC3000	Biofilm	algR	alginate biosynthesis regulatory protein AlgR	33.3	1.75E-26	102
ECs_3157	elaD	protease	VFG003978 (gb|WP_001017745)	TTSS-2 secreted effectors (VF0947)	GogB; PipB; PipB2 (Directly binds kinesin, a plus end-directed microtubule motor protein. Localizes to the phagosome; Promotes Sif extension, recuits kinesin-1 to	setA	Salmonella enterica subsp. enterica serovar	Effector delivery system	sseL	type III secretion system effector SseL,	27.3	4.00E-28	113

SCV.); SifA (GEF. Localizes to the cytosolic face of the SCV and binds to host protein-kinesin-binding protein SKIP to modulate phagosome tubulation; required for SCV membrane integrity and Sif formation; maintains perinuclear SCV positioning; redirects exocytic vesicles to SCV.); SifB (GEF, WxxxE family.); SopD2 (Inhibits vesicle transport from the vacuole; down-modulates the formation of tubular structures.); SpiC/ssaB (SsaL/ssaM/spiC regulatory complex, sensing host cell signals to activate effector secretion.); SpvB (ADP-ribosyltransferase. Causes cytoskeletal disruption and apoptosis.); SpvC; SpvD; SseF (Localizes to the phagosome, contributes to Sif formation; recruits dynein to SCV; maintains perinuclear SCV positioning; required for formation of microtubule bundles around SCV; redirects exocytic transport vesicles to SCV.); SseG (Same as SseF.); SseI/srfH (Blocks the	Typhi str. CT18	deubiquitinase

					migration of host immune cells by associating with host protein IQGAP1, an important regulator of cell migration; attenuates the host's ability to clear systemic bacteria.); SseJ (Glycerophospholipid-cholesterol acyl transferase and deacylase. SseJ and SifA interact in a protein complex with SKIP and RhoA family GTPases as a mechanism to promote phagosome tubulation.); SseK1; SseK2; SseL (Deubiquitinase. Required for Salmonella-induced delayed cytotoxicity of macrophages.); SspH2 (Novel E3 ubiquitin-ligase. Specifically localizes to the brush border of polorized epithelial cells.); SteC (Serine/Threonine kinase. Required for SPI-2 dependent F-actin remodelling.)								
ECs_3153	menF	isochorismate synthase 2	VFG016150 (gb|WP_011061768)	Pyochelin (VF0095)	Effective at promoting iron uptake in P. aeruginosa; catalyzes the formation of tissue-damaging free radicals; pyochelin also binds other transition metals (e.g. Mo(IV), Co(II)) with	setA	Pseudomonas fluorescens Pf-5	Nutritional/Metabolic factor	pchA	salicylate biosynthesis isochorismate	34.2	1.54E-41	153

					appreciable affinity and is implicated in the delivery of both Co(II) and Mo(IV) to P. aeruginosa cells					synthase PchA			
ECs_2826	hisF	imidazole glycerol phosphate synthase catalytic subunit	VFG045297 (gb|WP_01 1213307)	LPS (VF0171)	Phase variation associated with changes in virulence	setA	Legionella pneumophila subsp. pneumophila str. Philadelphia 1	Immune modulation	wbuZ	glycosyl amidation-associated protein WbuZ	36.4	1.30E-52	171
ECs_2825	hisA	N-(5'-phospho-L-ribosyl-formimino)-5-amino-1-(5'-phosphoribosyl)-4-imidazolecarboxamide isomerase	VFG045297 (gb|WP_01 1213307)	LPS (VF0171)	Phase variation associated with changes in virulence	setA	Legionella pneumophila subsp. pneumophila str. Philadelphia 1	Immune modulation	wbuZ	glycosyl amidation-associated protein WbuZ	27.6	9.62E-20	85.1
ECs_2824	hisH	imidazole glycerol phosphate synthase glutamine	VFG014106 (gb|NP_25 1842)	LPS (VF0085)	Mediates biological effects including resistance to serum killing and phagocytosis; the binding to normal CFTR (cystic fibrosis transmembrane conductance regulator) and invasion of	setA	Pseudomonas aeruginosa PAO1	Immune modulation	hisH2	imidazole glycerol phosphate synthase subunit	42.8	2.81E-45	148

		amidotransferase subunit			host cells may make a contribution to virulence in the human eye; internalization by binding to normal CFTR protein expressed by airway epithelial cells followed by desquamation of bacteria-laden epithelial cells, constitutes a host defense mechanism. If this mechanism fails to function properly, abnormally high bacterial carriage would promote the establishment of chronic bacterial infection					HisH			
ECs_2332	ydgJ	oxidoreductase	VFG000038 (gb|WP_003807115)	LPS (VF0033)	Prevents clearance of the organism by host surfactant protein; confers protection to the bacterium from complement-mediated cell lysis	setA	Bordetella pertussis Tohama I	Immune modulation	bplA	probable oxidoreductase	22.2	1.16E-08	55.8
ECs_3553	csrA	pleiotropic regulatory protein for carbon source metabolism	VFG010906 (gb|WP_011213314)	CsrA (VF0261)	Post-transcriptional repression of the transmission regulon	setA	Legionella pneumophila subsp. pneumophila str. Philadelphia 1	Regulation	csrA	carbon storage regulator CsrA	76.7	6.66E-27	92
ECs_3554	alaS	alanyl-tR	VFG015881	Phytotoxi	-	setB	Pseudomona	Exotoxin	PSPTO_RS24	alanyl-tRN	29.7	1.00E-	50.4

		NA synthetase	(gb|WP_005888845)	n coronatine (VF0916)			s syringae pv. tomato str. DC3000		365	A editing protein		06	
ECs_3558	mltB	membrane-bound lytic murein transglycosylase B	VFG015347 (gb|WP_005763248)	P. syringae TTSS effectors (VF0911)	-	setB	Pseudomonas syringae pv. tomato str. DC3000	Effector delivery system	hopAJ2	lytic murein transglycosylase	33.2	1.93E-30	121
ECs_3697	ygeA	aspartate/glutamate racemase	VFG011805 (gb|WP_011732196)	LOS (VF0326)	LOS diversity is important for the ability to colonize a wide variety of hosts and intestinal niches; the ability to generate variation at high frequency, the molecular mimicry evident in LOS structure support a role in the avoidance of host defences; the similarity of LOS structures to host gangliosides and the subsequent ability to generate crossreacting antibodies forms the pathological basis for the association of preceding C. jejuni infection with Guillain-Barre syndrome	setB	Campylobacter fetus subsp. fetus 82-40	Immune modulation	CFF8240_RS06905	aspartate/glutamate racemase family protein	45.7	4.93E-79	236
ECs_3908	qseC	quorum sensing	VFG041841 (gb|WP_01	Cpi-1a + Cpi-1	-	setA	Chromobacterium	Effector delivery	CV_RS12895	two-component sensor	37.7	1.27E-28	116

		sensory histidine kinase in two-component regulatory system with QseB	1136181)	(SPI-1 like) (VF1259)			violaceum ATCC 12472	system		histidine kinase			
ECs_4884	eptC	LPS heptose I phosphoethanolamine transferase	VFG034688 (gb|NP_312911)	Ibes (VF0237)	Contributes to brain microvascular endothelial cells (BMECs) invasion via a ligand-receptor interaction	setA	Escherichia coli O157:H7 str. Sakai	Invasion	ibeC	LPS heptose I phosphoethanolamine transferase	100	0	1168
ECs_5106	dcuR	two-component regulatory system response regulator DcuR	VFG043408 (gb|WP_000940577)	Peritrichous flagella (VF0670)	-	setB	Bacillus cereus ATCC 10987	Motility	BCE_RS08700	response regulator	26.9	2.98E-09	53.5
ECs_4583	escR	T3SS structure protein EscR	VFG000827 (gb|WP_001299599)	TTSS (VF0191)	Injects Tir and other effector molecules directly into the host cell. Effector molecules activate cell-signaling pathways, causing	setA	Escherichia coli O157:H7 str. EDL933	Effector delivery system	escR	Type III secretion systemminor export	100	1.92E-140	391

					alterations in the host cell cytoskeleton and resulting in the depolymerization of actin and the loss of microvilli					apparatus protein EscR		
ECs_4722	rffH	glucose-1-phosphate thymidylyltransferase	VFG048826 (gb|YP_005227857)	Capsule (VF0560)	Assisting in evading the host immune system by protecting bacteria from opsonophagocytosis and serum killing	setA	Klebsiella pneumoniae subsp. pneumoniae HS11286	Immune modulation	KPHS_35570	glucose-1-phosphate thymidylyltransferase	74.8	4.73E-157	439
ECs_3718	ECs_3718	type III secretion protein EprI	VFG042090 (gb|NP_311745)	ETT2 (VF1161)	-	setA	Escherichia coli O157:H7 str. Sakai	Effector delivery system	ECs_3718	type III secretion protein EprI	100	5.99E-53	159
ECs_4721	rfbB	dTDP-glucose 4%2C6-dehydratase	VFG007659 (gb|WP_011261022)	Capsular polysaccharide (VF0624)	-	setA	Vibrio fischeri ES114	Immune modulation	rmlB	dTDP-glucose 4,6-dehydratase	79.5	4.91E-214	589
ECs_5008	pgi	glucosephosphate isomerase	VFG013531 (gb|WP_011272485)	Exopolysaccharide (VF0755)	-	setA	Haemophilus influenzae 86-028NP	Immune modulation	pgi	glucose-6-phosphate isomerase	77.6	0	880
ECs_4676	atpA	F0F1 ATP synthase subunit alpha	VFG041746 (gb|WP_004528905)	TTS2 (VF0703)	-	setA	Burkholderia pseudomallei K96243	Effector delivery system	sctN	type III secretion system ATPase SctN	31.5	4.40E-43	158

ECs_4674	atpD	F0F1 ATP synthase subunit beta	VFG041746 (gb|WP_004528905)	TTS2 (VF0703)	-	setA	Burkholderia pseudomallei K96243	Effector delivery system	sctN	type III secretion system ATPase SctN	29.1	1.21E-45	164
ECs_4671	glmS	L-glutamine:D-fructose-6-phosphate aminotransferase	VFG011222 (gb|WP_012415791)	LPS (VF0033)	Prevents clearance of the organism by host surfactant protein; confers protection to the bacterium from complement-mediated cell lysis	setB	Bordetella avium 197N	Immune modulation	BAV_RS00460	LPS biosynthesis protein	29.2	8.42E-10	61.6
ECs_4672	glmU	bifunctional N-acetylglucosamine-1-phosphate uridyltransferase/glucosamine-1-phosphate	VFG047265 (gb|WP_014715028)	LPS (VF0542)	A key virulence factor does not signal through, and is not an agonist of, toll like receptor 4 (TLR-4) and has little endotoxic activity	setA	Francisella noatunensis subsp. orientalis str. Toba 04	Immune modulation	lpxA/glmU	UDP-N-acetylglucosamine pyrophosphorylase/glucosamine-1-phosphate N-acetyltransferase	50.7	6.70E-152	439
ECs_0787	galE	UDP-galactose-4-epimerase	VFG013286 (gb|WP_005694325)	LOS (VF0044)	Major immunogen; LOS phosphorylcholine (ChoP) may influence invasion via interaction with PAF receptor and stimulates of inflammatory signals; LPS phase	setA	Haemophilus influenzae Rd KW20	Immune modulation	galE	UDP-glucose 4-epimerase	57.7	3.23E-138	395

					variation is characterized by the spontaneous loss and gain of oligosaccharide structures present in the outer core. the phase variable expression of LPS biosynthesis genes promotes evasion of antigen-specific host immune defences and allow colonization of different host microenvironments								
ECs_0542	ECs_0542	hypotheti cal protein	VFG037636 (gb|WP_00 1187640)	Bap (VF0462)	Contributes to biofilm formation and adhesion to eukaryotic host cells	setA	Acinetobac ter baumannii AB0057	Biofilm	bap	biofilm-as sociated protein	29.5	8.31E-151	532
ECs_0541	ECs_0541	hypotheti cal protein	VFG037636 (gb|WP_00 1187640)	Bap (VF0462)	Contributes to biofilm formation and adhesion to eukaryotic host cells	setB	Acinetobac ter baumannii AB0057	Biofilm	bap	biofilm-as sociated protein	30.3	9.69E-67	250
ECs_0544	ECs_0544	membrane fusion protein of a transport system	VFG044152 (gb|WP_01 1063598)	HasA-type hemophore -mediated heme uptake system (VF0940)	-	setA	Pseudomona s fluorescen s Pf-5	Nutritiona l/Metaboli c factor	PFL_RS2727 5	HlyD family type I secretion periplasmi c adaptor subunit	29.6	1.53E-39	146
ECs_3695	lysA	diaminopi melate	VFG026414 (gb|WP_19	Lysine synthesis	-	setB	Mycobacter ium	Nutritiona l/Metaboli	lysA	diaminopim elate	30.5	1.13E-36	139

		decarboxylase	8344965)	(VF0815)			smegmatis JS623	c factor		decarboxylase			
ECs_3308	eutR	transcriptional regulator	VFG041990 (gb|WP_011004170)	T3SS (VF1295)	-	setB	Ralstonia solanacearum GMI1000	Effector delivery system	hrpB	transcriptional regulator HrpB	29	2.83E-09	58.2
ECs_3709	ygeH	transcriptional regulator	VFG042097 (gb|NP_311736)	ETT2 (VF1161)	-	setA	Escherichia coli O157:H7 str. Sakai	Effector delivery system	ygeH	transcriptional regulator	100	0	901
ECs_0162	btuF	vitamin B12 ABC transporter periplasmic binding protein	VFG044078 (gb|YP_002344986)	Direct heme uptake system (VF0724)	-	setB	Campylobacter jejuni subsp. jejuni NCTC 11168	Nutritional/Metabolic factor	chuD	hemin uptake system substrate-binding protein	29.6	2.54E-14	70.9
ECs_4380	ECs_4380	ligand-gated channel protein	VFG012597 (gb|WP_000089593)	Chu (VF0234)	Iron uptake: the ability to use heme and/or hemoglobin might be especially advantageous to pathogenic bacteria. These pathogens often secrete cytotoxins, which gain access to the intracellular heme reservoir besides initiating tissue invasion. Cytotoxin production coupled with the capability	setA	Escherichia coli O157:H7 str. EDL933	Nutritional/Metabolic factor	chuA	Outer membrane heme/hemoglobin receptor ChuA	100	0	1311

					to utilize heme and/or hemoglobin could serve as an effective iron acquisition strategy during the progression of infection								
ECs_2822	hisC	histidinol-phosphate aminotransferase	VFG001965 (gb|YP_002344820)	Capsule (VF0323)	Play an important role in bacterial survival and persistence in the environment and evasion of host immune response; the presence of heptose residues in the capsule may be important for virulence. Heptose residues found in some cell surface-located glycoconjugates are required for adhesion	setA	Campylobacter jejuni subsp. jejuni NCTC 11168	Immune modulation	Cj1437c	aminotransferase	27.2	3.27E-26	107
ECs_3528	ygaV	tributyltin-inducible repressor	VFG050075 (gb|WP_000215714)	PagR-XO1 (VF0664)	-	setB	Bacillus cereus biovar anthracis str. CI	Regulation	pagR-XO1	transcriptional repressor PagR	36.8	1.00E-07	45.8
ECs_0578	allC	allantoate amidohydrolase	VFG049122 (gb|WP_012737539)	Allantion utilization (VF0572)	Providing a nitrogen source to increase virulence in K. pneumoniae at certain sites of infection	setA	Klebsiella pneumoniae subsp. pneumoniae NTUH-K2044	Nutritional/Metabolic factor	allC	allantoate amidohydrolase	80	6.27E-243	666
ECs_2823	hisB	histidinol-phosphatase and	VFG011816 (gb|WP_010404164)	LOS (VF0326)	LOS diversity is important for the ability to colonize a wide variety of hosts and intestinal niches; the	setA	Campylobacter fetus subsp.	Immune modulation	gmhB	D-glycero-beta-D-manno-heptose	33.3	1.47E-21	90.1

		imidazole glycerol-phosphate dehydratase			ability to generate variation at high frequency, the molecular mimicry evident in LOS structure support a role in the avoidance of host defences; the similarity of LOS structures to host gangliosides and the subsequent ability to generate crossreacting antibodies forms the pathological basis for the association of preceding C. jejuni infection with Guillain-Barre syndrome		fetus 82-40			1,7-bisphosphate 7-phosphatase			
ECs_0202	gmhB	D%2CD-heptose 1%2C7-bisphosphate phosphatase	VFG011816 (gb|WP_010404164)	LOS (VF0326)	LOS diversity is important for the ability to colonize a wide variety of hosts and intestinal niches; the ability to generate variation at high frequency, the molecular mimicry evident in LOS structure support a role in the avoidance of host defences; the similarity of LOS structures to host gangliosides and the subsequent ability to generate crossreacting antibodies forms the pathological basis for the association of preceding C. jejuni infection with Guillain-Barre syndrome	setA	Campylobacter fetus subsp. fetus 82-40	Immune modulation	gmhB	D-glycero-beta-D-manno-heptose 1,7-bisphosphate 7-phosphatase	47.1	6.96E-45	146
ECs_1453	flgD	flagellar	VFG043076	Peritrich	-	setA	Escherichi	Motility	flgD	flagellar	99.6	1.46E-	420

		hook assembly protein	(gb|WP_000020482)	ous flagella (VF1154)			a coli O157:H7 str. EDL933			hook assembly protein FlgD		151	
ECs_3639	eno	enolase	VFG005579 (gb|WP_000022815)	Streptococcal enolase (VF1060)	-	setB	Streptococcus pneumoniae D39	Exoenzyme	eno	phosphopyruvate hydratase	58.9	4.64E-166	473
ECs_1450	flgA	assembly protein for flagellar basal-body periplasmic P ring	VFG043073 (gb|WP_000879706)	Peritrichous flagella (VF1154)	-	setA	Escherichia coli O157:H7 str. EDL933	Motility	flgA	flagellar basal body P-ring formation protein FlgA	100	1.87E-148	411
ECs_0975	ftsK	cell division protein FtsK	VFG040864 (gb|WP_001172770)	T7SS (VF0581)	In the pathogen B. anthracis, six WXG100 proteins were discovered by genome analysis: EsxB, EsxL, EsxP, EsxQ, EsxV, and EsxW. It was shown that under laboratory conditions, EsxW and EsxB are actively secreted.	setA	Bacillus anthracis str. Sterne	Effector delivery system	essC	type VII secretion system protein EssC	29.2	1.50E-17	89
ECs_3046	yeiG	S-formylglutathione hydrolase	VFG008175 (gb|WP_011894102)	Antigen 85 (VF0311)	Mycolyltransferase activity that transfer long-chain mycolic acids to trehalose derivatives (i.e., cord factor, a toxic glycolipid consisting	setB	Mycobacterium gilvum PYR-GCK	Adherence	fbpC	Secreted antigen 85-C FbpC (85C)	28.5	5.10E-06	47

					of two mycolic acid molecules linked to trehalose by the hydroxyl groups of carbons 6 and 6' --> trehalose 6,6'-dimycolate (TDM), is an important immunomodulatory component of the cell wall), pivotal for the biosynthesis of the mycobacterial cell wall and for the survival of mycobacteria; binds the cell matrix protein fibronectin thereby enhancing the uptake of mycobacteria macrophages via complement-mediated phagocytosis					(antigen 85 complex C) (AG58C) (mycolyl transferase 85C) (fibronectin-binding protein C)			
ECs_3045	folE	GTP cyclohydrolase I	VFG041115 (gb|WP_004528803)	T6SS-1 (VF0429)	Essential for virulence and plays an important role in the intracellular lifestyle of B. pseudomallei; In B. mallei, the T6SS cluster homologous to B. pseudomallei T6SS-1, is important for actin-based motility, multinucleated giant cell formation, intracellular growth in murine macrophages, and virulence in hamsters	setB	Burkholderia mallei ATCC 23344	Effector delivery system	BMA_RS19520	GTP cyclohydrolase I	28	1.33E-12	64.3
ECs_4570	mpc	regulator Mpc	VFG000814 (gb|WP_001050992)	TTSS (VF0191)	Injects Tir and other effector molecules directly into the host cell. Effector molecules activate cell-signaling pathways, causing alterations in the host cell	setA	Escherichia coli O157:H7 str. EDL933	Effector delivery system	cesL	chaperone for SepL	100	4.82E-81	233

					cytoskeleton and resulting in the depolymerization of actin and the loss of microvilli								
ECs_4057	ftsH	ATP-dependent zinc-metallo protease	VFG031153 (gb|YP_001702897)	Proteasome-associated proteins (VF0847)	-	setB	Mycobacterium abscessus ATCC 19977	Stress survival	mpa	Putative AAA-family ATPase	34.9	3.89E-32	131
ECs_4590	espG	T3SS secreted effector EspG	VFG000833 (gb|WP_001301460)	TTSS secreted effectors (VF1110)	Cif (Deamidase. Induces cytopathic effects of actin stress fiber formation and cell cycle arrest.); EspB (Pore formation, actin disruption, microvilli effacement, anti-phagocytosis.); EspF (Inducing degradation of the aniapoptic protein AbcF2, tight junction disruption, microvilli effacement and elongation, mitochondrial dysfunction, N-WASP activation, SGLT-1 inactivation, pedestal maturation, inhibition of NHE3 activity, membrane remodelling; targets and disrupts the nucleolus late in infection, which is temporally controlled by host mitochondria.); EspFu/tccP (Inducing degradation of the aniapoptic protein AbcF2, tight	setA	Escherichia coli O157:H7 str. EDL933	Effector delivery system	espG	Type III secretion system effector EspG, GTPase activating protein	100	1.04E-274	744

junction disruption, microvilli effacement and elongation, mitochondrial dysfunction, N-WASP activation, SGLT-1 inactivation, pedestal maturation, inhibition of NHE3 activity, membrane remodelling; targets and disrupts the nucleolus late in infection, which is temporally controlled by host mitochondria.); EspG (TBC-like GTPase activating protein. Efficiently catalyzes GTP hydrolysis in Rab1 to disrupt of Rab1-mediated ER-to-Golgi trafficking.); EspH (First bacterial effector acting directly on RhoGEFs, EspH directly binds to the DH-PH domain in RhoGEFs to disrupt RhoGEF-Rho signaling; critical for inhibiting macrophage phagocytosis.); EspJ (Inhibit both IgG- and complement receptor-mediated phagocytosis.); EspK; EspL1; EspL2 (Cysteine protease. Bounds F-actin-aggregating annexin 2 directly to increase annexin 2's ability to aggregate Tir-induced F-actin; block necroptosis and in

flammation.); EspL4; EspM1 (GEF. Activates the RhoA signaling pathway and induce the formation of stress fibres; inhibit pedestal formation and induce tight junction mislocalization.); EspM2 (GEF. Activates the RhoA signaling pathway and induce the formation of stress fibres; inhibit pedestal formation and induce tight junction mislocalization.); EspN; EspO1-1; EspO1-2; EspR1; EspR3; EspR4; EspT (GEF. Activates Rac1 and Cdc42 leading to formation of membrane ruffles and lamellipodia; induces membrane ruffles to facilitate bacterial invasion into non-phagocytic cells in a process involving Rac1 and Wave2.); EspW; EspX1; EspX2; EspX4; EspX5; EspX6; EspX7/nleL (E3 ubiquitin ligase, HECT-like. Modulates pedestal formation.); EspY1; EspY2; EspY3; EspY4; EspY5; Map (GEF. Mimics the host Dbl and catalyses the exchange of GDP for GTP in Cdc42, involved in effacement, SGLT1 inhibition,

formation of filopodia and disruption of mitochondrial function.); NleA/espI (Disruption of tight junctions by inhibition of host cell protein trafficking through COPII-dependent pathways.); NleB1 (Blocks translocation of the p65 and to the host cell nucleus to inhibit NF-<kappa>B pathway, but NleE and NleB act at different points in the NF-<kappa>B signaling pathway.); NleB2 (May also have anti-inflammatory activity.); NleC (Metalloprotease. Zn-dependent endopeptidases that specifically clip and inactivate RelA (p65), thus blocking NF-<kappa>B pathway.); NleD (Metalloprotease. Zn-dependent endopeptidases that specifically clip and inactivate JNK and p38, thus blocking AP-1 pathway.); NleE (PMN tran-epithelial migration; blocks translocation of the p65 to the host cell nucleus by preventing I<kappa>B degradation to inhibit NF-<kappa>B pathway.); NleF; NleG-1; NleG2-2; NleG2-3; NleG2-4; NleG5-1;

NleG5-2; NleG6-1; NleG6-2; NleG6-3; NleG7 (U-box type E3 ubiquitin ligases.); NleG8-2; NleH1 (Ser/Thr protein kinase. Binds directly to a subunit of NF-<kappa>B, the ribosomal protein S3 (RPS3), reducing the nuclear abundance of RPS3 to dampen host transcriptional outputs; interact with Bax inhibitor-1 to block apoptosis.); NleH2 (Putative kinase. Attenuates NF-<kappa>B pathway.); SepZ/espZ (EspZ interacts with CD98 in host cell membranes to promote host cell survival, therefore provide the pathogen with valuable time to colonize efficiently prior to dissemination.); TccP2; Tir (Mimics host immunoreceptor tyrosine-based inhibition motifs (ITIMs), also see helicobacter CagA. EHEC Tir lacks the Nck binding site. Conserved NPY (Asn-Pro-Tyr) motif recruits the adaptor protein IRTKS and/or IRSp53. IRTKS/IRSp53 link Tir and TccP/EspFu, which in turn activates N-WASP; Receptor for intimin; effacement;

					SGLT1 inhibition; recruits SHIP2 to control actin-pedestal morphology; maintains the integrity of the epithelium by keeping the destructive activity of EspG and EspG2 in check.)							
ECs_5271	fimB	type 1 fimbriae regulatory protein FimB	VFG012263 (gb|NP_418732)	Type 1 fimbriae (VF0221)	Makes an important contribution to colonization of the bladder	setA	Escherichia coli str. K-12 substr. MG1655	Adherence	fimB	Type 1 fimbriae Regulatory protein fimB	100	2.73E-152	419
ECs_3950	rpoD	RNA polymerase sigma 70 factor RpoD	VFG026980 (gb|YP_005923858)	SigA (VF0257)	Sigma A interacts with a transcriptional activator WhiB3 to allow the expression of genes necessary for virulence	setA	Mycobacterium tuberculosis RGTB423	Regulation	sigA/rpoV	RNA polymerase sigma factor	60.3	1.83E-91	285
ECs_4733	hemX	uroporphyrinogen III methyltransferase	VFG013206 (gb|WP_041605348)	Heme biosynthesis (VF0758)	-	setB	Haemophilus somnus 2336	Nutritional/Metabolic factor	hemX	uroporphyrinogen-III C-methyltransferase	43.1	9.73E-53	180
ECs_1480	fhuE	ferric-rhodotorulic acid outer membrane transport	VFG044124 (gb|WP_010930941)	Alcaligin (VF0688)	-	setA	Bordetella pertussis Tohama I	Nutritional/Metabolic factor	fauA	TonB-dependent alcaligin siderophore receptor FauA	37.7	4.56E-148	449

ECs_4928	purD	er phosphoribosylglycinamide synthetase phosphoribosylamine-glycine ligase	VFG047576 (gb|WP_012279908)	Purine biosynthesis (VF0559)	Required for bacterial cytosolic replication	setA	Francisella philomiragia subsp. philomiragia ATCC 25017	Nutritional/Metabolic factor	purCD	fusion protein PurC/PurD	35.5	1.82E-69	234
ECs_1001	kdsB	3-deoxy-manno-octulosonate cytidylyltransferase	VFG038845 (gb|WP_016351249)	Polar flagella (VF0473)	Necessary for motility, adhesion and invasion; glycosylation of the flagellin may play a role in provoking a proinflammatory response	setA	Aeromonas hydrophila ML09-119	Motility	nueA	NeuA protein	69.9	7.66E-122	347
ECs_1003	elyC	envelope biogenesis factor	VFG045590 (gb|WP_010948328)	Dot/Icm T4SS secreted effectors (VF0798)	AnkB/legAU13/ceg27 (E3 Ubiquitin Ligase Activity, bounds Skp1, targets host protein parvin B.); AnkC/legA12 (Ankyrin repeat.); AnkD/legA15 (Ankyrin repeat.); AnkF/legA14/ceg31 (Ankyrin repeat.); AnkG/ankZ/legA7 (Ankyrin repeat.); AnkH/legA3/ankW (Ankyrin repeat, NF-<kappa>B inhibitor.); AnkI/legAS4 (Ankyrin repeat.); AnkJ/legA11 (Ankyrin	setA	Legionella pneumophila subsp. pneumophila str. Philadelphia 1	Effector delivery system	LPG_RS13265	Dot/Icm type IV secretion system effector	30.5	1.59E-25	100

repeat.); AnkK/legA5 (Ankyrin repeat.); AnkN/ankX/legA8 (Phosphocholination of Rab1 and Rab35 to regulate their activity; modulation of endosomal trafficking.); AnkQ/legA10; AnkY/legA9 (Ankyrin repeat, STPK, Enhancer of autophagy.); Ceg10; Ceg14/sidL (Inhibition of host protein synthesis leading to activation of the NF-<kappa>B pathway.); Ceg15; Ceg17; Ceg18; Ceg19 (Vesicle trafficking.); Ceg23; Ceg25; Ceg28; Ceg29; Ceg3; Ceg30; Ceg32/sidI (Interacts with eEF1A to inhibit host protein synthesis.); Ceg33; Ceg34; Ceg4; Ceg5; Ceg7; Ceg8; Ceg9 (Vesicle trafficking.); CegC1 (Zinc metallophospholipase C. Zinc metallophospholipase C.); CegC2 (Ninein domain.); CegC3; CegC4; DrrA/sidM (Rab1-GEF and GDF (RabGDI displacement factor) activity responsible for Rab1 recruitment to LCV, C-terminal PI4P binding domain responsible for membrane binding;

N-terminal AMPylation activity.); LaiE (SidE paralog.); LegA1; LegA2; LegA6; LegA7; LegC1; LegC3/ppeA (Vesicle trafficking.); LegC4 (Coiled-coil.); LegC6 (Coiled-coil.); LegD1; LegD2; LegG2 (Ras GEF.); LegK1 (Eukaryotic-like Ser/Thr kinase activity, directly activates NF-<kappa>B pathway by phosphorylating the I<kappa>B family of inhibitors.); LegK2 (Ser/Thr kinase.); LegK3 (STPK.); LegL1 (Leucine-rich repeats.); LegL2 (Leucine-rich repeats.); LegL3 (Leucine-rich repeats.); LegL5 (Leucine-rich repeats.); LegL6 (Leucine-rich repeats.); LegL7 (Leucine-rich repeats.); LegLC4 (Leucine-rich repeats, coiled-coil.); LegLC8 (Leucine-rich repeats, coiled-coil.); LegN; LegP (Astacin protease.); LegS1; LegS2 (Putative Sphingosine-1-phosphate lyase 1 (SP-lyase).); LegT (Thaumatin domain.); LegU1 (E3 Ubiquitin Ligase, targets host chaperone protein

BAT3.); LegY; Lem1; Lem10; Lem11; Lem12; Lem14; Lem15; Lem16; Lem17; Lem19; Lem2; Lem20; Lem21; Lem22; Lem23; Lem24; Lem25; Lem26; Lem27; Lem28; Lem29; Lem3 (Dephosphoryl-cholinase relieving the AnkX-mediated modification on Rab1.); Lem4/smdA (PI4P-binding protein.); Lem5; Lem6; Lem7; Lem8; Lem9; LepA (Nonlytic release from protozoa.); LepB (Rab1 GAP, vesicle trafficking and bacterial egress.); Lgt2/legC8 (Glucosyltransferase, inhibits host protein systhesis by glucosylating mammalian elongation factor eEF1A at serine-53.); Lgt3/legC5 (Glucosyltransferase, inhibits host protein systhesis by glucosylating mammalian elongation factor eEF1A at serine-53.); LidA (Promotion of Rab1 recruitment and tethering of ER derived vesicles to the LCV; stabilization of Rab guanosine nucleotide complex.); LidL (EnhC paralogue.); LirA; LirB (Peptidyl-prolyl cis-trans isomerase

A (rotamase A).); Lpg0045; Lpg0081; Lpg0294; Lpg0365; Lpg0518; Lpg0634; Lpg0963; Lpg1148; Lpg1158; Lpg1273; Lpg1689; Lpg1717; Lpg1751; Lpg2160 (Associates with BAT3 independently of LegU1; LegU1 and Lpg2160 may function redundantly or in concert to modulate BAT3 activity during the course of infection.); Lpg2327; Lpg2407; Lpg2525 (F-box protein.); Lpg2527; Lpg2744; LpnE (Putative Beta-lactamase.); LubX/legU2 (E3 ubiquitin ligase, targets another effector protein SidH to proteasome-mediated protein degradation in the host cells; cell cycle modulation via Clk1.); MavA; MavB; MavC; MavE; MavF; MavG; MavH; MavI; MavJ; MavL; MavM; MavN; MavV; PieA/lirC; PieB/lirD; PieC/lirE; PieD/lirF; PieE; PieF; PieG/legG1 (Regulator of chromosome condensation RCC.); PpeB; PpgA (Regulator of chromosome condensation.); RalF (Arf-GEF; Arf1 recuitment to LCV.); RavE; RavF; RavG; RavH; RavI; RavJ;

RavK; RavL; RavM; RavN; RavO; RavP; RavQ; RavR; RavS; RavT; RavW; RavX; RavY; RavZ (Cysteine protease. Inhibits host autophagy by cleaving and deconjugating LC3-PE.); RvfA; SdbA (Contributes to sustained NF-<kappa>B activation.); SdbB (SidB paralog.); SdbC (SidB paralog.); SdcA (SidC paralog, anchors to PtdIns(4)P on LCVs.); SdeA/laiA (Adherence and/or uptake.); SdeB/laiB; SdeC/laiC; SdeD/laiF (SidE paralog.); SdhA (Maintenance of LCV integrity preventing cell death and type I interferon induction.); SdhB (Paralog of sidH, ANTH domain.); SdjA; SetA (Vesicle trafficking.); SidA; SidB (Rtx toxin, lipase.); SidC (ER recuitment.); SidD; SidE/laiD; SidF (Anti-apoptosis by targeting pro-death members of the Bcl2 protein family.); SidG (Coiled-coil.); SidH (A substrate of LubX E3 ubiquitin ligase.); SidJ (ER recuitment.); SidK (Interacting with VatA, a key component of the proton pump.

Inhibition of LCV acidification.);
VipA (Actin nucleator contributing to modulate organelle trafficking.);
VipD (Phospholipase A1, removes PI(3)P from early endosomes.); VipE; VipF (N-terminal acetyltransferase, GNAT family.); VpdA/vipD2 (VipD paralog, Acyl transferase/acyl hydrolase/lysophospholipase.);
VpdB/vipD3 (VipD paralog, phospholipase.); VpdC; WipA; WipB;
YlfA/legC7 (Vesicle trafficking.);
YlfB/legC2 (Vesicle trafficking.);
CegC4; MesI; LPG_RS00040;
LPG_RS00105; LPG_RS00150;
LPG_RS00200; LPG_RS00235;
LPG_RS00300; LPG_RS00665 (PI-3-phosphatase.); LPG_RS00825;
LPG_RS00870; LPG_RS00880;
LPG_RS00925; LPG_RS01290;
LPG_RS01305; LPG_RS01820;
LPG_RS01880; LPG_RS02025;
LPG_RS03550; LPG_RS03935;
LPG_RS04800; LPG_RS05365;
LPG_RS05490; LPG_RS05590;
LPG_RS05660; LPG_RS05710;

LPG_RS05830; LPG_RS07260;

LPG_RS07280; LPG_RS07435;

LPG_RS07905; LPG_RS08210;

LPG_RS08285; LPG_RS08320;

LPG_RS08345; LPG_RS08350;

LPG_RS08370; LPG_RS08445;

LPG_RS08450; LPG_RS08485;

LPG_RS08595; LPG_RS08775;

LPG_RS08895; LPG_RS09040;

LPG_RS09470; LPG_RS09565;

LPG_RS09650; LPG_RS09825;

LPG_RS09915; LPG_RS09965;

LPG_RS10290; LPG_RS10795;

LPG_RS10800; LPG_RS11170;

LPG_RS11255; LPG_RS11415;

LPG_RS11860; LPG_RS11920;

LPG_RS11940; LPG_RS11990;

LPG_RS12190; LPG_RS12265;

LPG_RS12310; LPG_RS12405;

LPG_RS12815; LPG_RS12820;

LPG_RS12830; LPG_RS12855;

LPG_RS12885; LPG_RS12900;

LPG_RS13265; LPG_RS13310;

LPG_RS13590; LPG_RS13860;

LPG_RS14265; LPG_RS14285;

LPG_RS14345; LPG_RS14500;

					LPG_RS14525; LPG_RS14550; LPG_RS14555; LPG_RS14570; LPG_RS14710; LPG_RS14840; LPG_RS15050; LPG_RS15170								
ECs_0972	cydD	amino acid ABC transporter permease	VFG000365 (gb|WP_002214412)	Yersiniabactin (VF0136)	FyuA/Psn-Irp system uses yersiniabactin, a siderophore that can remove iron from a number of mammalian proteins, due to its extremely high affinity for ferric iron	setA	Yersinia pestis CO92	Nutritional/Metabolic factor	ybtP	yersiniabactin ABC transporter ATP-binding/permease protein YbtP	28.5	6.31E-51	184
ECs_4212	fic	cell filamentation protein Fic	VFG002244 (gb|WP_011181141)	VirB/VirD4 T4SS secreted effectors (VF0682)	BepA (Inhibits endothelial cell's apoptosis.); BepB; BepC (Promotes invasome formation depend on cofilin1.); BepD; BepE (Preserves endothelial cells from fragmentation and blocks defects of dendritic cells migration caused by other Beps.); BepF (Promotes invasome formation depend on cofilin1.); BepG (Triggers invasome-mediated internalization primarily by inhibiting bacterial endocytosis.)	setA	Bartonella henselae str. Houston-1	Effector delivery system	bepC	Bartonella effector protein BepC	30.2	1.07E-20	89
ECs_4211	pabA	aminodeoxychorisma	VFG015515 (gb|NP_25	Pyocyanin (VF0100)	Toxic to bacterial and eukaryotic cells due to the reactive oxygen	setA	Pseudomonas	Nutritional/Metaboli	phzE1	phenazine biosynthes	26.2	3.03E-11	61.2

		te synthase subunit II	2903)		intermediates it generates, such as superoxide radical and hydrogen peroxide		aeruginosa PAO1	c factor		is protein PhzE			
ECs_5017	malE	maltose transport system	VFG030533 (gb|WP_014941453)	Trehalose-recycling ABC transporter (VF0842)	-	setB	Mycobacterium indicus pranii MTCC 9506	Nutritional/Metabolic factor	lpqY	extracellular solute-binding protein	24.9	9.27E-11	63.2

The VFDB is a database of microbial-derived substances called virulence factors that contribute to microbial infection and disease in specific hosts, including bacterial toxins, cell-surface proteins that regulate bacterial adhesion, proteins that protect the bacteria themselves, cell-surface carbohydrates, and hydrolytic enzymes that have bacterial pathogenicity. Higher scores represent higher values and higher homology of the two sequences.
